# Sumo-regulatory SENP2 controls the homeostatic squamous mitosis-differentiation checkpoint

**DOI:** 10.1038/s41419-024-06969-z

**Published:** 2024-08-16

**Authors:** Jesús Galán-Vidal, Lorena García-Gaipo, Rut Molinuevo, Samantha Dias, Alex Tsoi, Javier Gómez-Román, James T. Elder, Helfrid Hochegger, Alberto Gandarillas

**Affiliations:** 1 Cell cycle, Stem Cell Fate and Cancer Laboratory, Institute for Research Marqués de Valdecilla (IDIVAL), 39011 Santander, Spain; 2 Genome Damage and Stability Centre, School of Life Sciences, University of Sussex, Brighton, BN19RQ UK; 3 Department of Dermatology, University of Michigan, Ann Arbor, MI USA; 4 Dermatology Service, Ann Arbor Veterans Affairs Hospital, Ann Arbor, MI USA; 5 Pathology Department, Marqués de Valdecilla University Hospital, Institute of Research Valdecilla (IDIVAL), School of Medicine, University of Cantabria, 39008 Santander, Spain; 6 Institut national de la santé et de la recherche médicale, (INSERM), Délégation Occitanie, 34394 Montpellier, France

**Keywords:** Differentiation, Cancer

## Abstract

Squamous or epidermoid cancer arises in stratified epithelia but also is frequent in the non-epidermoid epithelium of the lung by unclear mechanisms. A poorly studied mitotic checkpoint drives epithelial cells bearing irreparable genetic damage into epidermoid differentiation. We performed an RNA-sequencing gene search to target unknown regulators of this response and selected the SUMO regulatory protein SENP2. Alterations of SENP2 expression have been associated with some types of cancer. We found the protein to be strongly localised to mitotic spindles of freshly isolated human epidermal cells. Primary cells rapidly differentiated after silencing *SENP2* with specific shRNAs. Loss of SENP2 produced in synchronised epithelial cells delays in mitotic entry and exit and defects in chromosomal alignment. The results altogether strongly argue for an essential role of SENP2 in the mitotic spindle and hence in controlling differentiation. In addition, the expression of SENP2 displayed an inverse correlation with the immuno-checkpoint biomarker PD-L1 in a pilot collection of aggressive lung carcinomas. Consistently, metastatic head and neck cancer cells that do not respond to the mitosis-differentiation checkpoint were resistant to depletion of SENP2. Our results identify SENP2 as a novel regulator of the epithelial mitosis-differentiation checkpoint and a potential biomarker in epithelial cancer.

## Introduction

The deadliest forms of cancer arise in self-renewing tissues and most commonly in epithelia. Aggressiveness of cancer cells mostly depends on genomic instability often caused by carcinogens [1]. Self-renewing epithelia are continuously exposed to genetic hazard and therefore need robust mechanisms to maintain genomic stability. A still largely unexplored DNA damage-induced differentiation response has been found in some cell types and might be an important homeostatic mechanism, barrier to genomic instability [e.g., 2, 3]. A mitosis-mediated DNA damage-differentiation response suppresses epithelial proliferation upon irreparable DNA damage [4–7]. This response occurs in cells of the squamous epithelia of the skin and head and neck and in the lung, where it promotes squamous metaplasia [8]. This epithelial mitosis-differentiation checkpoint (MDC) maintains the proliferation/differentiation balance while discarding keratinocytes bearing pre-cancerous mutations via desquamation [9–11]. Alterations in the MDC contribute to cancer progression [12]. However, the molecular mechanisms controlling the MDC are poorly understood.

Aiming to find key molecules involved in the control of the MDC and potentially in cancer progression, we performed genetic screens by targeting the early differentiation responsive signal. One of the candidate regulators we identified was SUMO specific peptidase 2 (SENP2). SENP family of proteins belong to the SUMOylation pathways that covalently modify proteins through binding a SUMO (*Small ubiquitin-like modifier*) group [13]. SUMOylation has critical roles during cell cycle regulation, terminal differentiation, or DNA damage response. The SUMO family includes 4 identified members. SUMO-2 and 3 display ∼95% sequence similarity, leading some authors to refer to them as SUMO-2/3 [14]. However, only SUMO-2 deficiency results in lethality during embryo development, indicating that it has unique specific functions [15, 16]. Despite the important functions of SUMOylation, members of the SENP family have been scarcely studied.

SUMOylation occurs through an enzymatic cascade executed by an activating enzyme (E1), a conjugating enzyme (E2) and a SUMO ligase (E3). SUMO maturation and deSUMOylation activities rely on SENPs proteases (*Sentrin-specific proteases*). SENP family members are SENP1, 2, 3, 5, 6 and 7, which differ in location and substrate affinity. SUMO-2/3 can be processed by all SENP family members, but SUMO-1 can only be processed by SENP1 and SENP2 [13, 17].

The function of SENP proteins and in particular SENP2 is poorly understood. SENP2 regulates SUMO availability and its half-time attached to a target protein. SUMOylation is a widely spread post-translational modification which can regulate protein activity, location, or degradation [13, 18]. Specific SUMOylation patterns have been identified during terminal differentiation of T cells, osteocytes, neuros, oocytes, or blastocytes [19, 20]. Within the SENP family, only depletion of either SENP1, or SENP2 or SENP3 is lethal at the foetus stage, therefore indicating their important role in development [19, 21, 22]. In addition, it is intriguing that although the lack of SUMO-1 can be compensated for in vivo [15], the only two proteases known to control SUMO-1 function (SENP1 and SENP2) are essential. This, points out unique functions of SENP1 and SENP2 independent of SUMO-1.

To determine whether SENP2 is a regulator of the epithelial MDC, we made use of shRNAs to silence the gene in primary human epithelial cells. We also investigated its role in mitosis control. The results indicate that SENP2 critically controls human epithelial mitosis and differentiation. In addition, we studied the expression of the protein in a pilot collection of lung squamous carcinoma biopsies in situ. Interestingly, we found a negative correlation between expression of SENP2 and the immuno-checkpoint protein PD-L1. Moreover, and contrary to the rapid differentiation response following SENP2 depletion in non-transformed epithelial cells, aggressive carcinoma cells were resistant to loss of SENP2. Therefore, SENP2 is a candidate to control the epidermoid mitosis-differentiation response and a potential biomarker for aggressiveness of squamous cancer. Our results provide new insight into the mechanisms maintaining epithelial genomic stability.

## Results

We designed a RNA sequencing (RNAseq) assay to identify differentially expressed genes (DEGs) early during the response signal to differentiation, potentially involved in the MDC control. To this end, we induced MDC activation in human primary epidermal keratinocytes by four different stimuli: i- inducing replication stress by an oncogenic mutation (knock-down of p53; [23]); ii- direct DNA damage by the genotoxic Doxorubicin (DOXO); iii- blocking mitosis by inhibition of Aurora Kinase B (specific inhibitor ZM44739, ZM) or by iv- inhibition of Polo like Kinase (Plk-1) (specific inhibitor BI2536, BI). It is worth noting that although ZM and BI both act during mitosis, their effects are slightly different. While Aurora B inhibition induces high levels of polyploidy, this effect is not so drastic when Plk-1 is inhibited. By combining the results after the different treatments, we hoped not to select genes merely involved in polyploidisation, the cell cycle or differentiation, as the four treatments all have in common a rapid trigger of terminal differentiation [23, 24]. We set the time of induction of the differentiation signal for DOXO; ZM and BI at 16 hours, just before the first differentiation markers are detected [24]. We harvested p53-silenced cells 30 hours after delivery of the shRNA, as this is the time we identified for the differentiation signal [23].

**Fig. 1:**
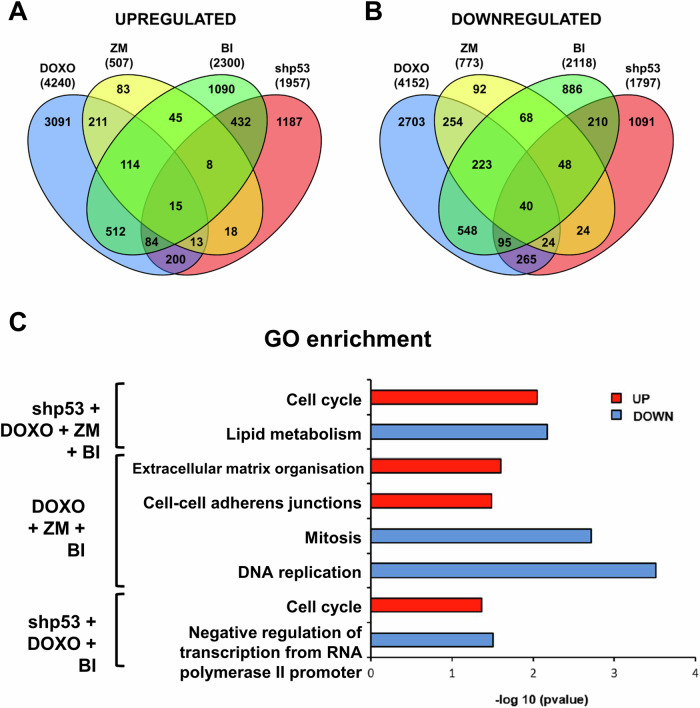
Analyses of DEGs common along treatments and biological processes. **A**, **B** Venn diagrams of up-regulated or down-regulated DEGs after the treatments with doxorubicin (DOXO, blue), Aurora B inhibition (ZM, yellow) or Plk-1 inhibition (BI, green) for 16 hours and infection with shRNA against p53 (shp53, red) 30 hours later. Numbers indicate the amount of DEGs in each combination. **C** Bar histogram representing *p* values (-log_10_) of the most significant GO terms enriched by commonly up-regulated (red bars) or down-regulated (blue bars) DEGs for the treatments indicated.

For each treatment, we selected DEGs presenting a *p* value < 0,05 and a fold change of 1 ± 0.05, where 1 was the value in untreated cells (Fig. 1; Table 1; Supplementary Table 1). When we infected keratinocytes with shp53, 3.754 DEGs were identified. Within these, 1.957 genes were up-regulated and 1.797 were down-regulated. In the case of DOXO 8.392 DEGs were identified (4.240 up-regulated and 4.152 genes down-regulated). In the case of Aurora B inhibition with ZM, we identified 1.280 DEGs (507 up and 773 down). Finally, when we inhibited Plk-1 by the use of BI, we identified 4.418 DEGs. Within these, 2.300 were up-regulated and 2.118 were down-regulated.

**Table 1:** Quantification of up and down-regulated DEGs for each treatment.

Treatment	Total DEGs	Up-regulated DEGs	Down-regulated DEGs
**shp53**	2.984	1.957	1.797
**DOXO**	8.392	4.240	4.152
**ZM**	1.280	507	773
**BI**	4.418	2.300	2.118

We performed Gene Ontology (GO) enrichment analyses focused on the biological processes of the sets of DEGs found in each treatment. Up and down-regulated DEGs were analysed independently. GO analyses revealed that p53 silencing and Plk-1 inhibition presented more similarities than DOXO treatment or Aurora B inhibition. DEGs found after p53 silencing and Plk-1 inhibition showed enrichment in DNA repair, mitosis progression, karyokinesis or cell division processes (Supplementary Fig. 1A, B). These processes were significantly enriched in down-regulated DEGs after DOXO and AurB inhibition (Supplementary Fig. 2A, B).

After analyses of up- and down-regulated genes separately, we found that 15 DEGs were commonly up-regulated in all the treatments (Fig. 1A; Supplementary Fig. 3A). GO enrichment analyses of these 15 DEGs, revealed significant enrichment for biological processes (BP) terms involving cell cycle regulation (*p* value < 0,01; Fig. 1C). 40 DEGs were down-regulated across all treatments (Fig. 1B; Supplementary Fig. 3B). Of these, we found that GO-BP terms involving lipid metabolism were significantly enriched (*p* value < 0,01; Fig. 1C). We initially analysed genes based on previously known functions, mentions in cancer and pattern of expression in the epidermis (Supplementary Fig. 4A). One of the genes that caught our attention was SUMO specific peptidase 2 (*SENP*2, commonly changed upon shP3+DOXO + BI), encoding the homonym protein, SENP2. SENP2 regulates SUMOylation processes and is broadly present in human cells. According to our hypothesis that the MDC is a barrier against genomic instability, we focused on this molecule because it is involved in post-translational protein regulation and because it has been suggested to play a role in some forms of cancer [13, 20]. In a second level of validation to select the best candidates, we found that SENP2 is significantly expressed in peribasal cells of human epidermis that initiate differentiation (Supplementary Fig. 4A, B; the same pattern is observed in ENSG00000163904-SENP2/tissue/skin, available from: https://www.proteinatlas.org/).

**Fig. 2:**
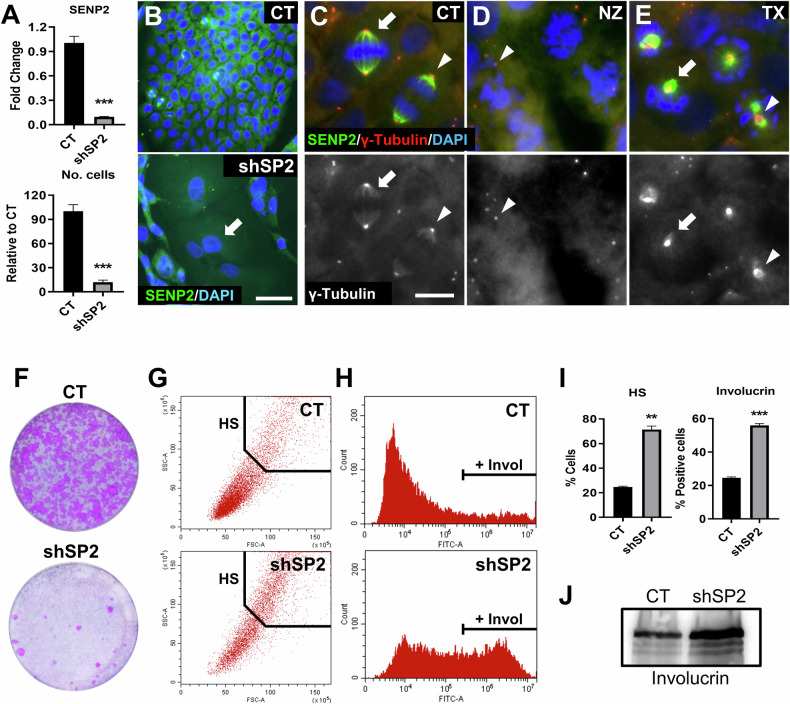
Silencing of *SENP2* causes a rapid loss of proliferative capacity and differentiation in human primary epidermal cells. **A**–**I** were performed 7 days post-infections; **J**, 5 days post-infections. **A** Top: expression of SENP2 quantitated by qRT-PCR 5 days post-infection, relative to CT (*n* = 3). Bottom: number of harvested cells 7 days post-infections, relative to CT (*n* = 3). **B** Immunofluorescence for SENP2 (green). Nuclei labelled with DAPI in blue. Arrow indicates a multinucleated cell. Scale bar 50 μm. Top: immunofluorescence for SENP2 (green) and γ-Tubulin (red) in human primary epidermal cells treated with DMSO only (**C**), Nocodazole (**D**) or Taxol (**E**) for 24 hours. Nuclei labelled with DAPI in blue. Bottom: γ-Tubulin. Arrows for SENP2 accumulation. Arrowheads for centrosomes. Scale bar: 10 μm. **F** Clonal expansion capacity of CT or shSP2 cells as monitored by clonogenicity assays. 7.500 total cells were plated per well and stained 8 days after plated (*n* = 3). **G** Representative flow cytometry dot plot displaying size and complexity light scatter parameters of CT or shSP2, as indicated. HS: the fraction of cells with high scatter values, typical of terminal differentiation. **H** Representative flow cytometry histogram for differentiation marker involucrin (invol) of CT or shSP2. **I** Percent of cells with high scatter values (HS), or positive cells for involucrin, as indicated. Quantitated by flow cytometry (*n* = 2). **J** Analyses by western blotting of the expression of Involucrin in primary keratinocytes.

To investigate the role of SENP2 in the control of the MDC, we silenced *SENP2* in human primary keratinocytes. Cells were infected with either of two specific lentiviral constructs expressing shRNA specific to different sequences of *SENP2* mRNA (shSP2a/b), or a non-targeting control (CT). Either shSP2a or shSP2b rapidly inhibited cell proliferation (Supplementary Fig. 5). shSP2a was so effective that we could not obtain enough cells for reliable molecular or cellular analyses. shSP2b (from now on shSP2) efficiently depleted *SENP2*, as measured by quantitative RT-PCR (Fig. 2A). The phenotypic effects observed upon both shRNAs, were very consistent 5 days after shRNA delivery, as keratinocytes rapidly displayed a highly differentiated morphology including large and frequently multinucleated cells (Supplementary Fig. 5A). Immunofluorescence analyses confirmed the diminished expression of SENP2 protein (Fig. 2B). In CT cells, SENP2 staining was cytoplasmic and strikingly accumulated and co-localised with γ−Tubulin along the mitotic spindle microtubuli (Fig. 2C; arrow). However, unlike γ−Tubulin, SENP2 did not localise to the centrosomes (arrowhead). To further explore whether SENP2 expression peaks at mitosis, we treated primary epidermal cells with either Nocodazole (Nz) that inhibits the polymerisation of Tubulin, or Taxol (Tx), that inhibit its depolymerisation. Interestingly, a mitosis block with Tx, but not a prometaphase block with Nz that hampers the formation of mitotic spindles (Fig. 2D), induced a strong accumulation of SENP2 around the disorganised centrosomes (Fig. 2E; arrow; Supplementary Fig. 6). Again, SENP2 did not co-localise with γ-Tubulin at the centrosomes (in red; arrowhead). Conversely, we could not detect mitotic figures or spindles after silencing SENP2. Within the same lines, the absence of SENP2 resulted in a marked loss of proliferation, as measured by both the number of cells and their clonogenic capacity after 7 days (Fig. 2A, F; Supplementary Fig. 5). The results altogether strongly suggest that SENP2 elicits its main function at mitosis and that it is here required to form the chromosomal spindle.

The loss of proliferative capacity was due to irreversible epidermoid terminal differentiation. The differentiated phenotype after silencing *SENP2* was quantitated by flow cytometry, what showed an increase in the proportion of cells with large size and high complexity (high light scatter, HS, typical of epidermoid differentiation; Fig. 2G, I) and in the proportion of cells expressing the differentiation marker involucrin (Fig. 2H–J). No sub-G1 cells indicative of apoptosis were detected in the cell cycle profiles in cells expressing shSP2 (see below). In addition, silencing of SENP2 did not cause cellular senescence, according to the β-Gal activity assay (Supplementary Fig. 7). Therefore, the results altogether suggest that silencing of SENP2 triggered epidermoid differentiation from a mitotic block. Although there was some proliferation in shSP2 cells these might express low levels of the shSP2, did not differentiate and eventually managed to divide.

**Fig. 3:**
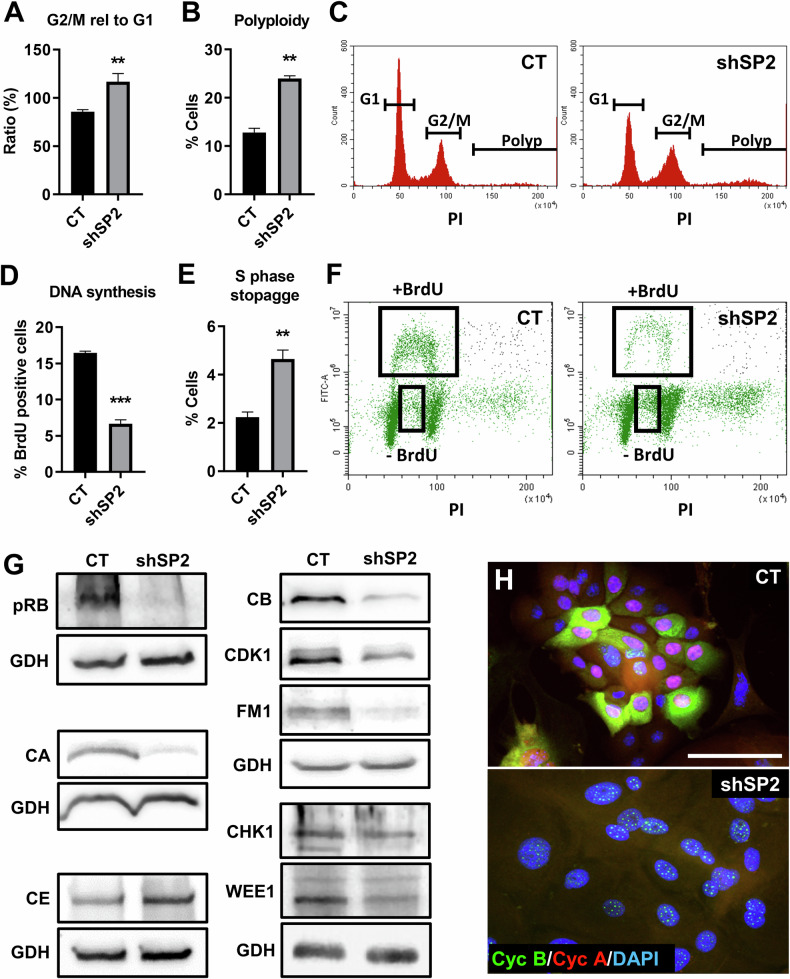
Depletion of SENP2 induces cellular polyploidy and other cell cycle defects in primary keratinocytes. Results were obtained 5 days post-infections. **A**, **B**. Bar histograms displaying the percent of cells in G2/M relative to G1 population or polyploid cells, as indicated (*n* = 3). **C** Representative flow cytometry histograms of cell cycle profiles labelled with Propidium Iodide (PI). **D**, **E** Bar histograms displaying the percent of positive cells for BrdU staining, and for BrdU negative cells in S phase, as indicated (*n* = 3). **F** Representative flow cytometry dot plot for BrdU incorporation along cell cycle phases. **G** Western blotting analyses of the expression of pRB, Cyclin A (CA), Cyclin E (CE), Cyclin B (CB), CDK1, FOXM1 (FM1), CHK1 and WEE1. GAPDH (GDH) as loading control. **H** Double immunofluorescence for Cyclin B (green) and Cyclin A (red) labelling. Nuclei labelled with DAPI in blue. Scale bar 50 μm.

Consistently with a mitosis defect and induction of epidermoid differentiation, the absence of SENP2 caused an increment in the ratio of G2/M and polyploid cells (5 days after delivery of shSP2; Fig. 3A–C). Polyploidy typically arises during epidermoid differentiation due to mitotic slippage [5, 7]. We also observed a significant decrease of DNA replication activity in shSP2 cells (Fig. 3D, F), as well as an increase of the proportion of BrdU negative cells in S phase (Fig. 3E, F), suggesting that the absence of SENP2 might affect DNA replication, or it might be a secondary consequence of mitotic defects.

We observed a generalised striking down-regulation of mitotic markers by western blotting 5 days after *SENP2* silencing: pRB, involved in S phase and mitosis progression; Cdk1, Cyclin A and Cyclin B, main drivers of the G2/M transition and mitosis [25, 26]; and FOXM1, global regulator of mitosis (Fig. 3G; Supplementary Fig. 8). In contrast, the DNA replication driver Cyclin E was increased in shSP2 cells (Fig. 3G; Supplementary Fig. 8). Cyclin E accumulates during endoreplication and terminal differentiation of keratinocytes [24, 27]. Immunofluorescence analyses confirmed that Cyclin A and B expression were notably down-regulated after silencing *SENP2* (Fig. 3G, H). Interestingly, the Cyclin B localisation pattern changed from the usual cytoplasmic staining to small nuclear foci in shSP2 cells. Nuclear Cyclin B precedes nuclear membrane breakdown and accumulates upon DNA damage before mitosis [28, 29].

**Fig. 4:**
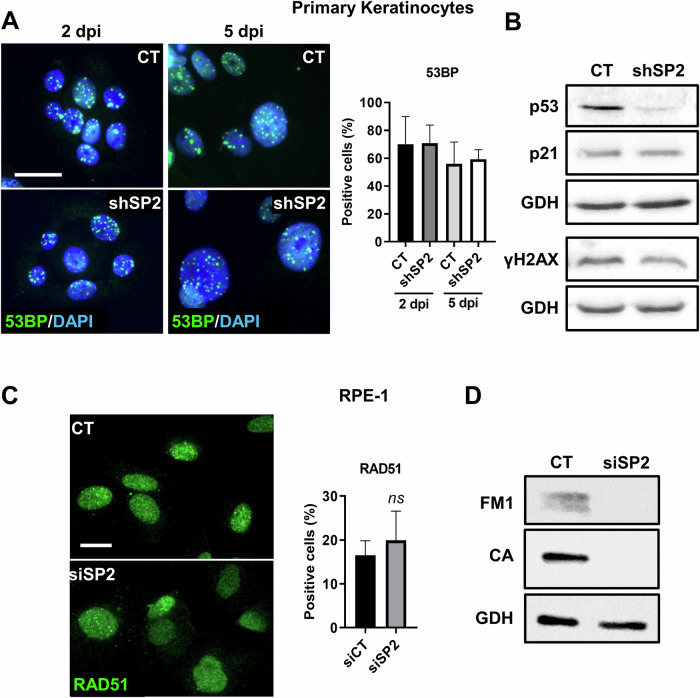
Cell cycle defects induced by *SENP2* silencing do not activate a DNA damage response. **A** Immunofluorescence for 53BP (green) in primary keratinocytes 2 or 5 days post-infection (dpi). Nuclear DNA labelled with DAPI in blue. Scale bar 50 μm. Bar histogram: percent of positive 53BP cells, 2 or 5 days post-infection with shSP2, as indicated (*n* = 71-381 cells). **B** Western blotting for the expression of p51, p21 or γH2AX in primary keratinocytes, 5 days post-infection. GAPDH (GDH) as loading control. **C** Immunofluorescence for RAD51 in RPE-1 cells 3 days post-transfections with CT or siSP2 as indicated. Scale bar 20 μm. Bar histogram: percent of positive RPE-1 cells with multiple foci of RAD51, 3 days post-transfection with siSP2 (*n* = 65–69 cells). **D** Western blotting for the expression of FOXM1 (FM1) and Cyclin A (CA) in RPE-1 cells, 3 days post-transfections with siSP2. GAPDH (GDH) as loading control.

A blockade in S and G2/M might be a response to DNA damage (DDR) via cell cycle checkpoints. For this reason, we analysed whether the DDR proteins were activated by silencing *SENP2*. However, neither checkpoint molecules Chk1 or Wee1 (Fig. 3G; Supplementary Fig. 8), nor DNA repair 53BP (Fig. 4A), nor DNA damage marker γH2AX (Fig. 4B; Supplementary Fig. 8) showed significant variations after silencing *SENP2* in human epidermal cells. In keratinocytes, DDR signalling proteins are lost during terminal differentiation [6]. Since the differentiation response was rapid, we analysed 53BP shortly after shSP2 delivery (2 days post-infection), with no changes observed (Fig. 4A). p53 and its transcriptional target p21CIP (p21) are induced in response to DNA damage and trigger cell cycle arrest [30]. However, neither p53 nor p21 was induced after silencing *SENP2* (Fig. 4B; Supplementary Fig. 8). On the contrary, p53 was down-regulated, possibly due to known the drop of the protein at the initiation of epidermoid differentiation [23]. In contrast, the stable expression of p21 might be due to a known p53-independent expression [23]. Similarly, we detected no differences in the expression of the DNA repair marker RAD51 in RPE-1 cells upon SENP2 RNAi, even though mitotic markers were consistently down-regulated (Fig. 4C, D; Supplementary Fig. 8). These results further suggest that the effects caused by silencing SENP2 are independent of the DDR and might directly control the epidermoid mitosis-differentiation link.

**Fig. 5:**
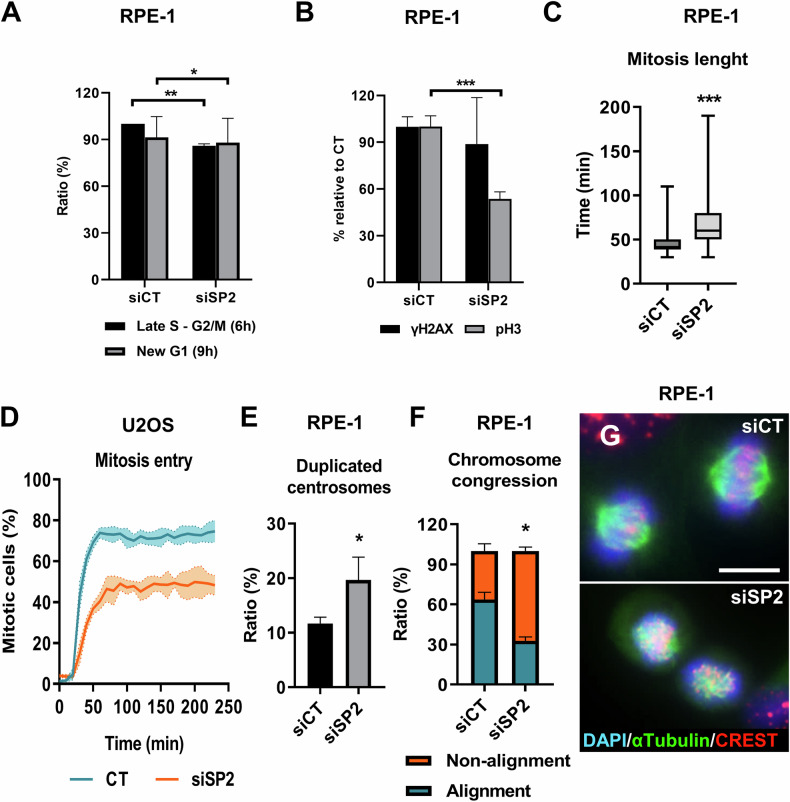
*SENP2* silencing causes mitotic delays and chromosome alignment defects. Results were obtained at 3 days post-transfection. **A** Bar histogram of the percent of EdU-labelled RPE-1 cells by flow cytometry after delivery of siCT or siSP2, as indicated, in Late S/G2 phase and back into G1 phase after completing a whole cell cycle. Cells analysed 6 hours and 9 hours after EdU removal, respectively (*n* = 2). **B** Bar histogram of the percent of pH3 or γH2AX positive RPE-1 cells, relative to siCT quantitated by immunofluorescence (*n* = 3 randomly selected fields). **C** Bar histogram for the time (min) needed for a cell to fulfil mitosis, measured by live cell imaging in RPE-1 cells (*n* = 107–119 cells). **D** Quantitation of the percentage of U2OS Cdk1AS H2B-mCherry cells entering mitosis after Cdk1 release, as measured by live cell imaging (*n* = 3). **E** Bar histogram of the percent of cells with duplicated centrosomes quantitated by immunofluorescence (*n* = 3 randomly selected fields). **F** Bar histogram for percent of RPE-1 cells with a correct or incorrect chromosome congression, as indicated. Quantitation by immunofluorescence after blocking mitosis exit for 3 hours with MG132 (*n* = 112–200 cells). **G** Representative immunofluorescence images for α-Tubulin (green) and centromeres (CREST, red) of RPE-1 cells blocked in metaphase after 3 hours of MG132 treatment. Nuclei labelled with DAPI in blue. Scale bar 10 μm.

We aimed to define the cell cycle defect caused by silencing *SENP2*. Since primary epithelial cells cannot be synchronised and were highly sensitive to SENP2 depletion, we made use of hTERT-immortalised retinal pigment epithelial cells RPE-1. We studied the cell cycle dynamics after silencing *SENP2* using siRNAs in RPE-1 cells. An EDU pulse-chase assay revealed a delay in S phase (siSP2 versus siCT; late S-G2/M and new G1; Fig. 5A; Supplementary Fig. 9). This result suggested a slight perturbation of S phase in siSP2 cells. However, and as for primary keratinocytes, we did not detect an induction of the DNA damage marker γH2AX (Fig. 5B; Supplementary Fig. 10). In contrast, we observed a drop in the phosphorylation of histone H3 (pH3; Fig. 5B; Supplementary Fig. 10), marker of chromosome condensation. On the other hand, a live cell imaging analysis revealed a significant increase in the length of mitosis (Fig. 5C; Supplementary Video 1). Therefore, we designed an assay to monitor mitosis entry. We used a modified osteosarcoma U2OS cell line bearing a Cdk1AS (Analogue sensitive; [31, 32]) to synchronise cells in the G2/M transition. These cells can be synchronised in G2/M due to inactivation of mitotic kinase Cdk1 and then released and held in metaphase by use of a protease inhibitor (MG132). Cells were monitored by live cell imaging and they displayed a significant delay in mitosis entry in the absence of SENP2 (Fig. 5D). Concomitantly, we observed an accumulation of cells with duplicated and separated centrosomes (Fig. 5E). The results indicate that cells were arrested in G2, prior to mitosis entry.

We questioned whether the delay in mitosis entry in the absence of SENP2 caused chromosomal defects. To analyse this, we blocked RPE-1 cells in metaphase by addition of MG132 for 3 hours and quantitated the ratio of cells that achieved correct chromosome alignment in the metaphase plate. siSP2 cells displayed a high ratio of aberrant chromosome alignment (Fig. 5F, G), as compared to non-targeting siRNA, siCT. Taken together, the results point out a critical role of SENP2 in progression of mitosis.

**Fig. 6:**
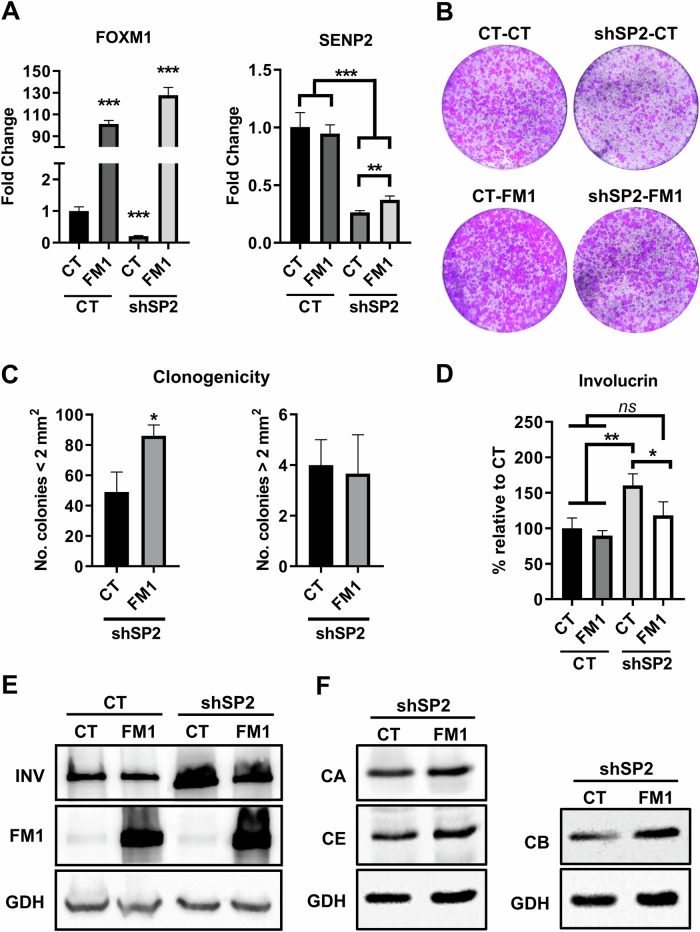
Mitosis exit driven by FOXM1 relieves differentiation caused by SENP2 depletion in primary keratinocytes. **A** Expression of FOXM1 (left) and SENP2 (right) as quantitated by qRT-PCR 5 days post-infection, in CT or shSP2, as indicated, relative to CT (*n* = 3). **B** Representative clonogenic capacity monitored by clonogenicity assays of cells plated 7 days post-infections. 7.500 total cells were plated per well and stained 8 days after plated (*n* = 3). **C** Number of colonies smaller or larger than 2 mm^2^ in the clonogenicity assays in **B** (*n* = 3). **D** Bar histogram displays the percent of positive cells for involucrin, quantitated by flow cytometry, 6 days post-infection (*n* = 3). **E** Analyses by western blotting of the expression of involucrin (INV) and FOXM1 (FM1), 6 days post-infections, cells as indicated. GAPDH (GDH) as loading control. **F** Western blotting for the expression of Cyclin A (CA), Cyclin E (CE) or Cyclin B (CB) 6 days post-infections. GAPDH (GDH) as loading control.

To challenge the hypothesis that loss of SENP2 triggered MDC activation in primary keratinocytes through mitosis, we wondered whether boosting the mitotic machinery would relief the differentiation response. For this purpose, we generated double mutant cells shSP2-FOXM1 (Fig. 6A) in primary epidermal keratinocytes. FOXM1 is a master regulator of mitosis and induces essential mitotic proteins, such as Cyclin B, Aurora B and Plk-1 [33, 34]. Furthermore, FOXM1 overexpression in primary keratinocytes can overcome the MDC even in conditions of oncogenic replication stress [35, 36]. shSP2/FOXM1 double mutant cells displayed increased clonogenic capacity compared to shSP2 cells (Fig. 6B, C). In addition, the percent of cells with high involucrin expression significantly diminished (Fig. 6D, E). Concomitantly, the expression of mitotic cyclins A and B was rescued (Fig. 6F; Supplementary Fig. 8). These results show that the MDC, induced in absence of SENP2, was alleviated upon FOXM1 overexpression.

**Fig. 7:**
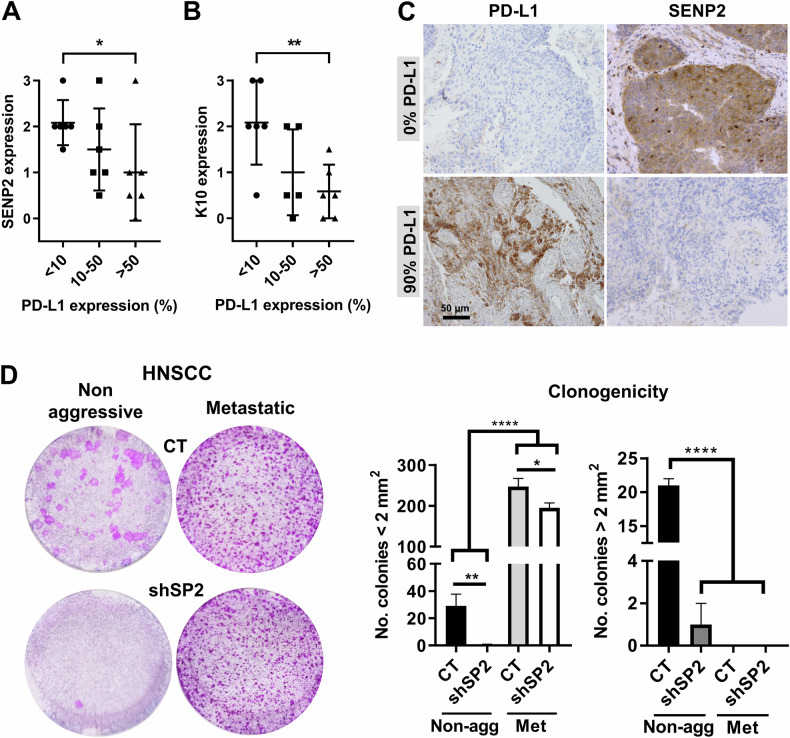
Loss of SENP2 correlates with expression of immunocheckpoint protein PD-L1 in lung SCC. **A**, **B** Quantitation by immunohistochemistry of SENP2 or keratin K10, respectively, in LSCC according to the expression levels of PD-L1, as indicated (*n* = 6, each subgroup). **C** Microphotographs from paraffin sections of representative cases of LSCCs with high (top) or low (bottom) expression of SENP2 and low (left) or high (right) expression of PD-L1 or SENP2. Scale bar 50 μm. **D** Representative clonogenicity assays of a non-aggressive, MDC-responding or a metastatic MDC-non-responding HNSCC after silencing *SENP2* by shSP2. 7.500 total cells were plated per well and stained 10 days later (*n* = 3). Bar histograms display the number of colonies smaller or larger than 2 mm^2^ (*n* = 3).

The epithelial MDC arises as a protective barrier against oncogenic mutations and genomic instability. Some aggressive lung SCC express the immunocheckpoint marker PD-L1 [37, 38]. Therefore, we investigated a possible relationship between SENP2 and PD-L1 expression, utilising a pilot collection of lung SCCs. In the non-lesional lung epithelium, SENP2 expression was prominent in pseudostratified cells (Supplementary Fig. 4B). Interestingly, we found low expression of SENP2 in samples with high PD-L1 expression and vice versa (Fig. 7A, C). While keratins K1/10 are scarcely expressed in the hyperproliferative conditions in vitro, they form the cytoskeleton of differentiating squamous epidermoid cells in well-differentiated SCC [39]. Therefore, expression of K10 is generally marker of less aggressive SCC. Tumours with low PD-L1 expression also displayed high expression of the differentiation marker Keratin K10 (Fig. 7B).

To investigate a functional link between the loss of SENP2 and the MDC in aggressive carcinomas, we made use of two head and neck carcinomas explanted in vitro. ORT5 was derived from a non-aggressive oral squamous carcinoma and displayed an intact MDC (unpublished). ORT3 was derived from a metastatic oral squamous carcinoma and did not conserve the MDC. We silenced *SENP2* in either tumour by shSP2 and determined the loss of proliferative capacity. Interestingly, MDC-responding SCC was highly affected by shSP2, whereas MDC-nonresponding metastatic SCC was unaffected (Fig. 7D).

## Discussion

The search for novel molecules involved in the MDC regulation is an essential step to understand the mechanisms coordinating growth with differentiation in self-renewing epithelia face to carcinogens. From our genetic and functional study, SENP2 emerges as a potential MDC regulator.

### SENP2 in the control of mitosis and differentiation

Within the cell fates induced by unrepaired DNA damage, terminal differentiation is the most poorly studied [10]. Contrary to apoptosis or senescence, terminal differentiation preserves both the integrity and the function of the tissue. This is critical to self-renewal differentiating tissues that are continuously exposed to genotoxic hazard. We previously demonstrated that a DNA damage-induced mitosis-differentiation response is present in cells from a variety of human epithelia, from the epidermis to the lung or the mammary gland [8, 11, 40]. Our current results strongly suggest that SENP2 has a dual role in mitosis and in epidermoid differentiation. Accumulation and co-localisation with γTubulin at the mitotic spindle but not at the centrosomes, the lack of mitotic figures upon silencing the protein, the defects found then in chromosomal segregation and the time for mitosis in immortalised cells, they all argue for an essential role of SENP2 to form and to maintain the mitotic spindle. Interestingly, SENP1 has also been found to localise to the chromosomal spindle and kinetochores in HeLa cells [41] and SENP2 has been suggested to be involved in kinetochore dynamics during mitosis in trophoblasts [42, 43]. The observation that mitotic FOXM1 alleviates epidermoid differentiation induced upon SENP2 inhibition, further argues for a simultaneous role of the latter in mitosis and in differentiation. FOXM1 is a global regulator of a set of proteins of mitosis [34]. In addition, FOXM1 degradation is mediated by SUMOylation, with the involvement of SENP2 [44, 45]. It is therefore tempting to speculate that the silencing of *SENP2* might cause premature degradation of G2/M regulators, obstructing mitotic entry and aggravating mitotic defects.

A prolonged G2/M arrest in keratinocytes leads to mitotic slippage and polyploidisation and epidermal cells became multinucleated upon silencing *SENP2*. This is consistent with the finding of SENP2 expression in peribasal cells of human epidermis that initiate differentiation. These cells are mostly mitotic [27, 46]. The nuclear expression pattern of Cyclin B, observed after SENP2 depletion, is usually linked to a prolonged G2/M blockade due to DNA damage or mitotic exit defects [28, 29]. Interestingly, either SENP2 overexpression or inhibition, was described to generate disorders in DNA repair upon double strain breaks [47]. Within these lines, in addition to the striking mitotic block, inhibition of SENP2 also caused S phase progression delay in RPE-1 cells. Similarly, Lin et al., [48] observed that SENP2-mediated deSUMOylation affected S phase progression. SUMOylation has been proposed to be important during DNA replication [49–51]. Therefore, we cannot rule out that the absence of SENP2 in epidermal cells causes errors during S phase. However, we detected no increase of DNA damage sensor proteins and our experiments on synchronised cells unequivocally show a direct role for SENP2 in mitosis. DNA replication delays might also be consequence of chromosomal defects during mitosis. Nevertheless, although SUMOylation is involved in the regulation of a diversity of proteins and it is impossible at this stage to elucidate which ones are directly regulated by SENP2, the results here shown altogether strongly argue for a direct role of SENP2 in the control of the epidermoid mitosis/differentiation switch.

The localisation of SENP2 strongly accumulated around mitotic microtubules that we found in the human primary cells resembles that of SUMO proteins. SUMO-1 and SUMO-2/3 patterns in microtubuli change through mitosis, setting a sequence during spindle formation, chromosome alignment and chromosome segregation [42, 52, 53]. SUMOylation was therefore proposed to play a significant role during mitosis [41, 42, 54]. Our results show largely multinucleated epidermal cells and mitotic aberrations in RPE-1 epithelial cells after SENP2 depletion. Cubeñas-Potts et al., 2013 reported mitotic defects by SENP2 overexpression in HeLa cell line, but not by its knockdown. The absence of phenotype after silencing *SENP2* in HeLa cells but not in RPE-1 cells might be explained by the additional loss of a normal regulatory mechanism, such as the inactive p53 karyotype for instance [55–58]. Both the inhibition and the deregulation of the cell cycle regulatory function of SENP2 might cause a cell cycle defect. This also might explain why both alterations of SENP2 have been found associated with some cancers.

### SENP2 and cancer

SENP2 has been described as a prognostic marker in bladder, breast or hepatocellular cancer, and in osteosarcoma [59–63]. In addition, the 3q26-29 amplicon, where the *SENP2* gene is located was found amplified in 70-80% of the head and neck and lung SCCs [64, 65]. Independently, Karatas et al., 2021 [66] and Meng and Li, 2021 [67] found SENP2 overexpressed in head and neck SCC, suggesting it as a marker of poor prognosis, alone or in combination with multiple SUMOylation-regulated genes. Finally, Wang et al., 2013 [68] identified SENP2 amplification in 34% of analysed LSCC, associating a better response to chemotherapy treatment.

PD-L1 is a biomarker of prognosis and choice in immunotherapy of some types of cancer [69]. Overexpression of PD-L1 was suggested to identify aggressive lesions. Less differentiated SCCs tend to develop a more aggressive behaviour, with a poorer response to chemotherapy [70–72]. However, they display a good response ratio to immunotherapy treatment [37, 73]. Some aggressive LSCCs have been shown to respond well to immunotherapy [38]. The MDC constitutes a barrier against genomic instability and cancer aggressiveness. The involvement of SENP2 in this checkpoint might explain the correlation that we found in our LSCCs pilot survey, between its loss, the loss of keratin K10 and the expression of PD-L1. Consistently, in our study oral metastatic SCC cells were resistant to the inhibition of SENP2. New biomarkers of choice of immunotherapy of aggressive carcinomas are required to complement PD-L1. Our results encourage larger studies for a correlation between SENP2 expression and immunotherapy response of epithelial cancer.

**Fig. 8:**
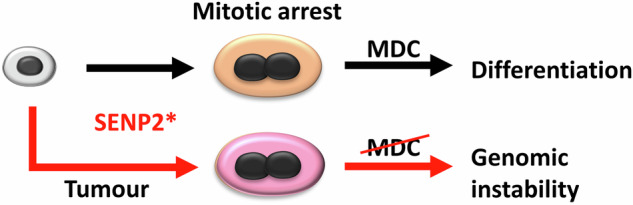
Model for the deregulation of SENP2 leading to genomic instability in cancer cells bypassing the MDC. Alterations (*) in the expression of SENP2 would trigger the MDC in normal epithelial cells. However, in cancer cells with impaired MDC, it would lead to genomic instability due to mitosis defects.

As conclusion, alteration of SENP2 function in the MDC, might contribute to genomic instability (Fig. 8) and explain the positive or negative association of SENP2 with cancer. As we knew little about the SENP2 functions, our results provide mechanisms by which its alteration can contribute to cancer.

## Materials and methods

### Ethics

Ethical permissions for this study were requested, approved, and obtained from the Ethical Committee for Clinical Research of Cantabria Council, Spain (2014.166, 2017.259 and PI20/00880). In all cases, human tissue material discarded after surgery was obtained with written consent presented by clinicians to the patients, and it was treated anonymously.

### Cell culture

Cells were cultured at 37 °C and 5% CO_2_. Primary keratinocytes were isolated from neonatal human foreskin and grown in co-culture with a mouse fibroblast feeder layer (inactivated by mitomycin C), in Rheinwald FAD medium as previously described [74, 75]: 3:1 (v/v) DMEM/Ham’s F12 (Dulbecco’s Modified Eagle Medium; ref. BE12‐604 F, Lonza; ref. BE12‐615 F, Lonza), 5% Fetal Bovine Serum (FBS, ref. F7524, Sigma-Aldrich), 0,5 μg/mL hydrocortisone (ref. H0888, Sigma‐Aldrich), 5 ng/mL Epidermal Growth Factor (EGF; ref. E9644, Sigma‐Aldrich), 9 ng/mL Cholera toxin (ref. C8052, Sigma‐Aldrich), 180 μM adenine (ref. A2786; Sigma‐Aldrich), 5 μg/mL insulin (ref. I5500, Sigma‐Aldrich), 2 mM L‐glutamine (ref. BE17‐605E; Lonza), 0,75 mM Sodium Pyruvate (ref. BE13‐115E, Lonza), and 100 U/mL Penicillin-Streptomycin (Pen-Strep. ref. E17‐602E, Lonza).

Low passages (1–3) keratinocytes from three different individuals were utilised. Mouse fibroblast J2 cell line used as feeder layer was cultured in DMEM, 10% Donor calf serum (ref. 16030074, Gibco), 2 mM L‐glutamine and 100 U/mL Pen-Strep. hTERT RPE-1 are human retinal pigment epithelial cells immortalised by ectopic expression of telomerase hTERT. U2OS Cdk1AS H2B-mCherry are human bone osteosarcoma epithelial cells. These cell lines were cultured in DMEM, 10% FBS, 2 mM L‐glutamine and 100 U/mL Pen-Strep.

Primary human keratinocytes were treated for the lengths of time indicated throughout with the following chemical compounds: 0,5 μM Doxorrubicin (ref. D1515; Sigma-Aldrich), 10 nM BI2536 (Ref. 1129; Axon Medchem), 2 μM ZM44739 (ref. 2458; Tocris), 20 μM Nocodazole (M1404; Sigma-Aldrich) or 100 nM Taxol (Paclitaxel T7402; Sigma-Aldrich) diluted in DMSO. Control samples contained an equal volume of drug vehicle.

### Lentiviral infections and siRNA transfections

For gene delivery in primary keratinocytes the following lentiviral constructs driven by constitutive promoters were used: a control vector pLK01 (ref. SHC001, Sigma-Adrich), a construct expressing shRNA against p53, shp53 (ref. 19119; Addgene) and two different constructs expressing shRNA against SENP2 with different target sequences: shSP2a (ref. TRCN0000004578) and shSP2b (ref. TRCN0000004579), both from Sigma-Aldrich, showed results correspond to shSP2b construction due to it higher effectiveness; a control vector pLVX-AcGFP1-N1 (ref. 632154, Clontech) and a construction to overexpress FOXM1 (pLVX-FOXM1; [76]). Lentiviral production was performed by transient transfection of 293 T cells. Concomitantly, keratinocytes were cultured in FAD medium until confluence and lentiviral infections were performed as previously described [77]. Lentiviral infections were made in FAD medium. Double mutants were co-infected at the same time.

For RPE-1 and U2OS cell lines, RNAi transfection was performed using Lipofectamine RNAiMAX (ref. 13778-075, Invitrogen), following the commercial protocol. A SMARTpool scrambled control siRNA was used was negative control (ref. 1027280, Qiagen), and a SMARTpool ON-TARGET plus against SENP2 (ref. L-006033-00-0005, Dharmacon).

### Live cell imaging

To measure mitosis length in hTERT RPE-1 cells, live cell imaging assays were performed using an Olympus IX71 microscope equipped with an environmental chamber (Digital Pixel, Microscopy Systems & Solutions), maintaining conditions at 37 °C and 5% CO_2_. Cells were imaged using differential interference contrast (DIC) with a 20x lens equipped with an OrcaFlash cMos camera (Hamatsu). Images were acquired every 10 min for 48 hours using Micromanager v1.4 software. Time lapse between cells rounded-up and attached-back again was measured. Analysis was performed with ImageJ.

To perform mitosis entry assays, U2OS Cdk1AS H2B-mCherry cells were plated at a density of 6.000–9.000 cells per well into 96 well plates suitable for microscopy (ref. CLS3614, Corning). 24 hours later, fresh medium with 2 μM 1NM-PP1 was added and incubated for 20 hours. To release the blockage and mitosis entry, samples were washed 5 times with medium. Last wash contained 25 μM MG132. Cells were incubated in an Operetta CLS (PerkinElmer) at 37 °C and 5% CO_2._ Mitosis entry was recorded for 4 hours imaging every 10 min. Mitotic release dynamics were quantitatively assessed using a bespoke Python script available from: https://github.com/HocheggerLab/Mitotic-Release.

### Clonogenicity assays

For clonogenicity assays, primary keratinocytes were grown at low density (7.500 cells per well) in FAD medium and plated in 6 well dishes in triplicates as previously described [78]. After 8–10 days, wells were washed with 1x PBS and fixed with 3.7% formaldehyde (ref. F8775, Sigma‐Aldrich) for 10 min. Wells were then washed with PBS and stained with Rhodanile‐Blue solution (1% Rhodamine B, ref. R6626, Sigma‐Aldrich; 1% Nile Blue A, ref. N5632, Sigma‐Aldrich; in distilled water) for 12 min. Then, wells were washed with distilled water three times and dried at room temperature. The total number of colonies with a diameter smaller or larger than 2 mm^2^ was counted.

### Antibodies

The following primary antibodies were used: anti-SENP2 (ref. ab58418, Abcam; Immunofluorescence (IF) and immunohistochemistry (IHC), anti-GAPDH (0411; ref. sc-47724, Santa Cruz Biotechnology; Western Blot, WB), anti-BrdU (B44, ref. 347580, BD Biosciences; Flow cytometry, FC), anti-p53 (FL-393, ref. sc-6243, Santa Cruz Biotechnology; WB), anti-p21 (WAF1/Cip1; ref. P1484, Sigma-Aldrich; WB), anti-γH2AX Ser139 (JBW301; ref. 05–636, Merck Millipore; IF and WB), anti-53BP1 (ref. A300-272A, Bethyl; IF), anti-involucrin (SY3; [79]; FC and WB), anti-Cyclin A (H-432; ref. sc-751, Santa Cruz Biotechnology; IF and WB), anti-Cyclin B (GNS1, ref. sc-245, Santa Cruz Biotechnology; IF and WB), anti-Cyclin E1 (HE12, ref. sc-247, Santa Cruz Biotechnology; WB), anti-Cdk1 (A17.1.1, ref. MAB8878, Milipore; WB), anti-CREST (ref. 115–234, Antibodies Inc; IF), anti-FOXM1 (ref. sc-502, Santa Cruz Biotechnology; WB), anti-CHK1 (FL-476; ref. sc-7898, Santa Cruz Biotechnology; WB), anti-WEE1 (B-11; ref. sc-5285, Santa Cruz Biotechnology; WB), anti-Histone phosphor-H3 ser10 (pH3; ref. sc-8656-R, Santa Cruz Biotechnology; IF), anti-pRB ser780 (ref. 9307, Cell signalling; WB), anti-α-Tubulin (ref. T6199, Sigma-Aldrich; IF), anti-γ-tubulin (GTU-88; ref. T6557, Sigma-Aldrich; IF), anti-RAD51 (ref. sc-8349, Santa Cruz Biotechnology; IF) and anti-PD-L1 (22C3; ref. GE006, Agilent; IHC).

The following secondary antibodies from Jackson ImmunoResearch were used: Alexa Fluor® 488-conjugated goat anti-rabbit or anti-mouse IgG antibodies (ref. 111-547-003 and 115-547-003, respectively; FC and IF) and Alexa Fluor® 594-conjugated goat anti-rabbit or anti-mouse IgG antibodies (ref. 111–517 and ref. 115–517, respectively; IF). Other secondary antibodies used were: DyLight 800-conjugated goat anti-rabbit or anti-mouse IgG antibodies (ref. SA5-35571 and ref. SA5-35521, respectively; ThermoFisher; WB), DyLight 680-conjugated goat anti-mouse IgG antibody (ref. SA5-35518; ThermoFisher; WB), DyLight 650-conjugated goat anti-Human (ref. #SA5-10137, Invitrogene; IF) and HRP-conjugated goat anti-rabbit or anti-mouse IgG antibodies (ref. 170–6515 and ref. 170–6516, respectively; Bio-Rad; WB).

### Flow cytometry

Cells were harvested, fixed in ethanol 70% v/v and stained as previously described [80]. For involucrin staining cells were fixed for 10 min in formaldehyde 3,7% v/v. All antibody staining was controlled using a similar concentration of negative isotype immunoglobulins (mouse or rabbit serum). Cytometry assays were performed on a CytoFLEX (Beckman Coulter). 10.000 events were gated and acquired in mode list. DNA content analysis with PI (25 μg/ml, 12 hours) were performed as previously described [24].

For BrdU staining, keratinocytes were incubated with 10 μM BrdU for 2 hours before harvesting and fixation. For pulse-chase assays, RPE-1 cells were incubated with 10 μM EdU for 1 hour and washed 3 times with PBS before re-incubating in media. Then, cells were harvested every 3 hours for 24 hours. Staining was made following the Click-iT™ EdU Alexa Fluor™ 488 Flow Cytometry Assay Kit (ref. C10425, Thermo-Fisher).

### Tissue histology and immunodetection

For immunohistology, samples were retrieved from the department of Anatomical Pathology (Assignment code CS22-092) of Hospital Universitario Marqués de Valdecilla (Santander, Spain). Sections were cut at 4 μm and deparaffinized using a standard protocol using a heat-induced antigen retrieval in EnVision™ FLEX High pH buffer (ref. GV800, Dako Omnis) for 20 min at 98 °C. The slides were then incubated with primary antibodies for 12 hours at 4 °C. Immunohistochemistry (IHC) sections were incubated with the appropriate secondary antibodies and revealed using an EnVision FLEX kit (ref. K8000 y GV800, Dako Omnis). Contrast was added by haematoxylin staining (GC808, Dako Omnis) for 5 min. Finally, coverslips and slides were mounted with Prolong Gold Antifade Reagent (ref. P10144, Life Technologies).

For immunofluorescence, cells were grown on glass coverslips, fixed, and stained as previously described [24]. For determination of protein expression, cells were washed with PBS, lysed with sonication, and subjected to SDS-PAGE electrophoresis followed by western blotting as previously described [24]. Same number of lysed cells (Involucrin) or same amount of lysed protein was loaded on the electrophoresis per sample.

### qRT-PCR and RNAseq

For qRT-CPR assays, total RNA was isolated and reverse-transcribed using the NucleoSpin^®^ RNA kit (Macherey-Nagel; ref. 740955.50) and the iScript™ cDNA synthesis kit (Bio-Rad; ref. 4106228) according to the manufacturer’s instructions. The cDNA was amplified by real-time PCR using iQ™ SYBR Green supermix (ref. #1708880; Bio-Rad). Primers utilized in this study for human genes were: FOXM1 (5’-CTGTGCAGATGGTGAGGCAG-3’ and 5’-AGTCATGCGCTTCCTCTCAG-3’), SENP1 (5’-CGAGCACGAGAAAGATTGCG-3’ and 5’-ACTGAATGTTCCCGCTCCTG-3’), SENP2 (5’-CTTGTGAACTGACAGGTTCTGG-3’ and 5’-ACCAAAGGAAGGCAGGACTC-3’), SENP3 (5’-CCGACCCTCTTTTGATGCCT-3’ and 5’-CAGCTGACTCCATCTTGGGG-3’), SENP5 (5’-CAGGTGAGAGTGGCACGATT-3’ and 5’-CAGCAGCCGTAACAAAAGCC-3’), SENP6 (5’-GATTAAGAAGGAGGCGGCGT-3’ and 5’-GTAATCTCCCCTGCGCTACC-3’), SENP7 (5’-GCCAACAAGGTGCAATCAGA-3’ and 5’-TAAGGCTTTGGCGAAGAGGT-3’), β-Actin (5’-GCGGGAAATCGTGCGTGACATT-3’ and 5’-GATGGAGTTGAAGGTAGTTTCGTG-3’) and β-2-Microglobulin (5’-GAGACATGTAAGCAGCATCA-3’ and 5’-AGCAACCTGCTCAGATACAT-3’). qRT-PCR results are presented normalized to β-actin or β-2-Microglobulin signal of each sample and relative to controls.

For the RNAseq analysis, total RNA was isolated from keratinocytes collected 30 hours after infection with shp53-427 or the corresponding empty vector, and 16 hours after treatment with Doxorubicin, ZM447439 or BI2536. In the case of shp53, the NucleoSpin® RNA isolation kit (Macherey-Nagel) was used and RNA samples were sent to the Centro Nacional de Análisis Genómico (CNAG) for their quantification and quality control. DNA libraries were prepared and sequenced according to CNAG procedures. RNA from the other treatments was isolated using DNA/RNA Mini Kit (Quiagen). These samples were sent to the Sequencing core of the University of Michigan for library preparation and sequencing. Reads were mapped using STAR [81] and gene expression levels were measured and normalized by HTSeq [82] and DESeq2 [83]. Differentially expressed genes (DEGs) were identified based on their *p* value < 0,05. The Database for Annotation, Visualization, and Integrated Discovery (DAVID; http://david.abcc.ncifcrf.gov/) was used to classify the DEGs according to their biological processes by using the Gene Ontology (GO) Consortium Reference [84, 85]. Common relevant gene changes found are listed in Supplementary Fig. 3 (Supplementary Table 1 for individual treatments).

### Senescence by β-Galactosidase activity assay

The expression of senescence marker β-Galactosidase was analysed adapting the protocol described by Itahana et al., 2012 [86]. Cells were grown on round glass coverslips, fixed with formaldehyde 3.7% for 5 min and incubated for 16 hours, at 37 °C in an incubator without CO_2_ supply, with the X-gal staining solution (1 mg/ml X-gal (SIGMA, B4252), 40 mM citric acid/sodium phosphate buffer (pH 5.2) (SIGMA, C1909; 55136), 5 mM potassium ferricyanide (SIGMA, 702587), 5 mM potassium ferrocyanide (SIGMA, P3289), 150 mM NaCl (Acros), and 2 mM MgCl2 (SIGMA, 208337)). Staining solution was then removed and cells were washed twice with PBS and mounted with the Gold Antifade Reagent Prolong mounting medium (Thermo Fisher Scientific, P10144). Cells were visualised and photographed by microscopy (ECLIPSE TS100F and LEDCMOS 5MPCOLOR Nikon) under contrast phase or bright field (for blue colour).

### Image analysis and quantitations

Analysis of images was performed using ImageJ software. For scoring 53BP and Rad51 positive cells by immunofluorescence, multifoci positive cells were manually counted with respect to the total number of nuclei in the field, based on DAPI staining. The results were expressed as percent of positive cells. In the case of pH3 or γH2AX, an intensity threshold was defined by using ImageJ to determine the number of nuclei that were positive for pH3 or γH2AX. The results were expressed relative to siCT.

Western blot band intensities were quantitated by using ImageJ software. The intensity of each band was represented relative to loading control band.

### Statistical analyses

Data are presented as mean ± SD from two or more independent culture dishes conditions, as indicated in each figure, and at least two independent experiments. An unpaired two-tailed Student’s *t* test was used when two data sets were compared or One-way ANOVA when more than two data sets were analysed. For multiple comparison, depending on the data dispersion, tests used were Tukey test or Newman-Keuls test, as indicated in each figure legend. *p* values considered statistically significant are indicated as *p < 0.05, **p < 0.01, ***p < 0.001 and ****p < 0.0001. In every case, sample size was chosen accordingly. Damaged samples were excluded from analyses.

### Supplementary information

Supplementary Video 1

Supplementary Figures

Supplementary Table 1

Original western blots

## Data Availability

All data supporting this study are presented in this published article and in its Supplementary information files.
