# Supplementary material for: Sumo-regulatory SENP2 controls the homeostatic squamous mitosis-differentiation checkpoint
Source: Cell Death Dis. 2024 Aug 16;15(8):596. doi: 10.1038/s41419-024-06969-z (PMC11329632; doi:10.1038/s41419-024-06969-z)
Supplement: Supplementary file 2 — Supplementary Figures [file 41419_2024_6969_MOESM2_ESM.pdf]

Sumo-regulatory SENP2 controls the homeostatic squamous mitosis-differentiation checkpoint.

Galán-Vidal et al

SUPPLEMENTARY VIDEO

Supplementary Video 1. Time-lapse microscopic recording showing the time needed for a cell to complete mitosis, as monitored by live cell imaging. RPE-1 cells transfected with control RNAi (siCT) or SENP2 siRNA (siSP2), as indicated. Time lapse of 180 min, frames recorded every 10 min. Scale bar, 10 µm.

SUPPLEMENTARY FIGURES

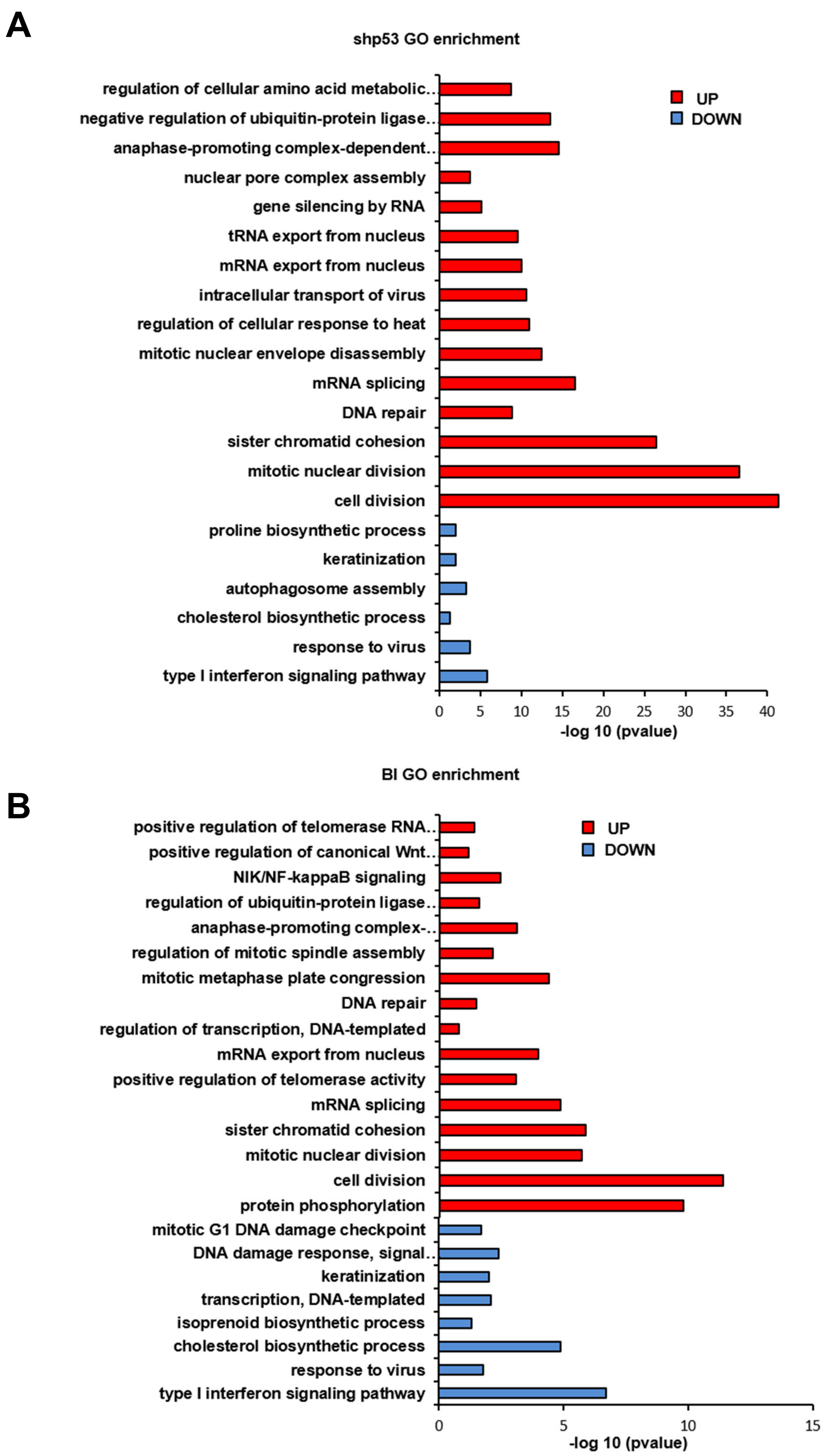

Supplementary Figure 1. Bar histogram representing p values (-log 10) of the most significant GO terms enriched by up- (red bars) or down-regulated (blue bars) DEGs identified after infection with shp53 (**A**) for 30 h or treatment with BI2536 (**B**) for 16 h. It complements Figure 1.

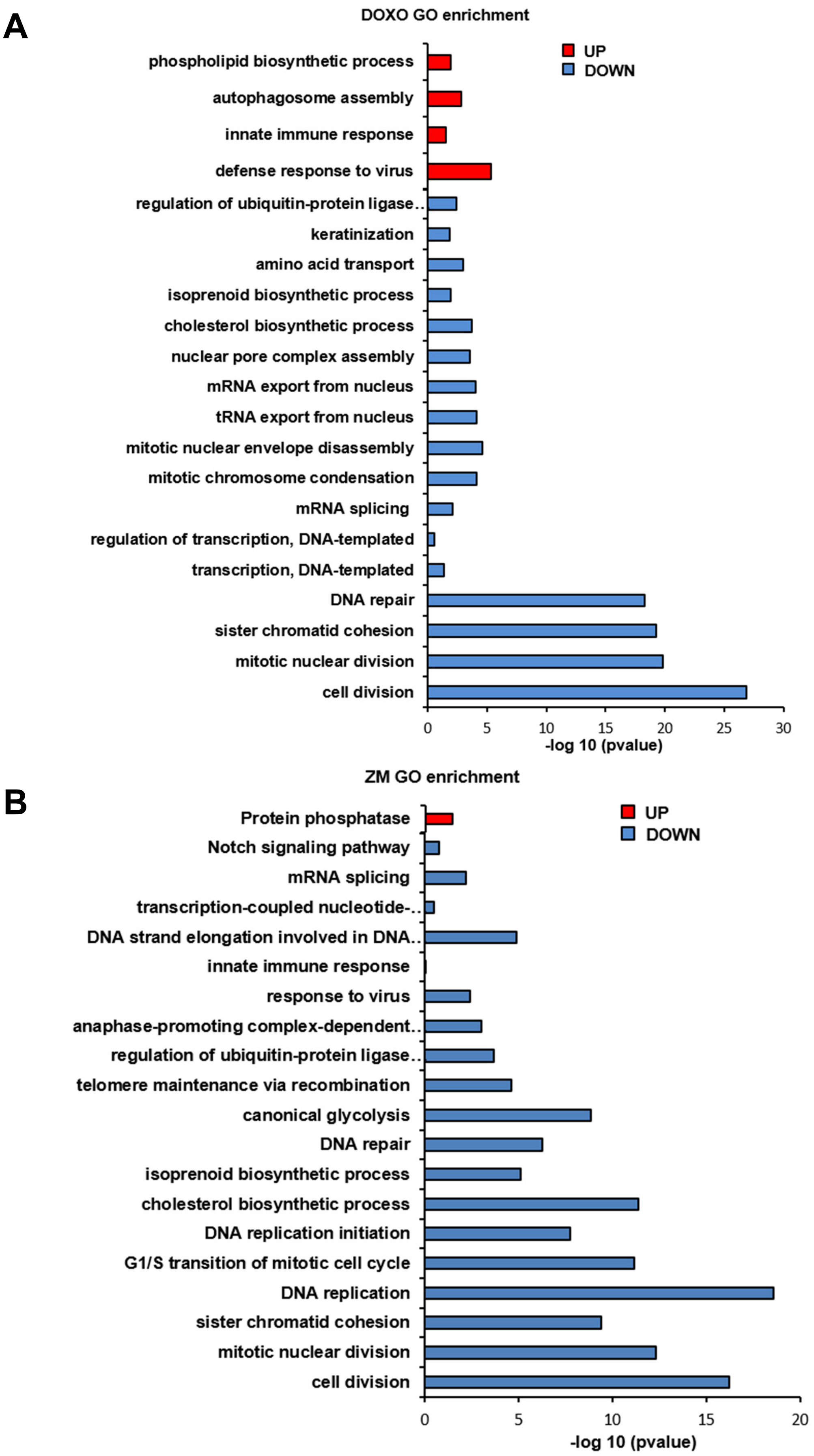

Supplementary Figure 2. Bar histogram representing p values (-log 10) of the most significant GO terms enriched by up- (red bars) or down-regulated (blue bars) DEGs identified after treatment with Doxorubicin (**A**) or ZM44739 (**B**) for 16 h. It complements Figure 1.

A

Up-regulated Genes	
**LRP5**	Low-Density Lipoprotein Receptor-Related Protein 5
**EIF2AK4**	Eukaryotic Translation Initiation Factor 2 Alpha Kinase 4
**PKD2**	Polycystin 2, Transient Receptor Potential Cation Channel
**RPS6KA1**	Ribosomal Protein S6 Kinase A1
**MGEA5**	Meningioma Expressed Antigen 5
**NECAP1**	NECAP Endocytosis Associated 1
**MARK4**	Microtubule Affinity Regulating Kinase 4
**UBR1**	Ubiquitin Protein Ligase E3 Component N-Recognin 1
**CCNG1**	Cyclin G1
**GDF11**	Growth Differentiation Factor 11
**G0S2**	G0/G1 Switch 2
**PLAU**	Plasminogen Activator, Urokinase
**TOP1**	DNA Topoisomerase I
**ARRDC4**	Arrestin Domain Containing 4
**OSTM1**	Osteoclastogenesis Associated Transmembrane Protein 1

B

Down-regulated Genes	
**GINS2**	GINS Complex Subunit 2
**SLC3A2**	Solute Carrier Family 3 Member 2
**TMEM45A**	Transmembrane Protein 45A
**CTDSP1**	CTD Small Phosphatase 1
**SQLE**	Squalene Epoxidase
**FAM213B**	Peroxiredoxin like 2B
**ACSS2**	Acyl-CoA Synthetase Short Chain Family Member 2
**PDPN**	Podoplanin
**ALDOC**	Aldolase, Fructose-Bisphosphate C
**ANGPTL4**	Angiopoietin Like 4
**CYP26B1**	Cytochrome P450 Family 26 Subfamily B Member 1
**MVK**	Mevalonate Kinase
**SLC38A5**	Solute Carrier Family 38 Member 5
**SLC2A1**	Solute Carrier Family 2 Member 1
**MUC16**	Mucin 16, Cell Surface Associated
**SPRR1A**	Small Proline Rich Protein 1A
**LSS**	Lanosterol Synthase
**TYMP**	Thymidine Phosphorylase
**KRTDAP**	Keratinocyte Differentiation Associated Protein
**LAD1**	Ladinin 1
**TMEM184A**	Transmembrane Protein 184A
**IFI44L**	Interferon Induced Protein 44 Like
**ARTN**	Artemin
**ADM**	Adrenomedullin
**DHCR24**	24-Dehydrocholesterol Reductase
**RP11-1002K11.1**	lncRNA
**KIAA1161**	Myogenesis regulating glycosidase
**SPINK5**	Serine Peptidase Inhibitor Kazal Type 5
**PVRL1**	Poliovirus receptor-related protein 1
**ACAT2**	Acetyl-CoA Acetyltransferase 2
**CRABP2**	Cellular Retinoic Acid Binding Protein 2
**MIR210HG**	MIR210 Host Gene
**FDPS**	Farnesyl Diphosphate Synthase
**ADAMTS1**	ADAM Metallopeptidase With Thrombospondin Type 1 Motif 1
**KLF6**	KLF Transcription Factor 6
**NOTCH3**	Notch Receptor 3
**C6orf132**	Chromosome 6 Open Reading Frame 132
**EIF1AD**	Eukaryotic Translation Initiation Factor 1A Domain Containing
**WDR34**	WD repeat domain 34
**UPK1B**	Uroplakin 1B

Supplementary Figure 3. Commonly up-regulated (**A**) or down-regulated (**B**) genes after the treatments with doxorubicin, Aurora B inhibition or Plk-1 inhibition for 16 h, or 30 h after infection with shRNA against p53. It complements Figure 1.

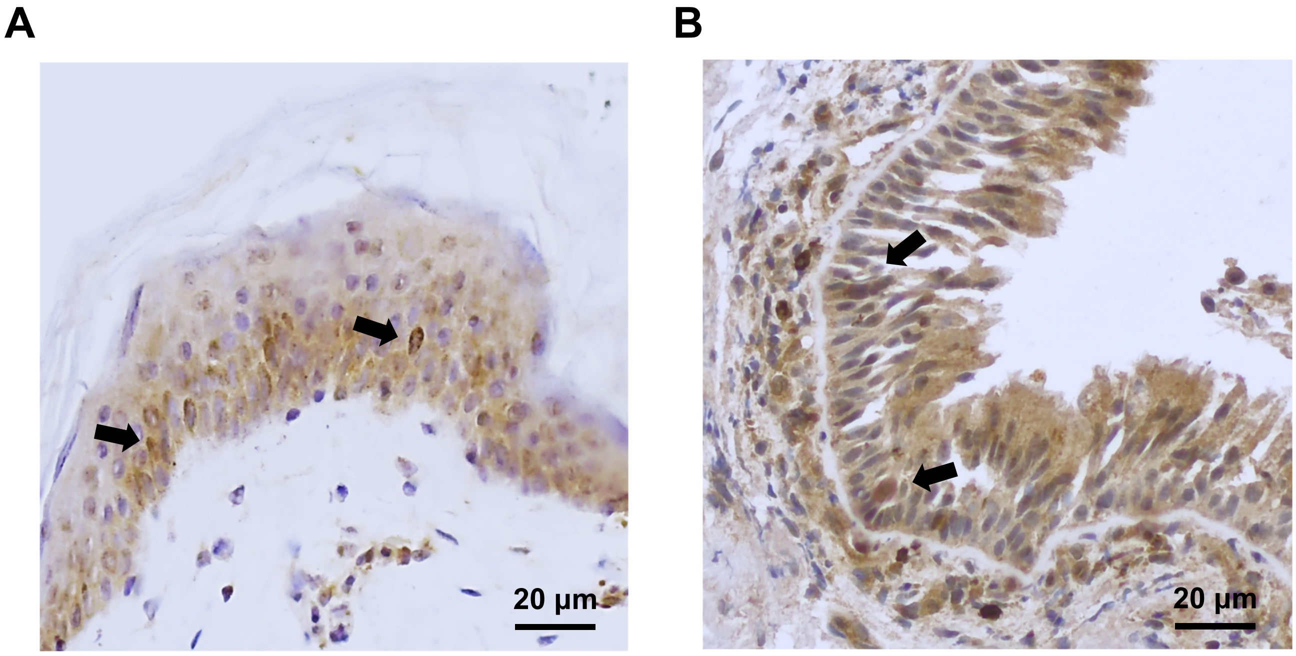

Supplementary Figure 4. Representative fields of paraffin sections of human non-lesional skin epidermis (**A**) or lung epithelium (**B**) stained for SENP2. Black arrows point at stratifying cells with high SENP2 expression. Scale bar 20 µm.

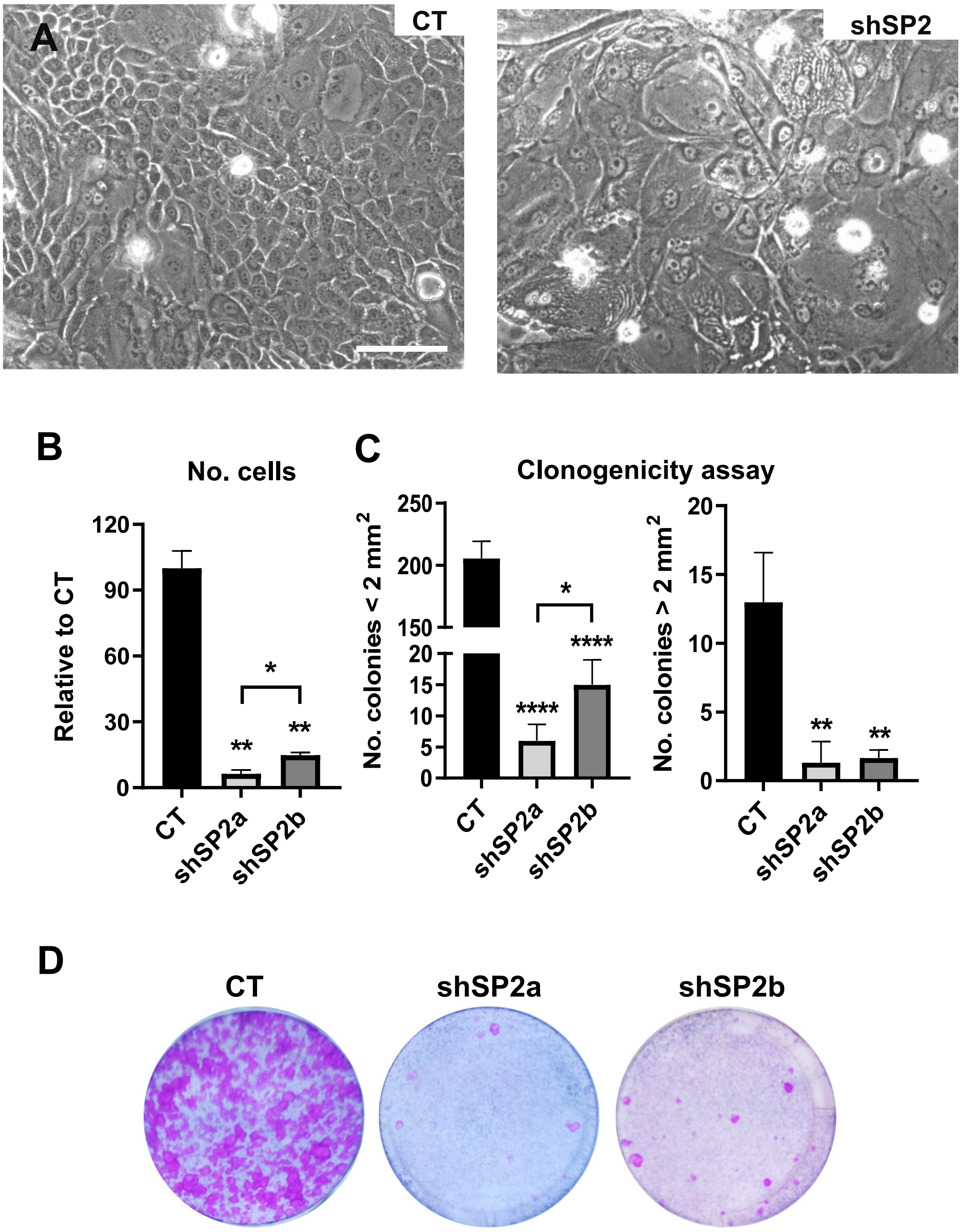

Supplementary Figure 5. A Representative phase-contrast microphotographs of primary keratinocytes 5 days after infection with CT or shSP2, as indicated. Scale bar 50 µm. **B** Number of harvested cells 7 days after infections, relative to CT (*n*=2). **C** Number of colonies in the clonogenicity assays for clonal expansion capacity of CT, shSP2a or shSP2b -infected cells, as indicated (*n*=3: smaller (left) or larger (right) than 2 mm^2. 7,500 total cells were plated per well and wells stained 8 days later). **D** Representative wells of the clonogenicity assays in C.

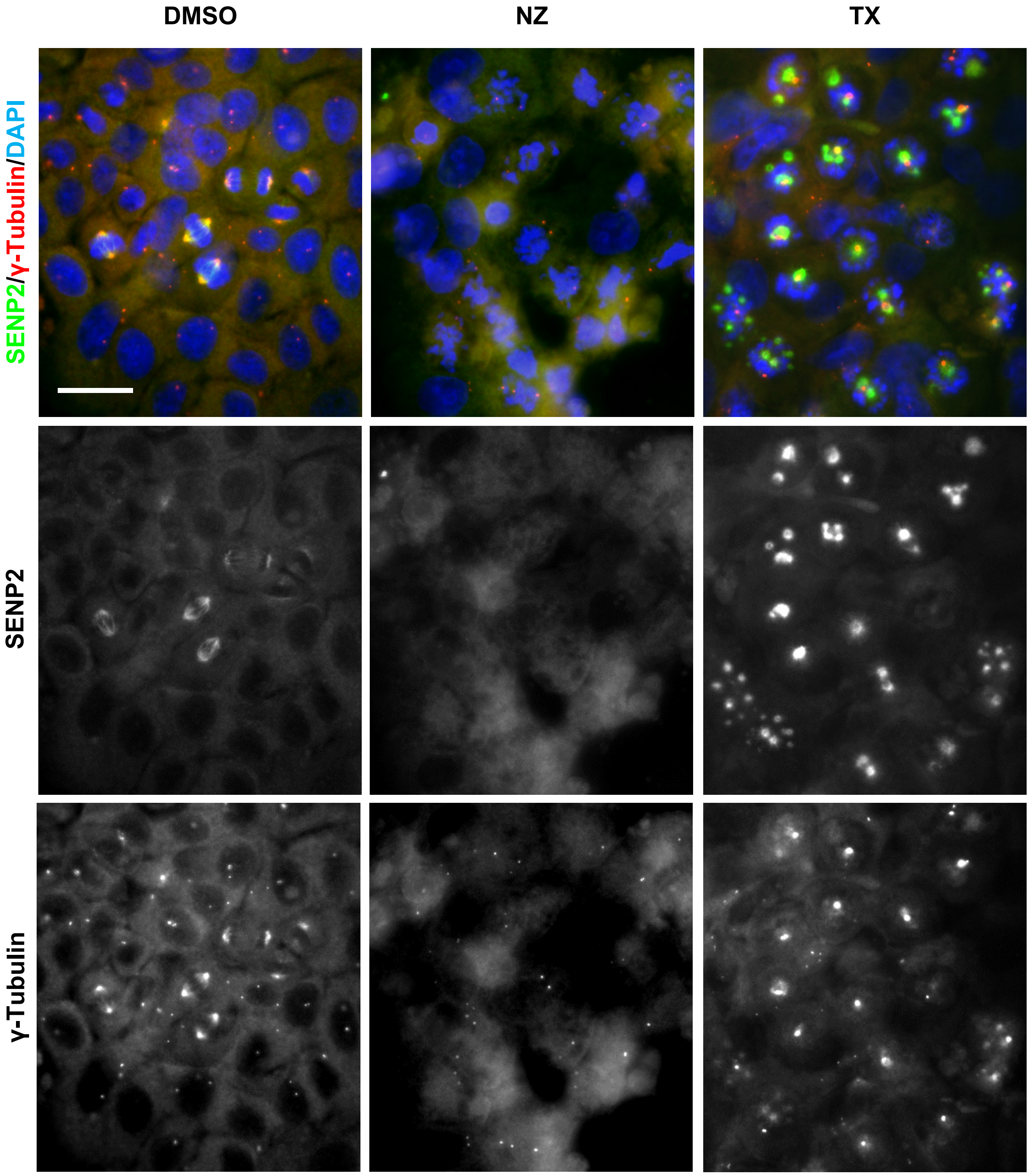

Supplementary Figure 6. Representative immunofluorescence images for SENP-2 (Green) and α-Tubulin (Red) of primary keratinocytes treated with DMSO, Nocodazole or Taxol, as indicated. Nuclei labelled with DAPI in blue. Scale bar 50 µm.

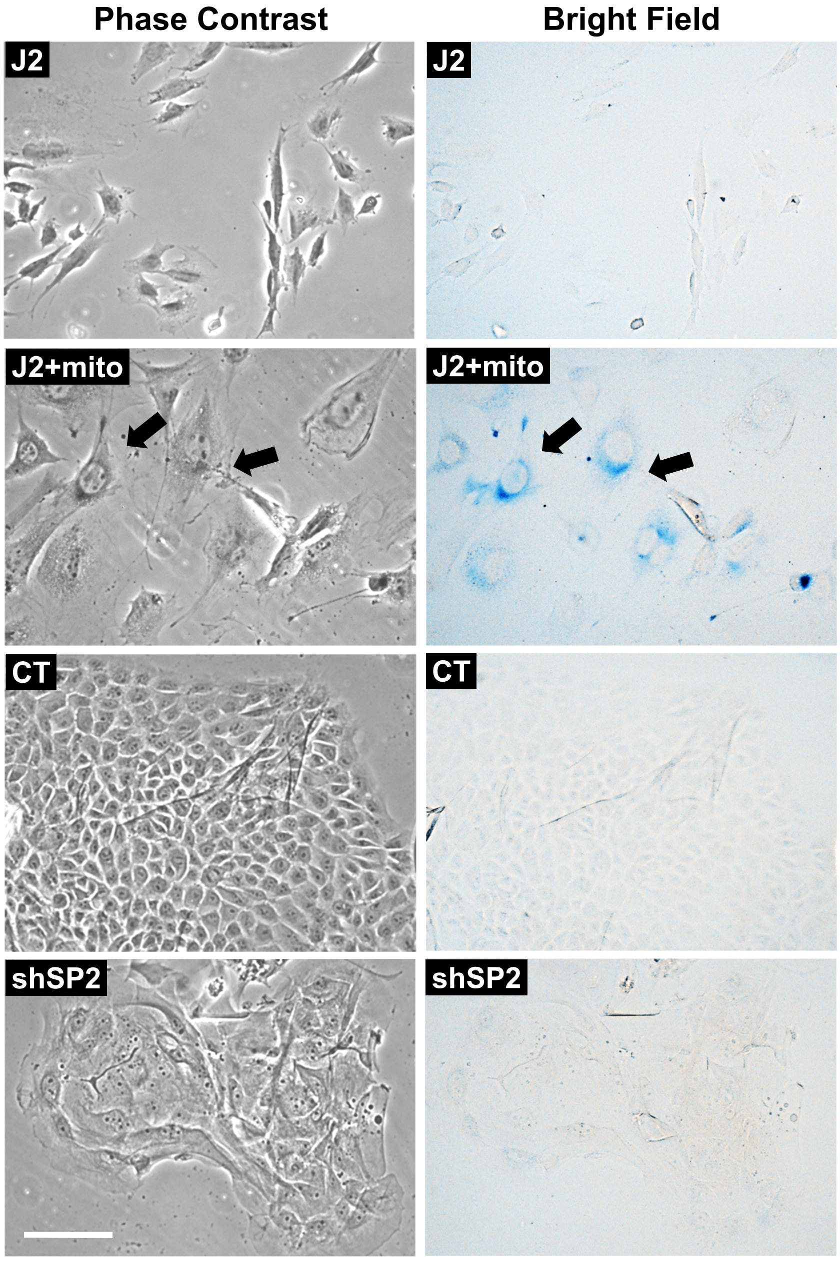

Supplementary Figure 7. ß-galactosidase staining of primary keratinocytes as a marker of senescence. 7 days after infection with CT or shSP2, as indicated. Proliferating J2 cells are negative control. J2 cells 3 d after a 2 h treatment with Mitomycin C (mito), are positive control (blue; arrows). Images are of phase contrast (left) or bright field (right) microscopy. Scale bar, 50 µm. Three independent assays on two different keratinocyte strains were performed.

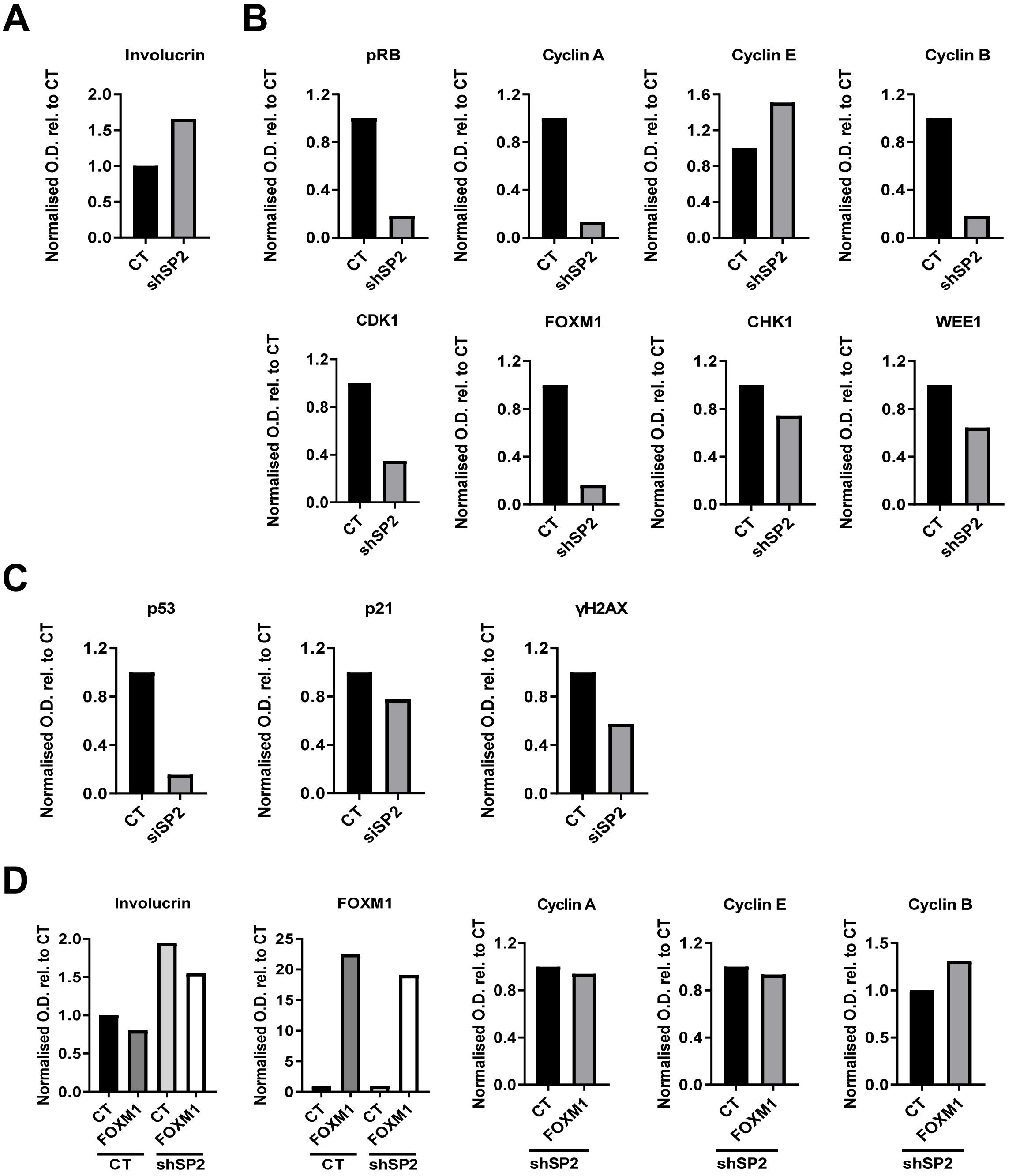

Supplementary Figure 8. Quantitation of the immunoblots corresponding to Figures 2I (**A**), 3G (**B**), 4B, D (**C**) or 6E, F (**D**).

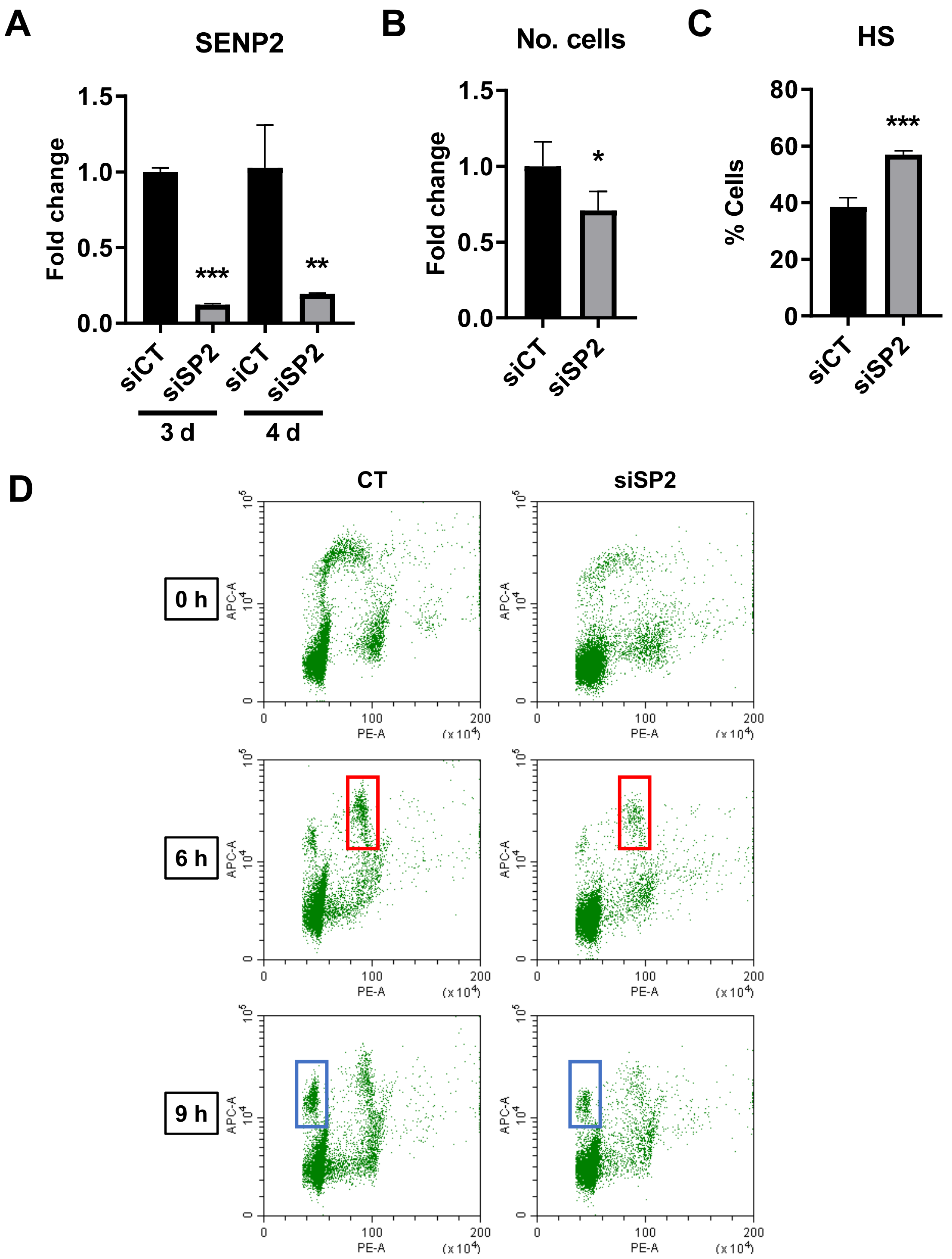

Supplementary Figure 9. Cell cycle analyses of RPE-1 cells after silencing of SENP2. **A** Expression of SENP2 as quantitated by qRT-PCR, 3 or 4 days post-transfection of RPE-1 cells with specific siSP2 RNA, relative to siCT, as indicated (*n*=3). **B** Number of harvested RPE-1 cells 3 days post-transfection, relative to siCT (*n*=4). **C** Percent of cells with high light scatter values (HS), quantitated by flow cytometry, 3 days post-infections (*n*=4). **D** Representative flow cytometry dot plots of pulse-chase assays. Red and blue rectangles highlight areas quantitated in Figure 5 A and B, respectively.

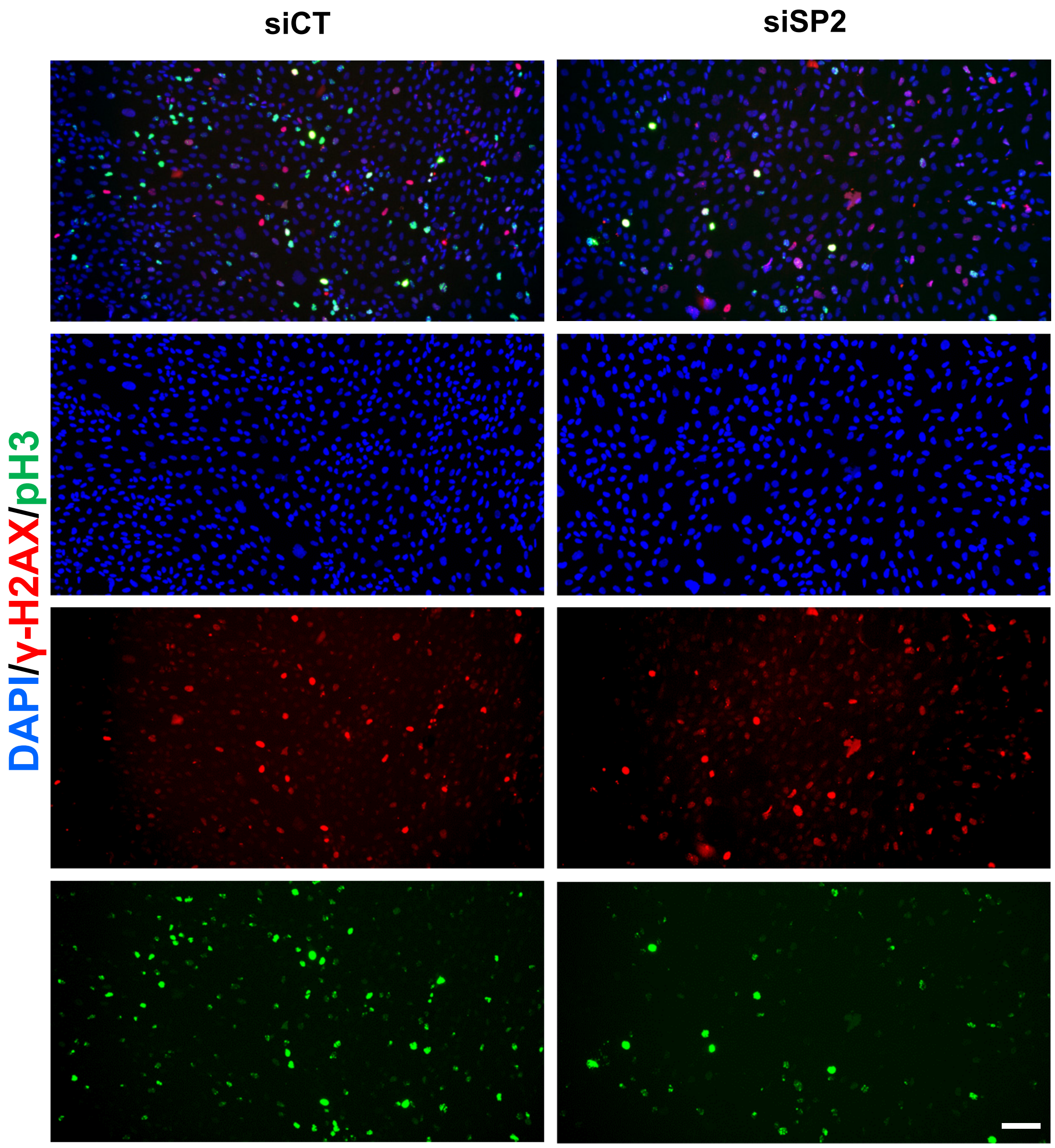

Supplementary Figure 10. Representative immunofluorescence images for pH3 (Green) and γ-H2AX (red) of RPE-1 cells infected with siCT (left) or siSP2 (right). Nuclei labelled with DAPI in blue. Scale bar 10 µm. It complements Figure 5D. Scale bar, 100 µm.
