# Supplementary material for: Sumo-regulatory SENP2 controls the homeostatic squamous mitosis-differentiation checkpoint
Source: Cell Death Dis. 2024 Aug 16;15(8):596. doi: 10.1038/s41419-024-06969-z (PMC11329632; doi:10.1038/s41419-024-06969-z)
Supplement: Supplementary file 3 — Supplementary Table 1 [file 41419_2024_6969_MOESM3_ESM.pdf]

Galán-Vidal et al. Supplementary Table 1

Selected genes with p value <0.05 and fold change 1+/-0.05, where 1 is the value of untreated control cells, upon the four treatments.

DMSO vs DOXO					DMSO vs ZM				
**Gene_ID**	**log2FoldChange**	**Fold Change**	**pvalue**	**padj**	**Gene_ID**	**log2FoldChange**	**Fold Change**	**pvalue**	**padj**
**INPP5D**	3.10	8.59	0.00E+00	0.00E+00	**CDKN1A**	0.66	1.58	2.80E-50	2.87E-46
**CUL9**	2.78	6.88	0.00E+00	0.00E+00	**SLC2A1**	-0.47	0.72	2.46E-34	1.26E-30
**PLK1**	-3.55	0.09	0.00E+00	0.00E+00	**INPP5D**	0.59	1.50	5.68E-20	9.69E-17
**CDKN1A**	2.65	6.28	0.00E+00	0.00E+00	**MDM2**	0.62	1.53	3.31E-24	8.47E-21
**TRIM22**	2.78	6.84	0.00E+00	0.00E+00	**H19**	-0.74	0.60	6.75E-29	2.30E-25
**KIF20A**	-3.48	0.09	0.00E+00	0.00E+00	**F3**	-0.42	0.75	3.72E-23	7.62E-20
**KRT15**	2.78	6.88	2.18E-302	5.51E-299	**SLC6A8**	-0.42	0.75	1.23E-17	1.80E-14
**CDIP1**	2.70	6.51	6.90E-291	1.52E-287	**TMEM97**	-0.58	0.67	2.57E-16	3.29E-13
**RRM2B**	3.26	9.60	3.78E-285	7.43E-282	**GJB2**	-0.36	0.78	5.16E-16	5.87E-13
**CDC20**	-2.47	0.18	2.05E-274	3.63E-271	**RRM2**	-0.34	0.79	3.65E-14	3.74E-11
**MDM2**	2.59	6.00	2.45E-261	3.94E-258	**TXNIP**	0.36	1.29	5.44E-14	5.06E-11
**GDF15**	6.31	79.19	3.46E-248	5.10E-245	**ADM**	-0.53	0.69	1.66E-13	1.42E-10
**CCNB1**	-2.08	0.24	7.04E-214	9.56E-211	**ANGPTL4**	-0.44	0.74	2.06E-13	1.62E-10
**SLC12A4**	2.13	4.37	9.13E-205	1.15E-201	**MVD**	-0.44	0.74	1.08E-12	7.90E-10
**CENPF**	-2.81	0.14	9.93E-203	1.17E-199	**FADS2**	-0.54	0.69	4.64E-12	3.17E-09
**CALML3**	3.13	8.73	2.31E-192	2.55E-189	**LSS**	-0.32	0.80	6.47E-12	4.14E-09
**TPX2**	-1.94	0.26	1.38E-185	1.44E-182	**DDIT4**	-0.28	0.82	3.35E-11	2.02E-08
**GM2A**	1.48	2.80	7.03E-181	6.90E-178	**FAM111B**	-0.44	0.74	4.57E-11	2.60E-08
**BUB1B**	-2.58	0.17	1.22E-180	1.12E-177	**TIMP3**	-0.33	0.79	1.88E-10	1.01E-07
**BTG2**	2.00	4.01	1.27E-180	1.12E-177	**TGFBI**	-0.39	0.77	1.99E-10	1.02E-07
**PGF**	2.60	6.08	1.14E-178	9.59E-176	**RRM2B**	0.48	1.39	2.79E-10	1.36E-07
**TGM1**	-1.79	0.29	5.22E-178	4.19E-175	**CUL9**	0.39	1.31	4.29E-10	2.00E-07
**FGFBP1**	-1.63	0.32	1.06E-172	8.12E-170	**MX1**	-0.44	0.74	5.60E-10	2.46E-07
**UBE2C**	-2.59	0.17	1.78E-171	1.31E-168	**GJB6**	-0.39	0.77	5.77E-10	2.46E-07
**NINJ1**	2.98	7.89	9.61E-169	6.80E-166	**SMOC1**	0.39	1.31	1.72E-09	7.04E-07
**TNFRSF10B**	1.57	2.97	1.92E-158	1.30E-155	**IVL**	-0.23	0.85	1.85E-09	7.29E-07
**HJURP**	-2.65	0.16	8.61E-155	5.64E-152	**SPRR1A**	-0.37	0.77	2.03E-09	7.72E-07
**ASPM**	-2.63	0.16	3.41E-154	2.15E-151	**FAM110C**	0.37	1.29	2.75E-09	1.01E-06
**SUGCT**	3.38	10.41	8.62E-154	5.25E-151	**LDHA**	-0.27	0.83	4.64E-09	1.64E-06
**KPNA2**	-1.47	0.36	9.20E-153	5.42E-150	**KRT78**	-0.43	0.74	8.43E-09	2.88E-06
**BUB1**	-2.25	0.21	1.11E-152	6.34E-150	**TFPI2**	0.27	1.21	1.63E-08	5.37E-06
**PRC1**	-1.83	0.28	1.05E-147	5.81E-145	**ZNF395**	-0.36	0.78	1.74E-08	5.58E-06
**APOBEC3C**	1.68	3.21	4.27E-146	2.29E-143	**PRNP**	0.22	1.17	2.21E-08	6.86E-06
**TM7SF3**	1.43	2.69	6.27E-145	3.26E-142	**IL33**	0.48	1.40	3.00E-08	9.03E-06
**DLGAP5**	-2.23	0.21	8.13E-145	4.10E-142	**XPC**	0.40	1.32	4.19E-08	1.23E-05
**ESPN**	3.17	8.99	4.71E-142	2.31E-139	**KIAA0040**	0.29	1.22	4.42E-08	1.26E-05
**TACC3**	-1.93	0.26	3.35E-140	1.60E-137	**CCND2**	0.28	1.22	4.60E-08	1.27E-05
**CEP55**	-2.08	0.24	1.96E-139	9.13E-137	**PTAFR**	0.36	1.29	4.75E-08	1.28E-05
**GJB2**	-1.73	0.30	2.35E-139	1.06E-136	**CDIP1**	0.35	1.27	5.49E-08	1.44E-05
**TRIB3**	-1.78	0.29	7.12E-138	3.15E-135	**NDRG1**	-0.42	0.75	5.67E-08	1.45E-05
**PRR11**	-1.93	0.26	3.13E-137	1.35E-134	**EDN2**	-0.46	0.73	5.99E-08	1.50E-05
**CCNA2**	-2.15	0.23	5.62E-137	2.37E-134	**APOBEC3C**	0.29	1.23	7.01E-08	1.71E-05
**TIGAR**	2.09	4.27	1.22E-136	5.02E-134	**TENM2**	-0.28	0.82	9.08E-08	2.16E-05
**SESN2**	2.19	4.57	4.21E-136	1.69E-133	**ZMAT3**	0.37	1.29	1.03E-07	2.40E-05
**LMNB1**	-2.37	0.19	2.30E-132	9.04E-130	**TNFRSF10B**	0.28	1.22	1.36E-07	3.09E-05
**SAT1**	1.61	3.04	2.74E-131	1.05E-128	**BTG2**	0.33	1.25	1.79E-07	3.99E-05
**CCNB2**	-2.06	0.24	4.53E-131	1.70E-128	**HK2**	-0.28	0.82	2.80E-07	6.09E-05
**H2AFX**	-1.80	0.29	2.58E-129	9.50E-127	**ASNS**	0.25	1.19	3.05E-07	6.52E-05
**NCAPD2**	-1.45	0.37	2.98E-129	1.07E-126	**NINJ1**	0.43	1.35	3.35E-07	6.99E-05
**HMGB2**	-1.87	0.27	6.76E-129	2.39E-126	**TM7SF3**	0.26	1.20	4.64E-07	9.51E-05
**TENM2**	-1.67	0.32	1.12E-128	3.88E-126	**PLAT**	0.30	1.23	5.10E-07	1.02E-04
**TNFRSF10C**	5.36	41.05	2.15E-128	7.30E-126	**ETS1**	-0.28	0.83	5.41E-07	1.06E-04
**KLK10**	-1.43	0.37	2.54E-127	8.47E-125	**UBE2C**	-0.29	0.82	6.29E-07	1.21E-04
**MCM6**	-1.94	0.26	9.02E-127	2.95E-124	**EGR1**	0.31	1.24	6.38E-07	1.21E-04
**TOP2A**	-1.74	0.30	1.17E-126	3.77E-124	**IFI6**	-0.32	0.80	7.63E-07	1.42E-04
**LIMK2**	1.33	2.52	1.76E-126	5.56E-124	**LOXL2**	-0.43	0.74	8.92E-07	1.63E-04
**TMEM229B**	4.10	17.20	2.33E-126	7.22E-124	**MCL1**	0.21	1.15	1.23E-06	2.21E-04
**SLC7A11**	-3.08	0.12	2.68E-126	8.16E-124	**ACAT2**	-0.33	0.80	1.28E-06	2.26E-04
**XPC**	1.95	3.87	1.50E-125	4.50E-123	**FDPS**	-0.27	0.83	1.48E-06	2.58E-04
**RGS12**	1.52	2.87	3.34E-125	9.85E-123	**MKNK2**	-0.21	0.86	1.54E-06	2.63E-04
**TP53INP1**	3.12	8.72	5.69E-125	1.65E-122	**INSIG1**	-0.33	0.79	1.61E-06	2.70E-04
**SDPR**	2.21	4.64	8.99E-125	2.56E-122	**HMGB2**	-0.24	0.85	1.90E-06	3.14E-04
**FAM198B**	4.25	18.97	2.46E-124	6.90E-122	**AREG**	0.36	1.28	1.98E-06	3.21E-04
**PYGB**	-1.35	0.39	2.19E-123	6.06E-121	**SFPQ**	-0.22	0.86	2.17E-06	3.48E-04
**PHF19**	-2.15	0.22	7.58E-123	2.06E-120	**RPS6KA4**	-0.26	0.83	2.27E-06	3.58E-04
**FDXR**	1.85	3.61	9.94E-122	2.66E-119	**IFFO2**	-0.25	0.84	2.56E-06	3.91E-04
**PIDD1**	2.46	5.51	4.12E-121	1.09E-118	**ADAM8**	0.28	1.22	2.56E-06	3.91E-04
**NYNRIN**	1.78	3.44	5.04E-120	1.31E-117	**MCM7**	-0.21	0.86	2.79E-06	4.20E-04
**KIFC1**	-1.99	0.25	1.86E-118	4.77E-116	**NCAPG**	-0.29	0.82	2.97E-06	4.37E-04
**ARHGAP11A**	-1.97	0.26	1.83E-116	4.62E-114	**FAM111A**	-0.35	0.78	3.00E-06	4.37E-04
**TGFBI**	1.48	2.79	4.31E-114	1.07E-111	**UBTF**	-0.23	0.85	3.03E-06	4.37E-04
**NADSYN1**	1.67	3.17	1.08E-113	2.64E-111	**GM2A**	0.22	1.16	3.09E-06	4.39E-04
**HMMR**	-2.79	0.14	2.20E-113	5.33E-111	**CTDSP1**	-0.26	0.84	3.32E-06	4.66E-04
**KIF14**	-2.52	0.17	8.56E-112	2.05E-109	**ACLY**	-0.25	0.84	3.45E-06	4.77E-04
**MCM3**	-1.73	0.30	1.98E-111	4.66E-109	**P2RY2**	-0.28	0.82	3.80E-06	5.18E-04
**CENPE**	-2.07	0.24	2.90E-111	6.75E-109	**POLH**	0.29	1.22	3.95E-06	5.33E-04
**ZMAT3**	1.77	3.42	1.41E-109	3.25E-107	**H2AFX**	-0.28	0.82	4.16E-06	5.53E-04
**IL1B**	1.50	2.82	1.06E-108	2.39E-106	**MCM3**	-0.24	0.85	5.58E-06	7.32E-04
**FAM111B**	-2.74	0.15	5.91E-107	1.32E-104	**UHRF1**	-0.30	0.81	5.64E-06	7.32E-04
**NUSAP1**	-1.70	0.31	1.75E-106	3.87E-104	**CCNF**	-0.28	0.82	6.34E-06	8.09E-04
**KIF23**	-1.83	0.28	8.70E-105	1.90E-102	**SRSF6**	-0.24	0.85	6.39E-06	8.09E-04
**XPOT**	-1.27	0.41	1.36E-104	2.93E-102	**ANLN**	-0.22	0.86	7.06E-06	8.82E-04
**ANLN**	-1.43	0.37	2.38E-104	5.06E-102	**HJURP**	-0.27	0.83	8.94E-06	1.10E-03
**PTPRZ1**	-1.67	0.31	3.60E-104	7.57E-102	**JUN**	-0.28	0.82	9.26E-06	1.13E-03
**WDR63**	3.87	14.65	4.52E-104	9.39E-102	**NBR1**	0.23	1.17	9.39E-06	1.13E-03

PSRC1	-3.01	0.12	5.81E-100	1.19E-97
GALNT5	1.28	2.43	1.06E-99	2.16E-97
KRT8	1.58	2.98	1.28E-99	2.57E-97
ISYNA1	2.72	6.60	1.47E-99	2.92E-97
METTL7A	2.20	4.59	3.53E-98	6.93E-96
SLC35D1	1.56	2.95	1.16E-97	2.26E-95
PLK3	1.94	3.84	2.81E-97	5.41E-95
CCNF	-2.01	0.25	4.81E-97	9.13E-95
DEPDC1	-2.64	0.16	1.18E-95	2.22E-93
TAP1	1.63	3.09	4.06E-95	7.55E-93
CTGF	2.17	4.51	3.68E-94	6.78E-92
KIAA0040	1.25	2.38	1.38E-93	2.52E-91
IL36RN	1.61	3.05	2.78E-93	5.01E-91
RACGAP1	-1.52	0.35	3.57E-93	6.38E-91
KITLG	1.85	3.60	4.30E-93	7.61E-91
WDR76	-2.58	0.17	3.09E-92	5.41E-90
MLF2	1.18	2.27	1.75E-91	3.03E-89
FBXO2	2.33	5.02	1.51E-90	2.59E-88
AURKA	-1.76	0.30	2.99E-90	5.08E-88
AURKB	-1.97	0.26	8.55E-90	1.44E-87
SBSN	-1.34	0.40	1.62E-89	2.70E-87
CYFIP2	4.54	23.29	3.71E-89	6.13E-87
TEP1	1.74	3.34	6.94E-88	1.14E-85
HK2	-1.36	0.39	2.08E-87	3.37E-85
ZNF561	1.58	2.98	2.19E-87	3.52E-85
CA2	1.33	2.52	5.68E-87	9.04E-85
POLH	1.40	2.63	4.90E-86	7.74E-84
SULF2	1.05	2.07	1.77E-84	2.77E-82
CDCA8	-1.71	0.31	1.83E-83	2.84E-81
PHACTR3	-3.84	0.07	3.88E-83	5.97E-81
NOTCH1	1.29	2.44	6.53E-82	9.96E-80
ANP32B	-1.17	0.44	8.88E-82	1.34E-79
MKI67	-2.54	0.17	5.90E-80	8.84E-78
HSPA8	-1.01	0.50	9.22E-80	1.37E-77
CKAP2L	-1.80	0.29	2.09E-79	3.08E-77
EGR1	-1.70	0.31	8.60E-79	1.26E-76
TNFRSF14	2.48	5.59	7.46E-78	1.08E-75
LRPAP1	1.19	2.28	1.13E-77	1.62E-75
EPS8L2	1.17	2.25	1.24E-77	1.77E-75
CITED4	1.41	2.65	2.72E-76	3.85E-74
SLC3A2	-1.42	0.37	3.04E-76	4.26E-74
RRAD	1.68	3.20	3.20E-76	4.46E-74
ANK1	3.68	12.78	8.60E-76	1.19E-73
SMOC1	1.43	2.69	1.47E-75	2.01E-73
CDCA2	-2.02	0.25	1.83E-75	2.48E-73
CDCA3	-2.20	0.22	1.93E-75	2.60E-73
GPX1	1.36	2.57	1.95E-75	2.61E-73
TMEM173	1.55	2.92	9.28E-75	1.23E-72
GJB4	1.99	3.97	1.23E-74	1.62E-72
CYSRT1	1.63	3.09	1.51E-74	1.98E-72
TFPI2	-1.16	0.45	3.82E-74	4.96E-72
KIF11	-1.48	0.36	7.95E-74	1.03E-71
KIF18B	-1.86	0.28	1.18E-73	1.51E-71
SRSF1	-1.19	0.44	1.57E-73	1.99E-71
AKR1B10	2.65	6.28	6.25E-73	7.89E-71
SEMA3B	3.60	12.14	6.69E-73	8.39E-71
TUBB	-1.05	0.48	3.30E-72	4.10E-70
GPX3	1.10	2.14	5.81E-72	7.18E-70
SCARA3	-1.40	0.38	9.80E-72	1.20E-69
ETS1	-1.20	0.43	1.03E-71	1.25E-69
ELFN2	3.17	9.01	1.14E-71	1.38E-69
CPA4	1.11	2.15	4.62E-71	5.55E-69
COL7A1	1.21	2.31	7.38E-71	8.82E-69
INCENP	-1.52	0.35	9.07E-71	1.08E-68
DAPK1	2.17	4.49	3.13E-70	3.69E-68
KRT24	1.33	2.52	3.34E-70	3.91E-68
RP11-115D19.1	2.91	7.51	8.10E-70	9.41E-68
MR1	1.51	2.86	1.47E-69	1.70E-67
KIAA1217	1.21	2.32	1.87E-69	2.14E-67
XDH	1.18	2.27	2.02E-69	2.30E-67
UNG	-1.70	0.31	2.23E-69	2.52E-67
MCM7	-1.28	0.41	4.17E-69	4.70E-67
DEPDC1B	-2.05	0.24	9.51E-69	1.06E-66
RPL22L1	-1.68	0.31	1.65E-68	1.83E-66
FOS	1.51	2.84	1.77E-68	1.96E-66
KIF2C	-1.47	0.36	5.33E-68	5.85E-66
UBE2S	-1.91	0.27	5.37E-68	5.86E-66
SLC6A15	-1.66	0.32	8.13E-68	8.82E-66
AKR1C1	2.47	5.53	2.69E-67	2.90E-65
MFGE8	1.56	2.95	4.31E-67	4.62E-65
SPATA18	2.20	4.59	5.58E-67	5.94E-65
NDC80	-2.18	0.22	1.57E-66	1.66E-64
UHRF1	-1.78	0.29	1.89E-66	1.99E-64
STC2	-3.06	0.12	2.00E-66	2.09E-64
ZNF488	2.20	4.58	6.49E-66	6.75E-64
WNT9A	3.16	8.96	7.16E-66	7.40E-64
RPL3	-0.84	0.56	1.68E-65	1.72E-63
MCM5	-1.41	0.38	2.51E-65	2.56E-63
LFNG	-2.56	0.17	4.22E-65	4.29E-63
SPAG5	-1.50	0.35	5.78E-65	5.84E-63
DSG1	-0.27	0.83	1.04E-05	1.24E-03
SHC1	0.19	1.14	1.06E-05	1.25E-03
EGLN3	-0.38	0.77	1.17E-05	1.36E-03
FHL1	0.24	1.18	1.28E-05	1.48E-03
IFIT1	-0.38	0.77	1.34E-05	1.52E-03
DDB2	0.30	1.23	1.37E-05	1.54E-03
APLN	-0.36	0.78	1.38E-05	1.54E-03
DHCR24	-0.20	0.87	1.55E-05	1.71E-03
HMGCR	-0.26	0.84	1.63E-05	1.78E-03
FBXO5	-0.34	0.79	1.71E-05	1.84E-03
MLF2	0.19	1.14	1.98E-05	2.11E-03
TP53INP1	0.37	1.29	2.05E-05	2.16E-03
NCOA5	-0.30	0.81	2.09E-05	2.19E-03
BAG1	-0.27	0.83	2.16E-05	2.23E-03
SAT1	0.24	1.18	2.57E-05	2.63E-03
TCF19	-0.25	0.84	2.78E-05	2.82E-03
RP11-543P15.1	-0.35	0.79	2.95E-05	NA
KITLG	0.28	1.22	3.22E-05	3.22E-03
EGFR	-0.20	0.87	3.23E-05	3.22E-03
KIF23	-0.23	0.85	3.34E-05	3.28E-03
MCM4	-0.18	0.88	3.36E-05	3.28E-03
ARL4C	-0.20	0.87	3.65E-05	3.50E-03
CDC6	-0.24	0.85	3.66E-05	3.50E-03
SQLE	-0.24	0.85	3.77E-05	3.58E-03
ACTG1	-0.20	0.87	3.88E-05	3.64E-03
DPYSL2	-0.31	0.81	4.03E-05	3.74E-03
KIF18B	-0.26	0.83	4.05E-05	3.74E-03
BNIP3	-0.29	0.82	4.24E-05	3.88E-03
CDCA4	-0.25	0.84	4.34E-05	3.93E-03
BRCA1	-0.25	0.84	4.38E-05	3.93E-03
C6orf132	-0.18	0.88	4.46E-05	3.97E-03
KLF4	-0.25	0.84	4.93E-05	4.35E-03
PLK3	0.30	1.23	5.85E-05	5.10E-03
CSTB	-0.17	0.89	5.88E-05	5.10E-03
MOB3A	-0.22	0.86	5.94E-05	5.12E-03
UBE2S	-0.29	0.82	6.34E-05	5.41E-03
HERPUD1	-0.29	0.82	6.77E-05	5.73E-03
MKI67	-0.23	0.85	6.86E-05	5.75E-03
E2F8	-0.29	0.82	6.90E-05	5.75E-03
ZC3H4	-0.25	0.84	7.17E-05	5.92E-03
CALML5	-0.28	0.82	7.30E-05	5.98E-03
UBXN8	-0.34	0.79	7.50E-05	6.10E-03
CSF3	0.29	1.22	7.54E-05	NA
MYBL2	-0.22	0.86	7.68E-05	6.20E-03
AK4	-0.25	0.84	7.80E-05	6.24E-03
DDX60	-0.30	0.81	8.17E-05	6.49E-03
EBP	-0.23	0.85	8.76E-05	6.89E-03
PLK1	-0.19	0.88	8.81E-05	6.89E-03
MT2A	0.20	1.15	9.35E-05	7.22E-03
MXD4	0.32	1.25	9.37E-05	7.22E-03
NRARP	-0.31	0.81	9.57E-05	7.31E-03
PFKFB4	-0.32	0.80	9.64E-05	7.31E-03
FLG	-0.33	0.80	9.78E-05	NA
EPAS1	0.16	1.12	1.01E-04	7.63E-03
MALL	-0.29	0.82	1.11E-04	8.23E-03
MVK	-0.25	0.84	1.11E-04	8.23E-03
TICRR	-0.28	0.82	1.13E-04	8.36E-03
ADGRF1	-0.27	0.83	1.14E-04	8.36E-03
ITPKC	0.23	1.17	1.18E-04	8.54E-03
GTSE1	-0.27	0.83	1.18E-04	8.54E-03
DNER	-0.32	0.80	1.44E-04	1.03E-02
DHCR7	-0.22	0.86	1.46E-04	1.04E-02
ESPL1	-0.23	0.85	1.54E-04	1.09E-02
PFKFB3	-0.23	0.85	1.55E-04	1.09E-02
COA7	-0.24	0.85	1.57E-04	1.09E-02
SEMA5A	-0.29	0.82	1.79E-04	NA
PLCB3	-0.18	0.88	1.93E-04	1.33E-02
IGFL1	-0.28	0.82	2.20E-04	1.51E-02
KIF11	-0.22	0.86	2.25E-04	1.53E-02
AVPI1	0.23	1.18	2.25E-04	1.53E-02
HMGCS1	-0.32	0.80	2.30E-04	1.54E-02
KCTD11	-0.29	0.82	2.31E-04	1.54E-02
NRG1	-0.26	0.83	2.32E-04	1.54E-02
EMP2	-0.21	0.87	2.38E-04	1.57E-02
XYLB	-0.32	0.80	2.45E-04	1.61E-02
SCARA3	-0.20	0.87	2.51E-04	1.64E-02
NRP2	0.21	1.15	2.59E-04	1.68E-02
UNG	-0.24	0.85	2.61E-04	1.68E-02
PHF19	-0.22	0.86	2.68E-04	1.71E-02
PRKAR1B	-0.28	0.82	2.69E-04	1.71E-02
RGS2	0.19	1.14	2.73E-04	1.73E-02
TUFT1	-0.24	0.85	2.80E-04	1.76E-02
METRNL	-0.23	0.85	2.81E-04	1.76E-02
FDFT1	-0.20	0.87	2.85E-04	1.76E-02
UPP1	0.18	1.13	2.85E-04	1.76E-02
PTMA	-0.15	0.90	2.92E-04	1.79E-02
VIM	-0.25	0.84	3.03E-04	1.85E-02
FLRT2	-0.25	0.84	3.08E-04	1.87E-02
FBXO22	0.25	1.19	3.11E-04	1.88E-02
PTPRE	0.28	1.21	3.21E-04	1.92E-02

CKS2	-1.50	0.35	7.68E-65	7.71E-63
SFRP1	-1.01	0.50	8.77E-65	8.76E-63
GRN	1.19	2.28	2.79E-64	2.77E-62
CLU	1.74	3.34	8.26E-64	8.15E-62
CTSL	1.09	2.12	1.37E-63	1.35E-61
C10orf99	3.07	8.42	1.95E-63	1.90E-61
FAM46A	2.33	5.03	8.61E-63	8.36E-61
LACC1	2.13	4.37	1.35E-62	1.30E-60
KLK13	-1.36	0.39	1.39E-62	1.34E-60
TRIM38	1.48	2.80	1.75E-62	1.68E-60
DNAJB1	-0.97	0.51	1.85E-62	1.75E-60
ADAMTS7	5.25	38.16	3.42E-62	3.23E-60
ASNS	-1.13	0.46	5.98E-62	5.62E-60
RETSAT	1.14	2.20	6.98E-62	6.53E-60
FAM129A	-1.37	0.39	8.72E-62	8.11E-60
FLRT3	-1.19	0.44	9.51E-62	8.80E-60
TP63	-0.92	0.53	1.13E-61	1.04E-59
XBP1	-1.29	0.41	1.43E-61	1.31E-59
EIF4EBP1	-1.27	0.41	1.69E-61	1.54E-59
MXD1	1.71	3.26	2.92E-61	2.64E-59
USP11	1.57	2.97	3.16E-61	2.85E-59
WHSC1	-1.07	0.48	3.21E-61	2.88E-59
CSNK1G1	1.39	2.61	4.25E-61	3.79E-59
EDA2R	2.87	7.33	4.73E-61	4.20E-59
TMEM63B	1.12	2.18	6.35E-61	5.61E-59
KIF22	-1.24	0.42	6.98E-61	6.13E-59
CROT	1.59	3.01	7.64E-61	6.68E-59
SCN4B	3.02	8.10	8.72E-61	7.60E-59
IP6K2	1.23	2.35	1.29E-60	1.12E-58
PLAT	1.52	2.87	1.50E-60	1.29E-58
LAMP3	2.21	4.61	1.55E-60	1.33E-58
CCND2	1.04	2.05	1.65E-60	1.41E-58
RPS27L	1.50	2.82	2.03E-60	1.72E-58
IL20RB	-0.93	0.52	2.36E-60	1.99E-58
KIF4A	-1.51	0.35	2.59E-60	2.18E-58
CYGB	3.86	14.53	2.63E-60	2.20E-58
GAL3ST4	2.15	4.44	2.65E-60	2.21E-58
DGKA	1.01	2.01	2.95E-60	2.45E-58
NCAPG	-1.59	0.33	3.51E-60	2.90E-58
GADD45A	1.12	2.17	9.47E-60	7.79E-58
EPHA2	1.19	2.29	1.50E-59	1.22E-57
C21orf91	1.76	3.39	1.74E-59	1.42E-57
BTG3	1.27	2.41	2.18E-59	1.76E-57
CES3	2.61	6.09	4.92E-59	3.97E-57
HNRNPR	-0.96	0.51	6.76E-59	5.43E-57
BBC3	3.19	9.12	7.93E-59	6.34E-57
LMNB2	-1.06	0.48	1.53E-58	1.22E-56
GLTP	-1.12	0.46	2.23E-58	1.77E-56
DUSP5	1.11	2.17	6.98E-58	5.51E-56
MXD4	1.85	3.61	1.97E-57	1.55E-55
MT2A	-2.11	0.23	2.94E-57	2.30E-55
SIDT2	1.44	2.72	3.54E-57	2.76E-55
DDB2	1.32	2.49	4.89E-57	3.79E-55
HNRNPDL	-1.10	0.47	5.15E-57	3.98E-55
SULT2B1	-0.98	0.51	6.94E-57	5.33E-55
SLC39A6	0.97	1.95	7.44E-57	5.69E-55
SAPCD2	-1.57	0.34	1.50E-56	1.14E-54
GJB6	-1.34	0.40	1.96E-56	1.49E-54
EI24	0.82	1.77	2.55E-56	1.93E-54
FBXO22	1.25	2.38	2.63E-56	1.98E-54
RNASE7	2.63	6.19	2.69E-56	2.01E-54
MUC1	-1.47	0.36	2.91E-56	2.17E-54
CSF3	3.54	11.60	2.95E-56	2.19E-54
MAGED2	1.07	2.10	3.04E-56	2.24E-54
IL33	2.21	4.63	4.14E-56	3.05E-54
DFNB31	2.23	4.68	4.78E-56	3.50E-54
ACER2	2.70	6.52	4.80E-56	3.50E-54
STIL	-1.63	0.32	4.91E-56	3.57E-54
TOP1	0.99	1.98	1.00E-55	7.26E-54
KCTD15	-1.42	0.37	1.28E-55	9.20E-54
SLC2A1	-0.86	0.55	1.33E-55	9.53E-54
FGFR3	-1.00	0.50	1.41E-55	1.01E-53
GAA	1.44	2.71	3.21E-55	2.29E-53
08-mar	1.41	2.66	3.32E-55	2.35E-53
KLF7	-0.98	0.51	4.93E-55	3.49E-53
GTSE1	-1.74	0.30	5.80E-55	4.09E-53
SLC43A2	1.80	3.49	8.49E-55	5.95E-53
HNRNPF	-0.91	0.53	1.25E-54	8.75E-53
MSH6	-1.33	0.40	1.33E-54	9.26E-53
DBN1	-1.17	0.44	1.99E-54	1.38E-52
TFCP2L1	-1.04	0.49	2.21E-54	1.53E-52
ECE1	1.09	2.13	2.40E-54	1.65E-52
EMP2	-1.23	0.43	2.77E-54	1.90E-52
LCE1C	4.70	26.02	2.90E-54	1.98E-52
SLC30A1	1.43	2.70	3.60E-54	2.45E-52
FAM212B	1.33	2.51	3.74E-54	2.53E-52
ARNT2	3.30	9.83	5.31E-54	3.58E-52
FAM84B	1.01	2.01	7.05E-54	4.74E-52
TP53I3	1.22	2.33	1.20E-53	8.01E-52
SGOL2	-1.57	0.34	1.38E-53	9.23E-52

MAL	-0.31	0.81	3.35E-04	1.99E-02
LMNB1	-0.22	0.86	3.49E-04	2.06E-02
SRSF2	-0.21	0.87	3.59E-04	2.11E-02
BCLAF1	-0.17	0.89	3.63E-04	2.12E-02
CXCL1	-0.31	0.81	3.64E-04	2.12E-02
PDLIM2	-0.24	0.85	3.81E-04	2.21E-02
CLSPN	-0.27	0.83	3.99E-04	2.29E-02
ATF4	-0.21	0.86	4.00E-04	2.29E-02
CHAF1A	-0.22	0.86	4.12E-04	2.35E-02
HAGH	0.27	1.21	4.20E-04	2.38E-02
KRT31	-0.31	0.81	4.35E-04	2.45E-02
SLC41A1	0.22	1.17	4.39E-04	2.45E-02
CDCA5	-0.22	0.86	4.40E-04	2.45E-02
GJA1	-0.17	0.89	4.47E-04	2.47E-02
PKMYT1	-0.23	0.85	4.76E-04	2.62E-02
FANCI	-0.20	0.87	4.83E-04	2.64E-02
CH17-360D5.3	-0.29	0.82	4.86E-04	2.65E-02
PNMAL1	0.27	1.21	4.90E-04	2.66E-02
LRAT	-0.29	0.82	4.95E-04	2.66E-02
ID4	-0.30	0.81	4.95E-04	2.66E-02
MMP14	0.16	1.12	5.21E-04	2.78E-02
ZFC3H1	0.25	1.19	5.30E-04	2.81E-02
THBD	-0.17	0.89	5.35E-04	2.82E-02
SLC38A5	-0.21	0.87	5.38E-04	2.82E-02
RCC2	-0.16	0.90	5.42E-04	2.83E-02
DFNA5	0.20	1.15	5.49E-04	2.84E-02
DNAJB5	0.26	1.20	5.50E-04	2.84E-02
S100A3	0.29	1.22	5.60E-04	2.88E-02
CAMK2N1	-0.19	0.88	5.75E-04	2.94E-02
E2F2	-0.28	0.83	5.84E-04	2.98E-02
RACGAP1	-0.17	0.89	5.90E-04	2.99E-02
SYDE1	-0.28	0.82	6.02E-04	3.04E-02
PVRL1	-0.13	0.92	6.12E-04	3.07E-02
KIF24	-0.29	0.82	6.33E-04	3.16E-02
RP11-800A3.4	-0.24	0.85	6.60E-04	3.28E-02
CDCA3	-0.24	0.85	6.74E-04	3.33E-02
SLFN5	0.20	1.15	6.79E-04	3.33E-02
KRT6C	-0.24	0.85	6.80E-04	3.33E-02
LAMTOR2	0.26	1.19	6.83E-04	3.33E-02
FGFR3	-0.15	0.90	6.85E-04	3.33E-02
TMEM63B	0.19	1.14	7.30E-04	3.52E-02
WDR63	0.27	1.21	7.37E-04	NA
PRR11	-0.18	0.88	7.39E-04	3.55E-02
DSG4	-0.28	0.82	7.43E-04	3.56E-02
ARRDC3	-0.24	0.85	7.48E-04	3.56E-02
SLC1A6	-0.24	0.85	7.57E-04	NA
HNRNPDL	-0.17	0.89	7.66E-04	3.63E-02
ALDOA	-0.15	0.90	7.77E-04	3.67E-02
LINC00511	-0.29	0.82	7.88E-04	3.67E-02
SLC29A1	-0.23	0.85	7.89E-04	3.67E-02
PLA2G3	-0.29	0.82	7.90E-04	3.67E-02
RBL1	-0.25	0.84	7.95E-04	3.67E-02
CKAP2L	-0.20	0.87	8.00E-04	3.67E-02
P4HA1	-0.26	0.84	8.00E-04	3.67E-02
ZFAS1	0.19	1.14	8.07E-04	3.69E-02
RP11-421F16.3	0.29	1.22	8.18E-04	3.72E-02
PRKX	0.23	1.17	8.23E-04	3.73E-02
RECQL4	-0.23	0.85	8.31E-04	3.74E-02
CNTNAP2	-0.29	0.82	8.36E-04	3.74E-02
TONSL	-0.22	0.86	8.36E-04	3.74E-02
RRAD	-0.26	0.84	8.47E-04	3.77E-02
PRIM1	-0.27	0.83	8.53E-04	3.78E-02
BHLHE40	-0.16	0.90	8.56E-04	3.78E-02
TOB1	0.20	1.15	8.68E-04	3.82E-02
FOSL2	-0.16	0.90	8.73E-04	3.82E-02
NLRP1	0.23	1.17	8.97E-04	3.89E-02
CLIC3	-0.23	0.86	8.98E-04	3.89E-02
U2AF2	-0.16	0.89	9.00E-04	3.89E-02
C19orf33	-0.28	0.82	9.20E-04	3.94E-02
TNC	-0.20	0.87	9.23E-04	3.94E-02
TINF2	0.21	1.15	9.25E-04	3.94E-02
TNFRSF14	0.29	1.22	9.28E-04	3.94E-02
IFI44L	-0.27	0.83	9.29E-04	NA
WDR76	-0.23	0.85	9.59E-04	4.06E-02
SRSF1	-0.17	0.89	9.68E-04	4.06E-02
GSS	0.18	1.13	9.68E-04	4.06E-02
ALDH7A1	0.18	1.14	9.71E-04	4.06E-02
NR2F2	-0.21	0.87	9.76E-04	4.06E-02
USP1	-0.20	0.87	9.95E-04	4.13E-02
USP20	0.25	1.19	1.00E-03	4.13E-02
FAM212B	0.24	1.18	1.02E-03	4.18E-02
IP6K2	0.21	1.16	1.04E-03	4.28E-02
TSKU	-0.20	0.87	1.06E-03	4.31E-02
PKP3	-0.14	0.91	1.07E-03	4.35E-02
MEX3D	-0.26	0.84	1.11E-03	4.48E-02
MAOA	0.20	1.15	1.12E-03	4.51E-02
CCNG1	0.22	1.16	1.12E-03	4.52E-02
KREMEN1	-0.17	0.89	1.13E-03	4.53E-02
CSNK1E	0.17	1.12	1.14E-03	4.55E-02
PAQR7	0.23	1.17	1.16E-03	4.60E-02

TBC1D2	1.12	2.18	2.10E-53	1.40E-51
GAS2L3	-3.27	0.10	2.37E-53	1.57E-51
PI4K2A	1.26	2.39	3.01E-53	1.99E-51
SLBP	-1.22	0.43	3.50E-53	2.30E-51
BCAT1	-1.60	0.33	4.84E-53	3.17E-51
ECT2	-1.34	0.40	9.76E-53	6.36E-51
SLC1A5	-1.23	0.43	2.64E-52	1.71E-50
SMIM10L2A	4.24	18.92	4.82E-52	3.12E-50
AVPI1	1.19	2.28	5.61E-52	3.62E-50
AARS	-1.07	0.48	7.28E-52	4.68E-50
HNRNPD	-1.00	0.50	8.41E-52	5.39E-50
NCAPH	-1.34	0.40	9.33E-52	5.95E-50
SRSF7	-0.97	0.51	1.08E-51	6.87E-50
ANXA1	-0.91	0.53	1.16E-51	7.34E-50
CDT1	-1.56	0.34	1.24E-51	7.81E-50
NFIX	-1.28	0.41	1.68E-51	1.06E-49
ITM2B	1.09	2.13	2.29E-51	1.44E-49
CIT	-1.35	0.39	2.66E-51	1.66E-49
SEMA3G	4.41	21.20	3.74E-51	2.33E-49
TOB1	1.07	2.10	4.13E-51	2.56E-49
TSPAN14	0.97	1.96	4.69E-51	2.90E-49
RAD51C	1.43	2.70	6.36E-51	3.92E-49
CES2	0.96	1.95	6.88E-51	4.22E-49
EIF4B	-0.86	0.55	7.05E-51	4.31E-49
GPSM2	-1.10	0.47	7.19E-51	4.38E-49
ASS1	-1.65	0.32	7.37E-51	4.47E-49
PAQR7	1.30	2.46	8.07E-51	4.89E-49
AREG	1.50	2.83	8.55E-51	5.16E-49
NLRP1	1.27	2.42	8.82E-51	5.30E-49
TTK	-1.49	0.36	1.63E-50	9.74E-49
PARP1	-0.88	0.54	1.84E-50	1.10E-48
PTPRF	-0.89	0.54	1.93E-50	1.15E-48
SLC29A1	-1.45	0.37	3.34E-50	1.98E-48
CENPA	-2.56	0.17	3.75E-50	2.22E-48
SRSF2	-1.08	0.47	6.43E-50	3.79E-48
CDCA7	-1.24	0.42	7.52E-50	4.42E-48
SPTLC2	-1.12	0.46	8.15E-50	4.77E-48
HSPA5	-0.79	0.58	1.29E-49	7.50E-48
SPTBN1	-0.86	0.55	1.75E-49	1.02E-47
FAS	1.44	2.71	2.47E-49	1.43E-47
ABCA12	0.98	1.97	3.13E-49	1.81E-47
FAM84A	1.08	2.11	3.46E-49	1.99E-47
KRBA1	1.73	3.31	4.36E-49	2.50E-47
BTBD19	3.20	9.20	4.43E-49	2.54E-47
DKK3	0.97	1.96	5.80E-49	3.31E-47
CSF1	3.19	9.11	2.77E-48	1.58E-46
CDKN3	-1.89	0.27	2.80E-48	1.59E-46
RP11-597D13.9	3.57	11.85	3.30E-48	1.87E-46
ATF4	-0.97	0.51	3.35E-48	1.89E-46
CALML3-AS1	2.90	7.45	3.65E-48	2.05E-46
GARS	-0.97	0.51	3.76E-48	2.10E-46
HSPA4L	1.04	2.06	1.48E-47	8.27E-46
LRP1	1.20	2.29	1.57E-47	8.75E-46
FKBP5	-1.03	0.49	1.61E-47	8.92E-46
NABP1	1.50	2.83	1.92E-47	1.06E-45
H2AFZ	-0.95	0.52	4.64E-47	2.56E-45
PAICS	-0.83	0.56	5.83E-47	3.20E-45
ARSA	1.29	2.44	6.95E-47	3.80E-45
TAB3	1.18	2.26	7.06E-47	3.85E-45
GINS2	-1.45	0.37	7.86E-47	4.28E-45
SKP2	-1.14	0.45	9.93E-47	5.38E-45
RP11-369C8.1	5.16	35.78	1.42E-46	7.66E-45
SPAG1	1.60	3.02	1.86E-46	1.00E-44
CASZ1	1.22	2.32	2.39E-46	1.28E-44
ANKRD65	1.81	3.51	5.04E-46	2.70E-44
SDC4	0.76	1.69	7.41E-46	3.96E-44
CTDSP1	-1.19	0.44	7.91E-46	4.21E-44
SYTL2	2.14	4.41	8.37E-46	4.44E-44
TMEM97	-1.48	0.36	9.15E-46	4.84E-44
NFIC	-1.03	0.49	9.23E-46	4.87E-44
XG	1.39	2.62	1.51E-45	7.94E-44
RTN4RL1	-1.58	0.33	1.65E-45	8.67E-44
LBR	-1.14	0.45	5.23E-45	2.73E-43
SMC4	-1.09	0.47	6.26E-45	3.26E-43
RGS16	3.24	9.47	9.82E-45	5.10E-43
TIMP3	-0.95	0.52	1.01E-44	5.22E-43
SSRP1	-0.82	0.57	1.02E-44	5.23E-43
SLAMF7	1.64	3.12	1.02E-44	5.23E-43
NUP62	-1.07	0.48	1.02E-44	5.24E-43
SYTL1	1.08	2.12	1.03E-44	5.30E-43
FABP5	-1.18	0.44	1.44E-44	7.35E-43
LTBP1	-0.95	0.52	1.44E-44	7.35E-43
NUMBL	-1.71	0.31	1.76E-44	8.95E-43
BIRC5	-1.84	0.28	1.90E-44	9.62E-43
NUPR1	1.29	2.45	2.76E-44	1.39E-42
CASC5	-1.46	0.36	3.00E-44	1.51E-42
ARHGEF4	0.91	1.87	3.05E-44	1.53E-42
DUT	-1.33	0.40	4.31E-44	2.16E-42
PLA2G4D	3.27	9.64	4.55E-44	2.27E-42
GRK6	-1.33	0.40	7.99E-44	3.98E-42

CRABP2	-0.15	0.90	1.16E-03	4.60E-02
TRIB3	-0.22	0.86	1.17E-03	4.60E-02
IFRD1	-0.23	0.85	1.19E-03	4.68E-02
SEC14L2	0.19	1.14	1.20E-03	4.68E-02
MCM5	-0.16	0.89	1.20E-03	4.69E-02
GPI	-0.16	0.90	1.21E-03	4.71E-02
PRR5L	-0.26	0.84	1.22E-03	4.72E-02
ARID1A	-0.19	0.88	1.24E-03	4.76E-02
MAPKBP1	0.19	1.14	1.26E-03	4.83E-02
BRCA2	-0.26	0.83	1.26E-03	4.83E-02
RARRES1	-0.26	0.83	1.29E-03	NA
ZWINT	-0.19	0.87	1.32E-03	5.00E-02
CA12	-0.15	0.90	1.32E-03	5.00E-02
ERO1A	-0.19	0.88	1.32E-03	5.00E-02
TMEM229B	0.26	1.20	1.33E-03	NA
PCYT2	-0.21	0.86	1.33E-03	5.00E-02
FUT11	-0.27	0.83	1.33E-03	5.00E-02
CDCA8	-0.19	0.88	1.34E-03	5.01E-02
CAV1	-0.13	0.92	1.35E-03	5.01E-02
POLD1	-0.20	0.87	1.35E-03	5.01E-02
PABPC1L	0.28	1.21	1.35E-03	5.01E-02
TPX2	-0.14	0.91	1.39E-03	5.13E-02
TUBB	-0.13	0.91	1.43E-03	5.24E-02
H6PD	0.20	1.15	1.45E-03	5.30E-02
AC005019.2	-0.14	0.90	1.46E-03	NA
IDI1	-0.20	0.87	1.49E-03	5.43E-02
AURKB	-0.19	0.88	1.51E-03	5.48E-02
ARHGEF2	0.17	1.13	1.54E-03	5.59E-02
BLOC1S2	0.22	1.16	1.59E-03	5.72E-02
ASS1	0.21	1.16	1.61E-03	5.79E-02
PNRC1	0.26	1.20	1.66E-03	5.93E-02
PPARD	-0.17	0.89	1.68E-03	5.99E-02
HSPB1	-0.14	0.90	1.68E-03	5.99E-02
KRT24	-0.22	0.86	1.73E-03	6.11E-02
SKA1	-0.25	0.84	1.73E-03	6.11E-02
RINL	0.27	1.21	1.75E-03	6.15E-02
PHF20	0.19	1.14	1.76E-03	6.17E-02
FANCD2	-0.21	0.86	1.78E-03	6.21E-02
CD44	0.13	1.09	1.78E-03	6.21E-02
MAFB	-0.27	0.83	1.80E-03	6.24E-02
TPBG	-0.19	0.88	1.80E-03	6.24E-02
HSPA4L	0.17	1.13	1.82E-03	6.26E-02
ABCG1	0.27	1.21	1.82E-03	6.26E-02
ELMSAN1	-0.19	0.88	1.84E-03	6.29E-02
MIR210HG	-0.27	0.83	1.86E-03	6.33E-02
SPRR3	-0.27	0.83	1.86E-03	6.33E-02
COL5A3	-0.25	0.84	1.89E-03	6.42E-02
MARVELD1	0.18	1.13	1.92E-03	6.46E-02
TERF2IP	0.21	1.16	1.92E-03	6.46E-02
MMP9	-0.22	0.86	1.93E-03	6.47E-02
SAMHD1	-0.25	0.84	1.98E-03	6.63E-02
CA9	-0.26	0.84	1.99E-03	6.63E-02
DTYMK	-0.20	0.87	1.99E-03	6.63E-02
SCRIB	0.16	1.12	2.00E-03	6.63E-02
NEDD4	-0.20	0.87	2.04E-03	6.72E-02
TIGAR	0.23	1.17	2.10E-03	6.92E-02
ERRFI1	-0.16	0.89	2.12E-03	6.96E-02
KLK13	-0.20	0.87	2.13E-03	6.98E-02
MARK4	0.19	1.14	2.17E-03	7.07E-02
RNF123	0.23	1.17	2.18E-03	7.09E-02
MTHFD1	-0.15	0.90	2.19E-03	7.09E-02
PTBP1	-0.15	0.90	2.21E-03	7.13E-02
MARCKS	-0.17	0.89	2.22E-03	7.15E-02
ATAD3B	-0.20	0.87	2.23E-03	7.15E-02
DNMT1	-0.15	0.90	2.25E-03	7.21E-02
KPRP	-0.25	0.84	2.25E-03	NA
PGP	-0.22	0.86	2.28E-03	7.27E-02
GAPDH	-0.11	0.93	2.29E-03	7.27E-02
CSNK1G1	0.21	1.16	2.31E-03	7.29E-02
SSH3	0.17	1.12	2.32E-03	7.29E-02
TRIM8	0.18	1.14	2.32E-03	7.29E-02
SYNM	-0.26	0.84	2.33E-03	7.29E-02
DSE	0.15	1.11	2.33E-03	7.29E-02
POLD3	-0.20	0.87	2.34E-03	7.31E-02
CENPO	-0.24	0.85	2.35E-03	7.31E-02
SLC3A2	-0.17	0.89	2.36E-03	7.31E-02
PLA2G4E	-0.23	0.85	2.37E-03	7.33E-02
ICA1	0.24	1.18	2.37E-03	NA
KIRREL	-0.16	0.90	2.39E-03	7.35E-02
CYR61	0.20	1.15	2.39E-03	7.35E-02
RFX5	0.24	1.18	2.42E-03	7.42E-02
HNRNPA1P33	-0.26	0.83	2.46E-03	7.49E-02
MELK	-0.17	0.89	2.47E-03	7.49E-02
PTGS1	-0.25	0.84	2.47E-03	7.49E-02
CMTM7	0.21	1.16	2.50E-03	7.56E-02
CBX5	-0.17	0.89	2.51E-03	7.58E-02
ITPR1	-0.26	0.83	2.54E-03	7.64E-02
TGM1	0.11	1.08	2.59E-03	7.78E-02
ILVBL	-0.18	0.88	2.61E-03	7.81E-02
BAIAP2L2	-0.26	0.83	2.62E-03	7.81E-02

**ANP32E**	-1.12	0.46	9.04E-44	4.49E-42
**HIST2H2BE**	2.14	4.42	9.90E-44	4.90E-42
**APLN**	-2.12	0.23	1.60E-43	7.90E-42
**GPC1**	0.87	1.83	1.91E-43	9.40E-42
**GPX8**	-2.04	0.24	2.08E-43	1.02E-41
**HDGF**	-0.69	0.62	2.19E-43	1.07E-41
**MMP14**	0.84	1.80	2.34E-43	1.14E-41
**FHL2**	1.15	2.22	2.44E-43	1.19E-41
**MTUS1**	-1.26	0.42	2.44E-43	1.19E-41
**DAZAP1**	-0.82	0.57	2.48E-43	1.20E-41
**SORBS3**	-1.08	0.47	3.53E-43	1.70E-41
**SPRR1B**	-0.93	0.52	3.62E-43	1.75E-41
**BLOC1S2**	1.15	2.22	3.81E-43	1.83E-41
**STMN1**	-0.85	0.56	3.89E-43	1.86E-41
**ANO1**	-1.88	0.27	3.98E-43	1.90E-41
**RNF220**	-1.24	0.42	4.36E-43	2.08E-41
**AGBL5**	1.16	2.24	4.40E-43	2.09E-41
**LAD1**	-0.78	0.58	5.03E-43	2.38E-41
**NRARP**	-1.89	0.27	6.05E-43	2.86E-41
**ITM2A**	1.55	2.94	9.11E-43	4.29E-41
**IQGAP3**	-1.30	0.41	9.72E-43	4.57E-41
**C2orf54**	-1.41	0.38	9.88E-43	4.63E-41
**HERC5**	2.26	4.79	2.01E-42	9.37E-41
**FUT3**	-1.11	0.46	2.02E-42	9.40E-41
**PTHLH**	-1.03	0.49	2.10E-42	9.75E-41
**KRT6C**	1.08	2.11	2.16E-42	1.00E-40
**CLMP**	-1.69	0.31	2.42E-42	1.12E-40
**ARHGAP23**	-0.96	0.51	2.66E-42	1.23E-40
**PPM1D**	1.55	2.93	3.49E-42	1.61E-40
**RAC3**	-2.13	0.23	3.87E-42	1.78E-40
**MT1E**	-1.31	0.40	4.88E-42	2.24E-40
**KCTD11**	1.33	2.52	5.04E-42	2.30E-40
**NRP2**	0.89	1.85	5.91E-42	2.69E-40
**UBL4A**	-1.18	0.44	8.32E-42	3.78E-40
**TRAF4**	1.06	2.08	9.08E-42	4.12E-40
**MICALL2**	1.40	2.64	1.15E-41	5.18E-40
**CDH4**	-1.60	0.33	1.19E-41	5.37E-40
**ARHGAP19**	-1.64	0.32	1.29E-41	5.80E-40
**UBE2H**	0.90	1.87	1.65E-41	7.42E-40
**FOXM1**	-1.10	0.47	2.28E-41	1.02E-39
**KIF20B**	-1.22	0.43	2.29E-41	1.02E-39
**OSTM1**	1.36	2.57	2.56E-41	1.14E-39
**DOCK1**	-1.19	0.44	2.85E-41	1.27E-39
**COL4A5**	0.94	1.92	2.90E-41	1.29E-39
**HIST1H2AC**	1.80	3.49	5.84E-41	2.58E-39
**AIF1L**	-1.66	0.32	6.27E-41	2.76E-39
**MCM2**	-1.18	0.44	6.30E-41	2.77E-39
**RFX5**	1.40	2.65	7.41E-41	3.25E-39
**MDC1**	-1.07	0.48	7.52E-41	3.29E-39
**MYADM**	-1.35	0.39	8.76E-41	3.82E-39
**ZNF195**	1.44	2.71	1.40E-40	6.09E-39
**PML**	1.03	2.05	1.41E-40	6.12E-39
**SPIN4**	1.45	2.72	1.43E-40	6.17E-39
**MRPL39**	1.05	2.07	1.49E-40	6.45E-39
**DOCK8**	1.92	3.79	1.84E-40	7.91E-39
**NTN1**	4.22	18.66	1.87E-40	8.05E-39
**ABHD4**	1.26	2.39	2.13E-40	9.14E-39
**DHX15**	-0.74	0.60	2.66E-40	1.14E-38
**SQLE**	-1.07	0.48	2.93E-40	1.25E-38
**SLC38A1**	-0.79	0.58	3.77E-40	1.61E-38
**BAX**	0.92	1.89	4.24E-40	1.80E-38
**BICD2**	0.74	1.67	5.61E-40	2.38E-38
**PRKAB1**	1.23	2.35	5.98E-40	2.53E-38
**PYCR1**	-1.38	0.38	6.84E-40	2.88E-38
**TNK1**	1.39	2.62	7.96E-40	3.35E-38
**SLC16A2**	0.98	1.97	1.09E-39	4.60E-38
**TICRR**	-1.45	0.37	1.96E-39	8.20E-38
**FHL1**	-0.96	0.51	2.01E-39	8.38E-38
**SET**	-0.76	0.59	2.47E-39	1.03E-37
**IFI27**	0.97	1.96	2.97E-39	1.23E-37
**CDK5R1**	-1.31	0.40	3.12E-39	1.30E-37
**HSP90B1**	-0.73	0.60	3.20E-39	1.33E-37
**GPR87**	1.09	2.13	5.30E-39	2.19E-37
**ALDH7A1**	0.88	1.84	5.64E-39	2.32E-37
**FASN**	-0.96	0.51	5.84E-39	2.40E-37
**EGFR**	-0.74	0.60	8.54E-39	3.50E-37
**TMEM123**	-0.98	0.51	9.95E-39	4.07E-37
**RPL10A**	-0.70	0.62	1.16E-38	4.72E-37
**FBXO5**	-1.85	0.28	1.35E-38	5.51E-37
**WSB1**	1.02	2.03	1.49E-38	6.05E-37
**CSPG4**	1.94	3.85	1.72E-38	6.98E-37
**THBS2**	-0.89	0.54	1.81E-38	7.34E-37
**RCC1**	-0.90	0.54	2.69E-38	1.09E-36
**EHBP1L1**	-1.01	0.50	2.80E-38	1.13E-36
**RNPEP**	0.89	1.85	2.81E-38	1.13E-36
**PLAU**	0.97	1.96	3.27E-38	1.31E-36
**RRP1B**	-0.87	0.55	3.42E-38	1.37E-36
**PAQR4**	-1.58	0.34	3.53E-38	1.41E-36
**CD274**	1.97	3.91	4.38E-38	1.74E-36
**DPYSL4**	4.12	17.44	4.45E-38	1.77E-36
**CDC20**	-0.15	0.90	2.64E-03	7.86E-02
**ADIPOR1**	0.13	1.10	2.66E-03	7.90E-02
**BCL11B**	-0.21	0.86	2.69E-03	7.93E-02
**SDK2**	0.23	1.17	2.69E-03	7.93E-02
**ID2**	-0.26	0.84	2.72E-03	8.00E-02
**KIFC1**	-0.16	0.89	2.72E-03	8.00E-02
**PHGDH**	0.15	1.11	2.78E-03	8.12E-02
**NGFR**	0.24	1.18	2.78E-03	8.12E-02
**WDTC1**	0.18	1.14	2.80E-03	8.14E-02
**ABCA7**	0.21	1.15	2.82E-03	8.19E-02
**BDH1**	-0.19	0.87	2.85E-03	8.24E-02
**EVI5L**	0.19	1.14	2.86E-03	8.24E-02
**PKP1**	-0.11	0.93	2.87E-03	8.27E-02
**SATB1**	0.26	1.20	2.90E-03	8.32E-02
**HES4**	-0.26	0.84	2.91E-03	8.33E-02
**PEG10**	-0.19	0.88	2.96E-03	NA
**ARHGAP21**	-0.17	0.89	2.96E-03	8.46E-02
**EXO1**	-0.23	0.85	3.02E-03	8.58E-02
**LGALSL**	-0.18	0.88	3.02E-03	8.58E-02
**PPM1B**	0.22	1.16	3.06E-03	8.61E-02
**MCM2**	-0.16	0.89	3.06E-03	8.61E-02
**LFNG**	-0.23	0.86	3.06E-03	8.61E-02
**LMNB2**	-0.14	0.90	3.07E-03	8.61E-02
**WHSC1**	-0.13	0.91	3.08E-03	8.61E-02
**HNRNPU**	-0.12	0.92	3.10E-03	8.65E-02
**EML2**	0.16	1.12	3.12E-03	8.67E-02
**CENPN**	-0.24	0.85	3.16E-03	8.76E-02
**UNC13D**	-0.23	0.85	3.17E-03	8.76E-02
**LAMB3**	0.16	1.11	3.17E-03	8.76E-02
**NBEAL2**	0.13	1.10	3.18E-03	8.77E-02
**SPTSSA**	0.22	1.16	3.23E-03	8.87E-02
**SOAT1**	-0.19	0.88	3.25E-03	8.89E-02
**MRTO4**	-0.16	0.89	3.29E-03	8.99E-02
**FEN1**	-0.17	0.89	3.31E-03	9.01E-02
**TTK**	-0.19	0.87	3.38E-03	9.17E-02
**RBBP8NL**	-0.25	0.84	3.43E-03	9.31E-02
**SLC35D1**	0.19	1.14	3.47E-03	9.36E-02
**ADRB2**	-0.22	0.86	3.47E-03	9.36E-02
**ELOVL6**	-0.17	0.89	3.53E-03	9.49E-02
**EIF1AD**	-0.21	0.86	3.55E-03	9.52E-02
**ETS2**	-0.15	0.90	3.63E-03	9.70E-02
**MCM10**	-0.19	0.88	3.64E-03	9.71E-02
**UBLCP1**	0.20	1.15	3.66E-03	9.73E-02
**C14orf1**	-0.18	0.88	3.68E-03	9.75E-02
**XBP1**	-0.18	0.88	3.69E-03	9.75E-02
**SERPINE1**	-0.13	0.91	3.71E-03	9.76E-02
**MPZL2**	0.14	1.11	3.71E-03	9.76E-02
**YPEL5**	0.19	1.14	3.72E-03	9.76E-02
**STIL**	-0.20	0.87	3.74E-03	9.76E-02
**CDK1**	-0.18	0.88	3.75E-03	9.76E-02
**DFNB31**	0.25	1.19	3.76E-03	9.76E-02
**AMZ2**	0.17	1.13	3.76E-03	9.76E-02
**ZNF561**	0.19	1.14	3.80E-03	9.85E-02
**MAP4K4**	0.13	1.09	3.83E-03	9.87E-02
**PRKCDBP**	-0.21	0.87	3.83E-03	9.87E-02
**CLTA**	-0.14	0.91	3.84E-03	9.87E-02
**LRRC23**	0.24	1.18	3.86E-03	NA
**SYNCRIP**	-0.13	0.91	3.92E-03	1.01E-01
**SLC6A11**	-0.19	0.88	3.95E-03	1.01E-01
**CENPI**	-0.23	0.85	4.01E-03	1.02E-01
**RPS27L**	0.22	1.16	4.03E-03	1.02E-01
**KRTDAP**	-0.16	0.89	4.03E-03	1.02E-01
**PRPF4**	-0.16	0.89	4.04E-03	1.02E-01
**APBB2**	0.18	1.13	4.08E-03	1.03E-01
**NRM**	-0.23	0.85	4.08E-03	1.03E-01
**CD3EAP**	-0.25	0.84	4.11E-03	1.03E-01
**ASF1B**	-0.20	0.87	4.13E-03	1.04E-01
**OSTM1**	0.23	1.17	4.16E-03	1.04E-01
**HLA-G**	-0.25	0.84	4.17E-03	1.04E-01
**TK1**	-0.18	0.88	4.19E-03	1.04E-01
**MTHFD2**	-0.19	0.87	4.21E-03	1.05E-01
**PTPN6**	0.21	1.16	4.22E-03	1.05E-01
**PRODH**	-0.22	0.86	4.28E-03	1.06E-01
**FNBP4**	0.17	1.13	4.37E-03	1.08E-01
**ST6GAL1**	0.22	1.17	4.46E-03	1.10E-01
**MAT2A**	-0.14	0.91	4.48E-03	1.10E-01
**DUT**	-0.19	0.88	4.50E-03	1.10E-01
**WEE1**	-0.22	0.86	4.53E-03	1.11E-01
**ORC1**	-0.20	0.87	4.56E-03	1.11E-01
**ZNF703**	-0.22	0.86	4.59E-03	1.12E-01
**SLC12A4**	0.18	1.13	4.61E-03	1.12E-01
**SDC3**	-0.17	0.89	4.61E-03	1.12E-01
**RMI1**	-0.23	0.85	4.64E-03	1.12E-01
**HSPB8**	-0.19	0.88	4.69E-03	1.13E-01
**RPS23**	-0.18	0.88	4.72E-03	1.13E-01
**GINS2**	-0.19	0.87	4.76E-03	1.14E-01
**PGK1**	-0.12	0.92	4.77E-03	1.14E-01
**PDK1**	-0.20	0.87	4.78E-03	1.14E-01
**TEP1**	0.21	1.16	4.78E-03	1.14E-01
**WARS**	0.14	1.10	4.79E-03	1.14E-01

TFAP4	-1.68	0.31	5.68E-38	2.25E-36
PKD1	-1.32	0.40	5.77E-38	2.28E-36
PARP10	1.44	2.71	6.15E-38	2.43E-36
TNFSF9	1.52	2.86	6.26E-38	2.46E-36
04-mar	1.38	2.59	6.33E-38	2.49E-36
SDSL	2.08	4.23	6.86E-38	2.69E-36
KNSTRN	-1.17	0.44	9.34E-38	3.65E-36
PIF1	-2.56	0.17	1.05E-37	4.10E-36
CEL	2.88	7.35	1.13E-37	4.42E-36
DMKN	-0.80	0.58	1.62E-37	6.31E-36
KPNB1	-0.72	0.61	1.83E-37	7.10E-36
TCF3	-0.91	0.53	1.85E-37	7.17E-36
MCM4	-0.95	0.52	2.10E-37	8.12E-36
CNTRL	-1.54	0.34	2.17E-37	8.37E-36
VWCE	4.01	16.12	2.58E-37	9.92E-36
KIF15	-1.57	0.34	3.57E-37	1.37E-35
OVOL1	1.23	2.34	4.22E-37	1.61E-35
LRRC8A	-0.74	0.60	4.23E-37	1.62E-35
ABTB2	1.72	3.29	4.28E-37	1.63E-35
STAT2	1.07	2.10	5.33E-37	2.03E-35
EFNA3	-1.90	0.27	6.60E-37	2.50E-35
TRA2B	-0.75	0.59	7.96E-37	3.01E-35
RP11-363E7.4	2.84	7.14	1.61E-36	6.08E-35
MOV10	0.99	1.99	1.64E-36	6.19E-35
ATF3	2.72	6.57	1.83E-36	6.88E-35
HIF1A	0.75	1.68	2.12E-36	7.94E-35
ZNF185	0.70	1.62	2.52E-36	9.42E-35
MROH6	-1.20	0.43	2.88E-36	1.08E-34
TNFRSF10A	1.10	2.14	3.07E-36	1.14E-34
S1PR5	-1.14	0.45	3.37E-36	1.25E-34
ZNF395	-1.10	0.46	4.84E-36	1.80E-34
SF3B3	-0.70	0.62	5.20E-36	1.93E-34
KCNN4	1.41	2.65	5.65E-36	2.09E-34
PRDM1	1.14	2.21	6.27E-36	2.31E-34
CAD	-1.02	0.49	6.34E-36	2.33E-34
DKC1	-0.94	0.52	6.39E-36	2.35E-34
PARP14	1.17	2.26	7.67E-36	2.81E-34
WDR1	0.72	1.64	1.07E-35	3.93E-34
HNRNPA1	-0.62	0.65	1.09E-35	3.97E-34
ARAP1	0.87	1.83	1.16E-35	4.21E-34
MELK	-1.04	0.49	1.39E-35	5.07E-34
ALS2CL	0.88	1.85	2.05E-35	7.43E-34
THRAP3	-0.72	0.61	2.15E-35	7.80E-34
TUBA1C	-0.85	0.55	2.24E-35	8.09E-34
TCF19	-1.03	0.49	2.30E-35	8.30E-34
IKBIP	1.29	2.44	2.50E-35	8.99E-34
PCYT2	-1.13	0.46	2.63E-35	9.43E-34
G2E3	-1.62	0.33	3.09E-35	1.11E-33
CIC	0.86	1.81	4.44E-35	1.59E-33
ATPIF1	0.81	1.75	4.50E-35	1.61E-33
TTYH3	0.92	1.89	4.94E-35	1.76E-33
H1FX	-1.23	0.43	5.32E-35	1.89E-33
DHCR7	-0.94	0.52	5.48E-35	1.94E-33
DDIT4	-0.78	0.58	6.09E-35	2.16E-33
ELMSAN1	-0.95	0.52	8.44E-35	2.98E-33
HMGA2	-0.74	0.60	9.06E-35	3.20E-33
FAM64A	-2.24	0.21	9.29E-35	3.27E-33
TUBB4B	-0.78	0.58	1.03E-34	3.61E-33
SLC35E4	1.44	2.72	1.22E-34	4.26E-33
MT1X	-1.36	0.39	1.41E-34	4.94E-33
PNRC1	1.57	2.96	1.60E-34	5.58E-33
GABRQ	3.45	10.96	1.63E-34	5.67E-33
TBL1X	-1.39	0.38	1.92E-34	6.67E-33
BNC1	-0.67	0.63	2.17E-34	7.55E-33
ATAD2	-0.92	0.53	2.23E-34	7.74E-33
AFAP1L1	-1.12	0.46	2.37E-34	8.19E-33
MAD2L1	-1.28	0.41	2.75E-34	9.49E-33
EVPL	-0.70	0.62	3.27E-34	1.13E-32
EPRS	-0.77	0.59	3.78E-34	1.30E-32
KCNK6	0.82	1.77	4.59E-34	1.58E-32
UTP20	-1.04	0.49	4.98E-34	1.71E-32
DFNA5	0.86	1.82	6.38E-34	2.18E-32
TMEM40	0.77	1.71	7.15E-34	2.44E-32
PLOD2	1.36	2.57	1.00E-33	3.41E-32
PHGDH	-0.98	0.51	1.14E-33	3.88E-32
ANXA4	0.81	1.75	1.25E-33	4.24E-32
KANK3	4.04	16.47	1.26E-33	4.26E-32
TMEM109	-0.98	0.51	1.34E-33	4.52E-32
RNF144B	0.94	1.92	1.39E-33	4.67E-32
TMEM30A	0.90	1.87	1.44E-33	4.84E-32
MAZ	-1.40	0.38	1.51E-33	5.07E-32
LRP8	-1.31	0.40	1.53E-33	5.13E-32
GCLC	-0.91	0.53	1.57E-33	5.26E-32
ATP6V1A	0.93	1.91	1.85E-33	6.17E-32
UBTD1	1.22	2.34	2.36E-33	7.87E-32
PCK2	-1.08	0.47	2.43E-33	8.09E-32
LINC00707	0.72	1.65	2.55E-33	8.47E-32
MAPRE3	1.32	2.49	2.88E-33	9.56E-32
TINAGL1	0.88	1.84	3.69E-33	1.22E-31
NEK2	-1.59	0.33	3.98E-33	1.31E-31
RPS23P8	-0.23	0.85	4.79E-03	NA
CDT1	-0.20	0.87	4.87E-03	1.15E-01
FZD8	-0.24	0.84	4.89E-03	1.16E-01
RP11-1002K11.1	-0.24	0.84	4.96E-03	1.17E-01
NCKAP5L	0.23	1.17	4.96E-03	1.17E-01
HELLS	-0.22	0.86	4.97E-03	1.17E-01
SMTN	0.14	1.10	5.03E-03	1.18E-01
ZC3HAV1L	-0.24	0.85	5.04E-03	1.18E-01
OAS3	-0.15	0.90	5.09E-03	1.19E-01
BIRC5	-0.20	0.87	5.16E-03	1.20E-01
PITPNM1	0.17	1.13	5.17E-03	1.20E-01
ARHGAP11A	-0.16	0.90	5.22E-03	1.21E-01
ALDH1A3	-0.14	0.91	5.27E-03	1.22E-01
SEC23A	0.15	1.11	5.27E-03	1.22E-01
SUOX	0.23	1.18	5.33E-03	1.23E-01
UTP20	-0.17	0.89	5.35E-03	1.23E-01
ACAA2	-0.24	0.85	5.35E-03	1.23E-01
SLC38A1	-0.14	0.91	5.36E-03	1.23E-01
ISCU	0.17	1.13	5.38E-03	1.23E-01
TFDP1	-0.13	0.92	5.38E-03	1.23E-01
ZNF180	-0.24	0.85	5.46E-03	1.24E-01
NPDC1	0.24	1.18	5.49E-03	1.24E-01
RPL12P47	-0.23	0.85	5.52E-03	NA
HR	-0.18	0.88	5.56E-03	1.26E-01
EIF4EBP2	-0.15	0.90	5.60E-03	1.26E-01
RP11-832N8.1	-0.24	0.85	5.63E-03	1.27E-01
ANKRD37	-0.21	0.86	5.63E-03	NA
APOBEC3A	-0.22	0.86	5.67E-03	NA
EEF1A1P10	-0.23	0.85	5.68E-03	NA
KDM7A	0.23	1.18	5.70E-03	1.28E-01
TMC4	0.24	1.18	5.73E-03	1.28E-01
TOMM5	-0.23	0.85	5.75E-03	NA
TUBG2	0.22	1.16	5.77E-03	1.29E-01
FAM83A	-0.12	0.92	5.80E-03	1.30E-01
MYEOV	-0.22	0.86	5.81E-03	NA
SDPR	0.22	1.17	5.96E-03	1.33E-01
PCDH7	-0.19	0.87	5.99E-03	1.33E-01
TGM2	-0.19	0.87	6.08E-03	NA
NCAPH	-0.17	0.89	6.11E-03	1.35E-01
SLC2A3	-0.20	0.87	6.14E-03	1.36E-01
FOXM1	-0.15	0.90	6.15E-03	1.36E-01
PPP1R3C	-0.23	0.85	6.27E-03	1.38E-01
AMIGO2	-0.20	0.87	6.28E-03	1.38E-01
KHSRP	-0.12	0.92	6.31E-03	1.38E-01
CDH3	0.12	1.09	6.32E-03	1.38E-01
MIR34AHG	0.19	1.14	6.37E-03	NA
TPI1	-0.11	0.93	6.44E-03	1.40E-01
EEF1A1	0.10	1.07	6.45E-03	1.40E-01
IKBKAP	-0.14	0.91	6.46E-03	1.40E-01
MASTL	-0.19	0.88	6.46E-03	1.40E-01
MFSD12	-0.20	0.87	6.59E-03	1.43E-01
TRAK1	0.15	1.11	6.65E-03	1.44E-01
NLRX1	-0.17	0.89	6.69E-03	1.44E-01
KLF16	-0.19	0.87	6.70E-03	1.44E-01
ATAT1	0.23	1.18	6.73E-03	1.44E-01
KRT7	-0.15	0.90	6.73E-03	1.44E-01
SRP9	0.18	1.13	6.76E-03	1.45E-01
RRS1	-0.17	0.89	6.78E-03	1.45E-01
SRSF5	0.16	1.12	6.79E-03	1.45E-01
IMPA2	-0.18	0.88	6.86E-03	1.46E-01
RP11-864N7.2	-0.23	0.85	6.87E-03	1.46E-01
SPEN	-0.16	0.89	6.89E-03	1.46E-01
UBE2H	0.14	1.10	6.91E-03	1.46E-01
SMC2	-0.17	0.89	6.95E-03	1.46E-01
CALCOCO1	0.19	1.14	6.97E-03	1.47E-01
SOX15	-0.14	0.91	7.14E-03	1.50E-01
KRT34	-0.18	0.89	7.20E-03	NA
RGS14	0.20	1.15	7.21E-03	1.51E-01
NECAP1	0.19	1.14	7.28E-03	1.52E-01
CEP55	-0.15	0.90	7.28E-03	1.52E-01
MTND6P4	0.23	1.17	7.30E-03	1.52E-01
CCDC28A	0.23	1.17	7.37E-03	1.53E-01
FAM214B	0.16	1.12	7.43E-03	1.54E-01
SETBP1	-0.23	0.85	7.45E-03	1.54E-01
NOTCH1	0.15	1.11	7.48E-03	1.54E-01
TCAF2	-0.20	0.87	7.50E-03	NA
C2orf54	-0.19	0.88	7.51E-03	1.55E-01
TTYH3	0.15	1.11	7.63E-03	1.57E-01
SH3GL1	-0.14	0.91	7.68E-03	1.58E-01
SPC25	-0.23	0.85	7.72E-03	1.58E-01
GDI1	0.14	1.10	7.76E-03	1.59E-01
UNC119	0.19	1.14	7.80E-03	1.59E-01
CCNO	0.23	1.17	7.80E-03	NA
ADAMTS1	-0.18	0.88	7.81E-03	1.59E-01
SBSN	-0.11	0.92	7.85E-03	1.59E-01
CDK4	-0.17	0.89	7.86E-03	1.59E-01
ARTN	-0.21	0.86	7.91E-03	1.60E-01
EIF5A	-0.12	0.92	7.94E-03	1.60E-01
PHLDA1	0.13	1.10	7.94E-03	1.60E-01
CASP9	0.23	1.17	7.97E-03	1.60E-01

**PTPRU**	0.91	1.88	3.99E-33	1.31E-31
**ARRDC4**	1.00	2.00	4.35E-33	1.43E-31
**IL1A**	0.96	1.94	4.64E-33	1.52E-31
**CPEB4**	1.06	2.09	5.28E-33	1.73E-31
**TCOF1**	-0.86	0.55	5.60E-33	1.83E-31
**PLIN4**	2.44	5.44	6.36E-33	2.08E-31
**WNT7A**	1.11	2.16	7.75E-33	2.53E-31
**NPM3**	-1.10	0.47	7.76E-33	2.53E-31
**SLFN5**	0.80	1.74	9.06E-33	2.94E-31
**CDK1**	-1.01	0.50	1.31E-32	4.26E-31
**PTP4A1**	0.72	1.64	1.54E-32	4.97E-31
**DUSP14**	0.87	1.83	1.63E-32	5.26E-31
**SHCBP1**	-1.22	0.43	1.84E-32	5.94E-31
**UQCC1**	0.93	1.91	1.92E-32	6.17E-31
**ANTXR1**	0.87	1.83	2.32E-32	7.45E-31
**NUF2**	-1.51	0.35	2.47E-32	7.93E-31
**PDLIM1**	0.68	1.61	3.06E-32	9.78E-31
**CHD4**	-0.68	0.63	3.18E-32	1.02E-30
**SIPA1**	-1.65	0.32	3.32E-32	1.06E-30
**GBP6**	-1.01	0.50	3.36E-32	1.07E-30
**CNFN**	-2.28	0.21	3.51E-32	1.11E-30
**KIF18A**	-1.74	0.30	4.15E-32	1.32E-30
**HERPUD1**	-1.27	0.42	4.16E-32	1.32E-30
**CCND1**	0.74	1.67	5.13E-32	1.62E-30
**CST3**	0.78	1.72	5.30E-32	1.67E-30
**RAD21**	-0.78	0.58	5.52E-32	1.74E-30
**GJA1**	-0.74	0.60	6.14E-32	1.93E-30
**E2F8**	-1.35	0.39	6.17E-32	1.94E-30
**CLIC1**	-0.68	0.62	6.28E-32	1.97E-30
**ACSS2**	-1.06	0.48	6.96E-32	2.18E-30
**SLC7A5**	-1.70	0.31	8.71E-32	2.72E-30
**RBM14**	-0.98	0.51	9.12E-32	2.84E-30
**TMEM246**	-1.18	0.44	9.48E-32	2.95E-30
**CYP2W1**	-2.81	0.14	1.08E-31	3.37E-30
**GART**	-0.77	0.59	1.40E-31	4.35E-30
**ISCU**	0.91	1.88	1.51E-31	4.68E-30
**UPK2**	3.15	8.88	1.53E-31	4.72E-30
**WEE1**	-1.35	0.39	1.56E-31	4.81E-30
**CABYR**	2.04	4.11	1.62E-31	4.98E-30
**FAM214A**	1.63	3.09	1.65E-31	5.08E-30
**TNKS1BP1**	-0.73	0.60	1.73E-31	5.32E-30
**PFAS**	-1.22	0.43	1.80E-31	5.53E-30
**EPAS1**	0.60	1.52	1.93E-31	5.91E-30
**SHROOM2**	1.03	2.04	2.22E-31	6.79E-30
**SHF**	-1.77	0.29	2.27E-31	6.91E-30
**MAPKBP1**	0.82	1.76	2.79E-31	8.49E-30
**GPRC5A**	0.93	1.90	3.02E-31	9.18E-30
**PCDH7**	-1.14	0.45	3.19E-31	9.68E-30
**STAT3**	0.71	1.64	3.22E-31	9.73E-30
**DPYSL2**	-1.41	0.38	4.27E-31	1.29E-29
**INPP1**	1.22	2.32	4.41E-31	1.33E-29
**CHEK1**	-1.13	0.46	4.41E-31	1.33E-29
**FOSL2**	0.71	1.63	4.49E-31	1.35E-29
**RPS6KA2**	-1.21	0.43	4.79E-31	1.44E-29
**LAMB1**	-0.67	0.63	5.48E-31	1.64E-29
**MYC**	-0.83	0.56	5.95E-31	1.78E-29
**LCE1B**	3.84	14.33	6.24E-31	1.86E-29
**YPEL5**	1.00	2.00	6.56E-31	1.95E-29
**CKAP2**	-0.88	0.54	6.97E-31	2.07E-29
**PLEKHO2**	1.22	2.33	7.42E-31	2.20E-29
**KRTDAP**	-0.79	0.58	7.52E-31	2.23E-29
**ARHGEF1**	-0.79	0.58	8.22E-31	2.43E-29
**CDCA5**	-1.11	0.46	8.24E-31	2.43E-29
**KDSR**	0.82	1.77	9.31E-31	2.75E-29
**PLK4**	-1.19	0.44	9.40E-31	2.77E-29
**NCKAP5L**	1.27	2.41	1.14E-30	3.36E-29
**JDP2**	-2.42	0.19	1.16E-30	3.40E-29
**NAV1**	-0.69	0.62	1.27E-30	3.73E-29
**EMP3**	-1.08	0.47	1.30E-30	3.79E-29
**TRIP13**	-1.00	0.50	1.35E-30	3.95E-29
**RASSF5**	1.14	2.20	1.53E-30	4.47E-29
**ZNF367**	-1.99	0.25	1.61E-30	4.68E-29
**SPRR1A**	-0.84	0.56	1.97E-30	5.74E-29
**BLVRB**	0.85	1.80	2.41E-30	6.99E-29
**TNFAIP8L1**	-1.49	0.35	2.66E-30	7.70E-29
**ARHGEF3**	0.82	1.76	2.73E-30	7.90E-29
**AC092171.4**	1.04	2.06	2.88E-30	8.30E-29
**ABCC2**	2.59	6.02	5.59E-30	1.61E-28
**IRF1**	1.77	3.40	6.21E-30	1.79E-28
**SERTAD1**	1.18	2.27	6.90E-30	1.98E-28
**UBTF**	-0.76	0.59	6.93E-30	1.99E-28
**SPRR3**	-0.81	0.57	7.01E-30	2.01E-28
**MARS**	-0.85	0.55	7.10E-30	2.03E-28
**NOLC1**	-0.69	0.62	7.63E-30	2.18E-28
**TMEM132A**	0.73	1.66	8.72E-30	2.49E-28
**SMPD1**	1.21	2.32	1.06E-29	3.01E-28
**BDH1**	-1.03	0.49	1.12E-29	3.20E-28
**SLCO4A1**	-1.17	0.44	1.28E-29	3.62E-28
**PEX6**	1.09	2.12	1.28E-29	3.63E-28
**FARSB**	-0.68	0.62	1.52E-29	4.31E-28

**FAM207A**	-0.21	0.86	7.97E-03	1.60E-01
**OAS2**	-0.15	0.90	7.98E-03	1.60E-01
**RP11-383G10.3**	-0.17	0.89	8.08E-03	NA
**VANGL1**	-0.14	0.91	8.09E-03	1.62E-01
**CA2**	-0.16	0.90	8.13E-03	1.62E-01
**WDR34**	-0.16	0.89	8.13E-03	1.62E-01
**CHAF1B**	-0.18	0.88	8.17E-03	1.63E-01
**RPL6**	0.11	1.08	8.19E-03	1.63E-01
**ITGA2**	0.14	1.11	8.22E-03	1.63E-01
**BUB3**	-0.13	0.92	8.31E-03	1.64E-01
**42799**	0.19	1.14	8.33E-03	1.64E-01
**NRK**	-0.22	0.86	8.35E-03	NA
**RPN1**	-0.11	0.92	8.35E-03	1.64E-01
**TRA2A**	-0.18	0.88	8.41E-03	1.65E-01
**FZR1**	-0.16	0.90	8.50E-03	1.67E-01
**ARAP1**	0.15	1.11	8.51E-03	1.67E-01
**ELK3**	0.13	1.10	8.53E-03	1.67E-01
**LAD1**	-0.11	0.93	8.54E-03	1.67E-01
**RAPGEF3**	0.20	1.15	8.57E-03	1.67E-01
**PRKD2**	0.16	1.11	8.58E-03	1.67E-01
**C6orf106**	0.13	1.09	8.67E-03	1.68E-01
**INO80D**	-0.22	0.86	8.71E-03	1.68E-01
**NCAPH2**	-0.18	0.89	8.71E-03	1.68E-01
**SAMD1**	-0.18	0.88	8.74E-03	1.68E-01
**FRMD8**	-0.16	0.90	8.78E-03	1.69E-01
**INCENP**	-0.14	0.91	8.92E-03	1.71E-01
**CYP26B1**	-0.20	0.87	8.95E-03	NA
**EIF3B**	-0.12	0.92	9.05E-03	1.73E-01
**RNF6**	0.16	1.11	9.06E-03	1.73E-01
**CTCF**	-0.15	0.90	9.21E-03	1.76E-01
**FAM8A1**	0.21	1.15	9.24E-03	1.76E-01
**YWHAH**	-0.13	0.91	9.26E-03	1.76E-01
**RGS12**	0.15	1.11	9.29E-03	1.76E-01
**CDC7**	-0.20	0.87	9.40E-03	1.78E-01
**CSRP2**	-0.22	0.86	9.41E-03	1.78E-01
**RASSF7**	-0.18	0.88	9.49E-03	1.79E-01
**SLC16A14**	0.22	1.17	9.54E-03	NA
**UBR1**	0.17	1.12	9.58E-03	1.81E-01
**TARS**	-0.12	0.92	9.63E-03	1.81E-01
**CALR**	-0.12	0.92	9.70E-03	1.82E-01
**NAA50**	-0.12	0.92	9.78E-03	1.83E-01
**CA11**	0.20	1.15	9.82E-03	NA
**KPNA2**	-0.11	0.93	9.99E-03	1.87E-01
**PLCH2**	0.14	1.10	9.99E-03	1.87E-01
**TRIM22**	0.19	1.14	1.00E-02	1.87E-01
**RRP7A**	-0.15	0.90	1.00E-02	1.87E-01
**TCTN2**	-0.22	0.86	1.01E-02	1.87E-01
**PTRF**	-0.11	0.93	1.01E-02	1.87E-01
**C9orf72**	0.22	1.17	1.01E-02	1.87E-01
**PLIN4**	0.20	1.15	1.02E-02	NA
**DCBLD2**	0.13	1.09	1.03E-02	1.90E-01
**ZNF503**	-0.19	0.88	1.03E-02	1.90E-01
**F11R**	0.15	1.11	1.03E-02	1.90E-01
**TAP1**	0.18	1.13	1.04E-02	1.91E-01
**ARRDC4**	0.17	1.12	1.05E-02	1.92E-01
**TENM3**	-0.19	0.88	1.05E-02	1.92E-01
**PLEKHG5**	0.13	1.10	1.05E-02	1.92E-01
**CHST6**	0.21	1.15	1.07E-02	NA
**CLSTN3**	0.21	1.16	1.07E-02	1.95E-01
**MIF4GD**	0.20	1.15	1.07E-02	1.95E-01
**CNFN**	-0.22	0.86	1.07E-02	1.95E-01
**LRRC8A**	0.11	1.08	1.08E-02	1.96E-01
**NUP62**	-0.15	0.90	1.08E-02	1.96E-01
**BRI3BP**	-0.16	0.89	1.09E-02	1.96E-01
**INPPL1**	0.17	1.13	1.09E-02	1.97E-01
**CASP4**	0.15	1.11	1.12E-02	2.01E-01
**PROCR**	0.17	1.13	1.12E-02	2.02E-01
**RPS6KL1**	0.21	1.16	1.12E-02	NA
**RBM14**	-0.15	0.90	1.13E-02	2.02E-01
**C19orf48**	-0.16	0.90	1.13E-02	2.03E-01
**OSBPL2**	0.17	1.12	1.13E-02	2.03E-01
**CYSTM1**	0.19	1.14	1.13E-02	2.03E-01
**C6orf1**	0.20	1.15	1.14E-02	2.03E-01
**TOP1**	0.13	1.09	1.14E-02	2.03E-01
**LIMA1**	0.10	1.07	1.14E-02	2.03E-01
**OSBP2**	0.14	1.10	1.15E-02	2.04E-01
**RUFY2**	0.20	1.15	1.15E-02	2.04E-01
**TNFAIP8L3**	-0.21	0.87	1.15E-02	NA
**PURB**	-0.17	0.89	1.17E-02	2.07E-01
**TP63**	-0.11	0.93	1.18E-02	2.09E-01
**TIMM13**	-0.14	0.91	1.18E-02	2.09E-01
**LAMA3**	0.11	1.08	1.19E-02	2.09E-01
**ATAD2**	-0.14	0.91	1.19E-02	2.09E-01
**ALKBH5**	-0.14	0.91	1.19E-02	2.09E-01
**RP11-298I3.4**	-0.10	0.93	1.21E-02	NA
**TOMM40**	-0.13	0.91	1.21E-02	2.12E-01
**SLC25A1**	-0.15	0.90	1.21E-02	2.12E-01
**PPCS**	0.17	1.13	1.22E-02	2.12E-01
**LY6D**	-0.12	0.92	1.22E-02	2.12E-01
**GPX8**	-0.19	0.87	1.22E-02	2.12E-01

SLC2A9	1.10	2.14	1.54E-29	4.35E-28	SIDT2	0.19	1.14	1.22E-02	2.12E-01
EMP1	-0.62	0.65	1.96E-29	5.51E-28	RABGGTB	0.16	1.11	1.22E-02	2.12E-01
CPT2	1.21	2.31	2.00E-29	5.64E-28	KIAA0430	0.15	1.11	1.22E-02	2.12E-01
PTMA	-0.69	0.62	2.06E-29	5.79E-28	SCARB1	-0.16	0.89	1.23E-02	2.12E-01
CAMKK1	-2.22	0.21	2.10E-29	5.90E-28	KRT6B	-0.09	0.94	1.24E-02	2.13E-01
SYNCRIP	-0.74	0.60	2.11E-29	5.92E-28	PACS1	0.14	1.10	1.24E-02	2.13E-01
CDCA7L	-1.16	0.45	2.16E-29	6.04E-28	GNPDA1	0.16	1.12	1.25E-02	2.14E-01
SLC19A1	-1.28	0.41	2.41E-29	6.72E-28	ITPRIPL2	-0.20	0.87	1.25E-02	2.15E-01
POLR2A	0.74	1.67	2.46E-29	6.85E-28	ALDH2	0.22	1.16	1.26E-02	2.15E-01
STAT1	0.72	1.64	2.51E-29	6.99E-28	SREBF1	0.14	1.10	1.26E-02	2.16E-01
SRC	0.83	1.77	2.85E-29	7.93E-28	EPHA2	0.14	1.10	1.27E-02	2.17E-01
CDCA4	-0.98	0.51	2.89E-29	8.03E-28	SHTN1	0.16	1.11	1.27E-02	2.17E-01
DBF4	-1.37	0.39	3.00E-29	8.31E-28	RP5-965G21.4	0.21	1.16	1.28E-02	NA
DSP	-0.67	0.63	3.06E-29	8.45E-28	RPS6KA2	0.17	1.13	1.28E-02	2.18E-01
YARS	-0.80	0.57	3.18E-29	8.78E-28	SEC31B	0.14	1.10	1.28E-02	NA
BLCAP	0.71	1.64	3.40E-29	9.39E-28	CDC14A	0.19	1.14	1.28E-02	NA
TCHP	-1.00	0.50	3.96E-29	1.09E-27	SESN1	0.21	1.15	1.29E-02	2.18E-01
PADI3	2.04	4.12	3.96E-29	1.09E-27	IQGAP3	-0.16	0.90	1.29E-02	2.18E-01
HSPG2	0.79	1.73	4.13E-29	1.13E-27	FAM84B	0.13	1.10	1.29E-02	2.18E-01
BRCA1	-0.99	0.50	5.17E-29	1.42E-27	EDA2R	0.20	1.15	1.30E-02	NA
TSPAN3	0.78	1.72	5.82E-29	1.59E-27	GMNN	-0.16	0.90	1.31E-02	2.21E-01
MCM10	-1.04	0.49	7.15E-29	1.95E-27	SLC25A29	0.22	1.16	1.31E-02	2.21E-01
ADH5	0.72	1.65	7.34E-29	2.00E-27	ERF	-0.14	0.91	1.31E-02	2.21E-01
DKK1	-0.90	0.54	7.61E-29	2.07E-27	NPM1P43	-0.18	0.88	1.32E-02	NA
MTSS1	-0.86	0.55	7.95E-29	2.16E-27	MGLL	-0.15	0.90	1.33E-02	2.23E-01
F2R	1.19	2.28	8.66E-29	2.35E-27	TAGLN	-0.21	0.86	1.34E-02	2.24E-01
MIS18BP1	-1.14	0.45	9.03E-29	2.45E-27	TRIAP1	0.19	1.14	1.34E-02	2.24E-01
NLN	-0.93	0.52	9.45E-29	2.56E-27	SNX2	0.16	1.12	1.34E-02	2.24E-01
FAM188A	1.07	2.10	1.12E-28	3.03E-27	SYT8	-0.19	0.88	1.35E-02	2.24E-01
PBK	-1.08	0.47	1.12E-28	3.03E-27	DUSP5	0.14	1.10	1.35E-02	2.24E-01
SLC9A1	0.96	1.95	1.13E-28	3.05E-27	RBM19	-0.17	0.89	1.35E-02	2.24E-01
TRIAP1	1.09	2.13	1.41E-28	3.79E-27	UPK1B	-0.13	0.91	1.36E-02	2.26E-01
SALL2	2.66	6.32	1.59E-28	4.27E-27	PSAT1	0.13	1.09	1.36E-02	2.26E-01
LSS	-0.81	0.57	1.59E-28	4.27E-27	RDH13	-0.19	0.88	1.36E-02	2.26E-01
LINC01503	1.31	2.48	1.64E-28	4.39E-27	NFIL3	-0.21	0.86	1.38E-02	2.28E-01
CSTB	-0.63	0.64	1.90E-28	5.07E-27	AUTS2	-0.17	0.89	1.38E-02	2.28E-01
DISP2	1.70	3.24	2.06E-28	5.51E-27	CEP78	-0.18	0.88	1.38E-02	2.28E-01
MCMBP	-0.76	0.59	2.15E-28	5.72E-27	EIF2AK2	-0.13	0.91	1.38E-02	2.28E-01
ABHD15	1.38	2.59	2.16E-28	5.75E-27	SDF2L1	-0.20	0.87	1.38E-02	2.28E-01
P4HA2	0.94	1.92	2.29E-28	6.10E-27	MR1	0.18	1.13	1.39E-02	2.28E-01
BEX4	1.35	2.55	2.52E-28	6.69E-27	TRIM25	-0.13	0.92	1.39E-02	2.28E-01
FRMD4A	1.23	2.34	2.68E-28	7.10E-27	PHLDB3	0.17	1.13	1.40E-02	2.28E-01
NAPRT	-1.16	0.45	2.89E-28	7.66E-27	SUGP2	0.16	1.11	1.40E-02	2.28E-01
SESN1	2.22	4.67	3.41E-28	9.00E-27	ERH	-0.13	0.91	1.41E-02	2.29E-01
SNN	0.83	1.77	3.53E-28	9.30E-27	FADS1	-0.18	0.88	1.41E-02	2.30E-01
CDC25C	-2.25	0.21	3.53E-28	9.30E-27	SNAI2	-0.13	0.91	1.41E-02	2.30E-01
ASB13	-1.36	0.39	3.58E-28	9.41E-27	TLN2	-0.20	0.87	1.42E-02	2.30E-01
FANCE	-1.37	0.39	3.94E-28	1.03E-26	GPX3	0.12	1.09	1.42E-02	2.30E-01
YPEL3	1.55	2.94	3.94E-28	1.03E-26	RASA1	0.11	1.08	1.42E-02	2.30E-01
TREM2	3.82	14.08	3.99E-28	1.04E-26	SMC1A	-0.11	0.93	1.43E-02	2.32E-01
HNRNPA2B1	-0.58	0.67	4.04E-28	1.06E-26	ACOT11	-0.20	0.87	1.44E-02	2.32E-01
MIR34AHG	2.57	5.92	4.44E-28	1.16E-26	JMY	0.21	1.16	1.45E-02	2.32E-01
HAP1	1.88	3.69	4.45E-28	1.16E-26	MAGOH	-0.17	0.89	1.45E-02	2.32E-01
FAT2	-0.88	0.54	4.50E-28	1.17E-26	KNTC1	-0.16	0.89	1.45E-02	2.32E-01
RAI1	-0.82	0.57	5.39E-28	1.40E-26	PLOD1	-0.13	0.91	1.45E-02	2.32E-01
ECM1	0.72	1.65	5.45E-28	1.41E-26	LINC00707	0.12	1.09	1.45E-02	2.32E-01
CYB5R2	-0.83	0.56	5.49E-28	1.42E-26	CHMP5	0.16	1.12	1.45E-02	2.32E-01
ZNF79	1.65	3.14	5.58E-28	1.44E-26	KIAA1586	0.21	1.16	1.46E-02	2.33E-01
TMPO	-0.80	0.57	5.74E-28	1.48E-26	CDCA2	-0.16	0.89	1.47E-02	2.34E-01
PRSS23	0.95	1.94	6.09E-28	1.57E-26	THOC3	-0.20	0.87	1.47E-02	2.34E-01
PTK7	0.76	1.69	6.96E-28	1.79E-26	CRAT	0.20	1.15	1.47E-02	NA
SGPP2	-2.14	0.23	7.12E-28	1.83E-26	ACSS2	-0.17	0.89	1.47E-02	2.34E-01
AZIN1	-0.69	0.62	8.65E-28	2.22E-26	JAG2	-0.16	0.90	1.48E-02	2.34E-01
PSTPIP2	0.89	1.86	9.14E-28	2.35E-26	ERCC5	0.20	1.15	1.48E-02	2.34E-01
PGM2	0.81	1.76	9.36E-28	2.40E-26	IFIT3	-0.21	0.86	1.48E-02	2.34E-01
PGLYRP3	-1.70	0.31	1.01E-27	2.59E-26	LY6K	-0.21	0.86	1.48E-02	2.34E-01
GGT1	2.16	4.46	1.02E-27	2.61E-26	SLC7A5	-0.19	0.88	1.50E-02	2.36E-01
EPPK1	0.99	1.99	1.04E-27	2.65E-26	EGLN1	-0.16	0.89	1.50E-02	2.36E-01
EPS8L1	-0.88	0.54	1.22E-27	3.10E-26	RUSC2	0.20	1.15	1.51E-02	2.37E-01
CYR61	0.90	1.87	1.37E-27	3.50E-26	EZH2	-0.17	0.89	1.51E-02	2.37E-01
SHMT1	-0.91	0.53	1.43E-27	3.62E-26	GOLGA7	0.16	1.12	1.53E-02	2.38E-01
ZNF496	-1.14	0.45	1.50E-27	3.81E-26	DGKQ	0.18	1.14	1.53E-02	2.38E-01
IGFBP3	1.99	3.98	1.72E-27	4.36E-26	GRN	0.13	1.09	1.53E-02	2.38E-01
ODF2	-0.82	0.57	1.80E-27	4.55E-26	DTX2	0.18	1.13	1.53E-02	2.38E-01
MVK	-0.92	0.53	1.86E-27	4.71E-26	KLHL21	-0.15	0.90	1.53E-02	2.38E-01
DHRS7	0.94	1.92	2.19E-27	5.53E-26	SBF2	0.16	1.12	1.54E-02	2.39E-01
TLE4	1.17	2.26	2.24E-27	5.64E-26	STC2	-0.20	0.87	1.54E-02	2.39E-01
CDKN2B	1.24	2.36	2.26E-27	5.69E-26	CEP68	0.20	1.15	1.54E-02	2.39E-01
LPCAT1	-0.90	0.54	2.28E-27	5.73E-26	ARL4D	-0.16	0.89	1.54E-02	2.39E-01
ARNTL2	-0.88	0.54	2.49E-27	6.24E-26	ENTPD6	0.15	1.11	1.56E-02	2.40E-01
DEK	-0.93	0.53	2.69E-27	6.73E-26	SLC35F3	-0.21	0.86	1.56E-02	2.40E-01
B4GALT2	-0.73	0.60	2.74E-27	6.85E-26	ETV4	0.14	1.10	1.56E-02	2.40E-01
MZF1	1.62	3.08	2.78E-27	6.93E-26	CTDSPL	-0.12	0.92	1.57E-02	2.40E-01
ENC1	0.79	1.73	3.52E-27	8.78E-26	FUS	-0.12	0.92	1.57E-02	2.40E-01
NFIB	-1.25	0.42	3.57E-27	8.89E-26	MT-ND3	0.15	1.11	1.57E-02	2.40E-01
CAPN15	-0.95	0.52	3.77E-27	9.36E-26	FBXO11	-0.16	0.90	1.58E-02	2.40E-01
IGSF3	-0.68	0.63	3.81E-27	9.45E-26	SP1	-0.13	0.92	1.58E-02	2.40E-01
CD44	0.57	1.48	4.33E-27	1.07E-25	WDR27	0.20	1.15	1.58E-02	2.40E-01
MAP3K6	-0.84	0.56	4.73E-27	1.17E-25	GTF3C3	0.16	1.11	1.58E-02	2.40E-01
AEN	0.86	1.81	5.50E-27	1.36E-25	CAPRIN2	0.20	1.15	1.58E-02	2.41E-01

MSH2	-0.96	0.51	6.57E-27	1.62E-25
NOS1	-1.17	0.44	7.57E-27	1.87E-25
MTCL1	0.91	1.87	8.07E-27	1.99E-25
SLC7A1	-0.71	0.61	8.08E-27	1.99E-25
CMBL	1.65	3.13	8.46E-27	2.08E-25
CALCOCO1	0.96	1.95	9.60E-27	2.35E-25
HCFC1R1	1.38	2.60	9.68E-27	2.37E-25
HAGH	1.08	2.12	1.03E-26	2.51E-25
PCSK9	-0.94	0.52	1.07E-26	2.60E-25
GLS2	3.61	12.23	1.13E-26	2.76E-25
PLXNB1	0.69	1.61	1.13E-26	2.76E-25
BNIP3	0.90	1.87	1.18E-26	2.87E-25
NGFR	1.26	2.39	1.25E-26	3.04E-25
SCPEP1	1.07	2.10	1.66E-26	4.03E-25
ATP6V1E1	0.70	1.62	1.67E-26	4.05E-25
FLNA	0.67	1.60	1.83E-26	4.43E-25
ATAD3B	-0.96	0.51	1.95E-26	4.70E-25
SYDE1	-1.61	0.33	1.97E-26	4.75E-25
CAPN14	-2.54	0.17	2.01E-26	4.84E-25
FXYD3	0.67	1.59	2.12E-26	5.11E-25
GAS6-AS1	3.95	15.46	2.43E-26	5.83E-25
PTGES3	-0.64	0.64	2.77E-26	6.64E-25
CDK18	1.16	2.24	2.78E-26	6.66E-25
EXOC7	0.76	1.69	4.04E-26	9.67E-25
PKP3	-0.56	0.68	4.05E-26	9.68E-25
ISM1	1.64	3.12	4.77E-26	1.14E-24
SSBP3	-1.08	0.47	5.34E-26	1.27E-24
PLEKHF1	1.62	3.08	5.55E-26	1.32E-24
KREMEN1	-0.71	0.61	6.27E-26	1.49E-24
E2F7	0.96	1.95	6.69E-26	1.59E-24
SERPINE1	-0.66	0.63	6.98E-26	1.65E-24
FBXW7	1.11	2.15	7.12E-26	1.68E-24
CYP4F3	1.84	3.57	7.35E-26	1.74E-24
MIDN	-0.78	0.58	7.73E-26	1.82E-24
SERPINB5	0.66	1.58	8.23E-26	1.94E-24
ARL6IP1	-0.89	0.54	8.52E-26	2.00E-24
NEFL	1.69	3.22	8.89E-26	2.09E-24
GRHL3	0.75	1.68	9.13E-26	2.14E-24
RHOD	0.67	1.59	9.24E-26	2.17E-24
JAG1	0.71	1.63	1.06E-25	2.48E-24
BLM	-1.22	0.43	1.09E-25	2.55E-24
NDE1	-0.81	0.57	1.09E-25	2.55E-24
DUSP1	0.94	1.92	1.10E-25	2.56E-24
STX12	0.83	1.78	1.12E-25	2.60E-24
MTHFD1	-0.66	0.63	1.36E-25	3.15E-24
STOX2	2.50	5.65	1.45E-25	3.36E-24
SSX2IP	-1.18	0.44	1.45E-25	3.36E-24
PHPT1	0.86	1.81	1.57E-25	3.63E-24
EWSR1	-0.63	0.64	1.66E-25	3.84E-24
GDNF	3.55	11.70	1.73E-25	4.00E-24
ZFYVE1	1.15	2.21	1.81E-25	4.18E-24
ANKRD35	-1.75	0.30	1.85E-25	4.25E-24
UBE2T	-1.12	0.46	1.90E-25	4.36E-24
NACA	-0.57	0.67	1.98E-25	4.55E-24
SH3D21	1.06	2.08	1.99E-25	4.57E-24
AMD1	-0.70	0.62	2.31E-25	5.28E-24
DUSP4	0.72	1.65	2.32E-25	5.32E-24
MRC2	0.79	1.72	2.36E-25	5.39E-24
PCBP4	0.90	1.86	2.53E-25	5.78E-24
YBX3	0.59	1.50	2.73E-25	6.23E-24
KAZN	0.85	1.80	3.08E-25	7.02E-24
CARM1	-0.71	0.61	3.12E-25	7.10E-24
LRRFIP1	-0.61	0.66	3.16E-25	7.18E-24
SYNRG	0.88	1.85	3.27E-25	7.42E-24
TMEM45A	-1.20	0.44	3.38E-25	7.67E-24
METTL8	0.87	1.83	4.02E-25	9.09E-24
FERMT1	-0.59	0.67	4.14E-25	9.35E-24
ACSF2	0.93	1.90	4.29E-25	9.68E-24
RP11-44F14.8	2.91	7.50	4.38E-25	9.88E-24
RAD51	-1.19	0.44	4.43E-25	9.97E-24
MRTO4	-0.83	0.56	4.55E-25	1.02E-23
RAB3B	1.91	3.76	4.87E-25	1.09E-23
EFNB3	-3.25	0.11	6.12E-25	1.37E-23
ZSWIM8	0.81	1.76	6.60E-25	1.48E-23
GNL3	-0.78	0.58	7.14E-25	1.60E-23
CCT5	-0.57	0.67	7.52E-25	1.68E-23
ACTA2	1.95	3.85	7.59E-25	1.69E-23
TRIM7	-1.11	0.46	9.29E-25	2.07E-23
TRPV3	1.68	3.20	9.71E-25	2.16E-23
CENPI	-1.46	0.36	9.76E-25	2.17E-23
PLA2G4F	0.86	1.82	1.00E-24	2.23E-23
FAM213B	-1.06	0.48	1.05E-24	2.33E-23
SNHG17	-1.23	0.43	1.06E-24	2.35E-23
CEP164	0.81	1.76	1.12E-24	2.48E-23
TRIM26	0.77	1.71	1.13E-24	2.50E-23
ZNF385A	0.71	1.64	1.14E-24	2.50E-23
LAMP1	0.53	1.45	1.18E-24	2.60E-23
MRPS34	-0.78	0.58	1.28E-24	2.82E-23
PTGES	1.04	2.06	1.36E-24	2.99E-23
APLP2	0.51	1.42	1.38E-24	3.02E-23
EHD1	-0.14	0.91	1.59E-02	2.41E-01
INO80	-0.14	0.90	1.59E-02	2.41E-01
APLP2	0.09	1.07	1.59E-02	2.41E-01
CDC25A	-0.17	0.89	1.61E-02	2.44E-01
RAB13	0.15	1.11	1.61E-02	2.44E-01
SLC16A9	-0.19	0.87	1.62E-02	2.44E-01
B4GALT3	0.16	1.12	1.62E-02	2.44E-01
C19orf43	-0.14	0.91	1.63E-02	2.45E-01
CYBRD1	0.20	1.15	1.63E-02	2.45E-01
GPR87	0.17	1.12	1.63E-02	2.45E-01
LCN2	0.18	1.14	1.64E-02	2.45E-01
PSMB9	-0.18	0.88	1.64E-02	NA
ZFP36	0.13	1.09	1.65E-02	2.47E-01
SLC20A1	0.12	1.09	1.65E-02	2.47E-01
ULK1	0.19	1.14	1.66E-02	2.47E-01
SEMA3A	0.18	1.14	1.66E-02	2.47E-01
PYGB	0.09	1.07	1.67E-02	2.48E-01
CENPF	-0.15	0.90	1.67E-02	2.48E-01
HMGN2	-0.13	0.91	1.67E-02	2.48E-01
ZCCHC11	0.16	1.12	1.68E-02	2.48E-01
RYBP	-0.17	0.89	1.68E-02	2.48E-01
NHSL1	-0.16	0.89	1.68E-02	2.48E-01
SLC16A5	0.21	1.16	1.68E-02	2.48E-01
NFKBIE	0.21	1.15	1.69E-02	2.48E-01
BRIP1	-0.20	0.87	1.69E-02	2.48E-01
SLF2	-0.17	0.89	1.69E-02	2.48E-01
IL17RA	-0.16	0.90	1.70E-02	2.48E-01
PCNA	-0.11	0.92	1.70E-02	2.48E-01
ADCY6	0.17	1.13	1.70E-02	2.49E-01
TMEM8B	0.20	1.15	1.71E-02	NA
FAS	0.19	1.14	1.71E-02	2.49E-01
IL1RAP	-0.13	0.92	1.71E-02	2.49E-01
PGAM1	-0.13	0.92	1.73E-02	2.52E-01
NDC80	-0.17	0.89	1.73E-02	2.52E-01
UBASH3B	-0.16	0.90	1.74E-02	2.52E-01
FBRSL1	-0.16	0.89	1.76E-02	2.54E-01
NDUFA4L2	-0.20	0.87	1.76E-02	2.54E-01
EIF2AK4	0.12	1.09	1.76E-02	2.54E-01
RTN4RL1	-0.17	0.89	1.77E-02	2.56E-01
DYNC1LI1	0.15	1.11	1.78E-02	2.57E-01
USB1	0.13	1.10	1.79E-02	2.57E-01
MT-TP	-0.21	0.87	1.80E-02	2.58E-01
KMT2C	-0.18	0.89	1.80E-02	2.58E-01
TMEM135	0.20	1.15	1.80E-02	2.58E-01
PPFIA4	-0.14	0.91	1.81E-02	NA
MAGED2	0.14	1.10	1.81E-02	2.59E-01
OSMR	0.13	1.10	1.81E-02	2.59E-01
CCNB1	-0.11	0.93	1.82E-02	2.59E-01
TRIO	-0.15	0.90	1.82E-02	2.59E-01
DSP	-0.12	0.92	1.83E-02	2.60E-01
ABHD14B	0.17	1.13	1.84E-02	2.61E-01
SERPINB1	0.13	1.10	1.85E-02	2.62E-01
FOXO4	0.20	1.15	1.85E-02	2.62E-01
DSC2	-0.13	0.92	1.85E-02	2.62E-01
CRIM1	-0.12	0.92	1.85E-02	2.62E-01
ZNF740	0.17	1.13	1.86E-02	2.62E-01
TRA2B	-0.11	0.93	1.86E-02	2.62E-01
CKS2	-0.14	0.91	1.86E-02	2.62E-01
JPH2	-0.20	0.87	1.87E-02	2.62E-01
NCL	-0.09	0.94	1.87E-02	2.62E-01
NAV2	-0.15	0.90	1.87E-02	2.62E-01
NUP153	-0.12	0.92	1.87E-02	2.62E-01
CORO2A	0.13	1.10	1.88E-02	2.63E-01
TTC9	-0.15	0.90	1.89E-02	2.63E-01
MIR9-3HG	-0.14	0.90	1.90E-02	NA
FCGRT	0.19	1.14	1.90E-02	2.65E-01
PDCD4	0.16	1.11	1.90E-02	2.65E-01
AK7	-0.13	0.91	1.90E-02	NA
ARSA	0.17	1.13	1.90E-02	2.65E-01
XG	0.18	1.14	1.91E-02	2.65E-01
B3GLCT	-0.20	0.87	1.92E-02	2.65E-01
NFIX	-0.13	0.91	1.92E-02	2.65E-01
PHLPP1	-0.16	0.89	1.93E-02	2.66E-01
TNIP1	-0.11	0.93	1.93E-02	2.66E-01
KCNN4	0.19	1.14	1.93E-02	2.66E-01
RTN4R	-0.19	0.88	1.93E-02	2.66E-01
MYLIP	0.18	1.13	1.94E-02	NA
DHFR	-0.18	0.88	1.94E-02	2.66E-01
APPL2	0.17	1.12	1.94E-02	2.66E-01
SLC2A6	-0.20	0.87	1.95E-02	2.68E-01
CARD10	-0.13	0.91	1.96E-02	2.68E-01
SEC14L1	0.14	1.10	1.96E-02	2.68E-01
ARG2	0.20	1.15	1.97E-02	2.69E-01
ISG15	-0.20	0.87	1.98E-02	2.69E-01
BZW1P1	-0.13	0.91	1.98E-02	NA
SH3BP2	-0.17	0.89	1.99E-02	2.71E-01
KANK3	0.10	1.07	2.01E-02	NA
FTL	0.11	1.08	2.02E-02	2.74E-01
NCBP2	0.14	1.10	2.02E-02	2.74E-01
GSTM4	0.14	1.10	2.02E-02	NA

TBX3	0.92	1.89	1.44E-24	3.16E-23
PRKRIR	-0.95	0.52	1.55E-24	3.40E-23
EHD1	0.79	1.73	1.66E-24	3.62E-23
IFRD2	-0.80	0.58	1.70E-24	3.71E-23
CASP10	1.15	2.22	1.74E-24	3.79E-23
ATP6V1H	0.81	1.76	1.76E-24	3.83E-23
AXL	0.87	1.82	1.82E-24	3.95E-23
MARVELD2	0.84	1.79	1.94E-24	4.22E-23
KRT7	0.71	1.64	2.09E-24	4.54E-23
RAPGEFL1	-0.91	0.53	2.18E-24	4.72E-23
C22orf29	0.82	1.76	2.23E-24	4.82E-23
RCAN1	1.05	2.08	2.58E-24	5.58E-23
PRKAB2	0.96	1.94	2.71E-24	5.86E-23
REXO2	0.75	1.68	2.75E-24	5.94E-23
FBLN2	1.80	3.48	3.05E-24	6.57E-23
ILF3	-0.59	0.66	3.11E-24	6.70E-23
ZER1	0.83	1.78	3.24E-24	6.97E-23
CDC42EP3	0.83	1.78	3.66E-24	7.87E-23
CYP3A5	1.79	3.46	4.00E-24	8.57E-23
BTBD10	0.70	1.63	4.00E-24	8.57E-23
ACOX1	0.76	1.69	4.09E-24	8.75E-23
ARHGEF39	-1.94	0.26	4.98E-24	1.06E-22
EPHX1	1.24	2.36	5.46E-24	1.16E-22
TUBGCP3	-0.92	0.53	5.82E-24	1.24E-22
MYO5B	-0.69	0.62	6.26E-24	1.33E-22
NIN	-0.86	0.55	6.31E-24	1.34E-22
RP11-268J15.5	1.72	3.31	6.35E-24	1.35E-22
VSIG10L	-1.19	0.44	6.35E-24	1.35E-22
OSBP2	0.75	1.68	6.56E-24	1.39E-22
IQSEC1	-1.26	0.42	6.64E-24	1.40E-22
BAIAP2L2	-1.87	0.27	6.81E-24	1.44E-22
AK1	1.78	3.44	6.85E-24	1.45E-22
NRF1	-1.36	0.39	7.40E-24	1.56E-22
DHCR24	-0.66	0.63	7.44E-24	1.57E-22
GUK1	-0.59	0.67	7.73E-24	1.63E-22
ICMT	-0.64	0.64	7.97E-24	1.67E-22
TEAD4	-1.28	0.41	8.24E-24	1.73E-22
SNX1	0.64	1.56	8.33E-24	1.75E-22
S100P	-0.82	0.57	8.77E-24	1.84E-22
RP11-58O9.2	1.13	2.19	9.03E-24	1.89E-22
RXRA	-0.70	0.62	9.38E-24	1.96E-22
ZFHX3	-1.31	0.40	1.02E-23	2.14E-22
HSPB8	0.81	1.76	1.10E-23	2.30E-22
FBL	-0.68	0.63	1.16E-23	2.41E-22
PKP1	-0.51	0.70	1.24E-23	2.57E-22
LIF	2.85	7.21	1.24E-23	2.58E-22
HYLS1	-1.61	0.33	1.32E-23	2.74E-22
RP11-421F16.3	1.31	2.48	1.33E-23	2.76E-22
PLP2	-0.68	0.62	1.39E-23	2.88E-22
PRKRA	0.91	1.88	1.43E-23	2.96E-22
ATAD3A	-0.81	0.57	1.85E-23	3.82E-22
MAF	1.52	2.86	1.92E-23	3.96E-22
PEAR1	1.40	2.64	2.37E-23	4.89E-22
ZFP36L1	-0.60	0.66	2.40E-23	4.93E-22
LYPLA2	0.74	1.67	3.08E-23	6.33E-22
UPP1	-0.62	0.65	3.42E-23	7.03E-22
MFAP5	-0.75	0.59	3.58E-23	7.35E-22
PRSS12	0.82	1.77	3.61E-23	7.40E-22
P3H2	0.62	1.54	3.73E-23	7.63E-22
CBX3	-0.76	0.59	3.82E-23	7.80E-22
ISG15	1.12	2.17	4.06E-23	8.28E-22
FBXO44	1.07	2.10	4.14E-23	8.43E-22
SRSF3	-0.61	0.65	4.15E-23	8.45E-22
RNF123	0.97	1.96	4.29E-23	8.72E-22
SF3A2	-0.80	0.57	4.64E-23	9.43E-22
MLLT6	-0.72	0.61	6.18E-23	1.25E-21
STARD5	1.58	2.99	6.21E-23	1.26E-21
GLUL	-0.69	0.62	6.61E-23	1.34E-21
ACACA	-0.65	0.64	7.61E-23	1.54E-21
RAET1E	-1.01	0.50	8.51E-23	1.72E-21
DIAPH1	-0.55	0.68	9.12E-23	1.84E-21
PRKD2	0.71	1.64	9.16E-23	1.85E-21
EBP	-0.81	0.57	9.61E-23	1.93E-21
GCC2	1.21	2.31	1.03E-22	2.06E-21
RBM38	0.98	1.97	1.03E-22	2.08E-21
GPRIN1	-1.14	0.45	1.08E-22	2.16E-21
GOT2	0.56	1.48	1.11E-22	2.23E-21
TYMP	-0.80	0.57	1.21E-22	2.41E-21
CDC20P1	-2.07	0.24	1.22E-22	2.45E-21
ICAM1	2.18	4.54	1.23E-22	2.46E-21
MTMR4	-0.85	0.55	1.26E-22	2.51E-21
CALD1	0.57	1.48	1.33E-22	2.65E-21
YWHAH	-0.59	0.66	1.33E-22	2.65E-21
PDS5A	-0.59	0.67	1.44E-22	2.86E-21
PIP4K2C	0.63	1.55	1.53E-22	3.04E-21
KAT2B	1.15	2.22	1.54E-22	3.06E-21
NCF2	-1.22	0.43	1.55E-22	3.07E-21
WDR5	-0.72	0.61	1.72E-22	3.41E-21
SLC38A5	-0.88	0.54	1.73E-22	3.42E-21
HIGD1A	0.70	1.63	1.88E-22	3.71E-21
SRXN1	-0.10	0.93	2.02E-02	NA
HIBCH	0.20	1.15	2.03E-02	2.75E-01
CRKL	-0.12	0.92	2.03E-02	2.75E-01
MON2	0.16	1.12	2.03E-02	2.75E-01
TIMP1	0.15	1.11	2.04E-02	2.76E-01
ZRANB2	0.15	1.11	2.04E-02	2.76E-01
MFSD6	0.13	1.09	2.05E-02	2.76E-01
BMP6	-0.18	0.89	2.05E-02	2.76E-01
GRAMD2	0.20	1.15	2.06E-02	2.77E-01
CELSR1	-0.13	0.91	2.06E-02	2.77E-01
TMCC3	0.14	1.10	2.07E-02	2.77E-01
RPUSD1	-0.15	0.90	2.07E-02	2.77E-01
THRAP3	-0.10	0.93	2.07E-02	2.77E-01
MAPRE2	-0.16	0.89	2.07E-02	2.77E-01
ARL6IP1	-0.12	0.92	2.08E-02	2.77E-01
SLC22A18	0.20	1.15	2.08E-02	2.77E-01
NDC1	-0.13	0.91	2.08E-02	2.77E-01
ANKMY1	-0.20	0.87	2.09E-02	2.78E-01
PAIP2	0.16	1.12	2.10E-02	2.78E-01
JDP2	-0.20	0.87	2.10E-02	2.78E-01
CCNA2	-0.12	0.92	2.10E-02	2.78E-01
BBS12	0.19	1.14	2.11E-02	NA
DGKA	0.12	1.08	2.11E-02	2.79E-01
SLC4A11	0.17	1.12	2.12E-02	2.79E-01
DEPDC1	-0.16	0.90	2.12E-02	2.80E-01
CDK5RAP2	-0.13	0.91	2.13E-02	2.80E-01
G0S2	0.19	1.14	2.13E-02	2.80E-01
BRPF1	-0.17	0.89	2.14E-02	2.80E-01
RBMS1P1	-0.06	0.96	2.14E-02	NA
IFNGR2	0.13	1.09	2.15E-02	2.82E-01
PRSS22	0.19	1.14	2.16E-02	2.82E-01
SLC8A1	-0.15	0.90	2.17E-02	NA
SPRED3	0.19	1.14	2.17E-02	NA
SNRPN	0.19	1.14	2.17E-02	NA
PLAU	0.14	1.10	2.18E-02	2.85E-01
LINC00630	0.17	1.13	2.20E-02	NA
RAP1GAP2	0.13	1.10	2.22E-02	2.90E-01
SBDSP1	-0.18	0.88	2.23E-02	2.91E-01
ATPIF1	0.13	1.09	2.24E-02	2.92E-01
NUCKS1	-0.11	0.93	2.24E-02	2.92E-01
NOTCH3	-0.10	0.93	2.24E-02	2.92E-01
VOPP1	-0.20	0.87	2.28E-02	2.94E-01
MZT2A	-0.16	0.90	2.28E-02	2.94E-01
MEF2D	0.14	1.10	2.28E-02	2.94E-01
GNL3L	-0.19	0.88	2.28E-02	2.94E-01
FERMT1	0.10	1.07	2.28E-02	2.94E-01
MAPK7	-0.17	0.89	2.28E-02	2.94E-01
GPALPP1	0.19	1.14	2.30E-02	2.96E-01
LRP5	0.13	1.10	2.30E-02	2.96E-01
SLC2A4RG	-0.14	0.91	2.30E-02	2.96E-01
AL627309.1	0.16	1.12	2.30E-02	NA
B3GALNT1	-0.20	0.87	2.30E-02	2.96E-01
TOPORS	0.16	1.12	2.32E-02	2.97E-01
ATAD5	-0.19	0.88	2.33E-02	2.98E-01
ADTRP	0.19	1.14	2.34E-02	NA
CLIP2	0.19	1.14	2.34E-02	2.99E-01
VEGFA	-0.13	0.91	2.34E-02	2.99E-01
PSMA6	-0.20	0.87	2.35E-02	3.00E-01
CNOT6	0.13	1.10	2.36E-02	3.00E-01
TM2D1	0.19	1.14	2.37E-02	3.02E-01
PRC1	-0.11	0.93	2.39E-02	3.03E-01
ANKLE1	-0.20	0.87	2.39E-02	3.03E-01
FRG1JP	0.11	1.08	2.39E-02	NA
SLC2A5	-0.19	0.88	2.40E-02	NA
TBC1D4	-0.16	0.90	2.41E-02	3.06E-01
TUBB1	-0.07	0.96	2.41E-02	NA
HCFC1	-0.12	0.92	2.42E-02	3.07E-01
TMEM222	-0.18	0.88	2.43E-02	3.07E-01
ATP8B2	0.15	1.11	2.44E-02	3.07E-01
PFKP	-0.10	0.93	2.44E-02	3.07E-01
SRSF11	0.12	1.08	2.44E-02	3.07E-01
ERCC1	0.14	1.10	2.44E-02	3.08E-01
RP1-228H13.1	-0.17	0.89	2.45E-02	NA
CHML	-0.19	0.87	2.46E-02	3.09E-01
POLA1	-0.16	0.90	2.46E-02	3.09E-01
ANKRA2	0.19	1.14	2.47E-02	3.10E-01
ZNF790	0.19	1.14	2.48E-02	NA
LINC01094	-0.10	0.93	2.48E-02	NA
RNF222	-0.19	0.88	2.48E-02	NA
DMGDH	0.06	1.04	2.49E-02	NA
STEAP1	-0.18	0.88	2.49E-02	3.12E-01
RPS28	-0.12	0.92	2.49E-02	3.12E-01
SPAG4	0.11	1.08	2.49E-02	NA
SULF2	-0.11	0.93	2.50E-02	3.13E-01
S1PR5	-0.15	0.90	2.52E-02	3.14E-01
INAFM2	0.18	1.14	2.52E-02	NA
POLB	0.16	1.12	2.52E-02	3.14E-01
SMAGP	-0.15	0.90	2.52E-02	3.14E-01
KPNA5	0.19	1.14	2.52E-02	NA
AIM1L	-0.11	0.92	2.53E-02	3.14E-01

ALDH4A1	0.88	1.84	1.99E-22	3.92E-21
CHADL	2.78	6.86	2.01E-22	3.96E-21
MYBPHL	3.47	11.06	2.21E-22	4.34E-21
CEP192	-0.87	0.55	2.54E-22	5.00E-21
DDX58	1.07	2.09	2.67E-22	5.25E-21
SLC6A8	-0.60	0.66	2.74E-22	5.37E-21
BCLAF1	-0.61	0.66	2.77E-22	5.42E-21
PCNA	0.54	1.45	2.81E-22	5.50E-21
TYSND1	0.89	1.85	2.89E-22	5.65E-21
PPP1R14A	1.83	3.56	3.04E-22	5.94E-21
NCEH1	0.94	1.92	3.09E-22	6.03E-21
SNRPA	-0.78	0.58	3.11E-22	6.06E-21
APOBEC3G	1.82	3.54	3.15E-22	6.13E-21
FAM49A	2.09	4.25	3.20E-22	6.22E-21
POLR2H	0.78	1.72	3.22E-22	6.25E-21
TARS	-0.56	0.68	3.55E-22	6.90E-21
SLC52A1	2.60	6.05	3.56E-22	6.90E-21
ULBP1	1.70	3.26	3.81E-22	7.37E-21
ADA	1.02	2.03	3.85E-22	7.44E-21
STEAP3	0.81	1.75	4.37E-22	8.45E-21
MRPL3	-0.68	0.62	4.58E-22	8.84E-21
ID3	0.85	1.80	4.74E-22	9.13E-21
CHAF1B	-0.98	0.51	4.75E-22	9.15E-21
TACSTD2	-0.61	0.66	4.85E-22	9.33E-21
SKA3	-1.07	0.47	5.02E-22	9.64E-21
IGSF9	-0.87	0.55	5.13E-22	9.84E-21
HSP90AA1	-0.50	0.71	5.65E-22	1.08E-20
NTPCR	0.93	1.91	5.93E-22	1.14E-20
BARD1	-1.35	0.39	5.94E-22	1.14E-20
SH3PXD2B	-0.78	0.58	6.98E-22	1.33E-20
CENPB	-0.72	0.61	7.59E-22	1.45E-20
PTBP1	-0.69	0.62	7.74E-22	1.48E-20
BEND3	-1.40	0.38	7.91E-22	1.51E-20
PRNP	-0.47	0.72	7.97E-22	1.52E-20
CCDC90B	0.87	1.83	8.35E-22	1.59E-20
CKAP5	-0.55	0.68	8.76E-22	1.66E-20
TENM3	-1.12	0.46	8.89E-22	1.69E-20
TST	1.19	2.28	9.52E-22	1.80E-20
SUV39H1	-1.09	0.47	9.77E-22	1.85E-20
PNPO	0.83	1.78	9.84E-22	1.86E-20
TBC1D14	-0.74	0.60	9.87E-22	1.86E-20
ID2	1.20	2.30	9.95E-22	1.88E-20
EXOSC2	-0.96	0.51	1.05E-21	1.97E-20
CEP170B	0.65	1.57	1.08E-21	2.03E-20
ULK1	0.97	1.96	1.25E-21	2.35E-20
SEPHS1	-0.77	0.59	1.31E-21	2.47E-20
LYNX1	1.67	3.19	1.31E-21	2.47E-20
KIAA0513	1.06	2.09	1.38E-21	2.59E-20
BACE1	0.81	1.76	1.43E-21	2.67E-20
NUP98	-0.60	0.66	1.57E-21	2.94E-20
VEZF1	-1.03	0.49	1.70E-21	3.18E-20
RP11-44F14.2	3.22	9.35	1.79E-21	3.34E-20
DHX9	-0.55	0.68	1.81E-21	3.38E-20
HMGB3	-0.77	0.59	1.93E-21	3.60E-20
PRKCDBP	-1.02	0.49	1.98E-21	3.68E-20
LTA4H	0.60	1.51	2.00E-21	3.72E-20
ABLIM1	-0.64	0.64	2.21E-21	4.09E-20
ELOVL6	-0.74	0.60	2.23E-21	4.13E-20
REEP2	2.64	6.25	2.23E-21	4.13E-20
HEATR1	-0.67	0.63	2.23E-21	4.13E-20
MIR22HG	1.18	2.27	2.33E-21	4.32E-20
SMARCA4	-0.54	0.69	2.34E-21	4.32E-20
EPHB2	1.81	3.50	2.35E-21	4.33E-20
PDPN	-1.06	0.48	2.55E-21	4.71E-20
PTPRE	0.88	1.84	2.57E-21	4.73E-20
CYB5R1	0.67	1.59	2.70E-21	4.96E-20
RP11-12G12.7	1.55	2.93	2.71E-21	4.98E-20
LCAT	1.59	3.01	2.78E-21	5.11E-20
LY6D	0.73	1.66	2.87E-21	5.27E-20
TLCD1	1.32	2.50	3.01E-21	5.52E-20
RPS6KA4	-0.70	0.61	3.25E-21	5.94E-20
SEC31A	0.52	1.43	3.30E-21	6.02E-20
ESPL1	-1.64	0.32	3.36E-21	6.14E-20
PGBD5	1.00	2.00	3.45E-21	6.29E-20
RIPK4	0.76	1.70	3.74E-21	6.81E-20
ARHGEF16	-0.90	0.54	3.77E-21	6.85E-20
PTPN6	0.90	1.87	3.78E-21	6.87E-20
MAP4	-0.49	0.71	3.78E-21	6.87E-20
BLNK	1.61	3.06	4.25E-21	7.71E-20
NBEAL2	-0.54	0.69	4.56E-21	8.27E-20
HIPK2	-0.79	0.58	4.62E-21	8.36E-20
POLD1	-0.77	0.59	4.70E-21	8.50E-20
JAG2	-0.75	0.59	4.91E-21	8.87E-20
TKT	0.53	1.44	5.20E-21	9.40E-20
CDH3	0.53	1.45	5.25E-21	9.46E-20
LRRC4	-2.66	0.16	5.55E-21	9.99E-20
TTLL4	-0.80	0.58	6.18E-21	1.11E-19
DDX21	-0.56	0.68	6.28E-21	1.13E-19
HSD17B10	0.64	1.56	6.65E-21	1.19E-19
SLC35E3	1.04	2.05	6.78E-21	1.22E-19
MIR205HG	0.13	1.09	2.53E-02	3.14E-01
NAGLU	0.18	1.14	2.53E-02	3.14E-01
GABRE	0.18	1.13	2.55E-02	3.16E-01
SIX4	0.19	1.14	2.56E-02	3.16E-01
SH3YL1	0.15	1.11	2.56E-02	3.16E-01
PES1	-0.11	0.93	2.57E-02	3.16E-01
CAPG	0.10	1.07	2.57E-02	3.16E-01
ST20	0.14	1.10	2.58E-02	NA
HN1	-0.10	0.93	2.58E-02	3.17E-01
SHCBP1	-0.16	0.90	2.58E-02	3.17E-01
NUP188	-0.10	0.93	2.58E-02	3.17E-01
NSDHL	-0.15	0.90	2.60E-02	3.19E-01
MAGED1	0.13	1.10	2.60E-02	3.19E-01
JMJD8	-0.18	0.88	2.61E-02	3.19E-01
HIST1H2AE	-0.09	0.94	2.61E-02	NA
GRB7	0.17	1.12	2.62E-02	3.20E-01
EPGN	-0.15	0.90	2.62E-02	3.20E-01
ANXA8L1	-0.19	0.88	2.64E-02	3.22E-01
ROR1	-0.19	0.88	2.64E-02	NA
ITGA5	-0.12	0.92	2.65E-02	3.23E-01
CLN6	-0.15	0.90	2.66E-02	3.24E-01
ARID1B	-0.14	0.91	2.66E-02	3.24E-01
GOLGB1	0.14	1.10	2.67E-02	3.24E-01
GADD45A	0.13	1.10	2.67E-02	3.24E-01
LZTS2	0.13	1.10	2.70E-02	3.26E-01
CCDC57	0.17	1.13	2.70E-02	3.26E-01
RAC2	0.15	1.11	2.70E-02	3.26E-01
AGO4	0.19	1.14	2.71E-02	3.27E-01
EIF4A3	-0.12	0.92	2.71E-02	3.27E-01
YDJC	-0.16	0.90	2.72E-02	3.27E-01
ATP6V0E2	0.19	1.14	2.72E-02	3.27E-01
TSC22D3	-0.17	0.89	2.73E-02	3.27E-01
ZNF204P	0.19	1.14	2.74E-02	NA
PDE6D	0.17	1.13	2.76E-02	3.30E-01
SPDL1	-0.15	0.90	2.76E-02	3.30E-01
PKP2	-0.11	0.92	2.76E-02	3.30E-01
DNAJC9	-0.18	0.88	2.76E-02	3.30E-01
SKI	-0.13	0.91	2.77E-02	3.30E-01
NCLN	-0.14	0.91	2.77E-02	3.30E-01
EDN1	-0.15	0.90	2.78E-02	3.31E-01
PLD3	0.12	1.09	2.79E-02	3.32E-01
IL18	0.14	1.10	2.80E-02	3.32E-01
CENPU	-0.15	0.90	2.81E-02	3.32E-01
DTL	-0.15	0.90	2.81E-02	3.32E-01
DLX1	-0.19	0.88	2.81E-02	3.32E-01
RP5-1052M9.1	-0.19	0.88	2.83E-02	3.34E-01
C16orf87	0.19	1.14	2.84E-02	3.35E-01
RGP1	0.13	1.09	2.84E-02	3.35E-01
SKA3	-0.15	0.90	2.87E-02	3.38E-01
ZBED3	-0.19	0.88	2.88E-02	3.39E-01
KLF6	-0.10	0.93	2.88E-02	3.39E-01
ZER1	0.15	1.11	2.89E-02	3.40E-01
HIST1H4I	0.18	1.13	2.90E-02	NA
RP11-157J24.2	-0.06	0.96	2.90E-02	NA
RIMS3	0.18	1.13	2.90E-02	NA
BEX4	0.19	1.14	2.90E-02	3.40E-01
ARFGEF2	0.13	1.09	2.91E-02	3.40E-01
SERTAD4	-0.19	0.88	2.91E-02	3.40E-01
HACL1	0.18	1.13	2.91E-02	3.40E-01
STAG1	-0.16	0.90	2.91E-02	3.40E-01
CST6	-0.16	0.90	2.91E-02	NA
ARFGEF3	-0.19	0.88	2.92E-02	3.41E-01
RP11-244J10.1	-0.07	0.95	2.93E-02	NA
TYMP	-0.13	0.91	2.93E-02	3.42E-01
MAN2C1	0.15	1.11	2.94E-02	3.42E-01
C1RL	0.18	1.14	2.94E-02	3.42E-01
GNG7	0.07	1.05	2.95E-02	NA
OLFML2A	-0.19	0.88	2.97E-02	3.44E-01
BRCC3	-0.15	0.90	2.97E-02	3.44E-01
CTU1	-0.19	0.88	2.97E-02	3.44E-01
ITGB1BP1	-0.13	0.91	2.97E-02	3.44E-01
RP11-253E3.3	-0.19	0.88	2.98E-02	3.45E-01
MGEA5	0.11	1.08	3.00E-02	3.47E-01
OBFC1	0.15	1.11	3.01E-02	3.47E-01
ADGRF4	-0.14	0.90	3.02E-02	3.47E-01
PEX19	0.14	1.10	3.06E-02	3.52E-01
UTP3	0.14	1.11	3.07E-02	3.52E-01
BAHCC1	-0.18	0.88	3.07E-02	3.52E-01
RP11-30K9.5	-0.04	0.97	3.08E-02	NA
NEBL	-0.13	0.91	3.08E-02	3.52E-01
DAG1	-0.10	0.93	3.08E-02	3.52E-01
BLM	-0.16	0.89	3.08E-02	3.52E-01
C6orf48	0.13	1.10	3.08E-02	3.52E-01
NOS1	-0.16	0.90	3.08E-02	3.52E-01
CUL7	0.16	1.12	3.11E-02	3.55E-01
NKD1	-0.12	0.92	3.13E-02	NA
TP53TG1	0.19	1.14	3.13E-02	3.57E-01
TMEM45A	-0.16	0.89	3.14E-02	3.57E-01
YPEL3	0.19	1.14	3.15E-02	3.57E-01
IDS	0.13	1.10	3.15E-02	3.57E-01

**RRP1**	-0.83	0.56	6.80E-21	1.22E-19
**ZFYVE21**	-0.70	0.62	6.85E-21	1.23E-19
**SIRT2**	0.81	1.75	6.95E-21	1.24E-19
**EXOSC8**	-0.95	0.52	7.35E-21	1.31E-19
**MYRF**	2.72	6.57	7.68E-21	1.37E-19
**ZNF767P**	1.60	3.02	7.81E-21	1.39E-19
**SMC2**	-0.83	0.56	8.31E-21	1.48E-19
**SLC35G2**	2.43	5.38	8.90E-21	1.58E-19
**SPTBN2**	-0.57	0.67	9.52E-21	1.69E-19
**JADE1**	-1.60	0.33	9.56E-21	1.70E-19
**SOWAHB**	1.17	2.25	9.62E-21	1.71E-19
**ITFG1**	0.85	1.81	1.03E-20	1.83E-19
**ORC1**	-0.96	0.51	1.09E-20	1.93E-19
**A2ML1**	-0.51	0.70	1.14E-20	2.01E-19
**CTNNAL1**	-0.77	0.59	1.15E-20	2.03E-19
**DCLRE1A**	-1.22	0.43	1.16E-20	2.05E-19
**RBL1**	-1.04	0.49	1.21E-20	2.13E-19
**HHAT**	1.71	3.28	1.24E-20	2.19E-19
**RALGDS**	0.69	1.61	1.31E-20	2.31E-19
**AP1B1**	0.57	1.49	1.35E-20	2.37E-19
**NTN4**	0.89	1.86	1.54E-20	2.71E-19
**LRRC8B**	-1.21	0.43	1.57E-20	2.75E-19
**ANXA3**	-0.59	0.67	1.59E-20	2.80E-19
**RBMX**	-0.65	0.64	1.69E-20	2.96E-19
**SLC6A14**	-0.88	0.54	1.71E-20	2.99E-19
**PCDHGC3**	-0.97	0.51	1.73E-20	3.03E-19
**SPDL1**	-0.92	0.53	1.82E-20	3.18E-19
**RHBDF1**	0.80	1.74	1.91E-20	3.34E-19
**SLC1A4**	-1.86	0.27	2.00E-20	3.49E-19
**SYNC**	2.25	4.75	2.10E-20	3.66E-19
**CS**	-0.52	0.70	2.26E-20	3.93E-19
**DTYMK**	-0.79	0.58	2.31E-20	4.01E-19
**CHAC1**	-1.81	0.28	2.32E-20	4.02E-19
**PEMT**	-1.11	0.46	2.32E-20	4.02E-19
**MVD**	-0.80	0.57	2.47E-20	4.28E-19
**E2F1**	-1.36	0.39	2.56E-20	4.43E-19
**SREBF1**	-0.72	0.61	2.58E-20	4.47E-19
**LTBP2**	0.62	1.54	2.71E-20	4.68E-19
**RHOBTB2**	-0.92	0.53	2.76E-20	4.76E-19
**HES6**	2.22	4.67	2.82E-20	4.86E-19
**USP1**	-0.78	0.58	2.85E-20	4.91E-19
**PIP5K1A**	0.59	1.51	2.86E-20	4.92E-19
**RAN**	-0.51	0.70	3.02E-20	5.19E-19
**DUSP13**	3.48	11.16	3.20E-20	5.50E-19
**SHISA6**	-1.54	0.34	3.40E-20	5.83E-19
**OAS3**	0.58	1.50	3.51E-20	6.01E-19
**CRIP2**	-1.91	0.27	3.61E-20	6.19E-19
**SSR2**	-0.60	0.66	3.80E-20	6.50E-19
**USF1**	0.89	1.85	3.99E-20	6.81E-19
**PTCHD4**	2.70	6.48	4.14E-20	7.07E-19
**CNN3**	0.73	1.66	4.20E-20	7.16E-19
**BBS7**	0.86	1.81	4.21E-20	7.18E-19
**TMEM8B**	1.70	3.24	4.38E-20	7.45E-19
**NCL**	-0.56	0.68	4.91E-20	8.35E-19
**POLD3**	-0.84	0.56	5.13E-20	8.71E-19
**NHLH2**	2.29	4.89	5.88E-20	9.98E-19
**CTSC**	-0.47	0.72	5.92E-20	1.00E-18
**PAPLN**	2.26	4.78	6.80E-20	1.15E-18
**GALNT11**	0.82	1.76	6.86E-20	1.16E-18
**S1PR1**	-1.43	0.37	7.58E-20	1.28E-18
**FLJ16779**	3.56	11.82	7.74E-20	1.31E-18
**VIM**	-0.80	0.58	8.92E-20	1.51E-18
**C20orf27**	-0.90	0.53	9.37E-20	1.58E-18
**AGPAT5**	-0.76	0.59	9.39E-20	1.58E-18
**MTHFD2**	-0.74	0.60	9.66E-20	1.63E-18
**TJP2**	0.53	1.44	1.02E-19	1.72E-18
**ATF5**	0.87	1.83	1.34E-19	2.24E-18
**USP13**	-1.40	0.38	1.40E-19	2.34E-18
**CNOT6**	0.69	1.62	1.45E-19	2.43E-18
**ASUN**	-0.80	0.57	1.48E-19	2.48E-18
**MAGED1**	0.59	1.50	1.61E-19	2.70E-18
**AQP3**	0.55	1.46	1.67E-19	2.79E-18
**DVL1**	-0.62	0.65	1.72E-19	2.87E-18
**MUC19**	3.17	9.01	1.73E-19	2.89E-18
**TLE3**	-0.76	0.59	1.74E-19	2.90E-18
**CHST3**	0.68	1.60	1.78E-19	2.96E-18
**SLC9B2**	-1.30	0.41	1.78E-19	2.97E-18
**CDR2**	-1.06	0.48	1.79E-19	2.98E-18
**SLC25A20**	1.20	2.29	1.92E-19	3.19E-18
**DSE**	0.55	1.47	1.97E-19	3.27E-18
**H19**	0.66	1.58	2.03E-19	3.36E-18
**PLK2**	0.51	1.42	2.05E-19	3.40E-18
**PCDH1**	-0.60	0.66	2.15E-19	3.55E-18
**GALNT18**	-0.89	0.54	2.45E-19	4.05E-18
**FAM196A**	3.07	8.41	2.62E-19	4.32E-18
**INPPL1**	0.75	1.68	2.62E-19	4.33E-18
**MEG3**	2.66	6.33	2.70E-19	4.45E-18
**AATBC**	2.17	4.50	2.79E-19	4.59E-18
**POLE2**	-1.36	0.39	2.98E-19	4.91E-18
**ATG4A**	0.89	1.85	3.00E-19	4.94E-18
**BTBD19**	0.15	1.11	3.15E-02	NA
**CHORDC1**	-0.15	0.90	3.16E-02	3.58E-01
**VEGFC**	-0.16	0.89	3.17E-02	3.59E-01
**DUOXA2**	0.10	1.07	3.17E-02	NA
**CYFIP2**	0.14	1.10	3.18E-02	NA
**ALAS1**	0.12	1.08	3.19E-02	3.61E-01
**CAMK2G**	0.15	1.11	3.20E-02	3.62E-01
**MSANTD3**	-0.17	0.89	3.23E-02	3.64E-01
**SH2D2A**	0.18	1.14	3.23E-02	3.64E-01
**HMHA1**	-0.19	0.88	3.23E-02	3.64E-01
**AMD1**	-0.10	0.93	3.24E-02	3.64E-01
**IGSF9**	-0.13	0.91	3.25E-02	3.64E-01
**CSPG4P11**	-0.11	0.93	3.25E-02	NA
**HSPA5**	-0.08	0.94	3.25E-02	3.64E-01
**ENC1**	-0.14	0.91	3.25E-02	3.64E-01
**EIF4G2**	-0.08	0.95	3.25E-02	3.64E-01
**B3GALT6**	-0.18	0.88	3.26E-02	3.65E-01
**CDH16**	-0.18	0.88	3.28E-02	NA
**TMEM184A**	-0.13	0.91	3.28E-02	3.66E-01
**MBD5**	0.17	1.13	3.28E-02	3.66E-01
**GS1-393G12.13**	-0.04	0.97	3.28E-02	NA
**FLVCR1**	-0.18	0.88	3.30E-02	3.67E-01
**BTN2A2**	0.19	1.14	3.30E-02	3.67E-01
**B4GAT1**	0.18	1.13	3.30E-02	3.67E-01
**PPP4R1**	-0.10	0.93	3.31E-02	3.68E-01
**TIMELESS**	-0.13	0.92	3.32E-02	3.68E-01
**MBTPS1**	0.11	1.08	3.32E-02	3.68E-01
**PKD2**	0.17	1.13	3.32E-02	3.68E-01
**ALDOC**	-0.16	0.89	3.33E-02	3.69E-01
**UPK1A**	-0.08	0.95	3.35E-02	NA
**RORA**	-0.18	0.88	3.35E-02	3.69E-01
**KIAA1161**	-0.15	0.90	3.35E-02	3.69E-01
**SPINK6**	-0.11	0.92	3.35E-02	3.69E-01
**RILPL1**	0.18	1.13	3.35E-02	3.69E-01
**GCNT2**	-0.18	0.88	3.36E-02	3.70E-01
**GLTSCR1**	-0.18	0.88	3.37E-02	3.70E-01
**LINC00640**	-0.05	0.97	3.39E-02	NA
**DDX3X**	-0.10	0.93	3.39E-02	3.73E-01
**YY1**	-0.11	0.93	3.39E-02	3.73E-01
**HAS2**	-0.17	0.89	3.41E-02	NA
**CCDC90B**	0.15	1.11	3.41E-02	3.73E-01
**SPINK5**	-0.12	0.92	3.41E-02	3.73E-01
**HNRNPD**	-0.10	0.93	3.41E-02	3.73E-01
**CHRAC1**	-0.15	0.90	3.42E-02	3.73E-01
**MYADM**	-0.14	0.91	3.43E-02	3.74E-01
**ING1**	-0.18	0.88	3.43E-02	3.74E-01
**THOP1**	-0.12	0.92	3.44E-02	3.75E-01
**SLC4A7**	0.15	1.11	3.45E-02	3.76E-01
**KIF5A**	0.09	1.06	3.46E-02	NA
**NAT10**	-0.11	0.93	3.46E-02	3.77E-01
**TRAFD1**	0.15	1.11	3.47E-02	3.77E-01
**VAMP2**	0.17	1.13	3.50E-02	3.79E-01
**ICMT**	-0.10	0.93	3.50E-02	3.79E-01
**AP1S3**	0.15	1.11	3.50E-02	3.79E-01
**HNRNPA0**	-0.11	0.93	3.51E-02	3.79E-01
**SFTA1P**	-0.15	0.90	3.52E-02	NA
**CBX2**	-0.17	0.89	3.52E-02	3.80E-01
**HYAL1**	0.16	1.12	3.52E-02	3.80E-01
**LRRC75B**	0.18	1.14	3.52E-02	3.80E-01
**AC091729.9**	0.17	1.13	3.53E-02	NA
**CKAP2**	-0.12	0.92	3.54E-02	3.81E-01
**ALMS1**	-0.17	0.89	3.55E-02	3.82E-01
**TMEM147**	-0.13	0.92	3.55E-02	3.82E-01
**CEACAM19**	-0.18	0.88	3.56E-02	3.83E-01
**ZNF555**	0.18	1.13	3.57E-02	NA
**ZNF266**	0.17	1.13	3.57E-02	3.83E-01
**SETD1A**	-0.14	0.91	3.58E-02	3.83E-01
**CTSC**	-0.08	0.94	3.58E-02	3.83E-01
**RP1-102E24.1**	-0.14	0.91	3.58E-02	NA
**IMP3**	-0.16	0.89	3.59E-02	3.83E-01
**RPF2**	-0.13	0.92	3.60E-02	3.84E-01
**ZNF318**	-0.13	0.92	3.60E-02	3.84E-01
**ADCK3**	0.18	1.13	3.60E-02	3.84E-01
**GLUL**	0.10	1.07	3.61E-02	3.85E-01
**TTLL5**	-0.14	0.91	3.62E-02	3.85E-01
**ZNF613**	0.17	1.13	3.63E-02	NA
**BARD1**	-0.17	0.89	3.64E-02	3.87E-01
**DNAH5**	-0.15	0.90	3.65E-02	NA
**TM9SF1**	-0.18	0.89	3.65E-02	NA
**VEZF1**	-0.15	0.90	3.65E-02	3.88E-01
**SESN2**	0.16	1.12	3.67E-02	3.89E-01
**LANCL2**	-0.17	0.89	3.68E-02	3.90E-01
**CYP20A1**	-0.18	0.88	3.69E-02	3.91E-01
**CYP1A1**	-0.09	0.94	3.71E-02	NA
**EPS8L2**	0.11	1.08	3.72E-02	3.91E-01
**GPR108**	0.13	1.09	3.72E-02	3.91E-01
**FOS**	0.15	1.11	3.72E-02	3.91E-01
**TRIM2**	-0.14	0.91	3.72E-02	3.91E-01
**ERVMER34-1**	-0.17	0.89	3.72E-02	3.91E-01
**LARP1**	-0.10	0.93	3.72E-02	3.91E-01

CDC25A	-1.02	0.49	3.03E-19	4.97E-18
SHMT2	-0.61	0.66	3.22E-19	5.29E-18
GPR155	1.47	2.77	3.28E-19	5.38E-18
TAF3	0.92	1.90	3.30E-19	5.40E-18
FBRSL1	-0.78	0.58	3.53E-19	5.78E-18
TECPR2	0.80	1.74	3.56E-19	5.81E-18
TCERG1	-0.61	0.65	3.81E-19	6.22E-18
SDK2	0.91	1.87	3.85E-19	6.28E-18
PRICKLE2	1.39	2.63	3.95E-19	6.44E-18
TENM4	-1.17	0.44	4.02E-19	6.55E-18
PITX1	-0.86	0.55	4.12E-19	6.70E-18
CDC7	-1.08	0.47	4.14E-19	6.73E-18
SLC43A3	-1.10	0.47	4.16E-19	6.75E-18
CHD7	-0.93	0.52	4.26E-19	6.91E-18
TRIM59	-1.47	0.36	4.29E-19	6.96E-18
PDE4A	2.59	6.03	4.43E-19	7.18E-18
TMEM2	0.77	1.70	4.47E-19	7.23E-18
OASL	1.44	2.72	4.58E-19	7.41E-18
HIBCH	1.07	2.10	4.99E-19	8.06E-18
LARP1	-0.58	0.67	5.45E-19	8.80E-18
OGDH	0.52	1.44	5.50E-19	8.86E-18
MON2	0.78	1.72	5.51E-19	8.88E-18
CLCN5	-1.01	0.50	5.98E-19	9.62E-18
PRIM1	-1.20	0.44	6.53E-19	1.05E-17
TOPBP1	-0.70	0.61	6.61E-19	1.06E-17
TSC22D1	0.57	1.49	6.67E-19	1.07E-17
GALE	0.64	1.56	7.38E-19	1.18E-17
TRAK1	0.60	1.51	7.90E-19	1.27E-17
PTAFR	0.72	1.65	8.17E-19	1.31E-17
ZFP90	0.89	1.85	8.45E-19	1.35E-17
UAP1	-0.59	0.67	1.03E-18	1.64E-17
PLXNA2	0.62	1.54	1.07E-18	1.70E-17
QSER1	-0.65	0.64	1.08E-18	1.72E-17
APEX2	0.69	1.61	1.10E-18	1.75E-17
RP3-510D11.2	2.43	5.37	1.11E-18	1.77E-17
CCDC127	-1.30	0.40	1.13E-18	1.79E-17
SMC3	-0.64	0.64	1.17E-18	1.86E-17
CDV3	-0.56	0.68	1.19E-18	1.89E-17
TERF2IP	0.77	1.71	1.21E-18	1.92E-17
SMARCC1	-0.51	0.70	1.23E-18	1.95E-17
LOXL2	-2.01	0.25	1.31E-18	2.08E-17
NOP14-AS1	0.98	1.97	1.46E-18	2.31E-17
STAG1	-0.93	0.52	1.46E-18	2.31E-17
SMYD5	-0.89	0.54	1.46E-18	2.31E-17
ACLY	-0.51	0.70	1.51E-18	2.39E-17
TNFAIP1	0.53	1.44	1.55E-18	2.44E-17
HMGB1	-0.56	0.68	1.69E-18	2.67E-17
RECQL4	-0.91	0.53	1.71E-18	2.68E-17
C11orf24	0.78	1.72	1.75E-18	2.75E-17
ANKRA2	1.09	2.14	1.76E-18	2.77E-17
HNRNPA0	-0.67	0.63	1.78E-18	2.79E-17
ADCY6	0.79	1.74	1.88E-18	2.95E-17
SERPINH1	-0.69	0.62	1.91E-18	3.00E-17
IRF2BPL	0.73	1.66	1.92E-18	3.00E-17
EFHD2	-0.62	0.65	2.04E-18	3.19E-17
EFNB1	0.54	1.45	2.15E-18	3.36E-17
EML2	0.61	1.53	2.19E-18	3.41E-17
IGFBP4	0.89	1.85	2.32E-18	3.62E-17
DSG4	-1.37	0.39	2.57E-18	4.00E-17
XPR1	0.73	1.66	2.79E-18	4.35E-17
SNRPD1	-0.68	0.62	2.80E-18	4.35E-17
CCNK	0.95	1.94	2.95E-18	4.59E-17
MN1	0.75	1.68	3.08E-18	4.79E-17
PTGFRN	-0.52	0.70	3.09E-18	4.79E-17
APOBEC3F	1.59	3.01	3.32E-18	5.14E-17
HMGXB3	0.70	1.62	3.53E-18	5.47E-17
RINL	1.43	2.69	3.55E-18	5.49E-17
LRRC20	-1.48	0.36	3.58E-18	5.54E-17
USP28	-0.77	0.59	3.68E-18	5.69E-17
STT3A	-0.55	0.69	4.01E-18	6.19E-17
STX6	0.67	1.59	4.03E-18	6.21E-17
DRAM1	1.04	2.06	4.15E-18	6.39E-17
NQO1	0.71	1.64	4.25E-18	6.54E-17
DLK2	-1.00	0.50	4.31E-18	6.63E-17
TCF7L1	-0.95	0.52	4.51E-18	6.94E-17
IVNS1ABP	-0.63	0.65	4.58E-18	7.03E-17
PTMS	-0.75	0.59	4.59E-18	7.04E-17
COL12A1	-0.57	0.67	4.59E-18	7.04E-17
LRRC8D	-0.79	0.58	4.95E-18	7.58E-17
IRS2	1.17	2.25	5.23E-18	8.00E-17
FBXL18	0.90	1.87	5.31E-18	8.11E-17
KCNQ5	-1.65	0.32	5.31E-18	8.11E-17
ZSWIM6	0.93	1.90	5.31E-18	8.11E-17
FPGS	-0.66	0.63	5.36E-18	8.17E-17
TINF2	0.65	1.57	5.36E-18	8.17E-17
TBL1XR1	-0.70	0.61	5.48E-18	8.35E-17
CCNG1	0.74	1.66	5.83E-18	8.87E-17
MAP3K10	1.01	2.01	6.12E-18	9.30E-17
GNAI1	0.73	1.65	6.13E-18	9.30E-17
HMGCL	0.84	1.79	6.25E-18	9.47E-17
FSCN1	-0.10	0.93	3.72E-02	3.91E-01
UST	0.17	1.13	3.73E-02	3.92E-01
PPM1A	0.15	1.11	3.74E-02	3.92E-01
PBXIP1	0.17	1.12	3.75E-02	3.92E-01
HNRNPUL1	-0.09	0.94	3.75E-02	3.92E-01
NOMO1	0.13	1.10	3.75E-02	3.92E-01
ENTPD4	0.14	1.10	3.76E-02	3.92E-01
PARD6G	-0.15	0.90	3.77E-02	3.93E-01
ISYNA1	0.18	1.13	3.77E-02	3.93E-01
IGFL2	-0.15	0.90	3.79E-02	3.94E-01
GDE1	0.12	1.09	3.79E-02	3.94E-01
NLGN2	0.18	1.13	3.80E-02	3.94E-01
C4orf48	-0.17	0.89	3.80E-02	NA
FAM122C	0.11	1.08	3.80E-02	NA
RFC3	-0.14	0.91	3.80E-02	3.94E-01
PLCB2	-0.14	0.91	3.81E-02	NA
BAG5	-0.13	0.91	3.81E-02	3.94E-01
POLR2L	-0.12	0.92	3.81E-02	3.94E-01
PPP1R26	0.14	1.10	3.81E-02	3.94E-01
EEF1GP3	-0.10	0.94	3.81E-02	NA
MMAB	-0.16	0.90	3.82E-02	3.94E-01
STX16	0.14	1.10	3.82E-02	3.94E-01
ESCO2	-0.17	0.89	3.82E-02	3.94E-01
NMD3	0.13	1.09	3.84E-02	3.96E-01
CASP14	-0.18	0.88	3.85E-02	3.96E-01
FRG1	0.16	1.12	3.85E-02	3.96E-01
STK17A	0.11	1.08	3.85E-02	3.96E-01
NAPB	0.18	1.13	3.86E-02	3.96E-01
FAXC	-0.12	0.92	3.87E-02	NA
MUC16	-0.16	0.89	3.88E-02	NA
AJUBA	-0.12	0.92	3.88E-02	3.98E-01
NUPR1	0.15	1.11	3.89E-02	3.98E-01
FIGNL1	-0.15	0.90	3.90E-02	3.99E-01
AIMP2	-0.15	0.90	3.90E-02	3.99E-01
RP11-567G24.1	-0.14	0.91	3.92E-02	NA
TUBB4B	-0.09	0.94	3.93E-02	4.01E-01
RP11-696N14.1	0.15	1.11	3.93E-02	NA
SPATS2	0.13	1.09	3.93E-02	4.01E-01
ARHGAP42	0.16	1.12	3.93E-02	NA
SLC4A2	-0.13	0.91	3.94E-02	4.01E-01
FYN	0.14	1.10	3.94E-02	4.01E-01
ACO2	0.11	1.08	3.94E-02	4.01E-01
ERI1	-0.16	0.89	3.95E-02	4.01E-01
SPTLC2	-0.12	0.92	3.96E-02	4.02E-01
TMEM106B	0.14	1.10	3.97E-02	4.02E-01
ELOVL4	-0.16	0.89	3.97E-02	4.02E-01
ATP9A	0.15	1.11	4.00E-02	4.04E-01
BTBD10	0.12	1.08	4.00E-02	4.04E-01
SERPINH1	-0.11	0.92	4.01E-02	4.05E-01
TBX18	-0.07	0.95	4.01E-02	NA
TRIM41	0.14	1.10	4.02E-02	4.05E-01
FBL	-0.09	0.94	4.02E-02	4.05E-01
PKN3	0.17	1.13	4.02E-02	4.05E-01
ROGDI	0.16	1.11	4.03E-02	4.05E-01
ELOVL7	0.17	1.12	4.04E-02	4.06E-01
GCSH	-0.15	0.90	4.04E-02	NA
LTBP4	0.13	1.09	4.04E-02	4.06E-01
PRKAR1A	0.11	1.08	4.05E-02	4.07E-01
KIAA1958	0.18	1.13	4.06E-02	4.07E-01
SALL4	0.07	1.05	4.06E-02	NA
LINC00657	0.10	1.07	4.07E-02	4.07E-01
GDF11	0.18	1.13	4.07E-02	4.07E-01
EVI2B	-0.06	0.96	4.08E-02	NA
MAK16	-0.15	0.90	4.08E-02	4.07E-01
CDC42EP1	-0.13	0.91	4.09E-02	4.07E-01
MEST	-0.18	0.89	4.09E-02	4.07E-01
STEAP2	0.18	1.13	4.09E-02	4.07E-01
LETMD1	0.16	1.12	4.09E-02	4.07E-01
ANKRD18A	0.17	1.12	4.09E-02	NA
MRC2	0.13	1.09	4.09E-02	4.07E-01
42983	-0.18	0.89	4.11E-02	4.07E-01
SDCBP	0.11	1.08	4.11E-02	4.07E-01
TMEM173	0.14	1.10	4.11E-02	4.07E-01
CYP2S1	0.16	1.12	4.11E-02	4.07E-01
NUCB1	0.13	1.09	4.12E-02	4.07E-01
RPS13P2	-0.18	0.88	4.13E-02	4.08E-01
TRAF4	0.13	1.09	4.15E-02	4.09E-01
GLT8D1	0.14	1.10	4.16E-02	4.10E-01
NAB2	-0.13	0.92	4.16E-02	4.10E-01
TUT1	0.18	1.13	4.16E-02	4.10E-01
GAPDHP60	-0.17	0.89	4.16E-02	4.10E-01
PDDC1	0.14	1.10	4.17E-02	4.10E-01
KRTAP2-3	-0.09	0.94	4.18E-02	NA
PNISR	0.14	1.10	4.18E-02	4.10E-01
RPL19P21	-0.08	0.95	4.19E-02	NA
SP110	-0.17	0.89	4.19E-02	4.10E-01
PALMD	-0.17	0.89	4.19E-02	4.10E-01
RP11-347C18.3	0.07	1.05	4.19E-02	NA
ABLIM3	-0.16	0.90	4.20E-02	4.11E-01
SLC9A3R2	0.17	1.13	4.21E-02	4.12E-01

FUBP1	-0.57	0.67	6.27E-18	9.50E-17
SUPT7L	0.77	1.71	6.52E-18	9.87E-17
PSAT1	-0.70	0.62	6.59E-18	9.98E-17
DBNDD1	1.05	2.07	6.72E-18	1.02E-16
PGPEP1	1.47	2.77	6.97E-18	1.05E-16
CCDC8	0.82	1.77	7.14E-18	1.08E-16
PDK1	-0.94	0.52	7.30E-18	1.10E-16
RRS1	-0.83	0.56	7.33E-18	1.10E-16
NEO1	1.01	2.02	7.51E-18	1.13E-16
SPRYD4	1.10	2.14	7.95E-18	1.20E-16
SFXN5	1.23	2.34	8.28E-18	1.24E-16
LRRC58	-0.82	0.57	8.58E-18	1.29E-16
VRK2	-0.81	0.57	8.75E-18	1.31E-16
NANOS1	2.14	4.40	9.18E-18	1.38E-16
CELF2	0.76	1.70	9.22E-18	1.38E-16
FKBP9	-0.54	0.69	9.69E-18	1.45E-16
PLXNB2	0.53	1.44	9.72E-18	1.45E-16
C17orf80	0.95	1.93	9.81E-18	1.47E-16
UBE2J1	-0.76	0.59	9.90E-18	1.48E-16
PES1	-0.65	0.64	1.08E-17	1.61E-16
EPHB3	0.72	1.64	1.10E-17	1.64E-16
THAP4	-0.66	0.63	1.26E-17	1.88E-16
PKP4	-0.58	0.67	1.27E-17	1.90E-16
RFNG	0.85	1.80	1.30E-17	1.94E-16
ZNF318	-0.67	0.63	1.36E-17	2.02E-16
B4GALNT3	-0.73	0.60	1.38E-17	2.05E-16
DYNC1LI2	0.55	1.46	1.42E-17	2.10E-16
TCP11L1	0.89	1.86	1.43E-17	2.11E-16
MBD2	-0.61	0.66	1.74E-17	2.58E-16
LYAR	-0.71	0.61	1.87E-17	2.76E-16
NDC1	-0.64	0.64	1.91E-17	2.82E-16
RIF1	-0.81	0.57	1.93E-17	2.85E-16
TSHZ1	-1.02	0.49	1.94E-17	2.86E-16
TFDP1	-0.49	0.71	2.00E-17	2.95E-16
DCPS	-0.92	0.53	2.00E-17	2.95E-16
STEAP4	0.89	1.85	2.01E-17	2.95E-16
EPN3	0.67	1.59	2.03E-17	2.99E-16
GOLGB1	0.70	1.62	2.08E-17	3.05E-16
C1orf21	-0.74	0.60	2.09E-17	3.07E-16
XPO5	-0.60	0.66	2.11E-17	3.09E-16
ANKFY1	0.59	1.50	2.12E-17	3.11E-16
LRAT	-1.07	0.48	2.15E-17	3.15E-16
MAD2L2	-0.87	0.55	2.21E-17	3.24E-16
CPT1A	-0.66	0.64	2.36E-17	3.45E-16
TRIM8	0.64	1.56	2.37E-17	3.46E-16
APPBP2	0.84	1.79	2.38E-17	3.48E-16
NISCH	0.62	1.53	2.41E-17	3.52E-16
ATP2C2	-1.21	0.43	2.45E-17	3.57E-16
PHB2	-0.50	0.71	2.46E-17	3.58E-16
PKMYT1	-0.78	0.58	2.50E-17	3.63E-16
UBE3B	0.68	1.60	2.77E-17	4.03E-16
PNLIPRP3	-0.89	0.54	2.91E-17	4.23E-16
GNS	0.58	1.49	2.93E-17	4.25E-16
EIF2B2	0.62	1.54	2.93E-17	4.25E-16
ANXA5	-0.53	0.69	3.05E-17	4.41E-16
AIM1	-0.57	0.67	3.13E-17	4.53E-16
NDRG2	-0.73	0.60	3.47E-17	5.03E-16
TMEM184A	-0.73	0.60	3.62E-17	5.24E-16
NT5DC2	-0.72	0.61	3.64E-17	5.26E-16
FRMD8	0.67	1.59	3.80E-17	5.48E-16
NUP153	-0.54	0.69	4.07E-17	5.87E-16
NAV3	-1.43	0.37	4.24E-17	6.10E-16
NFE2L1	-0.45	0.73	4.25E-17	6.11E-16
ST3GAL4	-0.94	0.52	4.29E-17	6.17E-16
CDK4	-0.84	0.56	4.41E-17	6.33E-16
RTN4R	-1.38	0.38	4.43E-17	6.36E-16
CCS	0.89	1.86	4.45E-17	6.38E-16
KLHL36	-0.64	0.64	4.64E-17	6.65E-16
VEGFB	-1.06	0.48	4.73E-17	6.78E-16
CCDC92	0.87	1.83	4.75E-17	6.80E-16
SGK223	0.69	1.61	4.78E-17	6.83E-16
MTA2	0.50	1.42	4.80E-17	6.85E-16
TNC	-0.69	0.62	4.82E-17	6.88E-16
WNT10A	-1.36	0.39	5.14E-17	7.33E-16
RP4-794I6.4	2.38	5.21	5.37E-17	7.65E-16
OBSL1	0.81	1.76	5.38E-17	7.66E-16
MPZL1	-0.48	0.72	5.38E-17	7.66E-16
COA7	-0.71	0.61	5.52E-17	7.85E-16
MANBA	1.02	2.02	5.66E-17	8.05E-16
NSG1	-0.62	0.65	5.70E-17	8.10E-16
SYNE2	-0.86	0.55	5.89E-17	8.35E-16
GIT1	0.62	1.53	5.99E-17	8.49E-16
GABARAPL1	1.21	2.32	6.34E-17	8.98E-16
SEC14L1	0.61	1.53	6.45E-17	9.13E-16
MT-RNR2	0.63	1.55	6.49E-17	9.18E-16
SDF2L1	-1.19	0.44	6.50E-17	9.19E-16
ENOPH1	-0.69	0.62	6.59E-17	9.31E-16
ALDH1L2	-1.18	0.44	6.72E-17	9.48E-16
COL27A1	0.75	1.68	6.74E-17	9.50E-16
SUSD6	0.77	1.70	6.76E-17	9.52E-16
RWDD2A	0.15	1.11	4.21E-02	NA
CHCHD2P2	-0.10	0.93	4.21E-02	NA
CTSZ	0.12	1.09	4.21E-02	4.12E-01
LANCL1	0.12	1.09	4.22E-02	4.12E-01
LEMD3	-0.15	0.90	4.23E-02	4.12E-01
FAM213B	-0.13	0.91	4.23E-02	4.12E-01
AC016734.2	-0.12	0.92	4.25E-02	NA
WDR45B	-0.11	0.93	4.25E-02	4.14E-01
POLE	-0.11	0.93	4.26E-02	4.14E-01
CC2D2A	-0.16	0.90	4.27E-02	4.14E-01
SLC9A7	0.15	1.11	4.27E-02	4.14E-01
LRRC41	0.11	1.08	4.27E-02	4.15E-01
C14orf80	-0.17	0.89	4.29E-02	4.15E-01
ARMC6	-0.14	0.91	4.29E-02	4.15E-01
VWA5A	0.14	1.10	4.29E-02	4.15E-01
BAZ1B	-0.11	0.93	4.30E-02	4.15E-01
PEAR1	0.18	1.13	4.31E-02	4.16E-01
DCLRE1B	-0.15	0.90	4.32E-02	4.17E-01
RFWD3	-0.12	0.92	4.35E-02	4.19E-01
FAM185A	0.16	1.12	4.35E-02	NA
TOMM22	-0.11	0.93	4.38E-02	4.21E-01
ABCC3	0.13	1.10	4.38E-02	4.21E-01
USP53	0.13	1.10	4.38E-02	4.21E-01
ST5	0.13	1.09	4.40E-02	4.22E-01
ECT2	-0.12	0.92	4.41E-02	4.23E-01
ENO1	-0.08	0.95	4.41E-02	4.23E-01
MIA3	0.13	1.10	4.42E-02	4.23E-01
TMUB1	-0.14	0.91	4.42E-02	4.23E-01
RSU1	-0.11	0.93	4.42E-02	4.23E-01
LIPT2	-0.11	0.92	4.44E-02	NA
LRG1	0.17	1.13	4.45E-02	4.25E-01
ACAD9	-0.13	0.91	4.46E-02	4.25E-01
PSTPIP2	0.13	1.09	4.47E-02	4.26E-01
ADH5	0.11	1.08	4.48E-02	4.26E-01
MPHOSPH10	-0.13	0.91	4.48E-02	4.26E-01
PPP1R3B	-0.16	0.90	4.49E-02	4.27E-01
AGO2	-0.14	0.91	4.49E-02	4.27E-01
CDH24	0.17	1.13	4.50E-02	4.27E-01
RP11-166O4.6	-0.04	0.97	4.51E-02	NA
FER1L5	-0.06	0.96	4.53E-02	NA
MBD2	-0.11	0.93	4.54E-02	4.30E-01
LYPLA1	0.12	1.09	4.54E-02	4.30E-01
HEATR1	-0.11	0.92	4.55E-02	4.31E-01
SLC39A6	0.10	1.07	4.56E-02	4.31E-01
ZBTB11	0.14	1.11	4.57E-02	4.31E-01
CENPP	0.17	1.13	4.57E-02	4.31E-01
CALML3-AS1	0.15	1.11	4.58E-02	NA
RAD51AP1	-0.16	0.89	4.58E-02	4.32E-01
IFRD2	-0.11	0.92	4.59E-02	4.32E-01
CTBS	0.17	1.13	4.59E-02	4.32E-01
FAM110A	-0.15	0.90	4.61E-02	4.33E-01
FAM3D	-0.13	0.91	4.63E-02	NA
ZDHHC1	0.17	1.13	4.63E-02	4.35E-01
ZCCHC3	-0.15	0.90	4.64E-02	4.35E-01
PI4K2A	0.13	1.09	4.64E-02	4.35E-01
SLC25A13	-0.12	0.92	4.64E-02	4.35E-01
PDPN	-0.15	0.90	4.65E-02	4.35E-01
ABI2	0.15	1.11	4.65E-02	4.35E-01
CTD-2666L21.1	-0.05	0.97	4.67E-02	NA
LINC01116	-0.14	0.90	4.67E-02	NA
METTL22	0.17	1.12	4.67E-02	4.35E-01
SLC16A2	-0.12	0.92	4.67E-02	4.35E-01
PPL	0.08	1.06	4.67E-02	4.35E-01
SCP2	0.12	1.09	4.68E-02	4.35E-01
FAM167A	-0.16	0.89	4.68E-02	4.35E-01
GRHL1	-0.12	0.92	4.68E-02	4.35E-01
RP11-325K4.3	-0.04	0.97	4.69E-02	NA
RP11-526I2.5	-0.08	0.95	4.70E-02	NA
TMEM68	0.16	1.12	4.70E-02	4.36E-01
BOP1	-0.17	0.89	4.71E-02	4.36E-01
CROCCP2	0.17	1.13	4.71E-02	4.36E-01
BCL2L1	0.11	1.08	4.71E-02	4.36E-01
MAP3K13	0.16	1.12	4.73E-02	4.37E-01
NOSIP	-0.14	0.91	4.73E-02	4.37E-01
C9orf16	0.13	1.10	4.73E-02	4.37E-01
KIF13B	0.15	1.11	4.74E-02	4.37E-01
SPAG5	-0.12	0.92	4.75E-02	4.38E-01
TMEM160	-0.17	0.89	4.76E-02	4.39E-01
MOAP1	0.17	1.12	4.77E-02	4.39E-01
TMEM19	-0.16	0.89	4.82E-02	4.42E-01
CAPN7	0.13	1.10	4.82E-02	4.42E-01
RPL3	0.07	1.05	4.82E-02	4.42E-01
KDM4D	-0.16	0.90	4.83E-02	NA
RP11-10C24.1	-0.11	0.93	4.83E-02	NA
C9orf91	-0.16	0.89	4.84E-02	4.43E-01
IL4R	0.11	1.08	4.84E-02	4.43E-01
LRRC20	0.17	1.12	4.84E-02	4.43E-01
GPR180	-0.14	0.90	4.84E-02	4.43E-01
CTD-2201G3.1	-0.05	0.97	4.85E-02	NA
CNP	-0.11	0.93	4.85E-02	4.43E-01

**CCDC85C**	-0.65	0.64	6.77E-17	9.53E-16
**SLC39A14**	-0.91	0.53	6.92E-17	9.73E-16
**PLPP2**	-1.60	0.33	7.28E-17	1.02E-15
**KLRG2**	1.71	3.28	7.41E-17	1.04E-15
**VILL**	-1.14	0.45	7.51E-17	1.05E-15
**HAAO**	3.32	9.95	7.63E-17	1.07E-15
**ALDOC**	-1.05	0.48	7.76E-17	1.09E-15
**HPDL**	-2.46	0.18	7.91E-17	1.11E-15
**SOX4**	0.72	1.65	8.14E-17	1.14E-15
**DQX1**	1.65	3.14	8.20E-17	1.15E-15
**SLC2A4RG**	-0.63	0.65	8.35E-17	1.17E-15
**PHLDB3**	0.77	1.70	8.62E-17	1.20E-15
**PPP1R14B**	-0.58	0.67	8.72E-17	1.22E-15
**RGS10**	-0.94	0.52	8.90E-17	1.24E-15
**LAMC1**	0.45	1.36	9.12E-17	1.27E-15
**UCK2**	-0.66	0.63	9.14E-17	1.27E-15
**POGK**	-0.65	0.64	9.22E-17	1.28E-15
**ZNF655**	0.69	1.61	9.36E-17	1.30E-15
**PINK1**	0.87	1.83	9.44E-17	1.31E-15
**IMPA2**	-0.79	0.58	9.63E-17	1.34E-15
**AGPAT3**	-0.57	0.68	9.80E-17	1.36E-15
**CITED2**	0.81	1.75	9.84E-17	1.36E-15
**FRMD4B**	-0.65	0.64	1.04E-16	1.44E-15
**RP1-302G2.5**	3.31	9.89	1.07E-16	1.48E-15
**PARP2**	-0.88	0.54	1.09E-16	1.50E-15
**KDM4B**	0.70	1.63	1.12E-16	1.55E-15
**EIF3E**	-0.51	0.70	1.18E-16	1.63E-15
**ANXA7**	-0.55	0.68	1.22E-16	1.68E-15
**ZNF425**	1.50	2.84	1.22E-16	1.68E-15
**PTP4A2**	-0.57	0.67	1.23E-16	1.69E-15
**TRIM35**	0.71	1.64	1.24E-16	1.70E-15
**NME4**	-0.66	0.63	1.28E-16	1.76E-15
**PTK6**	-0.72	0.61	1.30E-16	1.78E-15
**EIF5A**	-0.57	0.68	1.30E-16	1.79E-15
**IER5**	0.52	1.44	1.39E-16	1.91E-15
**ZC3HAV1**	0.56	1.47	1.44E-16	1.98E-15
**RP13-463N16.6**	1.78	3.43	1.46E-16	2.00E-15
**DTL**	-0.84	0.56	1.51E-16	2.06E-15
**SMCHD1**	-0.68	0.63	1.53E-16	2.09E-15
**PUS7**	-0.81	0.57	1.74E-16	2.38E-15
**ACTG1**	-0.57	0.68	1.80E-16	2.45E-15
**FGD5-AS1**	-0.61	0.65	1.82E-16	2.48E-15
**ARHGDIA**	-0.51	0.70	1.99E-16	2.71E-15
**RAPGEF1**	-0.69	0.62	2.07E-16	2.82E-15
**PRKACA**	0.59	1.51	2.28E-16	3.09E-15
**DUSP10**	1.18	2.27	2.36E-16	3.20E-15
**BIK**	1.56	2.95	2.38E-16	3.23E-15
**DTX2**	0.76	1.69	2.42E-16	3.28E-15
**PCYOX1L**	1.01	2.01	2.43E-16	3.30E-15
**ARHGAP33**	-2.52	0.17	2.48E-16	3.36E-15
**BMP2**	1.34	2.53	2.49E-16	3.37E-15
**RGMB**	-0.87	0.55	2.51E-16	3.39E-15
**SH3KBP1**	-0.58	0.67	2.58E-16	3.49E-15
**CYTH1**	0.72	1.64	2.62E-16	3.54E-15
**SH3BP1**	-0.61	0.66	2.69E-16	3.62E-15
**LCE1E**	2.86	7.25	2.89E-16	3.89E-15
**PXT1**	3.32	9.96	2.89E-16	3.90E-15
**CORO2B**	2.52	5.72	2.90E-16	3.90E-15
**CROCC**	0.80	1.74	3.04E-16	4.08E-15
**CNBP**	-0.45	0.73	3.08E-16	4.14E-15
**SOX13**	-0.87	0.55	3.15E-16	4.23E-15
**CCDC60**	3.16	8.97	3.15E-16	4.23E-15
**FAM83C**	-0.57	0.68	3.19E-16	4.27E-15
**HNRNPUL1**	-0.49	0.71	3.32E-16	4.45E-15
**HAS3**	0.48	1.39	3.35E-16	4.49E-15
**TRIM3**	0.93	1.90	3.45E-16	4.61E-15
**NLRP10**	1.11	2.15	3.66E-16	4.89E-15
**HSPB1**	-0.61	0.66	3.74E-16	5.00E-15
**NOP16**	-0.70	0.62	3.96E-16	5.29E-15
**ELL**	0.84	1.78	4.02E-16	5.36E-15
**TRIM5**	0.66	1.58	4.24E-16	5.66E-15
**TSC22D3**	-0.93	0.53	4.25E-16	5.66E-15
**ZC3H12C**	0.97	1.95	4.26E-16	5.67E-15
**DLX1**	-1.76	0.29	4.29E-16	5.71E-15
**SNX2**	0.65	1.56	4.44E-16	5.90E-15
**S100A3**	1.05	2.07	4.62E-16	6.13E-15
**AKAP8L**	0.74	1.68	4.68E-16	6.21E-15
**PREPL**	0.67	1.59	4.86E-16	6.45E-15
**LINC01133**	-0.89	0.54	5.09E-16	6.75E-15
**CERS4**	-1.25	0.42	5.15E-16	6.81E-15
**SOX15**	-0.61	0.65	5.30E-16	7.01E-15
**TMCO3**	-0.73	0.60	5.46E-16	7.21E-15
**TNIP1**	-0.49	0.71	5.51E-16	7.28E-15
**RP11-696N14.1**	1.99	3.97	5.69E-16	7.50E-15
**PLCXD2**	2.07	4.20	5.76E-16	7.60E-15
**NIPAL3**	0.58	1.50	6.23E-16	8.21E-15
**MICALL1**	0.59	1.50	6.25E-16	8.22E-15
**LRIG1**	-1.03	0.49	6.25E-16	8.22E-15
**TFAP2C**	0.75	1.68	6.39E-16	8.40E-15
**ING5**	-1.17	0.44	6.43E-16	8.45E-15
**TSPYL2**	0.16	1.12	4.85E-02	4.43E-01
**TFAP4**	-0.15	0.90	4.86E-02	4.43E-01
**SERPINB2**	-0.09	0.94	4.86E-02	4.43E-01
**ORMDL1**	0.15	1.11	4.88E-02	4.43E-01
**09/03/2017**	0.17	1.12	4.88E-02	4.43E-01
**TRMU**	-0.14	0.91	4.88E-02	4.43E-01
**AUNIP**	-0.16	0.89	4.88E-02	4.43E-01
**BAIAP2**	-0.10	0.93	4.89E-02	4.43E-01
**FAM71D**	-0.09	0.94	4.89E-02	NA
**TMEM50A**	0.12	1.09	4.90E-02	4.44E-01
**GLT8D2**	0.07	1.05	4.91E-02	NA
**RPS6KA1**	0.10	1.07	4.91E-02	4.45E-01
**GAS2L1**	-0.13	0.92	4.92E-02	4.45E-01
**TRPT1**	0.17	1.13	4.92E-02	4.45E-01
**KRIT1**	-0.13	0.91	4.93E-02	4.45E-01
**P3H4**	-0.16	0.89	4.93E-02	4.45E-01
**PITPNB**	-0.13	0.91	4.93E-02	4.45E-01
**NAPG**	0.14	1.11	4.95E-02	4.46E-01
**STAT5B**	0.14	1.10	4.95E-02	4.46E-01
**ASB2**	-0.09	0.94	4.95E-02	NA
**STEAP1B**	-0.16	0.90	4.95E-02	NA
**PTGES3P1**	-0.12	0.92	4.96E-02	NA
**BTG1**	-0.15	0.90	4.96E-02	4.46E-01
**MAP1LC3B**	0.13	1.09	4.98E-02	4.48E-01
**UBR2**	0.13	1.10	4.99E-02	4.48E-01

**STAMBP**	0.58	1.49	6.52E-16	8.56E-15
**SEH1L**	-0.68	0.62	6.53E-16	8.57E-15
**P4HTM**	1.58	2.99	6.57E-16	8.61E-15
**STOM**	0.53	1.45	6.69E-16	8.77E-15
**NSMAF**	-0.77	0.59	6.81E-16	8.91E-15
**RRP7A**	-0.61	0.65	6.82E-16	8.92E-15
**FADS3**	1.73	3.32	7.38E-16	9.65E-15
**SLC50A1**	0.58	1.50	7.44E-16	9.72E-15
**HNRNPH3**	-0.51	0.70	7.89E-16	1.03E-14
**PGAM5**	-0.64	0.64	8.08E-16	1.05E-14
**MTFR2**	-1.34	0.39	8.99E-16	1.17E-14
**LDB1**	0.53	1.44	9.03E-16	1.18E-14
**SLC25A12**	-0.92	0.53	9.12E-16	1.19E-14
**BORA**	-1.40	0.38	9.22E-16	1.20E-14
**PPP2R2D**	0.76	1.70	9.25E-16	1.20E-14
**NSF**	0.68	1.60	9.77E-16	1.27E-14
**TONSL**	-0.77	0.59	9.78E-16	1.27E-14
**BAZ1B**	-0.58	0.67	1.01E-15	1.31E-14
**RIN1**	0.62	1.54	1.02E-15	1.32E-14
**REPS1**	-0.57	0.67	1.04E-15	1.34E-14
**WDR66**	0.66	1.59	1.05E-15	1.36E-14
**PSAP**	0.43	1.34	1.05E-15	1.36E-14
**SFPQ**	-0.48	0.72	1.07E-15	1.38E-14
**CFAP97**	-0.74	0.60	1.16E-15	1.50E-14
**MTHFD1L**	-0.57	0.68	1.16E-15	1.50E-14
**KDM4A**	0.61	1.53	1.21E-15	1.56E-14
**PITPNC1**	-0.69	0.62	1.23E-15	1.58E-14
**KRT19**	-0.63	0.65	1.23E-15	1.59E-14
**RIOK1**	-0.79	0.58	1.28E-15	1.65E-14
**ATP6V0D1**	0.52	1.43	1.29E-15	1.66E-14
**STON2**	0.86	1.81	1.30E-15	1.67E-14
**PVRL1**	-0.47	0.72	1.33E-15	1.70E-14
**TNFRSF21**	-0.51	0.70	1.36E-15	1.74E-14
**E2F2**	-1.10	0.47	1.40E-15	1.80E-14
**ETV7**	2.81	7.00	1.47E-15	1.88E-14
**DTX4**	0.86	1.82	1.51E-15	1.94E-14
**BCAS4**	0.99	1.99	1.52E-15	1.94E-14
**LPCAT4**	-0.70	0.61	1.54E-15	1.96E-14
**LINC01451**	2.61	6.10	1.57E-15	2.01E-14
**C1orf74**	0.53	1.45	1.61E-15	2.05E-14
**IMP4**	-0.63	0.65	1.62E-15	2.06E-14
**SAMD1**	-0.74	0.60	1.65E-15	2.10E-14
**HSPA12A**	1.59	3.01	1.68E-15	2.14E-14
**NONO**	-0.49	0.71	1.72E-15	2.18E-14
**CD59**	0.44	1.36	1.80E-15	2.29E-14
**USP54**	-0.84	0.56	1.89E-15	2.41E-14
**SS18**	0.51	1.42	1.94E-15	2.46E-14
**UHRF1BP1**	-0.80	0.57	2.00E-15	2.53E-14
**SOCS4**	0.76	1.70	2.04E-15	2.59E-14
**IFNGR2**	-0.59	0.67	2.05E-15	2.59E-14
**RP6-65G23.3**	1.19	2.29	2.07E-15	2.61E-14
**PEX1**	0.78	1.72	2.08E-15	2.63E-14
**ARHGAP31**	-1.17	0.44	2.18E-15	2.76E-14
**POLA1**	-0.85	0.55	2.29E-15	2.89E-14
**FGFR2**	0.70	1.63	2.42E-15	3.05E-14
**NPNT**	-0.89	0.54	2.54E-15	3.20E-14
**SPRED3**	1.57	2.97	2.55E-15	3.22E-14
**AP3B2**	2.63	6.17	2.62E-15	3.30E-14
**POLE**	-0.61	0.66	2.63E-15	3.31E-14
**SUPT6H**	0.51	1.42	2.73E-15	3.43E-14
**ZNFX1**	0.61	1.52	2.82E-15	3.55E-14
**EPB41L4A**	-1.26	0.42	2.84E-15	3.57E-14
**CASP14**	-1.86	0.28	2.86E-15	3.59E-14
**SSR3**	-0.48	0.71	2.92E-15	3.66E-14
**HIST1H2BD**	1.52	2.87	3.12E-15	3.92E-14
**FJX1**	-0.72	0.61	3.28E-15	4.11E-14
**ZNF37A**	0.78	1.71	3.38E-15	4.24E-14
**CDK5RAP2**	-0.56	0.68	3.41E-15	4.27E-14
**NALT1**	2.78	6.85	3.48E-15	4.35E-14
**MARK4**	0.60	1.51	3.50E-15	4.37E-14
**GTF3A**	-0.69	0.62	3.50E-15	4.37E-14
**HNRNPU**	-0.43	0.74	3.61E-15	4.50E-14
**SMAD5**	0.60	1.51	3.63E-15	4.52E-14
**TLDC1**	0.51	1.42	3.69E-15	4.60E-14
**PIK3CD**	0.93	1.91	3.72E-15	4.63E-14
**WDR62**	-0.78	0.58	4.07E-15	5.06E-14
**BRCA2**	-1.02	0.49	4.08E-15	5.07E-14
**GRAMD2**	1.09	2.13	4.28E-15	5.32E-14
**NOP14**	-0.60	0.66	4.56E-15	5.67E-14
**WNT5A**	-0.96	0.51	4.97E-15	6.17E-14
**CASP3**	0.69	1.62	5.02E-15	6.23E-14
**PCED1A**	1.05	2.07	5.13E-15	6.36E-14
**MMP9**	-0.77	0.59	5.14E-15	6.36E-14
**RASA1**	-0.55	0.68	5.17E-15	6.39E-14
**AC007879.7**	1.28	2.42	5.17E-15	6.39E-14
**MPHOSPH10**	-0.72	0.61	5.25E-15	6.48E-14
**POGLUT1**	0.93	1.90	5.25E-15	6.48E-14
**ZBTB2**	-0.95	0.52	5.94E-15	7.32E-14
**ICK**	-1.13	0.46	6.11E-15	7.53E-14
**ZNF561-AS1**	1.44	2.71	6.20E-15	7.64E-14

**FIGNL1**	-0.84	0.56	6.27E-15	7.72E-14
**RPUSD1**	-0.83	0.56	6.45E-15	7.93E-14
**TMEM63C**	-0.91	0.53	6.51E-15	8.00E-14
**APLP1**	2.29	4.88	6.52E-15	8.00E-14
**PPA1**	-0.60	0.66	6.53E-15	8.02E-14
**PATZ1**	-1.10	0.47	6.58E-15	8.07E-14
**WDR82**	-0.46	0.73	6.86E-15	8.40E-14
**ZNF207**	-0.53	0.69	7.01E-15	8.58E-14
**RP11-132A1.4**	-1.64	0.32	7.27E-15	8.90E-14
**DEPDC7**	0.83	1.78	7.78E-15	9.52E-14
**MAL**	-1.38	0.38	7.89E-15	9.65E-14
**FAM72B**	-2.39	0.19	8.02E-15	9.79E-14
**TBCEL**	1.03	2.04	8.23E-15	1.00E-13
**ATP6V0A1**	0.65	1.57	8.45E-15	1.03E-13
**MYO1D**	-0.51	0.70	8.97E-15	1.09E-13
**UTP18**	-0.77	0.59	9.05E-15	1.10E-13
**CHST2**	2.05	4.13	9.07E-15	1.10E-13
**CAST**	-0.47	0.72	9.30E-15	1.13E-13
**FAM169A**	0.81	1.76	9.59E-15	1.17E-13
**NUP160**	-0.57	0.67	9.70E-15	1.18E-13
**AK4**	-0.63	0.64	1.05E-14	1.27E-13
**TUBA1B**	-0.61	0.66	1.07E-14	1.30E-13
**ERO1A**	-0.65	0.64	1.08E-14	1.31E-13
**RHOC**	0.57	1.48	1.09E-14	1.32E-13
**RP11-44F14.10**	2.90	7.47	1.09E-14	1.32E-13
**TSPAN10**	2.27	4.84	1.14E-14	1.38E-13
**SBF2**	-0.75	0.60	1.15E-14	1.39E-13
**NEDD4**	-0.69	0.62	1.16E-14	1.40E-13
**ACP5**	1.14	2.21	1.17E-14	1.42E-13
**PITPNM1**	0.60	1.51	1.20E-14	1.44E-13
**TMED4**	0.57	1.48	1.26E-14	1.52E-13
**ARMCX3**	0.86	1.82	1.27E-14	1.53E-13
**STRA13**	-0.77	0.59	1.27E-14	1.53E-13
**GLDC**	0.96	1.95	1.30E-14	1.56E-13
**SERPINB2**	0.45	1.37	1.30E-14	1.57E-13
**HCAR2**	0.98	1.97	1.31E-14	1.58E-13
**CSTF3**	0.68	1.60	1.34E-14	1.61E-13
**PER3**	-1.31	0.40	1.36E-14	1.63E-13
**RALGPS1**	1.47	2.77	1.36E-14	1.63E-13
**PRR12**	-0.65	0.64	1.37E-14	1.64E-13
**MYO1C**	-0.51	0.70	1.40E-14	1.68E-13
**PTTG1**	-0.51	0.70	1.41E-14	1.69E-13
**ADCK3**	1.21	2.32	1.55E-14	1.86E-13
**KLF10**	0.51	1.42	1.60E-14	1.91E-13
**CCM2**	0.61	1.53	1.67E-14	2.00E-13
**PTK2B**	0.57	1.48	1.69E-14	2.01E-13
**APEX1**	-0.50	0.71	1.78E-14	2.12E-13
**AGO2**	-0.67	0.63	1.83E-14	2.18E-13
**AAK1**	0.66	1.58	1.84E-14	2.20E-13
**PUSL1**	-0.97	0.51	1.88E-14	2.23E-13
**CASD1**	1.26	2.39	1.90E-14	2.26E-13
**HES2**	0.44	1.36	1.91E-14	2.27E-13
**ICE2**	-0.74	0.60	1.93E-14	2.30E-13
**LRRC8C**	-0.76	0.59	1.97E-14	2.34E-13
**GAS2L1**	-0.68	0.62	2.00E-14	2.38E-13
**AK3**	0.62	1.54	2.02E-14	2.40E-13
**ANAPC2**	0.76	1.69	2.03E-14	2.40E-13
**LTB4R**	-0.63	0.64	2.05E-14	2.42E-13
**TANC1**	-0.50	0.71	2.05E-14	2.42E-13
**CTBP2**	-0.58	0.67	2.05E-14	2.43E-13
**TGFB1I1**	-1.32	0.40	2.10E-14	2.48E-13
**DDX60L**	0.94	1.92	2.13E-14	2.52E-13
**LRRC59**	-0.42	0.75	2.16E-14	2.55E-13
**CBX2**	-0.96	0.51	2.22E-14	2.62E-13
**C15orf52**	0.66	1.58	2.23E-14	2.62E-13
**MLST8**	-0.71	0.61	2.24E-14	2.63E-13
**UBR1**	0.61	1.53	2.30E-14	2.71E-13
**AUTS2**	-0.75	0.60	2.39E-14	2.81E-13
**FAM219A**	0.76	1.70	2.43E-14	2.85E-13
**WBP11**	-0.50	0.71	2.47E-14	2.90E-13
**RP11-21B23.2**	2.01	4.02	2.48E-14	2.92E-13
**SMYD2**	-0.65	0.64	2.49E-14	2.92E-13
**PCBP1**	-0.54	0.69	2.54E-14	2.97E-13
**MTMR12**	-0.64	0.64	2.56E-14	3.00E-13
**ACTL6A**	-0.61	0.66	2.59E-14	3.03E-13
**NSMCE4A**	-0.90	0.54	2.63E-14	3.08E-13
**ARRB2**	-0.79	0.58	2.64E-14	3.09E-13
**USP9Y**	0.67	1.59	2.77E-14	3.24E-13
**ITPKB**	0.72	1.65	2.79E-14	3.26E-13
**AMOT**	0.92	1.89	2.82E-14	3.29E-13
**LYSMD3**	0.86	1.82	2.91E-14	3.39E-13
**AGRN**	0.49	1.41	3.06E-14	3.57E-13
**IFT27**	1.15	2.22	3.10E-14	3.61E-13
**BOD1**	-0.74	0.60	3.24E-14	3.77E-13
**ARRDC2**	1.19	2.28	3.25E-14	3.78E-13
**RP11-253E3.3**	-1.59	0.33	3.33E-14	3.87E-13
**SRSF8**	0.65	1.57	3.35E-14	3.88E-13
**BCL2L12**	-0.86	0.55	3.38E-14	3.92E-13
**GATA3**	0.63	1.54	3.41E-14	3.95E-13
**TIFA**	-1.43	0.37	3.45E-14	4.00E-13

**FBXL16**	-2.39	0.19	3.50E-14	4.06E-13
**NEK6**	-0.68	0.63	3.54E-14	4.10E-13
**SMARCA1**	0.85	1.81	3.85E-14	4.45E-13
**RAD23A**	-0.63	0.65	3.88E-14	4.48E-13
**KIAA1257**	2.61	6.12	3.94E-14	4.55E-13
**RFWD3**	-0.66	0.63	3.98E-14	4.59E-13
**MYO6**	0.56	1.47	4.00E-14	4.62E-13
**CDK2**	-0.66	0.63	4.20E-14	4.84E-13
**TMEM79**	0.49	1.40	4.20E-14	4.84E-13
**PIM3**	-0.62	0.65	4.47E-14	5.15E-13
**TYMS**	0.70	1.62	4.49E-14	5.17E-13
**WDR18**	-0.63	0.65	4.62E-14	5.31E-13
**PHLPP1**	-0.77	0.59	4.73E-14	5.43E-13
**DARS2**	-0.57	0.67	4.85E-14	5.57E-13
**CHST14**	1.03	2.04	5.03E-14	5.78E-13
**THSD1**	0.85	1.81	5.09E-14	5.84E-13
**C3**	1.04	2.05	5.12E-14	5.87E-13
**EPHA4**	1.23	2.34	5.16E-14	5.91E-13
**MRPL33**	0.75	1.68	5.19E-14	5.94E-13
**HAS2**	-2.28	0.21	5.42E-14	6.20E-13
**AC006262.5**	2.22	4.66	5.45E-14	6.23E-13
**TRAF2**	-0.90	0.54	5.56E-14	6.35E-13
**NAPA**	0.65	1.56	5.64E-14	6.44E-13
**TMEM59**	0.57	1.48	5.68E-14	6.48E-13
**SAAL1**	-0.79	0.58	5.89E-14	6.71E-13
**AXIN1**	-0.66	0.63	5.90E-14	6.72E-13
**ERF**	-0.52	0.70	6.06E-14	6.90E-13
**TRUB1**	0.76	1.70	6.09E-14	6.93E-13
**GSTP1**	0.45	1.37	6.39E-14	7.27E-13
**TSEN15**	-0.75	0.59	6.45E-14	7.33E-13
**VKORC1L1**	-0.61	0.65	6.47E-14	7.35E-13
**MFAP2**	-0.79	0.58	6.61E-14	7.50E-13
**SLX4**	-0.91	0.53	6.62E-14	7.51E-13
**IGFL2**	0.64	1.56	6.64E-14	7.53E-13
**FAM171B**	1.56	2.94	6.70E-14	7.59E-13
**TBC1D4**	-0.73	0.60	6.80E-14	7.70E-13
**ARF6**	-0.43	0.74	6.83E-14	7.73E-13
**AAAS**	-0.63	0.65	6.84E-14	7.73E-13
**DHRS3**	0.68	1.61	6.84E-14	7.73E-13
**TTLL3**	1.57	2.96	6.85E-14	7.74E-13
**KLK7**	-0.44	0.73	7.07E-14	7.98E-13
**STARD10**	0.60	1.52	7.14E-14	8.06E-13
**SNHG14**	0.73	1.66	7.20E-14	8.12E-13
**ITGAM**	2.90	7.48	7.21E-14	8.13E-13
**RASSF10**	1.04	2.06	7.23E-14	8.13E-13
**NLE1**	-0.78	0.58	7.26E-14	8.16E-13
**ARFGAP1**	0.63	1.54	7.30E-14	8.21E-13
**OTUB2**	1.30	2.46	7.36E-14	8.27E-13
**GYS1**	0.45	1.36	7.42E-14	8.33E-13
**AHNAK2**	-0.54	0.69	7.49E-14	8.41E-13
**GYLTL1B**	-1.08	0.47	7.79E-14	8.73E-13
**RFC4**	-0.84	0.56	7.88E-14	8.84E-13
**ACTB**	-0.46	0.73	7.93E-14	8.88E-13
**PRDX6**	0.41	1.33	7.98E-14	8.93E-13
**COPS6**	0.48	1.40	8.08E-14	9.04E-13
**MRPS6**	0.74	1.67	8.18E-14	9.14E-13
**NOL4L**	2.32	5.01	8.45E-14	9.44E-13
**MSN**	-0.41	0.75	8.47E-14	9.46E-13
**IGF2BP2**	-0.44	0.74	8.49E-14	9.47E-13
**LRRCC1**	-1.04	0.49	8.56E-14	9.54E-13
**G3BP1**	-0.47	0.72	8.62E-14	9.60E-13
**LSP1**	-1.23	0.43	8.65E-14	9.64E-13
**ERCC6L**	-0.85	0.55	8.75E-14	9.74E-13
**CTB-193M12.5**	-1.34	0.40	8.84E-14	9.83E-13
**CTDSPL**	-0.48	0.71	8.85E-14	9.84E-13
**ASB16-AS1**	1.42	2.67	9.00E-14	1.00E-12
**HMCES**	-0.60	0.66	9.41E-14	1.04E-12
**TMEM263**	0.97	1.96	9.64E-14	1.07E-12
**SETD5**	-0.49	0.71	9.81E-14	1.09E-12
**ISG20L2**	-0.63	0.65	9.87E-14	1.09E-12
**F11R**	0.54	1.45	1.04E-13	1.15E-12
**PRKCD**	0.58	1.50	1.09E-13	1.21E-12
**POMT1**	-0.87	0.55	1.13E-13	1.25E-12
**CSRNP1**	0.86	1.82	1.16E-13	1.28E-12
**CDC42BPG**	0.54	1.46	1.16E-13	1.28E-12
**NUAK2**	0.64	1.56	1.17E-13	1.29E-12
**APOBEC3D**	2.87	7.31	1.27E-13	1.40E-12
**TLE1**	-0.69	0.62	1.27E-13	1.40E-12
**TMEM175**	0.88	1.84	1.27E-13	1.40E-12
**TEX9**	1.70	3.26	1.30E-13	1.43E-12
**PDGFC**	-0.89	0.54	1.33E-13	1.47E-12
**MOB1B**	0.71	1.64	1.39E-13	1.53E-12
**APOOL**	0.70	1.62	1.40E-13	1.54E-12
**VPS72**	-0.58	0.67	1.41E-13	1.55E-12
**MAFB**	1.00	2.00	1.42E-13	1.55E-12
**RNF145**	-0.59	0.66	1.44E-13	1.58E-12
**PRSS16**	-1.46	0.36	1.45E-13	1.59E-12
**FAM57A**	-0.70	0.62	1.46E-13	1.60E-12
**TNFSF10**	1.12	2.18	1.46E-13	1.60E-12
**OSBPL5**	-0.86	0.55	1.49E-13	1.63E-12

**SCRIB**	0.50	1.42	1.53E-13	1.67E-12
**TPP2**	0.54	1.45	1.54E-13	1.68E-12
**KRT1**	-0.72	0.61	1.54E-13	1.68E-12
**MYO3B**	1.31	2.48	1.60E-13	1.75E-12
**ALMS1**	-0.89	0.54	1.62E-13	1.77E-12
**RASSF1**	-0.90	0.54	1.65E-13	1.80E-12
**CDC6**	-0.60	0.66	1.69E-13	1.84E-12
**AFAP1L2**	-0.62	0.65	1.69E-13	1.84E-12
**NBL1**	-1.06	0.48	1.69E-13	1.84E-12
**KLF16**	-0.76	0.59	1.72E-13	1.87E-12
**NOP58**	-0.54	0.69	1.75E-13	1.90E-12
**RHOB**	0.63	1.54	1.82E-13	1.98E-12
**CLSPN**	-0.88	0.54	1.83E-13	1.99E-12
**SH3PXD2A**	-0.44	0.74	1.83E-13	1.99E-12
**ZC3H7A**	0.57	1.48	1.90E-13	2.06E-12
**ADCY3**	-0.64	0.64	1.90E-13	2.06E-12
**CACYBP**	-0.59	0.66	1.95E-13	2.11E-12
**MUM1**	-0.92	0.53	2.01E-13	2.17E-12
**DDX39B**	0.73	1.66	2.03E-13	2.20E-12
**PMAIP1**	0.93	1.91	2.05E-13	2.22E-12
**MARVELD1**	0.48	1.40	2.08E-13	2.24E-12
**TSR1**	-0.49	0.71	2.11E-13	2.28E-12
**FAM13C**	2.90	7.46	2.15E-13	2.32E-12
**UBB**	0.61	1.53	2.16E-13	2.32E-12
**FOXO1**	0.68	1.60	2.19E-13	2.36E-12
**ADIPOR1**	0.41	1.33	2.20E-13	2.37E-12
**PLD3**	0.53	1.44	2.20E-13	2.37E-12
**SACS**	-0.66	0.63	2.29E-13	2.46E-12
**SETD8**	-0.68	0.62	2.36E-13	2.54E-12
**VASP**	-0.46	0.73	2.38E-13	2.56E-12
**GSTZ1**	0.81	1.76	2.39E-13	2.56E-12
**MTFR1L**	0.78	1.72	2.53E-13	2.72E-12
**CXCR2**	1.74	3.35	2.61E-13	2.80E-12
**C18orf54**	-1.04	0.48	2.65E-13	2.84E-12
**NAA15**	-0.50	0.71	2.69E-13	2.88E-12
**CHAF1A**	-0.69	0.62	2.74E-13	2.94E-12
**HLA-B**	0.65	1.57	2.78E-13	2.98E-12
**ABCA5**	1.14	2.20	2.84E-13	3.04E-12
**KIRREL**	0.47	1.39	2.85E-13	3.05E-12
**NLGN2**	0.96	1.95	2.94E-13	3.14E-12
**RP2**	0.90	1.86	2.95E-13	3.14E-12
**CGN**	0.62	1.54	2.96E-13	3.16E-12
**C6orf89**	0.51	1.43	2.96E-13	3.16E-12
**G0S2**	0.95	1.94	3.04E-13	3.24E-12
**CCDC130**	0.81	1.76	3.05E-13	3.24E-12
**C22orf23**	1.31	2.49	3.22E-13	3.43E-12
**FAM210B**	0.73	1.66	3.28E-13	3.49E-12
**IL20RA**	-0.82	0.57	3.31E-13	3.52E-12
**MSX2**	1.20	2.29	3.32E-13	3.53E-12
**LPAR2**	-0.96	0.52	3.44E-13	3.66E-12
**TMEM27**	2.43	5.39	3.59E-13	3.81E-12
**SMAP2**	0.55	1.47	3.60E-13	3.81E-12
**UROD**	0.51	1.42	3.67E-13	3.89E-12
**MMP10**	-1.48	0.36	3.69E-13	3.91E-12
**MPHOSPH9**	-0.95	0.52	3.74E-13	3.96E-12
**ZNF672**	0.64	1.56	3.79E-13	4.01E-12
**GPAT3**	-1.23	0.43	3.87E-13	4.09E-12
**CTD-2003C8.2**	2.60	6.07	3.94E-13	4.16E-12
**SEC24D**	-0.61	0.65	4.12E-13	4.35E-12
**TRPT1**	1.03	2.04	4.20E-13	4.43E-12
**SLC25A5**	0.37	1.30	4.27E-13	4.51E-12
**TOMM20**	-0.49	0.71	4.28E-13	4.51E-12
**DSCAM**	-1.16	0.45	4.29E-13	4.52E-12
**MRPL17**	-0.58	0.67	4.30E-13	4.53E-12
**PGP**	-0.76	0.59	4.31E-13	4.54E-12
**IL13RA1**	0.59	1.51	4.36E-13	4.59E-12
**TRANK1**	1.41	2.65	4.46E-13	4.68E-12
**PPM1J**	1.39	2.62	4.47E-13	4.69E-12
**MRPS18B**	-0.56	0.68	4.65E-13	4.88E-12
**WDR3**	-0.57	0.67	4.66E-13	4.89E-12
**AKAP13**	-0.59	0.66	4.82E-13	5.05E-12
**MAN1C1**	2.74	6.67	4.89E-13	5.13E-12
**MAST3**	1.12	2.18	5.13E-13	5.37E-12
**CA11**	1.71	3.27	5.17E-13	5.41E-12
**SYNM**	-1.06	0.48	5.29E-13	5.53E-12
**HSPA9**	-0.40	0.76	5.39E-13	5.63E-12
**PCNT**	-0.63	0.64	5.42E-13	5.67E-12
**SCNN1A**	0.74	1.67	5.44E-13	5.68E-12
**KCMF1**	-0.51	0.70	5.52E-13	5.76E-12
**ALDH3B2**	-0.66	0.63	5.65E-13	5.89E-12
**MAL2**	-0.49	0.71	5.68E-13	5.92E-12
**RPS19**	0.40	1.32	5.84E-13	6.08E-12
**ERRFI1**	-0.54	0.69	5.89E-13	6.13E-12
**PRPS1**	-0.74	0.60	6.06E-13	6.30E-12
**PPFIBP2**	-0.89	0.54	6.12E-13	6.36E-12
**PIFO**	1.96	3.88	6.13E-13	6.37E-12
**NEIL3**	-1.04	0.49	6.15E-13	6.39E-12
**MIS18A**	-0.81	0.57	6.22E-13	6.45E-12
**SBF1**	0.51	1.42	6.22E-13	6.45E-12
**FAM83D**	-0.56	0.68	6.50E-13	6.74E-12

**GMNN**	-0.62	0.65	6.63E-13	6.87E-12
**ZBED3**	-1.59	0.33	6.64E-13	6.88E-12
**PRR15**	-2.10	0.23	6.73E-13	6.97E-12
**FANCG**	-0.68	0.62	6.77E-13	7.00E-12
**NBN**	-0.74	0.60	6.86E-13	7.09E-12
**NID2**	1.61	3.06	6.88E-13	7.10E-12
**TMEM45B**	-0.72	0.61	7.00E-13	7.22E-12
**TMEM127**	0.49	1.41	7.02E-13	7.24E-12
**RRM2**	-0.54	0.69	7.20E-13	7.43E-12
**FAM104A**	0.73	1.66	7.37E-13	7.60E-12
**SNAI2**	0.46	1.37	7.38E-13	7.60E-12
**TIMM13**	-0.57	0.67	7.42E-13	7.64E-12
**MPRIP**	0.41	1.33	7.70E-13	7.92E-12
**ULBP2**	0.67	1.59	7.87E-13	8.09E-12
**S1PR3**	1.47	2.76	7.96E-13	8.18E-12
**MORC4**	0.59	1.50	8.03E-13	8.24E-12
**TCF20**	-0.47	0.72	8.06E-13	8.27E-12
**SLC24A1**	-0.89	0.54	8.24E-13	8.45E-12
**ARHGAP11B**	-1.09	0.47	8.35E-13	8.56E-12
**FEN1**	-0.56	0.68	8.37E-13	8.58E-12
**SLC35G1**	-1.22	0.43	8.42E-13	8.62E-12
**THAP11**	-0.87	0.55	8.49E-13	8.68E-12
**CECR5**	-0.70	0.62	8.51E-13	8.71E-12
**STK24**	-0.44	0.74	8.91E-13	9.11E-12
**DLL1**	-1.31	0.40	9.68E-13	9.89E-12
**TIMP1**	0.55	1.47	9.89E-13	1.01E-11
**RPS4X**	0.34	1.26	1.02E-12	1.04E-11
**CIZ1**	-0.45	0.73	1.03E-12	1.05E-11
**SPC25**	-1.09	0.47	1.06E-12	1.08E-11
**MOB3C**	0.79	1.73	1.07E-12	1.09E-11
**KRTCAP3**	-1.14	0.45	1.07E-12	1.09E-11
**ARHGEF10**	-0.78	0.58	1.07E-12	1.09E-11
**ADGRF1**	-0.63	0.65	1.08E-12	1.10E-11
**PAPD7**	-0.66	0.63	1.08E-12	1.10E-11
**HTATIP2**	0.64	1.56	1.08E-12	1.10E-11
**LDHA**	-0.36	0.78	1.12E-12	1.13E-11
**USP7**	-0.44	0.74	1.12E-12	1.14E-11
**UTP14A**	-0.67	0.63	1.16E-12	1.17E-11
**INSIG1**	-1.29	0.41	1.18E-12	1.19E-11
**DIS3L**	-0.96	0.51	1.19E-12	1.21E-11
**FRMD6**	0.48	1.40	1.27E-12	1.29E-11
**VCL**	0.38	1.31	1.29E-12	1.31E-11
**AES**	-0.47	0.72	1.30E-12	1.31E-11
**SCMH1**	-0.84	0.56	1.30E-12	1.31E-11
**F3**	0.41	1.33	1.30E-12	1.31E-11
**OIP5-AS1**	-0.51	0.70	1.32E-12	1.33E-11
**CAMSAP1**	0.53	1.44	1.33E-12	1.34E-11
**RNPS1**	-0.52	0.70	1.33E-12	1.34E-11
**NEFH**	0.91	1.87	1.34E-12	1.35E-11
**GABARAPL2**	0.69	1.61	1.36E-12	1.37E-11
**BMP1**	0.60	1.51	1.37E-12	1.38E-11
**PIK3R1**	-0.62	0.65	1.42E-12	1.43E-11
**DICER1**	-0.51	0.70	1.54E-12	1.55E-11
**ACO1**	0.59	1.51	1.57E-12	1.58E-11
**SLC5A1**	-0.82	0.56	1.60E-12	1.60E-11
**TTC27**	-0.68	0.63	1.60E-12	1.61E-11
**MED12**	0.59	1.50	1.68E-12	1.68E-11
**EAF1**	0.72	1.65	1.72E-12	1.73E-11
**PRKAR1A**	0.48	1.39	1.73E-12	1.73E-11
**RNASEH2B**	-0.95	0.52	1.74E-12	1.74E-11
**BTBD2**	-0.57	0.68	1.76E-12	1.76E-11
**FLJ26850**	2.76	6.77	1.76E-12	1.76E-11
**CEBPG**	-0.56	0.68	1.89E-12	1.89E-11
**DOCK4**	-0.76	0.59	1.89E-12	1.89E-11
**LPCAT3**	0.94	1.92	1.92E-12	1.92E-11
**POLRMT**	-0.65	0.64	1.96E-12	1.96E-11
**LITAF**	0.44	1.36	1.96E-12	1.96E-11
**RAC1**	-0.40	0.76	1.99E-12	1.99E-11
**ZMIZ1**	-0.54	0.69	2.08E-12	2.07E-11
**FARP2**	-0.54	0.69	2.11E-12	2.10E-11
**STEAP1**	-0.90	0.54	2.14E-12	2.13E-11
**RASIP1**	-0.72	0.61	2.17E-12	2.16E-11
**CCNE2**	-1.56	0.34	2.19E-12	2.18E-11
**DOCK11**	-0.93	0.53	2.21E-12	2.20E-11
**DNAJB4**	0.92	1.89	2.22E-12	2.20E-11
**PABPC4**	-0.41	0.75	2.23E-12	2.21E-11
**EDNRA**	-1.55	0.34	2.27E-12	2.25E-11
**SLC41A1**	0.54	1.46	2.28E-12	2.26E-11
**ADCY1**	1.92	3.79	2.29E-12	2.26E-11
**NOMO1**	0.55	1.46	2.30E-12	2.27E-11
**FBXO27**	-0.74	0.60	2.37E-12	2.34E-11
**USP20**	0.73	1.66	2.38E-12	2.35E-11
**SCD**	-1.22	0.43	2.38E-12	2.36E-11
**SLC35B2**	-0.56	0.68	2.41E-12	2.38E-11
**TTL**	-0.53	0.69	2.42E-12	2.39E-11
**SLC16A9**	-0.91	0.53	2.45E-12	2.41E-11
**EGLN3**	0.90	1.86	2.47E-12	2.44E-11
**TRPV1**	1.64	3.13	2.48E-12	2.45E-11
**LSM14A**	-0.45	0.73	2.52E-12	2.49E-11
**DDX5**	0.35	1.28	2.53E-12	2.50E-11

**IGF2BP3**	-0.60	0.66	2.55E-12	2.51E-11
**ZNF146**	-0.53	0.69	2.64E-12	2.60E-11
**DDX39A**	-0.47	0.72	2.77E-12	2.73E-11
**ZBTB5**	0.66	1.58	2.79E-12	2.74E-11
**SYNJ1**	0.79	1.73	2.82E-12	2.77E-11
**SURF1**	0.87	1.83	2.85E-12	2.80E-11
**GEMIN4**	-0.64	0.64	2.92E-12	2.86E-11
**FAM111A**	-0.75	0.59	2.92E-12	2.86E-11
**PUF60**	-0.48	0.72	2.93E-12	2.87E-11
**ZBED4**	-0.58	0.67	2.98E-12	2.92E-11
**RANBP10**	0.60	1.52	3.02E-12	2.95E-11
**MTND6P4**	0.98	1.97	3.05E-12	2.99E-11
**SLC44A1**	-0.56	0.68	3.14E-12	3.07E-11
**UPK3B**	1.07	2.09	3.18E-12	3.11E-11
**FANCD2**	-0.67	0.63	3.23E-12	3.16E-11
**ABCA7**	0.63	1.55	3.28E-12	3.20E-11
**DNMT1**	-0.48	0.71	3.29E-12	3.21E-11
**CAMKK2**	-0.58	0.67	3.31E-12	3.23E-11
**WFDC5**	-1.71	0.31	3.36E-12	3.27E-11
**SPATS2**	-0.58	0.67	3.38E-12	3.29E-11
**DEGS1**	-0.51	0.70	3.44E-12	3.35E-11
**CENPO**	-0.86	0.55	3.48E-12	3.39E-11
**NOP56**	-0.57	0.67	3.59E-12	3.49E-11
**VPS39**	0.50	1.41	3.74E-12	3.64E-11
**DNAJC18**	1.17	2.25	3.78E-12	3.67E-11
**KIF21B**	-2.04	0.24	3.81E-12	3.70E-11
**KALRN**	0.95	1.93	3.84E-12	3.73E-11
**CLCA2**	0.45	1.37	3.92E-12	3.80E-11
**RARA**	0.66	1.57	3.98E-12	3.85E-11
**MKLN1**	0.67	1.59	4.08E-12	3.95E-11
**PDIA6**	-0.44	0.74	4.09E-12	3.96E-11
**MOSPD1**	0.91	1.88	4.16E-12	4.03E-11
**TRMT6**	-0.61	0.65	4.18E-12	4.04E-11
**UNC93B1**	0.88	1.84	4.23E-12	4.09E-11
**EPB41L5**	0.60	1.52	4.25E-12	4.10E-11
**SRSF5**	0.49	1.41	4.32E-12	4.17E-11
**UBXN11**	0.93	1.91	4.42E-12	4.26E-11
**GABPB1**	-0.87	0.55	4.48E-12	4.32E-11
**DDHD2**	-0.57	0.68	4.58E-12	4.41E-11
**LAMB3**	0.47	1.38	4.59E-12	4.42E-11
**ADAMTS14**	-1.75	0.30	4.59E-12	4.42E-11
**RPL12**	-0.34	0.79	4.64E-12	4.47E-11
**TMBIM1**	0.38	1.30	4.68E-12	4.50E-11
**ABCD1**	1.58	3.00	4.88E-12	4.69E-11
**MT-ND3**	0.53	1.44	4.88E-12	4.69E-11
**ABCG4**	1.87	3.64	4.97E-12	4.77E-11
**CTNNBIP1**	0.47	1.39	5.00E-12	4.80E-11
**RNF26**	-0.67	0.63	5.04E-12	4.83E-11
**DGKZ**	0.49	1.41	5.10E-12	4.88E-11
**IMPDH1**	-0.51	0.70	5.11E-12	4.89E-11
**HLA-C**	0.40	1.32	5.13E-12	4.91E-11
**RP11-643A5.3**	2.83	7.11	5.37E-12	5.13E-11
**AHSA2**	1.07	2.11	5.55E-12	5.31E-11
**KHK**	-1.84	0.28	5.58E-12	5.33E-11
**SCARB2**	0.49	1.40	5.61E-12	5.36E-11
**NXT1**	-0.86	0.55	5.63E-12	5.37E-11
**PRPSAP2**	0.67	1.59	5.63E-12	5.37E-11
**ABCE1**	-0.51	0.70	5.63E-12	5.37E-11
**CXCL14**	0.82	1.77	5.72E-12	5.45E-11
**DDX51**	-0.74	0.60	5.73E-12	5.46E-11
**RANBP1**	-0.49	0.71	5.76E-12	5.48E-11
**WDR26**	0.48	1.39	6.10E-12	5.81E-11
**TRIM65**	-0.78	0.58	6.38E-12	6.06E-11
**OGG1**	-0.83	0.56	6.39E-12	6.07E-11
**MGRN1**	0.67	1.59	6.61E-12	6.28E-11
**SYNGR2**	-0.53	0.69	6.65E-12	6.31E-11
**ADI1**	0.46	1.37	6.68E-12	6.34E-11
**PUS1**	-0.79	0.58	6.76E-12	6.41E-11
**TIMM50**	-0.46	0.73	6.91E-12	6.55E-11
**CCDC61**	-1.03	0.49	6.91E-12	6.55E-11
**SASS6**	-0.90	0.54	6.99E-12	6.62E-11
**PPRC1**	-1.02	0.49	7.03E-12	6.66E-11
**FAM136A**	-0.56	0.68	7.05E-12	6.67E-11
**RUSC2**	0.79	1.73	7.24E-12	6.84E-11
**CDKN1C**	-1.29	0.41	7.34E-12	6.93E-11
**KCTD10**	0.45	1.37	7.34E-12	6.93E-11
**SERINC1**	0.54	1.45	7.44E-12	7.03E-11
**NCKAP1**	-0.45	0.73	7.49E-12	7.07E-11
**DCLRE1B**	-0.75	0.59	7.72E-12	7.28E-11
**OTP**	2.81	7.02	7.73E-12	7.29E-11
**SDC3**	-0.52	0.70	7.91E-12	7.45E-11
**PLSCR1**	0.49	1.40	7.93E-12	7.46E-11
**ERO1B**	1.90	3.74	8.04E-12	7.57E-11
**CENPC**	-0.96	0.51	8.39E-12	7.90E-11
**NOB1**	-0.60	0.66	8.52E-12	8.01E-11
**PRPF19**	-0.47	0.72	8.62E-12	8.10E-11
**USP18**	1.41	2.66	8.76E-12	8.22E-11
**YWHAE**	-0.35	0.79	8.98E-12	8.43E-11
**GCAT**	-0.79	0.58	9.00E-12	8.44E-11
**TMEM198B**	1.30	2.47	9.08E-12	8.51E-11

**GFM2**	0.64	1.56	9.14E-12	8.56E-11
**KIAA1549**	-1.05	0.48	9.22E-12	8.64E-11
**NOD2**	-1.07	0.48	9.33E-12	8.74E-11
**SIPA1L2**	0.63	1.55	9.58E-12	8.96E-11
**ZFPM1**	1.01	2.02	9.63E-12	9.01E-11
**SIKE1**	0.61	1.53	9.74E-12	9.10E-11
**GRINA**	0.51	1.42	9.94E-12	9.29E-11
**ANKRD29**	1.00	1.99	1.01E-11	9.41E-11
**OSTC**	-0.66	0.63	1.02E-11	9.55E-11
**DCAF16**	-0.78	0.58	1.06E-11	9.87E-11
**ADD3**	0.60	1.52	1.06E-11	9.92E-11
**05-sep**	-1.11	0.46	1.07E-11	9.92E-11
**KLHL17**	-0.90	0.54	1.09E-11	1.02E-10
**PSKH1**	0.65	1.57	1.10E-11	1.02E-10
**CENPU**	-0.67	0.63	1.10E-11	1.02E-10
**NOCT**	1.01	2.01	1.14E-11	1.06E-10
**KANK1**	-0.43	0.74	1.15E-11	1.07E-10
**PROM2**	0.45	1.36	1.18E-11	1.09E-10
**NOC3L**	-0.67	0.63	1.18E-11	1.10E-10
**RTN4**	-0.36	0.78	1.23E-11	1.14E-10
**GJB5**	-0.50	0.71	1.26E-11	1.17E-10
**GPR180**	-0.72	0.61	1.28E-11	1.19E-10
**IL1RAP**	-0.46	0.73	1.29E-11	1.19E-10
**LRRC45**	-0.85	0.55	1.30E-11	1.20E-10
**EXOSC10**	-0.45	0.73	1.31E-11	1.21E-10
**DDB1**	0.36	1.28	1.32E-11	1.23E-10
**B3GAT3**	0.77	1.70	1.33E-11	1.23E-10
**PYHIN1**	2.68	6.42	1.34E-11	1.24E-10
**TMCC2**	1.48	2.80	1.34E-11	1.24E-10
**IRX4**	-0.66	0.63	1.36E-11	1.26E-10
**CLPTM1**	0.48	1.40	1.37E-11	1.26E-10
**LMF2**	0.57	1.48	1.39E-11	1.29E-10
**RSRC1**	-0.79	0.58	1.40E-11	1.29E-10
**NFKBIE**	0.94	1.92	1.41E-11	1.30E-10
**BSDC1**	0.50	1.42	1.47E-11	1.35E-10
**GALNT3**	-0.53	0.69	1.49E-11	1.37E-10
**EPHX2**	1.16	2.23	1.53E-11	1.40E-10
**ATP6V1F**	0.57	1.48	1.56E-11	1.43E-10
**GEMIN2**	-1.02	0.49	1.56E-11	1.43E-10
**PRRG2**	1.03	2.04	1.57E-11	1.44E-10
**HMGCR**	-0.57	0.67	1.57E-11	1.44E-10
**SLC9A7**	0.66	1.58	1.57E-11	1.44E-10
**TP53I11**	0.65	1.57	1.57E-11	1.44E-10
**MLLT10**	-0.62	0.65	1.58E-11	1.45E-10
**CASP8AP2**	-0.74	0.60	1.58E-11	1.45E-10
**GADD45B**	0.79	1.73	1.64E-11	1.50E-10
**ABHD8**	1.10	2.14	1.66E-11	1.52E-10
**KLHL12**	0.59	1.51	1.72E-11	1.57E-10
**INF2**	-0.53	0.69	1.73E-11	1.58E-10
**SGCB**	0.65	1.57	1.74E-11	1.59E-10
**RGAG4**	2.65	6.30	1.74E-11	1.59E-10
**RAD51D**	-1.19	0.44	1.75E-11	1.60E-10
**SEC63**	-0.49	0.71	1.75E-11	1.60E-10
**CPPED1**	0.65	1.57	1.77E-11	1.61E-10
**SHC1**	0.40	1.32	1.77E-11	1.62E-10
**ATP6AP2**	0.51	1.42	1.79E-11	1.63E-10
**MRAS**	1.56	2.94	1.85E-11	1.68E-10
**C14orf80**	-0.93	0.53	1.88E-11	1.71E-10
**POLR3K**	-0.91	0.53	1.90E-11	1.73E-10
**NUDT21**	-0.46	0.73	1.92E-11	1.75E-10
**IGFL1**	0.66	1.58	1.97E-11	1.79E-10
**CYLD**	0.68	1.60	2.00E-11	1.82E-10
**MCTP2**	-0.88	0.55	2.01E-11	1.82E-10
**VANGL1**	-0.45	0.73	2.01E-11	1.83E-10
**PRSS3**	-0.58	0.67	2.02E-11	1.83E-10
**MAPK8IP3**	0.62	1.54	2.05E-11	1.86E-10
**C9orf142**	-0.80	0.58	2.06E-11	1.86E-10
**FUT2**	-1.02	0.49	2.08E-11	1.88E-10
**ABCD3**	0.64	1.56	2.09E-11	1.89E-10
**PORCN**	0.74	1.67	2.09E-11	1.89E-10
**RABEPK**	-0.75	0.60	2.14E-11	1.93E-10
**RBCK1**	-0.48	0.72	2.15E-11	1.94E-10
**POLR3G**	-0.72	0.61	2.17E-11	1.96E-10
**HNRNPK**	-0.36	0.78	2.32E-11	2.10E-10
**RPL3P4**	-1.11	0.46	2.33E-11	2.10E-10
**KHDRBS1**	-0.40	0.76	2.33E-11	2.10E-10
**MRPS2**	-0.63	0.64	2.37E-11	2.13E-10
**TCP1**	-0.37	0.77	2.46E-11	2.21E-10
**RDH10**	0.76	1.70	2.48E-11	2.23E-10
**SERPINB3**	-0.72	0.61	2.50E-11	2.25E-10
**SON**	-0.35	0.78	2.62E-11	2.36E-10
**CISD3**	-0.70	0.62	2.62E-11	2.36E-10
**SIRPB2**	2.11	4.31	2.66E-11	2.39E-10
**CD320**	-0.83	0.56	2.67E-11	2.39E-10
**GLTSCR2**	-0.45	0.73	2.68E-11	2.41E-10
**NRP1**	-0.82	0.57	2.79E-11	2.50E-10
**NUCKS1**	-0.44	0.74	2.85E-11	2.56E-10
**HOMER2**	-0.98	0.51	2.95E-11	2.64E-10
**KIAA1147**	-0.74	0.60	2.95E-11	2.64E-10
**FKBP1A**	0.37	1.29	2.96E-11	2.65E-10

**BUD13**	-0.69	0.62	3.05E-11	2.73E-10
**KMT2A**	-0.64	0.64	3.08E-11	2.75E-10
**SETD1B**	-0.64	0.64	3.08E-11	2.75E-10
**IGFBP2**	0.43	1.34	3.08E-11	2.75E-10
**SHANK3**	0.78	1.71	3.12E-11	2.79E-10
**GLIS2**	1.32	2.50	3.14E-11	2.80E-10
**SLC16A5**	1.03	2.04	3.26E-11	2.91E-10
**KRT78**	-0.69	0.62	3.33E-11	2.97E-10
**HNRNPL**	-0.39	0.76	3.34E-11	2.98E-10
**CRABP2**	-0.40	0.76	3.41E-11	3.03E-10
**CHD1**	-0.58	0.67	3.47E-11	3.09E-10
**DKFZp434J0226**	1.75	3.37	3.48E-11	3.10E-10
**HIST1H2BG**	2.25	4.75	3.52E-11	3.13E-10
**HOXC13**	0.82	1.77	3.57E-11	3.17E-10
**CTTN**	0.36	1.28	3.58E-11	3.18E-10
**SND1**	-0.37	0.78	3.61E-11	3.21E-10
**RGS3**	0.58	1.49	3.64E-11	3.23E-10
**SLC25A22**	-0.72	0.61	3.70E-11	3.28E-10
**SORBS1**	1.94	3.84	3.72E-11	3.30E-10
**SNUPN**	-1.07	0.48	3.82E-11	3.39E-10
**ZNF512B**	-0.58	0.67	3.84E-11	3.40E-10
**CLEC16A**	0.52	1.43	3.90E-11	3.45E-10
**METTL22**	0.82	1.77	4.03E-11	3.56E-10
**PPAT**	-0.79	0.58	4.13E-11	3.65E-10
**SKA1**	-1.61	0.33	4.16E-11	3.68E-10
**RSPH3**	0.77	1.71	4.21E-11	3.72E-10
**RGS4**	-2.12	0.23	4.28E-11	3.78E-10
**GDF11**	0.91	1.87	4.45E-11	3.93E-10
**ADAP1**	-1.25	0.42	4.56E-11	4.02E-10
**ATG13**	0.51	1.43	4.62E-11	4.07E-10
**PCIF1**	-0.72	0.61	4.66E-11	4.11E-10
**RBBP7**	-0.43	0.74	4.75E-11	4.18E-10
**NAB2**	-0.59	0.67	4.98E-11	4.39E-10
**MAK16**	-0.68	0.62	5.02E-11	4.41E-10
**SORCS2**	-1.60	0.33	5.10E-11	4.48E-10
**CDC27**	-0.48	0.72	5.12E-11	4.50E-10
**CCNY**	-0.51	0.70	5.15E-11	4.53E-10
**RSL24D1**	-0.63	0.64	5.19E-11	4.55E-10
**IVD**	0.65	1.57	5.22E-11	4.58E-10
**EIF3A**	-0.38	0.77	5.23E-11	4.59E-10
**TOM1L1**	-0.62	0.65	5.30E-11	4.64E-10
**KLF4**	0.49	1.40	5.33E-11	4.67E-10
**GOLGA7B**	0.74	1.67	5.38E-11	4.72E-10
**SERPINB1**	-0.59	0.66	5.46E-11	4.78E-10
**MACC1**	0.50	1.42	5.47E-11	4.79E-10
**SGSH**	0.74	1.67	5.48E-11	4.79E-10
**SNRPB**	-0.44	0.74	5.49E-11	4.80E-10
**ANKRD33B**	1.25	2.38	5.52E-11	4.82E-10
**NR1D1**	0.77	1.71	5.65E-11	4.93E-10
**FHL3**	-0.80	0.57	5.69E-11	4.97E-10
**PPP2R4**	-0.41	0.75	5.72E-11	4.99E-10
**SPRR2E**	-1.29	0.41	5.80E-11	5.06E-10
**NIPSNAP1**	0.54	1.46	5.81E-11	5.06E-10
**NEU1**	0.58	1.50	5.87E-11	5.11E-10
**ABHD2**	-0.45	0.73	5.93E-11	5.17E-10
**DNAJB5**	0.69	1.62	5.95E-11	5.18E-10
**RABAC1**	0.58	1.49	5.96E-11	5.18E-10
**ZKSCAN1**	0.44	1.36	6.06E-11	5.27E-10
**TPR**	-0.41	0.75	6.08E-11	5.28E-10
**RSAD1**	-0.76	0.59	6.12E-11	5.32E-10
**ZNF433**	1.35	2.54	6.19E-11	5.37E-10
**RAB3D**	-0.52	0.70	6.29E-11	5.45E-10
**MPI**	0.61	1.53	6.41E-11	5.56E-10
**NRM**	-0.83	0.56	6.53E-11	5.66E-10
**MYOF**	-0.35	0.79	6.67E-11	5.78E-10
**PLA2G3**	-1.57	0.34	6.71E-11	5.81E-10
**LAMA5**	-0.43	0.74	6.77E-11	5.86E-10
**PPM1A**	0.58	1.50	6.81E-11	5.89E-10
**MANSC1**	-0.64	0.64	6.82E-11	5.90E-10
**TAX1BP3**	0.92	1.90	6.83E-11	5.90E-10
**CYHR1**	0.70	1.63	6.87E-11	5.94E-10
**R3HDM4**	-0.48	0.71	6.90E-11	5.96E-10
**NR5A1**	2.73	6.63	6.99E-11	6.03E-10
**PHYKPL**	0.92	1.89	7.11E-11	6.13E-10
**RPAP1**	0.54	1.45	7.17E-11	6.19E-10
**CHEK2**	-0.82	0.57	7.24E-11	6.24E-10
**CSNK2A1**	-0.39	0.76	7.27E-11	6.26E-10
**MMP1**	-1.37	0.39	7.28E-11	6.27E-10
**SFXN4**	-0.74	0.60	7.30E-11	6.28E-10
**WDHD1**	-0.56	0.68	7.35E-11	6.32E-10
**YDJC**	-0.69	0.62	7.36E-11	6.33E-10
**ADM**	-0.67	0.63	7.38E-11	6.34E-10
**GPX2**	-1.01	0.50	7.55E-11	6.49E-10
**TNFRSF12A**	-0.46	0.73	7.61E-11	6.54E-10
**FAM217B**	-0.70	0.61	7.65E-11	6.56E-10
**HLA-E**	0.39	1.31	7.75E-11	6.65E-10
**SQRDL**	-0.62	0.65	8.00E-11	6.86E-10
**SDC1**	-0.35	0.78	8.03E-11	6.88E-10
**MTERF3**	-0.79	0.58	8.19E-11	7.02E-10
**OST4**	0.45	1.37	8.30E-11	7.11E-10

**CUEDC2**	0.51	1.42	8.33E-11	7.12E-10
**PLTP**	0.50	1.41	8.56E-11	7.32E-10
**IER3**	0.52	1.44	8.63E-11	7.37E-10
**RCBTB1**	0.69	1.62	8.75E-11	7.47E-10
**CEP295**	-0.90	0.54	8.75E-11	7.47E-10
**GDPD5**	1.92	3.79	9.00E-11	7.68E-10
**UNC13D**	-0.79	0.58	9.01E-11	7.68E-10
**TRIM28**	-0.43	0.74	9.04E-11	7.71E-10
**SYBU**	-1.23	0.43	9.24E-11	7.87E-10
**FBXL12**	0.78	1.71	9.25E-11	7.88E-10
**TNFSF4**	2.43	5.38	9.28E-11	7.90E-10
**RTTN**	0.54	1.46	9.41E-11	8.01E-10
**HSDL2**	0.55	1.47	9.72E-11	8.27E-10
**DYRK1B**	1.05	2.08	9.73E-11	8.27E-10
**TSG101**	0.43	1.35	9.81E-11	8.33E-10
**PRPF4**	-0.48	0.72	9.95E-11	8.45E-10
**PPTC7**	-0.49	0.71	9.97E-11	8.46E-10
**CUL4A**	-0.45	0.73	1.01E-10	8.56E-10
**LRRK1**	-0.60	0.66	1.03E-10	8.70E-10
**B2M**	0.42	1.33	1.04E-10	8.79E-10
**ACAA1**	0.53	1.44	1.04E-10	8.80E-10
**GBAS**	-0.68	0.62	1.07E-10	9.03E-10
**PSIP1**	-0.65	0.64	1.07E-10	9.06E-10
**ZNF215**	-1.17	0.44	1.09E-10	9.23E-10
**RAB3IP**	0.61	1.53	1.09E-10	9.23E-10
**SEC14L2**	0.56	1.47	1.11E-10	9.35E-10
**AGFG1**	0.42	1.34	1.11E-10	9.39E-10
**MCC**	0.46	1.38	1.11E-10	9.41E-10
**KLHL23**	-1.28	0.41	1.12E-10	9.47E-10
**TMEM177**	-0.86	0.55	1.13E-10	9.55E-10
**UBA3**	0.50	1.41	1.13E-10	9.56E-10
**RP11-356I2.4**	1.90	3.73	1.15E-10	9.68E-10
**NPM1**	-0.41	0.75	1.19E-10	1.01E-09
**PHYH**	1.01	2.01	1.21E-10	1.02E-09
**ARID4A**	0.85	1.80	1.22E-10	1.03E-09
**RIMS4**	2.59	6.02	1.22E-10	1.03E-09
**SAE1**	-0.41	0.75	1.26E-10	1.06E-09
**EXO1**	-0.81	0.57	1.29E-10	1.09E-09
**SLC35A4**	-0.47	0.72	1.32E-10	1.11E-09
**ATP5G2**	-0.44	0.73	1.32E-10	1.11E-09
**CYP4F22**	-1.97	0.26	1.33E-10	1.11E-09
**BCL2L1**	0.45	1.37	1.36E-10	1.14E-09
**C22orf39**	-0.86	0.55	1.36E-10	1.14E-09
**RP5-882C2.2**	2.14	4.42	1.41E-10	1.18E-09
**RCN1**	-0.52	0.70	1.43E-10	1.19E-09
**EDARADD**	-1.52	0.35	1.44E-10	1.20E-09
**CEP131**	-0.82	0.57	1.45E-10	1.22E-09
**DNASE1**	1.10	2.15	1.46E-10	1.22E-09
**MRPL24**	-0.54	0.69	1.46E-10	1.22E-09
**RP5-884C9.2**	2.55	5.84	1.46E-10	1.22E-09
**ZBTB7B**	-0.43	0.74	1.48E-10	1.24E-09
**CDKN2C**	-1.41	0.38	1.49E-10	1.25E-09
**FBXO41**	0.75	1.68	1.51E-10	1.26E-09
**MAGI1**	-0.72	0.61	1.52E-10	1.27E-09
**SIGIRR**	-1.42	0.37	1.54E-10	1.29E-09
**POLD2**	-0.47	0.72	1.55E-10	1.30E-09
**DNAJC13**	-0.48	0.72	1.57E-10	1.30E-09
**TP53I13**	0.65	1.57	1.60E-10	1.33E-09
**PLXNA1**	-0.44	0.74	1.62E-10	1.35E-09
**UCN2**	1.31	2.47	1.62E-10	1.35E-09
**CD46**	0.50	1.42	1.68E-10	1.40E-09
**OAS1**	0.66	1.58	1.72E-10	1.43E-09
**GTF3C2**	-0.50	0.71	1.73E-10	1.44E-09
**RP5-1039K5.19**	1.16	2.24	1.77E-10	1.47E-09
**ITGB4**	-0.43	0.74	1.79E-10	1.48E-09
**RABEP2**	0.69	1.61	1.80E-10	1.50E-09
**PSMG1**	-0.55	0.68	1.84E-10	1.52E-09
**KIAA0319L**	0.51	1.43	1.84E-10	1.53E-09
**GNAI2**	-0.39	0.76	1.85E-10	1.53E-09
**OAZ1**	-0.38	0.77	1.87E-10	1.55E-09
**RAC2**	0.58	1.49	1.99E-10	1.64E-09
**NFIA**	-0.61	0.65	2.06E-10	1.70E-09
**NCK1**	-0.66	0.63	2.11E-10	1.74E-09
**WBSCR27**	1.45	2.74	2.14E-10	1.77E-09
**PMF1**	-0.80	0.57	2.15E-10	1.77E-09
**KIAA0895L**	0.92	1.90	2.17E-10	1.79E-09
**NCALD**	-1.86	0.28	2.18E-10	1.80E-09
**PCLO**	0.69	1.61	2.20E-10	1.81E-09
**MAU2**	0.56	1.47	2.20E-10	1.82E-09
**SRSF4**	-0.43	0.74	2.23E-10	1.84E-09
**BCR**	-0.45	0.73	2.26E-10	1.86E-09
**PDP2**	-0.61	0.66	2.28E-10	1.87E-09
**CAPRIN1**	-0.35	0.78	2.28E-10	1.88E-09
**NCOA4**	0.39	1.31	2.36E-10	1.94E-09
**ZDHHC1**	1.14	2.21	2.38E-10	1.96E-09
**KDM3B**	-0.45	0.73	2.39E-10	1.96E-09
**FAF1**	-0.49	0.71	2.45E-10	2.01E-09
**C15orf59**	-1.49	0.36	2.51E-10	2.06E-09
**EME2**	0.93	1.90	2.59E-10	2.12E-09
**MAP3K5**	-0.82	0.57	2.65E-10	2.17E-09

**SIMC1**	-0.87	0.55	2.65E-10	2.17E-09
**DSN1**	-0.74	0.60	2.65E-10	2.17E-09
**CFL2**	0.74	1.67	2.70E-10	2.21E-09
**UBR3**	0.51	1.42	2.70E-10	2.21E-09
**CORO1C**	0.34	1.26	2.72E-10	2.23E-09
**FGD6**	-0.49	0.71	2.73E-10	2.24E-09
**C9orf91**	-0.87	0.55	2.75E-10	2.24E-09
**ZBTB7C**	1.63	3.09	2.75E-10	2.25E-09
**SSB**	-0.51	0.70	2.78E-10	2.27E-09
**PRKY**	0.93	1.90	2.80E-10	2.29E-09
**PITPNM3**	-0.45	0.73	2.81E-10	2.29E-09
**PVT1**	1.14	2.20	2.81E-10	2.29E-09
**JOSD1**	-0.46	0.73	2.98E-10	2.43E-09
**SDHC**	0.46	1.38	3.01E-10	2.45E-09
**HAUS8**	-0.96	0.51	3.02E-10	2.46E-09
**KRT13**	0.34	1.27	3.03E-10	2.47E-09
**JADE2**	-0.64	0.64	3.04E-10	2.47E-09
**CKAP4**	-0.36	0.78	3.20E-10	2.60E-09
**SNRNP27**	0.72	1.65	3.23E-10	2.62E-09
**RSL1D1**	-0.38	0.77	3.24E-10	2.63E-09
**ANKUB1**	2.64	6.23	3.29E-10	2.67E-09
**PARP12**	0.58	1.49	3.29E-10	2.67E-09
**SPATA7**	1.07	2.09	3.35E-10	2.71E-09
**C19orf24**	-0.71	0.61	3.35E-10	2.72E-09
**ELL2**	0.40	1.32	3.39E-10	2.75E-09
**ZNF812**	1.73	3.31	3.40E-10	2.76E-09
**FUT11**	-0.93	0.52	3.42E-10	2.77E-09
**ANKRD52**	-0.50	0.71	3.43E-10	2.78E-09
**BAHCC1**	-0.82	0.56	3.46E-10	2.80E-09
**HPRT1**	-0.61	0.66	3.50E-10	2.83E-09
**GALNT14**	-0.59	0.66	3.50E-10	2.83E-09
**LIG1**	0.46	1.38	3.57E-10	2.89E-09
**EIF4H**	0.34	1.27	3.60E-10	2.91E-09
**STX16**	0.51	1.43	3.62E-10	2.92E-09
**TMEM131**	0.42	1.34	3.63E-10	2.93E-09
**C9orf3**	-0.81	0.57	3.66E-10	2.95E-09
**MPV17**	0.52	1.43	3.68E-10	2.97E-09
**TSEN2**	-0.81	0.57	3.68E-10	2.97E-09
**DDAH2**	-1.21	0.43	3.73E-10	3.01E-09
**MRPS26**	-0.55	0.68	3.74E-10	3.01E-09
**ABCB10**	-0.67	0.63	3.75E-10	3.02E-09
**THRA**	-0.79	0.58	3.76E-10	3.03E-09
**SNHG5**	-0.50	0.71	3.77E-10	3.03E-09
**PBX1**	0.66	1.58	3.78E-10	3.03E-09
**UGGT2**	0.79	1.72	3.80E-10	3.05E-09
**SMARCB1**	-0.44	0.74	3.80E-10	3.05E-09
**ABCB9**	1.00	2.00	3.85E-10	3.09E-09
**FAXDC2**	1.70	3.25	3.85E-10	3.09E-09
**EVA1C**	-0.80	0.58	3.85E-10	3.09E-09
**GRAMD1B**	1.74	3.35	3.91E-10	3.13E-09
**PLCH2**	0.43	1.35	3.94E-10	3.16E-09
**LEPROT**	0.51	1.42	4.00E-10	3.21E-09
**CALML5**	-0.69	0.62	4.07E-10	3.26E-09
**RP11-54H7.4**	0.86	1.82	4.12E-10	3.30E-09
**PRKX**	0.54	1.45	4.14E-10	3.31E-09
**NR1H3**	0.92	1.89	4.17E-10	3.33E-09
**GTF3C1**	0.39	1.31	4.26E-10	3.40E-09
**SEMA3F**	0.39	1.31	4.33E-10	3.46E-09
**SLCO3A1**	-0.62	0.65	4.52E-10	3.61E-09
**HNRNPA3**	-0.46	0.73	4.57E-10	3.65E-09
**ITPK1**	-0.40	0.76	4.61E-10	3.67E-09
**SP6**	0.58	1.49	4.70E-10	3.74E-09
**MED29**	0.51	1.42	4.82E-10	3.84E-09
**SENP5**	0.62	1.53	4.83E-10	3.84E-09
**TET3**	-0.57	0.67	4.89E-10	3.89E-09
**AGTRAP**	0.57	1.48	4.94E-10	3.93E-09
**PRRC2A**	-0.40	0.76	4.95E-10	3.93E-09
**CA12**	-0.35	0.78	4.99E-10	3.97E-09
**PODXL2**	-1.10	0.47	5.10E-10	4.05E-09
**RPS6KA3**	-0.49	0.71	5.10E-10	4.05E-09
**RAD18**	-0.62	0.65	5.23E-10	4.15E-09
**PUM3**	-0.43	0.74	5.26E-10	4.17E-09
**MXRA5**	1.08	2.12	5.34E-10	4.23E-09
**PAK1IP1**	-0.64	0.64	5.39E-10	4.27E-09
**WNT7B**	-0.81	0.57	5.49E-10	4.35E-09
**DNHD1**	1.01	2.02	5.50E-10	4.36E-09
**UCA1**	1.41	2.65	5.53E-10	4.38E-09
**CLDN15**	1.35	2.56	5.63E-10	4.45E-09
**TPPP**	-0.69	0.62	5.63E-10	4.46E-09
**DONSON**	-0.76	0.59	5.79E-10	4.58E-09
**FBXO38**	0.54	1.45	5.81E-10	4.59E-09
**URB2**	-0.71	0.61	5.86E-10	4.63E-09
**LSM14B**	-0.56	0.68	5.86E-10	4.63E-09
**DDX10**	-0.57	0.68	5.93E-10	4.68E-09
**ECI1**	-0.63	0.65	5.94E-10	4.69E-09
**B3GNT3**	0.56	1.47	5.94E-10	4.69E-09
**NCOA7**	-0.67	0.63	5.97E-10	4.70E-09
**ZNF383**	1.13	2.19	5.98E-10	4.71E-09
**GATAD2A**	-0.49	0.71	6.01E-10	4.73E-09
**MTCH1**	0.41	1.32	6.02E-10	4.74E-09

**CREBL2**	0.64	1.56	6.05E-10	4.76E-09
**C10orf88**	0.94	1.92	6.05E-10	4.76E-09
**SLC41A3**	0.54	1.46	6.09E-10	4.79E-09
**BCL11A**	-1.48	0.36	6.16E-10	4.84E-09
**UBE2SP1**	-1.57	0.34	6.21E-10	4.88E-09
**DMPK**	1.01	2.01	6.24E-10	4.90E-09
**DAGLA**	1.95	3.88	6.30E-10	4.94E-09
**FAR2**	-0.99	0.50	6.31E-10	4.95E-09
**BAZ2A**	-0.35	0.78	6.32E-10	4.95E-09
**XYLB**	-1.15	0.45	6.36E-10	4.98E-09
**TMEM134**	0.57	1.49	6.68E-10	5.23E-09
**SPTLC1**	0.43	1.35	6.76E-10	5.30E-09
**SULT1A1**	0.99	1.99	6.82E-10	5.34E-09
**TMEM68**	0.75	1.68	6.82E-10	5.34E-09
**DCP1B**	0.72	1.65	6.84E-10	5.35E-09
**GEMIN5**	-0.55	0.68	6.92E-10	5.41E-09
**ZFR**	-0.40	0.76	7.11E-10	5.55E-09
**CLSTN3**	0.75	1.68	7.12E-10	5.56E-09
**FRAS1**	-0.85	0.55	7.15E-10	5.58E-09
**PPP1CC**	-0.42	0.75	7.21E-10	5.63E-09
**SMAGP**	-0.53	0.69	7.25E-10	5.66E-09
**FOXN1**	-1.04	0.49	7.35E-10	5.73E-09
**PLD5**	-0.67	0.63	7.36E-10	5.73E-09
**CORO1B**	0.43	1.35	7.47E-10	5.82E-09
**KCNE4**	2.47	5.53	7.49E-10	5.83E-09
**FAH**	0.62	1.54	7.58E-10	5.90E-09
**ZNF324**	0.82	1.77	7.62E-10	5.93E-09
**ERVMER34-1**	-0.83	0.56	7.64E-10	5.94E-09
**SPRYD7**	0.61	1.52	7.64E-10	5.94E-09
**DAB2IP**	-0.43	0.74	7.67E-10	5.96E-09
**TSR3**	-0.59	0.67	7.85E-10	6.09E-09
**CEPT1**	0.63	1.54	7.87E-10	6.11E-09
**HIST1H2BC**	1.78	3.44	7.90E-10	6.13E-09
**C1orf198**	-0.59	0.66	7.96E-10	6.17E-09
**TPCN1**	0.54	1.45	8.02E-10	6.22E-09
**DCUN1D5**	-0.53	0.69	8.06E-10	6.24E-09
**02-mar**	0.74	1.67	8.08E-10	6.26E-09
**FNDC3A**	-0.50	0.71	8.09E-10	6.26E-09
**CYP2J2**	1.35	2.55	8.15E-10	6.31E-09
**CBR4**	0.65	1.57	8.20E-10	6.34E-09
**CTR9**	0.45	1.37	8.27E-10	6.39E-09
**NAE1**	-0.51	0.70	8.31E-10	6.42E-09
**ARF4**	0.36	1.28	8.31E-10	6.42E-09
**SBK1**	2.18	4.54	8.31E-10	6.42E-09
**PRPS2**	-0.47	0.72	8.38E-10	6.46E-09
**IPO5**	-0.35	0.78	8.43E-10	6.50E-09
**PLEKHA7**	0.55	1.46	8.50E-10	6.55E-09
**C12orf45**	0.79	1.73	8.52E-10	6.57E-09
**NT5C**	-0.68	0.63	8.61E-10	6.63E-09
**GATS**	1.85	3.62	8.69E-10	6.69E-09
**S100A13**	0.60	1.52	8.75E-10	6.74E-09
**ESRRA**	0.50	1.41	8.84E-10	6.80E-09
**MEGF9**	0.61	1.53	8.90E-10	6.85E-09
**ANKLE1**	-1.32	0.40	9.11E-10	7.01E-09
**DTD1**	-0.52	0.70	9.13E-10	7.02E-09
**TXLNG**	-0.58	0.67	9.45E-10	7.26E-09
**TNKS2**	0.45	1.37	9.53E-10	7.32E-09
**NKX1-2**	2.26	4.80	9.62E-10	7.38E-09
**MAML3**	-1.12	0.46	9.62E-10	7.38E-09
**RBMS2**	-0.45	0.73	9.66E-10	7.40E-09
**CTF1**	1.53	2.88	9.77E-10	7.48E-09
**SLC12A8**	-0.97	0.51	9.80E-10	7.51E-09
**CHMP1B**	0.46	1.37	9.83E-10	7.53E-09
**IFITM10**	1.47	2.78	9.98E-10	7.64E-09
**ALOXE3**	-0.96	0.51	1.01E-09	7.75E-09
**MXI1**	0.74	1.67	1.01E-09	7.76E-09
**QTRTD1**	-0.60	0.66	1.03E-09	7.84E-09
**ZCCHC14**	-0.65	0.64	1.04E-09	7.94E-09
**COL9A2**	-1.46	0.36	1.04E-09	7.96E-09
**CARS**	-0.51	0.70	1.06E-09	8.07E-09
**SMARCD1**	-0.41	0.75	1.06E-09	8.09E-09
**SCNN1B**	-1.04	0.48	1.07E-09	8.15E-09
**RP11-449P15.2**	1.84	3.58	1.07E-09	8.18E-09
**AUNIP**	-0.85	0.56	1.07E-09	8.19E-09
**RP11-277P12.20**	1.35	2.55	1.08E-09	8.23E-09
**PCF11**	-0.54	0.69	1.08E-09	8.23E-09
**RHBDD2**	0.66	1.58	1.09E-09	8.28E-09
**AKR1B1**	0.52	1.43	1.09E-09	8.33E-09
**IRAK1**	-0.44	0.74	1.11E-09	8.41E-09
**SLC2A3**	-1.35	0.39	1.11E-09	8.41E-09
**BCL6**	0.66	1.58	1.11E-09	8.43E-09
**RP11-44F14.9**	2.41	5.30	1.12E-09	8.49E-09
**ENTPD3**	0.62	1.53	1.14E-09	8.65E-09
**FAM109B**	-0.94	0.52	1.15E-09	8.71E-09
**BRMS1**	0.47	1.38	1.16E-09	8.83E-09
**UFD1L**	0.45	1.37	1.22E-09	9.21E-09
**KMT2E**	-0.46	0.73	1.23E-09	9.32E-09
**DIP2C**	-0.73	0.60	1.24E-09	9.38E-09
**KIAA0368**	-0.37	0.77	1.27E-09	9.63E-09
**SCEL**	-0.42	0.75	1.27E-09	9.64E-09

**ZNF81**	0.94	1.92	1.28E-09	9.69E-09
**ZCCHC11**	0.50	1.42	1.29E-09	9.76E-09
**AC006262.4**	1.95	3.85	1.31E-09	9.89E-09
**UBIAD1**	-0.80	0.57	1.32E-09	9.95E-09
**SPRYD3**	-0.48	0.72	1.33E-09	1.00E-08
**TSC2**	0.45	1.36	1.33E-09	1.00E-08
**STK10**	0.47	1.39	1.36E-09	1.03E-08
**SUN2**	-0.49	0.71	1.37E-09	1.03E-08
**CYP27B1**	-0.58	0.67	1.41E-09	1.06E-08
**MTCO3P12**	0.52	1.44	1.46E-09	1.10E-08
**ZNF480**	-0.80	0.58	1.48E-09	1.12E-08
**FITM2**	0.73	1.66	1.49E-09	1.12E-08
**TTC5**	-0.86	0.55	1.53E-09	1.15E-08
**OCIAD1**	0.43	1.34	1.53E-09	1.15E-08
**PARP9**	0.57	1.48	1.54E-09	1.16E-08
**SRCIN1**	1.68	3.22	1.55E-09	1.16E-08
**TRIM62**	0.69	1.62	1.56E-09	1.18E-08
**PDLIM7**	0.49	1.40	1.57E-09	1.18E-08
**SLC6A11**	-0.53	0.69	1.58E-09	1.18E-08
**SHOC2**	0.51	1.42	1.63E-09	1.23E-08
**ZBTB40**	0.57	1.48	1.64E-09	1.23E-08
**MTR**	-0.54	0.69	1.66E-09	1.25E-08
**GMPS**	-0.41	0.75	1.67E-09	1.25E-08
**LINC00675**	2.51	5.70	1.73E-09	1.30E-08
**MAP1LC3B**	0.51	1.42	1.73E-09	1.30E-08
**TRMT112**	-0.41	0.75	1.75E-09	1.31E-08
**RAB38**	-0.42	0.75	1.75E-09	1.31E-08
**GSG2**	-0.90	0.54	1.76E-09	1.31E-08
**FAM72D**	-2.09	0.23	1.78E-09	1.33E-08
**GBA2**	0.50	1.41	1.79E-09	1.34E-08
**HNRNPAB**	-0.41	0.75	1.80E-09	1.35E-08
**GPT2**	-0.52	0.70	1.81E-09	1.35E-08
**CTNNB1**	0.34	1.27	1.83E-09	1.37E-08
**IFI6**	0.47	1.38	1.83E-09	1.37E-08
**SH3TC1**	0.58	1.49	1.86E-09	1.38E-08
**RNF19A**	0.61	1.52	1.93E-09	1.44E-08
**HCFC1**	-0.46	0.73	1.94E-09	1.44E-08
**DNAAF5**	-0.54	0.69	1.94E-09	1.44E-08
**ITPRIP**	0.44	1.36	1.94E-09	1.45E-08
**GLUD1**	-0.39	0.77	1.95E-09	1.45E-08
**ARTN**	-0.68	0.62	1.96E-09	1.46E-08
**RNF126**	-0.49	0.71	1.97E-09	1.46E-08
**RNF19B**	0.45	1.37	1.97E-09	1.46E-08
**ZNF654**	0.80	1.74	1.97E-09	1.46E-08
**NPLOC4**	0.37	1.30	1.99E-09	1.48E-08
**RPS16**	-0.31	0.81	1.99E-09	1.48E-08
**BOP1**	-0.93	0.52	2.00E-09	1.48E-08
**NUP107**	-0.51	0.70	2.02E-09	1.50E-08
**CDC42SE1**	0.34	1.26	2.08E-09	1.54E-08
**ANKRD18B**	-1.04	0.49	2.08E-09	1.54E-08
**ILF3-AS1**	-1.30	0.41	2.09E-09	1.55E-08
**PLEKHG4**	-0.90	0.54	2.11E-09	1.56E-08
**DNAJC9**	-0.76	0.59	2.13E-09	1.57E-08
**FAM189B**	-0.50	0.70	2.17E-09	1.60E-08
**MASTL**	-0.62	0.65	2.19E-09	1.62E-08
**FADS2**	-0.74	0.60	2.21E-09	1.64E-08
**H2AFY2**	-0.54	0.69	2.23E-09	1.65E-08
**SGOL1**	-0.98	0.51	2.25E-09	1.66E-08
**CHAMP1**	-0.55	0.68	2.26E-09	1.67E-08
**AKAP1**	-0.55	0.68	2.26E-09	1.67E-08
**TAF13**	0.70	1.62	2.31E-09	1.70E-08
**APOBEC3A**	1.06	2.08	2.32E-09	1.71E-08
**ILF2**	-0.34	0.79	2.37E-09	1.75E-08
**RBM39**	-0.36	0.78	2.39E-09	1.76E-08
**NEK9**	0.46	1.37	2.42E-09	1.78E-08
**CDK14**	-0.88	0.54	2.43E-09	1.79E-08
**CERS6**	0.51	1.42	2.44E-09	1.80E-08
**BCAM**	-0.43	0.74	2.47E-09	1.82E-08
**CARD11**	-1.55	0.34	2.47E-09	1.82E-08
**MAN2B2**	0.46	1.38	2.49E-09	1.83E-08
**PABPC1**	-0.30	0.81	2.49E-09	1.83E-08
**ITM2C**	0.65	1.57	2.54E-09	1.87E-08
**MKNK1**	0.48	1.40	2.61E-09	1.92E-08
**NELFB**	-0.46	0.73	2.61E-09	1.92E-08
**TMSB10**	-0.31	0.81	2.63E-09	1.93E-08
**ACHE**	2.39	5.25	2.66E-09	1.95E-08
**DIP2B**	-0.41	0.75	2.67E-09	1.96E-08
**SNAI3**	2.33	5.04	2.67E-09	1.96E-08
**CYP2S1**	0.62	1.54	2.70E-09	1.98E-08
**C9orf9**	1.08	2.11	2.71E-09	1.99E-08
**SURF4**	-0.33	0.80	2.72E-09	1.99E-08
**PLEKHA8**	0.65	1.57	2.75E-09	2.01E-08
**SPIRE1**	-0.62	0.65	2.79E-09	2.04E-08
**RNF146**	0.73	1.66	2.79E-09	2.04E-08
**HAUS6**	-0.56	0.68	2.82E-09	2.06E-08
**SIN3B**	0.46	1.38	2.82E-09	2.06E-08
**RHNO1**	-0.54	0.69	2.86E-09	2.09E-08
**HERC2**	-0.59	0.67	2.87E-09	2.09E-08
**AIM1L**	0.39	1.31	2.87E-09	2.09E-08
**AP1S3**	0.49	1.41	2.89E-09	2.10E-08

**CHMP5**	0.51	1.43	2.95E-09	2.15E-08
**IGF2R**	0.38	1.30	2.96E-09	2.16E-08
**EZH1**	0.64	1.56	2.98E-09	2.17E-08
**BLZF1**	0.51	1.42	3.01E-09	2.19E-08
**RALB**	0.44	1.35	3.01E-09	2.19E-08
**DNAJC10**	-0.38	0.77	3.04E-09	2.21E-08
**BRIP1**	-0.82	0.57	3.06E-09	2.23E-08
**ANKK1**	1.66	3.16	3.10E-09	2.25E-08
**BRI3BP**	-0.55	0.68	3.17E-09	2.30E-08
**SLC43A1**	-2.20	0.22	3.22E-09	2.34E-08
**CCDC142**	1.20	2.29	3.23E-09	2.34E-08
**SRRM1**	-0.39	0.76	3.28E-09	2.38E-08
**RIC1**	0.53	1.44	3.31E-09	2.40E-08
**NUP85**	-0.46	0.73	3.35E-09	2.43E-08
**SAMD8**	0.55	1.47	3.36E-09	2.44E-08
**ATP6V1D**	0.47	1.39	3.37E-09	2.44E-08
**PDLIM2**	-0.64	0.64	3.39E-09	2.45E-08
**PHF13**	-0.71	0.61	3.41E-09	2.47E-08
**IGSF8**	-0.61	0.66	3.43E-09	2.48E-08
**TNS3**	1.08	2.12	3.43E-09	2.48E-08
**C1orf112**	-0.74	0.60	3.48E-09	2.51E-08
**APOBEC3H**	2.47	5.54	3.50E-09	2.53E-08
**ETNK1**	0.60	1.52	3.52E-09	2.54E-08
**REPIN1**	-0.54	0.69	3.54E-09	2.56E-08
**ERLIN2**	0.42	1.34	3.55E-09	2.56E-08
**SPINK5**	-0.48	0.72	3.57E-09	2.58E-08
**MYO18A**	-0.33	0.79	3.60E-09	2.59E-08
**LARP1B**	-0.66	0.63	3.62E-09	2.61E-08
**NUP50**	-0.42	0.75	3.62E-09	2.61E-08
**ZFYVE27**	0.67	1.59	3.62E-09	2.61E-08
**ALKBH2**	-0.93	0.52	3.65E-09	2.63E-08
**TNNT1**	-0.56	0.68	3.66E-09	2.63E-08
**TM7SF2**	0.65	1.56	3.71E-09	2.67E-08
**ATP5L**	0.40	1.32	3.73E-09	2.68E-08
**HEXB**	0.42	1.34	3.78E-09	2.72E-08
**FN3KRP**	-0.59	0.66	3.78E-09	2.72E-08
**BTN2A2**	0.99	1.98	3.85E-09	2.76E-08
**MAN2A1**	-0.45	0.73	3.85E-09	2.77E-08
**BOC**	-1.40	0.38	4.07E-09	2.92E-08
**SLC26A11**	1.05	2.07	4.09E-09	2.93E-08
**NFKBIA**	0.45	1.37	4.12E-09	2.95E-08
**EPHX3**	-0.91	0.53	4.14E-09	2.97E-08
**DNAJA1**	0.37	1.29	4.16E-09	2.98E-08
**RP11-424C20.2**	-1.57	0.34	4.28E-09	3.06E-08
**ANGPTL4**	-1.04	0.49	4.31E-09	3.09E-08
**CFD**	-1.88	0.27	4.32E-09	3.09E-08
**FANCC**	-0.74	0.60	4.35E-09	3.11E-08
**DNER**	-0.80	0.57	4.41E-09	3.16E-08
**ETV4**	0.49	1.41	4.43E-09	3.17E-08
**CHST12**	0.79	1.73	4.55E-09	3.25E-08
**POU2F2**	1.32	2.50	4.57E-09	3.27E-08
**PSG4**	2.18	4.53	4.60E-09	3.28E-08
**FAM53B**	-0.57	0.68	4.76E-09	3.39E-08
**NUP93**	-0.38	0.77	4.80E-09	3.42E-08
**STX5**	0.50	1.41	4.81E-09	3.43E-08
**TARS2**	-0.48	0.72	4.85E-09	3.46E-08
**EIF4A3**	-0.38	0.77	4.89E-09	3.48E-08
**TEAD3**	0.44	1.36	4.95E-09	3.53E-08
**UTRN**	0.56	1.47	4.98E-09	3.54E-08
**NMRAL1**	0.49	1.41	5.04E-09	3.58E-08
**SH3BP2**	-0.63	0.65	5.04E-09	3.58E-08
**CLMN**	-1.53	0.35	5.05E-09	3.59E-08
**KDM5B**	0.37	1.30	5.13E-09	3.64E-08
**TMEM167A**	0.49	1.41	5.19E-09	3.69E-08
**PRR5L**	-0.70	0.62	5.41E-09	3.84E-08
**NAT10**	-0.43	0.74	5.49E-09	3.90E-08
**FOXL1**	1.19	2.28	5.57E-09	3.95E-08
**EED**	0.60	1.51	5.62E-09	3.98E-08
**AAED1**	-1.04	0.49	5.64E-09	3.99E-08
**NMNAT1**	0.74	1.67	5.67E-09	4.02E-08
**DHX38**	0.39	1.31	5.68E-09	4.02E-08
**TTC17**	0.48	1.39	5.73E-09	4.06E-08
**SPTSSA**	0.50	1.41	5.75E-09	4.07E-08
**NDST1**	0.34	1.26	5.76E-09	4.07E-08
**TOMM40**	-0.51	0.70	5.81E-09	4.11E-08
**ARHGAP40**	-0.83	0.56	5.93E-09	4.19E-08
**ALDH3A1**	1.88	3.67	5.93E-09	4.19E-08
**LPIN1**	-0.60	0.66	5.96E-09	4.21E-08
**KDM7A**	0.78	1.72	5.96E-09	4.21E-08
**GTF2B**	0.53	1.45	5.99E-09	4.23E-08
**TMEM43**	-0.37	0.77	6.09E-09	4.30E-08
**TMEM209**	-0.64	0.64	6.15E-09	4.34E-08
**DDX6**	-0.41	0.75	6.16E-09	4.34E-08
**MYH16**	1.59	3.02	6.23E-09	4.39E-08
**PDE4C**	2.41	5.30	6.32E-09	4.45E-08
**OSBPL6**	-0.65	0.64	6.43E-09	4.53E-08
**GCNT3**	1.54	2.92	6.48E-09	4.56E-08
**PPIB**	-0.42	0.75	6.56E-09	4.61E-08
**ASB1**	0.54	1.46	6.60E-09	4.64E-08
**CDK12**	-0.41	0.75	6.63E-09	4.66E-08

**MPG**	-0.61	0.66	6.77E-09	4.75E-08
**PCYOX1**	0.43	1.35	6.87E-09	4.82E-08
**BECN1**	0.44	1.36	7.01E-09	4.92E-08
**SPEN**	-0.42	0.75	7.03E-09	4.93E-08
**NCAPG2**	-0.44	0.74	7.03E-09	4.93E-08
**LRPPRC**	0.35	1.27	7.03E-09	4.93E-08
**HPSE**	-0.76	0.59	7.10E-09	4.97E-08
**COG4**	0.44	1.36	7.14E-09	5.00E-08
**TUBGCP6**	0.56	1.47	7.15E-09	5.01E-08
**MDH1**	0.38	1.30	7.17E-09	5.02E-08
**TNK2**	-0.42	0.75	7.24E-09	5.06E-08
**ZC3HAV1L**	-1.12	0.46	7.27E-09	5.08E-08
**MX1**	0.50	1.42	7.34E-09	5.13E-08
**USP35**	0.87	1.82	7.42E-09	5.18E-08
**ADCY9**	0.53	1.44	7.49E-09	5.23E-08
**STOX1**	-1.91	0.27	7.54E-09	5.26E-08
**ZBTB4**	-0.36	0.78	7.58E-09	5.29E-08
**AC004951.5**	-1.26	0.42	7.63E-09	5.33E-08
**PHF20**	0.45	1.36	7.74E-09	5.40E-08
**SMAD3**	0.40	1.32	7.82E-09	5.45E-08
**RP11-295M3.4**	2.00	4.01	7.87E-09	5.49E-08
**PPP2R5B**	0.61	1.53	7.90E-09	5.50E-08
**KLK11**	-0.41	0.75	7.93E-09	5.52E-08
**STXBP3**	0.66	1.58	8.02E-09	5.58E-08
**ATP13A1**	0.53	1.44	8.11E-09	5.65E-08
**LIMD1**	-0.60	0.66	8.27E-09	5.75E-08
**DNAJB2**	0.46	1.38	8.32E-09	5.78E-08
**KLHDC2**	0.68	1.60	8.38E-09	5.82E-08
**MANF**	-0.41	0.75	8.43E-09	5.86E-08
**CCDC18**	-1.00	0.50	8.48E-09	5.89E-08
**SMS**	-0.38	0.77	8.51E-09	5.91E-08
**FGFRL1**	-0.95	0.52	8.58E-09	5.95E-08
**RP11-706O15.1**	1.00	2.01	8.76E-09	6.08E-08
**PLXNB3**	0.73	1.65	8.79E-09	6.09E-08
**ELF3**	0.72	1.64	9.00E-09	6.24E-08
**ZBTB14**	-0.93	0.52	9.06E-09	6.28E-08
**IKZF2**	-0.66	0.63	9.13E-09	6.32E-08
**ANKRD42**	0.77	1.70	9.20E-09	6.37E-08
**S100A14**	-0.29	0.82	9.37E-09	6.48E-08
**SNRNP40**	-0.51	0.70	9.51E-09	6.58E-08
**GNB4**	-0.54	0.69	9.52E-09	6.58E-08
**DNAJC2**	-0.66	0.63	9.56E-09	6.61E-08
**PFN1**	-0.36	0.78	9.68E-09	6.69E-08
**INAFM2**	1.24	2.37	9.76E-09	6.74E-08
**PRMT1**	-0.40	0.76	9.79E-09	6.76E-08
**HNRNPH1**	-0.32	0.80	9.81E-09	6.77E-08
**EVC2**	-1.03	0.49	9.95E-09	6.86E-08
**SPIN1**	-0.43	0.74	9.97E-09	6.88E-08
**ACOT7**	0.43	1.34	1.01E-08	6.98E-08
**PNMAL1**	0.64	1.56	1.02E-08	7.04E-08
**VWA5A**	0.51	1.42	1.03E-08	7.08E-08
**SH2D2A**	0.78	1.72	1.03E-08	7.08E-08
**H2AFV**	-0.51	0.70	1.03E-08	7.09E-08
**SCLT1**	-0.77	0.58	1.03E-08	7.10E-08
**FOSL1**	0.48	1.39	1.03E-08	7.11E-08
**PAQR6**	1.59	3.01	1.05E-08	7.24E-08
**PTPN14**	-0.43	0.74	1.06E-08	7.25E-08
**SPECC1**	0.51	1.43	1.06E-08	7.29E-08
**MPP6**	-0.75	0.59	1.08E-08	7.44E-08
**EIF2B1**	0.42	1.34	1.08E-08	7.44E-08
**C19orf47**	0.59	1.51	1.09E-08	7.47E-08
**CARD19**	0.60	1.52	1.10E-08	7.51E-08
**KIAA1191**	0.39	1.31	1.10E-08	7.57E-08
**GCNT1**	-0.92	0.53	1.11E-08	7.58E-08
**PEX16**	0.61	1.53	1.12E-08	7.68E-08
**TUFT1**	0.44	1.36	1.12E-08	7.69E-08
**LINC00641**	-1.12	0.46	1.13E-08	7.70E-08
**PTGES2**	-0.46	0.73	1.13E-08	7.73E-08
**MMP25-AS1**	1.77	3.41	1.13E-08	7.73E-08
**HMGA1**	-0.40	0.76	1.14E-08	7.81E-08
**SPPL2B**	0.65	1.57	1.16E-08	7.90E-08
**MYBL2**	-0.47	0.72	1.17E-08	7.97E-08
**PRKDC**	-0.43	0.74	1.17E-08	7.99E-08
**PSME2**	0.43	1.34	1.19E-08	8.12E-08
**PM20D2**	-0.71	0.61	1.19E-08	8.12E-08
**IPO13**	0.43	1.35	1.19E-08	8.13E-08
**LLGL2**	-0.41	0.75	1.20E-08	8.17E-08
**ACD**	-0.61	0.66	1.21E-08	8.23E-08
**OAS2**	0.41	1.33	1.22E-08	8.29E-08
**KLF5**	-0.31	0.81	1.25E-08	8.54E-08
**LRP10**	0.40	1.32	1.27E-08	8.62E-08
**C1orf210**	0.98	1.97	1.28E-08	8.68E-08
**CSNK2B**	0.58	1.49	1.32E-08	8.95E-08
**BOK**	-0.51	0.70	1.32E-08	8.99E-08
**RASSF7**	-0.54	0.69	1.32E-08	9.00E-08
**MAPK12**	-1.01	0.50	1.35E-08	9.18E-08
**ARRDC1**	0.45	1.36	1.37E-08	9.31E-08
**ICAM5**	1.88	3.67	1.38E-08	9.36E-08
**CTD-2228K2.7**	0.78	1.71	1.39E-08	9.43E-08
**NIF3L1**	-0.56	0.68	1.40E-08	9.52E-08

**TMEM158**	1.99	3.96	1.42E-08	9.60E-08
**EXOSC9**	-0.61	0.66	1.42E-08	9.60E-08
**FAM234A**	0.58	1.50	1.43E-08	9.68E-08
**HLA-G**	-1.13	0.46	1.43E-08	9.70E-08
**FANCA**	-0.58	0.67	1.44E-08	9.78E-08
**HAUS4**	-0.95	0.52	1.46E-08	9.88E-08
**TRABD**	-0.45	0.73	1.48E-08	1.00E-07
**RPL13**	-0.32	0.80	1.50E-08	1.01E-07
**GTF2IRD1**	-0.51	0.70	1.50E-08	1.01E-07
**TTLL12**	-0.41	0.75	1.51E-08	1.02E-07
**SERTAD2**	0.42	1.33	1.54E-08	1.04E-07
**NODAL**	2.37	5.18	1.54E-08	1.04E-07
**ARL11**	1.36	2.57	1.57E-08	1.06E-07
**C1orf216**	-0.95	0.52	1.58E-08	1.07E-07
**FAM53C**	0.47	1.39	1.58E-08	1.07E-07
**FAM72A**	-2.02	0.25	1.59E-08	1.07E-07
**CWH43**	-0.44	0.74	1.59E-08	1.07E-07
**LARGE**	0.97	1.96	1.60E-08	1.08E-07
**01-mar**	-1.69	0.31	1.61E-08	1.08E-07
**LAMA1**	0.83	1.77	1.63E-08	1.10E-07
**MB21D1**	0.87	1.82	1.64E-08	1.10E-07
**TMED2**	-0.31	0.81	1.65E-08	1.11E-07
**SERBP1**	-0.29	0.82	1.67E-08	1.13E-07
**TPM3**	-0.29	0.82	1.68E-08	1.13E-07
**ZC3H14**	-0.39	0.76	1.68E-08	1.13E-07
**ZNF703**	0.59	1.50	1.69E-08	1.14E-07
**PPIH**	-0.51	0.70	1.69E-08	1.14E-07
**INPP4A**	0.57	1.48	1.72E-08	1.15E-07
**TBK1**	0.64	1.56	1.72E-08	1.16E-07
**HS3ST1**	0.54	1.45	1.75E-08	1.17E-07
**ARHGEF10L**	-0.58	0.67	1.78E-08	1.19E-07
**CCDC112**	-1.07	0.48	1.79E-08	1.20E-07
**KCNS2**	1.81	3.51	1.80E-08	1.21E-07
**RIC3**	1.69	3.24	1.80E-08	1.21E-07
**EVI5L**	0.45	1.37	1.81E-08	1.21E-07
**TMEM52**	1.70	3.25	1.81E-08	1.21E-07
**ZMAT2**	0.41	1.33	1.82E-08	1.22E-07
**TSPAN11**	2.39	5.23	1.83E-08	1.22E-07
**CCNA1**	-1.50	0.35	1.86E-08	1.25E-07
**ADAMTSL5**	-1.01	0.50	1.87E-08	1.25E-07
**TBCB**	-0.46	0.73	1.87E-08	1.25E-07
**TAF4**	-0.62	0.65	1.87E-08	1.25E-07
**RAET1L**	0.47	1.38	1.91E-08	1.28E-07
**AKIRIN1**	0.41	1.33	1.96E-08	1.31E-07
**NEK3**	-1.04	0.48	2.01E-08	1.34E-07
**LIPA**	0.43	1.35	2.02E-08	1.35E-07
**PKP2**	-0.38	0.77	2.03E-08	1.35E-07
**VAMP8**	0.39	1.31	2.03E-08	1.35E-07
**C1orf122**	0.50	1.41	2.04E-08	1.36E-07
**UHMK1**	0.39	1.31	2.07E-08	1.38E-07
**ARID4B**	0.58	1.50	2.09E-08	1.39E-07
**MCHR1**	2.24	4.74	2.09E-08	1.39E-07
**GPATCH2L**	0.48	1.40	2.09E-08	1.39E-07
**AHSA1**	-0.34	0.79	2.16E-08	1.43E-07
**HECTD3**	0.45	1.37	2.21E-08	1.47E-07
**PITRM1**	0.33	1.25	2.32E-08	1.54E-07
**RP11-416I2.1**	1.77	3.41	2.32E-08	1.54E-07
**COX11**	0.58	1.49	2.36E-08	1.56E-07
**CCNE1**	-0.62	0.65	2.36E-08	1.57E-07
**AFAP1**	-0.54	0.69	2.38E-08	1.58E-07
**MFSD10**	-0.50	0.71	2.42E-08	1.60E-07
**CPNE2**	-0.53	0.69	2.42E-08	1.60E-07
**PLCB2**	-2.02	0.25	2.43E-08	1.61E-07
**DUSP2**	-1.69	0.31	2.43E-08	1.61E-07
**DVL2**	-0.46	0.73	2.45E-08	1.62E-07
**PPCS**	0.49	1.40	2.46E-08	1.63E-07
**BBS2**	0.53	1.44	2.49E-08	1.65E-07
**GOLGA8B**	1.00	2.00	2.50E-08	1.65E-07
**CTPS1**	0.37	1.30	2.50E-08	1.65E-07
**R3HCC1**	-0.62	0.65	2.51E-08	1.66E-07
**ZNRF2**	0.92	1.89	2.53E-08	1.67E-07
**EIF2S2**	-0.37	0.77	2.53E-08	1.67E-07
**MICAL3**	-0.50	0.71	2.62E-08	1.73E-07
**SLC2A12**	1.80	3.48	2.62E-08	1.73E-07
**ASAP3**	0.49	1.40	2.62E-08	1.73E-07
**ZDHHC16**	0.46	1.38	2.64E-08	1.74E-07
**LMNA**	-0.29	0.82	2.68E-08	1.77E-07
**DANCR**	-0.62	0.65	2.69E-08	1.77E-07
**GOLGA2**	-0.40	0.76	2.70E-08	1.78E-07
**TLR3**	0.98	1.97	2.71E-08	1.78E-07
**SNX24**	-0.66	0.63	2.74E-08	1.80E-07
**TMEM67**	0.95	1.93	2.75E-08	1.81E-07
**FAM58A**	-0.61	0.66	2.77E-08	1.82E-07
**TESK2**	1.13	2.18	2.78E-08	1.83E-07
**PINLYP**	0.78	1.71	2.78E-08	1.83E-07
**CCDC64B**	-0.53	0.69	2.80E-08	1.84E-07
**CCDC88A**	-0.50	0.71	2.80E-08	1.84E-07
**SLC25A46**	0.46	1.37	2.83E-08	1.86E-07
**SLC25A42**	0.89	1.86	2.83E-08	1.86E-07
**RP11-1094H24.4**	1.44	2.71	2.87E-08	1.88E-07

**RNF165**	-1.40	0.38	2.87E-08	1.88E-07
**PTS**	0.60	1.51	2.88E-08	1.89E-07
**EHD3**	0.66	1.58	2.91E-08	1.91E-07
**LONRF1**	-0.83	0.56	2.93E-08	1.92E-07
**MAN2B1**	0.51	1.42	2.94E-08	1.92E-07
**APAF1**	0.58	1.50	2.95E-08	1.93E-07
**CCDC88C**	-0.50	0.71	2.98E-08	1.95E-07
**ANAPC1**	-0.47	0.72	3.00E-08	1.96E-07
**HYOU1**	-0.33	0.79	3.00E-08	1.96E-07
**SCLY**	-1.47	0.36	3.02E-08	1.97E-07
**PSG5**	1.54	2.90	3.02E-08	1.97E-07
**PPP4R3A**	0.40	1.32	3.03E-08	1.98E-07
**MCM8**	-0.56	0.68	3.05E-08	1.99E-07
**CENPJ**	-0.61	0.65	3.06E-08	2.00E-07
**KLC3**	0.49	1.40	3.07E-08	2.00E-07
**TRMT61A**	-0.74	0.60	3.10E-08	2.02E-07
**CGRRF1**	1.03	2.05	3.12E-08	2.03E-07
**PPP2CB**	0.43	1.35	3.14E-08	2.05E-07
**RPE65**	1.21	2.32	3.15E-08	2.05E-07
**TOR4A**	-0.52	0.70	3.15E-08	2.05E-07
**BCAT2**	-0.58	0.67	3.16E-08	2.06E-07
**MTDH**	-0.36	0.78	3.19E-08	2.08E-07
**C10orf82**	2.04	4.12	3.26E-08	2.12E-07
**NIT1**	0.56	1.48	3.29E-08	2.14E-07
**THEMIS2**	1.31	2.47	3.30E-08	2.14E-07
**PTK2**	0.37	1.29	3.30E-08	2.14E-07
**WARS**	-0.34	0.79	3.34E-08	2.17E-07
**GORASP2**	-0.36	0.78	3.36E-08	2.18E-07
**CALU**	-0.34	0.79	3.36E-08	2.18E-07
**LACTB2**	0.79	1.73	3.36E-08	2.18E-07
**ALPK3**	1.77	3.42	3.37E-08	2.18E-07
**CENPP**	0.91	1.88	3.38E-08	2.19E-07
**MND1**	-0.94	0.52	3.40E-08	2.21E-07
**PPARGC1B**	-0.70	0.62	3.48E-08	2.26E-07
**GDE1**	0.41	1.33	3.49E-08	2.26E-07
**TMSB4X**	-0.34	0.79	3.50E-08	2.26E-07
**NAT6**	0.66	1.58	3.54E-08	2.29E-07
**KLHL20**	0.59	1.50	3.55E-08	2.30E-07
**SRD5A3**	-0.77	0.59	3.65E-08	2.36E-07
**RAB11FIP1**	-0.34	0.79	3.65E-08	2.36E-07
**AC016735.2**	1.50	2.82	3.66E-08	2.37E-07
**POLR1B**	-0.46	0.73	3.73E-08	2.41E-07
**GLRX3**	-0.34	0.79	3.76E-08	2.43E-07
**KIAA1324**	2.06	4.18	3.77E-08	2.43E-07
**ATG2A**	0.52	1.43	3.79E-08	2.44E-07
**CTD-2267D19.3**	1.08	2.11	3.82E-08	2.46E-07
**PTCH1**	-1.02	0.49	3.88E-08	2.50E-07
**ACAD8**	0.60	1.52	3.96E-08	2.55E-07
**IRAK3**	-1.32	0.40	4.00E-08	2.58E-07
**SLC46A1**	1.47	2.77	4.02E-08	2.59E-07
**TMED10**	-0.31	0.81	4.03E-08	2.59E-07
**SLC9A3R2**	-0.96	0.52	4.04E-08	2.60E-07
**DHODH**	-0.91	0.53	4.05E-08	2.61E-07
**KIF24**	-0.80	0.57	4.07E-08	2.62E-07
**AP3M1**	-0.40	0.76	4.10E-08	2.64E-07
**RAP1GDS1**	-0.41	0.75	4.11E-08	2.64E-07
**TSC22D4**	0.42	1.34	4.18E-08	2.69E-07
**H1F0**	0.32	1.24	4.19E-08	2.69E-07
**CD2AP**	0.43	1.34	4.28E-08	2.75E-07
**CTD-2510F5.4**	-1.46	0.36	4.32E-08	2.77E-07
**DLAT**	-0.39	0.76	4.34E-08	2.79E-07
**CD164**	0.37	1.29	4.36E-08	2.79E-07
**MNT**	-0.55	0.68	4.38E-08	2.80E-07
**ZCCHC8**	-0.58	0.67	4.42E-08	2.83E-07
**LINC00888**	1.50	2.82	4.42E-08	2.83E-07
**ELMO3**	-0.51	0.70	4.47E-08	2.86E-07
**RMI2**	-0.83	0.56	4.52E-08	2.89E-07
**U2SURP**	-0.39	0.76	4.52E-08	2.89E-07
**C15orf40**	0.74	1.67	4.55E-08	2.91E-07
**EIF4A2**	-0.43	0.74	4.67E-08	2.98E-07
**LRRC28**	0.67	1.59	4.79E-08	3.06E-07
**MYO19**	-0.38	0.77	4.81E-08	3.07E-07
**IBTK**	-0.47	0.72	4.82E-08	3.08E-07
**SOD2**	-0.42	0.75	4.92E-08	3.14E-07
**U2AF2**	-0.38	0.77	4.92E-08	3.14E-07
**ATIC**	-0.37	0.78	4.96E-08	3.16E-07
**ALDH2**	0.95	1.93	4.99E-08	3.18E-07
**PARD3**	-0.42	0.74	5.04E-08	3.21E-07
**ELOVL5**	-0.41	0.75	5.09E-08	3.24E-07
**FAM83H**	-0.34	0.79	5.24E-08	3.33E-07
**SLC6A9**	-1.04	0.49	5.30E-08	3.37E-07
**C11orf68**	0.51	1.43	5.31E-08	3.38E-07
**PAK4**	-0.43	0.74	5.38E-08	3.42E-07
**BEX2**	1.29	2.45	5.39E-08	3.43E-07
**YEATS2**	-0.41	0.75	5.40E-08	3.43E-07
**BORCS7**	1.06	2.09	5.41E-08	3.44E-07
**HAGHL**	-1.21	0.43	5.45E-08	3.46E-07
**NUP188**	-0.32	0.80	5.45E-08	3.46E-07
**PALMD**	-0.73	0.60	5.54E-08	3.52E-07
**KDM2B**	-0.51	0.70	5.55E-08	3.52E-07

**MCF2**	1.19	2.28	5.58E-08	3.54E-07
**ERCC1**	0.42	1.34	5.59E-08	3.54E-07
**MTFR1**	-0.53	0.69	5.66E-08	3.59E-07
**CCDC64**	-0.63	0.64	5.68E-08	3.60E-07
**AKR1B15**	1.80	3.48	5.69E-08	3.60E-07
**DCK**	-0.68	0.63	5.72E-08	3.62E-07
**DOCK3**	1.25	2.38	5.75E-08	3.64E-07
**LZTS1**	-1.28	0.41	5.81E-08	3.68E-07
**MBOAT2**	-0.46	0.73	5.87E-08	3.71E-07
**PLEKHA1**	0.43	1.35	5.90E-08	3.73E-07
**CRTC1**	0.69	1.61	5.91E-08	3.73E-07
**CCDC80**	0.48	1.40	5.91E-08	3.73E-07
**CXADR**	0.60	1.51	5.94E-08	3.75E-07
**PDCD11**	-0.36	0.78	5.95E-08	3.76E-07
**RP11-649A18.12**	-2.23	0.21	5.95E-08	3.76E-07
**CFAP74**	2.30	4.93	5.97E-08	3.77E-07
**MYO1G**	2.15	4.43	6.03E-08	3.80E-07
**RFC1**	-0.41	0.75	6.05E-08	3.81E-07
**B3GALNT1**	-1.11	0.46	6.05E-08	3.81E-07
**TUBB6**	0.29	1.22	6.16E-08	3.88E-07
**ITGB1**	-0.30	0.81	6.23E-08	3.93E-07
**SLC35C1**	-0.49	0.71	6.31E-08	3.98E-07
**AC013461.1**	-0.39	0.76	6.32E-08	3.98E-07
**ELK1**	-0.50	0.71	6.51E-08	4.09E-07
**ACTR3**	-0.30	0.81	6.57E-08	4.13E-07
**CNTROB**	-0.46	0.73	6.58E-08	4.14E-07
**TBCE**	-0.59	0.66	6.64E-08	4.17E-07
**RP11-443P15.2**	-1.57	0.34	6.64E-08	4.17E-07
**FOXP1**	-0.53	0.69	6.67E-08	4.19E-07
**RNH1**	-0.36	0.78	6.74E-08	4.23E-07
**SMTN**	0.35	1.27	6.75E-08	4.24E-07
**C10orf2**	-0.61	0.66	6.76E-08	4.24E-07
**PLA2G4A**	-0.62	0.65	6.77E-08	4.25E-07
**ADNP**	-0.35	0.79	6.77E-08	4.25E-07
**C9orf72**	1.01	2.01	6.78E-08	4.25E-07
**MAPKAPK3**	-0.35	0.78	6.79E-08	4.25E-07
**RPS27**	0.31	1.24	6.80E-08	4.26E-07
**NARS**	-0.32	0.80	6.87E-08	4.30E-07
**ERMARD**	-0.72	0.61	6.92E-08	4.33E-07
**GRWD1**	-0.44	0.74	6.94E-08	4.34E-07
**GHITM**	0.34	1.27	6.97E-08	4.36E-07
**FDFT1**	-0.43	0.74	6.98E-08	4.36E-07
**SESN3**	0.63	1.55	7.00E-08	4.37E-07
**FAM173A**	-1.08	0.47	7.02E-08	4.39E-07
**DSCC1**	-0.87	0.55	7.21E-08	4.50E-07
**KHSRP**	-0.35	0.79	7.28E-08	4.55E-07
**RFK**	0.68	1.60	7.29E-08	4.55E-07
**FAM171A1**	-0.73	0.60	7.40E-08	4.62E-07
**UTP11L**	-0.43	0.74	7.40E-08	4.62E-07
**AGPAT1**	0.52	1.44	7.42E-08	4.63E-07
**ELFN1**	2.27	4.83	7.49E-08	4.67E-07
**REEP3**	-0.41	0.75	7.51E-08	4.68E-07
**GNPNAT1**	-0.56	0.68	7.53E-08	4.69E-07
**RPL4**	-0.29	0.82	7.60E-08	4.73E-07
**ATP8B2**	0.47	1.39	7.64E-08	4.76E-07
**KHNYN**	0.36	1.29	7.66E-08	4.77E-07
**THBS1**	-0.68	0.62	7.76E-08	4.83E-07
**ZDHHC18**	-0.50	0.71	7.89E-08	4.91E-07
**ADGRA3**	-0.43	0.74	8.01E-08	4.98E-07
**SUPT16H**	-0.31	0.80	8.03E-08	4.99E-07
**POLR2L**	0.42	1.33	8.04E-08	4.99E-07
**HES4**	-0.96	0.51	8.06E-08	5.00E-07
**IRF6**	0.29	1.22	8.11E-08	5.03E-07
**SLC4A3**	0.62	1.53	8.19E-08	5.08E-07
**FAM120B**	0.45	1.36	8.21E-08	5.09E-07
**BRD7**	-0.41	0.75	8.22E-08	5.10E-07
**KIAA1033**	0.41	1.33	8.23E-08	5.10E-07
**SLFN11**	-2.26	0.21	8.28E-08	5.13E-07
**DYRK3**	0.70	1.63	8.31E-08	5.14E-07
**SPC24**	-0.67	0.63	8.34E-08	5.16E-07
**GSDMB**	1.36	2.57	8.37E-08	5.18E-07
**PIK3R3**	0.79	1.72	8.39E-08	5.19E-07
**UBE4A**	0.40	1.32	8.39E-08	5.19E-07
**ELMOD3**	0.65	1.57	8.48E-08	5.24E-07
**RPH3AL**	1.69	3.23	8.49E-08	5.25E-07
**CCNB1IP1**	-0.56	0.68	8.65E-08	5.34E-07
**ECH1**	0.44	1.36	8.68E-08	5.36E-07
**FBXO17**	-1.00	0.50	8.73E-08	5.39E-07
**C7orf43**	0.65	1.57	8.74E-08	5.39E-07
**STIP1**	-0.30	0.81	8.76E-08	5.40E-07
**NEBL**	-0.50	0.71	8.82E-08	5.44E-07
**TAX1BP1**	0.42	1.34	8.85E-08	5.46E-07
**TLN2**	-0.69	0.62	8.87E-08	5.47E-07
**MDK**	0.66	1.58	8.97E-08	5.53E-07
**SETD2**	-0.37	0.77	9.10E-08	5.60E-07
**HIF1AN**	0.38	1.30	9.17E-08	5.64E-07
**SSNA1**	-0.46	0.73	9.20E-08	5.66E-07
**MST1R**	-0.40	0.76	9.21E-08	5.66E-07
**UBALD2**	-0.46	0.73	9.22E-08	5.67E-07
**RPS20**	0.31	1.24	9.23E-08	5.68E-07

**PDXK**	-0.33	0.80	9.47E-08	5.82E-07
**CATSPERG**	2.02	4.06	9.48E-08	5.82E-07
**FAR1**	-0.43	0.74	9.49E-08	5.83E-07
**C16orf62**	0.46	1.38	9.50E-08	5.83E-07
**PHLDA2**	0.43	1.35	9.60E-08	5.89E-07
**BAG2**	-1.39	0.38	9.61E-08	5.89E-07
**FARP1**	-0.73	0.60	9.67E-08	5.93E-07
**C5orf15**	0.48	1.39	9.70E-08	5.95E-07
**KAT5**	-0.62	0.65	9.71E-08	5.95E-07
**SF1**	-0.42	0.75	9.88E-08	6.06E-07
**WLS**	0.36	1.28	1.00E-07	6.12E-07
**TMEM38A**	1.22	2.33	1.01E-07	6.16E-07
**ERC1**	-0.36	0.78	1.01E-07	6.17E-07
**KLHL25**	0.80	1.74	1.02E-07	6.22E-07
**GTPBP2**	-0.42	0.75	1.04E-07	6.33E-07
**PKN3**	-0.87	0.55	1.04E-07	6.36E-07
**RPL5**	-0.33	0.80	1.05E-07	6.42E-07
**BAIAP2**	-0.34	0.79	1.06E-07	6.46E-07
**AP2M1**	0.28	1.22	1.06E-07	6.48E-07
**VPS9D1-AS1**	-0.83	0.56	1.06E-07	6.49E-07
**ZBTB43**	0.57	1.48	1.07E-07	6.51E-07
**TGFBR2**	-0.36	0.78	1.07E-07	6.52E-07
**CDC45**	-0.58	0.67	1.07E-07	6.52E-07
**ZMYND11**	-0.39	0.77	1.08E-07	6.57E-07
**TROAP**	-1.07	0.48	1.08E-07	6.57E-07
**PTPN2**	-0.51	0.70	1.09E-07	6.62E-07
**KDELR1**	-0.35	0.78	1.09E-07	6.63E-07
**NEK8**	1.14	2.20	1.09E-07	6.63E-07
**CD276**	-0.44	0.74	1.09E-07	6.66E-07
**RP5-1039K5.12**	1.05	2.07	1.10E-07	6.69E-07
**DDA1**	0.45	1.37	1.10E-07	6.71E-07
**CHPF2**	0.50	1.41	1.11E-07	6.76E-07
**EID1**	0.60	1.51	1.13E-07	6.87E-07
**IFT172**	-0.74	0.60	1.13E-07	6.88E-07
**NMRK1**	0.90	1.87	1.13E-07	6.88E-07
**MIR4435-2HG**	0.54	1.46	1.14E-07	6.91E-07
**HLCS**	-0.57	0.67	1.14E-07	6.93E-07
**CDH1**	0.26	1.19	1.15E-07	6.99E-07
**MPST**	-0.49	0.71	1.15E-07	7.00E-07
**ID4**	-1.09	0.47	1.17E-07	7.08E-07
**SH3D19**	-0.47	0.72	1.17E-07	7.09E-07
**MANEA**	-0.68	0.63	1.17E-07	7.11E-07
**MGA**	-0.49	0.71	1.19E-07	7.19E-07
**RP5-965G21.6**	-1.80	0.29	1.20E-07	7.26E-07
**ARRDC3**	0.48	1.39	1.20E-07	7.28E-07
**AC144652.1**	-2.04	0.24	1.21E-07	7.32E-07
**LCE1F**	2.23	4.70	1.22E-07	7.39E-07
**HSPA2**	-0.65	0.64	1.23E-07	7.43E-07
**MRPL49**	0.37	1.29	1.23E-07	7.44E-07
**THBD**	0.30	1.23	1.24E-07	7.48E-07
**LRSAM1**	0.55	1.47	1.25E-07	7.58E-07
**HNRNPC**	-0.27	0.83	1.26E-07	7.63E-07
**ARHGAP18**	-0.53	0.69	1.27E-07	7.68E-07
**CCK**	1.92	3.78	1.28E-07	7.70E-07
**NDUFAF4**	-0.71	0.61	1.29E-07	7.77E-07
**SGPL1**	-0.41	0.75	1.29E-07	7.77E-07
**TIMELESS**	-0.41	0.75	1.29E-07	7.78E-07
**APPL2**	0.49	1.40	1.30E-07	7.84E-07
**LCOR**	0.58	1.49	1.31E-07	7.89E-07
**TOM1L2**	0.38	1.30	1.33E-07	8.00E-07
**CALB2**	-1.18	0.44	1.33E-07	8.01E-07
**FADS1**	0.51	1.43	1.34E-07	8.07E-07
**SEL1L3**	-0.46	0.73	1.35E-07	8.14E-07
**SAMD4A**	-0.61	0.66	1.40E-07	8.40E-07
**CENPH**	-0.54	0.69	1.40E-07	8.42E-07
**SRP72**	-0.34	0.79	1.42E-07	8.53E-07
**KIAA1524**	-0.51	0.70	1.42E-07	8.55E-07
**CENPN**	-0.64	0.64	1.43E-07	8.61E-07
**GPC4**	1.80	3.47	1.43E-07	8.61E-07
**PGM1**	0.40	1.32	1.44E-07	8.65E-07
**BCS1L**	-0.50	0.71	1.44E-07	8.65E-07
**PICALM**	0.33	1.25	1.46E-07	8.78E-07
**PISD**	0.45	1.36	1.47E-07	8.79E-07
**TPD52L1**	-0.40	0.76	1.47E-07	8.80E-07
**PRRC1**	-0.41	0.76	1.47E-07	8.82E-07
**AKAP9**	0.51	1.43	1.48E-07	8.84E-07
**BBS4**	0.68	1.60	1.48E-07	8.86E-07
**GMPR2**	0.40	1.32	1.48E-07	8.88E-07
**CNIH1**	-0.37	0.77	1.49E-07	8.92E-07
**TMEM107**	0.68	1.60	1.49E-07	8.94E-07
**SLC25A45**	1.66	3.15	1.50E-07	8.95E-07
**GPI**	-0.33	0.79	1.50E-07	8.97E-07
**ZRANB2**	0.42	1.34	1.51E-07	9.00E-07
**FAM71E1**	1.79	3.45	1.52E-07	9.08E-07
**LYRM9**	1.45	2.73	1.53E-07	9.14E-07
**USP30**	0.75	1.68	1.54E-07	9.18E-07
**SPATA33**	-0.77	0.59	1.55E-07	9.25E-07
**NPBWR1**	2.23	4.70	1.55E-07	9.26E-07
**TRPS1**	-0.85	0.56	1.57E-07	9.33E-07
**CEACAM1**	0.66	1.58	1.57E-07	9.37E-07

**HYAL1**	0.67	1.59	1.59E-07	9.50E-07
**TDG**	0.47	1.39	1.60E-07	9.53E-07
**NDUFS5**	0.33	1.26	1.61E-07	9.62E-07
**MRPL15**	-0.42	0.75	1.63E-07	9.71E-07
**RASGRF1**	1.94	3.84	1.66E-07	9.87E-07
**CKMT1A**	-0.60	0.66	1.67E-07	9.94E-07
**PDE2A**	1.39	2.62	1.68E-07	9.99E-07
**DIP2A**	0.38	1.31	1.69E-07	1.00E-06
**HNRNPUL2**	-0.42	0.75	1.69E-07	1.00E-06
**CORO2A**	0.36	1.28	1.69E-07	1.00E-06
**NPL**	0.97	1.96	1.71E-07	1.01E-06
**VEGFC**	-0.56	0.68	1.73E-07	1.03E-06
**FKBP11**	-0.73	0.60	1.74E-07	1.03E-06
**RNF25**	0.57	1.49	1.75E-07	1.04E-06
**PCMTD1**	0.76	1.69	1.77E-07	1.05E-06
**CHI3L2**	1.85	3.61	1.77E-07	1.05E-06
**ERLIN1**	-0.36	0.78	1.80E-07	1.07E-06
**MIR503HG**	1.16	2.24	1.80E-07	1.07E-06
**ZNF217**	0.40	1.32	1.80E-07	1.07E-06
**TMX4**	0.55	1.47	1.81E-07	1.07E-06
**RB1**	0.48	1.40	1.81E-07	1.07E-06
**CEBPZ**	-0.47	0.72	1.84E-07	1.09E-06
**ENOX2**	-0.53	0.69	1.85E-07	1.09E-06
**APTX**	0.39	1.31	1.85E-07	1.09E-06
**PFKL**	0.32	1.25	1.85E-07	1.10E-06
**COL2A1**	2.22	4.65	1.86E-07	1.10E-06
**ATG4B**	0.40	1.32	1.87E-07	1.10E-06
**FAM50B**	-1.01	0.50	1.89E-07	1.11E-06
**C4B**	2.16	4.46	1.89E-07	1.11E-06
**EDEM1**	0.39	1.31	1.92E-07	1.13E-06
**DOPEY2**	0.50	1.42	1.92E-07	1.13E-06
**SCAP**	0.37	1.29	1.94E-07	1.14E-06
**LYRM5**	0.86	1.82	1.96E-07	1.15E-06
**SLC4A7**	-0.52	0.70	1.96E-07	1.16E-06
**MET**	-0.34	0.79	1.99E-07	1.17E-06
**NFATC1**	-1.26	0.42	1.99E-07	1.17E-06
**MTMR10**	0.49	1.40	2.00E-07	1.18E-06
**ATAD5**	-0.75	0.59	2.00E-07	1.18E-06
**RP11-83N9.5**	2.13	4.36	2.02E-07	1.19E-06
**GTF2A2**	0.44	1.36	2.03E-07	1.19E-06
**NR2C2AP**	-0.72	0.61	2.04E-07	1.20E-06
**CYB5D2**	0.80	1.74	2.06E-07	1.21E-06
**MAP2**	-0.38	0.77	2.06E-07	1.21E-06
**OS9**	0.34	1.27	2.07E-07	1.22E-06
**PAQR8**	-1.28	0.41	2.08E-07	1.22E-06
**TRIP10**	-0.35	0.78	2.10E-07	1.23E-06
**PTRF**	-0.30	0.81	2.11E-07	1.24E-06
**HSBP1**	0.37	1.29	2.11E-07	1.24E-06
**TP53TG1**	0.88	1.84	2.12E-07	1.24E-06
**ANK2**	-1.53	0.35	2.12E-07	1.24E-06
**TMEM181**	-0.44	0.74	2.13E-07	1.25E-06
**RND3**	0.38	1.30	2.14E-07	1.25E-06
**AC005083.1**	1.57	2.98	2.15E-07	1.26E-06
**MED8**	-0.48	0.72	2.17E-07	1.27E-06
**HMG20B**	-0.43	0.74	2.18E-07	1.28E-06
**GPR143**	1.55	2.93	2.23E-07	1.30E-06
**MANBAL**	-0.43	0.74	2.23E-07	1.30E-06
**FMNL2**	-0.43	0.74	2.25E-07	1.31E-06
**DUOX2**	1.05	2.07	2.28E-07	1.33E-06
**FLNB**	-0.28	0.83	2.30E-07	1.35E-06
**MAMDC4**	1.04	2.06	2.31E-07	1.35E-06
**CD3EAP**	-0.96	0.51	2.31E-07	1.35E-06
**BCL7B**	-0.48	0.72	2.32E-07	1.35E-06
**ADORA2B**	-0.54	0.69	2.33E-07	1.36E-06
**MAP2K3**	0.41	1.33	2.34E-07	1.36E-06
**BID**	-0.49	0.71	2.34E-07	1.37E-06
**TRIM4**	0.46	1.38	2.35E-07	1.37E-06
**C7orf50**	-0.38	0.77	2.35E-07	1.37E-06
**PRSS8**	-0.37	0.77	2.36E-07	1.38E-06
**GPR153**	0.45	1.36	2.37E-07	1.38E-06
**CMSS1**	-0.48	0.72	2.37E-07	1.38E-06
**C18orf25**	0.43	1.35	2.37E-07	1.38E-06
**WDR45**	0.50	1.41	2.39E-07	1.39E-06
**CARHSP1**	-0.52	0.70	2.41E-07	1.40E-06
**RP11-235E17.6**	2.11	4.33	2.42E-07	1.41E-06
**MGAT4B**	-0.38	0.77	2.43E-07	1.41E-06
**TRIM56**	-0.43	0.74	2.43E-07	1.41E-06
**ARNTL**	0.51	1.42	2.43E-07	1.41E-06
**AHCYL1**	0.31	1.24	2.44E-07	1.42E-06
**DDX26B**	0.73	1.66	2.47E-07	1.43E-06
**RRAGA**	0.40	1.32	2.48E-07	1.44E-06
**USP36**	-0.45	0.73	2.49E-07	1.45E-06
**STK17A**	0.42	1.34	2.50E-07	1.45E-06
**NPEPPS**	0.40	1.32	2.51E-07	1.46E-06
**LIX1L**	-0.71	0.61	2.54E-07	1.47E-06
**FAM98A**	-0.35	0.78	2.55E-07	1.48E-06
**PPP6R2**	0.41	1.33	2.57E-07	1.49E-06
**FER1L4**	2.07	4.21	2.58E-07	1.49E-06
**NSUN5**	-0.57	0.67	2.59E-07	1.50E-06
**LPP**	-0.50	0.71	2.62E-07	1.51E-06

**TRAPPC6B**	0.50	1.41	2.64E-07	1.53E-06
**D2HGDH**	0.63	1.55	2.64E-07	1.53E-06
**NUDT16L1**	-0.56	0.68	2.66E-07	1.54E-06
**PCNP**	0.41	1.33	2.67E-07	1.54E-06
**DDOST**	0.29	1.23	2.68E-07	1.55E-06
**TMEM171**	-1.06	0.48	2.69E-07	1.56E-06
**NASP**	-0.35	0.79	2.70E-07	1.56E-06
**ADSS**	-0.44	0.74	2.71E-07	1.56E-06
**TUSC2**	0.50	1.41	2.73E-07	1.57E-06
**SFR1**	-0.96	0.51	2.73E-07	1.57E-06
**AP4S1**	0.89	1.86	2.77E-07	1.60E-06
**PIGO**	-0.45	0.73	2.77E-07	1.60E-06
**PDIA3**	-0.31	0.81	2.78E-07	1.60E-06
**ARVCF**	0.94	1.92	2.80E-07	1.61E-06
**HDAC4**	-0.57	0.68	2.80E-07	1.61E-06
**MMP28**	0.66	1.59	2.80E-07	1.61E-06
**CORO6**	1.07	2.09	2.84E-07	1.64E-06
**ST6GALNAC2**	0.45	1.37	2.87E-07	1.65E-06
**RNF20**	-0.44	0.74	2.88E-07	1.65E-06
**APPL1**	-0.41	0.75	2.94E-07	1.69E-06
**STK32B**	1.20	2.30	2.95E-07	1.70E-06
**SCOC**	0.53	1.44	2.96E-07	1.70E-06
**HIRIP3**	-0.59	0.67	2.96E-07	1.70E-06
**COG5**	-0.39	0.76	2.96E-07	1.70E-06
**ZP3**	1.07	2.10	2.97E-07	1.71E-06
**LINC01376**	2.12	4.36	2.98E-07	1.71E-06
**SOCS3**	1.11	2.15	2.98E-07	1.71E-06
**TGFBRAP1**	0.41	1.33	3.00E-07	1.72E-06
**OSBPL3**	0.38	1.30	3.01E-07	1.73E-06
**NUFIP1**	-0.66	0.63	3.02E-07	1.73E-06
**IRX3**	-0.54	0.69	3.03E-07	1.74E-06
**CNTLN**	-0.86	0.55	3.04E-07	1.74E-06
**DGKH**	-0.61	0.66	3.05E-07	1.75E-06
**KIAA1586**	0.80	1.74	3.10E-07	1.77E-06
**SLC2A13**	1.24	2.36	3.11E-07	1.78E-06
**UMAD1**	0.76	1.69	3.12E-07	1.78E-06
**CACHD1**	-0.99	0.50	3.12E-07	1.78E-06
**PSMB4**	0.30	1.23	3.13E-07	1.79E-06
**BTG1**	0.48	1.40	3.13E-07	1.79E-06
**USP10**	-0.34	0.79	3.15E-07	1.80E-06
**C6orf48**	0.44	1.36	3.15E-07	1.80E-06
**RP11-521B24.4**	-1.54	0.34	3.18E-07	1.82E-06
**LAYN**	0.86	1.82	3.18E-07	1.82E-06
**GAR1**	-0.61	0.65	3.20E-07	1.83E-06
**KRT23**	-0.53	0.69	3.23E-07	1.84E-06
**ESD**	-0.37	0.78	3.23E-07	1.84E-06
**DDX3Y**	0.39	1.31	3.24E-07	1.84E-06
**GSPT1**	-0.29	0.82	3.26E-07	1.86E-06
**C5orf30**	-0.79	0.58	3.28E-07	1.87E-06
**FAM222B**	-0.41	0.75	3.28E-07	1.87E-06
**SELPLG**	1.74	3.33	3.29E-07	1.87E-06
**ANKRD27**	-0.44	0.74	3.43E-07	1.95E-06
**CFDP1**	-0.56	0.68	3.44E-07	1.96E-06
**DBF4B**	-0.68	0.62	3.47E-07	1.97E-06
**NACAD**	1.48	2.79	3.49E-07	1.99E-06
**THOP1**	-0.37	0.77	3.52E-07	2.00E-06
**SLC16A14**	0.99	1.99	3.52E-07	2.00E-06
**SPAG7**	-0.45	0.73	3.53E-07	2.01E-06
**RQCD1**	-0.36	0.78	3.55E-07	2.01E-06
**TGIF1**	0.43	1.35	3.57E-07	2.03E-06
**RORC**	-1.96	0.26	3.59E-07	2.04E-06
**C10orf54**	0.38	1.30	3.60E-07	2.04E-06
**TCEB3**	0.34	1.27	3.60E-07	2.04E-06
**MAP3K9**	0.38	1.30	3.61E-07	2.04E-06
**ARHGAP21**	-0.35	0.79	3.64E-07	2.06E-06
**SKA2**	-0.49	0.71	3.66E-07	2.07E-06
**ARPC1A**	-0.29	0.82	3.68E-07	2.08E-06
**ATP6AP1**	0.40	1.32	3.68E-07	2.08E-06
**RP11-7F17.7**	2.13	4.38	3.70E-07	2.09E-06
**TRAFD1**	0.41	1.33	3.70E-07	2.09E-06
**ARSD**	0.74	1.67	3.72E-07	2.10E-06
**RGP1**	0.37	1.29	3.73E-07	2.11E-06
**PDCD5**	-0.37	0.77	3.76E-07	2.13E-06
**EHMT1**	-0.38	0.77	3.84E-07	2.17E-06
**ZNF783**	-0.69	0.62	3.87E-07	2.19E-06
**ARID1A**	-0.37	0.77	3.87E-07	2.19E-06
**CASK**	0.41	1.33	3.89E-07	2.20E-06
**TMUB2**	0.55	1.46	3.94E-07	2.23E-06
**EIF3L**	-0.32	0.80	3.95E-07	2.23E-06
**PARPBP**	-0.82	0.57	3.96E-07	2.23E-06
**MX2**	1.26	2.40	4.01E-07	2.26E-06
**CSRNP2**	0.51	1.42	4.02E-07	2.26E-06
**KDELR3**	-0.76	0.59	4.07E-07	2.29E-06
**C6orf106**	0.30	1.23	4.08E-07	2.30E-06
**DCAF5**	0.37	1.29	4.08E-07	2.30E-06
**ROBO1**	-0.40	0.76	4.13E-07	2.32E-06
**ATAT1**	0.93	1.91	4.13E-07	2.32E-06
**FAM65B**	2.14	4.42	4.18E-07	2.35E-06
**GRTP1**	0.68	1.60	4.22E-07	2.37E-06
**VPS16**	0.58	1.50	4.29E-07	2.41E-06

**HSPA1A**	-0.54	0.69	4.31E-07	2.42E-06
**RPGRIP1L**	-0.62	0.65	4.33E-07	2.43E-06
**NAA25**	-0.45	0.73	4.33E-07	2.43E-06
**DMBT1**	2.10	4.28	4.39E-07	2.47E-06
**TBCD**	-0.35	0.79	4.40E-07	2.47E-06
**AP3S1**	0.37	1.30	4.40E-07	2.47E-06
**SLC5A3**	0.55	1.46	4.42E-07	2.48E-06
**SKIV2L**	0.40	1.32	4.44E-07	2.49E-06
**CTD-2015G9.2**	-1.54	0.34	4.46E-07	2.50E-06
**SLC13A5**	1.40	2.63	4.46E-07	2.50E-06
**DUOX1**	0.36	1.28	4.48E-07	2.51E-06
**TGFA**	0.31	1.24	4.49E-07	2.51E-06
**MACF1**	-0.74	0.60	4.49E-07	2.51E-06
**ZNF563**	1.19	2.29	4.53E-07	2.53E-06
**KDM6A**	0.50	1.41	4.53E-07	2.53E-06
**TINCR**	0.39	1.31	4.59E-07	2.57E-06
**FAM91A1**	0.38	1.30	4.59E-07	2.57E-06
**C4orf46**	-0.67	0.63	4.60E-07	2.57E-06
**PI4KB**	0.35	1.28	4.70E-07	2.63E-06
**HES5**	-1.21	0.43	4.79E-07	2.67E-06
**THG1L**	-0.73	0.60	4.79E-07	2.68E-06
**PLD2**	-0.38	0.77	4.80E-07	2.68E-06
**DIRC2**	0.59	1.51	4.81E-07	2.68E-06
**UBA52**	0.29	1.22	4.81E-07	2.68E-06
**ZBTB7A**	-0.35	0.78	4.83E-07	2.69E-06
**ACO2**	0.34	1.26	4.87E-07	2.72E-06
**MPP3**	-1.02	0.49	4.88E-07	2.72E-06
**CNTNAP2**	-0.93	0.53	4.89E-07	2.72E-06
**ASPG**	1.36	2.57	4.93E-07	2.74E-06
**DEFB1**	1.01	2.01	4.96E-07	2.76E-06
**BRPF3**	0.45	1.37	4.96E-07	2.76E-06
**CUTC**	-0.70	0.61	4.97E-07	2.76E-06
**TTC9**	-0.44	0.74	5.04E-07	2.80E-06
**SEC31B**	1.63	3.09	5.09E-07	2.83E-06
**SRPRB**	-0.46	0.73	5.12E-07	2.85E-06
**SCYL1**	-0.40	0.76	5.13E-07	2.85E-06
**FAM65A**	-0.59	0.67	5.14E-07	2.85E-06
**CNTN1**	-0.39	0.76	5.19E-07	2.88E-06
**COQ10B**	0.63	1.54	5.20E-07	2.89E-06
**L3MBTL1**	1.27	2.41	5.25E-07	2.92E-06
**IQCA1**	0.77	1.70	5.28E-07	2.93E-06
**PLBD2**	0.40	1.32	5.41E-07	3.00E-06
**RAB1A**	0.34	1.26	5.44E-07	3.02E-06
**C21orf2**	1.16	2.23	5.45E-07	3.02E-06
**ATP5J**	0.41	1.33	5.50E-07	3.05E-06
**SEC61A1**	0.32	1.25	5.51E-07	3.05E-06
**GNB5**	0.54	1.45	5.52E-07	3.06E-06
**PRRC2C**	-0.28	0.82	5.55E-07	3.07E-06
**GRHPR**	0.33	1.26	5.60E-07	3.10E-06
**CCDC120**	0.41	1.33	5.62E-07	3.11E-06
**USP19**	0.42	1.33	5.62E-07	3.11E-06
**SNU13**	-0.32	0.80	5.66E-07	3.13E-06
**KLHL24**	1.04	2.05	5.72E-07	3.16E-06
**ZNF274**	0.50	1.42	5.73E-07	3.16E-06
**MVP**	0.36	1.28	5.73E-07	3.16E-06
**RFX7**	0.40	1.32	5.76E-07	3.18E-06
**SPRTN**	0.54	1.46	5.80E-07	3.20E-06
**LDHB**	-0.26	0.83	5.85E-07	3.23E-06
**SLC25A30**	0.50	1.41	5.89E-07	3.25E-06
**BSPRY**	-0.70	0.62	5.90E-07	3.25E-06
**TTF2**	-0.41	0.75	5.91E-07	3.26E-06
**ITPR3**	-0.29	0.82	6.10E-07	3.36E-06
**STPG1**	0.50	1.41	6.12E-07	3.37E-06
**CNNM4**	-0.53	0.69	6.14E-07	3.38E-06
**TCEA3**	1.09	2.12	6.14E-07	3.38E-06
**DBT**	0.46	1.37	6.19E-07	3.41E-06
**LINC00958**	1.45	2.73	6.21E-07	3.41E-06
**TET2**	0.45	1.37	6.27E-07	3.45E-06
**NR3C1**	-0.36	0.78	6.35E-07	3.49E-06
**ALDH18A1**	-0.35	0.79	6.40E-07	3.52E-06
**ARHGAP1**	0.39	1.31	6.43E-07	3.53E-06
**ILVBL**	-0.41	0.75	6.44E-07	3.54E-06
**ATP6V1E2**	-1.01	0.50	6.45E-07	3.54E-06
**CYS1**	2.11	4.31	6.49E-07	3.56E-06
**MBOAT7**	0.38	1.30	6.49E-07	3.56E-06
**CLUH**	-0.38	0.77	6.53E-07	3.58E-06
**RNPEPL1**	-0.48	0.72	6.54E-07	3.59E-06
**UQCRQ**	0.37	1.30	6.56E-07	3.60E-06
**PTPRA**	-0.36	0.78	6.61E-07	3.62E-06
**MYCN**	1.08	2.12	6.62E-07	3.63E-06
**CCDC9**	-0.66	0.63	6.63E-07	3.63E-06
**EFNA1**	-0.53	0.69	6.66E-07	3.65E-06
**FBXO28**	0.42	1.34	6.70E-07	3.67E-06
**TMCC3**	0.37	1.29	6.71E-07	3.67E-06
**KCNG1**	0.45	1.37	6.72E-07	3.67E-06
**SCYL3**	0.72	1.64	6.75E-07	3.69E-06
**USP24**	-0.38	0.77	6.83E-07	3.73E-06
**SELENBP1**	1.48	2.79	6.85E-07	3.74E-06
**NTHL1**	-0.80	0.57	6.85E-07	3.75E-06
**CRNN**	-0.87	0.55	6.94E-07	3.79E-06

**SOS1**	0.39	1.31	6.97E-07	3.80E-06
**SRPX**	1.17	2.25	7.02E-07	3.84E-06
**CMTR1**	0.38	1.30	7.11E-07	3.88E-06
**CTH**	-1.14	0.45	7.11E-07	3.88E-06
**DPAGT1**	-0.42	0.75	7.16E-07	3.91E-06
**KLHL9**	-0.49	0.71	7.19E-07	3.92E-06
**F5**	2.09	4.25	7.20E-07	3.92E-06
**TRAP1**	-0.37	0.77	7.21E-07	3.93E-06
**STAMBPL1**	-0.87	0.55	7.25E-07	3.95E-06
**WASF3**	-0.68	0.62	7.28E-07	3.96E-06
**SPOCK1**	1.63	3.10	7.32E-07	3.99E-06
**ABCC3**	0.42	1.34	7.38E-07	4.01E-06
**09-mar**	-0.78	0.58	7.49E-07	4.08E-06
**WRN**	-0.58	0.67	7.50E-07	4.08E-06
**ARPC1B**	-0.35	0.78	7.50E-07	4.08E-06
**KEAP1**	-0.38	0.77	7.52E-07	4.09E-06
**TEPP**	1.68	3.20	7.56E-07	4.11E-06
**CTD-2184D3.3**	2.02	4.05	7.57E-07	4.11E-06
**NUCB2**	-0.54	0.69	7.60E-07	4.13E-06
**CARD10**	-0.35	0.78	7.64E-07	4.15E-06
**RP11-9E17.1**	1.02	2.03	7.66E-07	4.16E-06
**PPP1R26**	-0.45	0.73	7.74E-07	4.20E-06
**PLAC8**	1.89	3.70	7.75E-07	4.21E-06
**CPNE8**	0.64	1.55	7.75E-07	4.21E-06
**EHF**	0.33	1.26	7.78E-07	4.22E-06
**SYAP1**	0.38	1.30	7.81E-07	4.23E-06
**C1RL**	0.63	1.55	7.81E-07	4.23E-06
**AL591893.1**	1.87	3.65	7.85E-07	4.25E-06
**LRG1**	0.72	1.65	7.88E-07	4.27E-06
**SLC44A5**	0.94	1.92	7.89E-07	4.27E-06
**ATPAF2**	0.56	1.47	7.95E-07	4.30E-06
**PHKG2**	0.63	1.54	7.97E-07	4.31E-06
**CDC42EP4**	0.36	1.28	8.02E-07	4.34E-06
**KAT6A**	-0.39	0.76	8.03E-07	4.34E-06
**OSCP1**	0.87	1.82	8.11E-07	4.38E-06
**FZR1**	-0.41	0.75	8.14E-07	4.40E-06
**CBX5**	-0.37	0.78	8.35E-07	4.51E-06
**GNPTG**	0.53	1.44	8.37E-07	4.52E-06
**SOX9**	0.35	1.27	8.40E-07	4.53E-06
**TMEM147**	-0.40	0.76	8.42E-07	4.54E-06
**IL17RC**	-0.59	0.66	8.45E-07	4.56E-06
**TRPC6**	-1.80	0.29	8.49E-07	4.58E-06
**MT-ND5**	0.90	1.86	8.50E-07	4.58E-06
**TMEM179B**	0.60	1.52	8.59E-07	4.63E-06
**RFC2**	-0.45	0.73	8.61E-07	4.64E-06
**DNPEP**	-0.43	0.74	8.61E-07	4.64E-06
**CLIP2**	0.67	1.60	8.64E-07	4.65E-06
**TTC12**	0.56	1.48	8.70E-07	4.68E-06
**CCDC136**	1.90	3.74	8.77E-07	4.72E-06
**EBNA1BP2**	-0.30	0.81	8.85E-07	4.76E-06
**MCTP1**	-1.52	0.35	8.91E-07	4.79E-06
**LAPTM4B**	0.34	1.27	8.93E-07	4.80E-06
**NFE2L3**	-0.71	0.61	8.93E-07	4.80E-06
**ANXA11**	-0.28	0.82	9.03E-07	4.85E-06
**ZSWIM4**	0.84	1.79	9.06E-07	4.87E-06
**HSPD1**	-0.27	0.83	9.09E-07	4.88E-06
**PAFAH1B1**	-0.34	0.79	9.10E-07	4.89E-06
**WDR11**	0.34	1.27	9.14E-07	4.91E-06
**FAM83A-AS1**	-0.97	0.51	9.24E-07	4.96E-06
**RAB21**	0.42	1.34	9.28E-07	4.98E-06
**CTD-2619J13.14**	0.80	1.74	9.31E-07	4.99E-06
**CYP4F2**	2.02	4.05	9.47E-07	5.07E-06
**TMA7**	0.65	1.57	9.50E-07	5.09E-06
**MORF4L1**	-0.36	0.78	9.50E-07	5.09E-06
**ASXL1**	-0.38	0.77	9.52E-07	5.10E-06
**TMEM161A**	0.50	1.42	9.53E-07	5.10E-06
**IFT122**	-0.68	0.62	9.64E-07	5.16E-06
**CIRH1A**	-0.36	0.78	9.67E-07	5.18E-06
**SLC1A6**	-1.53	0.35	9.68E-07	5.18E-06
**AHCTF1**	-0.43	0.74	9.72E-07	5.20E-06
**SLC25A4**	0.47	1.39	9.75E-07	5.21E-06
**MICB**	0.83	1.78	9.77E-07	5.22E-06
**TP53AIP1**	0.77	1.70	9.78E-07	5.23E-06
**RNF125**	-1.25	0.42	9.83E-07	5.25E-06
**LONP2**	0.32	1.25	9.89E-07	5.28E-06
**PRDX1**	0.28	1.21	9.91E-07	5.29E-06
**HAX1**	-0.35	0.78	1.01E-06	5.39E-06
**WFS1**	0.47	1.39	1.01E-06	5.39E-06
**KIAA1161**	-0.48	0.72	1.02E-06	5.43E-06
**TMA16**	-0.51	0.70	1.02E-06	5.45E-06
**MCRS1**	-0.46	0.73	1.02E-06	5.46E-06
**ARHGEF40**	0.60	1.52	1.02E-06	5.46E-06
**SRGAP3**	0.46	1.38	1.03E-06	5.49E-06
**MYBBP1A**	-0.40	0.76	1.04E-06	5.51E-06
**HOMEZ**	0.44	1.35	1.05E-06	5.57E-06
**PAIP2**	0.44	1.36	1.05E-06	5.58E-06
**ZNF525**	-0.77	0.59	1.05E-06	5.59E-06
**WNK1**	-0.27	0.83	1.05E-06	5.59E-06
**NUP35**	-0.60	0.66	1.07E-06	5.70E-06
**LIG3**	-0.41	0.75	1.08E-06	5.71E-06

**PLEKHJ1**	-0.47	0.72	1.08E-06	5.73E-06
**FAM126B**	0.51	1.42	1.08E-06	5.74E-06
**SH3BP4**	-0.34	0.79	1.10E-06	5.82E-06
**NDUFB9**	0.36	1.28	1.10E-06	5.84E-06
**POLQ**	-0.55	0.68	1.10E-06	5.85E-06
**SGMS1**	-0.50	0.71	1.11E-06	5.88E-06
**LARP4B**	-0.34	0.79	1.11E-06	5.89E-06
**DCST2**	1.93	3.82	1.12E-06	5.93E-06
**EZR**	0.25	1.19	1.12E-06	5.95E-06
**MED23**	0.47	1.39	1.12E-06	5.96E-06
**NEAT1**	0.81	1.75	1.13E-06	6.00E-06
**OSR2**	0.62	1.54	1.16E-06	6.13E-06
**GRB10**	-1.15	0.45	1.16E-06	6.14E-06
**CASP4**	-0.39	0.76	1.17E-06	6.18E-06
**TGM4**	1.30	2.46	1.18E-06	6.24E-06
**SLC10A7**	-0.78	0.58	1.19E-06	6.31E-06
**C11orf45**	0.84	1.79	1.19E-06	6.32E-06
**CTCF**	-0.36	0.78	1.21E-06	6.37E-06
**MARCO**	0.80	1.74	1.22E-06	6.43E-06
**ERBB2IP**	-0.38	0.77	1.22E-06	6.44E-06
**KMT2C**	-0.45	0.73	1.23E-06	6.48E-06
**LINC00963**	0.74	1.67	1.25E-06	6.58E-06
**GTDC1**	-0.72	0.61	1.25E-06	6.60E-06
**CTDSPL2**	-0.48	0.72	1.26E-06	6.67E-06
**AP5M1**	0.47	1.39	1.27E-06	6.68E-06
**ARID1B**	-0.41	0.75	1.27E-06	6.69E-06
**STK3**	0.43	1.35	1.27E-06	6.72E-06
**HLA-DOA**	2.06	4.18	1.28E-06	6.73E-06
**KCNC3**	1.39	2.62	1.30E-06	6.85E-06
**HMGCS1**	-0.86	0.55	1.32E-06	6.95E-06
**SECISBP2L**	0.49	1.40	1.32E-06	6.96E-06
**CBFA2T3**	1.60	3.02	1.33E-06	6.99E-06
**WDR27**	0.65	1.57	1.33E-06	7.01E-06
**PYROXD1**	-0.61	0.65	1.34E-06	7.05E-06
**ZNF790**	1.01	2.01	1.38E-06	7.23E-06
**FAM73A**	0.50	1.41	1.38E-06	7.24E-06
**TBC1D16**	-0.41	0.75	1.39E-06	7.32E-06
**PXDC1**	0.48	1.39	1.41E-06	7.40E-06
**CTB-25B13.12**	1.32	2.50	1.41E-06	7.42E-06
**AC003088.1**	2.05	4.13	1.41E-06	7.43E-06
**BUB3**	-0.29	0.82	1.42E-06	7.47E-06
**INPP4B**	-0.43	0.74	1.42E-06	7.47E-06
**WDR90**	-0.49	0.71	1.43E-06	7.49E-06
**ADGRB2**	1.52	2.87	1.43E-06	7.50E-06
**HTATSF1**	-0.41	0.75	1.43E-06	7.50E-06
**USB1**	0.38	1.30	1.43E-06	7.51E-06
**WWC1**	0.40	1.32	1.44E-06	7.54E-06
**DIAPH3**	-0.42	0.75	1.45E-06	7.58E-06
**PSMG3**	-0.54	0.69	1.46E-06	7.66E-06
**CCDC178**	2.05	4.15	1.46E-06	7.66E-06
**YIPF6**	0.47	1.38	1.47E-06	7.69E-06
**MCUR1**	-0.46	0.73	1.48E-06	7.74E-06
**LZIC**	-0.53	0.69	1.48E-06	7.76E-06
**MT-ND6**	0.84	1.79	1.48E-06	7.76E-06
**SLC22A15**	0.67	1.59	1.49E-06	7.81E-06
**PCNX**	0.38	1.30	1.49E-06	7.81E-06
**SDCCAG3**	-0.40	0.76	1.51E-06	7.89E-06
**PLEK2**	-0.34	0.79	1.51E-06	7.90E-06
**ITGA5**	-0.34	0.79	1.51E-06	7.90E-06
**IL1RL1**	2.03	4.07	1.52E-06	7.95E-06
**EEF1A1**	-0.23	0.85	1.53E-06	8.01E-06
**OPLAH**	-0.61	0.66	1.54E-06	8.01E-06
**HCFC2**	0.64	1.55	1.54E-06	8.04E-06
**GIPC1**	-0.29	0.82	1.54E-06	8.05E-06
**TTC14**	0.77	1.71	1.56E-06	8.12E-06
**EPB41L2**	-0.37	0.78	1.57E-06	8.18E-06
**NARS2**	-0.58	0.67	1.57E-06	8.20E-06
**B4GALT4**	-0.41	0.75	1.60E-06	8.31E-06
**RP11-1246C19.1**	1.64	3.11	1.60E-06	8.31E-06
**KNOP1**	-0.52	0.70	1.60E-06	8.32E-06
**CYTH2**	0.41	1.33	1.60E-06	8.32E-06
**SMC6**	-0.41	0.75	1.61E-06	8.37E-06
**USP25**	-0.44	0.74	1.61E-06	8.37E-06
**STK32C**	-1.26	0.42	1.62E-06	8.40E-06
**ATP11A**	-0.42	0.75	1.62E-06	8.41E-06
**PWWP2A**	-0.60	0.66	1.63E-06	8.46E-06
**EMC3**	0.39	1.31	1.63E-06	8.47E-06
**NICN1**	0.80	1.74	1.64E-06	8.50E-06
**MARK3**	-0.33	0.80	1.64E-06	8.53E-06
**NUDCD2**	-0.53	0.69	1.67E-06	8.64E-06
**MRPL12**	-0.56	0.68	1.67E-06	8.65E-06
**TJP3**	-0.89	0.54	1.67E-06	8.68E-06
**BTN3A1**	-1.08	0.47	1.68E-06	8.69E-06
**RAB32**	-0.67	0.63	1.68E-06	8.70E-06
**TSC1**	0.44	1.36	1.68E-06	8.73E-06
**ANO6**	-0.37	0.77	1.70E-06	8.79E-06
**SIGMAR1**	-0.37	0.77	1.70E-06	8.81E-06
**SAMHD1**	0.53	1.44	1.72E-06	8.89E-06
**DDX18**	-0.33	0.79	1.73E-06	8.94E-06
**NPRL3**	-0.45	0.73	1.73E-06	8.96E-06

**SURF2**	-0.54	0.69	1.74E-06	9.01E-06
**THEM4**	-0.67	0.63	1.74E-06	9.02E-06
**UBE2E2**	-0.47	0.72	1.76E-06	9.09E-06
**CLCN6**	-0.69	0.62	1.77E-06	9.14E-06
**FRS2**	0.48	1.40	1.78E-06	9.20E-06
**ROCK2**	-0.39	0.76	1.78E-06	9.22E-06
**RPN2**	0.26	1.20	1.79E-06	9.23E-06
**HDAC5**	-0.58	0.67	1.81E-06	9.33E-06
**ZNF566**	-1.26	0.42	1.82E-06	9.41E-06
**FBXO4**	-0.91	0.53	1.83E-06	9.43E-06
**NIP7**	-0.43	0.74	1.83E-06	9.44E-06
**DUSP7**	-0.31	0.81	1.83E-06	9.45E-06
**LARS**	-0.29	0.82	1.83E-06	9.46E-06
**LRBA**	-0.36	0.78	1.84E-06	9.47E-06
**MSTO1**	-0.68	0.62	1.86E-06	9.60E-06
**SSBP2**	-0.67	0.63	1.87E-06	9.65E-06
**LAPTM5**	1.64	3.13	1.88E-06	9.70E-06
**RBM18**	0.48	1.40	1.89E-06	9.74E-06
**KPNA1**	0.39	1.31	1.92E-06	9.90E-06
**DCTPP1**	-0.54	0.69	1.93E-06	9.91E-06
**AP2A1**	0.30	1.23	1.93E-06	9.91E-06
**IFIT3**	0.71	1.64	1.94E-06	9.98E-06
**AP2B1**	-0.25	0.84	1.94E-06	9.99E-06
**SERTAD3**	0.61	1.53	1.95E-06	1.00E-05
**EP400**	-0.39	0.76	1.95E-06	1.00E-05
**GSE1**	0.41	1.33	1.96E-06	1.01E-05
**MRPL4**	-0.43	0.74	1.97E-06	1.01E-05
**WDR47**	0.42	1.34	1.98E-06	1.02E-05
**MKKS**	-0.45	0.73	1.99E-06	1.02E-05
**PPP3CB**	0.42	1.34	2.02E-06	1.04E-05
**HACD1**	0.81	1.75	2.06E-06	1.06E-05
**RPN1**	-0.26	0.84	2.06E-06	1.06E-05
**MSH3**	-0.47	0.72	2.10E-06	1.08E-05
**BARX2**	-0.39	0.76	2.11E-06	1.08E-05
**EIF1AY**	0.52	1.43	2.12E-06	1.08E-05
**RAB8B**	0.59	1.51	2.13E-06	1.09E-05
**DDN**	-1.79	0.29	2.13E-06	1.09E-05
**TRAF5**	-1.24	0.42	2.14E-06	1.09E-05
**MYCBPAP**	1.96	3.89	2.15E-06	1.10E-05
**RCBTB2**	0.80	1.74	2.19E-06	1.12E-05
**BCL11B**	-0.46	0.73	2.21E-06	1.13E-05
**TMEM168**	0.46	1.37	2.26E-06	1.16E-05
**UBE2Q1**	0.35	1.27	2.27E-06	1.16E-05
**C1orf68**	2.01	4.03	2.28E-06	1.16E-05
**TOLLIP**	-0.33	0.80	2.32E-06	1.18E-05
**COQ2**	-0.50	0.70	2.32E-06	1.18E-05
**CASP9**	0.78	1.72	2.34E-06	1.19E-05
**FTSJ2**	0.40	1.32	2.34E-06	1.20E-05
**FLVCR2**	-1.58	0.33	2.36E-06	1.20E-05
**SLC29A4**	1.36	2.57	2.36E-06	1.20E-05
**FAM120A**	-0.25	0.84	2.37E-06	1.21E-05
**SLC44A3**	0.79	1.73	2.37E-06	1.21E-05
**LGALS9C**	-1.10	0.47	2.37E-06	1.21E-05
**MTA1**	-0.40	0.76	2.38E-06	1.21E-05
**ACADM**	0.46	1.37	2.38E-06	1.21E-05
**DEGS2**	0.94	1.92	2.38E-06	1.21E-05
**TXNRD3**	-0.86	0.55	2.39E-06	1.22E-05
**C17orf100**	1.18	2.27	2.39E-06	1.22E-05
**RTKN2**	-0.53	0.69	2.40E-06	1.22E-05
**MLH3**	-0.61	0.65	2.40E-06	1.22E-05
**MT-CYB**	0.91	1.88	2.40E-06	1.22E-05
**ERCC4**	0.61	1.53	2.43E-06	1.24E-05
**DYNLT1**	0.38	1.30	2.43E-06	1.24E-05
**RPS9**	-0.25	0.84	2.44E-06	1.24E-05
**TBC1D13**	0.49	1.40	2.44E-06	1.24E-05
**CCDC122**	1.05	2.07	2.49E-06	1.27E-05
**TMEM62**	-0.68	0.62	2.49E-06	1.27E-05
**NOL3**	-0.55	0.68	2.50E-06	1.27E-05
**MYL5**	1.18	2.26	2.50E-06	1.27E-05
**HSD17B12**	0.36	1.29	2.50E-06	1.27E-05
**EHD2**	-0.30	0.81	2.51E-06	1.27E-05
**PIGC**	-0.69	0.62	2.52E-06	1.28E-05
**HIST1H2BJ**	1.53	2.89	2.53E-06	1.28E-05
**BRD9**	-0.44	0.74	2.55E-06	1.29E-05
**DGCR8**	-0.50	0.71	2.57E-06	1.30E-05
**KRT80**	0.29	1.22	2.59E-06	1.31E-05
**TCTA**	0.63	1.55	2.60E-06	1.32E-05
**LRRC41**	0.31	1.24	2.61E-06	1.32E-05
**QSOX1**	0.27	1.21	2.61E-06	1.32E-05
**PAX8-AS1**	0.83	1.78	2.63E-06	1.33E-05
**SULT1E1**	-0.89	0.54	2.63E-06	1.33E-05
**HYAL3**	0.80	1.74	2.64E-06	1.34E-05
**LSM6**	-0.65	0.64	2.66E-06	1.34E-05
**CSAD**	0.89	1.85	2.66E-06	1.34E-05
**KIAA0586**	-0.46	0.73	2.67E-06	1.35E-05
**ME1**	-0.46	0.73	2.69E-06	1.36E-05
**OCIAD2**	0.33	1.26	2.70E-06	1.36E-05
**REL**	-0.66	0.63	2.71E-06	1.37E-05
**C19orf66**	0.61	1.53	2.72E-06	1.37E-05
**ARHGEF12**	-0.33	0.79	2.77E-06	1.40E-05

**COPA**	0.25	1.19	2.81E-06	1.42E-05
**MRPL32**	0.36	1.29	2.82E-06	1.42E-05
**THNSL2**	0.75	1.68	2.82E-06	1.42E-05
**VDR**	0.33	1.26	2.84E-06	1.43E-05
**SCAND1**	0.51	1.42	2.85E-06	1.43E-05
**ZDBF2**	0.81	1.76	2.85E-06	1.43E-05
**ARID2**	-0.43	0.74	2.85E-06	1.43E-05
**MT-CO2**	0.59	1.51	2.85E-06	1.44E-05
**SIAE**	0.46	1.38	2.87E-06	1.44E-05
**ITGB1BP1**	-0.34	0.79	2.90E-06	1.46E-05
**TAF1**	-0.42	0.75	2.98E-06	1.50E-05
**TSEN34**	0.36	1.28	3.05E-06	1.53E-05
**MTHFR**	0.53	1.44	3.05E-06	1.53E-05
**FUCA1**	0.58	1.49	3.06E-06	1.54E-05
**NFYB**	-0.54	0.69	3.07E-06	1.54E-05
**TOR1AIP1**	-0.37	0.77	3.08E-06	1.55E-05
**PJA1**	0.39	1.31	3.09E-06	1.55E-05
**PLXDC2**	-0.75	0.60	3.10E-06	1.55E-05
**EBPL**	-0.51	0.70	3.10E-06	1.55E-05
**MMS22L**	-0.52	0.70	3.11E-06	1.56E-05
**MORF4L2**	-0.25	0.84	3.12E-06	1.56E-05
**ME2**	-0.39	0.77	3.13E-06	1.57E-05
**USP49**	0.89	1.85	3.13E-06	1.57E-05
**AHDC1**	-0.36	0.78	3.14E-06	1.57E-05
**NPR2**	1.30	2.45	3.17E-06	1.59E-05
**ST6GALNAC4**	-0.67	0.63	3.18E-06	1.59E-05
**RP5-1148A21.3**	1.59	3.00	3.20E-06	1.60E-05
**HIST3H2A**	0.88	1.85	3.21E-06	1.61E-05
**POF1B**	-0.38	0.77	3.23E-06	1.62E-05
**RP11-67L2.2**	0.98	1.97	3.24E-06	1.62E-05
**SLC25A32**	-0.46	0.73	3.25E-06	1.62E-05
**EYA3**	0.41	1.32	3.27E-06	1.63E-05
**FAM20C**	-1.01	0.50	3.30E-06	1.65E-05
**MIEF2**	0.69	1.61	3.33E-06	1.66E-05
**CENPL**	-0.70	0.62	3.34E-06	1.67E-05
**PEG10**	-1.59	0.33	3.39E-06	1.69E-05
**B4GALNT4**	-1.58	0.34	3.44E-06	1.72E-05
**SATB1**	0.84	1.79	3.45E-06	1.72E-05
**CACNB3**	-0.50	0.70	3.45E-06	1.72E-05
**KIAA1671**	-0.33	0.79	3.48E-06	1.74E-05
**HOXA9**	0.47	1.39	3.49E-06	1.74E-05
**NEIL2**	-0.68	0.63	3.52E-06	1.75E-05
**UBE2E3**	-0.42	0.74	3.53E-06	1.76E-05
**TXN**	0.35	1.27	3.53E-06	1.76E-05
**WBP1**	1.18	2.26	3.54E-06	1.76E-05
**SIL1**	0.53	1.44	3.54E-06	1.76E-05
**PLEKHM2**	0.35	1.27	3.62E-06	1.80E-05
**PDCD2L**	-0.96	0.52	3.63E-06	1.81E-05
**F12**	-0.88	0.54	3.63E-06	1.81E-05
**MICALCL**	0.48	1.40	3.63E-06	1.81E-05
**ZSCAN4**	1.92	3.78	3.64E-06	1.81E-05
**AMDHD2**	-0.85	0.56	3.65E-06	1.82E-05
**MTO1**	0.40	1.32	3.66E-06	1.82E-05
**HIVEP2**	-0.40	0.76	3.67E-06	1.82E-05
**HMBS**	-0.42	0.75	3.67E-06	1.82E-05
**ARHGAP5-AS1**	1.20	2.31	3.72E-06	1.85E-05
**UNC13A**	1.92	3.77	3.73E-06	1.85E-05
**SLC7A4**	-1.52	0.35	3.75E-06	1.86E-05
**MMP19**	1.37	2.58	3.78E-06	1.87E-05
**FAM43A**	1.02	2.02	3.83E-06	1.90E-05
**FOXO3**	-0.48	0.72	3.84E-06	1.90E-05
**ZKSCAN2**	0.72	1.65	3.87E-06	1.92E-05
**SMC5**	-0.43	0.74	3.98E-06	1.97E-05
**CHTOP**	-0.35	0.79	4.00E-06	1.98E-05
**MGEA5**	0.30	1.23	4.01E-06	1.98E-05
**HEATR3**	-0.48	0.72	4.02E-06	1.99E-05
**ALOX12B**	-1.24	0.42	4.03E-06	2.00E-05
**PICK1**	0.43	1.34	4.04E-06	2.00E-05
**NXPH4**	-1.06	0.48	4.05E-06	2.00E-05
**SETX**	-0.38	0.77	4.05E-06	2.00E-05
**TCN1**	0.60	1.52	4.05E-06	2.00E-05
**TRIO**	-0.33	0.80	4.13E-06	2.04E-05
**FBXO18**	0.33	1.26	4.14E-06	2.05E-05
**ZGRF1**	-0.77	0.59	4.14E-06	2.05E-05
**YAP1**	0.29	1.22	4.15E-06	2.05E-05
**GDPD2**	-1.10	0.47	4.15E-06	2.05E-05
**RP11-417L14.1**	1.95	3.85	4.15E-06	2.05E-05
**ONECUT3**	-1.76	0.29	4.15E-06	2.05E-05
**FAM127A**	0.36	1.28	4.18E-06	2.06E-05
**DALRD3**	-0.65	0.64	4.19E-06	2.07E-05
**TMEM44**	-0.73	0.60	4.21E-06	2.07E-05
**UBXN4**	0.34	1.26	4.28E-06	2.11E-05
**HPS1**	0.44	1.36	4.31E-06	2.12E-05
**DNPH1**	-0.45	0.73	4.33E-06	2.13E-05
**PLXNA3**	0.43	1.34	4.35E-06	2.14E-05
**MPZL2**	-0.28	0.82	4.38E-06	2.16E-05
**LBH**	1.56	2.95	4.39E-06	2.16E-05
**KCTD12**	0.36	1.29	4.39E-06	2.16E-05
**DFFB**	-0.92	0.53	4.42E-06	2.18E-05
**TIMM44**	-0.45	0.73	4.43E-06	2.18E-05

**DHRS9**	-1.75	0.30	4.47E-06	2.20E-05
**PLA2G4E**	0.42	1.34	4.47E-06	2.20E-05
**MTX3**	0.41	1.33	4.48E-06	2.20E-05
**RELL2**	0.76	1.69	4.49E-06	2.20E-05
**GTF2A1**	-0.45	0.73	4.50E-06	2.21E-05
**CLASRP**	-0.42	0.74	4.56E-06	2.24E-05
**RAD54L**	-0.62	0.65	4.56E-06	2.24E-05
**RBFA**	-0.68	0.62	4.56E-06	2.24E-05
**C2orf81**	1.17	2.25	4.56E-06	2.24E-05
**ZNF449**	-0.96	0.51	4.60E-06	2.25E-05
**PER2**	-0.52	0.70	4.63E-06	2.27E-05
**PER1**	0.34	1.27	4.64E-06	2.27E-05
**HERC1**	-0.43	0.74	4.65E-06	2.28E-05
**IFIH1**	0.50	1.42	4.69E-06	2.30E-05
**FAAP100**	-0.44	0.74	4.70E-06	2.30E-05
**FAM46B**	0.32	1.24	4.71E-06	2.31E-05
**CERK**	-0.40	0.76	4.71E-06	2.31E-05
**BLVRA**	-0.55	0.68	4.72E-06	2.31E-05
**RP11-196G18.22**	1.31	2.49	4.78E-06	2.34E-05
**GOLGA8A**	0.76	1.69	4.79E-06	2.34E-05
**LOXL1-AS1**	-1.23	0.43	4.80E-06	2.35E-05
**DDX11**	-0.54	0.69	4.87E-06	2.38E-05
**FAM127B**	0.37	1.30	4.93E-06	2.41E-05
**ACER1**	-1.48	0.36	4.94E-06	2.41E-05
**TYRO3**	-0.47	0.72	4.98E-06	2.43E-05
**LHFPL2**	-0.44	0.74	4.98E-06	2.43E-05
**ACAD10**	0.53	1.44	4.98E-06	2.43E-05
**ZNF76**	0.52	1.44	5.00E-06	2.44E-05
**STK39**	-0.42	0.75	5.02E-06	2.45E-05
**SLC22A3**	-0.87	0.55	5.06E-06	2.47E-05
**RHOV**	-0.37	0.77	5.07E-06	2.47E-05
**ST6GALNAC6**	-0.49	0.71	5.09E-06	2.48E-05
**ABR**	-0.36	0.78	5.22E-06	2.54E-05
**KCTD1**	-0.32	0.80	5.22E-06	2.54E-05
**SLFN13**	-1.00	0.50	5.27E-06	2.56E-05
**SLC31A1**	-0.35	0.78	5.27E-06	2.56E-05
**PEX5**	-0.44	0.73	5.30E-06	2.58E-05
**OAZ2**	0.34	1.26	5.31E-06	2.58E-05
**RAI14**	-0.40	0.76	5.38E-06	2.62E-05
**N4BP2**	-0.65	0.64	5.38E-06	2.62E-05
**ZNF697**	0.86	1.81	5.40E-06	2.62E-05
**HSD11B2**	-0.79	0.58	5.40E-06	2.62E-05
**GTPBP4**	-0.29	0.82	5.43E-06	2.64E-05
**ARMC6**	-0.43	0.74	5.44E-06	2.64E-05
**FAM213A**	0.34	1.26	5.46E-06	2.65E-05
**SHANK2**	-0.81	0.57	5.50E-06	2.67E-05
**ASB7**	0.63	1.55	5.51E-06	2.67E-05
**HS1BP3**	0.46	1.37	5.51E-06	2.67E-05
**MED4**	0.49	1.40	5.52E-06	2.68E-05
**SYT12**	-1.02	0.49	5.52E-06	2.68E-05
**PCCA**	0.79	1.73	5.59E-06	2.71E-05
**TNPO3**	-0.30	0.81	5.60E-06	2.71E-05
**ICOSLG**	0.82	1.77	5.62E-06	2.72E-05
**FYCO1**	-0.35	0.78	5.63E-06	2.72E-05
**SLC11A2**	-0.39	0.77	5.65E-06	2.73E-05
**KRR1**	0.39	1.31	5.68E-06	2.75E-05
**FOXD2-AS1**	-1.60	0.33	5.70E-06	2.76E-05
**DSCR3**	-0.39	0.77	5.72E-06	2.77E-05
**SSH3**	-0.36	0.78	5.75E-06	2.78E-05
**AF131217.1**	0.99	1.99	5.77E-06	2.79E-05
**NKIRAS2**	0.37	1.30	5.82E-06	2.81E-05
**SMARCA5**	-0.31	0.81	5.87E-06	2.84E-05
**C16orf59**	-0.68	0.62	5.90E-06	2.85E-05
**C17orf96**	-0.61	0.65	5.91E-06	2.85E-05
**RALBP1**	-0.30	0.81	5.93E-06	2.86E-05
**KIF16B**	-0.44	0.74	5.95E-06	2.87E-05
**PLIN3**	0.34	1.26	5.95E-06	2.87E-05
**AMZ2**	0.33	1.26	5.96E-06	2.88E-05
**WDR4**	-0.49	0.71	5.99E-06	2.89E-05
**ZBTB11**	0.42	1.33	6.09E-06	2.94E-05
**SSBP4**	-0.43	0.74	6.20E-06	2.99E-05
**CACFD1**	0.83	1.78	6.25E-06	3.01E-05
**MYO1A**	1.92	3.79	6.37E-06	3.07E-05
**ALDH1A3**	0.31	1.24	6.38E-06	3.07E-05
**HUWE1**	-0.58	0.67	6.38E-06	3.07E-05
**ACSL1**	-0.37	0.77	6.41E-06	3.09E-05
**TMEM214**	-0.32	0.80	6.45E-06	3.10E-05
**RPL23**	-0.29	0.82	6.48E-06	3.12E-05
**PASK**	-0.59	0.66	6.56E-06	3.16E-05
**RPS6KA1**	0.29	1.23	6.57E-06	3.16E-05
**RPS14**	-0.22	0.86	6.59E-06	3.17E-05
**FXN**	-0.74	0.60	6.61E-06	3.18E-05
**SHROOM3**	-0.43	0.74	6.61E-06	3.18E-05
**SLC30A6**	0.40	1.32	6.65E-06	3.19E-05
**NECAP2**	0.35	1.28	6.71E-06	3.22E-05
**SPSB1**	0.54	1.45	6.71E-06	3.22E-05
**CLP1**	0.52	1.44	6.72E-06	3.22E-05
**POU3F1**	0.88	1.84	6.72E-06	3.23E-05
**ING1**	-0.64	0.64	6.73E-06	3.23E-05
**GAB1**	0.47	1.39	6.77E-06	3.24E-05

**LINC01116**	-1.43	0.37	6.77E-06	3.25E-05
**GJC1**	-0.48	0.72	6.81E-06	3.27E-05
**FBF1**	0.75	1.68	6.87E-06	3.29E-05
**APOBEC2**	1.89	3.71	6.87E-06	3.29E-05
**RAB9A**	-0.49	0.71	6.92E-06	3.31E-05
**MLLT11**	0.55	1.47	6.93E-06	3.32E-05
**SPAG4**	1.65	3.14	6.97E-06	3.33E-05
**RNASEH2CP1**	1.52	2.86	6.98E-06	3.34E-05
**ATP6V1G1**	0.30	1.23	7.00E-06	3.35E-05
**MAML2**	-0.54	0.69	7.02E-06	3.36E-05
**HPS5**	0.45	1.36	7.04E-06	3.36E-05
**MGAT5**	-0.34	0.79	7.04E-06	3.37E-05
**C9orf114**	-0.49	0.71	7.13E-06	3.41E-05
**CCT3**	-0.23	0.85	7.15E-06	3.42E-05
**PTPN12**	-0.35	0.79	7.21E-06	3.44E-05
**STRAP**	-0.26	0.84	7.21E-06	3.44E-05
**HIF3A**	-1.91	0.27	7.25E-06	3.46E-05
**MFAP3L**	0.85	1.80	7.25E-06	3.46E-05
**ATOX1**	0.44	1.35	7.28E-06	3.47E-05
**MCFD2**	0.27	1.21	7.30E-06	3.48E-05
**VPS11**	0.40	1.32	7.30E-06	3.48E-05
**CA9**	-1.62	0.32	7.33E-06	3.50E-05
**ACOT11**	-0.56	0.68	7.36E-06	3.51E-05
**XRCC2**	-0.67	0.63	7.44E-06	3.55E-05
**FLRT2**	0.36	1.28	7.46E-06	3.56E-05
**TSTA3**	-0.31	0.81	7.52E-06	3.58E-05
**SLC13A3**	1.07	2.10	7.53E-06	3.59E-05
**TCEA1**	-0.41	0.75	7.54E-06	3.59E-05
**CSMD3**	1.54	2.90	7.56E-06	3.60E-05
**RCL1**	0.45	1.36	7.73E-06	3.68E-05
**CES4A**	1.08	2.12	7.76E-06	3.69E-05
**ADRBK2**	0.48	1.39	7.76E-06	3.69E-05
**OLFM2**	1.43	2.69	7.77E-06	3.69E-05
**DISP1**	1.36	2.56	7.81E-06	3.71E-05
**SLC35B4**	0.49	1.40	7.83E-06	3.72E-05
**TMEM14A**	-0.82	0.57	7.86E-06	3.73E-05
**RGMA**	1.30	2.47	7.93E-06	3.77E-05
**SAMM50**	0.33	1.25	7.98E-06	3.79E-05
**ZFP36L2**	0.25	1.19	8.01E-06	3.80E-05
**INO80**	-0.36	0.78	8.11E-06	3.85E-05
**NFATC3**	0.45	1.36	8.14E-06	3.86E-05
**MANEAL**	-1.63	0.32	8.20E-06	3.89E-05
**MBNL1**	-0.32	0.80	8.27E-06	3.92E-05
**IARS**	-0.27	0.83	8.29E-06	3.93E-05
**OXTR**	1.22	2.33	8.29E-06	3.93E-05
**PYCARD**	0.49	1.41	8.31E-06	3.94E-05
**LINC00152**	0.78	1.71	8.35E-06	3.96E-05
**CA13**	-0.81	0.57	8.43E-06	3.99E-05
**STXBP2**	0.43	1.34	8.47E-06	4.01E-05
**NKAPL**	1.41	2.66	8.48E-06	4.01E-05
**WNK3**	1.58	2.99	8.53E-06	4.03E-05
**PTPN23**	-0.35	0.79	8.54E-06	4.04E-05
**UBE2I**	-0.36	0.78	8.55E-06	4.04E-05
**ZWINT**	-0.39	0.76	8.61E-06	4.07E-05
**FIGN**	-0.77	0.59	8.61E-06	4.07E-05
**CLN6**	-0.47	0.72	8.62E-06	4.07E-05
**GCH1**	0.55	1.46	8.70E-06	4.11E-05
**KPNA3**	-0.34	0.79	8.78E-06	4.15E-05
**NAB1**	-0.43	0.74	8.81E-06	4.16E-05
**TOX4**	0.29	1.22	8.86E-06	4.18E-05
**AK8**	1.41	2.66	8.87E-06	4.18E-05
**VAMP3**	0.29	1.22	8.93E-06	4.21E-05
**DUSP16**	0.35	1.27	9.20E-06	4.34E-05
**FA2H**	-1.12	0.46	9.22E-06	4.35E-05
**IPMK**	0.69	1.61	9.23E-06	4.35E-05
**CCDC6**	-0.33	0.79	9.38E-06	4.42E-05
**IFT46**	-0.47	0.72	9.41E-06	4.43E-05
**MT-RNR1**	0.34	1.27	9.43E-06	4.44E-05
**SERP1**	-0.31	0.81	9.45E-06	4.45E-05
**GABPB2**	-0.56	0.68	9.53E-06	4.49E-05
**MRGBP**	-0.45	0.73	9.60E-06	4.52E-05
**EIF6**	-0.33	0.80	9.65E-06	4.54E-05
**KRTAP2-3**	1.41	2.66	9.66E-06	4.54E-05
**SNX10**	-1.56	0.34	9.68E-06	4.55E-05
**UACA**	-0.39	0.77	9.70E-06	4.56E-05
**ZNF396**	1.58	3.00	9.73E-06	4.57E-05
**TRNP1**	0.37	1.29	9.81E-06	4.61E-05
**CEP85**	-0.45	0.73	9.87E-06	4.63E-05
**FIG4**	0.55	1.46	9.89E-06	4.64E-05
**PGS1**	0.62	1.54	9.90E-06	4.65E-05
**FOXA2**	1.87	3.67	9.95E-06	4.67E-05
**UBE2L6**	0.49	1.41	9.98E-06	4.68E-05
**MVB12B**	0.67	1.59	9.98E-06	4.68E-05
**BASP1**	0.33	1.26	1.00E-05	4.69E-05
**PAFAH1B3**	-0.51	0.70	1.00E-05	4.69E-05
**ZNF532**	-0.33	0.80	1.02E-05	4.76E-05
**EIF5AL1**	-0.84	0.56	1.02E-05	4.77E-05
**PPP4R1**	-0.28	0.83	1.02E-05	4.78E-05
**NCAPD3**	-0.34	0.79	1.02E-05	4.78E-05
**ZNF639**	-0.59	0.67	1.02E-05	4.79E-05

**COX15**	0.40	1.32	1.02E-05	4.79E-05
**CCDC106**	-1.00	0.50	1.04E-05	4.86E-05
**CHST10**	-1.39	0.38	1.06E-05	4.96E-05
**RP11-347C18.3**	1.80	3.47	1.07E-05	5.02E-05
**AATF**	-0.30	0.81	1.08E-05	5.06E-05
**RNF167**	0.35	1.27	1.08E-05	5.07E-05
**C11orf84**	-0.52	0.70	1.09E-05	5.09E-05
**TIMM10**	-0.44	0.74	1.10E-05	5.13E-05
**MGARP**	1.85	3.60	1.10E-05	5.15E-05
**PAM**	0.32	1.24	1.11E-05	5.16E-05
**STAG3**	1.65	3.13	1.12E-05	5.21E-05
**UBE2W**	0.62	1.54	1.12E-05	5.22E-05
**ITSN1**	-0.35	0.79	1.13E-05	5.27E-05
**VPS36**	-0.40	0.76	1.13E-05	5.28E-05
**MMRN2**	-0.59	0.67	1.14E-05	5.30E-05
**FER1L6**	1.85	3.60	1.14E-05	5.30E-05
**UPF1**	0.25	1.19	1.14E-05	5.30E-05
**MSI2**	0.42	1.34	1.14E-05	5.31E-05
**TCAIM**	0.51	1.42	1.14E-05	5.32E-05
**IQCC**	-0.92	0.53	1.15E-05	5.37E-05
**SLC25A24**	-0.34	0.79	1.15E-05	5.37E-05
**AKNA**	-0.67	0.63	1.16E-05	5.40E-05
**FAAP24**	-0.84	0.56	1.16E-05	5.42E-05
**STK4**	0.34	1.27	1.17E-05	5.42E-05
**FGD3**	1.07	2.10	1.17E-05	5.42E-05
**FAM35A**	-0.51	0.70	1.17E-05	5.43E-05
**ANTXR2**	0.34	1.27	1.17E-05	5.46E-05
**MTMR6**	-0.38	0.77	1.18E-05	5.46E-05
**PTPRM**	0.56	1.48	1.18E-05	5.47E-05
**UBA2**	-0.32	0.80	1.19E-05	5.54E-05
**GINS4**	0.50	1.42	1.19E-05	5.55E-05
**UNC119**	0.43	1.34	1.20E-05	5.56E-05
**POFUT1**	-0.31	0.81	1.20E-05	5.56E-05
**DEDD2**	0.43	1.35	1.20E-05	5.57E-05
**FURIN**	0.27	1.21	1.20E-05	5.58E-05
**TPMT**	0.38	1.30	1.21E-05	5.61E-05
**TWSG1**	0.37	1.30	1.21E-05	5.61E-05
**RP11-115D19.3**	1.86	3.62	1.21E-05	5.61E-05
**AGO4**	-0.70	0.61	1.21E-05	5.62E-05
**NOC4L**	-0.45	0.73	1.22E-05	5.66E-05
**UTP3**	0.38	1.30	1.24E-05	5.73E-05
**SLC25A13**	-0.34	0.79	1.24E-05	5.74E-05
**ITGB8**	-0.41	0.75	1.24E-05	5.74E-05
**PDZK1IP1**	-0.96	0.51	1.24E-05	5.75E-05
**SLC7A8**	0.33	1.26	1.25E-05	5.77E-05
**C16orf13**	-0.43	0.74	1.25E-05	5.77E-05
**PLAUR**	0.38	1.30	1.25E-05	5.78E-05
**SIPA1L3**	0.41	1.33	1.26E-05	5.81E-05
**STK11**	0.33	1.25	1.26E-05	5.82E-05
**NDUFB1**	0.51	1.42	1.28E-05	5.92E-05
**SLC39A9**	0.31	1.24	1.30E-05	5.98E-05
**SUPT4H1**	0.38	1.31	1.30E-05	6.01E-05
**RNF141**	-0.33	0.79	1.30E-05	6.01E-05
**MAP3K7**	0.33	1.26	1.31E-05	6.03E-05
**PANK1**	0.58	1.50	1.31E-05	6.03E-05
**ZNF732**	-1.32	0.40	1.31E-05	6.06E-05
**NOD1**	-0.86	0.55	1.32E-05	6.07E-05
**FLVCR1**	-0.63	0.64	1.32E-05	6.09E-05
**MYPN**	1.76	3.38	1.32E-05	6.10E-05
**ZNF606**	0.92	1.89	1.33E-05	6.11E-05
**AIMP2**	-0.43	0.74	1.33E-05	6.11E-05
**MAP3K12**	0.83	1.78	1.33E-05	6.12E-05
**SREK1**	-0.39	0.77	1.33E-05	6.12E-05
**HSPBP1**	-0.36	0.78	1.33E-05	6.14E-05
**ZDHHC11**	1.27	2.41	1.34E-05	6.17E-05
**KLK14**	-0.90	0.54	1.35E-05	6.19E-05
**DNAJC7**	-0.33	0.80	1.35E-05	6.19E-05
**MAVS**	0.28	1.22	1.35E-05	6.22E-05
**N4BP3**	-0.62	0.65	1.36E-05	6.23E-05
**PELP1**	-0.37	0.77	1.36E-05	6.26E-05
**PWWP2B**	-0.44	0.74	1.37E-05	6.28E-05
**SIVA1**	-0.40	0.76	1.38E-05	6.33E-05
**PYCRL**	-0.56	0.68	1.38E-05	6.33E-05
**SPIDR**	-0.40	0.76	1.38E-05	6.35E-05
**RAD52**	0.68	1.60	1.39E-05	6.39E-05
**ARL5B**	0.46	1.37	1.39E-05	6.39E-05
**POLR3B**	-0.39	0.76	1.39E-05	6.40E-05
**DYSF**	-1.27	0.42	1.40E-05	6.40E-05
**ZNF213**	-0.55	0.68	1.40E-05	6.42E-05
**TSEN54**	-0.42	0.75	1.40E-05	6.43E-05
**SPACA6P**	1.31	2.49	1.40E-05	6.43E-05
**SMG8**	0.41	1.33	1.43E-05	6.53E-05
**TNFRSF19**	-1.07	0.48	1.43E-05	6.55E-05
**COX7A2**	0.31	1.24	1.43E-05	6.57E-05
**HACD2**	-0.28	0.82	1.44E-05	6.60E-05
**PRMT5**	-0.30	0.81	1.45E-05	6.63E-05
**TTC30A**	0.86	1.82	1.46E-05	6.66E-05
**EXTL2**	-0.79	0.58	1.47E-05	6.72E-05
**IFT20**	0.69	1.62	1.47E-05	6.72E-05
**C14orf79**	0.61	1.53	1.50E-05	6.85E-05

MBD5	0.53	1.44	1.52E-05	6.95E-05
KLF6	-0.28	0.83	1.53E-05	6.99E-05
CCP110	0.43	1.35	1.54E-05	7.02E-05
NEIL1	0.93	1.90	1.54E-05	7.02E-05
MFSD1	0.37	1.30	1.54E-05	7.03E-05
COPRS	-0.47	0.72	1.55E-05	7.06E-05
FBRS	0.31	1.24	1.55E-05	7.06E-05
MFSD12	-0.42	0.75	1.55E-05	7.08E-05
ITGB6	0.37	1.29	1.55E-05	7.09E-05
XRCC3	-0.66	0.63	1.57E-05	7.17E-05
DNAJC3	-0.41	0.75	1.57E-05	7.18E-05
GDI2	-0.24	0.85	1.58E-05	7.18E-05
HSPA4	0.29	1.22	1.58E-05	7.18E-05
FHDC1	-0.48	0.72	1.58E-05	7.20E-05
PSME4	0.33	1.25	1.58E-05	7.22E-05
WRAP73	0.43	1.35	1.59E-05	7.26E-05
LETM1	-0.30	0.81	1.60E-05	7.28E-05
CD164L2	1.11	2.15	1.60E-05	7.29E-05
H2AFJ	0.43	1.35	1.61E-05	7.32E-05
CYP26B1	-1.02	0.49	1.61E-05	7.32E-05
FLG-AS1	1.61	3.05	1.61E-05	7.33E-05
STK38L	0.41	1.33	1.62E-05	7.37E-05
NOL11	-0.35	0.78	1.62E-05	7.38E-05
CLK3	0.35	1.27	1.63E-05	7.42E-05
GNAQ	-0.35	0.79	1.63E-05	7.43E-05
RP11-539I5.1	-0.83	0.56	1.64E-05	7.46E-05
PLCL2	1.83	3.55	1.64E-05	7.46E-05
OLA1	-0.32	0.80	1.65E-05	7.49E-05
UBAP2L	-0.28	0.82	1.66E-05	7.53E-05
CDH24	0.62	1.54	1.66E-05	7.53E-05
TBC1D8	0.36	1.28	1.67E-05	7.57E-05
CST6	0.99	1.99	1.67E-05	7.59E-05
MINA	-0.40	0.76	1.68E-05	7.62E-05
SYK	-0.34	0.79	1.70E-05	7.69E-05
NUDT3	-0.48	0.71	1.70E-05	7.70E-05
WDR36	-0.37	0.77	1.70E-05	7.70E-05
ICE1	-0.34	0.79	1.70E-05	7.71E-05
MED15	0.30	1.23	1.72E-05	7.79E-05
B4GALT1	0.25	1.19	1.73E-05	7.85E-05
SLC39A13	0.45	1.37	1.73E-05	7.85E-05
ZNF451	0.41	1.33	1.74E-05	7.88E-05
UHRF2	-0.39	0.77	1.75E-05	7.91E-05
CEP76	0.64	1.56	1.76E-05	7.94E-05
ZNF774	0.93	1.91	1.76E-05	7.94E-05
PNPLA8	0.51	1.43	1.77E-05	7.99E-05
NME3	-0.91	0.53	1.77E-05	8.00E-05
CCDC138	-0.90	0.54	1.78E-05	8.04E-05
OSGIN2	0.46	1.38	1.79E-05	8.06E-05
GRIPAP1	0.36	1.29	1.79E-05	8.07E-05
PRPF4B	-0.33	0.80	1.79E-05	8.07E-05
WASL	-0.29	0.82	1.80E-05	8.11E-05
DFFA	0.29	1.22	1.82E-05	8.19E-05
INO80D	-0.54	0.69	1.82E-05	8.20E-05
RNF114	0.33	1.25	1.82E-05	8.21E-05
DTX3	0.80	1.74	1.82E-05	8.22E-05
PRELID1	-0.37	0.77	1.83E-05	8.23E-05
WIPI2	0.31	1.24	1.83E-05	8.23E-05
ZNF750	0.30	1.23	1.83E-05	8.23E-05
PI4KAP1	0.80	1.74	1.83E-05	8.25E-05
PDCD10	-0.36	0.78	1.85E-05	8.32E-05
KMT2B	-0.36	0.78	1.85E-05	8.32E-05
CTD-3099C6.9	-0.89	0.54	1.85E-05	8.33E-05
TDRKH	0.59	1.50	1.85E-05	8.34E-05
TGIF2	-0.58	0.67	1.86E-05	8.35E-05
KLK12	-1.03	0.49	1.86E-05	8.36E-05
DYNLL1	-0.23	0.85	1.87E-05	8.39E-05
TAF1C	0.46	1.37	1.87E-05	8.40E-05
ADM2	-0.87	0.55	1.87E-05	8.41E-05
EMC1	0.32	1.24	1.89E-05	8.50E-05
CHERP	-0.39	0.76	1.89E-05	8.50E-05
TFB2M	-0.59	0.67	1.90E-05	8.53E-05
MIR205HG	0.29	1.22	1.91E-05	8.56E-05
SUN1	-0.28	0.82	1.91E-05	8.57E-05
NAF1	-0.56	0.68	1.91E-05	8.59E-05
ADAMTS13	1.49	2.80	1.92E-05	8.62E-05
TUBGCP4	-0.41	0.75	1.93E-05	8.66E-05
LYPD5	-0.73	0.60	1.94E-05	8.72E-05
PAXBP1-AS1	1.74	3.34	1.95E-05	8.74E-05
TMCO1	0.34	1.26	1.97E-05	8.83E-05
FAM127C	0.52	1.43	1.97E-05	8.84E-05
GBP1	0.62	1.54	1.98E-05	8.88E-05
DMC1	-1.47	0.36	2.02E-05	9.04E-05
PLEKHM1	0.39	1.31	2.03E-05	9.08E-05
EXPH5	-0.62	0.65	2.03E-05	9.09E-05
MTURN	0.53	1.44	2.05E-05	9.16E-05
NDUFS2	-0.27	0.83	2.05E-05	9.18E-05
GOLGA7	0.37	1.29	2.05E-05	9.18E-05
FRMD5	0.88	1.85	2.07E-05	9.26E-05
GNB1	-0.23	0.85	2.09E-05	9.35E-05
ST6GALNAC5	1.00	2.00	2.10E-05	9.37E-05

**TPRN**	-0.65	0.64	2.10E-05	9.37E-05
**MFSD11**	0.67	1.59	2.10E-05	9.38E-05
**CTD-2555C10.3**	0.99	1.98	2.11E-05	9.43E-05
**HACD3**	-0.29	0.82	2.12E-05	9.45E-05
**RBBP8**	-0.32	0.80	2.12E-05	9.46E-05
**CTU2**	-0.58	0.67	2.13E-05	9.51E-05
**PSME1**	0.32	1.25	2.13E-05	9.52E-05
**NSDHL**	0.33	1.26	2.14E-05	9.54E-05
**OPTN**	0.35	1.27	2.15E-05	9.57E-05
**PPIE**	0.35	1.27	2.16E-05	9.61E-05
**KDM6B**	0.35	1.27	2.17E-05	9.68E-05
**ZNF211**	0.74	1.67	2.18E-05	9.71E-05
**ACADVL**	0.24	1.18	2.18E-05	9.72E-05
**KIF21A**	-0.38	0.77	2.20E-05	9.79E-05
**AP1M2**	-0.32	0.80	2.21E-05	9.83E-05
**INTS10**	-0.34	0.79	2.21E-05	9.83E-05
**ANKRD22**	-0.54	0.69	2.21E-05	9.85E-05
**CASC10**	0.87	1.83	2.22E-05	9.87E-05
**PBDC1**	-0.38	0.77	2.22E-05	9.89E-05
**FDPS**	-0.34	0.79	2.24E-05	9.95E-05
**LIPG**	0.27	1.21	2.24E-05	9.97E-05
**AC098823.3**	1.66	3.16	2.25E-05	9.98E-05
**PBRM1**	-0.32	0.80	2.25E-05	9.99E-05
**EVA1B**	1.00	2.00	2.25E-05	1.00E-04
**ZFYVE9**	-0.39	0.76	2.25E-05	1.00E-04
**ZNF44**	0.76	1.69	2.26E-05	1.00E-04
**ARSI**	0.72	1.65	2.26E-05	1.00E-04
**SIN3A**	-0.32	0.80	2.27E-05	1.01E-04
**C11orf87**	1.73	3.32	2.27E-05	1.01E-04
**PP7080**	-0.90	0.53	2.29E-05	1.02E-04
**C15orf39**	-0.41	0.75	2.31E-05	1.03E-04
**CNNM1**	-1.47	0.36	2.32E-05	1.03E-04
**SUFU**	0.41	1.33	2.32E-05	1.03E-04
**CBFA2T2**	0.42	1.34	2.34E-05	1.04E-04
**KCTD3**	-0.33	0.79	2.34E-05	1.04E-04
**WHSC1L1**	-0.32	0.80	2.35E-05	1.04E-04
**SAMD9**	0.65	1.57	2.35E-05	1.04E-04
**ZBTB48**	0.67	1.59	2.37E-05	1.05E-04
**PRPF18**	0.75	1.68	2.37E-05	1.05E-04
**MAGI2-AS3**	-0.99	0.50	2.37E-05	1.05E-04
**PTGER4**	1.03	2.04	2.38E-05	1.05E-04
**ZFP69B**	-1.15	0.45	2.38E-05	1.05E-04
**SRF**	0.37	1.29	2.39E-05	1.05E-04
**H6PD**	-0.37	0.78	2.41E-05	1.06E-04
**CFAP70**	1.54	2.91	2.41E-05	1.06E-04
**BFAR**	-0.30	0.81	2.41E-05	1.06E-04
**ANKRD28**	-0.38	0.77	2.41E-05	1.06E-04
**ARCN1**	0.24	1.18	2.41E-05	1.06E-04
**TIAM2**	-1.09	0.47	2.41E-05	1.06E-04
**SLC44A4**	1.27	2.42	2.41E-05	1.07E-04
**UQCR10**	0.33	1.26	2.45E-05	1.08E-04
**ATP10D**	-0.33	0.79	2.46E-05	1.08E-04
**PDIA4**	-0.24	0.85	2.46E-05	1.08E-04
**UHRF1BP1L**	0.47	1.38	2.46E-05	1.08E-04
**COMMD7**	-0.39	0.76	2.48E-05	1.09E-04
**HTT**	0.32	1.25	2.48E-05	1.09E-04
**KRT10**	-0.34	0.79	2.49E-05	1.10E-04
**RAB29**	0.39	1.31	2.49E-05	1.10E-04
**APC2**	1.50	2.83	2.50E-05	1.10E-04
**ANXA6**	-0.99	0.50	2.51E-05	1.11E-04
**BCL9**	-0.46	0.73	2.52E-05	1.11E-04
**DDIAS**	-0.46	0.73	2.52E-05	1.11E-04
**DNAJC28**	1.47	2.76	2.52E-05	1.11E-04
**E2F4**	-0.37	0.77	2.53E-05	1.11E-04
**PAQR3**	-0.48	0.72	2.53E-05	1.11E-04
**RP3-368A4.5**	1.28	2.42	2.54E-05	1.12E-04
**FASTKD2**	-0.36	0.78	2.54E-05	1.12E-04
**SAFB**	-0.29	0.82	2.55E-05	1.12E-04
**DAP3**	-0.29	0.82	2.56E-05	1.12E-04
**PTEN**	0.38	1.30	2.59E-05	1.14E-04
**PROCR**	0.37	1.29	2.61E-05	1.15E-04
**RNF4**	-0.30	0.81	2.65E-05	1.17E-04
**TGFB2**	1.16	2.23	2.66E-05	1.17E-04
**ARL8A**	0.35	1.28	2.66E-05	1.17E-04
**PDZD2**	-0.39	0.76	2.69E-05	1.18E-04
**NDUFV3**	0.35	1.27	2.70E-05	1.18E-04
**GINS1**	-0.43	0.74	2.70E-05	1.18E-04
**DMTF1**	0.42	1.33	2.71E-05	1.19E-04
**AC074289.1**	1.77	3.41	2.71E-05	1.19E-04
**SLC25A16**	0.53	1.45	2.72E-05	1.19E-04
**HELLS**	-0.54	0.69	2.75E-05	1.20E-04
**C16orf52**	0.60	1.51	2.76E-05	1.21E-04
**PHF14**	-0.46	0.73	2.77E-05	1.21E-04
**HELZ2**	0.33	1.26	2.77E-05	1.21E-04
**TCTN2**	-0.69	0.62	2.78E-05	1.22E-04
**ITPKC**	0.33	1.25	2.79E-05	1.22E-04
**GLI3**	-0.45	0.73	2.79E-05	1.22E-04
**LCN2**	0.47	1.39	2.79E-05	1.22E-04
**LINC01468**	0.50	1.41	2.80E-05	1.22E-04
**COX7B**	0.27	1.20	2.80E-05	1.22E-04

**FBLN7**	1.68	3.20	2.83E-05	1.24E-04
**ENDOV**	0.75	1.68	2.83E-05	1.24E-04
**PMS1**	-0.55	0.68	2.84E-05	1.24E-04
**AMMECR1**	-0.36	0.78	2.84E-05	1.24E-04
**RP11-458D21.1**	1.50	2.83	2.84E-05	1.24E-04
**AQP9**	-0.56	0.68	2.85E-05	1.24E-04
**RP11-285E9.6**	1.59	3.01	2.85E-05	1.25E-04
**RABL6**	-0.27	0.83	2.86E-05	1.25E-04
**YWHAG**	0.21	1.16	2.87E-05	1.25E-04
**BTBD7**	-0.34	0.79	2.88E-05	1.25E-04
**RALGAPB**	0.29	1.22	2.88E-05	1.26E-04
**RDH13**	-0.46	0.73	2.89E-05	1.26E-04
**NUDC**	-0.27	0.83	2.90E-05	1.26E-04
**SLC22A5**	0.53	1.45	2.91E-05	1.27E-04
**NGEF**	-1.22	0.43	2.92E-05	1.27E-04
**MYL12A**	0.25	1.19	2.92E-05	1.27E-04
**PKD1P6**	-0.70	0.62	2.97E-05	1.29E-04
**AF131215.2**	1.29	2.45	2.97E-05	1.29E-04
**ZFP92**	0.82	1.77	2.98E-05	1.29E-04
**DLX2**	-1.11	0.46	2.98E-05	1.29E-04
**UFM1**	0.39	1.31	2.98E-05	1.30E-04
**SOS2**	0.42	1.34	2.98E-05	1.30E-04
**GALNT2**	0.26	1.20	2.99E-05	1.30E-04
**TMTC4**	-0.60	0.66	2.99E-05	1.30E-04
**SLC45A3**	0.67	1.59	3.00E-05	1.30E-04
**C17orf51**	-0.82	0.57	3.00E-05	1.30E-04
**ADAM8**	-0.36	0.78	3.01E-05	1.31E-04
**CLEC2B**	1.01	2.01	3.06E-05	1.33E-04
**ZNF736P9Y**	-1.07	0.48	3.06E-05	1.33E-04
**PPP1R18**	-0.30	0.81	3.07E-05	1.33E-04
**MDGA1**	-1.34	0.40	3.07E-05	1.33E-04
**ATG7**	0.37	1.30	3.09E-05	1.34E-04
**TPRA1**	-0.44	0.74	3.10E-05	1.34E-04
**MORN4**	0.57	1.48	3.12E-05	1.35E-04
**PTGR1**	0.33	1.26	3.12E-05	1.35E-04
**NDRG1**	0.59	1.50	3.13E-05	1.36E-04
**BRI3**	-0.49	0.71	3.16E-05	1.37E-04
**ADCK4**	-0.49	0.71	3.16E-05	1.37E-04
**PRKD3**	-0.39	0.76	3.17E-05	1.37E-04
**C16orf87**	0.59	1.51	3.20E-05	1.39E-04
**HOXA5**	0.91	1.88	3.21E-05	1.39E-04
**PPP1R1B**	-1.20	0.44	3.21E-05	1.39E-04
**RAPGEF2**	-0.40	0.76	3.23E-05	1.40E-04
**UNC13B**	0.32	1.25	3.23E-05	1.40E-04
**IRGQ**	0.44	1.36	3.30E-05	1.43E-04
**ZBED5**	0.43	1.35	3.30E-05	1.43E-04
**PEX19**	0.33	1.26	3.33E-05	1.44E-04
**TSPAN5**	-0.55	0.68	3.34E-05	1.44E-04
**KIAA2026**	0.41	1.33	3.35E-05	1.45E-04
**HS3ST6**	-1.61	0.33	3.38E-05	1.46E-04
**AC093323.3**	-0.65	0.64	3.39E-05	1.46E-04
**C1orf174**	-0.44	0.74	3.41E-05	1.47E-04
**TMCO6**	-0.66	0.63	3.41E-05	1.47E-04
**SLC10A6**	-0.69	0.62	3.44E-05	1.48E-04
**FAM83A**	-0.29	0.82	3.44E-05	1.48E-04
**CPE**	-0.60	0.66	3.45E-05	1.49E-04
**PLEKHH1**	0.53	1.45	3.46E-05	1.49E-04
**P4HB**	-0.23	0.85	3.46E-05	1.49E-04
**LATS1**	-0.38	0.77	3.47E-05	1.49E-04
**BORCS6**	0.55	1.46	3.48E-05	1.50E-04
**ALOX12**	-1.16	0.45	3.50E-05	1.50E-04
**PKM**	0.21	1.16	3.51E-05	1.51E-04
**DSG3**	-0.23	0.85	3.51E-05	1.51E-04
**EML6**	1.15	2.22	3.52E-05	1.52E-04
**SNX7**	-0.47	0.72	3.55E-05	1.53E-04
**NRSN2**	0.43	1.34	3.55E-05	1.53E-04
**RRP9**	-0.39	0.76	3.56E-05	1.53E-04
**CLNS1A**	-0.28	0.82	3.57E-05	1.54E-04
**PDE4DIP**	-0.38	0.77	3.57E-05	1.54E-04
**EHMT2**	-0.37	0.77	3.58E-05	1.54E-04
**DHRS1**	0.29	1.22	3.62E-05	1.56E-04
**TTC28**	-0.92	0.53	3.64E-05	1.56E-04
**LPAR6**	-0.67	0.63	3.65E-05	1.57E-04
**ERMP1**	-0.34	0.79	3.66E-05	1.57E-04
**TRIM21**	0.44	1.35	3.68E-05	1.58E-04
**SLC10A3**	0.36	1.29	3.68E-05	1.58E-04
**ZFAND6**	0.38	1.30	3.68E-05	1.58E-04
**VSNL1**	-0.38	0.77	3.69E-05	1.58E-04
**TREML1**	1.75	3.36	3.74E-05	1.60E-04
**PI3**	-0.40	0.76	3.74E-05	1.60E-04
**STX2**	-0.59	0.66	3.75E-05	1.60E-04
**POP7**	-0.39	0.76	3.77E-05	1.62E-04
**PSG6**	1.52	2.87	3.78E-05	1.62E-04
**SERPINE2**	-0.26	0.83	3.78E-05	1.62E-04
**FGF5**	-1.41	0.38	3.81E-05	1.63E-04
**TRIM23**	0.69	1.61	3.81E-05	1.63E-04
**MIF4GD**	-0.53	0.69	3.81E-05	1.63E-04
**ANKRD13B**	0.55	1.46	3.81E-05	1.63E-04
**CCT4**	-0.23	0.85	3.83E-05	1.64E-04
**MRPS5**	-0.29	0.82	3.83E-05	1.64E-04

**ROCK1**	-0.32	0.80	3.83E-05	1.64E-04
**RP11-111F16.2**	-1.17	0.44	3.84E-05	1.64E-04
**MAP3K4**	-0.37	0.77	3.86E-05	1.65E-04
**ELAVL2**	-0.51	0.70	3.88E-05	1.66E-04
**VPS25**	0.32	1.25	3.88E-05	1.66E-04
**TADA1**	-0.65	0.64	3.88E-05	1.66E-04
**HAUS2**	-0.42	0.75	3.90E-05	1.66E-04
**ZMYM2**	0.32	1.25	3.90E-05	1.66E-04
**HVCN1**	1.46	2.75	3.91E-05	1.67E-04
**FH**	-0.30	0.81	3.92E-05	1.67E-04
**ARHGAP22**	0.78	1.72	3.93E-05	1.68E-04
**08-sep**	0.26	1.20	3.96E-05	1.69E-04
**NEURL1B**	-0.46	0.73	3.97E-05	1.69E-04
**RP11-336A10.4**	1.16	2.23	3.97E-05	1.69E-04
**ZMYM1**	-0.51	0.70	4.02E-05	1.71E-04
**MUTYH**	-0.68	0.62	4.02E-05	1.71E-04
**CRELD2**	-0.44	0.74	4.03E-05	1.71E-04
**AEBP2**	0.38	1.30	4.09E-05	1.74E-04
**EPHB6**	-1.05	0.48	4.10E-05	1.74E-04
**CSTA**	-0.30	0.81	4.13E-05	1.76E-04
**PLEC**	-0.26	0.84	4.14E-05	1.76E-04
**DZIP1**	0.95	1.93	4.18E-05	1.78E-04
**TXNL4A**	-0.32	0.80	4.19E-05	1.78E-04
**NRG1**	-0.39	0.76	4.22E-05	1.79E-04
**TPRXL**	0.58	1.50	4.24E-05	1.80E-04
**NHSL1**	-0.39	0.76	4.26E-05	1.81E-04
**TDP1**	-0.39	0.76	4.27E-05	1.81E-04
**POFUT2**	0.51	1.42	4.28E-05	1.82E-04
**SNAPC3**	0.40	1.32	4.31E-05	1.83E-04
**CCDC12**	-0.44	0.74	4.35E-05	1.85E-04
**ZC3H18**	-0.34	0.79	4.36E-05	1.85E-04
**ZNF57**	-0.70	0.62	4.37E-05	1.85E-04
**HOPX**	-0.36	0.78	4.39E-05	1.86E-04
**FAM161A**	-0.91	0.53	4.39E-05	1.86E-04
**CDKN1B**	-0.48	0.72	4.41E-05	1.87E-04
**LRFN4**	-0.55	0.68	4.41E-05	1.87E-04
**SNAPC2**	-0.54	0.69	4.41E-05	1.87E-04
**MICAL2**	0.28	1.22	4.43E-05	1.88E-04
**QKI**	-0.30	0.81	4.44E-05	1.88E-04
**KIAA1549L**	0.51	1.42	4.46E-05	1.89E-04
**RAP1GAP2**	0.31	1.24	4.48E-05	1.89E-04
**RMI1**	-0.52	0.70	4.50E-05	1.91E-04
**ACAT2**	-0.41	0.75	4.52E-05	1.91E-04
**ANXA2**	-0.19	0.87	4.53E-05	1.91E-04
**PGAP2**	-0.41	0.75	4.54E-05	1.92E-04
**DUSP9**	-1.61	0.33	4.57E-05	1.93E-04
**TMPRSS11F**	-0.97	0.51	4.58E-05	1.94E-04
**TMEM18**	-0.49	0.71	4.58E-05	1.94E-04
**VLDLR**	-0.56	0.68	4.59E-05	1.94E-04
**YJEFN3**	1.46	2.75	4.61E-05	1.95E-04
**MICU2**	0.37	1.29	4.61E-05	1.95E-04
**STRBP**	-0.39	0.76	4.65E-05	1.96E-04
**NLRX1**	0.33	1.25	4.67E-05	1.97E-04
**LYSMD2**	0.79	1.73	4.67E-05	1.97E-04
**NCKAP5**	0.49	1.41	4.71E-05	1.99E-04
**PPP5C**	-0.32	0.80	4.71E-05	1.99E-04
**DNA2**	-0.55	0.68	4.72E-05	1.99E-04
**KIAA1143**	-0.52	0.70	4.73E-05	1.99E-04
**ARSJ**	-0.42	0.75	4.73E-05	1.99E-04
**NTM**	-0.58	0.67	4.73E-05	1.99E-04
**SIPA1L1**	-0.32	0.80	4.76E-05	2.00E-04
**ERBB3**	-0.29	0.82	4.78E-05	2.01E-04
**SAP30**	-0.68	0.62	4.80E-05	2.02E-04
**AKT1**	-0.25	0.84	4.81E-05	2.03E-04
**TSSC1**	0.38	1.30	4.83E-05	2.03E-04
**MRPS10**	-0.30	0.81	4.84E-05	2.04E-04
**GNAS**	0.23	1.17	4.85E-05	2.04E-04
**RNF185**	0.41	1.33	4.85E-05	2.04E-04
**SMOC2**	-1.58	0.33	4.90E-05	2.06E-04
**ZBTB12**	-1.39	0.38	4.93E-05	2.07E-04
**VKORC1**	-0.62	0.65	4.93E-05	2.07E-04
**PCDH12**	1.45	2.74	4.94E-05	2.07E-04
**LENG1**	0.67	1.60	4.97E-05	2.09E-04
**ZNF252P**	0.46	1.38	4.98E-05	2.09E-04
**CCDC58**	-0.45	0.73	5.03E-05	2.11E-04
**GSK3B**	-0.27	0.83	5.03E-05	2.11E-04
**DPCD**	0.41	1.33	5.03E-05	2.11E-04
**ST3GAL1**	-0.43	0.74	5.08E-05	2.13E-04
**DPP8**	0.34	1.26	5.10E-05	2.14E-04
**ZNF702P**	1.33	2.51	5.12E-05	2.15E-04
**COG6**	0.41	1.33	5.12E-05	2.15E-04
**RP5-984P4.6**	1.65	3.14	5.13E-05	2.15E-04
**CAPN5**	0.95	1.93	5.14E-05	2.15E-04
**CENPT**	0.45	1.37	5.17E-05	2.16E-04
**ST8SIA1**	1.68	3.21	5.18E-05	2.17E-04
**ZAN**	1.71	3.28	5.21E-05	2.18E-04
**SVIP**	-0.51	0.70	5.22E-05	2.18E-04
**IRAK2**	0.87	1.83	5.23E-05	2.19E-04
**JTB**	-0.36	0.78	5.23E-05	2.19E-04
**NCS1**	-0.28	0.82	5.24E-05	2.19E-04

PTDSS1	-0.26	0.83	5.25E-05	2.19E-04
CMTM7	0.36	1.28	5.26E-05	2.20E-04
APH1A	-0.26	0.84	5.27E-05	2.20E-04
TALDO1	-0.24	0.85	5.29E-05	2.21E-04
RFC3	-0.40	0.76	5.30E-05	2.22E-04
GNA12	-0.32	0.80	5.31E-05	2.22E-04
TPP1	0.30	1.23	5.31E-05	2.22E-04
NPHP4	-0.52	0.70	5.36E-05	2.24E-04
COBL	0.67	1.59	5.36E-05	2.24E-04
ARL6IP6	-0.50	0.71	5.37E-05	2.24E-04
LGMN	0.33	1.25	5.37E-05	2.24E-04
GLYR1	0.28	1.22	5.38E-05	2.24E-04
PPP1R12C	0.37	1.29	5.41E-05	2.26E-04
ASIC3	1.58	3.00	5.51E-05	2.30E-04
C5orf34	-0.75	0.59	5.51E-05	2.30E-04
LDOC1	0.51	1.42	5.51E-05	2.30E-04
DIAPH2	-0.50	0.71	5.53E-05	2.31E-04
MEX3D	-0.46	0.73	5.58E-05	2.33E-04
ZFC3H1	0.43	1.35	5.61E-05	2.34E-04
SBDSP1	-0.50	0.71	5.62E-05	2.34E-04
NOTCH2	-0.26	0.84	5.64E-05	2.35E-04
DNM3	1.40	2.63	5.67E-05	2.36E-04
IGFBP7	0.31	1.24	5.72E-05	2.38E-04
ELAVL1	-0.29	0.82	5.74E-05	2.39E-04
EMC7	0.35	1.27	5.75E-05	2.39E-04
MYO5C	-0.75	0.60	5.77E-05	2.40E-04
METTL1	-0.61	0.66	5.77E-05	2.40E-04
UGDH	-0.45	0.73	5.83E-05	2.42E-04
PNPLA2	0.40	1.32	5.84E-05	2.43E-04
GBA	0.37	1.29	5.85E-05	2.43E-04
UBL5	0.29	1.22	5.86E-05	2.44E-04
SCFD2	-0.44	0.74	5.87E-05	2.44E-04
CYB561D1	-0.49	0.71	5.87E-05	2.44E-04
TTC26	-0.65	0.64	5.88E-05	2.44E-04
LINC00589	1.19	2.28	5.90E-05	2.45E-04
RPA2	-0.39	0.76	5.90E-05	2.45E-04
LNX2	0.39	1.31	5.92E-05	2.45E-04
EIF3D	-0.22	0.86	5.92E-05	2.45E-04
PAOX	0.86	1.81	5.94E-05	2.46E-04
VARS2	-0.45	0.73	5.95E-05	2.46E-04
GDPD3	-0.78	0.58	5.98E-05	2.48E-04
INSR	-0.77	0.59	6.00E-05	2.48E-04
NIPAL4	-0.28	0.82	6.01E-05	2.49E-04
PPP1R13B	-0.38	0.77	6.09E-05	2.52E-04
SPRR2A	-0.40	0.76	6.10E-05	2.52E-04
RNASET2	0.95	1.93	6.11E-05	2.53E-04
PPP1R15A	0.42	1.34	6.25E-05	2.58E-04
LPIN3	0.43	1.35	6.26E-05	2.59E-04
ZNF707	0.58	1.49	6.27E-05	2.59E-04
AFG3L1P	-0.60	0.66	6.27E-05	2.59E-04
ADSL	-0.33	0.79	6.28E-05	2.60E-04
DHFR	-0.43	0.74	6.28E-05	2.60E-04
PBXIP1	0.51	1.42	6.35E-05	2.62E-04
GFER	-0.66	0.63	6.37E-05	2.63E-04
BZW2	-0.31	0.81	6.37E-05	2.63E-04
CORO1A	0.57	1.48	6.38E-05	2.63E-04
AARS2	0.41	1.33	6.38E-05	2.64E-04
LDLRAD3	-0.42	0.75	6.41E-05	2.64E-04
CEP135	-0.66	0.63	6.43E-05	2.65E-04
RP1-228H13.5	-1.24	0.42	6.50E-05	2.68E-04
SPA17	-0.60	0.66	6.51E-05	2.68E-04
USP3	-0.52	0.70	6.53E-05	2.69E-04
CD151	-0.28	0.82	6.54E-05	2.70E-04
PIGG	0.32	1.25	6.55E-05	2.70E-04
CSPP1	-0.59	0.66	6.56E-05	2.70E-04
MPDU1	0.32	1.25	6.58E-05	2.71E-04
TPRG1	-0.86	0.55	6.60E-05	2.72E-04
MIER3	0.51	1.42	6.61E-05	2.72E-04
TSKU	-0.30	0.81	6.64E-05	2.73E-04
GCSH	-1.16	0.45	6.79E-05	2.79E-04
CXCL1	-0.64	0.64	6.81E-05	2.80E-04
CEP104	0.33	1.26	6.89E-05	2.83E-04
C1QTNF1-AS1	1.68	3.21	6.91E-05	2.84E-04
MALT1	0.33	1.26	6.96E-05	2.86E-04
PAK2	-0.24	0.84	6.97E-05	2.86E-04
ANKMY1	-0.61	0.65	6.98E-05	2.87E-04
SUSD4	-1.45	0.37	6.98E-05	2.87E-04
ORMDL2	0.40	1.32	6.99E-05	2.87E-04
ASTE1	-0.80	0.58	7.01E-05	2.88E-04
CRYZ	0.36	1.28	7.03E-05	2.89E-04
EPB41L1	0.48	1.39	7.05E-05	2.89E-04
CDK9	0.32	1.24	7.07E-05	2.90E-04
TIPIN	-0.58	0.67	7.14E-05	2.93E-04
FAM102A	0.42	1.34	7.15E-05	2.93E-04
FLYWCH1	0.32	1.25	7.15E-05	2.93E-04
RBBP8NL	-0.70	0.62	7.17E-05	2.94E-04
LGALSL	-0.34	0.79	7.22E-05	2.96E-04
ANKRD36BP2	-1.24	0.42	7.26E-05	2.97E-04
MED13	0.30	1.23	7.28E-05	2.98E-04
KIF3B	0.28	1.21	7.29E-05	2.99E-04

**C14orf28**	1.27	2.42	7.30E-05	2.99E-04
**DPP7**	-0.36	0.78	7.30E-05	2.99E-04
**PLPP1**	0.86	1.82	7.33E-05	3.00E-04
**RBM41**	0.48	1.40	7.41E-05	3.03E-04
**CCDC13**	1.68	3.20	7.41E-05	3.03E-04
**CAPN8**	1.38	2.60	7.44E-05	3.04E-04
**JHDM1D-AS1**	0.89	1.85	7.44E-05	3.04E-04
**SLC36A1**	0.46	1.37	7.48E-05	3.06E-04
**SEZ6L2**	0.54	1.45	7.50E-05	3.07E-04
**PARP3**	0.68	1.60	7.51E-05	3.07E-04
**TOP2B**	-0.31	0.80	7.55E-05	3.08E-04
**UTY**	0.53	1.44	7.55E-05	3.09E-04
**IDH3B**	0.29	1.22	7.59E-05	3.10E-04
**NOMO2**	0.51	1.42	7.65E-05	3.12E-04
**NEDD4L**	0.31	1.24	7.68E-05	3.13E-04
**DDX20**	-0.41	0.75	7.70E-05	3.14E-04
**RP5-1056L3.3**	-0.77	0.59	7.71E-05	3.15E-04
**CYB561**	-0.30	0.81	7.73E-05	3.15E-04
**BPNT1**	0.39	1.31	7.73E-05	3.15E-04
**TUSC3**	0.32	1.25	7.74E-05	3.16E-04
**XXYLT1**	-0.45	0.73	7.77E-05	3.17E-04
**BRK1**	0.27	1.20	7.84E-05	3.19E-04
**TMEM30B**	-0.36	0.78	7.91E-05	3.22E-04
**POLE3**	-0.29	0.82	7.94E-05	3.23E-04
**RP11-84G21.1**	-1.54	0.34	7.94E-05	3.23E-04
**WHAMM**	0.48	1.40	7.94E-05	3.23E-04
**ZNF766**	-0.63	0.65	7.96E-05	3.24E-04
**PRPF38B**	-0.31	0.81	8.04E-05	3.27E-04
**RBM12**	-0.25	0.84	8.14E-05	3.31E-04
**PRSS27**	0.40	1.32	8.17E-05	3.32E-04
**CRIM1**	-0.25	0.84	8.18E-05	3.33E-04
**HPX**	1.65	3.14	8.20E-05	3.33E-04
**CDC42BPA**	-0.32	0.80	8.20E-05	3.33E-04
**DNAJC21**	0.32	1.24	8.21E-05	3.34E-04
**RTKN**	-0.33	0.80	8.21E-05	3.34E-04
**HIC2**	0.54	1.45	8.22E-05	3.34E-04
**TMEM241**	-0.63	0.65	8.23E-05	3.34E-04
**MINPP1**	0.48	1.40	8.29E-05	3.36E-04
**P2RY1**	-0.83	0.56	8.34E-05	3.39E-04
**LINC00704**	0.88	1.84	8.36E-05	3.39E-04
**RPL36AL**	-0.28	0.82	8.37E-05	3.40E-04
**S100A10**	-0.22	0.86	8.38E-05	3.40E-04
**EPB41**	-0.36	0.78	8.40E-05	3.41E-04
**QDPR**	-0.39	0.76	8.40E-05	3.41E-04
**LAMB2**	0.33	1.26	8.41E-05	3.41E-04
**PPP2CA**	-0.27	0.83	8.44E-05	3.42E-04
**OIP5**	-0.76	0.59	8.51E-05	3.45E-04
**PEPD**	0.36	1.28	8.53E-05	3.45E-04
**CC2D1B**	0.34	1.27	8.55E-05	3.46E-04
**TXNDC11**	-0.36	0.78	8.57E-05	3.47E-04
**NFKB1**	-0.28	0.82	8.58E-05	3.47E-04
**SLC15A4**	-0.43	0.74	8.60E-05	3.48E-04
**MT-ND1**	0.79	1.73	8.71E-05	3.52E-04
**GID4**	0.39	1.31	8.75E-05	3.54E-04
**SLC38A4**	1.19	2.28	8.83E-05	3.57E-04
**EOMES**	1.25	2.38	8.83E-05	3.57E-04
**GJA5**	1.12	2.17	8.85E-05	3.58E-04
**SLK**	-0.27	0.83	8.89E-05	3.59E-04
**ZNHIT6**	-0.37	0.77	8.92E-05	3.61E-04
**CYP51A1**	-0.71	0.61	8.96E-05	3.62E-04
**METTL10**	-0.84	0.56	8.96E-05	3.62E-04
**NIFK**	-0.38	0.77	8.99E-05	3.63E-04
**MT-CO1**	0.65	1.57	9.01E-05	3.64E-04
**ATF2**	-0.42	0.75	9.09E-05	3.67E-04
**MMAB**	-0.42	0.75	9.12E-05	3.68E-04
**PSMA7**	-0.24	0.84	9.14E-05	3.69E-04
**CC2D2A**	-0.44	0.74	9.18E-05	3.70E-04
**WWP1**	0.33	1.26	9.19E-05	3.71E-04
**STEAP1B**	-1.03	0.49	9.21E-05	3.71E-04
**CRIPT**	0.67	1.59	9.24E-05	3.72E-04
**TRIM11**	0.37	1.30	9.30E-05	3.75E-04
**GLB1L3**	-0.98	0.51	9.31E-05	3.75E-04
**ZWILCH**	-0.37	0.77	9.35E-05	3.76E-04
**IFNAR1**	0.33	1.26	9.35E-05	3.77E-04
**NGLY1**	-0.38	0.77	9.38E-05	3.77E-04
**ADGRD2**	1.65	3.15	9.46E-05	3.81E-04
**NOL6**	-0.30	0.81	9.47E-05	3.81E-04
**SNX9**	-0.33	0.80	9.51E-05	3.83E-04
**EPS15L1**	-0.32	0.80	9.52E-05	3.83E-04
**NOMO3**	0.56	1.47	9.53E-05	3.83E-04
**G6PD**	0.30	1.23	9.57E-05	3.85E-04
**NDUFV1**	0.25	1.19	9.66E-05	3.88E-04
**SLC35D2**	-0.52	0.70	9.71E-05	3.90E-04
**PCTP**	0.44	1.36	9.71E-05	3.90E-04
**MXD3**	-0.95	0.52	9.72E-05	3.90E-04
**TOE1**	-0.61	0.66	9.72E-05	3.90E-04
**RUNX1**	0.33	1.26	9.72E-05	3.90E-04
**ISL1**	-0.70	0.61	9.77E-05	3.92E-04
**CRTC3**	-0.42	0.75	9.79E-05	3.93E-04
**COMMD10**	-0.57	0.67	9.83E-05	3.95E-04

**KDELC2**	0.45	1.37	9.84E-05	3.95E-04
**JPH1**	-0.59	0.66	9.84E-05	3.95E-04
**FAM171A2**	1.18	2.26	9.85E-05	3.95E-04
**ZNF423**	1.64	3.12	9.98E-05	4.00E-04
**GFPT1**	-0.33	0.80	1.00E-04	4.01E-04
**SEC16A**	-0.26	0.84	1.00E-04	4.02E-04
**ZNF682**	1.17	2.25	1.01E-04	4.05E-04
**PLEKHA2**	-0.31	0.81	1.01E-04	4.06E-04
**MLKL**	-0.42	0.75	1.02E-04	4.07E-04
**STK38**	0.30	1.23	1.02E-04	4.08E-04
**NCOA5**	-0.34	0.79	1.02E-04	4.08E-04
**LRP3**	-0.66	0.63	1.02E-04	4.10E-04
**HEXDC**	0.72	1.65	1.02E-04	4.10E-04
**TRRAP**	-0.37	0.77	1.03E-04	4.11E-04
**ELP5**	-0.37	0.77	1.03E-04	4.12E-04
**MAOA**	-0.36	0.78	1.03E-04	4.13E-04
**CALML6**	1.64	3.12	1.04E-04	4.14E-04
**STRN3**	0.40	1.32	1.04E-04	4.14E-04
**HSD3BP5**	1.65	3.13	1.04E-04	4.14E-04
**KLK1**	-1.02	0.49	1.04E-04	4.14E-04
**EDN2**	0.62	1.54	1.04E-04	4.15E-04
**GPX4**	-0.31	0.81	1.04E-04	4.15E-04
**LAMP2**	-0.27	0.83	1.04E-04	4.17E-04
**HIST1H1C**	0.82	1.77	1.05E-04	4.18E-04
**DCAF6**	0.29	1.23	1.05E-04	4.18E-04
**PRAF2**	0.75	1.69	1.06E-04	4.21E-04
**PCMTD2**	0.46	1.38	1.06E-04	4.23E-04
**NEDD9**	1.13	2.19	1.06E-04	4.24E-04
**RCCD1**	-0.53	0.69	1.06E-04	4.24E-04
**PTGS1**	-0.55	0.68	1.07E-04	4.25E-04
**SENP1**	-0.39	0.77	1.07E-04	4.26E-04
**NFYA**	-0.36	0.78	1.07E-04	4.26E-04
**TUBA4A**	-0.25	0.84	1.08E-04	4.29E-04
**NKTR**	-0.35	0.79	1.08E-04	4.30E-04
**TRIM68**	0.49	1.41	1.08E-04	4.30E-04
**ATG12**	0.39	1.31	1.08E-04	4.31E-04
**CHRAC1**	-0.37	0.78	1.08E-04	4.31E-04
**C17orf58**	-0.71	0.61	1.09E-04	4.32E-04
**RPL35A**	-0.22	0.86	1.09E-04	4.32E-04
**TIMMDC1**	0.29	1.22	1.09E-04	4.32E-04
**MEOX1**	-1.38	0.38	1.09E-04	4.33E-04
**DNAH9**	1.45	2.74	1.09E-04	4.34E-04
**RNF10**	0.24	1.18	1.09E-04	4.34E-04
**PLEKHA4**	0.69	1.61	1.09E-04	4.34E-04
**CNOT7**	-0.28	0.82	1.10E-04	4.35E-04
**SNRPE**	-0.47	0.72	1.10E-04	4.36E-04
**TULP4**	-0.42	0.75	1.10E-04	4.38E-04
**MAP9**	-0.55	0.68	1.10E-04	4.38E-04
**NECAB3**	0.72	1.65	1.11E-04	4.39E-04
**USP15**	0.33	1.26	1.11E-04	4.39E-04
**MAP2K7**	-0.34	0.79	1.11E-04	4.39E-04
**RAB18**	0.31	1.24	1.11E-04	4.42E-04
**ZNF117**	0.81	1.76	1.12E-04	4.44E-04
**CACNA2D2**	1.59	3.02	1.12E-04	4.45E-04
**PITPNB**	-0.34	0.79	1.13E-04	4.46E-04
**USP31**	-0.39	0.76	1.13E-04	4.47E-04
**AL627309.1**	1.12	2.17	1.13E-04	4.47E-04
**TBL2**	-0.37	0.78	1.13E-04	4.48E-04
**NFKBIZ**	-0.46	0.73	1.13E-04	4.49E-04
**NOTCH3**	-0.24	0.85	1.14E-04	4.51E-04
**PUS7L**	-0.43	0.74	1.14E-04	4.51E-04
**SDR16C5**	-0.63	0.65	1.14E-04	4.52E-04
**DNAJC16**	0.42	1.33	1.14E-04	4.52E-04
**ZNF706**	-0.30	0.81	1.14E-04	4.52E-04
**RALGPS2**	-0.40	0.76	1.15E-04	4.56E-04
**UBASH3B**	0.31	1.24	1.16E-04	4.58E-04
**ODF2L**	0.55	1.47	1.17E-04	4.62E-04
**XPO4**	-0.33	0.80	1.18E-04	4.65E-04
**SENP2**	0.33	1.26	1.18E-04	4.65E-04
**TMEM259**	0.27	1.21	1.19E-04	4.69E-04
**HMHA1**	-0.68	0.62	1.20E-04	4.74E-04
**PPP2R3A**	0.34	1.26	1.21E-04	4.77E-04
**FLJ42969**	1.59	3.02	1.21E-04	4.78E-04
**C4orf32**	-0.58	0.67	1.21E-04	4.79E-04
**SNX19**	0.26	1.20	1.22E-04	4.80E-04
**FBXO33**	0.47	1.39	1.22E-04	4.80E-04
**PLCE1**	-1.04	0.49	1.22E-04	4.81E-04
**ORAI3**	0.91	1.88	1.22E-04	4.81E-04
**VMP1**	0.30	1.23	1.22E-04	4.82E-04
**BAP1**	-0.27	0.83	1.23E-04	4.84E-04
**DECR1**	0.34	1.26	1.23E-04	4.84E-04
**LINC01021**	1.62	3.07	1.23E-04	4.84E-04
**PPP1R10**	0.26	1.20	1.23E-04	4.85E-04
**ALKBH1**	0.48	1.39	1.23E-04	4.86E-04
**SNRNP25**	-0.39	0.76	1.24E-04	4.88E-04
**GEN1**	-0.41	0.75	1.24E-04	4.90E-04
**CHID1**	0.31	1.24	1.25E-04	4.93E-04
**IL18**	0.33	1.25	1.25E-04	4.93E-04
**CDCP1**	0.24	1.18	1.26E-04	4.94E-04
**KIAA0556**	0.36	1.28	1.26E-04	4.96E-04

**TMEM50B**	-0.50	0.71	1.27E-04	4.97E-04
**MED6**	0.43	1.35	1.27E-04	5.00E-04
**DTWD2**	-0.94	0.52	1.28E-04	5.01E-04
**RPS10**	0.60	1.51	1.28E-04	5.02E-04
**KHDRBS3**	-1.04	0.49	1.28E-04	5.02E-04
**ARC**	1.61	3.06	1.28E-04	5.02E-04
**GJC2**	-1.41	0.38	1.29E-04	5.06E-04
**AC093673.5**	1.17	2.24	1.29E-04	5.07E-04
**ZCCHC3**	-0.42	0.75	1.29E-04	5.07E-04
**ARID3A**	-0.49	0.71	1.30E-04	5.09E-04
**GPR63**	-0.93	0.52	1.31E-04	5.14E-04
**UBE2F**	0.53	1.44	1.32E-04	5.17E-04
**TSPAN4**	-0.35	0.78	1.33E-04	5.21E-04
**RABEP1**	-0.31	0.81	1.33E-04	5.22E-04
**TIAM1**	-0.33	0.80	1.33E-04	5.22E-04
**KAT7**	-0.36	0.78	1.34E-04	5.25E-04
**UBE2O**	-0.32	0.80	1.35E-04	5.27E-04
**KLHL22**	0.47	1.39	1.35E-04	5.29E-04
**MIA3**	-0.34	0.79	1.35E-04	5.29E-04
**NOL10**	-0.35	0.78	1.35E-04	5.29E-04
**KRT77**	-0.72	0.61	1.35E-04	5.29E-04
**RHBDD3**	0.45	1.37	1.35E-04	5.30E-04
**SSFA2**	-0.26	0.84	1.35E-04	5.30E-04
**PLEKHG2**	-0.39	0.76	1.37E-04	5.34E-04
**RNF216P1**	-0.60	0.66	1.37E-04	5.35E-04
**KIF13A**	-0.25	0.84	1.37E-04	5.35E-04
**ZNF426**	0.48	1.39	1.37E-04	5.35E-04
**SLC35F3**	-0.71	0.61	1.37E-04	5.36E-04
**DMTN**	0.57	1.48	1.38E-04	5.38E-04
**ASCC3**	0.28	1.22	1.38E-04	5.40E-04
**EXOSC1**	0.37	1.30	1.39E-04	5.44E-04
**SDF2**	0.39	1.31	1.39E-04	5.44E-04
**SLC45A1**	1.61	3.05	1.40E-04	5.45E-04
**06-mar**	0.27	1.21	1.41E-04	5.50E-04
**SRSF10**	-0.49	0.71	1.42E-04	5.55E-04
**PRR13**	0.33	1.25	1.43E-04	5.56E-04
**ZSCAN25**	0.40	1.32	1.43E-04	5.56E-04
**DNAJC17**	-0.51	0.70	1.43E-04	5.57E-04
**HS6ST1**	0.35	1.27	1.43E-04	5.57E-04
**SERTAD4**	-0.60	0.66	1.43E-04	5.57E-04
**EDC3**	0.33	1.26	1.43E-04	5.57E-04
**DHRS4**	0.47	1.39	1.43E-04	5.57E-04
**MEAF6**	-0.35	0.79	1.44E-04	5.59E-04
**MAD1L1**	0.34	1.27	1.44E-04	5.59E-04
**SNX32**	1.47	2.76	1.45E-04	5.63E-04
**YLPM1**	-0.27	0.83	1.46E-04	5.67E-04
**RUNX2**	0.61	1.52	1.46E-04	5.67E-04
**SLC30A9**	0.34	1.27	1.46E-04	5.67E-04
**RNF216**	-0.33	0.80	1.46E-04	5.70E-04
**ORC3**	-0.35	0.79	1.47E-04	5.71E-04
**UBAC1**	-0.30	0.81	1.49E-04	5.78E-04
**MED16**	-0.32	0.80	1.49E-04	5.78E-04
**AC011738.4**	1.25	2.37	1.49E-04	5.79E-04
**GNG4**	-1.23	0.43	1.49E-04	5.79E-04
**WDR74**	-0.35	0.79	1.49E-04	5.80E-04
**CTC-308K20.1**	1.58	3.00	1.49E-04	5.80E-04
**DAAM1**	-0.30	0.81	1.49E-04	5.80E-04
**PPFIA3**	0.42	1.33	1.50E-04	5.81E-04
**AC068057.1**	1.61	3.05	1.50E-04	5.82E-04
**USP34**	-0.32	0.80	1.50E-04	5.83E-04
**SPATA13**	-1.11	0.46	1.51E-04	5.87E-04
**AP1M1**	0.29	1.22	1.52E-04	5.91E-04
**ATP5D**	-0.27	0.83	1.53E-04	5.92E-04
**SETD3**	0.27	1.21	1.53E-04	5.92E-04
**CDKL2**	-1.51	0.35	1.53E-04	5.93E-04
**ZBTB39**	-0.52	0.70	1.53E-04	5.93E-04
**DCTD**	-0.28	0.83	1.53E-04	5.93E-04
**RP11-104N10.2**	1.03	2.05	1.54E-04	5.98E-04
**L3MBTL2**	-0.41	0.75	1.55E-04	5.99E-04
**CBLC**	0.30	1.23	1.55E-04	5.99E-04
**ATP7B**	0.55	1.46	1.55E-04	6.00E-04
**GPD2**	-0.26	0.83	1.55E-04	6.00E-04
**TNFAIP3**	0.35	1.27	1.55E-04	6.01E-04
**OTUD4**	-0.30	0.81	1.56E-04	6.02E-04
**ROMO1**	0.33	1.26	1.56E-04	6.02E-04
**CYP24A1**	1.59	3.01	1.57E-04	6.07E-04
**RYR1**	0.88	1.84	1.58E-04	6.09E-04
**PGM3**	-0.41	0.75	1.58E-04	6.10E-04
**PDCD2**	-0.29	0.82	1.59E-04	6.13E-04
**AMT**	1.04	2.06	1.59E-04	6.13E-04
**RPUSD3**	-0.33	0.79	1.60E-04	6.17E-04
**DHRS2**	-1.35	0.39	1.60E-04	6.19E-04
**ERI2**	0.45	1.37	1.60E-04	6.20E-04
**COLGALT1**	-0.27	0.83	1.61E-04	6.20E-04
**SKI**	-0.29	0.82	1.61E-04	6.20E-04
**AK2**	-0.23	0.85	1.61E-04	6.20E-04
**ATP8B3**	-1.35	0.39	1.61E-04	6.21E-04
**CEP85L**	1.04	2.06	1.61E-04	6.22E-04
**HUNK**	-1.44	0.37	1.63E-04	6.27E-04
**ARHGEF2**	-0.29	0.82	1.63E-04	6.28E-04

**HSPA1B**	-0.39	0.76	1.64E-04	6.30E-04
**LIFR**	1.43	2.70	1.64E-04	6.31E-04
**FAM214B**	0.30	1.23	1.64E-04	6.31E-04
**VPRBP**	-0.32	0.80	1.64E-04	6.33E-04
**DOT1L**	-0.32	0.80	1.65E-04	6.34E-04
**BCOR**	-0.38	0.77	1.66E-04	6.38E-04
**RGL2**	0.31	1.24	1.66E-04	6.39E-04
**PHAX**	0.31	1.24	1.67E-04	6.41E-04
**DUSP12**	-0.46	0.73	1.67E-04	6.44E-04
**FBN2**	0.28	1.22	1.68E-04	6.45E-04
**ARMCX2**	0.45	1.37	1.68E-04	6.48E-04
**FAM174A**	0.42	1.34	1.69E-04	6.49E-04
**UTP6**	-0.30	0.81	1.69E-04	6.49E-04
**FAM83B**	-0.32	0.80	1.70E-04	6.52E-04
**XKRX**	-1.38	0.38	1.70E-04	6.54E-04
**GBF1**	0.25	1.19	1.70E-04	6.54E-04
**ADAL**	0.52	1.43	1.71E-04	6.55E-04
**SLC22A20**	1.27	2.42	1.72E-04	6.59E-04
**TRIM37**	-0.39	0.76	1.72E-04	6.60E-04
**TAF5L**	-0.33	0.80	1.72E-04	6.60E-04
**STK36**	0.46	1.38	1.72E-04	6.61E-04
**MRRF**	-0.31	0.81	1.72E-04	6.61E-04
**ZBTB45**	-0.44	0.74	1.73E-04	6.65E-04
**KCTD13**	0.52	1.43	1.73E-04	6.65E-04
**PSMC3IP**	-0.72	0.61	1.73E-04	6.65E-04
**ZFYVE19**	0.36	1.28	1.74E-04	6.66E-04
**RNF223**	0.87	1.83	1.74E-04	6.68E-04
**EIF3J**	-0.28	0.82	1.75E-04	6.72E-04
**ADAMTSL4**	-0.30	0.81	1.76E-04	6.72E-04
**LINC00265**	0.96	1.95	1.77E-04	6.76E-04
**EFCAB2**	1.12	2.17	1.78E-04	6.80E-04
**USP8**	0.30	1.23	1.78E-04	6.82E-04
**EDF1**	-0.22	0.86	1.81E-04	6.94E-04
**RP11-404O13.1**	1.48	2.78	1.81E-04	6.94E-04
**DNAJC14**	-0.45	0.73	1.82E-04	6.97E-04
**DLX4**	1.54	2.91	1.83E-04	6.98E-04
**WDR45B**	-0.25	0.84	1.83E-04	6.99E-04
**CYB561A3**	0.47	1.39	1.83E-04	7.01E-04
**DUSP3**	0.26	1.19	1.85E-04	7.09E-04
**VPS41**	0.32	1.24	1.87E-04	7.13E-04
**SLC35F2**	-0.29	0.82	1.87E-04	7.13E-04
**CDA**	0.32	1.25	1.87E-04	7.15E-04
**GJB3**	-0.24	0.85	1.87E-04	7.16E-04
**ANOS1**	1.51	2.84	1.88E-04	7.18E-04
**WAC**	-0.23	0.85	1.88E-04	7.19E-04
**MBD4**	-0.41	0.75	1.89E-04	7.20E-04
**RRNAD1**	0.48	1.40	1.90E-04	7.24E-04
**FZD1**	-0.41	0.75	1.90E-04	7.25E-04
**PCBP1-AS1**	0.89	1.85	1.90E-04	7.25E-04
**RPL12P47**	-0.87	0.55	1.90E-04	7.25E-04
**ARAP2**	-0.38	0.77	1.91E-04	7.27E-04
**RET**	1.59	3.00	1.91E-04	7.28E-04
**NKRF**	-0.51	0.70	1.92E-04	7.30E-04
**TBX6**	0.83	1.78	1.92E-04	7.30E-04
**CTSA**	0.29	1.22	1.92E-04	7.31E-04
**COL17A1**	0.18	1.13	1.93E-04	7.34E-04
**LUC7L3**	0.32	1.25	1.93E-04	7.35E-04
**DOCK9**	-0.27	0.83	1.93E-04	7.36E-04
**C2CD4C**	-1.59	0.33	1.94E-04	7.36E-04
**GAB2**	-0.62	0.65	1.94E-04	7.38E-04
**LMO7**	0.23	1.17	1.94E-04	7.38E-04
**SART3**	-0.28	0.82	1.96E-04	7.45E-04
**ARHGAP29**	0.31	1.24	1.96E-04	7.45E-04
**ZNF408**	0.44	1.36	1.97E-04	7.48E-04
**YIPF5**	0.30	1.23	1.97E-04	7.48E-04
**GBP2**	0.84	1.79	1.97E-04	7.50E-04
**STIM2**	-0.40	0.76	1.99E-04	7.56E-04
**RELT**	0.52	1.44	1.99E-04	7.57E-04
**SMPDL3B**	0.95	1.93	2.00E-04	7.58E-04
**TXNIP**	0.21	1.16	2.00E-04	7.59E-04
**CEP57**	-0.37	0.78	2.01E-04	7.62E-04
**S1PR4**	1.58	2.99	2.01E-04	7.62E-04
**PPAN**	-0.88	0.54	2.02E-04	7.65E-04
**MBP**	-0.29	0.82	2.03E-04	7.71E-04
**INTS7**	-0.37	0.78	2.03E-04	7.71E-04
**NDUFB5**	0.32	1.25	2.04E-04	7.72E-04
**LMAN2**	0.23	1.17	2.04E-04	7.74E-04
**HEXA**	0.44	1.36	2.05E-04	7.76E-04
**SLC2A5**	0.64	1.56	2.06E-04	7.79E-04
**IER2**	-0.34	0.79	2.06E-04	7.81E-04
**ENSA**	0.23	1.17	2.06E-04	7.82E-04
**RP1-202O8.2**	1.58	2.99	2.06E-04	7.82E-04
**SRM**	-0.32	0.80	2.07E-04	7.86E-04
**TAF15**	-0.25	0.84	2.08E-04	7.87E-04
**MAP3K13**	0.40	1.32	2.08E-04	7.87E-04
**IARS2**	-0.23	0.85	2.08E-04	7.89E-04
**SMIM7**	-0.45	0.73	2.09E-04	7.89E-04
**ANP32A**	-0.23	0.85	2.11E-04	7.97E-04
**HTR7**	0.52	1.44	2.11E-04	7.97E-04
**FGFR4**	-0.86	0.55	2.11E-04	7.98E-04

**SRSF11**	-0.24	0.85	2.11E-04	7.98E-04
**PGAP1**	0.50	1.41	2.12E-04	7.99E-04
**ADAMTS6**	-0.82	0.57	2.12E-04	8.00E-04
**ZXDC**	0.38	1.31	2.12E-04	8.02E-04
**MKNK2**	-0.27	0.83	2.13E-04	8.04E-04
**KDM5C**	0.25	1.19	2.13E-04	8.05E-04
**SYVN1**	0.32	1.25	2.13E-04	8.06E-04
**PQLC1**	-0.31	0.81	2.14E-04	8.08E-04
**SMARCD2**	-0.23	0.85	2.14E-04	8.08E-04
**ENOSF1**	-0.45	0.73	2.14E-04	8.09E-04
**TUBE1**	-0.60	0.66	2.15E-04	8.10E-04
**BRD3**	-0.39	0.76	2.16E-04	8.13E-04
**TAMM41**	-0.48	0.72	2.16E-04	8.13E-04
**CTDSP2**	-0.27	0.83	2.16E-04	8.13E-04
**VPS37A**	0.30	1.24	2.16E-04	8.13E-04
**PHF2**	-0.34	0.79	2.17E-04	8.18E-04
**SNX29**	-0.32	0.80	2.17E-04	8.19E-04
**SLC25A25-AS1**	0.96	1.94	2.20E-04	8.27E-04
**04-sep**	1.56	2.95	2.20E-04	8.29E-04
**LUC7L**	0.39	1.31	2.21E-04	8.32E-04
**CYB5R4**	0.41	1.33	2.22E-04	8.34E-04
**WNT4**	1.28	2.43	2.22E-04	8.35E-04
**LANCL1**	-0.28	0.82	2.22E-04	8.35E-04
**FAM114A1**	-0.36	0.78	2.22E-04	8.36E-04
**EPHA1**	-0.29	0.82	2.22E-04	8.36E-04
**SHISA5**	-0.27	0.83	2.23E-04	8.38E-04
**WDR35**	-0.50	0.71	2.23E-04	8.38E-04
**SORL1**	-0.24	0.85	2.23E-04	8.39E-04
**MAP10**	1.17	2.25	2.24E-04	8.42E-04
**RASSF9**	-0.73	0.60	2.24E-04	8.42E-04
**UBXN7**	0.34	1.27	2.24E-04	8.43E-04
**ACAP2**	-0.32	0.80	2.25E-04	8.43E-04
**NOA1**	-0.34	0.79	2.26E-04	8.47E-04
**VANGL2**	0.36	1.29	2.26E-04	8.47E-04
**NSUN2**	-0.23	0.85	2.28E-04	8.54E-04
**ECM2**	-1.56	0.34	2.28E-04	8.56E-04
**HACD4**	-0.64	0.64	2.29E-04	8.60E-04
**TAF12**	0.34	1.27	2.30E-04	8.63E-04
**TTC38**	-0.50	0.71	2.30E-04	8.63E-04
**WDTC1**	0.31	1.24	2.32E-04	8.71E-04
**RP11-511P7.5**	-1.33	0.40	2.33E-04	8.72E-04
**SLC9A8**	0.47	1.38	2.34E-04	8.77E-04
**LPGAT1**	-0.33	0.79	2.35E-04	8.79E-04
**C6orf132**	-0.23	0.85	2.35E-04	8.79E-04
**FUT10**	-0.57	0.68	2.36E-04	8.84E-04
**GATC**	0.42	1.33	2.37E-04	8.86E-04
**PGBD1**	-0.72	0.61	2.37E-04	8.87E-04
**QRICH1**	-0.24	0.84	2.37E-04	8.87E-04
**TNFAIP8L3**	-0.88	0.54	2.38E-04	8.90E-04
**EIF5**	-0.24	0.84	2.39E-04	8.93E-04
**RP3-326I13.1**	1.54	2.92	2.39E-04	8.93E-04
**ZNF165**	-1.00	0.50	2.40E-04	8.96E-04
**OVOL2**	-0.62	0.65	2.41E-04	9.02E-04
**PSMD10**	0.34	1.26	2.43E-04	9.09E-04
**SEC23A**	-0.28	0.83	2.45E-04	9.15E-04
**VPS51**	-0.31	0.81	2.45E-04	9.16E-04
**SRGAP1**	-0.42	0.75	2.47E-04	9.22E-04
**ALPK1**	-0.50	0.71	2.47E-04	9.23E-04
**CCDC22**	0.42	1.34	2.48E-04	9.25E-04
**NELFCD**	-0.26	0.84	2.48E-04	9.26E-04
**STAT5B**	-0.37	0.77	2.48E-04	9.27E-04
**TPST1**	0.60	1.51	2.53E-04	9.44E-04
**MRPL36**	-0.38	0.77	2.53E-04	9.44E-04
**METAP1D**	-0.68	0.63	2.57E-04	9.58E-04
**KIAA1522**	-0.24	0.85	2.57E-04	9.58E-04
**ARL4D**	-0.32	0.80	2.57E-04	9.59E-04
**C1orf116**	0.19	1.14	2.58E-04	9.60E-04
**MICU1**	0.30	1.23	2.58E-04	9.62E-04
**HINFP**	0.51	1.43	2.59E-04	9.63E-04
**VPS8**	0.34	1.27	2.59E-04	9.63E-04
**MYO1B**	-0.22	0.86	2.59E-04	9.66E-04
**QRFPR**	-1.51	0.35	2.60E-04	9.68E-04
**USP42**	-0.43	0.74	2.60E-04	9.68E-04
**CMIP**	0.26	1.19	2.60E-04	9.69E-04
**ZFAND5**	0.27	1.21	2.61E-04	9.69E-04
**CEP97**	0.48	1.39	2.61E-04	9.69E-04
**RTN4IP1**	-0.57	0.67	2.61E-04	9.72E-04
**CD47**	-0.35	0.78	2.62E-04	9.74E-04
**BHLHB9**	0.74	1.67	2.62E-04	9.75E-04
**ZNF692**	-0.46	0.73	2.63E-04	9.76E-04
**PPFIBP1**	0.26	1.19	2.63E-04	9.78E-04
**DESI2**	0.26	1.20	2.64E-04	9.79E-04
**LINC00475**	1.55	2.93	2.65E-04	9.85E-04
**RP11-650L12.2**	-1.36	0.39	2.66E-04	9.87E-04
**TRIM47**	-0.54	0.69	2.66E-04	9.89E-04
**PPP3CC**	-0.46	0.73	2.67E-04	9.91E-04
**MT-CO3**	0.45	1.36	2.68E-04	9.94E-04
**LATS2**	0.38	1.30	2.69E-04	1.00E-03
**CKMT1B**	-0.39	0.76	2.70E-04	1.00E-03
**NUP155**	-0.28	0.82	2.70E-04	1.00E-03

**LONP1**	-0.25	0.84	2.71E-04	1.00E-03
**TUSC1**	-0.50	0.71	2.71E-04	1.00E-03
**L1CAM**	-1.06	0.48	2.72E-04	1.01E-03
**QPRT**	0.65	1.57	2.74E-04	1.02E-03
**PSMD8**	0.21	1.16	2.75E-04	1.02E-03
**FUNDC1**	0.50	1.42	2.76E-04	1.02E-03
**C8orf33**	-0.31	0.81	2.78E-04	1.03E-03
**BCL7C**	-0.35	0.78	2.80E-04	1.04E-03
**POLL**	0.41	1.33	2.82E-04	1.04E-03
**SH3RF1**	-0.36	0.78	2.82E-04	1.04E-03
**PABPC4L**	-1.14	0.45	2.83E-04	1.05E-03
**GPRASP1**	1.36	2.57	2.83E-04	1.05E-03
**CALCOCO2**	-0.27	0.83	2.85E-04	1.05E-03
**CAPN2**	0.18	1.14	2.85E-04	1.05E-03
**HDAC11**	0.41	1.33	2.85E-04	1.06E-03
**COQ3**	-0.49	0.71	2.88E-04	1.07E-03
**NTMT1**	-0.43	0.74	2.90E-04	1.07E-03
**RARRES3**	1.17	2.26	2.90E-04	1.07E-03
**PPP1R16A**	-0.46	0.73	2.90E-04	1.07E-03
**ACADSB**	0.48	1.40	2.91E-04	1.07E-03
**PFKM**	-0.26	0.83	2.94E-04	1.08E-03
**RIOK3**	0.32	1.25	2.95E-04	1.09E-03
**RSRP1**	0.56	1.47	2.96E-04	1.09E-03
**PDHX**	0.35	1.28	2.97E-04	1.09E-03
**RASSF4**	1.53	2.89	2.97E-04	1.09E-03
**SAR1A**	0.25	1.19	2.97E-04	1.10E-03
**RNF11**	0.28	1.22	2.97E-04	1.10E-03
**VPS13C**	0.36	1.28	2.98E-04	1.10E-03
**KCNMA1**	1.27	2.41	2.99E-04	1.10E-03
**TMEM138**	-0.40	0.76	3.00E-04	1.11E-03
**ACTBL2**	1.46	2.75	3.00E-04	1.11E-03
**PELI3**	0.53	1.45	3.00E-04	1.11E-03
**LINGO1**	1.52	2.86	3.01E-04	1.11E-03
**GANAB**	0.21	1.16	3.02E-04	1.11E-03
**LINC01515**	-0.89	0.54	3.03E-04	1.12E-03
**CXorf23**	0.65	1.56	3.04E-04	1.12E-03
**C1orf131**	-0.52	0.70	3.04E-04	1.12E-03
**DHX34**	-0.38	0.77	3.05E-04	1.12E-03
**CHCHD6**	-0.46	0.73	3.05E-04	1.12E-03
**DUOXA2**	1.41	2.67	3.05E-04	1.12E-03
**TEX15**	1.51	2.86	3.06E-04	1.12E-03
**YY1**	-0.23	0.85	3.06E-04	1.12E-03
**CLIC6**	1.53	2.90	3.06E-04	1.12E-03
**HCP5**	0.79	1.73	3.06E-04	1.13E-03
**GLI4**	0.63	1.54	3.07E-04	1.13E-03
**SPINT1**	0.25	1.19	3.07E-04	1.13E-03
**GLTSCR1L**	-0.47	0.72	3.08E-04	1.13E-03
**APH1B**	0.74	1.67	3.08E-04	1.13E-03
**SLTM**	-0.24	0.85	3.09E-04	1.14E-03
**SERPINB7**	0.26	1.20	3.09E-04	1.14E-03
**ETHE1**	-0.26	0.84	3.10E-04	1.14E-03
**IFNK**	1.30	2.46	3.11E-04	1.14E-03
**C3orf67**	0.48	1.39	3.11E-04	1.14E-03
**HCCS**	0.32	1.25	3.11E-04	1.14E-03
**CENPM**	-0.58	0.67	3.12E-04	1.14E-03
**HIST1H4H**	1.22	2.34	3.12E-04	1.14E-03
**SEMA5A**	-0.95	0.52	3.12E-04	1.14E-03
**YIPF1**	0.37	1.29	3.13E-04	1.15E-03
**FZD7**	-0.92	0.53	3.13E-04	1.15E-03
**CHCHD4**	-0.39	0.76	3.13E-04	1.15E-03
**SRP14-AS1**	1.10	2.14	3.14E-04	1.15E-03
**SRD5A1**	-0.38	0.77	3.16E-04	1.16E-03
**PDIA5**	-0.39	0.76	3.16E-04	1.16E-03
**LCK**	-1.51	0.35	3.16E-04	1.16E-03
**FOXC1**	-0.34	0.79	3.18E-04	1.16E-03
**LRP4**	-0.50	0.71	3.18E-04	1.17E-03
**CTD-2033D15.2**	-1.47	0.36	3.19E-04	1.17E-03
**PIGA**	-0.43	0.74	3.20E-04	1.17E-03
**TMEM128**	0.47	1.38	3.21E-04	1.18E-03
**LNP1**	-1.41	0.38	3.22E-04	1.18E-03
**RBKS**	0.85	1.81	3.22E-04	1.18E-03
**LINC00884**	1.50	2.82	3.22E-04	1.18E-03
**PVR**	-0.28	0.83	3.22E-04	1.18E-03
**RBM28**	-0.31	0.81	3.23E-04	1.18E-03
**RBPMS**	-0.39	0.76	3.23E-04	1.18E-03
**CDC37**	0.23	1.17	3.24E-04	1.18E-03
**STARD7**	-0.22	0.86	3.24E-04	1.18E-03
**ZNF701**	-0.88	0.54	3.26E-04	1.19E-03
**FZD10**	1.37	2.58	3.26E-04	1.19E-03
**NPAS2**	-0.36	0.78	3.28E-04	1.20E-03
**MT-ND4L**	0.51	1.43	3.28E-04	1.20E-03
**BORCS8**	0.53	1.44	3.30E-04	1.20E-03
**EPM2AIP1**	0.53	1.45	3.30E-04	1.20E-03
**RABGGTA**	0.25	1.19	3.30E-04	1.20E-03
**FOXL2**	1.53	2.88	3.33E-04	1.21E-03
**FXR1**	-0.25	0.84	3.34E-04	1.22E-03
**ALOX5**	1.49	2.81	3.34E-04	1.22E-03
**NUP214**	-0.26	0.84	3.35E-04	1.22E-03
**PBLD**	0.99	1.99	3.36E-04	1.22E-03
**PHF20L1**	0.31	1.24	3.36E-04	1.22E-03

**LINC00294**	0.68	1.60	3.37E-04	1.22E-03
**TNFRSF1B**	1.52	2.86	3.37E-04	1.23E-03
**ATP2A2**	0.17	1.13	3.38E-04	1.23E-03
**ZNF724P**	-0.87	0.55	3.38E-04	1.23E-03
**TMEM237**	-0.35	0.78	3.39E-04	1.23E-03
**GSN**	0.22	1.17	3.42E-04	1.24E-03
**RBM3**	-0.22	0.86	3.42E-04	1.24E-03
**IGFL1P1**	1.52	2.87	3.43E-04	1.25E-03
**ZNF184**	-0.65	0.64	3.44E-04	1.25E-03
**UBE2V2**	0.31	1.24	3.45E-04	1.25E-03
**INO80E**	-0.35	0.79	3.46E-04	1.26E-03
**PEA15**	-0.21	0.86	3.47E-04	1.26E-03
**C16orf74**	0.32	1.25	3.48E-04	1.26E-03
**ATL3**	-0.29	0.82	3.48E-04	1.26E-03
**PCDHGA11**	-0.84	0.56	3.48E-04	1.26E-03
**POLR3C**	-0.38	0.77	3.49E-04	1.27E-03
**PIK3C2B**	-0.42	0.75	3.51E-04	1.27E-03
**TBC1D9**	0.43	1.35	3.51E-04	1.27E-03
**PROSER3**	-0.45	0.73	3.54E-04	1.28E-03
**ATP5E**	0.25	1.19	3.54E-04	1.28E-03
**DAPP1**	0.29	1.22	3.55E-04	1.29E-03
**RNF219**	-0.63	0.65	3.56E-04	1.29E-03
**TCEB1**	0.27	1.21	3.56E-04	1.29E-03
**GAS8**	0.49	1.40	3.57E-04	1.29E-03
**ATP6V0E1**	0.23	1.17	3.58E-04	1.30E-03
**CMTM4**	-0.29	0.82	3.59E-04	1.30E-03
**ING4**	0.59	1.51	3.59E-04	1.30E-03
**PSMC2**	0.23	1.17	3.60E-04	1.30E-03
**SEMA3A**	0.37	1.30	3.60E-04	1.30E-03
**CLDN9**	1.49	2.80	3.60E-04	1.30E-03
**APC**	-0.30	0.81	3.61E-04	1.31E-03
**GAD1**	1.34	2.53	3.62E-04	1.31E-03
**LCA5**	0.80	1.74	3.63E-04	1.31E-03
**CH17-360D5.3**	0.47	1.39	3.63E-04	1.31E-03
**RPS6KL1**	0.75	1.69	3.65E-04	1.32E-03
**RP11-257O5.2**	1.06	2.08	3.65E-04	1.32E-03
**KIF1C**	-0.20	0.87	3.66E-04	1.32E-03
**PAXIP1**	-0.39	0.76	3.69E-04	1.33E-03
**ZFP82**	-0.85	0.55	3.70E-04	1.34E-03
**RP11-447D11.3**	-0.96	0.52	3.71E-04	1.34E-03
**VPS45**	0.31	1.24	3.71E-04	1.34E-03
**RFT1**	-0.34	0.79	3.72E-04	1.34E-03
**UQCRC2**	0.19	1.14	3.73E-04	1.34E-03
**CLDN4**	-0.25	0.84	3.73E-04	1.35E-03
**ALCAM**	0.25	1.19	3.74E-04	1.35E-03
**POLR2K**	0.34	1.26	3.75E-04	1.35E-03
**LOXL1**	0.55	1.46	3.77E-04	1.36E-03
**NT5DC3**	-0.58	0.67	3.78E-04	1.36E-03
**RBL2**	0.30	1.23	3.78E-04	1.36E-03
**CEACAM19**	0.48	1.39	3.78E-04	1.36E-03
**LA16c-313D11.12**	1.17	2.25	3.79E-04	1.36E-03
**PSTPIP1**	-1.11	0.46	3.80E-04	1.37E-03
**MAPRE1**	0.22	1.17	3.82E-04	1.38E-03
**MSRB1**	0.41	1.33	3.84E-04	1.38E-03
**MINK1**	0.22	1.17	3.86E-04	1.39E-03
**GNB2L1**	-0.20	0.87	3.86E-04	1.39E-03
**TRAIP**	-0.54	0.69	3.87E-04	1.39E-03
**RP11-680F8.1**	1.51	2.84	3.87E-04	1.39E-03
**CEBPZOS**	0.35	1.27	3.88E-04	1.40E-03
**CYC1**	-0.22	0.86	3.89E-04	1.40E-03
**C19orf53**	-0.30	0.81	3.90E-04	1.40E-03
**CARD18**	1.15	2.22	3.91E-04	1.40E-03
**RPRD1A**	0.29	1.22	3.91E-04	1.40E-03
**SMAP1**	-0.48	0.72	3.91E-04	1.40E-03
**EPB41L4A-AS2**	-1.26	0.42	3.91E-04	1.41E-03
**GGA2**	0.27	1.21	3.92E-04	1.41E-03
**IDH2**	0.24	1.18	3.94E-04	1.42E-03
**ARPC4**	-0.27	0.83	3.96E-04	1.42E-03
**ITIH5**	1.50	2.84	3.97E-04	1.43E-03
**ACKR2**	1.30	2.46	4.00E-04	1.43E-03
**NRAV**	-0.44	0.74	4.02E-04	1.44E-03
**CHST15**	-0.31	0.81	4.02E-04	1.44E-03
**DPH3**	0.35	1.27	4.04E-04	1.45E-03
**AP1G1**	0.23	1.17	4.05E-04	1.45E-03
**ENO1**	-0.18	0.88	4.07E-04	1.46E-03
**PLEKHG3**	0.23	1.17	4.07E-04	1.46E-03
**GGCX**	0.29	1.22	4.08E-04	1.46E-03
**GINS3**	-0.48	0.72	4.10E-04	1.47E-03
**RP11-47A8.5**	0.88	1.84	4.11E-04	1.47E-03
**WDR77**	0.31	1.24	4.11E-04	1.47E-03
**NSMF**	-0.27	0.83	4.12E-04	1.48E-03
**RAB25**	-0.23	0.85	4.12E-04	1.48E-03
**UQCRB**	0.25	1.19	4.16E-04	1.49E-03
**ARFGAP3**	0.35	1.28	4.16E-04	1.49E-03
**PHRF1**	-0.26	0.83	4.18E-04	1.50E-03
**MATR3**	-0.21	0.86	4.18E-04	1.50E-03
**HEBP2**	-0.23	0.85	4.18E-04	1.50E-03
**IDUA**	1.12	2.18	4.18E-04	1.50E-03
**TOMM5**	-0.80	0.57	4.18E-04	1.50E-03
**MAPK4**	1.17	2.25	4.19E-04	1.50E-03

**SYT7**	-1.00	0.50	4.19E-04	1.50E-03
**ZNF382**	-1.15	0.45	4.21E-04	1.50E-03
**FCHO2**	0.34	1.26	4.22E-04	1.51E-03
**11-sep**	0.21	1.16	4.22E-04	1.51E-03
**MFHAS1**	-0.36	0.78	4.24E-04	1.51E-03
**SLC26A2**	0.42	1.34	4.25E-04	1.52E-03
**NR2F6**	-0.35	0.79	4.25E-04	1.52E-03
**ZNF585A**	0.70	1.63	4.26E-04	1.52E-03
**GOLIM4**	-0.29	0.82	4.26E-04	1.52E-03
**CHORDC1**	-0.34	0.79	4.27E-04	1.52E-03
**RIMS3**	0.76	1.70	4.27E-04	1.52E-03
**C1orf233**	-0.58	0.67	4.28E-04	1.52E-03
**SH2B1**	0.31	1.24	4.28E-04	1.52E-03
**MRPL53**	1.26	2.39	4.28E-04	1.52E-03
**LYRM4**	-0.37	0.78	4.28E-04	1.53E-03
**HIC1**	1.14	2.20	4.30E-04	1.53E-03
**TBL3**	-0.30	0.81	4.30E-04	1.53E-03
**ST13**	0.23	1.17	4.31E-04	1.53E-03
**BCL3**	0.37	1.30	4.31E-04	1.53E-03
**CIAPIN1**	0.26	1.20	4.31E-04	1.53E-03
**SCRN1**	-0.21	0.87	4.32E-04	1.54E-03
**TRIM2**	-0.31	0.81	4.33E-04	1.54E-03
**WDYHV1**	-0.69	0.62	4.33E-04	1.54E-03
**GSDMC**	-0.31	0.81	4.33E-04	1.54E-03
**USP6NL**	-0.30	0.81	4.35E-04	1.55E-03
**SLC25A15**	-0.47	0.72	4.35E-04	1.55E-03
**RP11-660L16.2**	-0.92	0.53	4.36E-04	1.55E-03
**PPA2**	-0.31	0.81	4.37E-04	1.55E-03
**TMC4**	0.59	1.51	4.38E-04	1.56E-03
**KCTD20**	-0.27	0.83	4.38E-04	1.56E-03
**SLC52A3**	-0.69	0.62	4.40E-04	1.56E-03
**AJAP1**	0.38	1.30	4.41E-04	1.57E-03
**SLC8A1**	-1.23	0.43	4.43E-04	1.57E-03
**CTSB**	0.20	1.15	4.44E-04	1.58E-03
**SH3BP5L**	0.30	1.23	4.45E-04	1.58E-03
**MARK1**	-0.48	0.72	4.46E-04	1.58E-03
**PIP4K2A**	-0.40	0.76	4.47E-04	1.59E-03
**AP4E1**	0.32	1.25	4.47E-04	1.59E-03
**CCDC57**	0.39	1.31	4.49E-04	1.59E-03
**CAND1**	-0.25	0.84	4.50E-04	1.60E-03
**MYT1**	1.49	2.81	4.51E-04	1.60E-03
**MSLN**	-0.28	0.82	4.52E-04	1.60E-03
**MOAP1**	0.46	1.38	4.52E-04	1.60E-03
**BRMS1L**	0.47	1.38	4.53E-04	1.60E-03
**STOML1**	-0.60	0.66	4.53E-04	1.60E-03
**MT1F**	-1.15	0.45	4.55E-04	1.61E-03
**HERC4**	0.30	1.23	4.55E-04	1.61E-03
**SLC29A2**	-0.70	0.62	4.56E-04	1.61E-03
**UNC5B-AS1**	1.47	2.77	4.58E-04	1.62E-03
**RP11-159G9.5**	0.77	1.71	4.58E-04	1.62E-03
**SH3BGRL**	0.33	1.26	4.59E-04	1.62E-03
**UCKL1**	-0.39	0.76	4.60E-04	1.63E-03
**LINC01151**	1.40	2.64	4.62E-04	1.63E-03
**POLD4**	0.67	1.60	4.62E-04	1.63E-03
**RAVER2**	-0.41	0.75	4.63E-04	1.64E-03
**KANSL3**	-0.29	0.82	4.64E-04	1.64E-03
**CAPS2**	1.42	2.68	4.64E-04	1.64E-03
**TRO**	1.30	2.47	4.65E-04	1.64E-03
**RP11-543P15.1**	-0.86	0.55	4.65E-04	1.64E-03
**CD2BP2**	0.26	1.20	4.65E-04	1.64E-03
**C11orf58**	0.24	1.18	4.66E-04	1.65E-03
**ATAD3C**	1.14	2.20	4.67E-04	1.65E-03
**MON1A**	-0.36	0.78	4.67E-04	1.65E-03
**IQCB1**	-0.50	0.71	4.67E-04	1.65E-03
**TRIM24**	0.33	1.26	4.69E-04	1.65E-03
**NCBP3**	-0.29	0.82	4.72E-04	1.67E-03
**CLCN2**	0.50	1.42	4.74E-04	1.67E-03
**FAM98B**	-0.36	0.78	4.74E-04	1.67E-03
**ARHGAP44**	1.22	2.33	4.74E-04	1.67E-03
**TLK1**	-0.30	0.81	4.75E-04	1.67E-03
**POLK**	-0.40	0.76	4.75E-04	1.67E-03
**TCTEX1D4**	1.49	2.80	4.76E-04	1.68E-03
**CRLS1**	-0.39	0.76	4.79E-04	1.69E-03
**JMJD6**	-0.41	0.75	4.79E-04	1.69E-03
**DZANK1**	0.92	1.89	4.79E-04	1.69E-03
**ACADS**	-0.64	0.64	4.79E-04	1.69E-03
**GMIP**	0.39	1.31	4.80E-04	1.69E-03
**CLDN11**	1.11	2.16	4.80E-04	1.69E-03
**LAMB4**	1.44	2.72	4.83E-04	1.70E-03
**RGS17**	1.48	2.80	4.85E-04	1.71E-03
**TRIOBP**	0.26	1.20	4.85E-04	1.71E-03
**FAM175B**	0.33	1.25	4.86E-04	1.71E-03
**C4orf47**	1.47	2.76	4.87E-04	1.71E-03
**SEC14L5**	1.47	2.77	4.88E-04	1.71E-03
**CSDE1**	-0.20	0.87	4.89E-04	1.72E-03
**LSM2**	-0.31	0.81	4.89E-04	1.72E-03
**IAH1**	-0.35	0.78	4.94E-04	1.74E-03
**TCERG1L**	1.40	2.64	4.95E-04	1.74E-03
**NDUFB11**	0.31	1.24	4.96E-04	1.74E-03
**UBQLN2**	-0.29	0.82	4.96E-04	1.74E-03

**SCRN3**	0.40	1.32	5.00E-04	1.76E-03
**AC093495.4**	1.36	2.57	5.01E-04	1.76E-03
**NT5DC1**	0.32	1.25	5.04E-04	1.77E-03
**CDK5RAP1**	0.34	1.27	5.04E-04	1.77E-03
**ATRN**	0.22	1.16	5.05E-04	1.77E-03
**TMEM203**	-0.38	0.77	5.06E-04	1.77E-03
**SECISBP2**	0.33	1.26	5.07E-04	1.78E-03
**NAA20**	-0.29	0.82	5.07E-04	1.78E-03
**QTRT1**	0.40	1.32	5.08E-04	1.78E-03
**MRPS11**	0.36	1.28	5.10E-04	1.79E-03
**CCDC186**	0.41	1.33	5.13E-04	1.80E-03
**RAB12**	0.32	1.25	5.17E-04	1.81E-03
**PHIP**	-0.32	0.80	5.17E-04	1.81E-03
**AP001046.5**	0.83	1.78	5.19E-04	1.82E-03
**SLC39A8**	-0.34	0.79	5.21E-04	1.82E-03
**DLEU2**	-0.99	0.50	5.21E-04	1.82E-03
**RWDD2B**	0.36	1.29	5.22E-04	1.83E-03
**GLB1L**	0.66	1.58	5.25E-04	1.84E-03
**DGAT2**	-0.52	0.70	5.26E-04	1.84E-03
**DIXDC1**	0.43	1.35	5.26E-04	1.84E-03
**HIST1H4I**	0.83	1.78	5.27E-04	1.84E-03
**NFU1**	0.35	1.27	5.27E-04	1.84E-03
**MTBP**	-0.51	0.70	5.29E-04	1.85E-03
**PAX5**	1.34	2.53	5.29E-04	1.85E-03
**HMGXB4**	-0.32	0.80	5.29E-04	1.85E-03
**PPP1R8**	-0.31	0.81	5.29E-04	1.85E-03
**DGCR6L**	0.43	1.34	5.30E-04	1.85E-03
**EMC8**	-0.32	0.80	5.31E-04	1.85E-03
**ZNF540**	1.46	2.76	5.37E-04	1.87E-03
**TRAPPC1**	0.27	1.20	5.43E-04	1.89E-03
**CMC2**	-0.41	0.75	5.43E-04	1.89E-03
**SH3RF2**	-0.28	0.83	5.44E-04	1.90E-03
**RAD51AP1**	-0.42	0.75	5.44E-04	1.90E-03
**CNDP2**	-0.23	0.85	5.45E-04	1.90E-03
**CDC42EP2**	-0.45	0.73	5.45E-04	1.90E-03
**VEZT**	-0.24	0.84	5.45E-04	1.90E-03
**KSR1**	0.43	1.35	5.45E-04	1.90E-03
**CELF1**	-0.23	0.85	5.46E-04	1.90E-03
**LINC01184**	-0.42	0.75	5.48E-04	1.91E-03
**STK19**	0.58	1.50	5.50E-04	1.91E-03
**TGFB1**	0.31	1.24	5.51E-04	1.92E-03
**NGRN**	-0.38	0.77	5.53E-04	1.92E-03
**DPYSL3**	-1.19	0.44	5.53E-04	1.92E-03
**LRRC61**	0.53	1.44	5.53E-04	1.92E-03
**WDR6**	0.26	1.20	5.56E-04	1.94E-03
**SLC25A40**	-0.51	0.70	5.57E-04	1.94E-03
**HIP1R**	0.24	1.18	5.60E-04	1.95E-03
**CLDN1**	0.21	1.16	5.62E-04	1.95E-03
**AC025335.1**	-0.88	0.54	5.64E-04	1.96E-03
**DDX54**	-0.30	0.81	5.66E-04	1.97E-03
**ZNF334**	1.01	2.01	5.67E-04	1.97E-03
**COX6B1**	0.23	1.18	5.70E-04	1.98E-03
**URI1**	-0.26	0.83	5.73E-04	1.99E-03
**SNAP47**	0.37	1.30	5.77E-04	2.00E-03
**CEP128**	-0.47	0.72	5.77E-04	2.00E-03
**BRD8**	-0.31	0.81	5.78E-04	2.00E-03
**ADIPOR2**	-0.22	0.86	5.79E-04	2.01E-03
**VPS4B**	0.22	1.16	5.80E-04	2.01E-03
**LIN7C**	0.37	1.30	5.81E-04	2.02E-03
**FSTL3**	-0.25	0.84	5.81E-04	2.02E-03
**TJAP1**	0.34	1.27	5.83E-04	2.02E-03
**TMEM230**	0.29	1.22	5.83E-04	2.02E-03
**TMEM92**	0.74	1.67	5.83E-04	2.02E-03
**MPZL3**	0.42	1.34	5.84E-04	2.03E-03
**TSPYL1**	0.24	1.18	5.87E-04	2.03E-03
**JMY**	0.53	1.44	5.90E-04	2.04E-03
**SPATA5**	-0.55	0.68	5.92E-04	2.05E-03
**MYCBP2**	-0.34	0.79	5.93E-04	2.05E-03
**IST1**	0.24	1.18	5.94E-04	2.06E-03
**FPGT**	-0.60	0.66	5.95E-04	2.06E-03
**SLC6A6**	0.30	1.23	5.96E-04	2.06E-03
**CYBRD1**	-0.59	0.67	5.97E-04	2.07E-03
**EPSTI1**	0.74	1.67	5.97E-04	2.07E-03
**SEMA3D**	-0.76	0.59	5.98E-04	2.07E-03
**PIK3AP1**	-1.44	0.37	6.00E-04	2.08E-03
**LINC00667**	0.41	1.33	6.03E-04	2.09E-03
**ARMCX5**	-0.52	0.70	6.04E-04	2.09E-03
**NUDT2**	-0.53	0.69	6.06E-04	2.09E-03
**OGFOD3**	-0.39	0.76	6.07E-04	2.10E-03
**HAUS1**	-0.39	0.77	6.08E-04	2.10E-03
**SF3B4**	-0.27	0.83	6.09E-04	2.10E-03
**TLL2**	1.45	2.74	6.09E-04	2.10E-03
**MYO1E**	0.21	1.16	6.10E-04	2.11E-03
**TIGD5**	-0.53	0.69	6.10E-04	2.11E-03
**LSM7**	-0.32	0.80	6.10E-04	2.11E-03
**ZNF441**	0.72	1.65	6.14E-04	2.12E-03
**CREM**	0.51	1.43	6.18E-04	2.13E-03
**KIAA1024**	-1.36	0.39	6.19E-04	2.14E-03
**ZNF507**	-0.39	0.76	6.21E-04	2.14E-03
**ALG3**	-0.32	0.80	6.22E-04	2.15E-03

**RGS19**	-0.42	0.75	6.26E-04	2.16E-03
**CD70**	1.45	2.73	6.27E-04	2.16E-03
**PSMD1**	0.19	1.14	6.30E-04	2.17E-03
**FANCI**	-0.25	0.84	6.30E-04	2.17E-03
**MAGEH1**	0.78	1.72	6.31E-04	2.17E-03
**UBE2G2**	0.25	1.19	6.31E-04	2.17E-03
**CBX1**	-0.25	0.84	6.31E-04	2.17E-03
**ARMT1**	0.34	1.26	6.33E-04	2.18E-03
**SMIM14**	0.46	1.37	6.38E-04	2.20E-03
**NRAS**	-0.25	0.84	6.39E-04	2.20E-03
**RAD1**	-0.38	0.77	6.40E-04	2.20E-03
**RERE**	-0.29	0.82	6.40E-04	2.20E-03
**PRRT4**	-1.04	0.49	6.42E-04	2.21E-03
**CCNL1**	0.33	1.26	6.42E-04	2.21E-03
**PMPCA**	0.28	1.21	6.42E-04	2.21E-03
**CDC16**	-0.28	0.82	6.44E-04	2.21E-03
**SLF1**	-0.46	0.73	6.44E-04	2.22E-03
**NUDT22**	0.39	1.31	6.45E-04	2.22E-03
**ERAL1**	0.26	1.20	6.46E-04	2.22E-03
**TMEM94**	0.30	1.23	6.48E-04	2.23E-03
**CSRP2BP**	-0.42	0.75	6.49E-04	2.23E-03
**LIMA1**	0.20	1.15	6.49E-04	2.23E-03
**STAR**	-1.37	0.39	6.51E-04	2.23E-03
**DSTN**	0.28	1.22	6.51E-04	2.23E-03
**DENND4C**	0.30	1.23	6.51E-04	2.23E-03
**LGALS9**	0.78	1.72	6.52E-04	2.24E-03
**COQ7**	-0.49	0.71	6.53E-04	2.24E-03
**NECAP1**	0.32	1.25	6.55E-04	2.25E-03
**ABHD11**	0.43	1.34	6.57E-04	2.25E-03
**GAST**	1.26	2.39	6.60E-04	2.26E-03
**RITA1**	-0.40	0.76	6.63E-04	2.27E-03
**SYT11**	1.43	2.70	6.63E-04	2.27E-03
**NMU**	-0.59	0.67	6.64E-04	2.27E-03
**RIOK2**	-0.39	0.76	6.65E-04	2.28E-03
**USP48**	0.27	1.21	6.69E-04	2.29E-03
**UBALD1**	0.41	1.33	6.70E-04	2.29E-03
**P3H1**	0.36	1.29	6.71E-04	2.30E-03
**SLC27A5**	-0.66	0.63	6.73E-04	2.30E-03
**RAPH1**	-0.72	0.61	6.74E-04	2.31E-03
**UBR5-AS1**	1.18	2.27	6.76E-04	2.31E-03
**STYX**	-0.33	0.79	6.79E-04	2.32E-03
**RP11-832N8.1**	-0.65	0.64	6.81E-04	2.33E-03
**EIF2AK4**	0.22	1.16	6.85E-04	2.34E-03
**CCDC24**	0.68	1.60	6.85E-04	2.34E-03
**AREL1**	0.24	1.18	6.89E-04	2.35E-03
**XRCC4**	-0.54	0.69	6.89E-04	2.36E-03
**DPH5**	-0.43	0.74	6.90E-04	2.36E-03
**ELF4**	-0.26	0.83	6.92E-04	2.36E-03
**CEBPD**	-0.38	0.77	6.94E-04	2.37E-03
**RIMKLB**	-0.33	0.79	6.96E-04	2.38E-03
**TIPARP**	-0.30	0.81	6.96E-04	2.38E-03
**NAA40**	-0.34	0.79	6.96E-04	2.38E-03
**GOLPH3**	-0.27	0.83	6.98E-04	2.38E-03
**LRRC29**	1.34	2.53	6.99E-04	2.38E-03
**LPAR3**	-0.33	0.80	7.02E-04	2.39E-03
**MRPL16**	-0.27	0.83	7.04E-04	2.40E-03
**COL16A1**	0.25	1.19	7.06E-04	2.41E-03
**ANKLE2**	-0.24	0.85	7.08E-04	2.41E-03
**COX6A1**	0.28	1.21	7.11E-04	2.42E-03
**OGFRL1**	-0.31	0.81	7.20E-04	2.45E-03
**C11orf80**	-0.50	0.71	7.21E-04	2.46E-03
**RECQL5**	0.33	1.26	7.22E-04	2.46E-03
**POGZ**	-0.31	0.81	7.23E-04	2.46E-03
**SMAD1**	-0.54	0.69	7.28E-04	2.48E-03
**UBXN8**	-0.52	0.70	7.28E-04	2.48E-03
**EXOC2**	0.28	1.22	7.29E-04	2.48E-03
**ATXN7L3B**	-0.27	0.83	7.30E-04	2.49E-03
**ATP1B1**	0.28	1.21	7.31E-04	2.49E-03
**BTBD6**	-0.30	0.81	7.33E-04	2.50E-03
**VAMP4**	0.51	1.42	7.34E-04	2.50E-03
**RPS23**	-0.29	0.82	7.36E-04	2.50E-03
**OTX1**	-0.59	0.66	7.36E-04	2.50E-03
**LENG8**	0.56	1.47	7.36E-04	2.50E-03
**NUP205**	-0.21	0.86	7.38E-04	2.51E-03
**RNF225**	-1.04	0.49	7.39E-04	2.51E-03
**EGFLAM**	1.01	2.01	7.40E-04	2.52E-03
**CDC25B**	-0.22	0.86	7.40E-04	2.52E-03
**CHD1L**	-0.28	0.83	7.43E-04	2.53E-03
**IZUMO4**	0.77	1.70	7.45E-04	2.53E-03
**C14orf119**	0.28	1.21	7.48E-04	2.54E-03
**MT-ND4**	0.61	1.53	7.49E-04	2.54E-03
**RP13-753N3.1**	1.44	2.70	7.51E-04	2.55E-03
**SAC3D1**	0.44	1.36	7.51E-04	2.55E-03
**C7orf60**	0.62	1.53	7.53E-04	2.56E-03
**ARAP3**	-0.35	0.78	7.57E-04	2.57E-03
**WDR75**	-0.24	0.85	7.60E-04	2.58E-03
**SLC45A4**	-0.47	0.72	7.60E-04	2.58E-03
**INTS8**	0.28	1.21	7.67E-04	2.60E-03
**UTP15**	-0.39	0.76	7.68E-04	2.60E-03
**CYB5RL**	-0.94	0.52	7.72E-04	2.62E-03

ANO9	0.36	1.28	7.72E-04	2.62E-03
CPNE7	-0.64	0.64	7.73E-04	2.62E-03
S100A6	0.44	1.36	7.73E-04	2.62E-03
SHC4	1.43	2.70	7.75E-04	2.62E-03
PFDN2	-0.26	0.83	7.76E-04	2.63E-03
TSGA10	0.75	1.68	7.77E-04	2.63E-03
SYMPK	0.23	1.17	7.77E-04	2.63E-03
SPCS3	-0.29	0.82	7.77E-04	2.63E-03
SUGT1	0.31	1.24	7.80E-04	2.64E-03
MROH8	1.35	2.55	7.80E-04	2.64E-03
NAGS	0.54	1.46	7.83E-04	2.65E-03
SF3B2	-0.19	0.87	7.84E-04	2.65E-03
RANGAP1	0.22	1.17	7.85E-04	2.65E-03
GTPBP3	-0.35	0.78	7.86E-04	2.66E-03
PTPN18	-0.33	0.80	7.90E-04	2.67E-03
AP001062.7	1.08	2.12	7.92E-04	2.68E-03
ACOT4	1.13	2.18	7.93E-04	2.68E-03
FOXRED2	-0.40	0.76	7.94E-04	2.68E-03
SNHG4	-1.13	0.46	7.95E-04	2.68E-03
HIST3H2BB	1.43	2.69	7.96E-04	2.69E-03
TM2D1	0.45	1.36	7.96E-04	2.69E-03
TBCCD1	-0.49	0.71	7.99E-04	2.70E-03
FAM83F	0.26	1.20	7.99E-04	2.70E-03
KIAA0907	0.32	1.24	7.99E-04	2.70E-03
GAK	0.23	1.17	8.01E-04	2.70E-03
WBP1L	0.25	1.19	8.03E-04	2.71E-03
CCNG2	0.58	1.50	8.04E-04	2.71E-03
SLC16A1	-0.22	0.86	8.06E-04	2.72E-03
GSR	0.22	1.17	8.07E-04	2.72E-03
PARS2	0.53	1.45	8.09E-04	2.73E-03
CTD-2376I4.2	1.39	2.61	8.12E-04	2.74E-03
ARL4A	0.50	1.42	8.12E-04	2.74E-03
FCHSD2	0.33	1.26	8.16E-04	2.75E-03
DHTKD1	-0.26	0.84	8.23E-04	2.77E-03
ADPGK	0.30	1.23	8.26E-04	2.78E-03
GRB2	0.23	1.17	8.29E-04	2.79E-03
WDFY1	0.27	1.21	8.34E-04	2.81E-03
NDUFA5	0.35	1.27	8.37E-04	2.82E-03
STYXL1	0.26	1.20	8.38E-04	2.82E-03
CREBRF	1.02	2.03	8.44E-04	2.84E-03
TEAD1	-0.24	0.85	8.45E-04	2.84E-03
ALDH1L1	0.91	1.87	8.45E-04	2.84E-03
PROSER2	0.31	1.24	8.48E-04	2.85E-03
ZFP36	-0.24	0.85	8.49E-04	2.85E-03
CEP19	0.72	1.64	8.49E-04	2.85E-03
RP11-452L6.5	0.93	1.91	8.53E-04	2.87E-03
PDSS1	-0.42	0.75	8.55E-04	2.87E-03
AP1S1	0.24	1.18	8.55E-04	2.87E-03
ICA1L	-0.88	0.54	8.57E-04	2.88E-03
HLA-K	-1.12	0.46	8.59E-04	2.88E-03
CYB5A	-0.34	0.79	8.59E-04	2.88E-03
MBOAT1	-0.66	0.63	8.63E-04	2.89E-03
FAM3D	-1.13	0.46	8.65E-04	2.90E-03
KRT14	-0.19	0.88	8.66E-04	2.90E-03
RGS20	0.39	1.31	8.68E-04	2.91E-03
PPP6C	0.26	1.20	8.69E-04	2.91E-03
RPAIN	0.48	1.40	8.72E-04	2.92E-03
ASIC1	-0.54	0.69	8.73E-04	2.93E-03
FDX1	0.46	1.37	8.76E-04	2.93E-03
TBC1D8B	0.52	1.44	8.77E-04	2.94E-03
IDE	-0.26	0.84	8.77E-04	2.94E-03
SERPING1	-0.60	0.66	8.78E-04	2.94E-03
SLC4A1AP	0.29	1.22	8.78E-04	2.94E-03
GNL2	-0.22	0.86	8.80E-04	2.95E-03
SLC7A2	-0.48	0.72	8.81E-04	2.95E-03
MSANTD4	0.45	1.37	8.84E-04	2.96E-03
EMD	-0.24	0.85	8.85E-04	2.96E-03
RP5-1028K7.2	0.77	1.71	8.86E-04	2.97E-03
TMEM99	0.61	1.52	8.87E-04	2.97E-03
ACP2	0.34	1.27	8.87E-04	2.97E-03
RAMP1	0.91	1.88	8.90E-04	2.98E-03
MED19	0.38	1.30	8.91E-04	2.98E-03
ST3GAL3	-0.62	0.65	8.91E-04	2.98E-03
EXOSC5	-0.43	0.74	8.94E-04	2.99E-03
PPP2R2A	-0.22	0.86	8.94E-04	2.99E-03
ISCA2	-0.45	0.73	8.95E-04	2.99E-03
RP11-120K24.5	1.40	2.65	8.95E-04	2.99E-03
LRR1	-0.46	0.73	8.96E-04	2.99E-03
TTC39B	-0.45	0.73	8.96E-04	2.99E-03
CDIPT	0.33	1.25	8.98E-04	3.00E-03
TOR1AIP2	0.29	1.22	9.00E-04	3.00E-03
HIATL1	-0.21	0.86	9.01E-04	3.01E-03
TMEM135	0.46	1.38	9.01E-04	3.01E-03
MAP2K4	0.26	1.20	9.06E-04	3.02E-03
CLK4	0.50	1.42	9.10E-04	3.03E-03
TRAF3IP2	-0.25	0.84	9.11E-04	3.04E-03
VPS33B	0.32	1.25	9.17E-04	3.06E-03
SLC29A3	1.08	2.11	9.17E-04	3.06E-03
ZNF467	-1.01	0.50	9.23E-04	3.08E-03
CCDC3	1.13	2.18	9.24E-04	3.08E-03

**NCOA6**	-0.24	0.84	9.26E-04	3.08E-03
**PMFBP1**	-1.06	0.48	9.26E-04	3.08E-03
**DYNLL2**	-0.22	0.86	9.33E-04	3.11E-03
**MUC16**	-0.85	0.56	9.33E-04	3.11E-03
**RC3H2**	0.24	1.18	9.36E-04	3.12E-03
**HM13**	0.21	1.15	9.41E-04	3.13E-03
**CRYBG3**	-0.34	0.79	9.42E-04	3.13E-03
**PIEZO1**	-0.23	0.85	9.42E-04	3.13E-03
**TNFAIP2**	0.48	1.40	9.43E-04	3.14E-03
**PRPF39**	-0.40	0.76	9.43E-04	3.14E-03
**FBXL4**	-0.36	0.78	9.44E-04	3.14E-03
**CLPP**	-0.27	0.83	9.45E-04	3.14E-03
**NCSTN**	0.22	1.16	9.45E-04	3.14E-03
**ZMYND8**	-0.24	0.85	9.48E-04	3.15E-03
**CACNB1**	-0.78	0.58	9.50E-04	3.16E-03
**CTC-490G23.2**	-1.41	0.38	9.50E-04	3.16E-03
**GPATCH4**	-0.26	0.83	9.63E-04	3.20E-03
**ZNF346**	0.44	1.35	9.67E-04	3.21E-03
**NPTN**	-0.21	0.87	9.71E-04	3.22E-03
**ADCY4**	1.39	2.63	9.72E-04	3.23E-03
**MTFP1**	-0.92	0.53	9.77E-04	3.24E-03
**DPY19L4**	0.36	1.28	9.80E-04	3.25E-03
**CLDND1**	0.27	1.21	9.82E-04	3.26E-03
**RP11-159D12.8**	-0.85	0.56	9.82E-04	3.26E-03
**RP11-7F17.1**	1.40	2.64	9.84E-04	3.26E-03
**VWA7**	1.07	2.10	9.88E-04	3.28E-03
**LAMTOR4**	0.32	1.25	9.90E-04	3.28E-03
**QRICH2**	1.05	2.07	9.93E-04	3.29E-03
**ZFP3**	0.65	1.56	9.93E-04	3.29E-03
**SUCO**	0.30	1.23	9.93E-04	3.29E-03
**ZNF600**	-0.55	0.68	9.98E-04	3.30E-03
**SMIM10L1**	0.51	1.43	9.98E-04	3.30E-03
**FBXL14**	-0.59	0.66	1.00E-03	3.31E-03
**AKR1B10P1**	1.39	2.62	1.00E-03	3.32E-03
**SPTSSB**	1.13	2.18	1.00E-03	3.32E-03
**POLR2J3**	-0.84	0.56	1.00E-03	3.32E-03
**ZNF197**	0.36	1.29	1.01E-03	3.33E-03
**DVL3**	0.22	1.16	1.01E-03	3.34E-03
**AFMID**	-0.48	0.72	1.01E-03	3.35E-03
**ZSCAN20**	0.62	1.54	1.02E-03	3.36E-03
**UBN1**	-0.21	0.86	1.02E-03	3.36E-03
**EDEM2**	-0.39	0.76	1.02E-03	3.37E-03
**PRRC2B**	0.24	1.18	1.03E-03	3.39E-03
**SFXN3**	-0.40	0.76	1.03E-03	3.41E-03
**LRRC42**	-0.30	0.81	1.04E-03	3.42E-03
**SLC38A10**	0.26	1.19	1.04E-03	3.43E-03
**IFT43**	0.42	1.33	1.04E-03	3.44E-03
**HOOK2**	-0.34	0.79	1.04E-03	3.44E-03
**ZNF821**	1.11	2.15	1.04E-03	3.45E-03
**CEP170**	-0.39	0.76	1.05E-03	3.46E-03
**NID1**	0.42	1.34	1.05E-03	3.46E-03
**SLC20A2**	-0.19	0.88	1.05E-03	3.47E-03
**ZNF438**	-0.70	0.61	1.05E-03	3.47E-03
**RBBP4**	-0.24	0.85	1.05E-03	3.47E-03
**ZC3H11A**	-0.25	0.84	1.05E-03	3.47E-03
**C12orf4**	0.32	1.25	1.05E-03	3.48E-03
**LZTS2**	0.24	1.18	1.06E-03	3.49E-03
**MPP1**	-0.76	0.59	1.06E-03	3.49E-03
**PEG13**	-1.39	0.38	1.08E-03	3.54E-03
**ZNF747**	0.60	1.52	1.08E-03	3.56E-03
**ZNF687**	0.32	1.25	1.08E-03	3.56E-03
**FAM160B2**	0.30	1.23	1.08E-03	3.57E-03
**MT-TC**	0.96	1.95	1.09E-03	3.57E-03
**GLIPR2**	0.60	1.52	1.09E-03	3.58E-03
**ANKRD17**	-0.21	0.87	1.09E-03	3.59E-03
**TXN2**	0.22	1.16	1.09E-03	3.60E-03
**C16orf45**	0.72	1.65	1.09E-03	3.60E-03
**XRCC5**	-0.17	0.89	1.10E-03	3.61E-03
**GDNF-AS1**	1.35	2.54	1.10E-03	3.61E-03
**PBX3**	-0.37	0.77	1.10E-03	3.61E-03
**MYB**	-1.31	0.40	1.10E-03	3.62E-03
**RNF214**	-0.40	0.76	1.10E-03	3.62E-03
**ARL15**	0.42	1.34	1.10E-03	3.62E-03
**ZNF844**	0.88	1.84	1.10E-03	3.62E-03
**GADD45GIP1**	-0.24	0.85	1.10E-03	3.62E-03
**ZKSCAN8**	0.41	1.33	1.10E-03	3.62E-03
**EPN1**	-0.22	0.86	1.11E-03	3.64E-03
**SLC25A44**	0.29	1.22	1.11E-03	3.64E-03
**CUL7**	0.32	1.24	1.11E-03	3.64E-03
**CD9**	-0.18	0.88	1.12E-03	3.68E-03
**MTHFSD**	0.41	1.33	1.12E-03	3.68E-03
**JMJD7-PLA2G4B**	0.68	1.60	1.12E-03	3.68E-03
**AP000432.1**	1.18	2.27	1.12E-03	3.69E-03
**TMEM87B**	0.30	1.23	1.13E-03	3.70E-03
**GPSM3**	-0.55	0.68	1.13E-03	3.71E-03
**LHX4**	1.13	2.19	1.14E-03	3.73E-03
**PTPRB**	-0.97	0.51	1.14E-03	3.73E-03
**POMGNT1**	0.24	1.18	1.14E-03	3.73E-03
**SDR9C7**	0.69	1.61	1.14E-03	3.74E-03
**QPCT**	0.74	1.67	1.14E-03	3.75E-03

**RUNDC1**	0.33	1.26	1.15E-03	3.75E-03
**CCDC91**	-0.40	0.76	1.15E-03	3.76E-03
**UBE2A**	0.25	1.19	1.16E-03	3.79E-03
**HS6ST2**	-0.60	0.66	1.16E-03	3.80E-03
**CDH16**	0.60	1.51	1.16E-03	3.81E-03
**FHOD1**	-0.26	0.83	1.17E-03	3.82E-03
**SIK2**	-0.31	0.81	1.17E-03	3.82E-03
**ANKRD36B**	-1.23	0.43	1.17E-03	3.83E-03
**S100A1**	1.18	2.26	1.17E-03	3.83E-03
**TMEM185A**	0.71	1.64	1.17E-03	3.83E-03
**GSS**	0.22	1.17	1.18E-03	3.85E-03
**ZNF429**	0.78	1.72	1.18E-03	3.86E-03
**ZNF180**	-0.57	0.67	1.18E-03	3.86E-03
**ENKD1**	-0.50	0.71	1.19E-03	3.88E-03
**ARHGAP35**	-0.21	0.86	1.19E-03	3.88E-03
**PIGCP1**	0.83	1.78	1.19E-03	3.88E-03
**SAYSD1**	-0.56	0.68	1.19E-03	3.90E-03
**HECTD4**	0.30	1.23	1.19E-03	3.90E-03
**ELOVL4**	-0.41	0.75	1.20E-03	3.90E-03
**LSM5**	-0.37	0.78	1.20E-03	3.91E-03
**MFSD6**	-0.24	0.84	1.20E-03	3.91E-03
**DMD**	0.41	1.33	1.20E-03	3.91E-03
**RP11-467J12.4**	1.38	2.60	1.20E-03	3.92E-03
**NOP2**	-0.25	0.84	1.20E-03	3.93E-03
**RXRB**	-0.31	0.81	1.20E-03	3.93E-03
**FDXACB1**	-1.03	0.49	1.21E-03	3.94E-03
**CCDC159**	0.78	1.72	1.21E-03	3.95E-03
**CCDC69**	-0.43	0.74	1.21E-03	3.95E-03
**NHP2**	-0.27	0.83	1.21E-03	3.95E-03
**IL17RA**	-0.27	0.83	1.22E-03	3.96E-03
**WDR33**	-0.24	0.85	1.22E-03	3.96E-03
**HEG1**	-0.36	0.78	1.22E-03	3.97E-03
**TRIM44**	-0.23	0.85	1.22E-03	3.97E-03
**GALNT7**	0.35	1.27	1.22E-03	3.98E-03
**VAV2**	0.24	1.18	1.22E-03	3.98E-03
**CALM2**	-0.23	0.85	1.22E-03	3.98E-03
**KPNA6**	0.23	1.17	1.23E-03	4.00E-03
**ERN1**	0.37	1.29	1.23E-03	4.00E-03
**RRP7BP**	-0.64	0.64	1.23E-03	4.00E-03
**WDR13**	-0.29	0.82	1.23E-03	4.01E-03
**ZNF658**	0.81	1.76	1.23E-03	4.01E-03
**NCOA3**	0.24	1.18	1.24E-03	4.02E-03
**SASH1**	0.28	1.22	1.24E-03	4.03E-03
**ZNF142**	-0.31	0.81	1.24E-03	4.03E-03
**MYO9A**	-0.30	0.81	1.24E-03	4.04E-03
**ACVR1B**	-0.29	0.82	1.24E-03	4.04E-03
**BCDIN3D**	0.84	1.79	1.25E-03	4.05E-03
**PXMP4**	0.42	1.33	1.25E-03	4.07E-03
**FUBP3**	0.22	1.17	1.26E-03	4.08E-03
**NDUFA3**	0.30	1.23	1.26E-03	4.08E-03
**ARFIP2**	0.25	1.19	1.26E-03	4.09E-03
**FAM3A**	0.30	1.24	1.26E-03	4.09E-03
**GABPB1-AS1**	0.51	1.43	1.27E-03	4.11E-03
**PIGU**	-0.34	0.79	1.27E-03	4.11E-03
**SCAMP5**	1.21	2.32	1.27E-03	4.12E-03
**RECQL**	-0.34	0.79	1.27E-03	4.12E-03
**TNFRSF10D**	0.37	1.30	1.27E-03	4.13E-03
**SPHK1**	0.48	1.39	1.27E-03	4.13E-03
**VIPR1**	0.86	1.81	1.28E-03	4.13E-03
**WDR12**	-0.26	0.83	1.28E-03	4.16E-03
**STRIP2**	0.37	1.29	1.29E-03	4.17E-03
**RP11-104N10.1**	1.37	2.58	1.29E-03	4.17E-03
**GAMT**	0.90	1.87	1.29E-03	4.18E-03
**TRAC**	1.35	2.55	1.29E-03	4.19E-03
**PATL1**	-0.23	0.85	1.30E-03	4.19E-03
**KLHDC10**	0.26	1.19	1.30E-03	4.19E-03
**EIF4E**	-0.30	0.81	1.30E-03	4.20E-03
**KRT40**	1.30	2.46	1.30E-03	4.21E-03
**ITPRIPL2**	-0.37	0.77	1.31E-03	4.23E-03
**POLR3H**	-0.34	0.79	1.31E-03	4.24E-03
**FAF2**	0.23	1.17	1.31E-03	4.25E-03
**UNC45A**	0.23	1.17	1.32E-03	4.26E-03
**NDUFS8**	0.27	1.20	1.32E-03	4.26E-03
**MRPL52**	0.25	1.19	1.32E-03	4.27E-03
**RPUSD2**	-0.45	0.73	1.32E-03	4.27E-03
**TMEM200A**	-1.17	0.44	1.32E-03	4.28E-03
**RP11-284F21.10**	1.37	2.58	1.33E-03	4.31E-03
**CCDC109B**	-0.42	0.75	1.34E-03	4.31E-03
**RAB24**	0.93	1.91	1.34E-03	4.32E-03
**VDAC1**	-0.18	0.88	1.34E-03	4.32E-03
**CEACAM6**	-0.33	0.80	1.34E-03	4.33E-03
**ALKBH6**	0.88	1.84	1.34E-03	4.33E-03
**UEVLD**	0.30	1.23	1.34E-03	4.33E-03
**TNRC18**	-0.21	0.87	1.35E-03	4.35E-03
**OARD1**	-0.42	0.75	1.35E-03	4.35E-03
**AKAP12**	-1.05	0.48	1.35E-03	4.35E-03
**ANKRD39**	-0.70	0.62	1.35E-03	4.36E-03
**DOLPP1**	-0.34	0.79	1.35E-03	4.36E-03
**CCDC25**	0.26	1.20	1.35E-03	4.36E-03
**APBB3**	0.64	1.56	1.36E-03	4.37E-03

TTC19	0.26	1.19	1.36E-03	4.37E-03
PRMT9	0.44	1.35	1.36E-03	4.38E-03
UBE2Z	-0.20	0.87	1.36E-03	4.38E-03
CERS2	-0.26	0.84	1.36E-03	4.38E-03
CYP2D7	-1.25	0.42	1.36E-03	4.39E-03
CENPK	-0.44	0.74	1.36E-03	4.39E-03
SLC7A5P1	-1.34	0.39	1.36E-03	4.39E-03
CCNL2	0.28	1.22	1.37E-03	4.40E-03
POLR3A	0.25	1.19	1.37E-03	4.40E-03
RBM25	-0.22	0.86	1.37E-03	4.41E-03
RPL10AP6	-0.73	0.60	1.37E-03	4.41E-03
SVIL	0.22	1.16	1.38E-03	4.44E-03
ZNF25	-0.60	0.66	1.38E-03	4.45E-03
KLHL28	0.50	1.42	1.39E-03	4.46E-03
ZBED6	-0.55	0.68	1.39E-03	4.47E-03
UBE2Q2	-0.33	0.80	1.39E-03	4.48E-03
KANK2	-0.38	0.77	1.40E-03	4.49E-03
ORAOV1	0.38	1.30	1.40E-03	4.50E-03
HOXA-AS2	0.93	1.91	1.40E-03	4.50E-03
CXorf38	-0.36	0.78	1.41E-03	4.51E-03
ORMDL1	0.34	1.27	1.41E-03	4.52E-03
FGD1	-0.29	0.82	1.41E-03	4.52E-03
GPALPP1	0.38	1.30	1.41E-03	4.53E-03
ZNF596	0.95	1.94	1.41E-03	4.54E-03
PFKFB4	-0.42	0.75	1.42E-03	4.55E-03
GSTM4	1.07	2.10	1.42E-03	4.55E-03
CARF	0.72	1.64	1.42E-03	4.55E-03
HCAR3	1.04	2.06	1.42E-03	4.55E-03
YIPF3	0.27	1.21	1.42E-03	4.55E-03
LSM3	0.26	1.20	1.42E-03	4.55E-03
INTU	0.44	1.36	1.42E-03	4.55E-03
AP5S1	-0.46	0.73	1.42E-03	4.55E-03
LINC00664	1.36	2.56	1.42E-03	4.56E-03
WDR89	-0.55	0.69	1.42E-03	4.56E-03
MRPL45	0.26	1.19	1.43E-03	4.56E-03
MAP2K2	0.21	1.15	1.43E-03	4.57E-03
FAM50A	0.30	1.23	1.43E-03	4.58E-03
PARVB	-0.51	0.70	1.43E-03	4.58E-03
RPGR	-0.64	0.64	1.43E-03	4.58E-03
RMND1	-0.40	0.76	1.44E-03	4.59E-03
MALSU1	-0.33	0.80	1.44E-03	4.60E-03
FAM134C	0.29	1.22	1.44E-03	4.60E-03
ZNF511	-0.57	0.67	1.44E-03	4.60E-03
ATP6V0B	0.23	1.18	1.44E-03	4.60E-03
ZNF75A	0.47	1.39	1.44E-03	4.60E-03
RUFY1	-0.31	0.81	1.44E-03	4.61E-03
RBM48	0.56	1.48	1.45E-03	4.62E-03
RAP2B	0.23	1.17	1.45E-03	4.64E-03
GRB7	0.34	1.27	1.46E-03	4.66E-03
RP4-717I23.3	0.86	1.82	1.47E-03	4.69E-03
CAP2	-0.56	0.68	1.47E-03	4.69E-03
ST5	-0.28	0.82	1.47E-03	4.70E-03
REEP5	-0.21	0.86	1.47E-03	4.70E-03
LZTFL1	-0.48	0.72	1.47E-03	4.70E-03
CTC-281F24.5	0.80	1.74	1.47E-03	4.71E-03
TMEM256-PLSCR3	-0.24	0.85	1.48E-03	4.73E-03
PEF1	-0.24	0.85	1.48E-03	4.73E-03
PTPRS	0.29	1.22	1.49E-03	4.75E-03
ORAI1	-0.30	0.81	1.49E-03	4.76E-03
COQ9	0.26	1.20	1.50E-03	4.77E-03
B3GALNT2	-0.34	0.79	1.50E-03	4.79E-03
XPO7	-0.19	0.88	1.51E-03	4.80E-03
F8	0.88	1.85	1.51E-03	4.80E-03
TMEM51	-0.29	0.82	1.52E-03	4.84E-03
TMEM150A	-0.51	0.70	1.53E-03	4.86E-03
MMP15	-0.39	0.76	1.53E-03	4.87E-03
MIB2	0.30	1.23	1.53E-03	4.88E-03
MRPS14	0.38	1.30	1.53E-03	4.88E-03
BRWD3	0.29	1.22	1.53E-03	4.88E-03
FLII	-0.19	0.88	1.54E-03	4.89E-03
PRELID2	-0.76	0.59	1.54E-03	4.89E-03
B3GALT4	-0.94	0.52	1.54E-03	4.90E-03
CHTF18	-0.27	0.83	1.54E-03	4.90E-03
MT-ATP6	0.58	1.49	1.54E-03	4.91E-03
FZD6	0.23	1.18	1.55E-03	4.92E-03
IFNAR2	-0.66	0.63	1.55E-03	4.92E-03
LRRC47	0.23	1.17	1.55E-03	4.92E-03
SRRM2	-0.18	0.88	1.56E-03	4.94E-03
ST6GAL1	0.35	1.28	1.56E-03	4.96E-03
ATP6V0A2	-0.39	0.76	1.58E-03	5.01E-03
USP38	0.32	1.25	1.58E-03	5.01E-03
ZFYVE28	0.91	1.88	1.58E-03	5.01E-03
ARFGEF2	0.24	1.18	1.58E-03	5.02E-03
PCGF5	0.34	1.27	1.58E-03	5.02E-03
DUS4L	-0.54	0.69	1.58E-03	5.02E-03
GLI1	-1.32	0.40	1.59E-03	5.04E-03
LINC00662	-0.55	0.68	1.60E-03	5.06E-03
KIF17	1.20	2.31	1.60E-03	5.07E-03
SEMA4G	0.74	1.67	1.60E-03	5.08E-03
GOLGA2P5	0.77	1.70	1.60E-03	5.09E-03

**WFDC2**	1.10	2.15	1.61E-03	5.10E-03
**MGST2**	0.31	1.24	1.61E-03	5.10E-03
**B3GNT4**	-0.97	0.51	1.61E-03	5.11E-03
**AK9**	0.77	1.70	1.62E-03	5.12E-03
**MLLT1**	-0.24	0.85	1.62E-03	5.12E-03
**INTS2**	-0.37	0.77	1.62E-03	5.13E-03
**KARS**	-0.17	0.89	1.62E-03	5.13E-03
**PCDHGB7**	-0.87	0.55	1.62E-03	5.13E-03
**COX8A**	0.24	1.18	1.62E-03	5.14E-03
**CTPS2**	-0.39	0.76	1.64E-03	5.19E-03
**OGFR**	-0.29	0.82	1.65E-03	5.22E-03
**NIPBL**	-0.24	0.85	1.65E-03	5.22E-03
**PLEKHM1P**	0.44	1.36	1.65E-03	5.23E-03
**TMEM245**	0.22	1.17	1.65E-03	5.23E-03
**DCTN5**	0.20	1.15	1.66E-03	5.24E-03
**ANKRD10**	0.45	1.37	1.66E-03	5.24E-03
**ADAMTS1**	-0.28	0.82	1.67E-03	5.27E-03
**AGPS**	-0.24	0.85	1.67E-03	5.28E-03
**MAN1A2**	0.26	1.20	1.68E-03	5.30E-03
**DDI2**	-0.26	0.83	1.68E-03	5.30E-03
**CFAP57**	-1.09	0.47	1.68E-03	5.30E-03
**TPD52L2**	-0.19	0.88	1.68E-03	5.32E-03
**RP3-512B11.3**	0.91	1.87	1.69E-03	5.32E-03
**GPAA1**	0.20	1.15	1.69E-03	5.33E-03
**NRSN2-AS1**	0.90	1.87	1.69E-03	5.34E-03
**FBXL19-AS1**	0.71	1.63	1.69E-03	5.34E-03
**GXYLT1**	0.37	1.29	1.69E-03	5.35E-03
**FGD4**	-0.33	0.79	1.70E-03	5.38E-03
**GLE1**	0.23	1.18	1.70E-03	5.38E-03
**EMG1**	-0.54	0.69	1.71E-03	5.38E-03
**ZNF883**	-0.67	0.63	1.71E-03	5.38E-03
**REV3L**	0.32	1.25	1.71E-03	5.38E-03
**ZFP1**	-0.45	0.73	1.71E-03	5.38E-03
**APMAP**	0.21	1.16	1.71E-03	5.38E-03
**LTBP3**	0.38	1.30	1.71E-03	5.38E-03
**AKR1C6P**	1.29	2.44	1.71E-03	5.39E-03
**BAIAP2L1**	0.23	1.17	1.71E-03	5.40E-03
**GNB1L**	-0.56	0.68	1.72E-03	5.41E-03
**CCDC30**	1.29	2.44	1.72E-03	5.43E-03
**TMOD3**	-0.19	0.88	1.73E-03	5.44E-03
**SECTM1**	0.62	1.53	1.73E-03	5.46E-03
**TOR1A**	0.25	1.19	1.74E-03	5.46E-03
**CTNS**	-0.42	0.75	1.74E-03	5.46E-03
**RPL36**	-0.20	0.87	1.74E-03	5.47E-03
**WDR59**	0.27	1.20	1.74E-03	5.47E-03
**CASC3**	-0.24	0.85	1.74E-03	5.47E-03
**PUM2**	-0.22	0.86	1.74E-03	5.47E-03
**MIEN1**	0.32	1.24	1.74E-03	5.48E-03
**CCHCR1**	-0.30	0.81	1.74E-03	5.48E-03
**PIGB**	-0.54	0.69	1.74E-03	5.48E-03
**CDC40**	0.35	1.28	1.75E-03	5.50E-03
**UBFD1**	0.24	1.18	1.75E-03	5.50E-03
**IL11RA**	1.05	2.07	1.75E-03	5.50E-03
**TMEM223**	-0.51	0.70	1.76E-03	5.54E-03
**IRX5**	0.42	1.34	1.76E-03	5.54E-03
**NKIRAS1**	0.53	1.44	1.77E-03	5.55E-03
**CCDC39**	1.31	2.48	1.77E-03	5.55E-03
**RP11-1002K11.1**	-0.49	0.71	1.77E-03	5.55E-03
**RASGEF1A**	-0.58	0.67	1.77E-03	5.55E-03
**ZNF236**	-0.39	0.77	1.77E-03	5.56E-03
**MFNG**	0.78	1.72	1.77E-03	5.56E-03
**MAPK3**	-0.23	0.85	1.77E-03	5.56E-03
**CHP1**	0.19	1.14	1.78E-03	5.57E-03
**ZNRF1**	-0.38	0.77	1.78E-03	5.59E-03
**RPRD2**	-0.24	0.85	1.79E-03	5.60E-03
**DNAJC8**	0.22	1.17	1.79E-03	5.60E-03
**UGCG**	0.24	1.18	1.79E-03	5.61E-03
**RP11-159N11.4**	1.25	2.37	1.79E-03	5.61E-03
**PI4KAP2**	0.65	1.57	1.79E-03	5.62E-03
**THAP6**	0.43	1.34	1.80E-03	5.62E-03
**PPP2R5A**	-0.32	0.80	1.81E-03	5.67E-03
**UXS1**	0.28	1.21	1.82E-03	5.68E-03
**BCL9L**	0.22	1.16	1.82E-03	5.68E-03
**CDKN2A**	-0.54	0.69	1.82E-03	5.68E-03
**LY6K**	0.42	1.34	1.83E-03	5.71E-03
**FAM86HP**	1.29	2.45	1.83E-03	5.73E-03
**ZNRF3**	-0.39	0.76	1.84E-03	5.74E-03
**KIFC3**	0.25	1.19	1.84E-03	5.76E-03
**ERCC6L2**	0.42	1.34	1.84E-03	5.76E-03
**RP11-7F17.8**	1.31	2.48	1.86E-03	5.80E-03
**FMR1**	0.28	1.22	1.86E-03	5.81E-03
**ARFRP1**	-0.32	0.80	1.86E-03	5.81E-03
**SLC5A8**	-1.32	0.40	1.86E-03	5.82E-03
**SOX2**	-0.79	0.58	1.86E-03	5.82E-03
**ADPRHL1**	0.92	1.90	1.86E-03	5.82E-03
**MALL**	-0.29	0.82	1.87E-03	5.83E-03
**MEGF8**	0.29	1.23	1.87E-03	5.83E-03
**MAX**	0.25	1.19	1.88E-03	5.86E-03
**TTLL7**	-0.72	0.61	1.88E-03	5.86E-03
**ZBED2**	0.23	1.17	1.88E-03	5.87E-03

**MTM1**	0.35	1.28	1.89E-03	5.88E-03
**SUPV3L1**	0.29	1.22	1.89E-03	5.89E-03
**BRIX1**	-0.30	0.81	1.89E-03	5.89E-03
**C15orf41**	-0.34	0.79	1.89E-03	5.90E-03
**C3orf14**	-0.46	0.73	1.89E-03	5.90E-03
**EIF2D**	0.24	1.18	1.90E-03	5.91E-03
**AP4B1**	-0.47	0.72	1.90E-03	5.91E-03
**ACAA2**	-0.46	0.73	1.90E-03	5.91E-03
**CTD-2033A16.2**	-0.99	0.50	1.90E-03	5.92E-03
**LYPD6B**	-0.54	0.69	1.91E-03	5.94E-03
**HELB**	-0.87	0.55	1.91E-03	5.94E-03
**ORC4**	0.36	1.28	1.91E-03	5.94E-03
**KIAA1468**	-0.24	0.85	1.91E-03	5.95E-03
**CBWD1**	0.55	1.46	1.91E-03	5.95E-03
**RNASEH2A**	-0.27	0.83	1.91E-03	5.95E-03
**BAIAP3**	1.24	2.37	1.91E-03	5.95E-03
**KCNAB2**	0.93	1.91	1.92E-03	5.96E-03
**BANF1**	-0.25	0.84	1.92E-03	5.96E-03
**S100PBP**	-0.36	0.78	1.93E-03	5.99E-03
**TFCP2**	-0.25	0.84	1.93E-03	5.99E-03
**CTD-2527I21.15**	1.32	2.49	1.93E-03	6.00E-03
**HRAS**	-0.24	0.84	1.93E-03	6.00E-03
**DDX42**	-0.20	0.87	1.93E-03	6.01E-03
**CNKSR1**	0.30	1.23	1.94E-03	6.02E-03
**FBXW11**	0.25	1.19	1.94E-03	6.04E-03
**C11orf70**	0.79	1.73	1.94E-03	6.04E-03
**LGALS8**	0.26	1.20	1.95E-03	6.05E-03
**HIVEP1**	-0.31	0.81	1.95E-03	6.05E-03
**PLB1**	-0.48	0.72	1.95E-03	6.05E-03
**DEAF1**	-0.35	0.78	1.95E-03	6.06E-03
**KLHL6**	1.30	2.47	1.95E-03	6.06E-03
**METAP1**	-0.22	0.86	1.95E-03	6.06E-03
**CEP290**	-0.43	0.74	1.95E-03	6.06E-03
**HINT2**	0.44	1.36	1.96E-03	6.07E-03
**HTRA1**	0.25	1.19	1.96E-03	6.07E-03
**IP6K1**	0.24	1.18	1.96E-03	6.08E-03
**FOXN3-AS1**	1.17	2.25	1.97E-03	6.09E-03
**ANGPT4**	1.27	2.41	1.97E-03	6.09E-03
**IFI44L**	-0.68	0.62	1.97E-03	6.09E-03
**HRSP12**	0.40	1.32	1.97E-03	6.10E-03
**C14orf159**	0.33	1.26	1.97E-03	6.11E-03
**BCL2**	-0.88	0.54	1.97E-03	6.11E-03
**CAMTA1**	0.34	1.27	1.97E-03	6.11E-03
**PRPSAP1**	-0.29	0.82	1.97E-03	6.11E-03
**EIF3B**	-0.16	0.89	1.98E-03	6.13E-03
**UBE4B**	-0.23	0.85	1.99E-03	6.15E-03
**NRIP3**	0.88	1.84	1.99E-03	6.16E-03
**ACTR10**	0.26	1.19	2.00E-03	6.18E-03
**CTSH**	0.30	1.23	2.00E-03	6.19E-03
**DNAH3**	1.13	2.19	2.01E-03	6.21E-03
**LSR**	0.22	1.17	2.01E-03	6.21E-03
**MTX1P1**	1.14	2.21	2.01E-03	6.23E-03
**LRRC75A-AS1**	0.19	1.14	2.01E-03	6.23E-03
**FAM83G**	0.22	1.16	2.02E-03	6.23E-03
**MKL1**	0.26	1.20	2.02E-03	6.24E-03
**TDRD3**	-0.42	0.75	2.02E-03	6.25E-03
**DBP**	0.68	1.61	2.02E-03	6.25E-03
**TCF7**	0.66	1.58	2.02E-03	6.25E-03
**OBFC1**	0.30	1.23	2.04E-03	6.30E-03
**GALNT1**	-0.24	0.85	2.05E-03	6.34E-03
**AP4M1**	-0.45	0.73	2.06E-03	6.35E-03
**ATF6**	0.23	1.17	2.06E-03	6.35E-03
**PDCD1LG2**	0.66	1.58	2.06E-03	6.37E-03
**RP11-195F19.9**	1.05	2.07	2.06E-03	6.37E-03
**GDPGP1**	0.76	1.69	2.07E-03	6.38E-03
**RP11-658F2.8**	0.87	1.82	2.08E-03	6.41E-03
**MT-ND2**	0.56	1.47	2.09E-03	6.43E-03
**PTMAP5**	-0.81	0.57	2.09E-03	6.45E-03
**RAPGEF5**	-0.48	0.71	2.09E-03	6.46E-03
**NACC2**	0.29	1.23	2.10E-03	6.47E-03
**RP11-474O21.5**	1.26	2.39	2.11E-03	6.50E-03
**GAPDH**	-0.15	0.90	2.11E-03	6.51E-03
**WDFY4**	1.30	2.47	2.13E-03	6.57E-03
**CIDECP**	0.60	1.52	2.14E-03	6.58E-03
**DLEU1**	-0.49	0.71	2.14E-03	6.59E-03
**PCAT6**	0.72	1.64	2.14E-03	6.59E-03
**CAMK1D**	-0.88	0.54	2.14E-03	6.60E-03
**TANK**	0.29	1.22	2.14E-03	6.60E-03
**SETD1A**	-0.28	0.83	2.15E-03	6.61E-03
**EFCAB6**	1.27	2.42	2.15E-03	6.61E-03
**PHLDA1**	0.23	1.17	2.15E-03	6.61E-03
**FAM135A**	-0.26	0.84	2.15E-03	6.61E-03
**RPS6KA5**	0.46	1.38	2.16E-03	6.63E-03
**ABCA10**	1.30	2.47	2.16E-03	6.63E-03
**MOB1A**	0.20	1.15	2.16E-03	6.64E-03
**FTH1P8**	0.96	1.95	2.17E-03	6.66E-03
**GATAD1**	0.31	1.24	2.18E-03	6.68E-03
**PLPP5**	-0.40	0.76	2.18E-03	6.69E-03
**WDR7**	0.33	1.26	2.19E-03	6.73E-03
**MRPL54**	-0.36	0.78	2.19E-03	6.73E-03

**IQSEC2**	0.40	1.32	2.20E-03	6.76E-03
**SHPK**	-0.73	0.60	2.21E-03	6.79E-03
**CCDC189**	1.30	2.45	2.21E-03	6.79E-03
**DYRK4**	-0.38	0.77	2.21E-03	6.80E-03
**CPA6**	-1.12	0.46	2.22E-03	6.80E-03
**CDKL1**	-0.82	0.57	2.22E-03	6.80E-03
**MRPL18**	0.21	1.16	2.22E-03	6.82E-03
**TMEM167B**	-0.36	0.78	2.23E-03	6.83E-03
**TBC1D22A**	0.27	1.21	2.23E-03	6.83E-03
**TRAF1**	0.73	1.66	2.23E-03	6.84E-03
**CTSF**	1.02	2.03	2.23E-03	6.84E-03
**CYP27C1**	-0.43	0.74	2.24E-03	6.85E-03
**SOWAHC**	-0.23	0.85	2.24E-03	6.85E-03
**HLF**	-1.28	0.41	2.24E-03	6.86E-03
**SOD1**	0.21	1.16	2.24E-03	6.87E-03
**FAM193B**	0.32	1.25	2.25E-03	6.88E-03
**PHLDA3**	0.23	1.17	2.25E-03	6.90E-03
**DR1**	0.25	1.19	2.25E-03	6.90E-03
**FRMPD2**	1.25	2.37	2.26E-03	6.93E-03
**MLLT4**	-0.19	0.88	2.27E-03	6.95E-03
**C16orf58**	0.31	1.24	2.27E-03	6.95E-03
**GPD1L**	0.39	1.31	2.28E-03	6.96E-03
**PUM1**	-0.19	0.88	2.28E-03	6.97E-03
**RBMXL1**	-0.31	0.80	2.29E-03	7.01E-03
**NIPAL2**	0.34	1.26	2.29E-03	7.01E-03
**RP11-1082L8.3**	1.29	2.45	2.29E-03	7.01E-03
**STAM**	0.27	1.20	2.30E-03	7.03E-03
**FBXO6**	-0.43	0.74	2.30E-03	7.04E-03
**FOXO4**	0.54	1.45	2.30E-03	7.04E-03
**IRX2**	0.26	1.20	2.31E-03	7.05E-03
**PSMD4**	0.19	1.14	2.31E-03	7.05E-03
**ZNF740**	0.30	1.23	2.31E-03	7.05E-03
**GADD45G**	1.25	2.38	2.31E-03	7.05E-03
**RP11-110G21.1**	0.94	1.92	2.31E-03	7.06E-03
**USP9X**	0.19	1.14	2.31E-03	7.07E-03
**SLITRK6**	-0.88	0.54	2.31E-03	7.07E-03
**RIPK1**	0.29	1.23	2.32E-03	7.07E-03
**TMPRSS4**	0.31	1.24	2.32E-03	7.08E-03
**CBLN3**	1.05	2.07	2.33E-03	7.12E-03
**SLC39A4**	-0.40	0.76	2.34E-03	7.14E-03
**INSIG2**	0.38	1.31	2.35E-03	7.15E-03
**RP11-1149O23.2**	1.02	2.02	2.35E-03	7.16E-03
**VLDLR-AS1**	-1.30	0.41	2.35E-03	7.16E-03
**C11orf54**	0.42	1.33	2.35E-03	7.17E-03
**NGFRAP1**	0.21	1.16	2.35E-03	7.17E-03
**KB-1732A1.1**	0.93	1.91	2.35E-03	7.17E-03
**DAXX**	-0.23	0.85	2.36E-03	7.20E-03
**CNNM2**	0.61	1.52	2.37E-03	7.22E-03
**RPL23AP53**	0.86	1.81	2.38E-03	7.24E-03
**ETFDH**	0.31	1.24	2.38E-03	7.26E-03
**LINC00511**	-0.47	0.72	2.40E-03	7.29E-03
**ST8SIA4**	-1.07	0.48	2.40E-03	7.32E-03
**RP11-18H7.1**	1.28	2.43	2.41E-03	7.32E-03
**FOXK2**	-0.19	0.88	2.41E-03	7.34E-03
**ZNF860**	-0.53	0.69	2.42E-03	7.35E-03
**ATRIP**	-0.34	0.79	2.42E-03	7.36E-03
**CHML**	-0.48	0.72	2.42E-03	7.37E-03
**C4orf48**	-0.66	0.63	2.43E-03	7.39E-03
**ARPC5**	-0.21	0.86	2.43E-03	7.40E-03
**STAT6**	-0.18	0.88	2.44E-03	7.41E-03
**TBRG4**	-0.23	0.85	2.45E-03	7.44E-03
**SMAD2**	-0.23	0.85	2.45E-03	7.44E-03
**VTA1**	0.23	1.17	2.45E-03	7.44E-03
**PPP1R3F**	0.78	1.72	2.45E-03	7.44E-03
**METTL6**	0.47	1.38	2.46E-03	7.48E-03
**SMG6**	0.25	1.19	2.48E-03	7.53E-03
**STARD4**	0.58	1.50	2.48E-03	7.54E-03
**NRTN**	-1.22	0.43	2.49E-03	7.54E-03
**DUSP11**	0.25	1.19	2.49E-03	7.55E-03
**FOXP2**	-1.29	0.41	2.49E-03	7.56E-03
**TPD52**	-0.29	0.82	2.50E-03	7.59E-03
**TAF10**	0.25	1.19	2.50E-03	7.59E-03
**ASB6**	0.28	1.21	2.50E-03	7.59E-03
**THRB**	0.46	1.38	2.52E-03	7.63E-03
**LCORL**	-0.61	0.65	2.52E-03	7.65E-03
**CDK6**	-0.21	0.86	2.53E-03	7.66E-03
**AASDHPPT**	0.28	1.21	2.53E-03	7.66E-03
**ADRM1**	0.26	1.20	2.54E-03	7.69E-03
**NCR3LG1**	-0.44	0.74	2.54E-03	7.69E-03
**GPR108**	0.25	1.19	2.54E-03	7.69E-03
**RAB3A**	0.80	1.74	2.54E-03	7.70E-03
**ADAT2**	-0.46	0.73	2.54E-03	7.70E-03
**ZNF674**	0.93	1.91	2.55E-03	7.72E-03
**INIP**	-0.34	0.79	2.55E-03	7.72E-03
**IMPACT**	-0.31	0.81	2.56E-03	7.74E-03
**MAGI3**	-0.38	0.77	2.56E-03	7.75E-03
**ADCK5**	0.44	1.36	2.57E-03	7.77E-03
**CD81**	0.21	1.16	2.57E-03	7.77E-03
**TSHZ3**	0.42	1.34	2.57E-03	7.77E-03
**TLR2**	-0.90	0.54	2.57E-03	7.77E-03

**CTBP1**	-0.25	0.84	2.58E-03	7.80E-03
**PPP4R1L**	0.57	1.48	2.59E-03	7.82E-03
**GAL**	-0.35	0.78	2.59E-03	7.82E-03
**ADGRE5**	0.34	1.27	2.59E-03	7.83E-03
**ASF1B**	-0.32	0.80	2.60E-03	7.85E-03
**AGA**	0.44	1.35	2.60E-03	7.85E-03
**MDN1**	-0.54	0.69	2.60E-03	7.86E-03
**VPS54**	-0.34	0.79	2.60E-03	7.86E-03
**REEP4**	-0.28	0.82	2.61E-03	7.88E-03
**TRIM46**	-0.87	0.55	2.61E-03	7.89E-03
**PPP2R1B**	-0.22	0.86	2.61E-03	7.89E-03
**TBPL1**	0.38	1.30	2.62E-03	7.89E-03
**ANKRD13A**	0.22	1.16	2.62E-03	7.89E-03
**MUC2**	1.27	2.41	2.64E-03	7.97E-03
**KCNC4**	0.55	1.47	2.64E-03	7.97E-03
**BMP2K**	0.32	1.25	2.64E-03	7.98E-03
**ITGA1**	-0.56	0.68	2.65E-03	7.98E-03
**SCCPDH**	0.42	1.34	2.65E-03	7.99E-03
**CXorf57**	-0.93	0.52	2.65E-03	7.99E-03
**NPM1P39**	-1.28	0.41	2.65E-03	7.99E-03
**C11orf30**	0.29	1.22	2.66E-03	8.00E-03
**LINC01003**	-1.26	0.42	2.66E-03	8.00E-03
**STAU2**	0.32	1.25	2.66E-03	8.00E-03
**SNHG8**	0.30	1.23	2.66E-03	8.01E-03
**DMWD**	0.30	1.23	2.66E-03	8.01E-03
**ASL**	-0.36	0.78	2.66E-03	8.02E-03
**ZNF552**	0.50	1.41	2.66E-03	8.02E-03
**AC023115.2**	1.22	2.33	2.67E-03	8.03E-03
**ZNF28**	0.45	1.37	2.67E-03	8.04E-03
**HES1**	-0.25	0.84	2.67E-03	8.04E-03
**LRRC3**	-0.70	0.62	2.68E-03	8.06E-03
**STK40**	0.26	1.20	2.68E-03	8.07E-03
**MCAM**	-0.39	0.76	2.69E-03	8.09E-03
**NDUFA13**	0.56	1.47	2.69E-03	8.10E-03
**TMEM87A**	-0.26	0.83	2.70E-03	8.12E-03
**MAPK9**	0.25	1.19	2.71E-03	8.13E-03
**SLC38A9**	0.36	1.28	2.71E-03	8.13E-03
**CTXN1**	-0.78	0.58	2.71E-03	8.14E-03
**GSTK1**	0.23	1.17	2.71E-03	8.14E-03
**SUCLA2**	0.26	1.20	2.71E-03	8.15E-03
**GAS5**	-0.22	0.86	2.72E-03	8.17E-03
**C18orf21**	0.41	1.33	2.73E-03	8.19E-03
**SORD**	-0.28	0.82	2.73E-03	8.20E-03
**RP11-479G22.8**	-0.82	0.57	2.73E-03	8.21E-03
**ADGRG1**	0.21	1.16	2.74E-03	8.21E-03
**IL32**	0.94	1.92	2.74E-03	8.22E-03
**VAPA**	-0.23	0.86	2.76E-03	8.27E-03
**ROM1**	0.95	1.93	2.77E-03	8.30E-03
**MFSD8**	0.46	1.37	2.77E-03	8.32E-03
**NDUFA12**	0.23	1.17	2.77E-03	8.32E-03
**CLASP1**	0.20	1.15	2.78E-03	8.33E-03
**ANKRD36**	-0.92	0.53	2.79E-03	8.36E-03
**ANKRD37**	-0.84	0.56	2.80E-03	8.38E-03
**SLF2**	-0.31	0.81	2.81E-03	8.40E-03
**CAV1**	-0.14	0.91	2.81E-03	8.40E-03
**OXER1**	1.25	2.38	2.81E-03	8.40E-03
**COG1**	0.32	1.24	2.81E-03	8.41E-03
**LMTK2**	0.23	1.18	2.82E-03	8.43E-03
**KIAA1211L**	0.47	1.38	2.82E-03	8.44E-03
**PKN2**	0.24	1.18	2.82E-03	8.44E-03
**PDE4B**	-0.75	0.60	2.84E-03	8.49E-03
**URB1**	0.22	1.16	2.84E-03	8.50E-03
**TLN1**	-0.19	0.88	2.85E-03	8.52E-03
**CTSO**	0.77	1.70	2.85E-03	8.52E-03
**NKAP**	0.33	1.26	2.86E-03	8.56E-03
**MUS81**	-0.30	0.81	2.86E-03	8.56E-03
**SKAP2**	0.30	1.23	2.86E-03	8.56E-03
**DIMT1**	-0.25	0.84	2.87E-03	8.58E-03
**CLPTM1L**	-0.22	0.86	2.87E-03	8.58E-03
**RABL2A**	0.81	1.75	2.88E-03	8.60E-03
**DHFRL1**	-0.50	0.71	2.89E-03	8.64E-03
**TOMM34**	-0.26	0.84	2.90E-03	8.65E-03
**SERPINB6**	-0.23	0.85	2.91E-03	8.68E-03
**BTN3A2**	-0.52	0.70	2.91E-03	8.68E-03
**FLJ20021**	0.57	1.49	2.91E-03	8.68E-03
**PAPD5**	0.31	1.24	2.92E-03	8.71E-03
**RP11-334C17.5**	1.09	2.13	2.92E-03	8.71E-03
**CBR3**	-1.11	0.46	2.93E-03	8.73E-03
**LRRC56**	1.04	2.06	2.93E-03	8.74E-03
**MAN2A2**	-0.32	0.80	2.93E-03	8.74E-03
**GNL3L**	-0.40	0.76	2.94E-03	8.76E-03
**DMAP1**	0.31	1.24	2.94E-03	8.76E-03
**B4GALT7**	0.30	1.23	2.95E-03	8.79E-03
**NCBP1**	-0.21	0.87	2.95E-03	8.81E-03
**FZD5**	0.37	1.29	2.97E-03	8.85E-03
**NOG**	1.24	2.37	2.98E-03	8.87E-03
**PTTG1IP**	0.19	1.14	2.98E-03	8.89E-03
**DUS3L**	-0.36	0.78	2.98E-03	8.89E-03
**GRAMD4**	-0.28	0.83	2.99E-03	8.89E-03
**CTD-3088G3.8**	1.24	2.37	3.01E-03	8.96E-03

**C15orf57**	0.64	1.56	3.01E-03	8.96E-03
**NSA2**	-0.21	0.87	3.01E-03	8.98E-03
**RPL3L**	-1.24	0.42	3.02E-03	8.98E-03
**STRIP1**	0.25	1.19	3.02E-03	8.99E-03
**RGS14**	-0.33	0.79	3.02E-03	8.99E-03
**COPS8**	0.22	1.17	3.03E-03	9.01E-03
**NLGN4Y**	0.46	1.37	3.03E-03	9.02E-03
**THAP9**	0.77	1.70	3.04E-03	9.03E-03
**RMDN2**	0.84	1.79	3.04E-03	9.03E-03
**LINC01426**	1.24	2.36	3.04E-03	9.04E-03
**PDZD11**	0.24	1.18	3.05E-03	9.07E-03
**SMG1**	-0.44	0.74	3.05E-03	9.07E-03
**MRPL23**	-0.28	0.82	3.05E-03	9.08E-03
**ADTRP**	-0.79	0.58	3.06E-03	9.08E-03
**NUBPL**	-0.36	0.78	3.06E-03	9.09E-03
**DCAF15**	-0.31	0.81	3.06E-03	9.10E-03
**HERPUD2**	-0.28	0.82	3.07E-03	9.11E-03
**UBE2B**	0.31	1.24	3.07E-03	9.12E-03
**HID1**	-0.87	0.55	3.07E-03	9.12E-03
**NBPF3**	-0.52	0.70	3.07E-03	9.13E-03
**KCNS1**	-0.96	0.51	3.08E-03	9.14E-03
**FBXO8**	0.44	1.36	3.08E-03	9.14E-03
**DIS3**	0.23	1.17	3.09E-03	9.17E-03
**RP11-119F19.5**	0.87	1.83	3.10E-03	9.18E-03
**TMOD2**	0.56	1.47	3.10E-03	9.18E-03
**IQGAP1**	0.17	1.12	3.10E-03	9.18E-03
**SGTA**	-0.22	0.86	3.10E-03	9.19E-03
**APOL6**	-0.35	0.78	3.10E-03	9.20E-03
**SMPD3**	1.12	2.18	3.11E-03	9.21E-03
**CEP44**	-0.52	0.70	3.11E-03	9.23E-03
**RHCG**	-0.43	0.74	3.12E-03	9.24E-03
**ZKSCAN5**	0.34	1.26	3.12E-03	9.24E-03
**RBM17**	-0.21	0.87	3.13E-03	9.26E-03
**SRPK2**	-0.26	0.84	3.13E-03	9.27E-03
**EML1**	-0.40	0.76	3.13E-03	9.27E-03
**SCML1**	0.38	1.30	3.13E-03	9.27E-03
**HDHD2**	0.34	1.27	3.14E-03	9.30E-03
**RP11-609N14.4**	-1.23	0.43	3.15E-03	9.31E-03
**GALNT6**	-0.24	0.85	3.16E-03	9.34E-03
**NAA30**	0.29	1.23	3.16E-03	9.35E-03
**LTB4R2**	-0.62	0.65	3.16E-03	9.35E-03
**TAPT1**	0.44	1.36	3.16E-03	9.35E-03
**MTRF1**	0.50	1.42	3.17E-03	9.37E-03
**STX7**	-0.26	0.84	3.17E-03	9.37E-03
**COMMD8**	-0.44	0.74	3.18E-03	9.39E-03
**SEC22A**	0.41	1.33	3.18E-03	9.39E-03
**TRPM6**	1.07	2.10	3.18E-03	9.40E-03
**RALGAPA2**	0.28	1.22	3.18E-03	9.40E-03
**DAG1**	-0.20	0.87	3.19E-03	9.41E-03
**MTA3**	0.26	1.20	3.20E-03	9.46E-03
**C6orf62**	0.18	1.13	3.20E-03	9.46E-03
**TMTC3**	0.25	1.19	3.21E-03	9.47E-03
**TMEM41A**	0.24	1.18	3.21E-03	9.47E-03
**IFI27L1**	0.59	1.51	3.21E-03	9.48E-03
**TRAF3**	-0.27	0.83	3.22E-03	9.50E-03
**EXT2**	0.19	1.14	3.22E-03	9.50E-03
**RP11-147L13.11**	0.51	1.42	3.22E-03	9.51E-03
**DCAF10**	0.29	1.22	3.23E-03	9.52E-03
**GNG11**	-1.19	0.44	3.23E-03	9.52E-03
**GYG1**	0.25	1.19	3.24E-03	9.54E-03
**ZNF837**	0.80	1.74	3.25E-03	9.58E-03
**LCLAT1**	-0.37	0.77	3.26E-03	9.61E-03
**STX10**	-0.26	0.84	3.26E-03	9.61E-03
**SEC23B**	0.18	1.13	3.27E-03	9.65E-03
**PRODH**	0.29	1.22	3.28E-03	9.68E-03
**BCL2L2**	0.24	1.18	3.29E-03	9.68E-03
**PRKAG1**	-0.20	0.87	3.29E-03	9.68E-03
**ABCC5**	0.25	1.19	3.29E-03	9.69E-03
**PPARD**	-0.22	0.86	3.30E-03	9.71E-03
**PPIL1**	-0.25	0.84	3.31E-03	9.73E-03
**NTAN1**	0.33	1.26	3.31E-03	9.73E-03
**MESDC2**	-0.22	0.86	3.31E-03	9.74E-03
**FMN1**	0.44	1.36	3.32E-03	9.77E-03
**PEBP1**	-0.16	0.89	3.32E-03	9.77E-03
**PTPN22**	0.75	1.68	3.32E-03	9.77E-03
**GTF3C4**	-0.22	0.86	3.32E-03	9.77E-03
**PADI1**	-0.76	0.59	3.34E-03	9.82E-03
**TTYH2**	-0.78	0.58	3.35E-03	9.84E-03
**KIZ**	-0.40	0.76	3.37E-03	9.90E-03
**SGSM2**	0.27	1.20	3.38E-03	9.92E-03
**LINC00657**	0.20	1.15	3.40E-03	9.98E-03
**DCAKD**	0.29	1.22	3.41E-03	1.00E-02
**CTBS**	0.51	1.42	3.42E-03	1.00E-02
**ANKRD36C**	-1.16	0.45	3.42E-03	1.00E-02
**RAB5A**	0.26	1.20	3.43E-03	1.01E-02
**FAM160A2**	0.30	1.23	3.43E-03	1.01E-02
**SIRPA**	0.23	1.17	3.44E-03	1.01E-02
**TRIM55**	1.18	2.27	3.44E-03	1.01E-02
**C2orf68**	0.35	1.28	3.44E-03	1.01E-02
**FGR**	1.18	2.27	3.45E-03	1.01E-02

**KDM4D**	-0.77	0.59	3.47E-03	1.02E-02
**CLCN7**	0.31	1.24	3.48E-03	1.02E-02
**SNRPN**	0.67	1.59	3.49E-03	1.02E-02
**THADA**	-0.24	0.85	3.49E-03	1.02E-02
**RP11-767C1.2**	-1.19	0.44	3.49E-03	1.02E-02
**DPM1**	0.26	1.20	3.49E-03	1.02E-02
**TRIM27**	-0.25	0.84	3.50E-03	1.03E-02
**TAPBPL**	0.51	1.42	3.50E-03	1.03E-02
**HSD17B4**	0.22	1.16	3.51E-03	1.03E-02
**CDNF**	1.00	2.00	3.52E-03	1.03E-02
**ING3**	-0.52	0.70	3.52E-03	1.03E-02
**ETFB**	0.31	1.24	3.53E-03	1.03E-02
**HUS1**	-0.34	0.79	3.53E-03	1.03E-02
**ZNF468**	0.39	1.31	3.53E-03	1.03E-02
**HIGD2A**	0.29	1.22	3.54E-03	1.03E-02
**SLU7**	0.27	1.21	3.54E-03	1.04E-02
**ATP7A**	0.37	1.29	3.54E-03	1.04E-02
**HLA-A**	0.19	1.14	3.54E-03	1.04E-02
**SOCS1**	0.68	1.60	3.55E-03	1.04E-02
**MUC21**	-0.79	0.58	3.55E-03	1.04E-02
**DCAF13**	-0.26	0.84	3.55E-03	1.04E-02
**WNT10B**	-1.20	0.44	3.56E-03	1.04E-02
**MOK**	-0.48	0.72	3.56E-03	1.04E-02
**MSX1**	1.22	2.33	3.57E-03	1.04E-02
**CTC-281F24.1**	0.86	1.81	3.57E-03	1.04E-02
**N4BP2L1**	1.24	2.35	3.57E-03	1.04E-02
**SLC47A2**	1.23	2.35	3.59E-03	1.05E-02
**CDPF1**	-0.62	0.65	3.61E-03	1.05E-02
**GLCE**	-0.34	0.79	3.61E-03	1.05E-02
**PCBD1**	-0.27	0.83	3.61E-03	1.05E-02
**SNX3**	-0.20	0.87	3.61E-03	1.05E-02
**FAM207A**	-0.34	0.79	3.62E-03	1.06E-02
**LSM11**	-0.51	0.70	3.63E-03	1.06E-02
**RDH14**	0.46	1.38	3.63E-03	1.06E-02
**COPS4**	0.24	1.18	3.63E-03	1.06E-02
**TTC37**	-0.20	0.87	3.63E-03	1.06E-02
**PLEKHG1**	0.38	1.30	3.64E-03	1.06E-02
**RAB40B**	-0.51	0.70	3.64E-03	1.06E-02
**C1orf56**	0.81	1.75	3.64E-03	1.06E-02
**RNF138**	-0.40	0.76	3.64E-03	1.06E-02
**PTAR1**	-0.30	0.81	3.65E-03	1.06E-02
**ITPRIPL1**	-0.74	0.60	3.65E-03	1.06E-02
**AZI2**	-0.36	0.78	3.65E-03	1.06E-02
**CD83**	0.82	1.76	3.66E-03	1.06E-02
**GTF2H2**	-0.71	0.61	3.67E-03	1.07E-02
**EIF1B**	0.33	1.26	3.67E-03	1.07E-02
**RP4-647C14.2**	1.21	2.31	3.67E-03	1.07E-02
**DNAJC6**	0.74	1.67	3.67E-03	1.07E-02
**AHR**	-0.29	0.82	3.68E-03	1.07E-02
**AKAP10**	0.29	1.22	3.68E-03	1.07E-02
**MKRN1**	0.21	1.16	3.68E-03	1.07E-02
**ABHD6**	0.49	1.41	3.68E-03	1.07E-02
**ZCCHC2**	-0.30	0.81	3.69E-03	1.07E-02
**PCNXL2**	0.34	1.26	3.70E-03	1.07E-02
**TNNI2**	0.47	1.38	3.71E-03	1.08E-02
**B4GALT3**	0.27	1.20	3.72E-03	1.08E-02
**ZNF337**	0.85	1.80	3.72E-03	1.08E-02
**AP000692.10**	1.24	2.36	3.72E-03	1.08E-02
**ARL6IP5**	0.23	1.17	3.73E-03	1.08E-02
**ZNF335**	0.26	1.20	3.75E-03	1.09E-02
**NCDN**	0.28	1.22	3.75E-03	1.09E-02
**CRYBB2P1**	-0.48	0.72	3.75E-03	1.09E-02
**SESTD1**	-0.31	0.80	3.76E-03	1.09E-02
**ATHL1**	-0.80	0.57	3.76E-03	1.09E-02
**RP9P**	-0.76	0.59	3.76E-03	1.09E-02
**LYSMD1**	0.52	1.44	3.76E-03	1.09E-02
**NOC2L**	-0.20	0.87	3.77E-03	1.09E-02
**LAS1L**	-0.21	0.86	3.77E-03	1.09E-02
**HMOX2**	0.18	1.13	3.80E-03	1.10E-02
**LEPR**	-0.62	0.65	3.81E-03	1.10E-02
**PADI4**	1.17	2.25	3.81E-03	1.11E-02
**PQLC3**	-0.50	0.71	3.83E-03	1.11E-02
**TIMM21**	0.27	1.20	3.84E-03	1.11E-02
**RHBDF2**	0.23	1.18	3.84E-03	1.11E-02
**MRPS23**	-0.25	0.84	3.84E-03	1.11E-02
**NPFFR1**	1.23	2.35	3.84E-03	1.11E-02
**TCN2**	0.70	1.63	3.84E-03	1.11E-02
**PPME1**	0.19	1.14	3.85E-03	1.11E-02
**RPL24**	0.15	1.11	3.85E-03	1.11E-02
**FKBP9P1**	-0.68	0.62	3.85E-03	1.11E-02
**ARFGEF3**	0.49	1.41	3.85E-03	1.11E-02
**CRYBA1**	1.17	2.24	3.86E-03	1.12E-02
**FAM199X**	0.24	1.18	3.86E-03	1.12E-02
**SUB1**	0.25	1.19	3.87E-03	1.12E-02
**RTN2**	-0.59	0.66	3.87E-03	1.12E-02
**SPG20**	-0.25	0.84	3.90E-03	1.13E-02
**RHEB**	-0.26	0.83	3.90E-03	1.13E-02
**POU2AF1**	1.20	2.30	3.91E-03	1.13E-02
**RFTN1**	-0.27	0.83	3.91E-03	1.13E-02
**MAT2B**	0.26	1.20	3.91E-03	1.13E-02

PCSK7	0.26	1.20	3.91E-03	1.13E-02
MT1G	-0.98	0.51	3.92E-03	1.13E-02
GK	0.77	1.71	3.93E-03	1.14E-02
PVRL2	0.19	1.14	3.93E-03	1.14E-02
OAT	0.20	1.14	3.93E-03	1.14E-02
RP11-281P23.1	1.23	2.34	3.94E-03	1.14E-02
FGFR1OP2	0.33	1.26	3.94E-03	1.14E-02
DRD2	1.22	2.33	3.95E-03	1.14E-02
HRNR	1.09	2.12	3.95E-03	1.14E-02
RP11-275N1.1	1.23	2.34	3.95E-03	1.14E-02
FILIP1	1.20	2.30	3.96E-03	1.14E-02
PRCP	0.31	1.24	3.97E-03	1.14E-02
RP11-756P10.3	1.06	2.09	3.97E-03	1.14E-02
TRIM69	-0.41	0.75	3.97E-03	1.15E-02
KDELR2	-0.18	0.88	3.98E-03	1.15E-02
CALM2P2	1.10	2.15	3.98E-03	1.15E-02
RASA2	-0.41	0.75	3.98E-03	1.15E-02
ANKRD19P	1.21	2.31	3.99E-03	1.15E-02
LINC01395	1.20	2.30	4.01E-03	1.16E-02
HIP1	0.32	1.25	4.01E-03	1.16E-02
NSFL1C	0.18	1.13	4.02E-03	1.16E-02
NDUFS3	0.24	1.18	4.02E-03	1.16E-02
IBA57	-0.47	0.72	4.03E-03	1.16E-02
STK11IP	-0.32	0.80	4.04E-03	1.16E-02
NAPG	0.27	1.21	4.04E-03	1.16E-02
TMEM5	-0.36	0.78	4.04E-03	1.16E-02
FBXW12	1.22	2.33	4.04E-03	1.16E-02
EPOR	-0.85	0.55	4.06E-03	1.17E-02
MYO15B	1.17	2.25	4.06E-03	1.17E-02
RP11-295P9.3	0.70	1.63	4.06E-03	1.17E-02
RHBDL1	1.00	2.00	4.06E-03	1.17E-02
GGN	1.12	2.18	4.07E-03	1.17E-02
FNDC3B	-0.25	0.84	4.07E-03	1.17E-02
AHCY	0.17	1.12	4.08E-03	1.17E-02
RP11-274B21.2	0.63	1.54	4.09E-03	1.17E-02
BYSL	-0.28	0.82	4.09E-03	1.17E-02
TRPC4AP	0.21	1.16	4.10E-03	1.18E-02
ATG10	-0.50	0.71	4.10E-03	1.18E-02
SLC22A18	0.54	1.45	4.10E-03	1.18E-02
DUS1L	0.20	1.15	4.11E-03	1.18E-02
RP11-517B11.7	-0.83	0.56	4.12E-03	1.18E-02
DERL3	-1.22	0.43	4.13E-03	1.19E-02
C12orf73	0.51	1.43	4.13E-03	1.19E-02
SH3YL1	-0.26	0.84	4.14E-03	1.19E-02
TMUB1	-0.28	0.82	4.14E-03	1.19E-02
BRF1	-0.30	0.81	4.14E-03	1.19E-02
PRKAR2A-AS1	1.08	2.11	4.14E-03	1.19E-02
RWDD4	-0.34	0.79	4.16E-03	1.19E-02
SSPO	1.19	2.29	4.16E-03	1.19E-02
THAP3	-0.49	0.71	4.17E-03	1.19E-02
ABHD10	-0.33	0.80	4.17E-03	1.19E-02
RP11-177C12.1	0.96	1.95	4.17E-03	1.20E-02
ARHGAP5	0.21	1.16	4.17E-03	1.20E-02
SNX18P7	-1.02	0.49	4.19E-03	1.20E-02
KRCC1	0.33	1.25	4.21E-03	1.21E-02
RP11-411K7.1	1.05	2.06	4.21E-03	1.21E-02
LRRC16A	0.24	1.18	4.22E-03	1.21E-02
POLR3F	0.35	1.28	4.22E-03	1.21E-02
ADAM15	-0.16	0.89	4.22E-03	1.21E-02
STAG2	-0.23	0.85	4.23E-03	1.21E-02
CMB9-22P13.1	1.07	2.10	4.23E-03	1.21E-02
MYLK	0.36	1.29	4.25E-03	1.22E-02
POC1B	0.35	1.27	4.25E-03	1.22E-02
FAM193A	-0.31	0.81	4.25E-03	1.22E-02
RPARP-AS1	-0.70	0.61	4.26E-03	1.22E-02
AGTPBP1	-0.38	0.77	4.27E-03	1.22E-02
FCRLA	1.20	2.29	4.27E-03	1.22E-02
C2orf76	-0.69	0.62	4.28E-03	1.22E-02
ARGLU1	0.26	1.19	4.28E-03	1.22E-02
RP4-765C7.2	-0.37	0.77	4.28E-03	1.22E-02
KLK6	0.70	1.62	4.29E-03	1.23E-02
MTFMT	0.34	1.27	4.29E-03	1.23E-02
PLEKHF2	0.30	1.23	4.30E-03	1.23E-02
PYCR2	0.28	1.21	4.30E-03	1.23E-02
EXT1	-0.22	0.86	4.30E-03	1.23E-02
SSUH2	1.15	2.22	4.31E-03	1.23E-02
KATNB1	-0.28	0.82	4.31E-03	1.23E-02
SAP30BP	0.21	1.15	4.32E-03	1.23E-02
FAM180A	-1.00	0.50	4.33E-03	1.23E-02
USP39	-0.21	0.87	4.34E-03	1.24E-02
UNC45B	1.21	2.31	4.34E-03	1.24E-02
CTC-378H22.2	1.15	2.22	4.34E-03	1.24E-02
ZNF213-AS1	0.59	1.50	4.34E-03	1.24E-02
TMEM164	0.21	1.15	4.36E-03	1.24E-02
MED22	-0.28	0.82	4.37E-03	1.25E-02
JMJD1C	-0.24	0.85	4.37E-03	1.25E-02
TAF6L	-0.45	0.73	4.37E-03	1.25E-02
MAP4K5	-0.24	0.84	4.38E-03	1.25E-02
PCID2	-0.29	0.82	4.39E-03	1.25E-02
RPL34	-0.19	0.88	4.39E-03	1.25E-02

**MECOM**	-0.47	0.72	4.40E-03	1.25E-02
**FAM63A**	0.28	1.22	4.40E-03	1.25E-02
**WDR41**	0.25	1.19	4.42E-03	1.26E-02
**NSUN4**	-0.28	0.82	4.44E-03	1.26E-02
**EXOC3L1**	1.10	2.14	4.44E-03	1.26E-02
**KCNN1**	1.21	2.31	4.45E-03	1.27E-02
**GDAP1**	0.35	1.28	4.45E-03	1.27E-02
**ZNF793-AS1**	1.11	2.15	4.45E-03	1.27E-02
**PWP1**	-0.21	0.86	4.46E-03	1.27E-02
**RAB13**	0.22	1.16	4.46E-03	1.27E-02
**LOR**	-1.20	0.44	4.47E-03	1.27E-02
**TXNDC16**	0.63	1.55	4.47E-03	1.27E-02
**KLHL7**	0.27	1.21	4.48E-03	1.27E-02
**C20orf24**	-0.54	0.69	4.49E-03	1.28E-02
**RP11-513G11.4**	1.20	2.30	4.49E-03	1.28E-02
**CERCAM**	0.26	1.20	4.50E-03	1.28E-02
**RPUSD4**	-0.31	0.80	4.50E-03	1.28E-02
**FEM1C**	0.32	1.25	4.51E-03	1.28E-02
**KCNIP2**	1.17	2.25	4.52E-03	1.28E-02
**PKI55**	-0.68	0.62	4.52E-03	1.28E-02
**BTN2A1**	0.36	1.28	4.52E-03	1.28E-02
**TAF2**	0.24	1.18	4.52E-03	1.28E-02
**LGALS9B**	-0.93	0.52	4.53E-03	1.29E-02
**RP11-288L9.4**	1.20	2.30	4.54E-03	1.29E-02
**HTR1D**	0.84	1.79	4.55E-03	1.29E-02
**ZNF646**	0.28	1.22	4.55E-03	1.29E-02
**LXN**	-0.58	0.67	4.55E-03	1.29E-02
**LPAR5**	0.27	1.21	4.56E-03	1.29E-02
**CPD**	0.23	1.17	4.56E-03	1.29E-02
**FIP1L1**	-0.27	0.83	4.57E-03	1.30E-02
**MFN1**	-0.22	0.86	4.57E-03	1.30E-02
**MAP3K3**	0.32	1.25	4.57E-03	1.30E-02
**KIAA0930**	0.18	1.14	4.58E-03	1.30E-02
**KMT2D**	-0.49	0.71	4.59E-03	1.30E-02
**RAB2A**	-0.23	0.85	4.60E-03	1.30E-02
**BNIP1**	-0.41	0.75	4.61E-03	1.31E-02
**HPCAL1**	0.28	1.21	4.62E-03	1.31E-02
**SLC12A9**	0.27	1.21	4.63E-03	1.31E-02
**GORASP1**	0.22	1.17	4.63E-03	1.31E-02
**PIGT**	0.24	1.18	4.63E-03	1.31E-02
**AGL**	0.27	1.21	4.63E-03	1.31E-02
**MZT2A**	-0.25	0.84	4.63E-03	1.31E-02
**TRIB1**	0.29	1.22	4.64E-03	1.31E-02
**CCDC51**	0.26	1.20	4.64E-03	1.31E-02
**CIB1**	0.22	1.16	4.65E-03	1.31E-02
**C3orf38**	0.31	1.24	4.66E-03	1.32E-02
**WDR46**	-0.22	0.86	4.67E-03	1.32E-02
**NFATC2IP**	-0.22	0.86	4.67E-03	1.32E-02
**SCP2**	0.23	1.17	4.67E-03	1.32E-02
**RCOR2**	-0.88	0.55	4.68E-03	1.32E-02
**ZFP30**	-0.53	0.69	4.68E-03	1.32E-02
**TXNDC12**	-0.21	0.87	4.68E-03	1.32E-02
**ZFAND2B**	-0.29	0.82	4.69E-03	1.32E-02
**RPL32P3**	0.72	1.64	4.69E-03	1.32E-02
**TREML2**	-1.20	0.43	4.69E-03	1.33E-02
**VPS18**	0.25	1.19	4.69E-03	1.33E-02
**MRPS27**	-0.18	0.88	4.70E-03	1.33E-02
**TMEM154**	-0.21	0.86	4.70E-03	1.33E-02
**ARIH1**	0.21	1.15	4.72E-03	1.33E-02
**RAB34**	-0.20	0.87	4.73E-03	1.33E-02
**DYNC2H1**	-0.29	0.82	4.73E-03	1.33E-02
**DNAJC11**	0.21	1.16	4.73E-03	1.33E-02
**PPP1R3G**	1.01	2.01	4.74E-03	1.34E-02
**CCDC113**	-0.41	0.75	4.75E-03	1.34E-02
**RPS2P5**	-0.30	0.81	4.76E-03	1.34E-02
**PSMB10**	0.67	1.59	4.76E-03	1.34E-02
**MAP7D1**	-0.16	0.89	4.76E-03	1.34E-02
**RPS12**	0.20	1.15	4.76E-03	1.34E-02
**ABHD17B**	0.45	1.36	4.77E-03	1.34E-02
**MAML1**	-0.24	0.85	4.78E-03	1.35E-02
**FUT1**	0.41	1.33	4.79E-03	1.35E-02
**ATG14**	0.36	1.28	4.79E-03	1.35E-02
**TXLNGY**	0.33	1.25	4.80E-03	1.35E-02
**VRK3**	0.31	1.24	4.80E-03	1.35E-02
**CXXC5**	-0.54	0.69	4.81E-03	1.35E-02
**FKBP3**	0.27	1.21	4.81E-03	1.35E-02
**ATE1-AS1**	1.19	2.29	4.83E-03	1.36E-02
**IPO7**	0.18	1.13	4.84E-03	1.36E-02
**EXD2**	-0.28	0.83	4.84E-03	1.36E-02
**RSF1**	0.27	1.21	4.84E-03	1.36E-02
**NUBP2**	0.22	1.17	4.84E-03	1.36E-02
**LY6G5B**	0.90	1.87	4.85E-03	1.36E-02
**HGSNAT**	0.22	1.17	4.86E-03	1.37E-02
**MALAT1**	0.68	1.60	4.87E-03	1.37E-02
**MYO7A**	1.08	2.11	4.87E-03	1.37E-02
**RRAGC**	0.35	1.27	4.88E-03	1.37E-02
**LINC01057**	0.87	1.83	4.88E-03	1.37E-02
**NBPF1**	0.32	1.25	4.89E-03	1.37E-02
**FAM101B**	-0.46	0.73	4.90E-03	1.38E-02
**MATN1-AS1**	1.00	2.00	4.91E-03	1.38E-02

**SNRK**	0.25	1.19	4.91E-03	1.38E-02
**ASB16**	1.16	2.24	4.92E-03	1.38E-02
**KCTD6**	-0.61	0.66	4.92E-03	1.38E-02
**EP300**	-0.22	0.86	4.93E-03	1.38E-02
**RPL11**	0.15	1.11	4.94E-03	1.39E-02
**GUSBP5**	-1.18	0.44	4.95E-03	1.39E-02
**PON2**	-0.28	0.82	4.95E-03	1.39E-02
**NKD2**	1.20	2.29	4.96E-03	1.39E-02
**DENND2D**	0.31	1.24	4.99E-03	1.40E-02
**PLCG2**	-0.58	0.67	5.00E-03	1.40E-02
**CCND3**	0.21	1.16	5.00E-03	1.40E-02
**CRTAC1**	1.12	2.18	5.01E-03	1.40E-02
**NELFA**	-0.28	0.82	5.02E-03	1.41E-02
**SYCE1L**	0.85	1.81	5.02E-03	1.41E-02
**BZW1**	-0.21	0.86	5.02E-03	1.41E-02
**ZNF813**	-0.50	0.71	5.02E-03	1.41E-02
**RNASEH2C**	0.41	1.33	5.03E-03	1.41E-02
**RP11-110I1.12**	0.87	1.83	5.04E-03	1.41E-02
**KIAA0753**	0.27	1.20	5.05E-03	1.41E-02
**TEX2**	-0.18	0.89	5.05E-03	1.41E-02
**PRKCZ**	0.33	1.26	5.05E-03	1.41E-02
**CLCN4**	0.94	1.92	5.06E-03	1.42E-02
**GSTT1**	0.41	1.33	5.08E-03	1.42E-02
**JUNB**	-0.18	0.88	5.08E-03	1.42E-02
**PSMB7**	0.16	1.12	5.09E-03	1.42E-02
**FAM8A1**	0.34	1.27	5.10E-03	1.43E-02
**ZNF486**	0.47	1.39	5.11E-03	1.43E-02
**C12orf10**	-0.28	0.82	5.11E-03	1.43E-02
**JRK**	-0.34	0.79	5.11E-03	1.43E-02
**PAN3**	-0.30	0.81	5.11E-03	1.43E-02
**EPM2A**	-0.65	0.64	5.11E-03	1.43E-02
**EPS15**	-0.23	0.85	5.12E-03	1.43E-02
**ZNF557**	0.55	1.46	5.13E-03	1.43E-02
**GPR176**	-0.57	0.67	5.13E-03	1.43E-02
**CNPPD1**	-0.25	0.84	5.15E-03	1.44E-02
**CNOT11**	-0.20	0.87	5.15E-03	1.44E-02
**RPS6KA6**	0.64	1.56	5.17E-03	1.44E-02
**PCSK5**	0.39	1.31	5.17E-03	1.44E-02
**STRN**	-0.24	0.85	5.17E-03	1.44E-02
**ATP13A2**	-0.19	0.88	5.18E-03	1.44E-02
**PPP1R12B**	0.39	1.31	5.22E-03	1.46E-02
**POMZP3**	0.74	1.67	5.22E-03	1.46E-02
**ABHD3**	0.41	1.33	5.22E-03	1.46E-02
**CERS5**	0.28	1.21	5.23E-03	1.46E-02
**RARS**	0.17	1.13	5.23E-03	1.46E-02
**CHODL**	1.18	2.27	5.23E-03	1.46E-02
**TMEM65**	0.33	1.26	5.25E-03	1.46E-02
**TNFRSF8**	-1.17	0.44	5.25E-03	1.46E-02
**NACC1**	-0.19	0.88	5.26E-03	1.46E-02
**TNFSF15**	0.80	1.75	5.26E-03	1.46E-02
**NSMCE2**	-0.33	0.79	5.28E-03	1.47E-02
**EGOT**	1.18	2.27	5.28E-03	1.47E-02
**CPSF4**	0.28	1.21	5.28E-03	1.47E-02
**RP1-272L16.1**	-1.06	0.48	5.30E-03	1.47E-02
**ESCO2**	-0.39	0.77	5.30E-03	1.48E-02
**SIRT6**	0.32	1.24	5.31E-03	1.48E-02
**HNRNPA1P33**	0.43	1.34	5.31E-03	1.48E-02
**CRHR1-IT1**	0.44	1.35	5.33E-03	1.48E-02
**NFAT5**	0.25	1.19	5.35E-03	1.49E-02
**ROBO3**	0.32	1.25	5.36E-03	1.49E-02
**ECD**	0.23	1.17	5.37E-03	1.49E-02
**KIF5A**	1.16	2.23	5.37E-03	1.49E-02
**SLC16A1-AS1**	-0.58	0.67	5.37E-03	1.49E-02
**IFNGR1**	0.23	1.18	5.37E-03	1.49E-02
**DPH7**	-0.35	0.79	5.37E-03	1.49E-02
**SEMA6C**	0.74	1.67	5.38E-03	1.50E-02
**HBS1L**	0.22	1.16	5.39E-03	1.50E-02
**TNFSF18**	-1.12	0.46	5.39E-03	1.50E-02
**NUCB1**	0.21	1.15	5.40E-03	1.50E-02
**PIAS3**	-0.23	0.85	5.40E-03	1.50E-02
**FOSB**	0.98	1.97	5.42E-03	1.51E-02
**STEAP2**	0.52	1.43	5.42E-03	1.51E-02
**ZBTB3**	-0.66	0.63	5.43E-03	1.51E-02
**LRP5**	0.21	1.16	5.44E-03	1.51E-02
**TGDS**	0.39	1.31	5.46E-03	1.51E-02
**MPLKIP**	0.29	1.23	5.46E-03	1.51E-02
**BOD1L1**	0.22	1.16	5.47E-03	1.52E-02
**DNTTIP2**	-0.22	0.86	5.47E-03	1.52E-02
**RPL14**	-0.15	0.90	5.49E-03	1.52E-02
**KCNK10**	-1.18	0.44	5.51E-03	1.53E-02
**PCCB**	0.23	1.18	5.52E-03	1.53E-02
**CWC15**	0.24	1.18	5.53E-03	1.53E-02
**YIF1B**	-0.29	0.82	5.53E-03	1.53E-02
**IFT74**	0.36	1.29	5.54E-03	1.53E-02
**EIF3G**	-0.19	0.88	5.54E-03	1.53E-02
**CTD-2034I21.2**	1.18	2.27	5.54E-03	1.54E-02
**TRAF7**	-0.20	0.87	5.55E-03	1.54E-02
**UBA7**	0.62	1.54	5.55E-03	1.54E-02
**COL4A2**	-0.17	0.89	5.56E-03	1.54E-02
**CCDC150**	-0.73	0.60	5.58E-03	1.54E-02

RPF2	-0.22	0.86	5.58E-03	1.54E-02
ARHGEF9	-0.28	0.82	5.60E-03	1.55E-02
ZNF614	-0.46	0.73	5.62E-03	1.56E-02
ARV1	0.31	1.24	5.62E-03	1.56E-02
NDUFA10	0.19	1.14	5.64E-03	1.56E-02
PANK4	-0.29	0.82	5.65E-03	1.56E-02
TEAD2	-0.43	0.74	5.65E-03	1.56E-02
DUS2	-0.29	0.82	5.67E-03	1.57E-02
CBS	-1.16	0.45	5.68E-03	1.57E-02
USP45	-0.41	0.75	5.69E-03	1.57E-02
RP5-1092A3.4	1.17	2.25	5.69E-03	1.57E-02
ESAM	-1.12	0.46	5.69E-03	1.57E-02
HOXC8	-0.75	0.59	5.70E-03	1.58E-02
PYGO2	-0.26	0.83	5.71E-03	1.58E-02
ELOVL7	-0.37	0.77	5.72E-03	1.58E-02
PSMD14	-0.19	0.88	5.73E-03	1.58E-02
ADAMTS17	0.91	1.88	5.73E-03	1.58E-02
CXorf40B	0.33	1.25	5.73E-03	1.58E-02
LINC00184	1.17	2.25	5.74E-03	1.58E-02
GNGT2	1.10	2.14	5.75E-03	1.59E-02
LDLRAP1	0.21	1.16	5.75E-03	1.59E-02
DGKD	-0.42	0.75	5.77E-03	1.59E-02
DHX40	0.28	1.22	5.77E-03	1.59E-02
LGALS3BP	0.19	1.14	5.77E-03	1.59E-02
METTL16	-0.24	0.85	5.78E-03	1.59E-02
SEMA4A	-0.27	0.83	5.79E-03	1.60E-02
HIRA	-0.37	0.77	5.79E-03	1.60E-02
FAM122A	0.32	1.25	5.80E-03	1.60E-02
ALG14	-0.42	0.75	5.81E-03	1.60E-02
GTF2H2C	-0.73	0.60	5.82E-03	1.60E-02
AHI1	-0.43	0.74	5.84E-03	1.61E-02
ZNF670	-0.73	0.60	5.84E-03	1.61E-02
SGK3	-0.68	0.62	5.91E-03	1.63E-02
TMEM161B	0.36	1.28	5.92E-03	1.63E-02
WWOX	-0.49	0.71	5.93E-03	1.63E-02
CLIC4	-0.20	0.87	5.93E-03	1.63E-02
IMPDH2	-0.15	0.90	5.94E-03	1.64E-02
DHRS4L2	0.43	1.35	5.95E-03	1.64E-02
GRAMD3	-0.35	0.78	5.96E-03	1.64E-02
KNTC1	-0.24	0.85	5.96E-03	1.64E-02
DTNBP1	-0.42	0.75	5.97E-03	1.64E-02
FAM175A	-0.64	0.64	5.97E-03	1.64E-02
HELZ	0.23	1.17	5.97E-03	1.64E-02
ZNF592	-0.24	0.85	5.98E-03	1.64E-02
LUZP1	0.17	1.13	5.99E-03	1.65E-02
CNKSR3	-0.39	0.76	6.01E-03	1.65E-02
ZNF763	1.14	2.21	6.02E-03	1.65E-02
CEP89	-0.27	0.83	6.02E-03	1.65E-02
MED28	-0.25	0.84	6.03E-03	1.66E-02
ZNF768	-0.25	0.84	6.03E-03	1.66E-02
PIBF1	-0.42	0.75	6.04E-03	1.66E-02
ACADL	1.16	2.24	6.05E-03	1.66E-02
C12orf65	-0.33	0.79	6.05E-03	1.66E-02
CREB3L4	-0.58	0.67	6.05E-03	1.66E-02
PSMG4	-0.49	0.71	6.06E-03	1.66E-02
TAF4B	-0.42	0.75	6.07E-03	1.67E-02
ERP29	-0.18	0.88	6.07E-03	1.67E-02
RNF44	-0.31	0.81	6.08E-03	1.67E-02
MAPK15	0.81	1.75	6.10E-03	1.67E-02
ZNF862	0.48	1.39	6.10E-03	1.67E-02
RP5-1014D13.2	1.09	2.12	6.10E-03	1.67E-02
ACOT8	0.37	1.29	6.11E-03	1.68E-02
CHPF	0.19	1.14	6.11E-03	1.68E-02
PLA2G4C	1.16	2.23	6.13E-03	1.68E-02
FAM157A	0.93	1.91	6.13E-03	1.68E-02
APOBEC3B	0.58	1.50	6.15E-03	1.68E-02
MMAA	-0.48	0.72	6.15E-03	1.68E-02
PLA2G6	0.56	1.47	6.15E-03	1.69E-02
HLA-DOB	0.90	1.86	6.16E-03	1.69E-02
TMTC2	-0.31	0.81	6.17E-03	1.69E-02
SP1	-0.17	0.89	6.18E-03	1.69E-02
LIMD2	0.56	1.48	6.18E-03	1.69E-02
EFCAB5	1.13	2.18	6.18E-03	1.69E-02
EMC10	0.24	1.18	6.20E-03	1.70E-02
ZMYND15	0.93	1.90	6.22E-03	1.70E-02
ACBD6	0.26	1.20	6.23E-03	1.70E-02
TGM2	-0.81	0.57	6.23E-03	1.70E-02
POMP	0.20	1.15	6.25E-03	1.71E-02
FSCN1	0.17	1.13	6.26E-03	1.71E-02
LY6G6C	0.56	1.47	6.27E-03	1.71E-02
DLG3	-0.28	0.83	6.27E-03	1.71E-02
CCDC77	-0.40	0.76	6.28E-03	1.72E-02
R3HDM1	-0.19	0.87	6.28E-03	1.72E-02
CTSZ	-0.20	0.87	6.28E-03	1.72E-02
HSD17B11	-0.78	0.58	6.29E-03	1.72E-02
CHD9	0.22	1.16	6.29E-03	1.72E-02
RP11-361L15.5	1.13	2.19	6.33E-03	1.73E-02
AC034220.3	0.95	1.93	6.33E-03	1.73E-02
INTS6	-0.24	0.85	6.34E-03	1.73E-02
FSD1	1.09	2.12	6.34E-03	1.73E-02

FAM19A2	1.12	2.17	6.34E-03	1.73E-02
C17orf107	0.88	1.84	6.35E-03	1.73E-02
VPS13B	-0.27	0.83	6.36E-03	1.73E-02
TMEM116	-0.73	0.60	6.36E-03	1.74E-02
ZSCAN18	0.59	1.51	6.38E-03	1.74E-02
ISG20	-0.47	0.72	6.41E-03	1.75E-02
WRAP53	-0.31	0.81	6.42E-03	1.75E-02
CRISPLD2	0.33	1.26	6.48E-03	1.77E-02
ZNF595	-1.03	0.49	6.49E-03	1.77E-02
POMK	-0.58	0.67	6.50E-03	1.77E-02
RPS15	0.15	1.11	6.51E-03	1.78E-02
KIAA0355	0.29	1.22	6.52E-03	1.78E-02
THTPA	0.66	1.58	6.52E-03	1.78E-02
SCML2	-0.92	0.53	6.53E-03	1.78E-02
FRG1	0.29	1.22	6.53E-03	1.78E-02
TRAK2	0.28	1.21	6.53E-03	1.78E-02
COX6C	0.23	1.17	6.54E-03	1.78E-02
DESI1	0.20	1.15	6.54E-03	1.78E-02
ZNF394	0.40	1.32	6.54E-03	1.78E-02
SEMA7A	0.37	1.29	6.54E-03	1.78E-02
RP11-90L1.8	0.81	1.76	6.54E-03	1.78E-02
IFFO2	0.19	1.14	6.54E-03	1.78E-02
TRIM41	0.26	1.20	6.59E-03	1.79E-02
ZMYND19	-0.28	0.82	6.59E-03	1.79E-02
UQCR11	-0.28	0.82	6.59E-03	1.79E-02
LYN	-0.36	0.78	6.59E-03	1.79E-02
ACOX2	-0.91	0.53	6.60E-03	1.79E-02
BACH1	-0.26	0.84	6.61E-03	1.80E-02
WDR19	-0.36	0.78	6.62E-03	1.80E-02
NPAT	-0.31	0.81	6.63E-03	1.80E-02
HDAC7	-0.24	0.85	6.63E-03	1.80E-02
TRIB2	-0.42	0.74	6.63E-03	1.80E-02
MYL6	0.14	1.10	6.64E-03	1.80E-02
AC131097.4	1.16	2.23	6.64E-03	1.80E-02
CCDC96	0.82	1.77	6.64E-03	1.80E-02
CEP70	-0.49	0.71	6.65E-03	1.81E-02
SARS	-0.15	0.90	6.68E-03	1.81E-02
ABHD18	0.36	1.28	6.70E-03	1.82E-02
AGBL1-AS1	1.15	2.22	6.71E-03	1.82E-02
SNAP23	-0.29	0.82	6.73E-03	1.83E-02
PLS3	-0.15	0.90	6.73E-03	1.83E-02
CDC42EP1	-0.25	0.84	6.74E-03	1.83E-02
SLC24A4	1.12	2.17	6.75E-03	1.83E-02
FAM177A1	0.25	1.19	6.77E-03	1.84E-02
RP5-858L17.1	1.14	2.21	6.78E-03	1.84E-02
RP11-484N16.1	1.15	2.22	6.80E-03	1.84E-02
HACL1	0.35	1.27	6.81E-03	1.84E-02
MDFI	0.21	1.16	6.81E-03	1.85E-02
ATP6V1B2	0.18	1.13	6.83E-03	1.85E-02
POLR2J4	0.59	1.51	6.84E-03	1.85E-02
BSG	0.17	1.12	6.89E-03	1.87E-02
AHNAK	-0.45	0.73	6.89E-03	1.87E-02
LANCL2	-0.32	0.80	6.91E-03	1.87E-02
DDX27	-0.19	0.87	6.91E-03	1.87E-02
SLC22A4	1.00	2.01	6.92E-03	1.87E-02
RP11-472K17.3	1.07	2.10	6.92E-03	1.87E-02
ARPC3	0.21	1.15	6.93E-03	1.87E-02
DPP3	0.20	1.15	6.97E-03	1.89E-02
COPS2	0.21	1.16	6.97E-03	1.89E-02
VPS37D	1.01	2.01	7.00E-03	1.89E-02
C3orf62	0.54	1.45	7.01E-03	1.90E-02
PLPP3	0.64	1.56	7.02E-03	1.90E-02
NEDD1	-0.23	0.85	7.02E-03	1.90E-02
MCF2L2	-1.00	0.50	7.03E-03	1.90E-02
TCTN1	0.34	1.26	7.04E-03	1.90E-02
PFN2	-0.18	0.88	7.04E-03	1.90E-02
RSG1	0.59	1.51	7.05E-03	1.91E-02
PAQR5	-0.57	0.68	7.06E-03	1.91E-02
RP1-191J18.66	0.97	1.95	7.07E-03	1.91E-02
MCOLN1	0.38	1.30	7.09E-03	1.91E-02
SENP6	0.21	1.16	7.11E-03	1.92E-02
RP11-541N10.3	0.63	1.55	7.13E-03	1.93E-02
ZNF518B	0.23	1.17	7.14E-03	1.93E-02
C18orf8	0.31	1.24	7.14E-03	1.93E-02
ZNF688	0.51	1.43	7.15E-03	1.93E-02
LINC00052	1.14	2.21	7.16E-03	1.93E-02
ZMAT5	0.51	1.43	7.17E-03	1.93E-02
TSPO	-0.19	0.88	7.17E-03	1.93E-02
NDFIP1	-0.21	0.86	7.18E-03	1.94E-02
GOLGA5	0.21	1.16	7.19E-03	1.94E-02
PSMD11	-0.17	0.89	7.19E-03	1.94E-02
ARID5B	0.33	1.25	7.20E-03	1.94E-02
RP11-398C13.6	-1.14	0.45	7.20E-03	1.94E-02
STYK1	0.66	1.58	7.21E-03	1.94E-02
ENTPD1	-0.70	0.62	7.22E-03	1.95E-02
CAMK2N1	-0.19	0.88	7.23E-03	1.95E-02
SLC40A1	0.89	1.85	7.24E-03	1.95E-02
SRI	-0.22	0.86	7.24E-03	1.95E-02
ZCCHC7	-0.31	0.81	7.25E-03	1.95E-02
RP11-428J1.5	0.66	1.58	7.26E-03	1.95E-02

**LINC01132**	1.13	2.19	7.28E-03	1.96E-02
**CFL1**	0.19	1.14	7.31E-03	1.97E-02
**UCN**	1.06	2.09	7.34E-03	1.98E-02
**NDEL1**	0.19	1.14	7.35E-03	1.98E-02
**KLF11**	-0.39	0.77	7.35E-03	1.98E-02
**EIF5B**	-0.15	0.90	7.36E-03	1.98E-02
**ASCC2**	0.19	1.14	7.38E-03	1.99E-02
**CISD2**	-0.39	0.76	7.40E-03	1.99E-02
**LINC00467**	0.51	1.43	7.40E-03	1.99E-02
**CREG1**	0.23	1.17	7.41E-03	1.99E-02
**DEF6**	0.21	1.16	7.42E-03	1.99E-02
**FKTN**	-0.26	0.84	7.44E-03	2.00E-02
**TAB1**	0.24	1.19	7.45E-03	2.00E-02
**SOCS7**	-0.26	0.83	7.47E-03	2.01E-02
**PERP**	0.14	1.10	7.49E-03	2.01E-02
**UPK1A**	-1.14	0.45	7.50E-03	2.01E-02
**ENGASE**	0.35	1.28	7.50E-03	2.01E-02
**RNF24**	-0.32	0.80	7.54E-03	2.02E-02
**SFT2D1**	0.29	1.22	7.55E-03	2.03E-02
**ZBTB33**	-0.27	0.83	7.56E-03	2.03E-02
**HADH**	0.23	1.17	7.56E-03	2.03E-02
**BRCC3**	-0.25	0.84	7.57E-03	2.03E-02
**RP3-510D11.4**	1.06	2.08	7.59E-03	2.04E-02
**TGM5**	-1.08	0.47	7.60E-03	2.04E-02
**RP11-379B18.5**	0.87	1.83	7.61E-03	2.04E-02
**CDC73**	-0.24	0.85	7.63E-03	2.05E-02
**MNS1**	-0.74	0.60	7.64E-03	2.05E-02
**CSRP1**	0.17	1.13	7.65E-03	2.05E-02
**BOLA1**	-0.46	0.73	7.65E-03	2.05E-02
**LLPH-AS1**	1.11	2.16	7.65E-03	2.05E-02
**RP11-244O19.1**	-0.29	0.82	7.65E-03	2.05E-02
**STOML2**	-0.17	0.89	7.67E-03	2.05E-02
**SUDS3**	0.22	1.16	7.68E-03	2.06E-02
**SMDT1**	0.38	1.30	7.68E-03	2.06E-02
**SEPSECS**	0.40	1.32	7.69E-03	2.06E-02
**HAS1**	1.10	2.15	7.70E-03	2.06E-02
**BLOC1S5**	-0.38	0.77	7.72E-03	2.07E-02
**RNF135**	0.31	1.24	7.72E-03	2.07E-02
**CAPN10**	0.31	1.24	7.72E-03	2.07E-02
**S100A16**	-0.16	0.90	7.74E-03	2.07E-02
**RP11-244M2.1**	-0.73	0.60	7.74E-03	2.07E-02
**KCTD14**	-0.80	0.57	7.75E-03	2.07E-02
**TFEB**	-0.36	0.78	7.76E-03	2.08E-02
**RILPL2**	-0.34	0.79	7.78E-03	2.08E-02
**RPL23AP82**	0.45	1.37	7.79E-03	2.08E-02
**RARRES1**	0.58	1.50	7.82E-03	2.09E-02
**UBR7**	-0.25	0.84	7.82E-03	2.09E-02
**ATP5H**	0.21	1.16	7.84E-03	2.10E-02
**GNG2**	-0.86	0.55	7.85E-03	2.10E-02
**CHMP4B**	0.18	1.13	7.88E-03	2.10E-02
**SLC2A14**	-0.97	0.51	7.89E-03	2.11E-02
**STT3B**	-0.17	0.89	7.89E-03	2.11E-02
**YIF1A**	-0.22	0.86	7.90E-03	2.11E-02
**RP11-640M9.2**	-0.44	0.73	7.90E-03	2.11E-02
**ZNF582**	0.88	1.83	7.91E-03	2.11E-02
**ARL13B**	-0.44	0.74	7.92E-03	2.11E-02
**CTB-175E5.7**	1.13	2.19	7.92E-03	2.11E-02
**NEFM**	0.59	1.51	7.93E-03	2.12E-02
**DHDDS**	0.20	1.15	7.93E-03	2.12E-02
**ARMCX1**	-0.40	0.76	7.94E-03	2.12E-02
**METTL2A**	-0.33	0.80	7.95E-03	2.12E-02
**RBM8A**	-0.20	0.87	7.97E-03	2.13E-02
**GSTO2**	-0.24	0.84	7.97E-03	2.13E-02
**HSBP1L1**	-0.31	0.80	7.97E-03	2.13E-02
**SRRT**	-0.21	0.87	7.98E-03	2.13E-02
**SWT1**	0.65	1.57	7.99E-03	2.13E-02
**P2RY6**	0.79	1.73	8.00E-03	2.13E-02
**COPB2**	0.16	1.12	8.01E-03	2.13E-02
**CXCL17**	1.08	2.11	8.01E-03	2.13E-02
**IL4R**	-0.20	0.87	8.02E-03	2.14E-02
**NFYC**	0.23	1.17	8.02E-03	2.14E-02
**KDM1B**	-0.24	0.85	8.02E-03	2.14E-02
**GTF2IP4**	0.95	1.93	8.02E-03	2.14E-02
**LIN37**	0.91	1.88	8.03E-03	2.14E-02
**IQCD**	1.03	2.04	8.04E-03	2.14E-02
**ARG2**	-0.41	0.75	8.05E-03	2.14E-02
**ST3GAL2**	0.44	1.35	8.06E-03	2.15E-02
**COQ4**	0.23	1.17	8.07E-03	2.15E-02
**RAD50**	-0.19	0.87	8.08E-03	2.15E-02
**SPAG9**	-0.18	0.88	8.10E-03	2.15E-02
**MITF**	0.58	1.49	8.10E-03	2.15E-02
**DSTYK**	0.25	1.19	8.11E-03	2.16E-02
**DHX37**	-0.24	0.85	8.14E-03	2.16E-02
**GGPS1**	-0.29	0.82	8.15E-03	2.17E-02
**FAM200A**	0.43	1.35	8.18E-03	2.17E-02
**NES**	-1.09	0.47	8.20E-03	2.18E-02
**PDCD6IP**	0.17	1.12	8.20E-03	2.18E-02
**MAST2**	0.20	1.15	8.20E-03	2.18E-02
**RP11-303E16.2**	-0.49	0.71	8.22E-03	2.18E-02
**ZNF579**	0.41	1.33	8.23E-03	2.18E-02

**TARSL2**	-0.45	0.73	8.25E-03	2.19E-02
**PRR5-ARHGAP8**	1.11	2.16	8.25E-03	2.19E-02
**HDDC2**	-0.23	0.86	8.26E-03	2.19E-02
**NCAPH2**	-0.23	0.85	8.26E-03	2.19E-02
**NR1D2**	0.31	1.24	8.26E-03	2.19E-02
**ZNF343**	0.42	1.34	8.28E-03	2.20E-02
**PMM2**	-0.28	0.82	8.29E-03	2.20E-02
**KRT75**	-0.83	0.56	8.30E-03	2.20E-02
**PKNOX1**	-0.31	0.81	8.30E-03	2.20E-02
**ENO3**	0.96	1.94	8.31E-03	2.20E-02
**GALNT10**	0.23	1.17	8.32E-03	2.21E-02
**PNPT1**	-0.21	0.87	8.33E-03	2.21E-02
**ZNF431**	0.30	1.23	8.34E-03	2.21E-02
**ITGA2**	0.21	1.16	8.36E-03	2.22E-02
**LGR4**	0.22	1.17	8.37E-03	2.22E-02
**TMEM41B**	-0.32	0.80	8.39E-03	2.22E-02
**PDGFA**	0.31	1.24	8.39E-03	2.22E-02
**FUZ**	0.52	1.44	8.39E-03	2.22E-02
**RGAG1**	1.08	2.12	8.42E-03	2.23E-02
**SF3B5**	0.19	1.14	8.44E-03	2.23E-02
**STRN4**	-0.17	0.89	8.45E-03	2.24E-02
**AMOTL1**	0.15	1.11	8.45E-03	2.24E-02
**RBM26**	-0.20	0.87	8.45E-03	2.24E-02
**NFATC4**	0.72	1.65	8.45E-03	2.24E-02
**RIMS2**	0.90	1.87	8.47E-03	2.24E-02
**USP14**	-0.19	0.88	8.47E-03	2.24E-02
**TTN**	0.59	1.50	8.52E-03	2.25E-02
**RP11-568J23.8**	1.06	2.08	8.53E-03	2.26E-02
**AC003682.17**	1.11	2.16	8.54E-03	2.26E-02
**ZSCAN12P1**	-1.03	0.49	8.54E-03	2.26E-02
**TIMM9**	-0.34	0.79	8.55E-03	2.26E-02
**DENND6A**	-0.29	0.82	8.55E-03	2.26E-02
**PGRMC2**	0.24	1.18	8.56E-03	2.26E-02
**TGS1**	0.24	1.18	8.56E-03	2.26E-02
**FBXO32**	0.87	1.83	8.57E-03	2.26E-02
**NIPAL1**	-0.21	0.86	8.59E-03	2.27E-02
**C19orf48**	-0.44	0.74	8.60E-03	2.27E-02
**CDC14A**	0.69	1.62	8.61E-03	2.27E-02
**ZC3H13**	-0.19	0.88	8.70E-03	2.30E-02
**RICTOR**	0.27	1.21	8.73E-03	2.30E-02
**GTF2F2**	0.21	1.16	8.74E-03	2.31E-02
**TMF1**	0.24	1.18	8.74E-03	2.31E-02
**PEX11A**	0.48	1.40	8.75E-03	2.31E-02
**RAP2A**	0.26	1.20	8.76E-03	2.31E-02
**STX4**	0.21	1.16	8.79E-03	2.32E-02
**EBLN3**	0.24	1.18	8.80E-03	2.32E-02
**RP11-894P9.1**	0.86	1.82	8.82E-03	2.33E-02
**RP11-288C18.1**	-0.79	0.58	8.82E-03	2.33E-02
**CNOT3**	-0.21	0.87	8.83E-03	2.33E-02
**FANCB**	-0.61	0.65	8.83E-03	2.33E-02
**MID1**	0.26	1.20	8.83E-03	2.33E-02
**OVOL1-AS1**	1.03	2.04	8.84E-03	2.33E-02
**ZNF630**	0.87	1.83	8.84E-03	2.33E-02
**MIER1**	0.28	1.21	8.84E-03	2.33E-02
**OXLD1**	0.37	1.29	8.85E-03	2.33E-02
**RAF1**	0.17	1.13	8.86E-03	2.33E-02
**CDYL**	-0.23	0.86	8.86E-03	2.33E-02
**LTBP4**	0.22	1.17	8.88E-03	2.34E-02
**DCTN1**	0.15	1.11	8.90E-03	2.34E-02
**PRIMPOL**	-0.40	0.76	8.93E-03	2.35E-02
**OTUD7A**	1.09	2.13	8.93E-03	2.35E-02
**FYTTD1**	-0.22	0.86	8.94E-03	2.35E-02
**C8orf82**	-0.47	0.72	8.96E-03	2.36E-02
**UBR4**	-0.41	0.75	8.97E-03	2.36E-02
**KDM3A**	0.24	1.18	8.97E-03	2.36E-02
**NMT1**	-0.18	0.88	8.98E-03	2.36E-02
**CHST11**	0.24	1.18	8.99E-03	2.36E-02
**WWTR1**	0.21	1.16	9.00E-03	2.36E-02
**CPNE1**	-0.19	0.87	9.00E-03	2.36E-02
**ELF2**	-0.32	0.80	9.01E-03	2.37E-02
**CLTB**	-0.16	0.89	9.02E-03	2.37E-02
**DPM2**	-0.23	0.85	9.04E-03	2.38E-02
**MTF1**	0.23	1.17	9.05E-03	2.38E-02
**GLRX2**	-0.49	0.71	9.06E-03	2.38E-02
**ALDH3A2**	0.20	1.15	9.08E-03	2.38E-02
**ZC3H15**	-0.18	0.88	9.08E-03	2.38E-02
**SUCLG2**	-0.25	0.84	9.08E-03	2.38E-02
**RP4-541C22.5**	0.87	1.82	9.10E-03	2.39E-02
**CTDNEP1**	-0.21	0.87	9.12E-03	2.39E-02
**CHGB**	0.97	1.96	9.15E-03	2.40E-02
**PAIP2B**	0.34	1.27	9.17E-03	2.40E-02
**FBLIM1**	0.19	1.14	9.19E-03	2.41E-02
**BCAR3**	-0.20	0.87	9.21E-03	2.41E-02
**BBX**	0.18	1.13	9.21E-03	2.41E-02
**SMG9**	0.23	1.17	9.21E-03	2.41E-02
**RASSF8**	-0.37	0.77	9.23E-03	2.42E-02
**WNT5A-AS1**	-1.02	0.49	9.25E-03	2.42E-02
**CCDC84**	0.52	1.44	9.27E-03	2.43E-02
**PCDHGA12**	-0.63	0.65	9.30E-03	2.44E-02
**CNOT2**	-0.20	0.87	9.32E-03	2.44E-02

**CUL4B**	-0.23	0.85	9.34E-03	2.45E-02
**ASF1A**	-0.33	0.80	9.34E-03	2.45E-02
**IRF7**	-0.31	0.81	9.39E-03	2.46E-02
**RP11-390P24.1**	0.91	1.87	9.41E-03	2.46E-02
**NAA10**	-0.27	0.83	9.43E-03	2.47E-02
**MED24**	0.19	1.14	9.44E-03	2.47E-02
**CHGA**	1.02	2.03	9.47E-03	2.48E-02
**HMGN4**	-0.25	0.84	9.53E-03	2.49E-02
**FES**	-0.99	0.50	9.56E-03	2.50E-02
**CACUL1**	0.22	1.17	9.56E-03	2.50E-02
**VARS**	-0.17	0.89	9.56E-03	2.50E-02
**ERH**	-0.18	0.88	9.57E-03	2.50E-02
**CLSTN2**	1.07	2.09	9.57E-03	2.50E-02
**FAM95C**	-0.98	0.51	9.59E-03	2.51E-02
**C5**	-0.73	0.60	9.62E-03	2.51E-02
**PLCD1**	0.16	1.12	9.64E-03	2.52E-02
**KRT16**	-0.12	0.92	9.64E-03	2.52E-02
**ZNF345**	0.95	1.93	9.65E-03	2.52E-02
**PPP1R21**	0.30	1.23	9.67E-03	2.53E-02
**TNS2**	-0.46	0.72	9.68E-03	2.53E-02
**RPL7L1**	-0.16	0.90	9.68E-03	2.53E-02
**MMACHC**	-0.30	0.81	9.71E-03	2.54E-02
**ANGPT1**	-0.98	0.51	9.71E-03	2.54E-02
**DARS**	-0.17	0.89	9.71E-03	2.54E-02
**ABCB6**	0.77	1.71	9.72E-03	2.54E-02
**STK17B**	-0.35	0.78	9.73E-03	2.54E-02
**RPL39L**	0.29	1.22	9.74E-03	2.54E-02
**AC000123.2**	-0.75	0.59	9.75E-03	2.54E-02
**VPS26B**	-0.19	0.88	9.75E-03	2.54E-02
**RP13-631K18.3**	1.01	2.02	9.80E-03	2.56E-02
**ACSS1**	0.63	1.55	9.80E-03	2.56E-02
**TPBG**	-0.19	0.88	9.81E-03	2.56E-02
**CTB-75G16.1**	1.09	2.13	9.81E-03	2.56E-02
**CXCL2**	-0.93	0.53	9.82E-03	2.56E-02
**LRP11**	-0.19	0.87	9.83E-03	2.56E-02
**FRAT2**	-0.44	0.74	9.83E-03	2.56E-02
**RPS15A**	-0.21	0.87	9.86E-03	2.57E-02
**RNF180**	-0.54	0.69	9.87E-03	2.57E-02
**FAHD1**	0.26	1.20	9.87E-03	2.57E-02
**RUVBL1**	-0.17	0.89	9.89E-03	2.58E-02
**ATP5C1**	-0.16	0.90	9.89E-03	2.58E-02
**SUV39H2**	-0.38	0.77	9.89E-03	2.58E-02
**NR4A2**	0.65	1.57	9.91E-03	2.58E-02
**PGAP3**	0.39	1.31	9.91E-03	2.58E-02
**TAF1A**	-0.50	0.71	9.93E-03	2.59E-02
**PFDN5**	0.18	1.13	9.94E-03	2.59E-02
**TET1**	0.69	1.62	9.94E-03	2.59E-02
**CCDC93**	0.22	1.17	9.94E-03	2.59E-02
**ZFP64**	-0.28	0.83	9.95E-03	2.59E-02
**SNIP1**	0.32	1.25	9.99E-03	2.60E-02
**C6orf99**	1.09	2.13	1.00E-02	2.60E-02
**RAD23B**	0.14	1.10	1.00E-02	2.61E-02
**ACVR2A**	0.45	1.36	1.00E-02	2.61E-02
**CAPN12**	0.86	1.82	1.00E-02	2.61E-02
**C4A**	1.09	2.13	1.00E-02	2.61E-02
**EIF4BP6**	-0.59	0.67	1.00E-02	2.61E-02
**RP11-798M19.6**	-1.06	0.48	1.00E-02	2.61E-02
**GALT**	0.38	1.30	1.01E-02	2.61E-02
**KLLN**	0.85	1.80	1.01E-02	2.61E-02
**FAM168B**	-0.17	0.89	1.01E-02	2.62E-02
**TBC1D30**	-0.60	0.66	1.01E-02	2.62E-02
**ZNF473**	-0.33	0.79	1.01E-02	2.62E-02
**MPHOSPH8**	0.21	1.16	1.01E-02	2.62E-02
**COTL1**	-0.17	0.89	1.01E-02	2.63E-02
**FBXO31**	0.25	1.19	1.01E-02	2.63E-02
**DNAJB14**	0.29	1.22	1.01E-02	2.63E-02
**TSNAX**	0.34	1.27	1.01E-02	2.63E-02
**RP11-319G6.1**	1.02	2.03	1.01E-02	2.63E-02
**THAP7**	-0.29	0.82	1.02E-02	2.63E-02
**RP5-1142A6.10**	-1.09	0.47	1.02E-02	2.64E-02
**RMND5B**	0.22	1.17	1.02E-02	2.64E-02
**HBEGF**	0.31	1.24	1.02E-02	2.64E-02
**EXOC1**	0.22	1.16	1.02E-02	2.65E-02
**ATP6V1C2**	-0.87	0.55	1.02E-02	2.65E-02
**SCN3B**	0.88	1.85	1.02E-02	2.65E-02
**ZSCAN2**	0.79	1.73	1.02E-02	2.66E-02
**COA1**	-0.24	0.85	1.03E-02	2.66E-02
**HGFAC**	1.00	2.00	1.03E-02	2.66E-02
**PSMA2**	0.57	1.49	1.03E-02	2.67E-02
**FUT8**	-0.35	0.79	1.03E-02	2.67E-02
**ERCC3**	0.19	1.14	1.03E-02	2.67E-02
**BRAF**	0.30	1.23	1.03E-02	2.67E-02
**NAA50**	-0.16	0.90	1.03E-02	2.68E-02
**PRMT6**	-0.28	0.82	1.04E-02	2.68E-02
**C17orf62**	0.27	1.21	1.04E-02	2.68E-02
**USP2**	0.97	1.96	1.04E-02	2.68E-02
**IL1R1**	-0.49	0.71	1.04E-02	2.68E-02
**DST**	-0.17	0.89	1.04E-02	2.68E-02
**RNF8**	-0.24	0.85	1.04E-02	2.69E-02
**NRBF2**	0.27	1.20	1.04E-02	2.69E-02

**DPH1**	-0.34	0.79	1.04E-02	2.69E-02
**GBP5**	-1.02	0.49	1.04E-02	2.69E-02
**RELB**	0.44	1.35	1.04E-02	2.70E-02
**DNAL4**	0.35	1.27	1.05E-02	2.70E-02
**ENPP2**	0.90	1.87	1.05E-02	2.70E-02
**PLCB3**	-0.16	0.89	1.05E-02	2.71E-02
**CYP2U1**	-0.50	0.71	1.05E-02	2.72E-02
**SLIT2**	0.24	1.18	1.05E-02	2.72E-02
**PIP5K1B**	-0.97	0.51	1.05E-02	2.72E-02
**PNPLA3**	-0.75	0.59	1.05E-02	2.72E-02
**C4orf3**	0.23	1.17	1.06E-02	2.73E-02
**RHOBTB3**	0.39	1.31	1.06E-02	2.73E-02
**CTD-2619J13.13**	-0.77	0.59	1.06E-02	2.73E-02
**ZSCAN12**	-0.43	0.74	1.06E-02	2.73E-02
**AL450992.2**	0.96	1.94	1.06E-02	2.74E-02
**OVGP1**	0.98	1.98	1.06E-02	2.74E-02
**RP11-488P3.1**	0.28	1.21	1.06E-02	2.74E-02
**EXOSC4**	0.28	1.21	1.06E-02	2.75E-02
**BPIFB4**	1.00	2.00	1.07E-02	2.75E-02
**SFTA1P**	-0.75	0.60	1.07E-02	2.75E-02
**ZNF772**	0.41	1.33	1.07E-02	2.75E-02
**ZCCHC9**	-0.31	0.81	1.07E-02	2.75E-02
**TPT1**	-0.16	0.90	1.07E-02	2.76E-02
**CREG2**	0.83	1.78	1.07E-02	2.76E-02
**HAUS3**	-0.37	0.77	1.08E-02	2.77E-02
**EXOC3**	0.22	1.16	1.08E-02	2.77E-02
**CCPG1**	0.64	1.56	1.08E-02	2.77E-02
**RAVER1**	-0.26	0.83	1.08E-02	2.77E-02
**ACTN4**	0.15	1.11	1.08E-02	2.77E-02
**HADHB**	0.16	1.12	1.08E-02	2.77E-02
**LAMTOR1**	-0.20	0.87	1.08E-02	2.77E-02
**RP3-467L1.6**	-1.01	0.50	1.08E-02	2.77E-02
**VDAC2**	0.18	1.14	1.08E-02	2.78E-02
**C9orf16**	0.23	1.18	1.08E-02	2.78E-02
**MICA**	-0.27	0.83	1.08E-02	2.78E-02
**MTHFD2L**	-0.35	0.79	1.08E-02	2.78E-02
**ABHD13**	0.37	1.30	1.08E-02	2.78E-02
**RP11-727A23.5**	1.02	2.03	1.08E-02	2.78E-02
**CCT6A**	-0.15	0.90	1.08E-02	2.79E-02
**LINC00431**	-1.08	0.47	1.09E-02	2.79E-02
**FCF1**	-0.20	0.87	1.09E-02	2.79E-02
**PARD6G**	-0.24	0.85	1.09E-02	2.79E-02
**XRCC6**	-0.14	0.91	1.09E-02	2.80E-02
**RAB35**	-0.20	0.87	1.09E-02	2.80E-02
**STC1**	0.72	1.65	1.09E-02	2.80E-02
**HRH1**	0.28	1.21	1.09E-02	2.81E-02
**ZNF326**	-0.27	0.83	1.09E-02	2.81E-02
**GEMIN8**	0.41	1.33	1.10E-02	2.82E-02
**PLA2G2F**	-0.80	0.57	1.10E-02	2.82E-02
**ITGAV**	0.21	1.16	1.10E-02	2.82E-02
**LRRC1**	-0.23	0.85	1.10E-02	2.82E-02
**ZNF846**	0.69	1.61	1.10E-02	2.83E-02
**NKPD1**	-0.84	0.56	1.10E-02	2.83E-02
**LINC01405**	0.65	1.57	1.11E-02	2.84E-02
**STX1B**	0.88	1.84	1.11E-02	2.84E-02
**TYW3**	-0.25	0.84	1.11E-02	2.84E-02
**NMT2**	0.27	1.20	1.11E-02	2.84E-02
**ANKZF1**	-0.24	0.85	1.11E-02	2.85E-02
**FNBP4**	-0.20	0.87	1.11E-02	2.85E-02
**TFR2**	-1.04	0.49	1.11E-02	2.85E-02
**ZNF784**	-0.50	0.71	1.11E-02	2.85E-02
**NDUFB2**	0.25	1.19	1.12E-02	2.86E-02
**RPA3**	-0.29	0.82	1.12E-02	2.86E-02
**EIF4G1**	-0.14	0.91	1.12E-02	2.87E-02
**FEZ2**	-0.24	0.85	1.12E-02	2.87E-02
**CTD-2650P22.2**	-1.08	0.47	1.12E-02	2.87E-02
**SMC1A**	-0.16	0.90	1.12E-02	2.87E-02
**DCAF11**	-0.24	0.85	1.12E-02	2.87E-02
**DNAH5**	-0.77	0.59	1.12E-02	2.88E-02
**SRY**	0.97	1.96	1.13E-02	2.88E-02
**KIFC2**	0.31	1.24	1.13E-02	2.89E-02
**HSPB11**	0.31	1.24	1.13E-02	2.89E-02
**RTF1**	0.17	1.13	1.13E-02	2.89E-02
**TATDN3**	-0.38	0.77	1.13E-02	2.89E-02
**PRICKLE1**	-0.60	0.66	1.13E-02	2.89E-02
**GLI2**	-1.08	0.47	1.13E-02	2.89E-02
**FAM229A**	1.05	2.08	1.14E-02	2.90E-02
**NUP54**	-0.22	0.86	1.14E-02	2.90E-02
**MB21D2**	-0.30	0.81	1.14E-02	2.91E-02
**OSBPL2**	0.22	1.17	1.14E-02	2.91E-02
**SRRD**	-0.38	0.77	1.14E-02	2.91E-02
**ATR**	-0.22	0.86	1.14E-02	2.91E-02
**NUB1**	0.21	1.16	1.14E-02	2.92E-02
**F2RL1**	-0.17	0.89	1.14E-02	2.92E-02
**SF3A1**	-0.17	0.89	1.14E-02	2.92E-02
**EBF3**	1.06	2.09	1.14E-02	2.92E-02
**DERA**	-0.21	0.86	1.14E-02	2.92E-02
**SMUG1**	0.25	1.19	1.15E-02	2.93E-02
**CLUHP3**	0.40	1.32	1.15E-02	2.93E-02
**ATE1**	-0.24	0.85	1.15E-02	2.94E-02

**MED7**	-0.41	0.75	1.15E-02	2.94E-02
**AC020571.3**	1.06	2.09	1.15E-02	2.94E-02
**S100A4**	0.34	1.27	1.15E-02	2.94E-02
**ZNF777**	-0.27	0.83	1.15E-02	2.94E-02
**PSG8**	0.98	1.98	1.16E-02	2.95E-02
**TBC1D23**	0.27	1.20	1.16E-02	2.95E-02
**SLC37A4**	0.30	1.23	1.16E-02	2.95E-02
**ZNF273**	-0.61	0.66	1.16E-02	2.95E-02
**KAT8**	-0.28	0.82	1.16E-02	2.96E-02
**ZNF41**	0.34	1.26	1.16E-02	2.97E-02
**SERPINI1**	0.70	1.63	1.17E-02	2.97E-02
**H3F3B**	0.13	1.10	1.17E-02	2.97E-02
**VSTM2L**	0.86	1.81	1.17E-02	2.98E-02
**42799**	0.26	1.20	1.17E-02	2.99E-02
**FAM183A**	0.85	1.80	1.17E-02	2.99E-02
**COPS7A**	-0.18	0.88	1.18E-02	2.99E-02
**NT5C3A**	-0.33	0.80	1.18E-02	3.00E-02
**RNFT1**	0.53	1.45	1.18E-02	3.00E-02
**CTD-2319I12.4**	1.06	2.09	1.18E-02	3.01E-02
**MAP4K2**	0.27	1.20	1.18E-02	3.01E-02
**CNIH4**	-0.24	0.85	1.18E-02	3.01E-02
**RP11-561O23.8**	1.06	2.08	1.18E-02	3.01E-02
**CDHR1**	-0.60	0.66	1.19E-02	3.02E-02
**TRIM25**	-0.16	0.89	1.19E-02	3.02E-02
**NBPF9**	-0.47	0.72	1.19E-02	3.03E-02
**CPOX**	0.21	1.16	1.19E-02	3.03E-02
**CDK5**	0.33	1.25	1.19E-02	3.03E-02
**DEPDC5**	0.29	1.22	1.19E-02	3.03E-02
**ZNF275**	0.32	1.25	1.19E-02	3.03E-02
**EIF3EP1**	0.62	1.54	1.20E-02	3.04E-02
**ZNF319**	0.37	1.29	1.20E-02	3.04E-02
**TMEM120B**	0.30	1.23	1.20E-02	3.05E-02
**KPNA5**	0.56	1.47	1.20E-02	3.05E-02
**ZUFSP**	0.33	1.26	1.20E-02	3.05E-02
**CARKD**	0.28	1.22	1.20E-02	3.05E-02
**NPEPL1**	0.50	1.42	1.20E-02	3.06E-02
**FBXO25**	0.30	1.24	1.21E-02	3.07E-02
**KLHDC7B**	-1.07	0.48	1.21E-02	3.07E-02
**LRRC49**	0.43	1.35	1.21E-02	3.07E-02
**SAPCD1**	-1.06	0.48	1.21E-02	3.07E-02
**ZNF720**	0.36	1.28	1.21E-02	3.07E-02
**RP11-304L19.3**	-0.99	0.50	1.21E-02	3.07E-02
**WDR43**	-0.18	0.88	1.21E-02	3.08E-02
**UBE2D3**	-0.14	0.90	1.21E-02	3.08E-02
**CBR1**	-0.19	0.87	1.21E-02	3.08E-02
**USP12**	-0.28	0.82	1.21E-02	3.08E-02
**PLAGL2**	-0.22	0.86	1.22E-02	3.09E-02
**DMXL2**	-0.29	0.82	1.22E-02	3.10E-02
**PRKAR1B**	0.25	1.19	1.22E-02	3.10E-02
**CELSR2**	-0.17	0.89	1.22E-02	3.10E-02
**BDNF**	1.06	2.08	1.22E-02	3.10E-02
**NLRC3**	1.05	2.07	1.22E-02	3.10E-02
**EARS2**	0.21	1.16	1.22E-02	3.10E-02
**MCU**	-0.21	0.87	1.23E-02	3.11E-02
**CCNYL1**	0.24	1.18	1.23E-02	3.11E-02
**PLEKHO1**	-0.60	0.66	1.23E-02	3.11E-02
**RBMS1**	-0.18	0.88	1.23E-02	3.11E-02
**RP3-416H24.1**	1.03	2.04	1.23E-02	3.11E-02
**FSTL1**	0.17	1.13	1.23E-02	3.12E-02
**TRAPPC9**	-0.28	0.82	1.23E-02	3.12E-02
**RP11-65I12.1**	-1.06	0.48	1.23E-02	3.12E-02
**FAM49B**	-0.19	0.87	1.23E-02	3.12E-02
**SLFNL1-AS1**	0.96	1.94	1.24E-02	3.13E-02
**SEMA3C**	-0.22	0.86	1.24E-02	3.13E-02
**ATP13A4**	-0.75	0.59	1.24E-02	3.14E-02
**N4BP1**	0.18	1.13	1.24E-02	3.14E-02
**OSBPL10**	-0.17	0.89	1.24E-02	3.14E-02
**IKBKB**	0.22	1.16	1.24E-02	3.14E-02
**HTRA2**	0.31	1.24	1.24E-02	3.14E-02
**DNASE1L3**	1.06	2.09	1.24E-02	3.15E-02
**ESF1**	-0.25	0.84	1.25E-02	3.15E-02
**CTD-2192J16.15**	-0.67	0.63	1.25E-02	3.15E-02
**NOXA1**	0.88	1.84	1.25E-02	3.15E-02
**FTH1**	0.38	1.31	1.25E-02	3.15E-02
**RBM4B**	-0.40	0.76	1.25E-02	3.15E-02
**ANXA9**	-0.35	0.79	1.25E-02	3.15E-02
**FOLR3**	-1.06	0.48	1.25E-02	3.15E-02
**PCAT1**	0.97	1.96	1.25E-02	3.15E-02
**RP11-958J22.2**	1.06	2.09	1.25E-02	3.16E-02
**CD24P4**	-0.16	0.89	1.25E-02	3.16E-02
**RP5-1198O20.4**	1.05	2.08	1.25E-02	3.16E-02
**NENF**	0.22	1.17	1.25E-02	3.16E-02
**ZNF175**	0.46	1.38	1.26E-02	3.17E-02
**SFN**	0.15	1.11	1.26E-02	3.17E-02
**SLC35F6**	-0.19	0.88	1.26E-02	3.17E-02
**GPATCH11**	-0.33	0.79	1.26E-02	3.17E-02
**FBXW4**	0.26	1.19	1.26E-02	3.18E-02
**ZNF3**	0.29	1.22	1.26E-02	3.18E-02
**ZNF681**	-0.55	0.68	1.26E-02	3.18E-02
**CEP162**	-0.48	0.72	1.26E-02	3.18E-02

**LIPE**	-0.32	0.80	1.26E-02	3.19E-02
**SLC27A3**	-0.40	0.76	1.27E-02	3.19E-02
**PSORS1C1**	0.84	1.79	1.27E-02	3.19E-02
**RIBC1**	0.99	1.98	1.27E-02	3.19E-02
**KRT42P**	-0.76	0.59	1.27E-02	3.20E-02
**RP11-448G15.3**	1.02	2.03	1.27E-02	3.20E-02
**B3GALT6**	-0.36	0.78	1.27E-02	3.21E-02
**SRCAP**	-0.41	0.75	1.27E-02	3.21E-02
**LINC01128**	-0.48	0.72	1.28E-02	3.21E-02
**SMARCA2**	0.19	1.14	1.28E-02	3.21E-02
**CCDC146**	0.80	1.75	1.28E-02	3.21E-02
**FAM86B1**	-0.79	0.58	1.28E-02	3.22E-02
**RBM10**	-0.18	0.88	1.28E-02	3.22E-02
**MPP7**	-0.26	0.83	1.28E-02	3.23E-02
**TMC6**	0.23	1.17	1.28E-02	3.23E-02
**JMJD8**	-0.29	0.82	1.29E-02	3.23E-02
**ZNF695**	-0.97	0.51	1.29E-02	3.23E-02
**PLEKHM3**	0.59	1.51	1.29E-02	3.25E-02
**HIST1H2BK**	0.53	1.45	1.29E-02	3.25E-02
**HABP4**	-0.49	0.71	1.29E-02	3.25E-02
**BMP7**	1.05	2.08	1.29E-02	3.25E-02
**NUP37**	-0.24	0.84	1.30E-02	3.25E-02
**ZNF778**	-0.33	0.79	1.30E-02	3.25E-02
**POU2F3**	0.54	1.45	1.30E-02	3.26E-02
**HEY1**	-1.03	0.49	1.30E-02	3.27E-02
**CPT1C**	0.84	1.79	1.30E-02	3.27E-02
**TMEM204**	-1.05	0.48	1.30E-02	3.27E-02
**NAPB**	0.46	1.37	1.31E-02	3.29E-02
**RP11-526F3.1**	0.71	1.63	1.31E-02	3.29E-02
**TRIM14**	-0.17	0.89	1.31E-02	3.30E-02
**AC009005.2**	-1.05	0.48	1.32E-02	3.30E-02
**POM121C**	0.24	1.18	1.32E-02	3.30E-02
**MTPN**	0.15	1.11	1.32E-02	3.31E-02
**LINC00202-1**	0.66	1.58	1.32E-02	3.31E-02
**RP11-680F8.3**	1.02	2.03	1.32E-02	3.32E-02
**GPR107**	0.16	1.12	1.33E-02	3.33E-02
**ACAP3**	0.22	1.17	1.33E-02	3.33E-02
**PRSS22**	0.35	1.28	1.33E-02	3.33E-02
**PABPC1L**	0.40	1.32	1.33E-02	3.33E-02
**CYYR1-AS1**	-1.05	0.48	1.33E-02	3.33E-02
**NME6**	-0.29	0.82	1.33E-02	3.33E-02
**IL21R**	1.01	2.02	1.33E-02	3.34E-02
**RP11-304L19.13**	1.05	2.08	1.33E-02	3.34E-02
**RUFY2**	0.27	1.21	1.34E-02	3.34E-02
**DDAH1**	0.48	1.40	1.34E-02	3.35E-02
**AC016747.3**	-0.43	0.74	1.34E-02	3.35E-02
**REC8**	0.72	1.64	1.34E-02	3.36E-02
**RP11-596C23.6**	0.94	1.91	1.34E-02	3.36E-02
**XPOTP1**	-1.03	0.49	1.34E-02	3.36E-02
**CMTR2**	-0.22	0.86	1.35E-02	3.37E-02
**MRGPRX3**	-0.94	0.52	1.35E-02	3.38E-02
**PMS2**	-0.32	0.80	1.35E-02	3.38E-02
**DCAF12**	-0.18	0.88	1.35E-02	3.39E-02
**ATP1A1-AS1**	0.87	1.83	1.35E-02	3.39E-02
**SLC25A10**	-0.48	0.72	1.36E-02	3.39E-02
**S100A7**	-0.61	0.65	1.36E-02	3.40E-02
**ZSCAN21**	0.58	1.50	1.36E-02	3.40E-02
**TCEAL1**	0.39	1.31	1.36E-02	3.40E-02
**IKBKE**	0.33	1.26	1.36E-02	3.40E-02
**ERGIC3**	0.16	1.12	1.36E-02	3.41E-02
**SPATA2L**	-0.47	0.72	1.36E-02	3.41E-02
**ERCC8**	0.37	1.30	1.36E-02	3.41E-02
**RP11-267J23.4**	-1.02	0.49	1.37E-02	3.41E-02
**OSBPL9**	0.18	1.14	1.37E-02	3.41E-02
**NTSR1**	0.39	1.31	1.37E-02	3.41E-02
**DACH2**	0.95	1.94	1.37E-02	3.41E-02
**ZBTB11-AS1**	0.73	1.66	1.37E-02	3.41E-02
**SMPD4**	-0.19	0.88	1.37E-02	3.42E-02
**AGFG2**	-0.32	0.80	1.37E-02	3.42E-02
**PAK6**	0.21	1.16	1.37E-02	3.42E-02
**RNF128**	0.60	1.51	1.37E-02	3.43E-02
**ANKRD16**	-0.46	0.73	1.38E-02	3.43E-02
**MCF2L**	1.03	2.05	1.38E-02	3.44E-02
**SEL1L**	-0.21	0.86	1.38E-02	3.45E-02
**CSE1L**	-0.15	0.90	1.39E-02	3.46E-02
**RP11-705O1.8**	1.00	2.00	1.39E-02	3.46E-02
**TMBIM6**	-0.12	0.92	1.39E-02	3.47E-02
**KIAA1429**	0.18	1.14	1.39E-02	3.47E-02
**TSNAXIP1**	0.98	1.98	1.39E-02	3.47E-02
**HLTF**	-0.28	0.83	1.39E-02	3.47E-02
**NDRG3**	0.25	1.19	1.40E-02	3.48E-02
**FBXO34**	0.25	1.19	1.40E-02	3.48E-02
**ZNF497**	0.97	1.96	1.40E-02	3.48E-02
**ING2**	-0.31	0.81	1.40E-02	3.48E-02
**HN1L**	-0.14	0.91	1.40E-02	3.49E-02
**RP1-317E23.3**	1.05	2.07	1.40E-02	3.49E-02
**PTPN9**	-0.20	0.87	1.40E-02	3.49E-02
**ZDHHC23**	-0.56	0.68	1.41E-02	3.50E-02
**CTC-526N19.1**	0.77	1.71	1.41E-02	3.51E-02
**NPTX1**	1.02	2.03	1.41E-02	3.52E-02

ZZZ3	-0.23	0.85	1.42E-02	3.52E-02
RAB10	-0.16	0.90	1.42E-02	3.52E-02
SNHG10	-0.84	0.56	1.43E-02	3.54E-02
TTF1	-0.29	0.82	1.43E-02	3.55E-02
OXR1	-0.24	0.85	1.43E-02	3.55E-02
CTB-119C2.1	1.04	2.06	1.43E-02	3.55E-02
COL13A1	-0.77	0.59	1.43E-02	3.56E-02
NCKIPSD	-0.26	0.83	1.43E-02	3.56E-02
TRIM6	-0.41	0.75	1.43E-02	3.56E-02
GPR68	-0.53	0.69	1.43E-02	3.56E-02
ARL8B	0.20	1.15	1.43E-02	3.56E-02
IRAK4	0.27	1.20	1.44E-02	3.57E-02
HSD3B7	-0.66	0.63	1.44E-02	3.57E-02
CDK16	-0.16	0.89	1.44E-02	3.57E-02
DCUN1D2	-0.47	0.72	1.44E-02	3.59E-02
IL15RA	-0.62	0.65	1.45E-02	3.60E-02
DCUN1D1	0.28	1.21	1.45E-02	3.60E-02
DNAL1	0.39	1.31	1.46E-02	3.61E-02
ZFHX4	-0.86	0.55	1.46E-02	3.61E-02
WASH2P	0.74	1.67	1.46E-02	3.61E-02
RP11-613M10.6	0.63	1.55	1.46E-02	3.61E-02
ZMPSTE24	0.19	1.14	1.46E-02	3.62E-02
THAP5	-0.34	0.79	1.46E-02	3.62E-02
NCK2	-0.22	0.86	1.46E-02	3.63E-02
SLC52A2	-0.22	0.86	1.46E-02	3.63E-02
POT1	-0.28	0.83	1.46E-02	3.63E-02
MAP7D2	-0.52	0.70	1.47E-02	3.64E-02
GRK5	0.35	1.27	1.47E-02	3.64E-02
DRAP1	0.16	1.11	1.47E-02	3.64E-02
BCORL1	0.32	1.25	1.48E-02	3.66E-02
RP11-834C11.4	-0.50	0.71	1.48E-02	3.67E-02
FAM172A	-0.35	0.79	1.48E-02	3.67E-02
SNCB	-1.04	0.49	1.48E-02	3.68E-02
PMP22	-0.99	0.50	1.48E-02	3.68E-02
NUP62CL	0.56	1.48	1.49E-02	3.68E-02
PPFIA1	-0.18	0.88	1.49E-02	3.68E-02
TMPRSS11E	-0.29	0.82	1.49E-02	3.69E-02
EFCAB10	0.99	1.98	1.50E-02	3.70E-02
KCNJ2	1.03	2.04	1.50E-02	3.71E-02
SACM1L	-0.25	0.84	1.50E-02	3.71E-02
IPO9	-0.16	0.89	1.50E-02	3.71E-02
DACT3	1.04	2.05	1.50E-02	3.72E-02
SLC30A4	0.44	1.35	1.50E-02	3.72E-02
THYN1	-0.23	0.85	1.50E-02	3.72E-02
LTBR	-0.16	0.89	1.51E-02	3.72E-02
FADD	-0.48	0.71	1.51E-02	3.72E-02
RTFDC1	0.21	1.16	1.51E-02	3.72E-02
EXOC8	0.30	1.23	1.51E-02	3.73E-02
ATP6V1C1	0.21	1.16	1.51E-02	3.73E-02
ASAH1	0.17	1.13	1.51E-02	3.74E-02
DAZAP2	-0.15	0.90	1.52E-02	3.76E-02
EMC4	0.17	1.12	1.52E-02	3.76E-02
NUDCD1	-0.22	0.86	1.52E-02	3.76E-02
C9orf66	1.02	2.03	1.53E-02	3.77E-02
EGFL7	0.38	1.30	1.53E-02	3.78E-02
ZNF559	-0.82	0.56	1.53E-02	3.78E-02
PSMA6	-0.44	0.74	1.53E-02	3.78E-02
RP11-378J18.8	-1.02	0.49	1.53E-02	3.78E-02
GPHN	0.22	1.16	1.54E-02	3.79E-02
CBWD2	0.42	1.34	1.54E-02	3.81E-02
RBSN	-0.22	0.86	1.55E-02	3.81E-02
ATP2B1	-0.21	0.86	1.55E-02	3.82E-02
MUL1	0.22	1.16	1.55E-02	3.83E-02
NR2C2	-0.24	0.85	1.55E-02	3.83E-02
LINC00941	-0.58	0.67	1.55E-02	3.83E-02
FBXO11	-0.22	0.86	1.56E-02	3.84E-02
ZNF675	-0.41	0.75	1.56E-02	3.84E-02
CEP68	0.30	1.23	1.56E-02	3.85E-02
PDP1	0.21	1.16	1.57E-02	3.86E-02
AACS	-0.21	0.87	1.57E-02	3.87E-02
ZNHIT1	0.19	1.14	1.57E-02	3.87E-02
RCN2	-0.22	0.86	1.57E-02	3.87E-02
SNAPC4	-0.26	0.83	1.57E-02	3.88E-02
ABCA13	0.92	1.89	1.57E-02	3.88E-02
STX16-NPEPL1	1.00	1.99	1.58E-02	3.89E-02
MLEC	0.15	1.11	1.58E-02	3.89E-02
TACC1	-0.20	0.87	1.58E-02	3.89E-02
CAP1	-0.12	0.92	1.58E-02	3.90E-02
TUBG1	0.16	1.12	1.58E-02	3.90E-02
HSDL1	0.29	1.22	1.59E-02	3.91E-02
KRT6A	-0.11	0.93	1.59E-02	3.92E-02
TMEM8A	0.25	1.19	1.60E-02	3.93E-02
HNRNPM	-0.15	0.90	1.60E-02	3.93E-02
CTA-29F11.1	-0.80	0.58	1.60E-02	3.93E-02
ELOVL2	0.92	1.90	1.60E-02	3.93E-02
CHAD	1.03	2.04	1.60E-02	3.94E-02
SLC25A38	0.26	1.20	1.61E-02	3.94E-02
MOXD1	0.80	1.75	1.61E-02	3.95E-02
ITLN2	1.01	2.01	1.61E-02	3.95E-02
RBMX2	-0.27	0.83	1.61E-02	3.95E-02

**GNE**	0.30	1.23	1.61E-02	3.95E-02
**METRN**	-0.31	0.80	1.61E-02	3.95E-02
**FAM27A**	0.82	1.77	1.61E-02	3.95E-02
**VPS13D**	-0.21	0.86	1.61E-02	3.95E-02
**SLC30A5**	-0.25	0.84	1.61E-02	3.95E-02
**TADA3**	-0.19	0.87	1.61E-02	3.96E-02
**PAPOLG**	0.28	1.21	1.61E-02	3.96E-02
**TPM1**	0.18	1.13	1.61E-02	3.96E-02
**FAM212A**	-0.35	0.78	1.61E-02	3.96E-02
**DDX41**	0.17	1.12	1.62E-02	3.97E-02
**TESK1**	0.27	1.20	1.62E-02	3.97E-02
**RPS3A**	0.15	1.11	1.62E-02	3.98E-02
**CEP152**	-0.35	0.78	1.62E-02	3.98E-02
**UPF3B**	0.33	1.25	1.62E-02	3.98E-02
**GCFC2**	-0.34	0.79	1.63E-02	3.99E-02
**LIPT1**	-0.73	0.60	1.63E-02	3.99E-02
**ATP5G3**	0.14	1.10	1.63E-02	3.99E-02
**ABL1**	-0.18	0.88	1.63E-02	3.99E-02
**UPK1B**	-0.15	0.90	1.63E-02	3.99E-02
**LINC01311**	-0.99	0.50	1.63E-02	4.00E-02
**TMEM216**	0.38	1.30	1.63E-02	4.00E-02
**TTC22**	-0.27	0.83	1.63E-02	4.00E-02
**RAB11FIP2**	0.30	1.24	1.64E-02	4.00E-02
**NUFIP2**	-0.17	0.89	1.64E-02	4.01E-02
**INHBA**	0.39	1.31	1.64E-02	4.01E-02
**NRBP2**	0.22	1.17	1.64E-02	4.02E-02
**CXCL3**	-0.82	0.56	1.64E-02	4.02E-02
**RP11-71E19.1**	0.97	1.96	1.65E-02	4.03E-02
**SMIM22**	-0.63	0.64	1.65E-02	4.03E-02
**XPNPEP3**	0.27	1.21	1.65E-02	4.03E-02
**C4orf36**	-0.54	0.69	1.65E-02	4.03E-02
**RP11-176H8.1**	0.93	1.90	1.65E-02	4.03E-02
**GNG7**	1.00	2.01	1.65E-02	4.04E-02
**KLHL30**	1.02	2.03	1.65E-02	4.04E-02
**GALR2**	0.92	1.90	1.66E-02	4.05E-02
**C2orf42**	0.38	1.30	1.66E-02	4.06E-02
**POLR1C**	-0.27	0.83	1.66E-02	4.06E-02
**PPIA**	0.18	1.13	1.66E-02	4.06E-02
**CCDC167**	-0.36	0.78	1.66E-02	4.06E-02
**CSTF2T**	-0.24	0.85	1.66E-02	4.06E-02
**LIN54**	-0.29	0.82	1.67E-02	4.07E-02
**NDUFA11**	0.31	1.24	1.67E-02	4.07E-02
**TAF5**	-0.35	0.79	1.67E-02	4.08E-02
**WDPCP**	-0.67	0.63	1.67E-02	4.08E-02
**STAT5A**	-0.72	0.61	1.67E-02	4.09E-02
**AC010127.3**	-1.01	0.50	1.67E-02	4.09E-02
**SSSCA1**	-0.27	0.83	1.67E-02	4.09E-02
**PLEKHA6**	0.36	1.29	1.68E-02	4.10E-02
**NUDT15**	-0.20	0.87	1.68E-02	4.10E-02
**HOTAIRM1**	0.48	1.39	1.68E-02	4.10E-02
**SDR39U1**	0.65	1.57	1.68E-02	4.10E-02
**PRKCSH**	-0.17	0.89	1.69E-02	4.11E-02
**SDCBP2**	0.21	1.15	1.69E-02	4.11E-02
**AC115522.3**	-1.02	0.49	1.69E-02	4.11E-02
**ZNF143**	0.27	1.21	1.69E-02	4.12E-02
**EIF2A**	-0.16	0.89	1.69E-02	4.13E-02
**CCDC82**	0.33	1.26	1.69E-02	4.13E-02
**DIS3L2**	0.23	1.17	1.70E-02	4.14E-02
**ATP11B**	-0.23	0.85	1.70E-02	4.14E-02
**C6orf203**	0.32	1.24	1.70E-02	4.15E-02
**RAB20**	-0.55	0.68	1.71E-02	4.15E-02
**PIR**	-0.42	0.75	1.72E-02	4.19E-02
**EFHC1**	0.41	1.33	1.72E-02	4.20E-02
**SFI1**	-0.31	0.81	1.73E-02	4.20E-02
**PIGH**	0.36	1.28	1.73E-02	4.20E-02
**ALKBH7**	-0.34	0.79	1.73E-02	4.20E-02
**CHIC2**	0.38	1.30	1.73E-02	4.21E-02
**ATG16L2**	0.47	1.39	1.73E-02	4.21E-02
**ACOT1**	0.93	1.90	1.73E-02	4.21E-02
**42988**	-0.19	0.87	1.73E-02	4.21E-02
**EFTUD1**	-0.21	0.86	1.73E-02	4.21E-02
**C6orf226**	0.68	1.61	1.73E-02	4.21E-02
**ZC3H3**	-0.25	0.84	1.73E-02	4.22E-02
**NMI**	-0.52	0.70	1.74E-02	4.22E-02
**GNA15**	-0.18	0.88	1.74E-02	4.22E-02
**STAT4**	0.92	1.89	1.74E-02	4.22E-02
**CIRBP-AS1**	0.88	1.84	1.74E-02	4.22E-02
**BBS5**	-0.47	0.72	1.74E-02	4.23E-02
**TRAPPC3**	-0.17	0.89	1.74E-02	4.23E-02
**IER5L**	0.27	1.21	1.74E-02	4.24E-02
**MLXIPL**	0.98	1.97	1.74E-02	4.24E-02
**PXDN**	0.14	1.10	1.75E-02	4.24E-02
**CNOT10**	-0.24	0.85	1.75E-02	4.24E-02
**CUTA**	0.19	1.14	1.75E-02	4.25E-02
**MRPL38**	0.83	1.78	1.75E-02	4.25E-02
**FBXO30**	-0.33	0.80	1.75E-02	4.26E-02
**MAPKAPK2**	-0.17	0.89	1.75E-02	4.26E-02
**TMEM33**	-0.17	0.89	1.76E-02	4.26E-02
**RILPL1**	0.30	1.23	1.76E-02	4.27E-02
**NRDE2**	-0.33	0.80	1.76E-02	4.27E-02

**PLOD1**	0.15	1.11	1.76E-02	4.27E-02
**PEX11G**	0.71	1.64	1.77E-02	4.29E-02
**RP11-449J21.3**	0.71	1.63	1.77E-02	4.30E-02
**SEC24C**	0.17	1.12	1.78E-02	4.31E-02
**EEF1E1**	-0.42	0.75	1.78E-02	4.32E-02
**LRRN4**	-0.93	0.53	1.78E-02	4.32E-02
**AKAP6**	-0.63	0.65	1.78E-02	4.32E-02
**ATP10B**	-0.54	0.69	1.79E-02	4.33E-02
**AC017006.3**	0.96	1.95	1.79E-02	4.33E-02
**PSEN2**	-0.37	0.77	1.79E-02	4.34E-02
**RP11-361L15.4**	0.96	1.95	1.79E-02	4.34E-02
**WDR60**	-0.33	0.79	1.79E-02	4.34E-02
**DOCK10**	0.84	1.79	1.79E-02	4.34E-02
**MRPL48**	0.26	1.20	1.79E-02	4.34E-02
**AC006116.20**	0.85	1.80	1.80E-02	4.35E-02
**RPL13P12**	-0.42	0.75	1.80E-02	4.35E-02
**RP11-267D19.2**	0.90	1.87	1.80E-02	4.35E-02
**PIM1**	-0.19	0.88	1.80E-02	4.35E-02
**SLC16A7**	-0.77	0.58	1.80E-02	4.35E-02
**PSME3**	0.13	1.09	1.80E-02	4.36E-02
**FOXD2**	-0.99	0.50	1.80E-02	4.36E-02
**EAPP**	0.25	1.19	1.81E-02	4.37E-02
**TRABD2A**	-0.86	0.55	1.81E-02	4.38E-02
**FBXO43**	-1.01	0.50	1.81E-02	4.38E-02
**RP11-30K9.6**	1.00	2.00	1.81E-02	4.38E-02
**TRMT61B**	0.32	1.25	1.81E-02	4.38E-02
**CAMSAP3**	-0.19	0.87	1.81E-02	4.38E-02
**P2RX5-TAX1BP3**	-0.99	0.50	1.82E-02	4.39E-02
**FCHO1**	-0.56	0.68	1.82E-02	4.40E-02
**SH3BP5**	0.57	1.49	1.83E-02	4.41E-02
**AZGP1**	-0.75	0.59	1.83E-02	4.41E-02
**ZFP42**	-0.54	0.69	1.83E-02	4.42E-02
**TSPAN15**	-0.53	0.69	1.83E-02	4.42E-02
**RPP40**	-0.34	0.79	1.83E-02	4.42E-02
**KIAA0100**	-0.15	0.90	1.84E-02	4.44E-02
**SLC22A17**	0.81	1.75	1.84E-02	4.44E-02
**TMEM160**	-0.45	0.73	1.84E-02	4.44E-02
**ASAP1**	0.16	1.12	1.84E-02	4.44E-02
**SATB2**	0.81	1.76	1.84E-02	4.45E-02
**RPL7P9**	-0.43	0.74	1.84E-02	4.45E-02
**DENND5A**	0.22	1.17	1.84E-02	4.45E-02
**C20orf197**	-0.66	0.63	1.84E-02	4.45E-02
**CD55**	0.23	1.17	1.85E-02	4.45E-02
**C16orf70**	0.24	1.18	1.85E-02	4.45E-02
**NANP**	-0.31	0.80	1.85E-02	4.46E-02
**PLS3-AS1**	0.96	1.94	1.85E-02	4.47E-02
**MCAT**	-0.28	0.82	1.86E-02	4.48E-02
**MRPL35**	-0.20	0.87	1.86E-02	4.49E-02
**RP11-549B18.1**	0.93	1.91	1.86E-02	4.49E-02
**CDK8**	-0.23	0.85	1.87E-02	4.50E-02
**GNPDA2**	0.30	1.23	1.87E-02	4.51E-02
**RHPN1**	0.40	1.32	1.87E-02	4.51E-02
**SDAD1**	-0.21	0.86	1.87E-02	4.51E-02
**SMARCD3**	0.70	1.62	1.87E-02	4.51E-02
**SDR42E1**	0.24	1.18	1.87E-02	4.51E-02
**TMEM198**	0.80	1.74	1.88E-02	4.51E-02
**IDS**	0.20	1.14	1.88E-02	4.52E-02
**BLOC1S4**	0.36	1.28	1.88E-02	4.53E-02
**RUVBL2**	-0.17	0.89	1.88E-02	4.53E-02
**ERI3**	-0.21	0.87	1.89E-02	4.55E-02
**ZNF16**	-0.50	0.71	1.89E-02	4.55E-02
**ARMC7**	-0.39	0.76	1.89E-02	4.55E-02
**NDUFC2**	0.31	1.24	1.89E-02	4.55E-02
**MPV17L**	-0.74	0.60	1.89E-02	4.55E-02
**CCDC43**	0.26	1.20	1.90E-02	4.56E-02
**CYYR1**	0.63	1.55	1.90E-02	4.56E-02
**DNTTIP1**	-0.24	0.85	1.90E-02	4.57E-02
**C9orf85**	0.34	1.26	1.90E-02	4.57E-02
**SMO**	-0.30	0.81	1.91E-02	4.58E-02
**LAP3**	0.17	1.12	1.92E-02	4.60E-02
**RNF6**	0.19	1.14	1.92E-02	4.61E-02
**IRF2BP2**	-0.17	0.89	1.92E-02	4.62E-02
**BCL2L13**	0.16	1.12	1.93E-02	4.64E-02
**EDAR**	0.69	1.61	1.93E-02	4.64E-02
**RNF181**	0.19	1.14	1.94E-02	4.65E-02
**CHST8**	0.99	1.98	1.94E-02	4.66E-02
**FBXL22**	-0.98	0.51	1.94E-02	4.66E-02
**RP11-399O19.9**	0.98	1.98	1.94E-02	4.66E-02
**HAT1**	-0.20	0.87	1.94E-02	4.67E-02
**LEF1**	-0.84	0.56	1.95E-02	4.67E-02
**CTA-228A9.3**	0.99	1.99	1.95E-02	4.67E-02
**CHRNA5**	-0.63	0.65	1.95E-02	4.67E-02
**KRT18P3**	0.99	1.98	1.95E-02	4.68E-02
**HNRNPU-AS1**	-0.99	0.50	1.95E-02	4.68E-02
**SIRT4**	0.99	1.98	1.95E-02	4.68E-02
**CECR1**	0.66	1.58	1.96E-02	4.69E-02
**KRT33A**	0.99	1.99	1.96E-02	4.69E-02
**H2AFY**	-0.13	0.92	1.96E-02	4.69E-02
**SEMA4F**	-0.31	0.81	1.96E-02	4.69E-02
**TTC39A**	-0.36	0.78	1.96E-02	4.69E-02

**MAPKAPK5**	-0.22	0.86	1.96E-02	4.70E-02
**FNIP2**	-0.27	0.83	1.96E-02	4.71E-02
**AC006128.2**	0.84	1.79	1.97E-02	4.71E-02
**PQBP1**	-0.20	0.87	1.97E-02	4.71E-02
**HILPDA**	0.32	1.24	1.97E-02	4.72E-02
**RBM43**	0.48	1.39	1.98E-02	4.73E-02
**CHIC1**	0.52	1.43	1.98E-02	4.73E-02
**MAPKAP1**	-0.14	0.91	1.98E-02	4.73E-02
**APBA3**	0.34	1.26	1.98E-02	4.74E-02
**CRYZL1**	-0.33	0.79	1.98E-02	4.74E-02
**C6orf120**	0.25	1.19	1.98E-02	4.75E-02
**ZNF365**	0.29	1.22	1.98E-02	4.75E-02
**DGCR2**	-0.16	0.89	1.99E-02	4.76E-02
**PTPN13**	-0.14	0.91	1.99E-02	4.77E-02
**SLC1A3**	0.23	1.18	2.00E-02	4.78E-02
**SS18L2**	-0.40	0.76	2.00E-02	4.78E-02
**HCG18**	-0.28	0.82	2.00E-02	4.79E-02
**CCDC66**	-0.33	0.80	2.00E-02	4.79E-02
**TAF11**	0.28	1.22	2.00E-02	4.79E-02
**PIK3C3**	0.22	1.16	2.01E-02	4.79E-02
**CTC-444N24.8**	-0.95	0.52	2.01E-02	4.80E-02
**CPM**	-0.29	0.82	2.01E-02	4.81E-02
**TMEM39B**	-0.35	0.78	2.01E-02	4.81E-02
**KLHDC1**	0.91	1.88	2.02E-02	4.82E-02
**LRRFIP2**	-0.16	0.90	2.02E-02	4.83E-02
**COL6A1**	0.70	1.62	2.02E-02	4.83E-02
**HIST1H2AG**	0.78	1.72	2.02E-02	4.83E-02
**SCARF2**	0.96	1.95	2.03E-02	4.84E-02
**UVSSA**	-0.33	0.79	2.03E-02	4.84E-02
**ZNF133**	0.31	1.24	2.03E-02	4.84E-02
**ISOC2**	-0.27	0.83	2.03E-02	4.85E-02
**SLC12A6**	0.19	1.14	2.03E-02	4.85E-02
**BMS1P10**	0.97	1.95	2.04E-02	4.86E-02
**PTPN21**	-0.27	0.83	2.04E-02	4.86E-02
**MYDGF**	-0.18	0.88	2.04E-02	4.86E-02
**ERLEC1**	0.22	1.16	2.04E-02	4.87E-02
**CHDH**	-0.98	0.51	2.04E-02	4.87E-02
**PDCD4-AS1**	0.95	1.93	2.04E-02	4.87E-02
**VGLL4**	0.21	1.16	2.05E-02	4.88E-02
**C17orf89**	0.24	1.18	2.05E-02	4.88E-02
**RP11-338I21.1**	0.98	1.97	2.05E-02	4.88E-02
**THOC1**	-0.25	0.84	2.05E-02	4.89E-02
**SEC22C**	-0.19	0.88	2.05E-02	4.89E-02
**SLC28A3**	0.61	1.53	2.06E-02	4.91E-02
**THOC6**	-0.28	0.82	2.06E-02	4.91E-02
**RBFOX2**	-0.15	0.90	2.06E-02	4.91E-02
**MICE**	-0.87	0.55	2.06E-02	4.91E-02
**EXOC4**	-0.17	0.89	2.06E-02	4.92E-02
**UBE2D2**	-0.15	0.90	2.07E-02	4.92E-02
**MFSD9**	0.29	1.22	2.07E-02	4.93E-02
**ELOF1**	0.19	1.14	2.07E-02	4.94E-02
**TM4SF1-AS1**	0.99	1.98	2.07E-02	4.94E-02
**LCE3D**	-0.89	0.54	2.08E-02	4.95E-02
**CES1**	0.87	1.83	2.08E-02	4.95E-02
**LINC01569**	0.94	1.91	2.08E-02	4.95E-02
**MOCS3**	0.28	1.22	2.08E-02	4.95E-02
**UCP2**	0.52	1.43	2.08E-02	4.95E-02
**COG2**	0.22	1.17	2.08E-02	4.95E-02
**ZDHHC5**	0.14	1.10	2.08E-02	4.95E-02
**TWISTNB**	-0.22	0.86	2.08E-02	4.95E-02
**CCT7**	0.12	1.08	2.09E-02	4.97E-02
**CABIN1**	-0.18	0.88	2.09E-02	4.97E-02
**RP11-524H19.2**	0.96	1.95	2.09E-02	4.97E-02
**CTC-497E21.3**	0.98	1.98	2.09E-02	4.97E-02
**TSPAN2**	0.98	1.97	2.09E-02	4.97E-02
**RP11-230F18.5**	-0.96	0.52	2.09E-02	4.97E-02
**VAMP5**	0.65	1.57	2.09E-02	4.97E-02
**PRMT3**	-0.21	0.86	2.09E-02	4.97E-02
**ZNF710**	-0.20	0.87	2.10E-02	4.99E-02
**FAM32A**	0.16	1.12	2.10E-02	4.99E-02
**RP11-390P2.4**	0.70	1.62	2.10E-02	5.00E-02
**NDUFA2**	0.30	1.23	2.11E-02	5.00E-02
**CARNS1**	0.98	1.97	2.11E-02	5.00E-02
**IL17RE**	-0.24	0.85	2.11E-02	5.00E-02
**SLMAP**	-0.16	0.90	2.11E-02	5.00E-02
**TP53BP2**	0.19	1.14	2.11E-02	5.00E-02
**C12orf76**	0.62	1.54	2.11E-02	5.00E-02
**RP1-313I6.12**	-0.86	0.55	2.11E-02	5.01E-02
**MECR**	0.23	1.18	2.11E-02	5.01E-02
**MYCL**	-0.30	0.81	2.11E-02	5.01E-02
**HPS3**	0.28	1.21	2.11E-02	5.01E-02
**TTBK2**	-0.34	0.79	2.11E-02	5.01E-02
**FOXA1**	0.67	1.59	2.13E-02	5.04E-02
**ZMYND12**	0.97	1.96	2.13E-02	5.04E-02
**NR2C1**	0.27	1.20	2.13E-02	5.04E-02
**MRPL2**	-0.21	0.86	2.13E-02	5.04E-02
**CTD-2314G24.2**	-0.83	0.56	2.13E-02	5.05E-02
**SMIM15**	-0.21	0.86	2.13E-02	5.05E-02
**TADA2A**	-0.28	0.82	2.13E-02	5.05E-02
**NUMA1**	-0.15	0.90	2.14E-02	5.06E-02

ACTR5	-0.34	0.79	2.14E-02	5.06E-02
ALAD	0.24	1.18	2.14E-02	5.06E-02
C7orf26	-0.28	0.82	2.14E-02	5.06E-02
CTA-390C10.10	0.86	1.82	2.14E-02	5.07E-02
LY6E	-0.15	0.90	2.14E-02	5.07E-02
RP11-94H18.1	0.79	1.73	2.14E-02	5.07E-02
ORC5	-0.23	0.85	2.15E-02	5.07E-02
RPS4XP19	0.77	1.71	2.15E-02	5.08E-02
NPIPP1	-0.66	0.63	2.15E-02	5.09E-02
AC046143.3	-0.83	0.56	2.16E-02	5.10E-02
WDR34	-0.20	0.87	2.16E-02	5.11E-02
GNA13	0.20	1.15	2.16E-02	5.11E-02
AIFM3	0.90	1.87	2.16E-02	5.11E-02
GLRA4	0.97	1.96	2.17E-02	5.13E-02
P2RX6	0.97	1.96	2.17E-02	5.13E-02
MAPKAPK5-AS1	-0.60	0.66	2.17E-02	5.13E-02
CADM4	0.23	1.17	2.18E-02	5.15E-02
CCDC183-AS1	0.82	1.76	2.18E-02	5.15E-02
RP3-402G11.28	0.97	1.96	2.19E-02	5.17E-02
TLR5	0.45	1.36	2.20E-02	5.18E-02
LYRM7	-0.28	0.82	2.20E-02	5.20E-02
LINC00973	0.95	1.93	2.20E-02	5.20E-02
FAXC	-0.80	0.57	2.20E-02	5.20E-02
RPS4XP16	0.93	1.90	2.20E-02	5.20E-02
TMCO4	0.27	1.20	2.21E-02	5.21E-02
RP1-150O5.3	0.92	1.89	2.21E-02	5.21E-02
FCMR	0.78	1.72	2.21E-02	5.21E-02
PIK3IP1	0.47	1.39	2.21E-02	5.22E-02
NXN	-0.16	0.90	2.22E-02	5.23E-02
SKIL	0.27	1.21	2.22E-02	5.23E-02
MCM9	-0.32	0.80	2.22E-02	5.24E-02
EPC1	-0.29	0.82	2.22E-02	5.24E-02
ADAT3	0.56	1.47	2.23E-02	5.25E-02
SVBP	-0.40	0.76	2.23E-02	5.25E-02
RP5-1099D15.1	-0.97	0.51	2.23E-02	5.25E-02
CNOT6L	0.24	1.18	2.23E-02	5.25E-02
LAT2	-0.76	0.59	2.23E-02	5.25E-02
NALCN	0.77	1.70	2.23E-02	5.25E-02
AL662800.1	0.86	1.82	2.23E-02	5.26E-02
RP11-554I8.2	0.31	1.24	2.24E-02	5.26E-02
POLR2I	-0.42	0.75	2.24E-02	5.27E-02
SH2D3A	0.21	1.15	2.24E-02	5.27E-02
LINC00592	0.93	1.90	2.24E-02	5.27E-02
C2orf49	0.27	1.20	2.24E-02	5.28E-02
SPOP	0.22	1.17	2.24E-02	5.28E-02
CTD-2366F13.1	0.50	1.41	2.24E-02	5.28E-02
HOXC11	-0.40	0.76	2.24E-02	5.28E-02
SEMA4C	-0.35	0.79	2.25E-02	5.28E-02
HIST1H2BL	-0.95	0.52	2.25E-02	5.29E-02
B3GLCT	0.34	1.27	2.25E-02	5.29E-02
VWA9	-0.20	0.87	2.25E-02	5.29E-02
GPR39	-0.56	0.68	2.26E-02	5.32E-02
RING1	-0.23	0.85	2.27E-02	5.33E-02
TIMM8B	0.23	1.17	2.27E-02	5.33E-02
GNG12	0.14	1.10	2.27E-02	5.33E-02
FKBP4	-0.13	0.92	2.27E-02	5.33E-02
DNAJC24	0.34	1.27	2.27E-02	5.33E-02
CENPW	-0.28	0.82	2.27E-02	5.34E-02
NPHP1	0.48	1.40	2.28E-02	5.36E-02
TMEM56	0.86	1.81	2.29E-02	5.37E-02
RAB28	0.30	1.23	2.29E-02	5.37E-02
PNPLA7	0.95	1.93	2.29E-02	5.37E-02
RP5-862P8.2	-0.27	0.83	2.29E-02	5.38E-02
PIN4	0.31	1.24	2.29E-02	5.38E-02
RP11-39C10.1	0.93	1.91	2.29E-02	5.38E-02
APBB2	0.18	1.13	2.30E-02	5.39E-02
SNHG3	-0.26	0.83	2.30E-02	5.39E-02
LMBRD2	0.27	1.20	2.30E-02	5.39E-02
IL12RB2	-0.56	0.68	2.30E-02	5.39E-02
RRAGB	0.55	1.46	2.31E-02	5.41E-02
ZNF114	0.74	1.67	2.31E-02	5.41E-02
PANK3	-0.18	0.89	2.31E-02	5.42E-02
RP3-395M20.8	0.96	1.95	2.31E-02	5.43E-02
SLC27A6	-0.72	0.61	2.31E-02	5.43E-02
GAS6	-0.17	0.89	2.32E-02	5.43E-02
OTUD5	0.18	1.13	2.32E-02	5.43E-02
C9orf69	-0.19	0.88	2.32E-02	5.44E-02
PRIM2	-0.29	0.82	2.32E-02	5.44E-02
ADH6	0.91	1.88	2.32E-02	5.44E-02
ANAPC13	0.21	1.16	2.33E-02	5.45E-02
CTD-2292M16.8	-0.97	0.51	2.33E-02	5.47E-02
RAB27A	-0.30	0.81	2.34E-02	5.47E-02
ANKRD11	-0.14	0.91	2.34E-02	5.48E-02
SNRPG	-0.28	0.82	2.34E-02	5.49E-02
SLC20A1	-0.14	0.91	2.35E-02	5.50E-02
MORC3	0.21	1.16	2.35E-02	5.50E-02
NDUFB8	0.30	1.23	2.35E-02	5.50E-02
WNT2B	-0.60	0.66	2.35E-02	5.50E-02
PLD6	-0.79	0.58	2.35E-02	5.50E-02
PLS1	0.21	1.16	2.36E-02	5.51E-02

**POLE4**	0.25	1.19	2.36E-02	5.51E-02
**COQ10A**	0.54	1.45	2.36E-02	5.51E-02
**PIGS**	-0.17	0.89	2.36E-02	5.52E-02
**TNS4**	0.16	1.12	2.36E-02	5.52E-02
**IPPK**	0.25	1.19	2.36E-02	5.53E-02
**ACBD5**	0.21	1.16	2.37E-02	5.54E-02
**QSOX2**	-0.24	0.85	2.38E-02	5.57E-02
**ALYREF**	-0.17	0.89	2.38E-02	5.57E-02
**NDOR1**	0.45	1.36	2.38E-02	5.57E-02
**SLC9A3**	0.76	1.69	2.38E-02	5.57E-02
**RP11-465N4.4**	0.96	1.95	2.39E-02	5.58E-02
**SKIV2L2**	-0.16	0.89	2.39E-02	5.58E-02
**TMEM136**	-0.79	0.58	2.39E-02	5.58E-02
**HNRNPH2**	-0.17	0.89	2.39E-02	5.59E-02
**ATXN2L**	-0.19	0.88	2.41E-02	5.62E-02
**CAMK2D**	0.20	1.15	2.41E-02	5.62E-02
**ANO2**	0.91	1.88	2.41E-02	5.63E-02
**CDKL5**	0.31	1.24	2.41E-02	5.63E-02
**ZMYM5**	0.33	1.26	2.42E-02	5.64E-02
**L1TD1**	-0.62	0.65	2.42E-02	5.64E-02
**MCM3AP-AS1**	-0.62	0.65	2.42E-02	5.64E-02
**RP11-1277A3.1**	0.71	1.64	2.42E-02	5.64E-02
**TTC23**	0.27	1.20	2.42E-02	5.65E-02
**SAMD10**	-0.35	0.78	2.43E-02	5.66E-02
**CTB-25B13.5**	-0.94	0.52	2.43E-02	5.68E-02
**SLC5A6**	-0.19	0.87	2.44E-02	5.68E-02
**SLC27A2**	-0.64	0.64	2.44E-02	5.68E-02
**ATXN1**	-0.25	0.84	2.44E-02	5.69E-02
**CHST6**	0.54	1.46	2.44E-02	5.69E-02
**TMEM47**	0.96	1.94	2.44E-02	5.70E-02
**POP4**	0.20	1.15	2.45E-02	5.72E-02
**CAMKMT**	-0.40	0.76	2.46E-02	5.73E-02
**SCD5**	0.18	1.13	2.46E-02	5.74E-02
**KIDINS220**	0.16	1.12	2.46E-02	5.74E-02
**TUBG2**	0.25	1.19	2.47E-02	5.74E-02
**FAM149A**	0.82	1.76	2.47E-02	5.74E-02
**ZNF205**	0.29	1.22	2.47E-02	5.74E-02
**MKL2**	-0.20	0.87	2.47E-02	5.74E-02
**PSMD5**	0.16	1.12	2.48E-02	5.76E-02
**SPINT2**	0.14	1.10	2.48E-02	5.77E-02
**SRPR**	-0.13	0.91	2.48E-02	5.77E-02
**YTHDF2**	-0.17	0.89	2.48E-02	5.77E-02
**ANK3**	-0.23	0.85	2.48E-02	5.78E-02
**RP11-244J10.1**	-0.95	0.52	2.49E-02	5.79E-02
**SUSD1**	-0.48	0.72	2.49E-02	5.80E-02
**ANO7P1**	0.50	1.41	2.49E-02	5.80E-02
**AF131215.9**	0.90	1.87	2.49E-02	5.80E-02
**MORN2**	0.38	1.30	2.49E-02	5.80E-02
**COQ6**	0.45	1.36	2.49E-02	5.80E-02
**CRAT**	0.50	1.42	2.50E-02	5.81E-02
**CCNT2**	-0.24	0.85	2.50E-02	5.81E-02
**SLC37A3**	0.22	1.16	2.50E-02	5.81E-02
**ANXA8L2**	-0.32	0.80	2.50E-02	5.82E-02
**CNPY4**	0.49	1.40	2.50E-02	5.82E-02
**RP11-95D17.1**	0.48	1.40	2.51E-02	5.82E-02
**WRB**	-0.26	0.84	2.51E-02	5.83E-02
**COX10**	-0.25	0.84	2.51E-02	5.83E-02
**INSL4**	0.95	1.94	2.51E-02	5.83E-02
**MTL5**	-0.36	0.78	2.51E-02	5.83E-02
**SH3RF3**	-0.90	0.53	2.51E-02	5.83E-02
**ERI1**	-0.25	0.84	2.52E-02	5.85E-02
**RP11-54C4.3**	0.95	1.93	2.52E-02	5.85E-02
**LRRC37A11P**	0.93	1.91	2.52E-02	5.86E-02
**ATF6B**	0.16	1.12	2.53E-02	5.86E-02
**LINC00887**	0.77	1.71	2.53E-02	5.88E-02
**APLF**	0.46	1.37	2.54E-02	5.88E-02
**ZNF791**	0.27	1.20	2.54E-02	5.88E-02
**EIF1AD**	-0.22	0.86	2.54E-02	5.88E-02
**DENND5B**	-0.35	0.78	2.54E-02	5.90E-02
**KB-1562D12.1**	-0.93	0.52	2.55E-02	5.90E-02
**ABHD17C**	-0.18	0.88	2.55E-02	5.90E-02
**FUS**	-0.16	0.90	2.56E-02	5.93E-02
**ADO**	0.22	1.17	2.56E-02	5.94E-02
**UBC**	0.11	1.08	2.57E-02	5.94E-02
**CHD3**	-0.14	0.91	2.57E-02	5.95E-02
**GGNBP2**	-0.18	0.88	2.57E-02	5.95E-02
**PPID**	-0.21	0.87	2.57E-02	5.95E-02
**ZRSR2**	0.39	1.31	2.57E-02	5.95E-02
**FAM72C**	-0.95	0.52	2.57E-02	5.95E-02
**WDR17**	0.95	1.93	2.57E-02	5.95E-02
**ARSK**	-0.40	0.76	2.58E-02	5.97E-02
**EIF4E3**	0.36	1.29	2.58E-02	5.97E-02
**GNAI3**	-0.16	0.90	2.58E-02	5.97E-02
**REXO1**	-0.19	0.88	2.58E-02	5.98E-02
**RNF213**	0.18	1.14	2.59E-02	5.99E-02
**FAM204A**	-0.22	0.86	2.59E-02	6.00E-02
**AFF3**	0.93	1.90	2.59E-02	6.00E-02
**DNAJC4**	0.33	1.26	2.60E-02	6.00E-02
**TMEM217**	0.80	1.75	2.60E-02	6.01E-02
**DRG1**	-0.20	0.87	2.61E-02	6.03E-02

**FAN1**	0.23	1.17	2.61E-02	6.03E-02
**TBC1D1**	-0.24	0.85	2.61E-02	6.03E-02
**AKT1S1**	-0.17	0.89	2.61E-02	6.03E-02
**PLD1**	0.22	1.17	2.61E-02	6.04E-02
**ZNF235**	-0.73	0.60	2.61E-02	6.04E-02
**ROPN1L**	0.95	1.93	2.61E-02	6.04E-02
**SLC30A7**	0.26	1.20	2.61E-02	6.04E-02
**TREX2**	0.87	1.83	2.62E-02	6.04E-02
**GOT1**	-0.17	0.89	2.62E-02	6.04E-02
**OFD1**	0.27	1.21	2.63E-02	6.07E-02
**LEMD3**	-0.21	0.87	2.63E-02	6.08E-02
**BVES**	0.94	1.92	2.63E-02	6.08E-02
**ACTR1A**	0.15	1.11	2.64E-02	6.10E-02
**HSCB**	0.47	1.39	2.64E-02	6.10E-02
**POC5**	-0.36	0.78	2.64E-02	6.10E-02
**SUGP1**	0.21	1.15	2.64E-02	6.10E-02
**CSNK1E**	0.14	1.10	2.65E-02	6.11E-02
**UNC119B**	0.21	1.15	2.65E-02	6.12E-02
**TBC1D22B**	0.25	1.19	2.65E-02	6.12E-02
**RP11-399B17.1**	0.89	1.86	2.65E-02	6.12E-02
**STUB1**	-0.21	0.86	2.66E-02	6.12E-02
**DDX17**	0.14	1.10	2.67E-02	6.15E-02
**PRDM4**	0.17	1.12	2.67E-02	6.15E-02
**RAPGEF4**	0.53	1.45	2.68E-02	6.17E-02
**STK26**	-0.21	0.86	2.68E-02	6.17E-02
**PKN1**	-0.42	0.75	2.68E-02	6.17E-02
**COA4**	-0.19	0.88	2.68E-02	6.18E-02
**TTC39C**	-0.31	0.81	2.68E-02	6.18E-02
**ZNF670-ZNF695**	-0.85	0.56	2.68E-02	6.18E-02
**KEL**	0.89	1.85	2.68E-02	6.18E-02
**KCNG2**	-0.85	0.56	2.69E-02	6.18E-02
**ARR3**	0.94	1.92	2.69E-02	6.19E-02
**HAS2-AS1**	-0.85	0.56	2.69E-02	6.20E-02
**APCDD1**	-0.71	0.61	2.70E-02	6.21E-02
**LARP7**	-0.22	0.86	2.70E-02	6.21E-02
**CDC123**	-0.16	0.90	2.70E-02	6.22E-02
**RP11-353H3.1**	0.57	1.49	2.71E-02	6.23E-02
**RMDN3**	0.20	1.15	2.71E-02	6.23E-02
**RP11-66B24.2**	0.82	1.77	2.71E-02	6.23E-02
**CINP**	-0.20	0.87	2.71E-02	6.24E-02
**CEP57L1**	-0.31	0.81	2.71E-02	6.24E-02
**C19orf12**	0.32	1.25	2.72E-02	6.25E-02
**ZNF696**	-0.42	0.75	2.72E-02	6.25E-02
**ZNF300**	0.34	1.26	2.72E-02	6.26E-02
**IFI27L2**	0.21	1.16	2.72E-02	6.26E-02
**SETBP1**	-0.31	0.81	2.73E-02	6.27E-02
**AC062029.1**	0.83	1.78	2.74E-02	6.29E-02
**RP11-572P18.1**	-0.46	0.73	2.74E-02	6.29E-02
**AC124789.1**	0.36	1.29	2.74E-02	6.29E-02
**LHFP**	-0.31	0.81	2.74E-02	6.29E-02
**CTU1**	-0.46	0.73	2.74E-02	6.29E-02
**BMP8B**	0.84	1.79	2.74E-02	6.30E-02
**KATNA1**	-0.25	0.84	2.74E-02	6.30E-02
**CD74**	0.63	1.55	2.75E-02	6.30E-02
**NUP88**	-0.16	0.90	2.75E-02	6.30E-02
**CYB561D2**	0.37	1.29	2.75E-02	6.31E-02
**SRMS**	-0.92	0.53	2.75E-02	6.31E-02
**FAM87A**	0.80	1.74	2.75E-02	6.32E-02
**TPM2**	-0.15	0.90	2.76E-02	6.33E-02
**C1orf35**	-0.25	0.84	2.76E-02	6.33E-02
**SCFD1**	0.18	1.13	2.76E-02	6.34E-02
**HIST1H2BF**	0.94	1.91	2.76E-02	6.34E-02
**SMPD2**	-0.29	0.82	2.77E-02	6.34E-02
**NOS3**	-0.77	0.59	2.77E-02	6.34E-02
**KBTBD2**	-0.22	0.86	2.77E-02	6.35E-02
**RP1-266L20.2**	-0.77	0.59	2.77E-02	NA
**RP11-322E11.5**	-0.77	0.59	2.77E-02	NA
**PAN3-AS1**	-0.93	0.52	2.77E-02	6.36E-02
**GEMIN6**	-0.26	0.83	2.78E-02	6.37E-02
**EIF4G3**	0.13	1.10	2.78E-02	6.37E-02
**CPEB2-AS1**	0.91	1.88	2.78E-02	6.37E-02
**PTRH2**	-0.23	0.85	2.78E-02	6.37E-02
**ZNF609**	-0.16	0.89	2.78E-02	6.37E-02
**DHX8**	0.16	1.11	2.78E-02	6.38E-02
**KLK8**	-0.19	0.88	2.79E-02	6.38E-02
**TMED9**	-0.13	0.91	2.79E-02	6.39E-02
**CCSER2**	0.19	1.14	2.79E-02	6.39E-02
**PAWR**	-0.18	0.88	2.80E-02	6.40E-02
**FAM69B**	0.90	1.87	2.80E-02	6.40E-02
**ZNF524**	0.29	1.22	2.80E-02	6.42E-02
**CRBN**	0.30	1.23	2.80E-02	6.42E-02
**BCL2L10**	0.75	1.68	2.81E-02	6.42E-02
**OSBPL1A**	-0.21	0.86	2.81E-02	6.43E-02
**GREB1**	0.92	1.89	2.81E-02	6.43E-02
**NLGN3**	0.82	1.76	2.82E-02	NA
**THUMPD1**	-0.19	0.87	2.82E-02	6.44E-02
**MYLK-AS1**	0.82	1.77	2.82E-02	6.45E-02
**RPL22**	-0.17	0.89	2.82E-02	6.45E-02
**N4BP2L2**	-0.21	0.87	2.82E-02	6.45E-02
**PHF11**	0.29	1.23	2.82E-02	6.46E-02

**SLC25A25**	-0.28	0.82	2.83E-02	6.46E-02
**TCEB2**	0.15	1.11	2.83E-02	6.46E-02
**PDCD4**	0.22	1.16	2.83E-02	6.46E-02
**DHPS**	-0.21	0.87	2.83E-02	6.46E-02
**ARFIP1**	0.20	1.15	2.83E-02	6.46E-02
**SYNPO**	0.28	1.21	2.83E-02	6.46E-02
**FAM63B**	0.22	1.16	2.83E-02	6.46E-02
**MSTO2P**	-0.79	0.58	2.83E-02	6.46E-02
**RPP14**	0.29	1.22	2.84E-02	6.47E-02
**ZNF550**	0.43	1.35	2.84E-02	6.47E-02
**DNAJC30**	0.34	1.27	2.84E-02	6.48E-02
**RP11-525K10.3**	0.93	1.91	2.84E-02	6.48E-02
**LCT**	0.81	1.76	2.84E-02	6.49E-02
**CAPN10-AS1**	-0.75	0.60	2.84E-02	6.49E-02
**TRAPPC12**	0.19	1.14	2.85E-02	6.50E-02
**RNF41**	-0.19	0.87	2.85E-02	6.50E-02
**KDM8**	0.53	1.45	2.85E-02	6.51E-02
**DCP1A**	0.21	1.15	2.86E-02	6.51E-02
**ARFGAP2**	-0.15	0.90	2.86E-02	6.53E-02
**MED30**	-0.37	0.77	2.86E-02	6.53E-02
**UBP1**	-0.16	0.89	2.87E-02	6.54E-02
**NOP9**	-0.18	0.88	2.87E-02	6.55E-02
**FERMT2**	-0.33	0.80	2.88E-02	6.56E-02
**STARD3**	0.20	1.15	2.88E-02	6.56E-02
**SLC25A53**	0.76	1.69	2.88E-02	6.57E-02
**B4GALT6**	0.39	1.31	2.89E-02	6.58E-02
**SLC39A3**	-0.30	0.81	2.89E-02	6.59E-02
**ALKBH5**	-0.15	0.90	2.90E-02	6.60E-02
**RHOT1**	-0.21	0.87	2.90E-02	6.60E-02
**PYGL**	-0.13	0.91	2.90E-02	6.60E-02
**FAM96B**	0.18	1.13	2.90E-02	6.60E-02
**CRTAP**	-0.14	0.91	2.91E-02	6.61E-02
**LA16c-380H5.5**	0.92	1.90	2.91E-02	6.61E-02
**HP1BP3**	-0.15	0.90	2.91E-02	6.62E-02
**ACIN1**	0.13	1.10	2.91E-02	6.63E-02
**MAEA**	0.14	1.11	2.92E-02	6.64E-02
**TMEM80**	0.34	1.27	2.92E-02	6.64E-02
**IFRD1**	0.23	1.17	2.93E-02	6.65E-02
**CIR1**	0.25	1.19	2.93E-02	6.66E-02
**GPR137**	0.28	1.21	2.93E-02	6.66E-02
**CCDC110**	0.80	1.75	2.93E-02	6.67E-02
**ZNF470**	0.47	1.39	2.93E-02	6.67E-02
**ZCCHC6**	0.19	1.14	2.94E-02	6.67E-02
**PKD2**	0.27	1.21	2.94E-02	6.67E-02
**RP11-424G14.1**	-0.76	0.59	2.94E-02	NA
**KIAA0922**	-0.23	0.85	2.94E-02	6.69E-02
**CTD-3128G10.6**	0.92	1.89	2.94E-02	6.69E-02
**MIR3176**	-0.93	0.53	2.95E-02	6.69E-02
**ZNF629**	0.18	1.13	2.95E-02	6.70E-02
**LRRC37A9P**	-0.92	0.53	2.95E-02	6.70E-02
**ACYP2**	0.47	1.38	2.95E-02	6.70E-02
**KIF1BP**	-0.19	0.88	2.96E-02	6.72E-02
**DDX56**	-0.17	0.89	2.96E-02	6.73E-02
**USF2**	-0.16	0.89	2.97E-02	6.74E-02
**AC007246.3**	0.59	1.51	2.97E-02	6.74E-02
**GINM1**	0.23	1.17	2.97E-02	6.74E-02
**HEPHL1**	0.19	1.14	2.97E-02	6.75E-02
**CTD-3184A7.4**	0.70	1.62	2.98E-02	6.75E-02
**ZFP41**	0.38	1.30	2.98E-02	6.76E-02
**OSMR**	0.16	1.12	2.98E-02	6.76E-02
**KRT3**	0.70	1.63	2.98E-02	6.77E-02
**FAM86FP**	0.79	1.72	2.98E-02	6.77E-02
**LEP**	0.92	1.90	2.99E-02	6.78E-02
**DBR1**	-0.26	0.83	2.99E-02	6.79E-02
**NSL1**	0.25	1.19	2.99E-02	6.79E-02
**ATG9A**	-0.17	0.89	3.00E-02	6.80E-02
**NEDD8**	0.17	1.13	3.00E-02	6.81E-02
**FAM200B**	0.31	1.24	3.00E-02	6.81E-02
**BORCS5**	-0.33	0.79	3.01E-02	6.82E-02
**SMPDL3A**	0.34	1.27	3.01E-02	6.82E-02
**ACSF3**	0.25	1.19	3.01E-02	6.82E-02
**SLC25A23**	-0.17	0.89	3.01E-02	6.82E-02
**TIPRL**	-0.18	0.88	3.01E-02	6.82E-02
**RP11-429J17.8**	0.91	1.88	3.01E-02	6.83E-02
**ITGA9-AS1**	0.71	1.63	3.02E-02	6.83E-02
**HDDC3**	-0.45	0.73	3.02E-02	6.83E-02
**GBP4**	-0.92	0.53	3.02E-02	6.84E-02
**COMMD1**	0.23	1.17	3.03E-02	6.85E-02
**R3HDM2**	0.22	1.17	3.03E-02	6.85E-02
**ETNK2**	-0.36	0.78	3.04E-02	6.87E-02
**CTD-2256P15.2**	0.89	1.85	3.04E-02	6.87E-02
**RP11-274B21.4**	0.59	1.50	3.04E-02	6.87E-02
**ARAF**	-0.17	0.89	3.04E-02	6.88E-02
**TBL1Y**	-0.85	0.55	3.04E-02	6.89E-02
**ATP2C1**	-0.17	0.89	3.05E-02	6.89E-02
**NDUFA4**	0.16	1.12	3.05E-02	6.89E-02
**ZNF385B**	0.90	1.87	3.05E-02	6.90E-02
**RP11-44N21.1**	0.91	1.87	3.05E-02	6.90E-02
**PRRT3-AS1**	-0.89	0.54	3.06E-02	6.90E-02
**RBMS1P1**	-0.89	0.54	3.06E-02	6.91E-02

**CYTH4**	0.92	1.89	3.06E-02	6.92E-02
**FAM208B**	-0.15	0.90	3.07E-02	6.93E-02
**GLRX5**	-0.20	0.87	3.07E-02	6.93E-02
**RNMT**	-0.20	0.87	3.07E-02	6.94E-02
**SIAH3**	-0.87	0.55	3.07E-02	6.94E-02
**PHF21A**	0.24	1.18	3.07E-02	6.94E-02
**GLT8D2**	0.92	1.89	3.08E-02	6.95E-02
**RP5-821D11.7**	0.58	1.49	3.08E-02	6.96E-02
**IFITM2**	-0.36	0.78	3.09E-02	6.96E-02
**TAOK3**	-0.17	0.89	3.09E-02	6.96E-02
**HIST1H3H**	0.84	1.79	3.09E-02	6.97E-02
**LINC00493**	0.37	1.29	3.09E-02	6.97E-02
**ZNF623**	0.23	1.18	3.09E-02	6.97E-02
**NAP1L1P1**	0.92	1.89	3.09E-02	6.98E-02
**RP11-17M16.2**	0.75	1.68	3.10E-02	6.98E-02
**PPP2R2B**	0.47	1.39	3.10E-02	6.98E-02
**FAM216A**	0.34	1.27	3.10E-02	6.99E-02
**PSMD6**	0.14	1.10	3.10E-02	6.99E-02
**RP11-317N8.5**	0.92	1.89	3.10E-02	6.99E-02
**FARSA**	-0.17	0.89	3.10E-02	6.99E-02
**FBXL17**	-0.29	0.82	3.10E-02	6.99E-02
**MRPS15**	0.13	1.10	3.10E-02	6.99E-02
**FAM120AOS**	0.23	1.17	3.10E-02	6.99E-02
**DNAJB6**	0.17	1.12	3.11E-02	7.00E-02
**TICAM1**	-0.19	0.88	3.11E-02	7.01E-02
**RP11-734K21.2**	0.91	1.87	3.12E-02	7.03E-02
**ASH1L**	-0.16	0.89	3.12E-02	7.04E-02
**LURAP1L**	-0.26	0.83	3.13E-02	7.04E-02
**CLIP1**	-0.13	0.91	3.13E-02	7.04E-02
**PABPN1**	-0.19	0.88	3.13E-02	7.04E-02
**AC019178.2**	0.79	1.73	3.13E-02	NA
**RANBP6**	0.24	1.18	3.13E-02	7.06E-02
**ZNF253**	0.54	1.46	3.14E-02	7.07E-02
**STIM1**	0.14	1.10	3.15E-02	7.10E-02
**HERC2P2**	0.42	1.34	3.16E-02	7.10E-02
**GNG12-AS1**	0.91	1.87	3.16E-02	7.11E-02
**GAS1**	-0.60	0.66	3.17E-02	7.12E-02
**NAGLU**	-0.29	0.82	3.17E-02	7.12E-02
**MARCKSL1**	-0.19	0.88	3.17E-02	7.12E-02
**MESP1**	0.91	1.88	3.17E-02	7.13E-02
**ENAH**	-0.15	0.90	3.17E-02	7.13E-02
**HIST1H2BH**	-0.84	0.56	3.18E-02	7.14E-02
**ZDHHC6**	-0.22	0.86	3.18E-02	7.14E-02
**C6orf141**	0.32	1.25	3.18E-02	7.15E-02
**DNAJA4**	0.22	1.16	3.18E-02	7.15E-02
**PRICKLE4**	0.28	1.22	3.18E-02	7.15E-02
**LRP12**	0.24	1.18	3.18E-02	7.15E-02
**RFX2**	-0.26	0.83	3.19E-02	7.16E-02
**ANAPC7**	0.18	1.13	3.19E-02	7.16E-02
**PPIL2**	-0.19	0.88	3.19E-02	7.17E-02
**RPS19P1**	0.46	1.37	3.20E-02	7.19E-02
**CTSW**	-0.88	0.54	3.20E-02	7.19E-02
**DNMBP**	0.16	1.12	3.21E-02	7.22E-02
**RP11-456K23.1**	0.90	1.86	3.22E-02	7.22E-02
**POU5F1**	-0.83	0.56	3.22E-02	7.22E-02
**FMO4**	0.62	1.53	3.22E-02	7.22E-02
**COA5**	0.38	1.30	3.22E-02	7.22E-02
**PLA2G15**	0.31	1.24	3.23E-02	7.24E-02
**RAB2B**	0.29	1.22	3.23E-02	7.24E-02
**HGH1**	-0.80	0.57	3.23E-02	7.25E-02
**C6orf25**	0.79	1.73	3.23E-02	7.25E-02
**RARG**	-0.14	0.91	3.23E-02	7.25E-02
**TMEM104**	0.19	1.14	3.24E-02	7.26E-02
**RUSC1**	-0.19	0.88	3.24E-02	7.26E-02
**POM121**	0.19	1.14	3.24E-02	7.26E-02
**SMARCC2**	-0.15	0.90	3.24E-02	7.27E-02
**UBQLN4**	-0.18	0.88	3.25E-02	7.27E-02
**SLC27A1**	0.33	1.26	3.25E-02	7.27E-02
**C9orf172**	0.56	1.47	3.25E-02	7.27E-02
**PPP2R1A**	-0.14	0.90	3.25E-02	7.29E-02
**MYLIP**	0.56	1.47	3.26E-02	7.29E-02
**SURF6**	-0.17	0.89	3.26E-02	7.30E-02
**RP11-38M8.1**	0.90	1.87	3.26E-02	7.30E-02
**GPR173**	-0.91	0.53	3.26E-02	7.30E-02
**COBLL1**	0.15	1.11	3.26E-02	7.30E-02
**FOXK1**	0.18	1.13	3.28E-02	7.34E-02
**RHOXF1-AS1**	0.91	1.87	3.28E-02	7.35E-02
**HHIPL1**	0.83	1.78	3.28E-02	7.35E-02
**HNRNPA1L2**	-0.55	0.68	3.28E-02	7.35E-02
**GON4L**	0.18	1.14	3.28E-02	7.35E-02
**BCAP31**	-0.15	0.90	3.29E-02	7.35E-02
**FBXO7**	-0.15	0.90	3.29E-02	7.35E-02
**ARMCX5-GPRASP2**	0.57	1.48	3.29E-02	7.35E-02
**TMEM218**	-0.32	0.80	3.29E-02	7.35E-02
**LINC01550**	0.33	1.25	3.30E-02	7.37E-02
**ATP5S**	-0.34	0.79	3.30E-02	7.38E-02
**C19orf43**	-0.15	0.90	3.30E-02	7.38E-02
**AGPAT2**	-0.19	0.87	3.31E-02	7.39E-02
**ARL14EP**	0.28	1.21	3.31E-02	7.40E-02
**EFNB2**	-0.13	0.91	3.31E-02	7.40E-02

AIFM1	0.17	1.12	3.32E-02	7.43E-02
HARS2	-0.21	0.86	3.34E-02	7.45E-02
ADAM19	-0.24	0.85	3.34E-02	7.46E-02
CBL	-0.17	0.89	3.35E-02	7.48E-02
GCNT2	-0.37	0.77	3.35E-02	7.48E-02
ASCC1	0.20	1.15	3.35E-02	7.48E-02
ZBTB17	0.21	1.16	3.35E-02	7.48E-02
TXNDC5	-0.81	0.57	3.36E-02	7.50E-02
TPRKB	-0.25	0.84	3.36E-02	7.50E-02
ARL9	-0.90	0.53	3.36E-02	7.51E-02
ZNF580	0.32	1.24	3.37E-02	7.51E-02
GHET1	0.79	1.73	3.37E-02	7.51E-02
C19orf25	0.25	1.19	3.37E-02	7.52E-02
CTD-3157E16.1	0.75	1.68	3.37E-02	7.52E-02
DPY19L3	0.30	1.23	3.37E-02	7.52E-02
SEC24B	-0.15	0.90	3.37E-02	7.52E-02
SLC4A11	0.21	1.16	3.38E-02	7.53E-02
RP11-30P6.6	0.72	1.64	3.39E-02	7.56E-02
MAP1A	0.57	1.48	3.39E-02	7.56E-02
CCDC157	0.68	1.61	3.39E-02	7.56E-02
PSMD10P2	0.90	1.87	3.40E-02	7.57E-02
INPP5A	-0.26	0.84	3.40E-02	7.57E-02
XAB2	0.18	1.13	3.40E-02	7.57E-02
FADS6	-0.90	0.54	3.40E-02	7.57E-02
AC016708.2	-0.54	0.69	3.40E-02	7.57E-02
EEF1A2	-0.64	0.64	3.40E-02	7.58E-02
PPIP5K2	-0.20	0.87	3.40E-02	7.58E-02
ACKR4	-0.85	0.56	3.41E-02	7.59E-02
NIPA2	-0.18	0.88	3.41E-02	7.59E-02
MAT2A	-0.13	0.91	3.41E-02	7.60E-02
METTL14	0.22	1.17	3.42E-02	7.60E-02
RUFY3	-0.26	0.84	3.42E-02	7.60E-02
GTPBP1	0.17	1.13	3.42E-02	7.60E-02
LINC01572	-0.74	0.60	3.42E-02	7.60E-02
RP4-694A7.2	0.64	1.55	3.42E-02	7.61E-02
CUL3	-0.16	0.90	3.43E-02	7.62E-02
TCHH	0.72	1.65	3.43E-02	7.62E-02
TDRD9	-0.89	0.54	3.43E-02	7.62E-02
STX17	0.25	1.19	3.43E-02	7.63E-02
EIF3M	-0.14	0.91	3.44E-02	7.64E-02
RP11-473M20.5	-0.68	0.62	3.44E-02	7.65E-02
AC009061.1	-0.87	0.55	3.45E-02	7.66E-02
YKT6	0.13	1.09	3.45E-02	7.66E-02
SMAD7	0.36	1.28	3.45E-02	7.66E-02
VAT1	-0.14	0.91	3.45E-02	7.66E-02
ATG101	-0.28	0.83	3.46E-02	7.69E-02
TAPBP	0.15	1.11	3.46E-02	7.69E-02
AP001258.4	0.71	1.63	3.46E-02	7.69E-02
KRT16P5	-0.83	0.56	3.47E-02	7.70E-02
SLC11A1	0.86	1.82	3.47E-02	7.70E-02
RNA5SP283	0.90	1.87	3.47E-02	7.71E-02
MGMT	-0.24	0.85	3.48E-02	7.72E-02
KXD1	-0.20	0.87	3.48E-02	7.73E-02
VGF	-0.84	0.56	3.48E-02	7.73E-02
CLN8	0.24	1.19	3.48E-02	7.73E-02
PHF6	0.22	1.16	3.49E-02	7.73E-02
ROR1	0.41	1.33	3.49E-02	7.74E-02
RP11-527N22.2	-0.49	0.71	3.49E-02	7.74E-02
NFKBIB	-0.22	0.86	3.49E-02	7.75E-02
ATP9B	0.28	1.21	3.51E-02	7.78E-02
TMEM102	-0.32	0.80	3.51E-02	7.78E-02
COX7A2L	-0.16	0.90	3.51E-02	7.79E-02
DDX49	0.19	1.14	3.51E-02	7.79E-02
MERTK	0.79	1.73	3.52E-02	7.79E-02
RAB33B	0.37	1.30	3.52E-02	7.80E-02
MIR210HG	-0.39	0.76	3.52E-02	7.80E-02
TK2	-0.35	0.79	3.52E-02	7.80E-02
ZNRD1	-0.32	0.80	3.52E-02	7.81E-02
DEF8	-0.17	0.89	3.53E-02	7.81E-02
KLK5	0.14	1.10	3.53E-02	7.81E-02
SVIL-AS1	0.24	1.18	3.53E-02	7.81E-02
RP11-290F24.6	-0.84	0.56	3.53E-02	7.82E-02
MGST1	-0.16	0.90	3.53E-02	7.82E-02
NDUFS7	0.24	1.18	3.53E-02	7.82E-02
RPS7	-0.15	0.90	3.53E-02	7.82E-02
ZSCAN30	0.31	1.24	3.54E-02	7.83E-02
GSDMA	-0.31	0.81	3.54E-02	7.84E-02
PYROXD2	0.67	1.59	3.56E-02	7.87E-02
AC005019.2	-0.87	0.55	3.56E-02	7.87E-02
C1orf43	-0.13	0.91	3.56E-02	7.88E-02
KATNBL1	-0.24	0.85	3.57E-02	7.89E-02
TPH1	0.87	1.83	3.57E-02	7.89E-02
NRADDP	-0.87	0.55	3.58E-02	7.91E-02
ZNF529-AS1	0.67	1.59	3.60E-02	7.95E-02
CAPZB	-0.13	0.92	3.60E-02	7.95E-02
NUDT8	0.40	1.32	3.60E-02	7.96E-02
COG8	-0.30	0.81	3.60E-02	7.96E-02
ATXN7L2	-0.39	0.76	3.62E-02	7.99E-02
SMU1	0.16	1.12	3.62E-02	8.00E-02
CSMD2	0.87	1.82	3.62E-02	8.00E-02

**MAGEF1**	-0.27	0.83	3.63E-02	8.02E-02
**ARL10**	-0.28	0.82	3.63E-02	8.02E-02
**MAPK8IP2**	-0.72	0.61	3.63E-02	8.03E-02
**GOLGA4**	-0.17	0.89	3.64E-02	8.04E-02
**C6orf136**	-0.27	0.83	3.64E-02	8.04E-02
**EIF1**	0.12	1.08	3.64E-02	8.05E-02
**RP11-91J19.3**	-0.85	0.55	3.65E-02	8.05E-02
**PIGL**	0.30	1.23	3.65E-02	8.05E-02
**MAFA**	0.85	1.81	3.65E-02	8.06E-02
**FBXL8**	0.66	1.58	3.66E-02	8.07E-02
**EGLN1**	-0.18	0.88	3.66E-02	8.07E-02
**NAP1L1**	-0.15	0.90	3.66E-02	8.08E-02
**NUDT19**	-0.21	0.86	3.67E-02	8.09E-02
**SNX18**	-0.23	0.85	3.67E-02	8.10E-02
**ALPP**	0.85	1.81	3.68E-02	8.11E-02
**TECR**	0.16	1.12	3.68E-02	8.12E-02
**TMEM180**	-0.33	0.80	3.69E-02	8.13E-02
**MED11**	0.27	1.21	3.70E-02	8.15E-02
**GNB2**	0.13	1.09	3.70E-02	8.15E-02
**TTC7B**	0.24	1.18	3.70E-02	8.15E-02
**ERV3-1**	0.44	1.36	3.71E-02	8.17E-02
**RP11-115H13.1**	-0.71	0.61	3.72E-02	8.19E-02
**CHUK**	0.19	1.14	3.72E-02	8.19E-02
**PRKAG2**	0.20	1.15	3.72E-02	8.19E-02
**COIL**	-0.22	0.86	3.73E-02	8.20E-02
**CTA-217C2.2**	-0.82	0.57	3.73E-02	8.21E-02
**MXRA8**	0.43	1.35	3.73E-02	8.21E-02
**RP3-337H4.8**	0.85	1.80	3.73E-02	8.21E-02
**ZNF285**	-0.83	0.56	3.74E-02	8.22E-02
**WNT5B**	0.89	1.85	3.74E-02	8.23E-02
**JARID2**	0.17	1.12	3.74E-02	8.23E-02
**ZNF330**	-0.21	0.86	3.74E-02	8.23E-02
**AC005624.2**	-0.87	0.55	3.75E-02	8.25E-02
**MFN2**	-0.11	0.93	3.75E-02	8.25E-02
**CCDC28B**	-0.56	0.68	3.76E-02	8.26E-02
**SPATA6**	-0.49	0.71	3.76E-02	8.28E-02
**RPS15AP17**	-0.56	0.68	3.76E-02	8.28E-02
**FAM120C**	0.34	1.26	3.77E-02	8.30E-02
**GEMIN7**	-0.38	0.77	3.78E-02	8.30E-02
**TAF7**	0.21	1.16	3.78E-02	8.31E-02
**IKZF5**	0.31	1.24	3.78E-02	8.31E-02
**PINX1**	0.23	1.18	3.79E-02	8.32E-02
**CTD-2201G3.1**	-0.83	0.56	3.79E-02	8.33E-02
**ZNF513**	0.28	1.21	3.80E-02	8.34E-02
**PIK3CB**	0.17	1.13	3.80E-02	8.35E-02
**CYBA**	0.18	1.13	3.80E-02	8.35E-02
**KLHL8**	-0.24	0.84	3.81E-02	8.37E-02
**SLC41A2**	0.82	1.77	3.81E-02	8.37E-02
**ZNF219**	-0.17	0.89	3.82E-02	8.38E-02
**DNAAF3**	0.71	1.64	3.82E-02	8.39E-02
**HDAC9**	-0.60	0.66	3.83E-02	8.40E-02
**LAGE3**	0.32	1.25	3.83E-02	8.40E-02
**CTD-2179L22.1**	0.87	1.83	3.83E-02	8.40E-02
**FAM160B1**	-0.28	0.83	3.83E-02	8.40E-02
**SYNJ2BP**	0.23	1.18	3.83E-02	8.40E-02
**PIP5K1C**	0.20	1.15	3.83E-02	8.41E-02
**RAB8A**	-0.17	0.89	3.83E-02	8.41E-02
**HAPLN3**	0.44	1.36	3.83E-02	8.41E-02
**DHRS13**	-0.60	0.66	3.85E-02	8.44E-02
**ADIRF-AS1**	0.35	1.28	3.85E-02	8.44E-02
**POLDIP2**	0.13	1.09	3.85E-02	8.44E-02
**RP11-326K13.4**	0.88	1.84	3.85E-02	8.45E-02
**HOXD8**	-0.50	0.71	3.85E-02	8.45E-02
**MRPL13**	0.19	1.14	3.86E-02	8.45E-02
**TP53INP2**	0.32	1.25	3.86E-02	8.45E-02
**PHTF1**	0.34	1.26	3.86E-02	8.45E-02
**UBR2**	0.19	1.14	3.86E-02	8.46E-02
**KDM5D**	-0.21	0.86	3.86E-02	8.46E-02
**ZNF341**	0.34	1.27	3.86E-02	8.46E-02
**SLC37A1**	-0.33	0.80	3.86E-02	8.46E-02
**AC093627.9**	-0.88	0.54	3.86E-02	8.46E-02
**LINC01578**	-0.34	0.79	3.87E-02	8.47E-02
**TPI1P2**	0.76	1.70	3.87E-02	8.48E-02
**ETV2**	-0.74	0.60	3.88E-02	8.49E-02
**SH3BGRL2**	0.26	1.19	3.88E-02	8.49E-02
**PHKA1**	0.17	1.13	3.88E-02	8.50E-02
**TGOLN2**	-0.13	0.92	3.88E-02	8.50E-02
**MEP1A**	0.85	1.80	3.89E-02	8.52E-02
**CCBE1**	-0.24	0.84	3.89E-02	8.52E-02
**SERPINF1**	0.50	1.41	3.90E-02	8.52E-02
**ADAMTSL1**	-0.82	0.57	3.90E-02	8.52E-02
**LINC01126**	0.85	1.80	3.90E-02	8.52E-02
**MEA1**	0.18	1.13	3.90E-02	8.53E-02
**TEF**	-0.25	0.84	3.90E-02	8.53E-02
**ZNF518A**	-0.23	0.85	3.90E-02	8.54E-02
**RNASEL**	-0.30	0.81	3.91E-02	8.54E-02
**TOMM70A**	-0.17	0.89	3.91E-02	8.54E-02
**MYL12B**	0.12	1.08	3.91E-02	8.54E-02
**PGRMC1**	-0.17	0.89	3.92E-02	8.56E-02
**KRT16P6**	0.36	1.28	3.92E-02	8.57E-02

**COL8A1**	-0.45	0.73	3.93E-02	8.58E-02
**VSIG1**	0.52	1.44	3.94E-02	8.60E-02
**CHD6**	0.19	1.14	3.94E-02	8.60E-02
**PIM2**	-0.33	0.80	3.95E-02	8.63E-02
**ITGA6**	-0.13	0.92	3.96E-02	8.64E-02
**ZNF74**	-0.26	0.84	3.96E-02	8.64E-02
**OR7E91P**	-0.88	0.55	3.97E-02	8.67E-02
**KAT2A**	-0.19	0.88	3.97E-02	8.67E-02
**CXCR1**	0.71	1.64	3.98E-02	8.67E-02
**IDH3A**	0.18	1.13	3.98E-02	8.68E-02
**RSAD2**	0.79	1.74	3.98E-02	8.68E-02
**C5orf38**	0.48	1.40	3.98E-02	8.69E-02
**EFCAB11**	-0.35	0.79	3.99E-02	8.70E-02
**HSD17B14**	0.85	1.81	3.99E-02	8.71E-02
**RP11-568K15.1**	0.36	1.29	3.99E-02	8.71E-02
**BMPR2**	0.20	1.15	3.99E-02	8.71E-02
**DYNC2LI1**	-0.30	0.81	4.00E-02	8.71E-02
**ZMYM6**	-0.40	0.76	4.00E-02	8.72E-02
**LINC00885**	0.83	1.77	4.00E-02	8.73E-02
**ZSCAN22**	0.38	1.30	4.01E-02	8.73E-02
**HSD11B1**	0.84	1.80	4.01E-02	8.73E-02
**POLN**	0.83	1.78	4.01E-02	8.73E-02
**RP11-164J13.1**	0.77	1.71	4.02E-02	8.75E-02
**RP11-382A20.3**	0.17	1.13	4.03E-02	8.78E-02
**GJA3**	0.85	1.81	4.04E-02	8.79E-02
**RPS3AP6**	0.53	1.45	4.05E-02	8.82E-02
**GABRB3**	0.65	1.57	4.06E-02	8.83E-02
**IGF2BP2-AS1**	0.87	1.83	4.06E-02	8.84E-02
**ASAH2B**	0.66	1.58	4.06E-02	8.85E-02
**NINL**	0.43	1.35	4.07E-02	8.87E-02
**LINC01465**	-0.85	0.55	4.07E-02	8.87E-02
**SEC61G**	0.25	1.19	4.08E-02	8.89E-02
**GTF2H2B**	-0.83	0.56	4.08E-02	8.89E-02
**PMEL**	0.62	1.54	4.09E-02	8.90E-02
**C1orf226**	0.31	1.24	4.09E-02	8.91E-02
**NOP10**	-0.13	0.91	4.09E-02	8.91E-02
**RP11-1060J15.9**	0.84	1.79	4.10E-02	8.91E-02
**RWDD1**	-0.17	0.89	4.11E-02	8.93E-02
**TRIM17**	-0.72	0.61	4.11E-02	8.93E-02
**DNAJC15**	-0.20	0.87	4.11E-02	8.94E-02
**PRR5**	-0.31	0.81	4.11E-02	8.94E-02
**CREB5**	-0.41	0.75	4.11E-02	8.94E-02
**PNPLA4**	0.23	1.17	4.12E-02	8.95E-02
**ADCK2**	-0.24	0.85	4.12E-02	8.95E-02
**LNX1**	-0.49	0.71	4.12E-02	8.95E-02
**PFDN4**	0.28	1.22	4.13E-02	8.96E-02
**TRAPPC11**	-0.16	0.89	4.13E-02	8.96E-02
**RP11-1275H24.1**	0.68	1.60	4.13E-02	8.98E-02
**TCF7L2**	-0.28	0.82	4.14E-02	8.99E-02
**ASPDH**	0.87	1.83	4.14E-02	8.99E-02
**PNP**	0.14	1.10	4.14E-02	8.99E-02
**RP11-122K13.12**	0.80	1.74	4.14E-02	9.00E-02
**FAM134A**	-0.18	0.88	4.14E-02	9.00E-02
**ZNF789**	0.47	1.39	4.15E-02	9.00E-02
**MZT1**	-0.31	0.80	4.15E-02	9.01E-02
**RP11-546D6.3**	-0.82	0.57	4.17E-02	9.05E-02
**TNFRSF1A**	0.13	1.09	4.18E-02	9.06E-02
**ATG5**	-0.22	0.86	4.18E-02	9.06E-02
**BMPER**	0.84	1.79	4.18E-02	9.07E-02
**AC007193.6**	0.86	1.82	4.18E-02	9.07E-02
**ZNF266**	0.26	1.20	4.18E-02	9.07E-02
**RRP15**	-0.21	0.87	4.19E-02	9.07E-02
**PDAP1**	-0.12	0.92	4.19E-02	9.09E-02
**ZBTB21**	0.22	1.17	4.21E-02	9.12E-02
**TMEM170A**	-0.26	0.83	4.21E-02	9.12E-02
**RP11-320L11.2**	0.85	1.81	4.21E-02	9.13E-02
**ARHGAP42**	0.52	1.43	4.22E-02	9.14E-02
**TUBB1**	-0.86	0.55	4.22E-02	9.14E-02
**ZNF667**	0.77	1.71	4.23E-02	9.16E-02
**CTD-2574D22.4**	0.79	1.73	4.25E-02	9.20E-02
**TSPAN6**	0.25	1.19	4.25E-02	9.20E-02
**SP3**	0.15	1.11	4.25E-02	9.20E-02
**ATP1A1**	0.10	1.07	4.25E-02	9.21E-02
**LANCL3**	-0.58	0.67	4.25E-02	9.21E-02
**FMOD**	-0.86	0.55	4.26E-02	9.21E-02
**PPM1G**	-0.13	0.91	4.26E-02	9.21E-02
**PGLS**	-0.17	0.89	4.26E-02	9.21E-02
**C8orf37**	0.50	1.42	4.26E-02	9.21E-02
**TBX2**	0.83	1.78	4.26E-02	9.22E-02
**USP33**	0.18	1.13	4.26E-02	9.22E-02
**SNHG16**	-0.21	0.87	4.26E-02	9.22E-02
**TMEM86A**	0.23	1.17	4.27E-02	9.23E-02
**VTCN1**	0.86	1.82	4.27E-02	9.23E-02
**KIF13B**	0.21	1.16	4.27E-02	9.23E-02
**RP13-128O4.3**	0.86	1.82	4.28E-02	9.25E-02
**MED20**	0.21	1.16	4.28E-02	9.25E-02
**C1QBP**	-0.12	0.92	4.28E-02	9.25E-02
**AC083899.3**	-0.74	0.60	4.29E-02	9.27E-02
**HERC3**	-0.14	0.91	4.30E-02	9.28E-02
**SLC33A1**	-0.19	0.88	4.30E-02	9.29E-02

**ZNF689**	-0.34	0.79	4.30E-02	9.29E-02
**CELP**	0.80	1.74	4.31E-02	9.30E-02
**ANAPC5**	-0.13	0.92	4.31E-02	9.30E-02
**SOSTDC1**	-0.68	0.62	4.31E-02	NA
**42801**	0.16	1.12	4.31E-02	9.31E-02
**RP11-561I11.3**	0.80	1.74	4.31E-02	9.31E-02
**SH3GLB1**	-0.14	0.91	4.32E-02	9.32E-02
**NEMP1**	-0.19	0.88	4.32E-02	9.33E-02
**PALLD**	0.12	1.09	4.32E-02	9.33E-02
**XAF1**	0.67	1.59	4.33E-02	9.34E-02
**NRD1**	0.12	1.09	4.33E-02	9.35E-02
**42993**	0.15	1.11	4.33E-02	9.35E-02
**MYEOV2**	0.21	1.16	4.34E-02	9.35E-02
**SERINC5**	-0.15	0.90	4.34E-02	9.35E-02
**ZNF554**	0.55	1.46	4.35E-02	9.37E-02
**ATN1**	0.17	1.13	4.35E-02	9.38E-02
**PHF23**	-0.16	0.90	4.35E-02	9.39E-02
**SCDP1**	-0.85	0.55	4.36E-02	9.39E-02
**PRKACB**	0.24	1.18	4.36E-02	9.39E-02
**LIN9**	-0.30	0.81	4.36E-02	9.41E-02
**PRKCQ-AS1**	-0.40	0.76	4.36E-02	9.41E-02
**RP11-774O3.3**	0.79	1.73	4.37E-02	9.41E-02
**ALOX12P2**	0.64	1.55	4.37E-02	9.42E-02
**YES1**	0.17	1.13	4.37E-02	9.43E-02
**DDX55**	-0.22	0.86	4.38E-02	9.43E-02
**ATP2B4**	-0.13	0.92	4.38E-02	9.44E-02
**SOAT1**	-0.17	0.89	4.38E-02	9.44E-02
**GMEB1**	-0.26	0.84	4.39E-02	9.45E-02
**RP11-259O2.2**	-0.82	0.56	4.39E-02	9.45E-02
**FAM118B**	0.26	1.20	4.39E-02	9.45E-02
**VGLL1**	-0.19	0.88	4.39E-02	9.45E-02
**VDAC3**	-0.14	0.91	4.39E-02	9.45E-02
**MYOM3**	0.69	1.61	4.39E-02	9.46E-02
**ESYT2**	0.12	1.09	4.40E-02	9.46E-02
**RP11-930P14.2**	0.80	1.74	4.40E-02	9.47E-02
**ADRB1**	0.79	1.73	4.40E-02	9.47E-02
**RP11-110I1.11**	0.85	1.81	4.40E-02	9.47E-02
**ZCRB1**	-0.17	0.89	4.40E-02	9.47E-02
**NXPE3**	0.28	1.21	4.41E-02	9.48E-02
**RP3-424M6.4**	0.84	1.79	4.41E-02	9.49E-02
**YWHAB**	-0.10	0.93	4.42E-02	9.50E-02
**NET1**	-0.11	0.93	4.42E-02	9.50E-02
**WAPL**	-0.16	0.90	4.42E-02	9.50E-02
**BRAT1**	-0.19	0.88	4.42E-02	9.51E-02
**DSC3**	0.12	1.09	4.42E-02	9.51E-02
**RAB3GAP1**	0.14	1.10	4.43E-02	9.51E-02
**C3orf17**	0.20	1.15	4.43E-02	9.51E-02
**SLC19A2**	-0.26	0.83	4.44E-02	9.53E-02
**BRAP**	0.19	1.14	4.44E-02	9.53E-02
**PRKRIP1**	0.23	1.18	4.44E-02	9.54E-02
**POU2F1**	-0.23	0.86	4.44E-02	9.54E-02
**TSPAN9**	0.19	1.14	4.45E-02	9.55E-02
**KLHL7-AS1**	0.86	1.81	4.45E-02	9.55E-02
**RP11-342K6.1**	-0.34	0.79	4.45E-02	9.55E-02
**RARS2**	-0.22	0.86	4.45E-02	9.56E-02
**TNRC6B**	-0.19	0.88	4.46E-02	9.57E-02
**GAS2L2**	0.83	1.78	4.46E-02	9.57E-02
**DNAJC19**	-0.26	0.84	4.46E-02	9.57E-02
**RP3-395M20.12**	-0.79	0.58	4.47E-02	9.58E-02
**SH3BP5-AS1**	0.71	1.63	4.47E-02	9.59E-02
**FRY**	-0.85	0.55	4.47E-02	9.59E-02
**SSR4**	0.14	1.10	4.48E-02	9.60E-02
**MPC2**	0.22	1.17	4.49E-02	9.63E-02
**XRN2**	-0.13	0.92	4.49E-02	9.63E-02
**HYAL2**	-0.18	0.88	4.49E-02	9.63E-02
**HOXC12**	-0.63	0.65	4.49E-02	9.63E-02
**CCDC171**	-0.72	0.61	4.50E-02	9.64E-02
**TRAPPC2L**	0.18	1.14	4.50E-02	9.64E-02
**PARG**	0.26	1.20	4.50E-02	9.65E-02
**SLC38A6**	0.49	1.40	4.50E-02	9.65E-02
**SZT2**	0.19	1.14	4.51E-02	9.66E-02
**RP11-540O11.1**	0.85	1.81	4.51E-02	9.66E-02
**RP11-1348G14.4**	0.82	1.77	4.51E-02	9.66E-02
**RP11-45M22.2**	0.49	1.40	4.51E-02	9.67E-02
**TMEM132B**	0.85	1.80	4.52E-02	9.67E-02
**DSC1**	-0.43	0.74	4.52E-02	9.68E-02
**UBXN6**	-0.16	0.90	4.53E-02	9.70E-02
**MZF1-AS1**	0.81	1.75	4.53E-02	9.70E-02
**PODXL**	0.59	1.51	4.54E-02	9.71E-02
**KTN1**	-0.15	0.90	4.54E-02	9.71E-02
**ODF3L1**	0.83	1.77	4.54E-02	9.71E-02
**IK**	0.13	1.10	4.54E-02	9.72E-02
**FAM86C1**	-0.37	0.78	4.54E-02	9.72E-02
**NDUFA8**	0.17	1.13	4.55E-02	9.74E-02
**SYNGAP1**	0.25	1.19	4.56E-02	9.74E-02
**SCIN**	0.79	1.73	4.56E-02	9.74E-02
**ANKRD6**	0.53	1.44	4.56E-02	9.74E-02
**MAGOH**	-0.20	0.87	4.56E-02	9.74E-02
**GOSR1**	0.16	1.12	4.56E-02	9.75E-02
**LINC01224**	-0.67	0.63	4.56E-02	NA

**IDI1**	-0.32	0.80	4.56E-02	9.75E-02
**RAB30**	0.36	1.29	4.57E-02	9.76E-02
**POP5**	0.24	1.18	4.57E-02	9.76E-02
**SPTY2D1**	0.17	1.12	4.57E-02	9.76E-02
**RP11-452L6.6**	-0.74	0.60	4.57E-02	9.76E-02
**ANKRD24**	0.82	1.77	4.57E-02	9.76E-02
**ZG16B**	0.50	1.41	4.57E-02	9.77E-02
**RP11-166P13.3**	0.85	1.80	4.57E-02	9.77E-02
**PIGP**	0.30	1.23	4.58E-02	9.77E-02
**KCNK15**	0.53	1.45	4.58E-02	9.77E-02
**SMAD4**	-0.19	0.88	4.59E-02	9.80E-02
**TCAP**	0.85	1.80	4.60E-02	9.82E-02
**HSPE1**	-0.23	0.85	4.60E-02	9.83E-02
**IL20RB-AS1**	-0.75	0.60	4.60E-02	9.83E-02
**LINC00672**	0.85	1.80	4.60E-02	9.83E-02
**RAB37**	0.78	1.72	4.61E-02	9.84E-02
**IL17RD**	0.45	1.37	4.62E-02	9.85E-02
**ZNF24**	0.17	1.12	4.62E-02	9.85E-02
**ZNF780B**	0.44	1.36	4.62E-02	9.85E-02
**ZNF440**	0.30	1.23	4.62E-02	9.86E-02
**NKX3-1**	0.60	1.51	4.63E-02	9.87E-02
**C5orf51**	0.17	1.13	4.63E-02	9.87E-02
**GPR161**	-0.22	0.86	4.64E-02	9.88E-02
**COL5A3**	-0.24	0.85	4.64E-02	9.89E-02
**TRIM52-AS1**	-0.48	0.72	4.64E-02	9.89E-02
**RP11-166D19.1**	0.37	1.29	4.64E-02	9.90E-02
**C10orf12**	-0.31	0.81	4.65E-02	9.90E-02
**CLOCK**	-0.15	0.90	4.66E-02	9.92E-02
**GRSF1**	-0.15	0.90	4.66E-02	9.92E-02
**MARS2**	-0.25	0.84	4.66E-02	9.92E-02
**MAP3K14-AS1**	0.58	1.49	4.66E-02	9.92E-02
**RP11-755F10.3**	-0.84	0.56	4.67E-02	9.94E-02
**METTL21B**	0.42	1.34	4.67E-02	9.94E-02
**EVI2B**	-0.82	0.57	4.67E-02	9.94E-02
**EAF2**	0.52	1.43	4.67E-02	9.94E-02
**INPP5F**	-0.18	0.88	4.67E-02	9.94E-02
**WTAP**	-0.17	0.89	4.67E-02	9.94E-02
**NUDT16**	-0.18	0.88	4.67E-02	9.94E-02
**RP1-234P15.4**	0.80	1.74	4.67E-02	9.94E-02
**DCAF8**	0.21	1.16	4.68E-02	9.95E-02
**UBE2K**	-0.14	0.91	4.68E-02	9.95E-02
**CD6**	0.55	1.47	4.68E-02	9.95E-02
**JAK3**	0.75	1.68	4.68E-02	9.96E-02
**MUT**	0.19	1.14	4.69E-02	9.96E-02
**MAN1A1**	0.34	1.27	4.70E-02	9.98E-02
**STK4-AS1**	-0.84	0.56	4.71E-02	1.00E-01
**ZFAT**	0.30	1.23	4.71E-02	1.00E-01
**ITPR2**	0.20	1.15	4.71E-02	1.00E-01
**LYST**	0.19	1.14	4.71E-02	1.00E-01
**CBFB**	-0.17	0.89	4.72E-02	1.00E-01
**UBE2E1**	0.14	1.10	4.72E-02	1.00E-01
**CHSY1**	0.18	1.13	4.73E-02	1.01E-01
**SYDE2**	-0.45	0.73	4.73E-02	1.01E-01
**API5**	0.13	1.10	4.74E-02	1.01E-01
**CENPQ**	-0.37	0.77	4.74E-02	1.01E-01
**RP11-384K6.6**	0.36	1.29	4.76E-02	1.01E-01
**FAM149B1**	0.24	1.18	4.77E-02	1.01E-01
**C2orf69**	-0.25	0.84	4.77E-02	1.01E-01
**WASH1**	0.44	1.36	4.77E-02	1.01E-01
**PROSER1**	-0.15	0.90	4.78E-02	1.01E-01
**SPAST**	-0.26	0.83	4.79E-02	1.02E-01
**NUTF2**	-0.14	0.91	4.79E-02	1.02E-01
**SNTA1**	0.34	1.26	4.80E-02	1.02E-01
**TMEM139**	0.42	1.33	4.80E-02	1.02E-01
**ENOX1**	-0.84	0.56	4.80E-02	1.02E-01
**GGH**	-0.18	0.88	4.80E-02	1.02E-01
**C2orf15**	-0.69	0.62	4.80E-02	1.02E-01
**EVA1A**	-0.38	0.77	4.81E-02	1.02E-01
**ALDH9A1**	-0.16	0.90	4.82E-02	1.02E-01
**DLEU2L**	0.83	1.78	4.82E-02	1.02E-01
**RP11-93H12.4**	0.81	1.75	4.82E-02	1.02E-01
**PCDHGC5**	0.80	1.74	4.82E-02	1.02E-01
**PKD1L1**	0.84	1.79	4.83E-02	1.02E-01
**GBP1P1**	-0.83	0.56	4.83E-02	1.02E-01
**CD99L2**	-0.29	0.82	4.83E-02	1.02E-01
**RP11-166B2.1**	0.77	1.70	4.83E-02	1.02E-01
**CTC1**	0.19	1.14	4.83E-02	1.02E-01
**SNX30**	-0.20	0.87	4.84E-02	1.02E-01
**CEBPB**	0.17	1.13	4.84E-02	1.02E-01
**CBR3-AS1**	0.73	1.66	4.84E-02	1.02E-01
**GPR37L1**	0.84	1.79	4.84E-02	1.02E-01
**ADSSL1**	0.55	1.47	4.85E-02	1.03E-01
**BTG4**	0.84	1.79	4.86E-02	1.03E-01
**KCNJ14**	-0.83	0.56	4.86E-02	1.03E-01
**TMTC1**	-0.20	0.87	4.86E-02	1.03E-01
**SUOX**	0.26	1.20	4.86E-02	1.03E-01
**ADARB1**	0.20	1.15	4.87E-02	1.03E-01
**NACA3P**	-0.68	0.63	4.87E-02	1.03E-01
**RASAL1**	0.49	1.40	4.87E-02	1.03E-01
**PADI2**	0.78	1.72	4.87E-02	1.03E-01

**PP14571**	-0.83	0.56	4.88E-02	1.03E-01
**LINC01106**	-0.68	0.62	4.88E-02	1.03E-01
**EXOC3L2**	0.72	1.65	4.88E-02	1.03E-01
**DGCR11**	-0.52	0.70	4.88E-02	1.03E-01
**UBE2L3**	-0.12	0.92	4.89E-02	1.03E-01
**RP11-288H12.3**	0.83	1.77	4.89E-02	1.03E-01
**EMILIN2**	0.70	1.63	4.90E-02	1.03E-01
**ZDHHC24**	-0.24	0.85	4.90E-02	1.04E-01
**APCDD1L**	0.48	1.40	4.91E-02	1.04E-01
**PPIAP19**	0.47	1.38	4.91E-02	1.04E-01
**NKILA**	0.76	1.70	4.92E-02	1.04E-01
**RDH5**	0.83	1.78	4.92E-02	1.04E-01
**RP1-151F17.1**	0.83	1.78	4.93E-02	1.04E-01
**ZRANB3**	-0.28	0.82	4.93E-02	1.04E-01
**RHOT2**	0.16	1.12	4.94E-02	1.04E-01
**LINC00324**	0.80	1.74	4.94E-02	1.04E-01
**GMCL1**	0.21	1.16	4.95E-02	1.04E-01
**NFKB2**	-0.18	0.88	4.95E-02	1.04E-01
**GLT8D1**	-0.18	0.88	4.95E-02	1.04E-01
**SLC25A34**	0.80	1.74	4.95E-02	1.04E-01
**LRIG2**	0.27	1.21	4.96E-02	1.05E-01
**PSMA4**	-0.14	0.91	4.96E-02	1.05E-01
**TARDBP**	-0.15	0.90	4.96E-02	1.05E-01
**SH3BGR**	0.68	1.60	4.97E-02	1.05E-01
**SYT17**	0.67	1.59	4.98E-02	1.05E-01
**HCN2**	0.78	1.71	4.98E-02	1.05E-01
**SEC13**	-0.13	0.91	4.99E-02	1.05E-01
**YBX1P10**	0.83	1.78	5.00E-02	1.05E-01
**UBA1**	0.11	1.08	5.00E-02	1.05E-01

**DMSO vs BI**					**shCT vs shp53**				
**Gene_ID**	**log2FoldChange**	**Fold Change**	**pvalue**	**padj**	**Gene_ID**	**log2FoldChange**	**Fold Change**	**pvalue**	**padj**
**H19**	-3.40	0.09	4.82E-211	6.54E-207	**ETHE1**	-2.14	0.23	1.45E-112	1.96E-108
**THBD**	-1.63	0.32	1.35E-116	9.13E-113	**WDTC1**	-2.01	0.25	3.13E-106	2.11E-102
**FAM111B**	-1.62	0.32	1.03E-47	2.33E-44	**AP2M1**	-1.36	0.39	1.04E-67	2.21E-64
**UNG**	-1.55	0.34	4.49E-56	1.52E-52	**CAV2**	-1.33	0.40	1.61E-74	5.42E-71
**LY6D**	-1.36	0.39	2.30E-59	1.04E-55	**FTL**	-1.51	0.35	1.68E-102	7.54E-99
**SPRR1A**	-1.05	0.48	5.62E-50	1.53E-46	**CDH1**	-1.15	0.45	1.13E-69	3.04E-66
**ATP12A**	-1.38	0.39	2.30E-46	4.45E-43	**ACO2**	-1.47	0.36	1.15E-67	2.21E-64
**MCM5**	-1.07	0.48	5.54E-46	9.40E-43	**RWDD1**	-1.85	0.28	1.71E-65	2.89E-62
**CALML5**	-1.70	0.31	3.43E-45	5.17E-42	**AP1M1**	-1.54	0.34	8.00E-63	1.20E-59
**KRT15**	-1.12	0.46	6.54E-45	8.87E-42	**ARPC5**	-1.27	0.42	3.68E-58	4.96E-55
**MCM2**	-1.03	0.49	1.07E-39	1.32E-36	**SIRT2**	-1.49	0.36	1.47E-55	1.80E-52
**RRM2**	-0.80	0.57	3.85E-36	4.36E-33	**CDC25B**	1.44	2.72	9.70E-54	1.09E-50
**PLA2G4E**	-1.40	0.38	2.19E-35	2.29E-32	**RHOC**	-1.45	0.37	1.68E-53	1.75E-50
**EDN2**	-2.24	0.21	1.61E-34	1.56E-31	**TP53**	-1.64	0.32	2.55E-52	2.46E-49
**PCNA**	-0.81	0.57	2.19E-34	1.98E-31	**LBR**	1.42	2.68	4.90E-51	4.41E-48
**WDR76**	-1.28	0.41	1.59E-32	1.35E-29	**POLR2A**	-0.98	0.51	1.11E-50	9.36E-48
**CHAC1**	-1.94	0.26	1.29E-31	1.03E-28	**TOM1L2**	-1.18	0.44	9.42E-49	7.36E-46
**GJB2**	-0.68	0.62	1.96E-28	1.48E-25	**STIM1**	-1.13	0.46	9.83E-49	7.36E-46
**MCM6**	-0.84	0.56	4.14E-28	2.96E-25	**CHPF**	-1.07	0.48	1.09E-47	7.77E-45
**SPRR2A**	-1.13	0.46	5.82E-28	3.95E-25	**TNFRSF21**	-1.03	0.49	4.72E-46	3.18E-43
**SBSN**	-0.72	0.61	7.05E-28	4.56E-25	**IL1B**	-1.29	0.41	1.50E-45	9.62E-43
**CA12**	-0.64	0.64	8.53E-27	5.26E-24	**GXYLT1**	1.36	2.57	1.84E-45	1.13E-42
**TIMP3**	-0.80	0.57	1.60E-26	9.44E-24	**SERPINB2**	-1.13	0.46	1.67E-44	9.79E-42
**KRTDAP**	-0.79	0.58	3.30E-26	1.87E-23	**SCAMP3**	-1.37	0.39	8.24E-44	4.63E-41
**AQP3**	-0.70	0.61	3.58E-26	1.95E-23	**TMEM115**	-1.54	0.34	9.25E-44	4.99E-41
**THBS2**	-0.74	0.60	2.84E-25	1.48E-22	**DAB2IP**	-1.41	0.38	9.64E-44	5.00E-41
**ALOX15B**	-0.81	0.57	9.55E-25	4.80E-22	**C11orf24**	-1.61	0.33	2.11E-43	1.06E-40
**SYT8**	-1.21	0.43	1.28E-24	6.21E-22	**CDIP1**	-1.40	0.38	4.33E-43	2.09E-40
**E2F8**	-1.04	0.48	6.33E-23	2.96E-20	**DCP2**	1.19	2.28	5.34E-43	2.48E-40
**H1F0**	-0.65	0.64	7.26E-23	3.28E-20	**MED28**	-1.36	0.39	9.48E-43	4.26E-40
**KRT78**	-1.09	0.47	1.84E-22	8.07E-20	**SLC35A2**	-1.43	0.37	3.20E-41	1.39E-38
**ASAP1**	0.68	1.60	6.37E-22	2.70E-19	**PLOD3**	-1.27	0.42	1.45E-40	6.10E-38
**NDRG1**	-0.65	0.64	7.18E-22	2.95E-19	**LRP11**	-0.96	0.52	2.11E-40	8.63E-38
**OAS2**	-0.74	0.60	8.74E-22	3.49E-19	**TRAK2**	1.12	2.18	3.95E-39	1.57E-36
**NUPR1**	-1.07	0.48	1.23E-21	4.75E-19	**CUL9**	-1.04	0.49	4.63E-39	1.79E-36
**SAT1**	0.71	1.64	1.50E-21	5.64E-19	**LIMK2**	-0.88	0.54	6.86E-39	2.57E-36
**SLC2A1**	-0.61	0.65	1.81E-21	6.64E-19	**MDM2**	-1.23	0.43	7.28E-39	2.65E-36
**TSC22D3**	-1.09	0.47	2.50E-21	8.92E-19	**GJB3**	-1.11	0.46	1.15E-37	4.08E-35
**FADS1**	-1.04	0.49	3.19E-21	1.11E-18	**LRP10**	-1.06	0.48	7.14E-37	2.47E-34
**SPRR3**	-0.69	0.62	3.48E-20	1.18E-17	**B4GALT3**	-1.49	0.36	1.31E-36	4.41E-34
**DDIT4**	-0.64	0.64	3.73E-20	1.23E-17	**LSS**	-0.87	0.55	2.88E-35	9.48E-33
**ARRDC3**	-0.96	0.51	5.68E-20	1.83E-17	**AHCYL2**	-1.11	0.46	8.43E-35	2.71E-32
**CTSD**	-0.66	0.63	9.22E-20	2.91E-17	**PLS3**	-0.80	0.57	5.15E-34	1.62E-31
**TEAD1**	0.59	1.51	1.85E-19	5.71E-17	**CEP170B**	-1.04	0.49	5.92E-34	1.82E-31
**SYNM**	-1.26	0.42	1.92E-19	5.80E-17	**PI4KB**	-1.21	0.43	1.25E-33	3.74E-31
**DTL**	-0.94	0.52	3.66E-19	1.08E-16	**KIAA1522**	-0.94	0.52	1.89E-33	5.56E-31
**MUC1**	-0.79	0.58	4.50E-19	1.30E-16	**BCL7B**	-1.45	0.36	4.08E-33	1.17E-30
**ALDH3B2**	-0.84	0.56	4.88E-19	1.38E-16	**KRT6B**	-0.76	0.59	4.30E-33	1.21E-30
**GINS2**	-0.85	0.55	7.26E-19	2.01E-16	**PARP10**	-1.14	0.45	6.02E-33	1.66E-30
**FBXO5**	-1.03	0.49	1.12E-18	3.05E-16	**IGFL3**	-1.21	0.43	1.18E-32	3.13E-30
**ZFP36L2**	-0.57	0.68	1.31E-18	3.49E-16	**CERS2**	-1.54	0.34	1.18E-32	3.13E-30
**EPPK1**	-0.97	0.51	1.98E-18	5.18E-16	**TIMM17B**	-1.33	0.40	1.24E-32	3.22E-30
**PTK6**	-0.73	0.60	2.25E-18	5.76E-16	**SSR2**	-1.10	0.47	2.42E-32	6.17E-30
**GLTP**	-0.55	0.68	2.31E-18	5.82E-16	**IL1RN**	-0.92	0.53	3.46E-32	8.63E-30
**ELMSAN1**	-0.82	0.57	2.77E-18	6.84E-16	**LHPP**	-1.64	0.32	4.90E-31	1.20E-28
**IGFL1**	-0.97	0.51	2.94E-18	7.13E-16	**ALDH18A1**	-1.08	0.47	5.14E-31	1.24E-28
**GJB6**	-0.73	0.60	3.02E-18	7.20E-16	**NLRX1**	-1.10	0.47	1.45E-30	3.42E-28
**PHLDB2**	0.60	1.52	3.57E-18	8.36E-16	**ALDOC**	-1.63	0.32	1.91E-30	4.43E-28
**E2F2**	-1.06	0.48	4.11E-18	9.46E-16	**WDR34**	-1.05	0.48	5.85E-30	1.34E-27

AHNAK2	-0.62	0.65	9.27E-18	2.10E-15
TGFBI	-0.69	0.62	1.41E-17	3.13E-15
STX3	0.71	1.63	2.46E-17	5.40E-15
WNT10A	-1.34	0.39	2.56E-17	5.51E-15
DPYSL2	-0.97	0.51	2.96E-17	6.28E-15
MCM7	-0.61	0.66	3.03E-17	6.33E-15
FADS2	-0.85	0.56	3.22E-17	6.61E-15
CNFN	-1.25	0.42	7.80E-17	1.58E-14
LTB4R	-0.74	0.60	8.00E-17	1.60E-14
FGFBP1	-0.47	0.72	9.76E-17	1.92E-14
HOPX	-0.71	0.61	1.28E-16	2.48E-14
CYR61	0.68	1.60	1.42E-16	2.71E-14
TACC3	0.58	1.50	1.55E-16	2.93E-14
DCBLD2	0.60	1.52	1.73E-16	3.21E-14
TRIB3	-0.59	0.66	2.04E-16	3.74E-14
VSNL1	-0.81	0.57	2.38E-16	4.30E-14
KLF5	-0.49	0.71	2.64E-16	4.71E-14
CAPN14	-1.39	0.38	3.72E-16	6.55E-14
KRT24	-0.78	0.58	4.96E-16	8.63E-14
FUBP1	0.51	1.43	5.52E-16	9.47E-14
TXNIP	0.49	1.41	6.11E-16	1.04E-13
FAM83C	-0.62	0.65	7.27E-16	1.22E-13
MYO10	0.52	1.43	1.27E-15	2.11E-13
TGM1	-0.50	0.71	1.87E-15	3.06E-13
CLIC3	-0.64	0.64	1.93E-15	3.13E-13
DUT	-0.72	0.61	2.51E-15	4.00E-13
ASS1	-0.81	0.57	2.82E-15	4.45E-13
SERPINB13	-0.87	0.55	2.90E-15	4.52E-13
SP1	-0.53	0.69	3.28E-15	5.05E-13
STC2	-1.05	0.48	3.69E-15	5.63E-13
ARTN	-0.88	0.54	5.29E-15	7.98E-13
CRABP2	-0.50	0.71	7.57E-15	1.13E-12
SRSF5	0.57	1.48	9.45E-15	1.39E-12
MCAM	-1.01	0.50	1.04E-14	1.52E-12
PALMD	-1.09	0.47	1.06E-14	1.52E-12
H2AFX	-0.77	0.59	1.15E-14	1.65E-12
NYNRIN	-0.68	0.62	1.58E-14	2.24E-12
PTPRU	-0.72	0.61	1.63E-14	2.28E-12
TXNRD1	0.62	1.53	1.75E-14	2.43E-12
HSPA8	0.44	1.35	1.89E-14	2.59E-12
INPP5D	-0.74	0.60	2.14E-14	2.90E-12
FLRT2	-0.71	0.61	2.26E-14	3.03E-12
G0S2	0.93	1.90	2.33E-14	3.10E-12
TFCP2L1	-0.53	0.69	2.37E-14	3.12E-12
CCL20	1.40	2.64	2.74E-14	3.57E-12
CDC20	0.53	1.45	3.35E-14	4.33E-12
AURKA	0.59	1.51	3.59E-14	4.60E-12
MCM3	-0.54	0.69	4.01E-14	5.09E-12
HMGB2	-0.52	0.70	5.33E-14	6.70E-12
EGLN3	-1.12	0.46	5.50E-14	6.85E-12
TNFAIP3	0.67	1.59	6.64E-14	8.19E-12
SDC3	-0.58	0.67	9.76E-14	1.19E-11
TYMP	-0.65	0.64	1.04E-13	1.26E-11
SPINK5	-0.63	0.65	1.16E-13	1.40E-11
RGS3	-0.76	0.59	1.20E-13	1.43E-11
COL5A3	-0.95	0.52	1.24E-13	1.46E-11
ADAM8	0.62	1.54	1.32E-13	1.54E-11
ANKRD11	0.49	1.41	1.36E-13	1.57E-11
MN1	-0.75	0.60	1.60E-13	1.84E-11
TNNI2	-1.24	0.42	1.63E-13	1.86E-11
IGFBP6	-0.64	0.64	2.05E-13	2.32E-11
FMNL2	0.57	1.48	2.11E-13	2.36E-11
CLSPN	-0.78	0.58	2.13E-13	2.37E-11
THSD4	0.53	1.44	2.46E-13	2.72E-11
EMP2	-0.57	0.68	3.19E-13	3.49E-11
OSMR	0.54	1.46	3.47E-13	3.77E-11
PHACTR3	-0.92	0.53	3.75E-13	4.04E-11
FAM213A	-0.57	0.67	5.07E-13	5.42E-11
MT2A	0.58	1.49	5.13E-13	5.44E-11
ADGRF1	-0.72	0.61	5.59E-13	5.88E-11
TCERG1	0.50	1.41	5.86E-13	6.12E-11
CCNE2	-1.26	0.42	6.55E-13	6.78E-11
MYO1E	0.42	1.34	6.69E-13	6.87E-11
GOLGA3	0.53	1.45	6.73E-13	6.87E-11
DSG4	-1.00	0.50	7.03E-13	7.11E-11
CASP14	-0.83	0.56	7.08E-13	7.12E-11
ADM	-0.73	0.60	7.50E-13	7.49E-11
MFSD2A	-0.70	0.62	7.58E-13	7.51E-11
ARSJ	0.64	1.56	7.98E-13	7.85E-11
APOL6	-0.86	0.55	1.31E-12	1.28E-10
LRP1	-0.77	0.59	1.33E-12	1.29E-10
ARL4C	-0.47	0.72	1.52E-12	1.47E-10
SNX9	0.52	1.44	1.56E-12	1.49E-10
PLA2G4F	-0.72	0.61	1.57E-12	1.49E-10
SERPINE1	-0.42	0.75	2.41E-12	2.27E-10
GOLGA8B	1.09	2.12	2.65E-12	2.48E-10
SPG7	0.56	1.47	2.73E-12	2.54E-10
MCM4	-0.42	0.75	2.88E-12	2.66E-10
SLC7A8	-0.55	0.68	3.88E-12	3.56E-10
MLLT4	0.46	1.37	4.25E-12	3.87E-10

AP1B1	-0.83	0.56	9.04E-30	2.03E-27
SPINK6	-0.86	0.55	1.51E-29	3.34E-27
LINC00707	-0.97	0.51	1.83E-29	3.99E-27
DICER1	0.80	1.74	4.78E-29	1.02E-26
CCNYL1	-1.12	0.46	5.49E-29	1.16E-26
IVL	-0.85	0.56	1.70E-28	3.48E-26
DUSP23	-2.09	0.23	1.70E-28	3.48E-26
TMEM104	-1.32	0.40	2.19E-28	4.41E-26
TRIM22	-0.87	0.55	1.12E-27	2.23E-25
TMEM245	0.94	1.92	3.14E-27	6.14E-25
LASP1	-1.01	0.50	3.71E-27	7.15E-25
MARVELD1	-1.09	0.47	4.02E-27	7.64E-25
PDLIM5	-0.90	0.54	6.50E-27	1.22E-24
IL6R	1.24	2.36	8.72E-27	1.61E-24
CLDN1	-0.98	0.51	1.07E-26	1.95E-24
POLG	-0.86	0.55	1.80E-26	3.23E-24
IPO13	-0.87	0.55	2.00E-26	3.54E-24
ZBTB4	-0.79	0.58	2.03E-26	3.54E-24
HMGB2	0.99	1.99	2.05E-26	3.54E-24
DEPDC1	1.04	2.05	2.81E-26	4.79E-24
UXS1	-1.09	0.47	3.09E-26	5.20E-24
HSPA5	0.70	1.62	3.47E-26	5.78E-24
ATG4D	-1.16	0.45	4.05E-26	6.67E-24
CYP2S1	-1.26	0.42	4.21E-26	6.84E-24
KRT16P6	-0.75	0.59	4.75E-26	7.63E-24
POLDIP2	-0.92	0.53	5.07E-26	8.04E-24
TBC1D13	-1.20	0.43	1.04E-25	1.64E-23
YOD1	1.13	2.19	2.79E-25	4.32E-23
RARG	-0.89	0.54	3.24E-25	4.96E-23
CMTM4	0.75	1.69	4.29E-25	6.50E-23
PLCH2	-0.71	0.61	6.36E-25	9.54E-23
TAGLN2	-0.72	0.61	6.72E-25	9.96E-23
CDR2L	-0.89	0.54	1.13E-24	1.66E-22
ARHGAP19	1.07	2.10	1.16E-24	1.69E-22
TMEM184B	-0.93	0.53	1.49E-24	2.14E-22
WIZ	-1.07	0.48	2.39E-24	3.40E-22
ANLN	0.90	1.87	3.03E-24	4.26E-22
SLC12A6	-0.89	0.54	5.50E-24	7.64E-22
TRIM44	-0.84	0.56	1.62E-23	2.23E-21
ANKRD46	1.17	2.25	3.30E-23	4.50E-21
TAPBP	-0.74	0.60	3.44E-23	4.64E-21
CDC25A	0.98	1.98	4.17E-23	5.57E-21
SLC9A1	-1.01	0.50	4.45E-23	5.88E-21
DAG1	-0.82	0.57	5.96E-23	7.81E-21
FOXP4	-1.38	0.38	8.22E-23	1.07E-20
INSIG1	0.71	1.63	1.07E-22	1.38E-20
ISG15	-0.74	0.60	1.37E-22	1.74E-20
KIAA0040	-0.95	0.52	1.81E-22	2.28E-20
ARL6IP1	0.81	1.75	2.19E-22	2.74E-20
TNIP1	-0.75	0.59	2.24E-22	2.78E-20
SH3GL1	-0.87	0.55	2.72E-22	3.33E-20
HMMR	1.00	2.01	3.86E-22	4.69E-20
ITPKC	-0.79	0.58	5.28E-22	6.36E-20
TNRC6A	0.71	1.64	5.80E-22	6.92E-20
PITPNM3	-0.98	0.51	6.66E-22	7.88E-20
EXTL3	-0.99	0.50	6.91E-22	8.10E-20
AREG	-1.30	0.41	9.29E-22	1.08E-19
NGRN	-1.10	0.47	9.71E-22	1.12E-19
SDC4	-0.65	0.64	1.51E-21	1.73E-19
ADAMTS1	-0.97	0.51	2.00E-21	2.26E-19
TMEM109	-0.83	0.56	2.01E-21	2.26E-19
CSNK1G2	-0.88	0.55	2.15E-21	2.40E-19
PGM2L1	1.41	2.65	2.81E-21	3.10E-19
EPS8L2	-0.71	0.61	2.85E-21	3.13E-19
VGLL4	-0.89	0.54	3.17E-21	3.45E-19
NUP58	0.71	1.63	3.21E-21	3.46E-19
ADRBK1	-0.73	0.60	3.62E-21	3.87E-19
C6orf132	-0.71	0.61	3.78E-21	4.02E-19
SEMA4A	-1.28	0.41	4.07E-21	4.29E-19
PTGS2	-1.52	0.35	4.67E-21	4.88E-19
WASF2	-0.78	0.58	5.23E-21	5.43E-19
CAPNS1	-0.65	0.64	6.05E-21	6.23E-19
PHGDH	0.67	1.59	6.15E-21	6.29E-19
MXD4	-1.02	0.49	6.80E-21	6.90E-19
MFAP5	-0.69	0.62	6.97E-21	7.01E-19
PPARA	-0.98	0.51	1.08E-20	1.08E-18
TNS4	-0.70	0.62	1.25E-20	1.24E-18
GADD45A	-0.66	0.63	1.36E-20	1.34E-18
GLYR1	-0.79	0.58	1.66E-20	1.62E-18
PGAM1	-0.73	0.60	2.13E-20	2.07E-18
MAST4	-0.73	0.60	2.19E-20	2.11E-18
CNP	-0.74	0.60	2.28E-20	2.18E-18
EPGN	-0.82	0.57	2.87E-20	2.73E-18
ST14	-0.60	0.66	3.05E-20	2.87E-18
C1orf74	-0.94	0.52	3.19E-20	2.99E-18
SH3PXD2B	-0.76	0.59	4.05E-20	3.76E-18
ADAMTSL4	-0.88	0.55	4.58E-20	4.23E-18
MIR205HG	-0.78	0.58	5.10E-20	4.68E-18
IFI44L	-0.87	0.55	5.18E-20	4.72E-18
H19	0.82	1.76	5.23E-20	4.73E-18

MAL	-1.04	0.49	4.50E-12	4.06E-10
DSC2	-0.54	0.69	4.54E-12	4.06E-10
CKS2	0.55	1.47	4.55E-12	4.06E-10
CA2	-0.55	0.69	5.87E-12	5.20E-10
PSAT1	-0.43	0.74	6.97E-12	6.14E-10
ARL4D	-0.65	0.64	8.12E-12	7.11E-10
CCNB1	0.42	1.34	8.48E-12	7.34E-10
LAMB3	0.49	1.41	8.49E-12	7.34E-10
ARHGAP29	0.57	1.49	8.70E-12	7.47E-10
SVIL	0.48	1.39	8.88E-12	7.58E-10
MET	0.47	1.39	9.86E-12	8.35E-10
TMEM97	-0.66	0.63	9.90E-12	8.35E-10
SULT2B1	-0.56	0.68	1.06E-11	8.86E-10
PRODH	-0.74	0.60	1.15E-11	9.56E-10
E2F1	-0.88	0.54	1.92E-11	1.59E-09
TP73	-1.25	0.42	1.93E-11	1.59E-09
MYH9	0.40	1.32	1.98E-11	1.62E-09
EPAS1	0.39	1.31	2.08E-11	1.69E-09
ALOX12B	-1.25	0.42	2.40E-11	1.94E-09
SLC7A2	0.76	1.70	2.43E-11	1.95E-09
IL1RN	-0.42	0.75	2.54E-11	2.03E-09
PLBD1	-0.77	0.59	2.58E-11	2.05E-09
ZNF503	-0.70	0.61	2.88E-11	2.27E-09
SH3BP4	0.47	1.38	3.07E-11	2.40E-09
SLC23A2	0.57	1.49	3.37E-11	2.63E-09
ADARB1	0.62	1.53	3.57E-11	2.76E-09
SMURF2	0.56	1.47	3.70E-11	2.85E-09
SAMD4A	0.67	1.59	3.72E-11	2.85E-09
BUB1	0.45	1.37	4.00E-11	3.05E-09
GABRP	-0.77	0.58	4.23E-11	3.21E-09
MYO1B	0.43	1.34	4.28E-11	3.22E-09
ACAP3	-0.63	0.65	4.59E-11	3.44E-09
PRC1	0.40	1.32	5.27E-11	3.93E-09
MIB2	-0.67	0.63	5.36E-11	3.98E-09
CDCP1	0.42	1.34	5.39E-11	3.98E-09
IFRD1	-0.65	0.64	6.68E-11	4.90E-09
ADM2	-1.14	0.45	6.76E-11	4.93E-09
MKNK2	-0.41	0.75	7.34E-11	5.33E-09
TOPBP1	-0.52	0.70	7.40E-11	5.34E-09
CD44	0.37	1.29	7.57E-11	5.43E-09
PPP1R9B	-0.54	0.69	8.42E-11	6.01E-09
SAMD1	-0.60	0.66	8.49E-11	6.03E-09
OLFML2A	-1.05	0.48	8.69E-11	6.14E-09
S1PR5	-0.59	0.67	1.11E-10	7.82E-09
TRIM47	-0.90	0.54	1.15E-10	8.04E-09
ANGPTL4	-0.49	0.71	1.18E-10	8.18E-09
AK6	0.81	1.76	1.25E-10	8.68E-09
NHS	0.63	1.55	1.29E-10	8.88E-09
JUNB	-0.53	0.69	1.30E-10	8.88E-09
LIMA1	0.41	1.33	1.35E-10	9.19E-09
TFPI2	0.41	1.33	1.39E-10	9.43E-09
KREMEN2	-1.17	0.44	1.42E-10	9.57E-09
PXN	0.45	1.37	1.44E-10	9.65E-09
FNDC3B	0.52	1.43	1.45E-10	9.69E-09
UNC5B	-0.76	0.59	1.46E-10	9.75E-09
MAP3K6	-0.50	0.71	1.53E-10	1.01E-08
IFI6	-0.60	0.66	1.59E-10	1.05E-08
HIP1	-0.80	0.58	1.66E-10	1.09E-08
PALLD	0.46	1.37	1.79E-10	1.17E-08
CAPN2	0.34	1.26	1.81E-10	1.18E-08
ANKRD35	-0.89	0.54	1.85E-10	1.19E-08
MIDN	-0.59	0.67	1.85E-10	1.19E-08
CAB39	0.42	1.34	1.87E-10	1.19E-08
PPP6R3	0.47	1.39	1.96E-10	1.25E-08
SDC4	0.37	1.29	2.31E-10	1.47E-08
BCL7A	0.49	1.40	2.36E-10	1.49E-08
DDX21	0.39	1.31	2.70E-10	1.69E-08
DHFR	-0.69	0.62	3.04E-10	1.90E-08
CLASRP	0.59	1.50	3.12E-10	1.94E-08
KRT10	-0.50	0.71	3.65E-10	2.26E-08
DOCK4	0.56	1.48	3.72E-10	2.29E-08
MAP2K1	0.42	1.33	3.76E-10	2.31E-08
WNT5A	-0.70	0.61	3.78E-10	2.31E-08
MGEA5	0.47	1.38	3.83E-10	2.33E-08
CXCL14	-0.87	0.55	3.88E-10	2.35E-08
FAM213B	-0.56	0.68	3.97E-10	2.39E-08
HMMR	0.61	1.53	4.00E-10	2.40E-08
C2orf54	-0.60	0.66	4.12E-10	2.46E-08
RFC2	-0.55	0.68	4.20E-10	2.50E-08
SPRR1B	-0.43	0.74	4.33E-10	2.57E-08
EPHB3	-0.59	0.66	4.40E-10	2.60E-08
ZBTB40	0.59	1.50	4.53E-10	2.66E-08
UNC13D	-0.73	0.60	4.64E-10	2.72E-08
PPP1R3C	-0.83	0.56	5.14E-10	2.99E-08
TRIO	0.44	1.36	5.73E-10	3.32E-08
CBX5	-0.43	0.74	6.26E-10	3.62E-08
NEDD4L	0.50	1.41	6.34E-10	3.65E-08
NET1	-0.36	0.78	6.40E-10	3.66E-08
MTCO3P12	0.55	1.46	6.64E-10	3.79E-08
SLC29A1	-0.59	0.67	6.74E-10	3.83E-08

FBLIM1	-0.81	0.57	5.29E-20	4.76E-18
WEE1	0.85	1.80	7.52E-20	6.71E-18
TXNRD1	0.72	1.65	8.52E-20	7.56E-18
FKBP1A	-0.63	0.64	9.14E-20	8.06E-18
STX12	-0.83	0.56	9.40E-20	8.23E-18
SLC35A4	-2.12	0.23	1.40E-19	1.22E-17
NLRP1	-0.73	0.60	1.79E-19	1.54E-17
B4GAT1	0.94	1.92	1.85E-19	1.59E-17
CPA4	-0.86	0.55	1.86E-19	1.59E-17
GPRC5A	-0.67	0.63	1.97E-19	1.67E-17
MED24	-0.86	0.55	2.22E-19	1.87E-17
KITLG	-0.93	0.52	2.28E-19	1.91E-17
DCBLD2	-0.80	0.57	2.47E-19	2.06E-17
BCL2L1	-0.82	0.57	2.67E-19	2.21E-17
LY6D	-0.85	0.55	3.14E-19	2.59E-17
ZER1	-0.77	0.59	3.38E-19	2.77E-17
PRX	-2.23	0.21	3.75E-19	3.05E-17
PLK2	-0.68	0.62	4.01E-19	3.24E-17
SHCBP1	0.93	1.91	4.56E-19	3.66E-17
HSP90B1	0.64	1.56	8.73E-19	6.97E-17
ADGRG1	-0.62	0.65	9.22E-19	7.31E-17
SLC35F6	-0.79	0.58	9.65E-19	7.61E-17
IRF6	-0.57	0.67	1.02E-18	7.98E-17
DENND6A	0.75	1.68	1.09E-18	8.54E-17
HSPB8	-0.86	0.55	1.14E-18	8.82E-17
LIMK1	-0.96	0.51	1.69E-18	1.30E-16
DDB2	-0.87	0.55	2.38E-18	1.82E-16
ITGA3	-1.62	0.33	3.21E-18	2.45E-16
HAS3	-0.80	0.58	3.41E-18	2.59E-16
CMTR1	-0.71	0.61	4.97E-18	3.75E-16
GNAI2	-0.66	0.63	5.27E-18	3.95E-16
SOX9	-0.69	0.62	5.59E-18	4.16E-16
RAB1B	-0.69	0.62	5.69E-18	4.20E-16
SIDT2	-0.80	0.57	5.70E-18	4.20E-16
ATG9A	-0.68	0.63	7.44E-18	5.45E-16
MYCL	-1.06	0.48	7.59E-18	5.54E-16
MAP4K2	-0.92	0.53	7.79E-18	5.65E-16
JAG1	-0.64	0.64	8.31E-18	6.00E-16
MUC19	-2.74	0.15	8.65E-18	6.21E-16
FAM120B	-0.98	0.51	9.20E-18	6.56E-16
MICALL1	-0.67	0.63	9.48E-18	6.73E-16
MINK1	-0.57	0.67	1.01E-17	7.14E-16
SNRPB	-0.74	0.60	1.02E-17	7.19E-16
QSOX1	-0.60	0.66	1.54E-17	1.07E-15
RP11-115D19.1	-1.02	0.49	1.91E-17	1.33E-15
TMEM40	-0.69	0.62	2.10E-17	1.45E-15
C1orf116	-0.61	0.65	2.22E-17	1.53E-15
C6orf120	0.89	1.85	2.25E-17	1.54E-15
NEDD4	0.81	1.75	2.29E-17	1.56E-15
IK	-0.69	0.62	2.44E-17	1.65E-15
NDUFA3	-0.92	0.53	3.64E-17	2.45E-15
ZNF367	1.13	2.18	4.00E-17	2.68E-15
BUB1	0.86	1.82	4.07E-17	2.72E-15
SPARC	0.61	1.52	5.60E-17	3.72E-15
SMIM13	0.87	1.83	6.20E-17	4.10E-15
LUZP1	-0.62	0.65	6.58E-17	4.33E-15
ASS1	0.68	1.60	6.94E-17	4.54E-15
MYO3B	-1.00	0.50	7.36E-17	4.79E-15
CHD7	0.70	1.62	7.80E-17	5.06E-15
MMP24-AS1	-0.99	0.50	8.54E-17	5.51E-15
CDT1	0.82	1.77	8.89E-17	5.71E-15
SUGCT	-1.22	0.43	1.09E-16	6.95E-15
RRAD	-0.99	0.50	1.12E-16	7.12E-15
ATPAF1	-0.81	0.57	1.35E-16	8.55E-15
MX2	-0.90	0.53	1.67E-16	1.05E-14
CDK9	-0.77	0.59	1.70E-16	1.06E-14
PARL	-0.87	0.55	1.75E-16	1.10E-14
HERPUD1	0.80	1.74	1.90E-16	1.18E-14
ATP6AP1	-0.63	0.65	2.06E-16	1.27E-14
CFL2	0.85	1.80	2.37E-16	1.46E-14
MORF4L1	0.55	1.47	2.44E-16	1.50E-14
CCND2	-0.73	0.60	2.58E-16	1.58E-14
IFNK	-1.08	0.47	3.07E-16	1.86E-14
ZFHX3	-0.86	0.55	3.16E-16	1.91E-14
AIM1L	-0.68	0.62	3.27E-16	1.97E-14
POLH	-0.75	0.59	3.94E-16	2.36E-14
UBE2Z	-0.60	0.66	4.36E-16	2.60E-14
KRT18	0.82	1.76	4.68E-16	2.78E-14
FAM35A	0.76	1.70	5.18E-16	3.06E-14
HSPD1	0.66	1.59	5.28E-16	3.11E-14
MMGT1	-0.81	0.57	5.64E-16	3.31E-14
CYB5R1	-0.55	0.68	8.29E-16	4.84E-14
MCAM	-0.80	0.57	1.04E-15	6.03E-14
KIF23	0.77	1.70	1.04E-15	6.03E-14
HSP90AB1	-0.65	0.64	1.23E-15	7.09E-14
RACGAP1	0.70	1.62	1.30E-15	7.47E-14
BAK1	-0.84	0.56	1.41E-15	8.08E-14
MAPK6	0.56	1.48	1.50E-15	8.55E-14
DCAF7	0.56	1.47	1.84E-15	1.04E-13
PCDHGC3	-0.87	0.55	1.90E-15	1.07E-13

CDC45	-0.65	0.64	8.73E-10	4.92E-08
MAT2A	-0.40	0.76	8.74E-10	4.92E-08
SREBF1	-0.46	0.73	9.29E-10	5.21E-08
SLC2A4RG	-0.57	0.68	9.78E-10	5.46E-08
RBL1	-0.63	0.65	1.03E-09	5.72E-08
TNFAIP1	0.41	1.33	1.13E-09	6.26E-08
PNPO	-0.63	0.65	1.17E-09	6.45E-08
LSP1	-0.87	0.55	1.24E-09	6.82E-08
DSG1	-0.52	0.70	1.29E-09	7.05E-08
SPRR2E	-0.99	0.50	1.31E-09	7.12E-08
PPFIA1	0.45	1.37	1.31E-09	7.12E-08
KREMEN1	-0.43	0.74	1.32E-09	7.12E-08
F3	0.38	1.30	1.32E-09	7.12E-08
PRSS27	-0.65	0.64	1.37E-09	7.35E-08
SCD5	0.48	1.39	1.51E-09	8.04E-08
DMKN	-0.40	0.76	1.52E-09	8.07E-08
EIF1AD	-0.65	0.64	1.62E-09	8.59E-08
PKD1	-0.63	0.64	1.66E-09	8.79E-08
FERMT1	-0.36	0.78	1.83E-09	9.61E-08
CYSRT1	-0.76	0.59	1.92E-09	1.01E-07
ESF1	0.57	1.48	2.06E-09	1.08E-07
NCF2	-0.71	0.61	2.07E-09	1.08E-07
FOS	-0.63	0.64	2.17E-09	1.12E-07
SPRR2D	-0.42	0.75	2.24E-09	1.16E-07
WWC2	0.55	1.46	2.25E-09	1.16E-07
RAET1E	-0.60	0.66	2.38E-09	1.22E-07
TCF19	-0.49	0.71	2.44E-09	1.25E-07
AP3D1	0.42	1.33	2.47E-09	1.26E-07
BHLHE40	-0.37	0.77	2.49E-09	1.26E-07
RAPGEF1	0.48	1.39	2.92E-09	1.47E-07
RP11-800A3.4	-0.56	0.68	3.03E-09	1.52E-07
MYADM	-0.54	0.69	3.10E-09	1.55E-07
SBF2	0.51	1.42	3.14E-09	1.57E-07
CHST3	-0.50	0.71	3.34E-09	1.66E-07
ACSS2	-0.47	0.72	4.00E-09	1.98E-07
GBP6	-0.55	0.68	4.04E-09	1.99E-07
DOCK5	0.44	1.35	4.10E-09	2.02E-07
EXT1	0.46	1.37	4.19E-09	2.05E-07
PRKCDBP	-0.61	0.65	4.24E-09	2.07E-07
CEP104	0.48	1.40	4.32E-09	2.10E-07
DLX3	-0.95	0.52	4.51E-09	2.18E-07
PPARGC1B	-0.73	0.60	4.52E-09	2.18E-07
STIP1	0.34	1.27	4.63E-09	2.23E-07
TAF9	0.43	1.35	5.00E-09	2.40E-07
IER2	-0.56	0.68	5.04E-09	2.41E-07
PHYHIP	-1.04	0.49	5.06E-09	2.41E-07
PTGS1	-0.76	0.59	5.17E-09	2.45E-07
KRT6C	-0.56	0.68	5.18E-09	2.45E-07
NDUFA4L2	-1.00	0.50	5.24E-09	2.47E-07
NCCRP1	-0.41	0.75	5.29E-09	2.48E-07
ETS1	0.39	1.31	5.38E-09	2.52E-07
PIP5K1A	0.41	1.33	5.43E-09	2.53E-07
UBE2G2	0.42	1.33	5.53E-09	2.57E-07
DIAPH3	0.50	1.41	5.82E-09	2.69E-07
CDC6	-0.46	0.73	5.88E-09	2.71E-07
AGFG1	0.42	1.34	6.20E-09	2.85E-07
NSUN2	0.37	1.29	6.74E-09	3.09E-07
BIRC3	0.86	1.82	7.11E-09	3.25E-07
MMP28	-0.78	0.58	7.55E-09	3.44E-07
PROSER1	0.43	1.35	7.60E-09	3.44E-07
HAS2	-1.08	0.47	7.61E-09	3.44E-07
HP1BP3	0.40	1.32	7.81E-09	3.52E-07
KRT80	-0.41	0.75	8.08E-09	3.63E-07
PTPRK	0.41	1.33	8.22E-09	3.68E-07
CRYAB	-0.73	0.60	8.44E-09	3.76E-07
LARP4	0.48	1.39	8.46E-09	3.76E-07
RBM12	0.38	1.30	8.51E-09	3.76E-07
LMTK2	0.46	1.38	8.51E-09	3.76E-07
WAC	0.38	1.30	8.96E-09	3.94E-07
GAL	-0.68	0.62	8.97E-09	3.94E-07
RELB	0.84	1.79	9.06E-09	3.97E-07
ACOT11	-0.71	0.61	9.52E-09	4.15E-07
SF1	0.46	1.37	9.55E-09	4.15E-07
CSNK1E	0.44	1.35	9.57E-09	4.15E-07
TFRC	0.44	1.35	9.62E-09	4.16E-07
CITED4	-0.58	0.67	9.72E-09	4.19E-07
FBXO27	-0.59	0.66	1.00E-08	4.31E-07
POLR3E	0.47	1.39	1.01E-08	4.33E-07
PTTG1	0.38	1.31	1.02E-08	4.34E-07
PTPN22	-1.06	0.48	1.03E-08	4.38E-07
BCR	0.42	1.34	1.03E-08	4.38E-07
HBEGF	0.65	1.57	1.04E-08	4.39E-07
UBE3C	0.34	1.27	1.05E-08	4.42E-07
CLCA2	-0.43	0.74	1.07E-08	4.48E-07
GLUL	-0.34	0.79	1.12E-08	4.68E-07
COL4A5	0.43	1.35	1.15E-08	4.80E-07
SLC20A2	0.34	1.27	1.17E-08	4.89E-07
ZNF436	-0.58	0.67	1.28E-08	5.30E-07
RAPGEFL1	-0.45	0.73	1.30E-08	5.37E-07
RHCG	-0.38	0.77	1.30E-08	5.38E-07
RTKN	-0.61	0.65	1.93E-15	1.08E-13
FLRT3	-0.59	0.67	1.93E-15	1.08E-13
NR1D1	0.93	1.91	2.13E-15	1.18E-13
IQCE	-0.84	0.56	2.65E-15	1.47E-13
GOLM1	0.73	1.66	2.69E-15	1.49E-13
KAT6A	-0.69	0.62	2.88E-15	1.59E-13
COL4A1	0.66	1.58	3.25E-15	1.78E-13
SPAG5	0.82	1.77	3.45E-15	1.88E-13
ATP6V0D1	-0.64	0.64	3.81E-15	2.07E-13
CLEC16A	-0.66	0.63	3.89E-15	2.11E-13
TYMP	-0.59	0.66	5.59E-15	3.02E-13
STAT2	-0.58	0.67	8.22E-15	4.42E-13
KIAA1217	-0.67	0.63	9.27E-15	4.94E-13
LMNB1	0.85	1.80	9.27E-15	4.94E-13
KRT81	0.97	1.96	1.02E-14	5.41E-13
PPM1F	-0.63	0.64	1.09E-14	5.77E-13
NOA1	-0.81	0.57	1.12E-14	5.87E-13
CCNG1	0.60	1.52	1.12E-14	5.87E-13
BUB1B	0.76	1.69	1.19E-14	6.22E-13
FILIP1L	-0.84	0.56	1.20E-14	6.27E-13
EEF2K	-0.81	0.57	1.26E-14	6.53E-13
SLC2A1	-0.48	0.71	1.28E-14	6.63E-13
APOBEC3C	-0.62	0.65	1.42E-14	7.30E-13
SIPA1L3	-0.78	0.58	1.45E-14	7.43E-13
CCDC120	-0.67	0.63	1.56E-14	7.98E-13
TIMP2	-1.09	0.47	1.58E-14	8.06E-13
RGS2	-0.89	0.54	1.64E-14	8.31E-13
GRHL3	-0.60	0.66	1.66E-14	8.37E-13
SLC7A5	-0.72	0.61	1.86E-14	9.34E-13
SLC29A1	0.65	1.57	2.07E-14	1.04E-12
ATP11A	-0.70	0.62	2.09E-14	1.04E-12
SERTAD2	-0.77	0.59	2.15E-14	1.07E-12
ANP32E	0.70	1.62	2.19E-14	1.09E-12
EPCAM	0.99	1.99	2.44E-14	1.20E-12
SPRYD4	-1.28	0.41	2.68E-14	1.32E-12
PCBD1	-0.76	0.59	2.83E-14	1.39E-12
TOP2A	0.74	1.66	2.86E-14	1.40E-12
MECP2	-0.76	0.59	2.94E-14	1.43E-12
DLGAP5	0.79	1.73	2.95E-14	1.43E-12
CCNB2	0.76	1.70	3.16E-14	1.52E-12
EVI5L	-0.67	0.63	3.16E-14	1.52E-12
PHLDA1	-0.61	0.66	4.00E-14	1.92E-12
CKS2	0.68	1.60	4.44E-14	2.12E-12
ACP2	-1.01	0.50	4.61E-14	2.20E-12
KRT77	-1.68	0.31	5.65E-14	2.68E-12
KIRREL	-0.73	0.60	5.85E-14	2.77E-12
AXL	0.76	1.69	5.90E-14	2.78E-12
PPIF	0.64	1.56	6.12E-14	2.88E-12
AURKA	0.80	1.74	6.19E-14	2.90E-12
GM2A	-0.57	0.68	6.50E-14	3.03E-12
SPON2	1.04	2.06	6.95E-14	3.23E-12
EPHA4	-0.83	0.56	7.06E-14	3.27E-12
EPB41L2	-0.64	0.64	7.45E-14	3.43E-12
HERC6	-0.59	0.67	7.46E-14	3.43E-12
STMN1	0.66	1.57	7.53E-14	3.46E-12
KPNA2	0.69	1.61	7.68E-14	3.51E-12
HECTD3	-0.69	0.62	1.07E-13	4.85E-12
PPP2R1B	0.58	1.49	1.07E-13	4.87E-12
NDST1	-0.54	0.69	1.26E-13	5.72E-12
CCDC117	0.76	1.69	1.28E-13	5.77E-12
SH3PXD2A	-0.56	0.68	1.32E-13	5.91E-12
NEMP1	0.67	1.59	1.35E-13	6.06E-12
NOTCH1	-0.60	0.66	1.38E-13	6.18E-12
NRG1	-0.75	0.60	1.43E-13	6.38E-12
DUSP2	1.29	2.44	1.45E-13	6.41E-12
SLC25A22	-0.99	0.50	1.48E-13	6.55E-12
RETSAT	-0.56	0.68	1.70E-13	7.49E-12
RNF38	0.73	1.66	1.82E-13	7.98E-12
ADGRL2	-0.65	0.64	1.91E-13	8.35E-12
HMGB1	0.59	1.51	2.04E-13	8.90E-12
HSP90AA1	0.59	1.51	2.15E-13	9.35E-12
MIR22HG	-0.78	0.58	2.26E-13	9.82E-12
UBE2C	0.75	1.68	2.34E-13	1.01E-11
SPATA18	-0.82	0.57	2.49E-13	1.07E-11
TNPO1	0.51	1.42	2.86E-13	1.23E-11
DUOX1	-0.57	0.67	2.87E-13	1.23E-11
TMED5	0.59	1.50	2.97E-13	1.27E-11
SPDL1	0.82	1.76	3.00E-13	1.28E-11
ATP2B4	-0.53	0.69	3.08E-13	1.31E-11
MACC1	-0.58	0.67	3.66E-13	1.55E-11
AP3S2	-0.96	0.51	3.95E-13	1.67E-11
RND3	-0.52	0.70	4.28E-13	1.80E-11
POLQ	0.83	1.77	4.66E-13	1.95E-11
PTPRS	0.60	1.52	4.88E-13	2.04E-11
RCC2	-0.51	0.70	4.91E-13	2.05E-11
NAB1	-0.59	0.67	5.11E-13	2.12E-11
SAMD4B	-0.74	0.60	5.27E-13	2.18E-11
SPOCK1	1.15	2.21	5.74E-13	2.37E-11
SLC16A3	1.03	2.04	6.32E-13	2.60E-11
PTP4A2	0.54	1.45	6.35E-13	2.60E-11

MX1	-0.54	0.69	1.32E-08	5.42E-07
XPR1	0.48	1.40	1.34E-08	5.49E-07
AIF1L	-0.60	0.66	1.35E-08	5.51E-07
AHCTF1	0.47	1.39	1.36E-08	5.55E-07
INADL	0.44	1.36	1.41E-08	5.72E-07
GPX2	-0.79	0.58	1.45E-08	5.88E-07
SLC39A14	0.54	1.45	1.50E-08	6.08E-07
METTL7A	-0.79	0.58	1.53E-08	6.18E-07
PPP4R2	0.58	1.50	1.58E-08	6.33E-07
CDIP1	-0.53	0.69	1.60E-08	6.40E-07
P3H4	-0.75	0.59	1.67E-08	6.68E-07
MMRN2	-0.75	0.60	1.73E-08	6.89E-07
RMI2	-0.79	0.58	1.80E-08	7.13E-07
PNISR	0.52	1.44	1.80E-08	7.13E-07
PIK3C2B	-0.67	0.63	1.81E-08	7.13E-07
INTS7	0.51	1.42	1.84E-08	7.23E-07
IFFO2	-0.38	0.77	1.86E-08	7.29E-07
PAQR4	-0.65	0.64	1.87E-08	7.33E-07
CCDC57	0.58	1.50	1.96E-08	7.63E-07
RBBP6	0.47	1.38	2.11E-08	8.19E-07
PKP4	0.40	1.32	2.14E-08	8.29E-07
ZNF672	-0.61	0.66	2.17E-08	8.39E-07
IRF2BPL	-0.58	0.67	2.20E-08	8.47E-07
LRRCC1	-0.79	0.58	2.20E-08	8.47E-07
OAS1	-0.64	0.64	2.25E-08	8.64E-07
TROAP	0.53	1.44	2.29E-08	8.77E-07
NOTCH1	-0.46	0.73	2.42E-08	9.21E-07
CXCL1	0.69	1.62	2.64E-08	1.00E-06
RRM1	-0.36	0.78	2.68E-08	1.02E-06
LMNB1	-0.46	0.73	2.71E-08	1.03E-06
KIF20B	0.54	1.46	2.74E-08	1.03E-06
MFHAS1	0.51	1.43	2.75E-08	1.03E-06
OSBPL10	0.39	1.31	2.82E-08	1.06E-06
RXRA	-0.42	0.75	2.83E-08	1.06E-06
UAP1L1	-0.76	0.59	2.96E-08	1.10E-06
USP10	0.37	1.29	2.99E-08	1.11E-06
CSPG4	-1.00	0.50	3.02E-08	1.12E-06
LAMA3	0.31	1.24	3.06E-08	1.13E-06
MALL	-0.60	0.66	3.11E-08	1.15E-06
EPHA2	0.44	1.35	3.18E-08	1.17E-06
EGR1	-0.53	0.69	3.19E-08	1.17E-06
TYMS	-0.57	0.67	3.25E-08	1.19E-06
CPSF6	0.38	1.30	3.29E-08	1.20E-06
MEOX1	-0.96	0.51	3.31E-08	1.20E-06
ADIPOR1	0.32	1.25	3.35E-08	1.22E-06
FOSL1	0.44	1.35	3.40E-08	1.23E-06
UHRF1	-0.49	0.71	3.49E-08	1.26E-06
DEPDC1	0.56	1.48	3.54E-08	1.28E-06
IL18	0.45	1.36	3.70E-08	1.33E-06
CSNK2A2	0.39	1.31	3.73E-08	1.33E-06
SERPINB3	-0.59	0.66	3.90E-08	1.39E-06
TICRR	-0.54	0.69	3.90E-08	1.39E-06
HPDL	-1.01	0.50	4.06E-08	1.44E-06
RBM33	0.45	1.37	4.13E-08	1.46E-06
METRNL	-0.55	0.68	4.34E-08	1.53E-06
ATAD3B	0.46	1.38	4.34E-08	1.53E-06
ZNF207	0.36	1.29	4.69E-08	1.65E-06
TP53AIP1	-0.89	0.54	4.87E-08	1.71E-06
GTPBP4	0.37	1.29	5.02E-08	1.76E-06
NRM	-0.64	0.64	5.09E-08	1.78E-06
KRT31	-0.82	0.57	5.27E-08	1.83E-06
LARP4B	0.37	1.30	5.35E-08	1.86E-06
NPLOC4	0.36	1.29	6.00E-08	2.08E-06
ZFC3H1	0.55	1.46	6.03E-08	2.08E-06
KRT1	-0.52	0.70	6.26E-08	2.16E-06
SETD1B	-0.53	0.69	6.28E-08	2.16E-06
FXYD3	-0.35	0.79	6.62E-08	2.27E-06
MAPRE1	0.37	1.29	6.71E-08	2.29E-06
TMPRSS4	-0.58	0.67	6.73E-08	2.29E-06
FZR1	-0.45	0.73	6.91E-08	2.35E-06
PTK2	0.36	1.28	7.05E-08	2.39E-06
IVNS1ABP	-0.41	0.75	7.09E-08	2.40E-06
SULF2	-0.33	0.80	7.13E-08	2.40E-06
PBX1	-0.66	0.63	7.14E-08	2.40E-06
PVRL4	-0.42	0.75	7.38E-08	2.48E-06
C10orf54	-0.49	0.71	7.42E-08	2.49E-06
QKI	0.41	1.32	7.51E-08	2.51E-06
BIRC5	0.48	1.40	7.59E-08	2.53E-06
HSPH1	0.40	1.32	7.66E-08	2.55E-06
LGALS7	-0.58	0.67	7.88E-08	2.61E-06
BCAR3	0.41	1.33	7.89E-08	2.61E-06
EPS8L1	-0.42	0.75	7.93E-08	2.62E-06
FAM111A	-0.57	0.67	8.10E-08	2.67E-06
GDPD2	-0.95	0.52	8.19E-08	2.69E-06
FAM53C	0.45	1.37	8.76E-08	2.87E-06
MIR205HG	-0.41	0.75	8.82E-08	2.88E-06
GAL3ST4	-0.91	0.53	8.98E-08	2.93E-06
ARPIN	-0.56	0.68	9.03E-08	2.94E-06
ITCH	0.42	1.34	9.19E-08	2.98E-06
IFITM3	-0.41	0.75	9.24E-08	2.99E-06

ZBTB45	-0.89	0.54	6.58E-13	2.69E-11
RPS27L	-0.67	0.63	6.77E-13	2.76E-11
ARHGEF3	-0.60	0.66	6.93E-13	2.81E-11
TUBA1C	0.56	1.47	7.23E-13	2.93E-11
KRT8	0.49	1.40	7.44E-13	3.01E-11
ABAT	-0.76	0.59	7.67E-13	3.09E-11
NACC2	0.65	1.57	8.34E-13	3.35E-11
KIF11	0.64	1.56	8.41E-13	3.37E-11
SMS	0.59	1.51	8.51E-13	3.40E-11
SEC24C	-0.58	0.67	8.63E-13	3.43E-11
VPS9D1-AS1	-1.43	0.37	9.90E-13	3.93E-11
WNT10A	0.56	1.47	1.01E-12	4.01E-11
PLAGL2	-0.63	0.65	1.10E-12	4.34E-11
CDC6	0.66	1.59	1.15E-12	4.53E-11
CDKN1B	0.67	1.59	1.19E-12	4.68E-11
MEN1	-0.69	0.62	1.36E-12	5.30E-11
BTBD19	-1.34	0.40	1.38E-12	5.40E-11
MTF2	0.70	1.63	1.41E-12	5.49E-11
F3	-0.54	0.69	1.44E-12	5.57E-11
CES2	-0.61	0.66	1.46E-12	5.64E-11
GPR1	-0.85	0.55	1.52E-12	5.87E-11
IGFL2	-0.80	0.57	1.57E-12	6.04E-11
MAML1	-0.77	0.59	1.62E-12	6.21E-11
ASB13	0.66	1.58	1.66E-12	6.35E-11
BCL9L	-0.64	0.64	1.72E-12	6.55E-11
DYRK1B	-1.13	0.46	1.73E-12	6.57E-11
COPS7A	-0.63	0.65	1.77E-12	6.70E-11
CBX5	0.50	1.42	1.78E-12	6.74E-11
MICAL3	-0.54	0.69	1.82E-12	6.86E-11
MOSPD3	-1.13	0.46	1.83E-12	6.86E-11
NDC80	0.84	1.79	2.03E-12	7.59E-11
SMIM10L2A	-1.60	0.33	2.11E-12	7.88E-11
MCFD2	0.53	1.45	2.12E-12	7.90E-11
GHDC	-1.19	0.44	2.13E-12	7.90E-11
IFFO2	-0.56	0.68	2.17E-12	8.05E-11
SLC44A2	-0.46	0.73	2.25E-12	8.31E-11
RABL6	-0.63	0.65	2.43E-12	8.97E-11
LENG8	-0.53	0.69	2.46E-12	9.03E-11
ARHGEF16	0.65	1.56	2.59E-12	9.49E-11
KDM2B	-0.66	0.63	2.63E-12	9.60E-11
PSRC1	0.85	1.80	2.68E-12	9.77E-11
TPX2	0.67	1.59	2.77E-12	1.01E-10
MYH9	-0.54	0.69	2.79E-12	1.01E-10
DNAJC5	-0.60	0.66	2.84E-12	1.03E-10
CIC	-0.58	0.67	2.88E-12	1.04E-10
TPM2	0.58	1.49	2.93E-12	1.05E-10
SERPINB7	-0.47	0.72	3.10E-12	1.11E-10
KIF22	0.59	1.51	3.42E-12	1.22E-10
DNMT3B	0.85	1.80	4.02E-12	1.44E-10
RERE	-0.61	0.66	4.09E-12	1.46E-10
SAT1	-0.53	0.69	4.20E-12	1.49E-10
ENTPD7	0.69	1.61	4.35E-12	1.54E-10
NCEH1	0.64	1.55	4.44E-12	1.57E-10
PHLPP2	0.82	1.76	4.45E-12	1.57E-10
FBXL12	-0.85	0.55	4.90E-12	1.72E-10
F11R	-0.50	0.71	4.95E-12	1.73E-10
MAP3K3	-0.72	0.61	4.95E-12	1.73E-10
IKZF2	-0.69	0.62	4.99E-12	1.74E-10
DIXDC1	0.63	1.55	5.09E-12	1.77E-10
TTYH3	-0.53	0.69	5.78E-12	2.00E-10
GATAD2A	-0.62	0.65	5.94E-12	2.05E-10
DUSP3	-0.63	0.65	6.32E-12	2.18E-10
ZMAT3	-0.79	0.58	6.78E-12	2.33E-10
GRN	-0.46	0.73	7.12E-12	2.44E-10
KLK10	-0.53	0.69	7.34E-12	2.51E-10
LPCAT4	-0.58	0.67	7.44E-12	2.54E-10
NELFCD	-0.53	0.69	7.79E-12	2.65E-10
NYNRIN	-0.62	0.65	8.05E-12	2.74E-10
THUMPD1	0.56	1.47	8.43E-12	2.86E-10
NIPAL4	-0.53	0.69	8.81E-12	2.98E-10
EIF4EBP2	0.50	1.42	9.45E-12	3.19E-10
TACC3	0.66	1.58	9.73E-12	3.27E-10
MRPS11	-0.90	0.54	1.02E-11	3.41E-10
ATP11C	0.68	1.60	1.05E-11	3.52E-10
CARNMT1	0.63	1.55	1.11E-11	3.71E-10
TAB3	-0.57	0.68	1.17E-11	3.88E-10
KDM6B	-0.62	0.65	1.23E-11	4.09E-10
FERMT2	0.76	1.70	1.24E-11	4.10E-10
CHFR	-0.67	0.63	1.26E-11	4.16E-10
TGM1	-0.55	0.68	1.26E-11	4.16E-10
STRADB	0.83	1.78	1.27E-11	4.19E-10
ECT2	0.56	1.47	1.30E-11	4.27E-10
SART1	-0.57	0.68	1.35E-11	4.43E-10
ALS2CL	-0.53	0.69	1.51E-11	4.92E-10
DOCK5	-0.53	0.69	1.54E-11	5.01E-10
C16orf70	-0.68	0.63	1.57E-11	5.10E-10
PTPRZ1	-0.55	0.68	1.64E-11	5.31E-10
VAV3	0.54	1.45	1.69E-11	5.46E-10
LAMC1	0.44	1.36	1.77E-11	5.72E-10
GMFB	-0.56	0.68	1.90E-11	6.13E-10

ZC3H12C	0.63	1.55	9.25E-08	2.99E-06
RIN1	-0.45	0.73	9.61E-08	3.10E-06
STK10	0.42	1.34	1.02E-07	3.27E-06
CERS6	0.49	1.41	1.04E-07	3.34E-06
MT-CO3	0.44	1.36	1.04E-07	3.34E-06
KSR1	-0.69	0.62	1.10E-07	3.51E-06
ATP1B1	-0.49	0.71	1.13E-07	3.60E-06
KCNN4	-0.71	0.61	1.13E-07	3.60E-06
MT-ND3	0.48	1.40	1.14E-07	3.60E-06
CDC42EP4	-0.43	0.74	1.20E-07	3.78E-06
MALAT1	0.90	1.87	1.20E-07	3.78E-06
CBX4	-0.67	0.63	1.23E-07	3.86E-06
SUGP2	0.42	1.34	1.24E-07	3.89E-06
PRIM1	-0.64	0.64	1.24E-07	3.89E-06
PSME4	0.41	1.33	1.25E-07	3.90E-06
INSIG1	-0.46	0.73	1.30E-07	4.06E-06
TMEM184B	0.42	1.34	1.32E-07	4.10E-06
LDOC1L	-0.57	0.67	1.33E-07	4.13E-06
CDH16	-0.95	0.52	1.39E-07	4.31E-06
PRRC2C	0.31	1.24	1.42E-07	4.39E-06
PICALM	0.34	1.27	1.46E-07	4.52E-06
CEP128	0.61	1.53	1.48E-07	4.54E-06
SCD	-0.43	0.74	1.53E-07	4.71E-06
NEFL	-0.95	0.52	1.59E-07	4.86E-06
BDH1	-0.44	0.74	1.59E-07	4.86E-06
CNN3	0.38	1.31	1.62E-07	4.95E-06
MT-CO2	0.45	1.37	1.65E-07	5.01E-06
KLK13	-0.45	0.73	1.68E-07	5.09E-06
XYLB	-0.85	0.56	1.72E-07	5.22E-06
DENND5A	0.49	1.40	1.73E-07	5.23E-06
MAP3K1	-0.62	0.65	1.83E-07	5.52E-06
CES2	-0.37	0.77	1.85E-07	5.58E-06
KIF4A	0.41	1.33	1.92E-07	5.77E-06
PHGDH	-0.39	0.76	1.96E-07	5.87E-06
KDELR3	-0.73	0.60	2.00E-07	5.98E-06
ARHGEF11	-0.51	0.70	2.05E-07	6.10E-06
CTGF	0.64	1.56	2.06E-07	6.13E-06
GTPBP3	-0.65	0.64	2.13E-07	6.33E-06
LYPD5	-0.77	0.59	2.16E-07	6.39E-06
VANGL2	-0.58	0.67	2.19E-07	6.49E-06
P2RY2	-0.47	0.72	2.26E-07	6.66E-06
LRRC45	-0.61	0.65	2.29E-07	6.73E-06
DIXDC1	-0.76	0.59	2.40E-07	7.05E-06
CYP2W1	-0.84	0.56	2.41E-07	7.05E-06
MROH6	-0.50	0.71	2.41E-07	7.05E-06
TRIM7	-0.54	0.69	2.43E-07	7.09E-06
SRRM2	0.30	1.23	2.47E-07	7.21E-06
DLX1	-0.85	0.55	2.50E-07	7.28E-06
MMP9	-0.53	0.69	2.56E-07	7.41E-06
TINAGL1	0.37	1.29	2.58E-07	7.46E-06
CXCL8	0.86	1.82	2.58E-07	7.46E-06
SLC1A4	-0.80	0.57	2.64E-07	7.60E-06
UBQLN1	0.30	1.23	2.65E-07	7.63E-06
TRIM22	-0.55	0.68	2.72E-07	7.81E-06
FAM208B	0.38	1.30	2.78E-07	7.95E-06
ANLN	0.34	1.27	2.81E-07	8.03E-06
CTNNB1	0.32	1.25	2.89E-07	8.23E-06
FOXN1	-0.76	0.59	2.91E-07	8.27E-06
USP37	-0.69	0.62	2.91E-07	8.27E-06
SPDL1	0.46	1.37	3.04E-07	8.61E-06
RBM6	0.44	1.35	3.07E-07	8.68E-06
C19orf43	-0.48	0.72	3.14E-07	8.87E-06
CRIM1	0.33	1.26	3.25E-07	9.14E-06
BMS1	0.37	1.29	3.27E-07	9.20E-06
EIF3B	0.30	1.23	3.33E-07	9.33E-06
CDKN2C	-0.86	0.55	3.35E-07	9.34E-06
ACTG1	0.35	1.28	3.35E-07	9.34E-06
UAP1	0.36	1.29	3.35E-07	9.34E-06
ZC3H12A	0.52	1.44	3.39E-07	9.42E-06
MED15	0.40	1.32	3.39E-07	9.42E-06
TNFRSF10B	0.35	1.27	3.48E-07	9.64E-06
ACLY	-0.31	0.81	3.50E-07	9.67E-06
CHMP1B	0.38	1.31	3.60E-07	9.92E-06
KLK10	-0.40	0.76	3.61E-07	9.93E-06
TMEM86A	-0.61	0.65	3.68E-07	1.01E-05
AHDC1	-0.49	0.71	3.71E-07	1.02E-05
PIGR	-0.80	0.57	3.72E-07	1.02E-05
SPINK6	-0.35	0.78	3.89E-07	1.06E-05
MBNL3	-0.57	0.67	4.01E-07	1.09E-05
AKIRIN1	0.40	1.32	4.04E-07	1.10E-05
ZNF395	-0.44	0.74	4.29E-07	1.16E-05
SNAI2	-0.36	0.78	4.31E-07	1.17E-05
IDH2	-0.35	0.78	4.33E-07	1.17E-05
C1orf74	0.36	1.28	4.38E-07	1.18E-05
CDYL	0.41	1.33	4.74E-07	1.28E-05
CDH3	0.32	1.25	4.80E-07	1.29E-05
SLC25A1	-0.43	0.74	4.84E-07	1.30E-05
RAET1L	-0.46	0.73	5.05E-07	1.35E-05
AMMECR1L	0.40	1.32	5.08E-07	1.36E-05
POU3F1	-0.92	0.53	5.23E-07	1.39E-05
MAST2	-0.58	0.67	1.93E-11	6.19E-10
PRC1	0.64	1.56	1.95E-11	6.23E-10
MGRN1	-0.66	0.63	1.95E-11	6.23E-10
FZD10	1.08	2.11	2.03E-11	6.48E-10
MIR503HG	-0.68	0.62	2.08E-11	6.60E-10
LAD1	-1.33	0.40	2.08E-11	6.60E-10
HN1	0.46	1.37	2.09E-11	6.62E-10
HIPK3	0.52	1.43	2.21E-11	6.98E-10
FAT2	0.45	1.37	2.24E-11	7.05E-10
C2CD2	0.61	1.52	2.25E-11	7.06E-10
FAT1	-0.43	0.74	2.27E-11	7.11E-10
PBK	0.68	1.61	2.37E-11	7.42E-10
H2AFZ	0.60	1.52	2.53E-11	7.91E-10
DOCK11	1.12	2.17	2.60E-11	8.10E-10
ATG13	-0.62	0.65	2.71E-11	8.43E-10
MALAT1	-0.47	0.72	2.76E-11	8.55E-10
KCTD20	0.51	1.42	3.16E-11	9.76E-10
UPK1B	-0.52	0.70	3.17E-11	9.77E-10
WDR63	-1.29	0.41	3.60E-11	1.11E-09
KLHDC3	0.53	1.44	4.00E-11	1.23E-09
NEK2	0.74	1.67	4.15E-11	1.27E-09
BPNT1	-0.64	0.64	4.29E-11	1.31E-09
KIFC2	-0.65	0.64	4.32E-11	1.32E-09
PCNXL3	-0.60	0.66	4.37E-11	1.33E-09
DQX1	-1.15	0.45	4.45E-11	1.35E-09
CENPE	0.64	1.55	4.68E-11	1.42E-09
NCKAP5L	-0.65	0.64	4.71E-11	1.42E-09
C19orf70	-0.68	0.63	5.04E-11	1.52E-09
TFAM	0.56	1.47	5.48E-11	1.65E-09
PLK1	0.70	1.63	5.53E-11	1.66E-09
DDA1	-0.59	0.67	5.73E-11	1.72E-09
LHFP	-0.92	0.53	5.78E-11	1.73E-09
ARL2	-0.89	0.54	5.85E-11	1.75E-09
PTPN21	-0.66	0.63	6.11E-11	1.82E-09
ZNF267	0.82	1.76	6.74E-11	2.00E-09
FAM160B2	-0.64	0.64	6.84E-11	2.03E-09
INPPL1	-0.44	0.74	6.88E-11	2.04E-09
OS9	-0.47	0.72	7.39E-11	2.18E-09
CANX	0.41	1.33	7.68E-11	2.26E-09
MAP2	-0.52	0.70	7.69E-11	2.26E-09
MPV17L2	-0.81	0.57	7.85E-11	2.30E-09
PPP1R18	-0.55	0.68	7.95E-11	2.32E-09
PRR14	-0.73	0.60	8.33E-11	2.43E-09
FKBP5	0.46	1.37	8.39E-11	2.44E-09
SULT2B1	-0.70	0.61	8.43E-11	2.45E-09
NCLN	-0.56	0.68	8.93E-11	2.59E-09
HMGN2	0.52	1.44	9.51E-11	2.75E-09
UBE2K	0.47	1.39	1.05E-10	3.04E-09
RCOR1	0.45	1.37	1.10E-10	3.16E-09
GPCPD1	0.60	1.51	1.11E-10	3.19E-09
NADSYN1	-0.51	0.70	1.24E-10	3.56E-09
PTTG1	0.55	1.47	1.28E-10	3.67E-09
TOP1	0.46	1.37	1.40E-10	3.99E-09
L1CAM	0.73	1.66	1.42E-10	4.04E-09
PRRC2A	-0.49	0.71	1.42E-10	4.04E-09
NKIRAS2	-0.66	0.63	1.49E-10	4.24E-09
ZNF165	1.03	2.05	1.54E-10	4.35E-09
AP2B1	0.49	1.40	1.63E-10	4.60E-09
NUSAP1	0.64	1.56	1.65E-10	4.65E-09
DPM2	-0.66	0.63	1.93E-10	5.42E-09
MAP2K3	-0.65	0.64	2.00E-10	5.62E-09
NUFIP2	0.43	1.35	2.02E-10	5.66E-09
CDH3	-0.43	0.74	2.02E-10	5.66E-09
SNRPB2	0.58	1.49	2.09E-10	5.85E-09
ERBB3	0.52	1.44	2.13E-10	5.93E-09
FAM213B	-0.91	0.53	2.16E-10	6.01E-09
SHANK3	-0.66	0.63	2.21E-10	6.14E-09
SPR	-0.72	0.61	2.22E-10	6.15E-09
AC006262.4	-1.19	0.44	2.24E-10	6.20E-09
HNRNPA3	0.41	1.33	2.29E-10	6.31E-09
KRT6C	-0.47	0.72	2.37E-10	6.52E-09
CCNA2	0.66	1.57	2.38E-10	6.53E-09
RP11-67L2.2	-1.06	0.48	2.53E-10	6.93E-09
TUBA1B	-0.56	0.68	2.53E-10	6.93E-09
MMP15	-0.73	0.60	2.72E-10	7.44E-09
PPAT	0.63	1.55	2.84E-10	7.74E-09
TES	-0.47	0.72	2.92E-10	7.95E-09
PSIP1	0.64	1.55	2.94E-10	7.99E-09
HDAC5	-0.81	0.57	3.01E-10	8.15E-09
SLC43A2	-0.72	0.61	3.08E-10	8.33E-09
BCAM	0.42	1.34	3.28E-10	8.84E-09
CASP2	-0.57	0.67	3.39E-10	9.13E-09
FAM49B	0.51	1.43	3.42E-10	9.18E-09
TMEM131	-0.48	0.72	3.43E-10	9.20E-09
APPL2	-0.62	0.65	3.68E-10	9.86E-09
EPHB2	0.96	1.94	3.75E-10	1.00E-08
PAQR7	-0.50	0.70	3.90E-10	1.04E-08
TOMM34	0.54	1.45	4.19E-10	1.11E-08
DENND2D	-0.77	0.59	4.22E-10	1.12E-08
DUSP14	-0.49	0.71	4.35E-10	1.15E-08

**SFPQ**	0.30	1.24	5.38E-07	1.43E-05	**LRFN3**	-0.95	0.52	4.56E-10	1.21E-08
**AKR1B10**	-0.89	0.54	5.52E-07	1.47E-05	**SSH2**	-0.60	0.66	4.62E-10	1.22E-08
**BMP6**	-0.54	0.69	5.59E-07	1.48E-05	**HJURP**	0.71	1.64	4.70E-10	1.24E-08
**PLCD1**	-0.33	0.80	5.75E-07	1.52E-05	**MBOAT1**	0.96	1.95	4.85E-10	1.27E-08
**ALDH1L2**	-0.59	0.67	5.79E-07	1.53E-05	**ZNF561**	-0.52	0.70	4.88E-10	1.28E-08
**TRIM65**	-0.54	0.69	5.86E-07	1.54E-05	**DCAF11**	-0.52	0.70	5.27E-10	1.38E-08
**SLC16A13**	-0.79	0.58	5.98E-07	1.57E-05	**LMO7**	-0.63	0.65	5.90E-10	1.54E-08
**NCOA6**	0.36	1.28	6.04E-07	1.58E-05	**MARCKS**	0.45	1.37	6.46E-10	1.68E-08
**NPC1**	0.36	1.28	6.08E-07	1.59E-05	**PTGES3**	0.43	1.34	6.47E-10	1.68E-08
**LPAR5**	-0.52	0.70	6.08E-07	1.59E-05	**IRF7**	-0.58	0.67	6.47E-10	1.68E-08
**DDX5**	0.29	1.22	6.12E-07	1.60E-05	**KIFC1**	0.59	1.51	6.51E-10	1.69E-08
**CCDC6**	0.39	1.31	6.12E-07	1.60E-05	**NXN**	-0.42	0.75	7.22E-10	1.87E-08
**DGKD**	0.61	1.53	6.20E-07	1.61E-05	**KIF20B**	0.54	1.45	7.48E-10	1.93E-08
**SLC9A1**	0.46	1.37	6.27E-07	1.63E-05	**ATMIN**	-0.50	0.71	7.54E-10	1.95E-08
**POLH**	-0.45	0.73	6.49E-07	1.68E-05	**KIF4A**	0.63	1.55	7.64E-10	1.97E-08
**CAMSAP2**	0.36	1.28	6.64E-07	1.72E-05	**RP5-991G20.1**	-1.17	0.45	7.78E-10	2.00E-08
**PLK1**	0.37	1.29	6.66E-07	1.72E-05	**ZYG11B**	-0.55	0.68	7.82E-10	2.01E-08
**MSH6**	-0.38	0.77	6.82E-07	1.75E-05	**COQ10B**	-0.74	0.60	8.18E-10	2.09E-08
**CRNN**	-0.77	0.59	6.83E-07	1.75E-05	**GNS**	0.43	1.34	8.37E-10	2.14E-08
**ZRANB2**	0.47	1.39	6.85E-07	1.76E-05	**SERPINB5**	-0.38	0.77	8.78E-10	2.24E-08
**NUP98**	0.31	1.24	6.88E-07	1.76E-05	**PSMF1**	-0.54	0.69	8.80E-10	2.24E-08
**CAV2**	0.37	1.30	6.91E-07	1.77E-05	**HNRNPA2B1**	0.41	1.33	8.81E-10	2.24E-08
**GDI1**	0.37	1.29	7.10E-07	1.81E-05	**CBX1**	0.49	1.40	8.99E-10	2.28E-08
**PHF20**	0.40	1.32	7.12E-07	1.81E-05	**RBSN**	-0.53	0.69	9.00E-10	2.28E-08
**HSPB8**	-0.43	0.74	7.20E-07	1.83E-05	**CDKN1A**	-1.22	0.43	9.12E-10	2.30E-08
**RARG**	-0.35	0.79	7.23E-07	1.83E-05	**CDK1**	0.56	1.48	9.16E-10	2.31E-08
**LINC00854**	0.81	1.75	7.25E-07	1.84E-05	**ODC1**	0.57	1.48	9.20E-10	2.31E-08
**E2F3**	0.50	1.41	7.41E-07	1.87E-05	**MOB3B**	-0.44	0.74	9.24E-10	2.32E-08
**ZFAND3**	0.37	1.30	7.43E-07	1.87E-05	**ABHD15**	-0.91	0.53	9.34E-10	2.34E-08
**BBOX1**	-0.79	0.58	7.77E-07	1.96E-05	**EIF3J**	0.45	1.36	9.67E-10	2.42E-08
**SERPINB2**	-0.29	0.82	7.79E-07	1.96E-05	**CTDSPL**	-0.46	0.73	9.85E-10	2.46E-08
**ASIC1**	-0.75	0.60	7.83E-07	1.96E-05	**SMC4**	0.56	1.47	9.97E-10	2.48E-08
**CHCHD10**	-0.62	0.65	7.88E-07	1.97E-05	**WBP1**	-0.99	0.50	1.02E-09	2.54E-08
**DDX10**	0.43	1.35	7.96E-07	1.99E-05	**WIPI2**	-0.50	0.71	1.03E-09	2.57E-08
**PLA2G3**	-0.81	0.57	8.09E-07	2.02E-05	**FNDC3A**	0.49	1.40	1.08E-09	2.67E-08
**NFKB2**	0.43	1.34	8.16E-07	2.03E-05	**NUDC**	0.43	1.35	1.09E-09	2.69E-08
**IL36RN**	-0.47	0.72	8.31E-07	2.07E-05	**TINAGL1**	-0.52	0.70	1.09E-09	2.69E-08
**SLC4A7**	0.52	1.44	8.53E-07	2.12E-05	**COX6A1**	-0.64	0.64	1.11E-09	2.75E-08
**ADGRA3**	0.39	1.31	8.66E-07	2.14E-05	**IGF2BP3**	0.56	1.47	1.18E-09	2.90E-08
**PCNT**	0.39	1.31	8.76E-07	2.16E-05	**RTN4RL1**	0.61	1.52	1.24E-09	3.05E-08
**HES4**	-0.75	0.59	8.77E-07	2.16E-05	**GPAT4**	0.53	1.45	1.26E-09	3.09E-08
**CALML3**	-0.73	0.60	9.18E-07	2.26E-05	**ARPIN**	-0.55	0.68	1.26E-09	3.09E-08
**LRBA**	0.34	1.27	9.48E-07	2.33E-05	**DGCR6L**	-0.68	0.63	1.30E-09	3.17E-08
**AMER1**	-0.65	0.64	9.64E-07	2.37E-05	**UHMK1**	0.43	1.35	1.31E-09	3.18E-08
**YTHDC1**	0.42	1.33	9.79E-07	2.40E-05	**FZD3**	0.69	1.61	1.36E-09	3.30E-08
**GYLTL1B**	-0.60	0.66	9.86E-07	2.41E-05	**LCOR**	0.58	1.49	1.36E-09	3.30E-08
**SLC39A13**	0.54	1.45	1.00E-06	2.45E-05	**CNN2**	-1.25	0.42	1.50E-09	3.63E-08
**SNHG15**	-0.60	0.66	1.01E-06	2.46E-05	**ORAI3**	-1.44	0.37	1.64E-09	3.96E-08
**ZBTB7B**	-0.40	0.76	1.02E-06	2.49E-05	**NHLH2**	-1.06	0.48	1.64E-09	3.97E-08
**S100A9**	-0.39	0.77	1.03E-06	2.49E-05	**CPEB3**	0.97	1.95	1.66E-09	4.00E-08
**ANO6**	0.35	1.28	1.03E-06	2.49E-05	**PORCN**	-0.60	0.66	1.67E-09	4.03E-08
**NLRX1**	-0.45	0.73	1.04E-06	2.51E-05	**AQP9**	-1.05	0.48	1.68E-09	4.04E-08
**FKBP14**	0.53	1.44	1.04E-06	2.52E-05	**MESDC2**	0.46	1.38	1.77E-09	4.24E-08
**MED13L**	0.35	1.27	1.08E-06	2.59E-05	**MYC**	-0.47	0.72	1.77E-09	4.25E-08
**ABCC3**	0.44	1.36	1.09E-06	2.62E-05	**CFLAR**	-0.51	0.70	1.80E-09	4.31E-08
**MARK4**	0.43	1.34	1.13E-06	2.71E-05	**TMEM2**	0.54	1.46	1.85E-09	4.42E-08
**RNF139**	-0.52	0.70	1.13E-06	2.71E-05	**PLPP2**	0.73	1.65	1.89E-09	4.50E-08
**EZH2**	-0.46	0.73	1.13E-06	2.71E-05	**SRM**	0.43	1.35	1.91E-09	4.53E-08
**EGR3**	-0.91	0.53	1.18E-06	2.82E-05	**ANKRD13C**	-0.66	0.63	1.96E-09	4.66E-08
**PRDM1**	-0.54	0.69	1.20E-06	2.87E-05	**PRPF38A**	0.53	1.45	1.97E-09	4.67E-08
**AREG**	0.58	1.49	1.21E-06	2.87E-05	**SQSTM1**	0.50	1.41	1.98E-09	4.69E-08
**ARHGAP35**	0.34	1.27	1.21E-06	2.87E-05	**RALGDS**	-0.53	0.69	2.02E-09	4.77E-08
**APBB2**	0.39	1.31	1.22E-06	2.90E-05	**PLAT**	0.88	1.84	2.16E-09	5.08E-08
**FEZ1**	0.41	1.33	1.23E-06	2.92E-05	**RUBCN**	-0.53	0.69	2.17E-09	5.10E-08
**NOL3**	-0.57	0.67	1.24E-06	2.94E-05	**CDCA2**	0.62	1.54	2.17E-09	5.10E-08
**DHCR7**	-0.31	0.81	1.27E-06	3.00E-05	**MYL9**	-1.07	0.48	2.23E-09	5.23E-08
**DNAJB5**	-0.60	0.66	1.34E-06	3.16E-05	**08-sep**	-0.43	0.74	2.26E-09	5.30E-08
**BCL11B**	-0.47	0.72	1.36E-06	3.20E-05	**SFPQ**	0.39	1.31	2.31E-09	5.40E-08
**LUC7L3**	0.45	1.37	1.37E-06	3.21E-05	**STARD7**	0.42	1.34	2.33E-09	5.43E-08
**SLC39A2**	-0.76	0.59	1.37E-06	3.21E-05	**ARRDC3**	0.53	1.44	2.48E-09	5.76E-08
**SEMA3C**	0.43	1.34	1.39E-06	3.26E-05	**SLC3A2**	-0.50	0.71	2.48E-09	5.76E-08
**MTND6P4**	0.66	1.58	1.41E-06	3.29E-05	**PIKFYVE**	0.47	1.39	2.58E-09	5.99E-08
**TOP2A**	-0.33	0.80	1.41E-06	3.29E-05	**RRM2B**	-0.61	0.65	2.60E-09	6.03E-08
**IDS**	0.37	1.29	1.45E-06	3.38E-05	**CAMTA2**	-0.54	0.69	2.62E-09	6.05E-08
**ASAP2**	0.34	1.27	1.46E-06	3.39E-05	**GLUL**	-0.49	0.71	2.62E-09	6.05E-08
**PMAIP1**	0.59	1.51	1.46E-06	3.40E-05	**LYPD3**	-0.52	0.70	2.65E-09	6.10E-08
**USP53**	0.46	1.37	1.47E-06	3.41E-05	**ARSI**	0.49	1.40	2.70E-09	6.21E-08
**KRT5**	-0.26	0.83	1.48E-06	3.43E-05	**CARM1**	-0.62	0.65	2.78E-09	6.39E-08
**STXBP1**	0.40	1.32	1.53E-06	3.52E-05	**HMGB3**	0.51	1.42	2.84E-09	6.51E-08
**GPBP1**	0.44	1.36	1.56E-06	3.59E-05	**RHOD**	-0.45	0.73	2.96E-09	6.79E-08
**ZNF750**	-0.41	0.75	1.62E-06	3.73E-05	**PEPD**	-0.56	0.68	2.98E-09	6.80E-08
**PRDM10**	0.52	1.43	1.69E-06	3.88E-05	**PRPF4**	0.47	1.39	3.11E-09	7.10E-08
**LINC00707**	0.33	1.26	1.70E-06	3.90E-05	**PDIA4**	0.45	1.37	3.19E-09	7.26E-08
**RBBP4**	-0.39	0.76	1.71E-06	3.91E-05	**FAM193B**	-0.51	0.70	3.21E-09	7.31E-08
**DIAPH1**	0.28	1.22	1.75E-06	3.99E-05	**FAM84A**	-0.58	0.67	3.25E-09	7.37E-08
**PITPNM3**	-0.39	0.77	1.75E-06	3.99E-05	**LURAP1L**	-0.65	0.64	3.35E-09	7.60E-08
**FOXRED2**	-0.56	0.68	1.75E-06	3.99E-05	**DHDDS**	-0.54	0.69	3.54E-09	8.01E-08
**TMEM63C**	-0.53	0.69	1.76E-06	4.00E-05	**RAD51AP1**	0.73	1.66	3.61E-09	8.17E-08
**MYEOV**	-0.88	0.54	1.78E-06	4.04E-05	**FBRSL1**	-0.72	0.61	3.78E-09	8.54E-08
**DHX30**	0.35	1.28	1.79E-06	4.04E-05	**NRBP2**	-0.53	0.69	3.84E-09	8.65E-08

IER5L	-0.67	0.63	1.80E-06	4.06E-05
PTPN1	0.32	1.25	1.80E-06	4.06E-05
PITX1	-0.47	0.72	1.80E-06	4.07E-05
LRAT	-0.56	0.68	1.81E-06	4.08E-05
NR1D1	-0.62	0.65	1.84E-06	4.13E-05
CNTROB	0.42	1.34	1.84E-06	4.13E-05
PLEKHM2	0.39	1.31	1.88E-06	4.21E-05
GRHL3	-0.35	0.78	1.92E-06	4.28E-05
PPP1R13B	0.42	1.33	1.92E-06	4.29E-05
ZBED2	0.38	1.30	1.93E-06	4.31E-05
TGFBR2	0.31	1.24	1.98E-06	4.40E-05
E2F7	-0.50	0.71	2.00E-06	4.44E-05
C1orf233	-0.71	0.61	2.00E-06	4.44E-05
MAPK7	-0.56	0.68	2.02E-06	4.48E-05
SH3TC1	-0.50	0.70	2.03E-06	4.50E-05
ACTN1	0.32	1.24	2.07E-06	4.55E-05
PPM1B	0.48	1.39	2.07E-06	4.55E-05
ANKRD65	-0.73	0.60	2.07E-06	4.56E-05
RBBP8NL	-0.75	0.59	2.08E-06	4.58E-05
IL22RA1	-0.56	0.68	2.10E-06	4.60E-05
C15orf59	-0.84	0.56	2.20E-06	4.81E-05
FGFR3	-0.37	0.77	2.22E-06	4.85E-05
CDV3	0.32	1.25	2.22E-06	4.85E-05
SOX9	-0.35	0.78	2.26E-06	4.92E-05
DKC1	0.30	1.23	2.30E-06	5.01E-05
AXIN1	0.39	1.31	2.32E-06	5.03E-05
ZNF408	-0.61	0.65	2.34E-06	5.07E-05
TP53	-0.41	0.75	2.37E-06	5.13E-05
ATP13A3	0.36	1.28	2.40E-06	5.18E-05
CXCL3	0.86	1.82	2.42E-06	5.23E-05
GGT6	-0.54	0.69	2.44E-06	5.25E-05
MFSD5	-0.44	0.74	2.45E-06	5.26E-05
PLTP	-0.37	0.77	2.45E-06	5.26E-05
ANKRD13C	0.47	1.39	2.48E-06	5.32E-05
MCM3AP	0.42	1.33	2.49E-06	5.32E-05
ELL2	0.33	1.26	2.49E-06	5.33E-05
TACSTD2	0.34	1.26	2.53E-06	5.40E-05
GPRC5C	-0.87	0.55	2.58E-06	5.51E-05
SGOL2	0.48	1.40	2.60E-06	5.53E-05
PTMA	-0.27	0.83	2.61E-06	5.54E-05
TOMM70A	-0.36	0.78	2.65E-06	5.62E-05
RNF6	0.38	1.30	2.66E-06	5.64E-05
ORC2	0.42	1.34	2.67E-06	5.64E-05
FGD6	0.35	1.28	2.67E-06	5.64E-05
FAM168B	0.30	1.23	2.82E-06	5.95E-05
SERPINE2	-0.33	0.80	2.83E-06	5.96E-05
WDR43	0.34	1.27	2.84E-06	5.96E-05
HIVEP2	0.38	1.30	2.84E-06	5.96E-05
CLASP2	0.35	1.28	2.85E-06	5.96E-05
RAB12	0.46	1.38	2.90E-06	6.06E-05
AFAP1	0.42	1.34	2.92E-06	6.10E-05
SYMPK	0.33	1.26	2.94E-06	6.14E-05
VAV3	-0.58	0.67	3.07E-06	6.40E-05
RAC3	-0.61	0.66	3.10E-06	6.45E-05
IGSF8	-0.49	0.71	3.17E-06	6.58E-05
HNRNPH1	0.29	1.22	3.18E-06	6.58E-05
ZBED3	-0.79	0.58	3.23E-06	6.69E-05
FANCG	-0.45	0.73	3.25E-06	6.71E-05
ZNF518B	0.41	1.32	3.28E-06	6.77E-05
STK3	0.46	1.38	3.29E-06	6.77E-05
ISG15	-0.61	0.66	3.33E-06	6.84E-05
TPRG1	-0.81	0.57	3.36E-06	6.89E-05
ANTXR2	0.36	1.29	3.36E-06	6.89E-05
OSBPL2	0.40	1.32	3.37E-06	6.89E-05
ZNF618	-0.54	0.69	3.37E-06	6.89E-05
TOP1	0.34	1.27	3.45E-06	7.03E-05
TRIP12	0.29	1.22	3.53E-06	7.20E-05
ADGRL1	-0.54	0.69	3.60E-06	7.33E-05
SPATS2	0.36	1.28	3.61E-06	7.33E-05
TPM1	0.34	1.27	3.61E-06	7.33E-05
KDM3B	0.34	1.27	3.64E-06	7.38E-05
TSPAN14	0.33	1.26	3.66E-06	7.40E-05
EIF4G2	0.25	1.19	3.74E-06	7.56E-05
DHX38	0.33	1.26	3.80E-06	7.66E-05
VSIG10L	-0.48	0.72	3.81E-06	7.67E-05
MAST2	0.39	1.31	3.82E-06	7.67E-05
KPNA4	0.37	1.29	3.83E-06	7.68E-05
NUMB	0.31	1.24	3.88E-06	7.78E-05
KHK	-0.83	0.56	3.96E-06	7.93E-05
PDGFA	-0.59	0.66	4.02E-06	8.04E-05
RIPK1	0.44	1.35	4.07E-06	8.13E-05
TRANK1	-0.86	0.55	4.12E-06	8.22E-05
IRF2BP1	-0.62	0.65	4.14E-06	8.24E-05
MGST2	-0.49	0.71	4.16E-06	8.27E-05
PINLYP	-0.68	0.62	4.18E-06	8.30E-05
PDGFB	-0.74	0.60	4.21E-06	8.34E-05
PRR3	-0.69	0.62	4.28E-06	8.46E-05
RNF111	0.39	1.31	4.32E-06	8.53E-05
SRSF11	0.33	1.26	4.35E-06	8.59E-05
HSPB1	-0.38	0.77	4.39E-06	8.64E-05

PCBP4	-0.50	0.71	3.85E-09	8.66E-08
SLC27A4	-0.54	0.69	3.87E-09	8.69E-08
HYOU1	0.43	1.34	3.93E-09	8.80E-08
HNRNPU	0.41	1.33	3.94E-09	8.82E-08
CISH	0.66	1.58	3.97E-09	8.87E-08
PPT1	0.45	1.36	4.10E-09	9.15E-08
DHCR24	-0.40	0.76	4.12E-09	9.17E-08
ELF4	-0.55	0.68	4.23E-09	9.40E-08
ARHGEF5	-0.58	0.67	4.26E-09	9.44E-08
TK2	-0.85	0.56	4.28E-09	9.48E-08
B4GALT2	-0.50	0.71	4.36E-09	9.65E-08
S100A2	-0.42	0.75	4.39E-09	9.69E-08
GCNT4	-0.80	0.57	4.58E-09	1.01E-07
RFC3	0.65	1.57	4.68E-09	1.03E-07
ULK1	-0.49	0.71	5.07E-09	1.11E-07
TBCD	0.45	1.37	5.10E-09	1.12E-07
ARL5B	0.48	1.40	5.27E-09	1.15E-07
WWC2	0.49	1.40	5.43E-09	1.19E-07
CDS2	0.47	1.39	5.63E-09	1.23E-07
PHB	-0.49	0.71	5.77E-09	1.26E-07
SLC7A1	-0.37	0.77	6.34E-09	1.38E-07
TJP2	-0.40	0.76	6.36E-09	1.38E-07
CELSR2	-0.45	0.73	6.36E-09	1.38E-07
AHSA2	-0.61	0.66	6.42E-09	1.39E-07
PLK3	-0.56	0.68	6.44E-09	1.39E-07
NDC1	0.55	1.46	6.53E-09	1.41E-07
STARD5	-0.80	0.57	6.55E-09	1.41E-07
FAM222B	-0.54	0.69	6.62E-09	1.42E-07
NRP2	0.48	1.39	6.72E-09	1.44E-07
UBN1	-0.47	0.72	6.75E-09	1.45E-07
TFRC	0.45	1.37	6.76E-09	1.45E-07
WHSC1	0.50	1.41	6.94E-09	1.48E-07
NR1D2	0.65	1.57	7.40E-09	1.58E-07
CCNB1IP1	-0.55	0.68	7.79E-09	1.66E-07
NIT1	-0.58	0.67	7.94E-09	1.69E-07
DGUOK	-0.70	0.62	8.14E-09	1.73E-07
NICN1	-0.72	0.61	8.38E-09	1.78E-07
NETO2	-0.49	0.71	8.43E-09	1.79E-07
IL36RN	-0.98	0.51	8.82E-09	1.86E-07
HMGA2	1.38	2.60	8.83E-09	1.86E-07
XG	-0.53	0.69	9.16E-09	1.93E-07
CYB5R3	-0.39	0.76	9.22E-09	1.94E-07
ATP8B2	0.56	1.47	9.31E-09	1.96E-07
EPHA3	-1.75	0.30	9.56E-09	2.01E-07
CCDC91	-0.67	0.63	9.80E-09	2.05E-07
PRDX3	0.41	1.33	9.86E-09	2.06E-07
C2CD3	-0.57	0.67	9.97E-09	2.08E-07
ANP32B	0.43	1.35	1.00E-08	2.09E-07
NCAPG	0.61	1.53	1.01E-08	2.10E-07
CYP2W1	-1.13	0.46	1.01E-08	2.10E-07
ACSL4	0.51	1.42	1.02E-08	2.11E-07
RP11-7K24.3	-0.87	0.55	1.03E-08	2.13E-07
BCL2L12	0.61	1.52	1.05E-08	2.17E-07
DDX3X	0.37	1.30	1.07E-08	2.21E-07
LPCAT1	0.47	1.39	1.08E-08	2.22E-07
HAUS8	0.80	1.74	1.12E-08	2.30E-07
ERO1A	0.51	1.43	1.14E-08	2.35E-07
SLC1A5	-0.44	0.74	1.15E-08	2.36E-07
SLC6A8	0.50	1.41	1.16E-08	2.39E-07
MARCKSL1	-0.74	0.60	1.20E-08	2.45E-07
SEMA4C	0.60	1.51	1.23E-08	2.50E-07
MYO9B	-0.47	0.72	1.23E-08	2.51E-07
EDN1	-0.54	0.69	1.27E-08	2.58E-07
P3H3	0.69	1.61	1.28E-08	2.60E-07
OIP5-AS1	0.41	1.33	1.31E-08	2.66E-07
FOLR3	-0.92	0.53	1.37E-08	2.79E-07
LINC00294	-0.65	0.64	1.40E-08	2.83E-07
EPPK1	-0.41	0.75	1.40E-08	2.83E-07
CYTH3	-0.47	0.72	1.41E-08	2.85E-07
URM1	-0.63	0.65	1.42E-08	2.85E-07
UBE2S	0.58	1.50	1.42E-08	2.85E-07
TM7SF3	-0.39	0.76	1.44E-08	2.90E-07
PRPS1	0.52	1.44	1.45E-08	2.92E-07
SORBS3	-0.56	0.68	1.46E-08	2.92E-07
ACKR3	-0.44	0.74	1.47E-08	2.93E-07
SNHG16	-0.62	0.65	1.52E-08	3.05E-07
CBFB	0.47	1.38	1.56E-08	3.12E-07
TTK	0.64	1.56	1.66E-08	3.31E-07
KLF7	-0.50	0.71	1.70E-08	3.38E-07
MBP	0.44	1.36	1.72E-08	3.41E-07
TIGAR	-0.54	0.69	1.74E-08	3.44E-07
GPR153	-0.55	0.68	1.82E-08	3.61E-07
CACNB3	-0.62	0.65	1.84E-08	3.64E-07
PLEC	-0.38	0.77	1.88E-08	3.72E-07
KIF26B	-0.74	0.60	1.91E-08	3.76E-07
MARK2	-0.47	0.72	1.91E-08	3.76E-07
FGFR3	0.46	1.38	2.01E-08	3.95E-07
GAS2L3	0.71	1.64	2.08E-08	4.09E-07
CYP26B1	-0.78	0.58	2.10E-08	4.12E-07
STX6	0.44	1.35	2.14E-08	4.18E-07

**WDR26**	0.34	1.26	4.42E-06	8.70E-05
**MDC1**	0.33	1.26	4.44E-06	8.72E-05
**NCOA5**	0.39	1.31	4.47E-06	8.76E-05
**CLMP**	-0.49	0.71	4.47E-06	8.76E-05
**PPP1R10**	0.35	1.27	4.48E-06	8.77E-05
**CDK4**	-0.43	0.74	4.54E-06	8.87E-05
**SUN1**	0.30	1.23	4.55E-06	8.87E-05
**KRT16**	-0.33	0.80	4.60E-06	8.95E-05
**VLDLR**	-0.60	0.66	4.61E-06	8.95E-05
**SNAP23**	0.44	1.36	4.61E-06	8.95E-05
**GOLGA8A**	0.67	1.59	4.62E-06	8.95E-05
**FAM110A**	-0.54	0.69	4.65E-06	9.01E-05
**SMURF1**	0.36	1.28	4.66E-06	9.01E-05
**COX5B**	-0.38	0.77	4.71E-06	9.09E-05
**ACP5**	-0.72	0.61	4.80E-06	9.25E-05
**07-mar**	0.40	1.32	4.85E-06	9.33E-05
**IMPA2**	-0.42	0.75	4.96E-06	9.53E-05
**B3GNT9**	-0.67	0.63	4.99E-06	9.58E-05
**ZMAT3**	-0.50	0.71	5.05E-06	9.68E-05
**LATS1**	0.39	1.31	5.07E-06	9.71E-05
**ALOXE3**	-0.61	0.66	5.21E-06	9.96E-05
**LYRM1**	-0.53	0.69	5.26E-06	1.00E-04
**SACS**	0.40	1.32	5.29E-06	1.01E-04
**CHMP2B**	0.44	1.36	5.29E-06	1.01E-04
**CTTN**	0.26	1.20	5.30E-06	1.01E-04
**WDR34**	-0.46	0.73	5.35E-06	1.02E-04
**PPHLN1**	0.36	1.28	5.40E-06	1.02E-04
**IFIT1**	-0.67	0.63	5.47E-06	1.04E-04
**PDPN**	-0.49	0.71	5.53E-06	1.05E-04
**GNAO1**	-0.83	0.56	5.55E-06	1.05E-04
**FASN**	-0.29	0.82	5.57E-06	1.05E-04
**CD59**	0.26	1.20	5.67E-06	1.07E-04
**CTDP1**	0.45	1.36	5.82E-06	1.09E-04
**S100A8**	-0.50	0.71	5.98E-06	1.12E-04
**H2AFY2**	-0.41	0.75	5.99E-06	1.12E-04
**DDX60**	-0.51	0.70	6.29E-06	1.18E-04
**CIT**	0.33	1.26	6.34E-06	1.18E-04
**YWHAG**	0.25	1.19	6.41E-06	1.20E-04
**PAK6**	-0.44	0.74	6.48E-06	1.21E-04
**NDEL1**	0.33	1.26	6.49E-06	1.21E-04
**CTDSP1**	-0.38	0.77	6.60E-06	1.23E-04
**MAP1LC3B**	0.38	1.30	6.61E-06	1.23E-04
**PLIN2**	-0.50	0.71	6.72E-06	1.25E-04
**POLA1**	-0.45	0.73	6.76E-06	1.25E-04
**NT5E**	0.34	1.27	6.81E-06	1.26E-04
**ERCC1**	0.34	1.27	6.82E-06	1.26E-04
**SIPA1L1**	0.34	1.27	6.97E-06	1.28E-04
**SECTM1**	-0.79	0.58	6.98E-06	1.29E-04
**NUP50**	0.31	1.24	7.04E-06	1.30E-04
**TDG**	0.42	1.34	7.30E-06	1.34E-04
**FXR1**	0.32	1.25	7.39E-06	1.35E-04
**ANXA2**	0.23	1.17	7.46E-06	1.37E-04
**POLR2A**	0.29	1.22	7.51E-06	1.37E-04
**FAM83H-AS1**	-0.48	0.72	7.51E-06	1.37E-04
**GLS**	0.45	1.37	7.54E-06	1.38E-04
**RTN4R**	-0.60	0.66	7.62E-06	1.39E-04
**MAP4K4**	0.26	1.20	7.63E-06	1.39E-04
**PRR11**	0.31	1.24	7.72E-06	1.40E-04
**CEP170B**	0.38	1.30	7.85E-06	1.42E-04
**CEACAM1**	-0.61	0.65	7.90E-06	1.43E-04
**PTPRG**	0.45	1.37	7.96E-06	1.44E-04
**TM4SF1**	0.34	1.27	8.08E-06	1.46E-04
**ACAT2**	-0.39	0.76	8.13E-06	1.47E-04
**SLC20A1**	-0.29	0.82	8.14E-06	1.47E-04
**CHD7**	0.40	1.32	8.15E-06	1.47E-04
**TNKS2**	0.35	1.28	8.28E-06	1.49E-04
**SPAG5**	0.33	1.26	8.35E-06	1.50E-04
**LTBP2**	-0.35	0.79	8.43E-06	1.51E-04
**THEM6**	-0.55	0.68	8.62E-06	1.54E-04
**LINC01468**	0.52	1.44	8.67E-06	1.55E-04
**ARHGEF10**	0.40	1.32	8.75E-06	1.56E-04
**FUBP3**	0.32	1.25	8.77E-06	1.56E-04
**LYAR**	0.33	1.26	8.87E-06	1.58E-04
**CHMP7**	0.38	1.30	8.89E-06	1.58E-04
**STX1A**	0.63	1.55	8.92E-06	1.58E-04
**DDIAS**	0.45	1.37	8.94E-06	1.59E-04
**IL1A**	0.42	1.34	8.96E-06	1.59E-04
**ARFGEF2**	0.33	1.25	8.98E-06	1.59E-04
**PFKP**	0.28	1.21	9.11E-06	1.61E-04
**HIP1R**	0.36	1.28	9.13E-06	1.61E-04
**PRRG1**	0.50	1.42	9.14E-06	1.61E-04
**MAML1**	0.38	1.30	9.15E-06	1.61E-04
**UNK**	-0.50	0.71	9.27E-06	1.63E-04
**TMEM246**	-0.40	0.76	9.29E-06	1.63E-04
**GBF1**	0.32	1.25	9.41E-06	1.65E-04
**TUBB6**	0.29	1.22	9.57E-06	1.68E-04
**ARHGEF16**	-0.45	0.73	9.70E-06	1.70E-04
**TMEM184A**	-0.36	0.78	9.78E-06	1.71E-04
**CCDC93**	0.39	1.31	9.91E-06	1.73E-04
**DMTF1**	0.45	1.37	9.91E-06	1.73E-04
**QKI**	0.43	1.35	2.15E-08	4.21E-07
**RALGAPA1**	-0.55	0.68	2.16E-08	4.22E-07
**U2SURP**	0.39	1.31	2.18E-08	4.24E-07
**CDCA5**	0.58	1.50	2.18E-08	4.25E-07
**IGFL1**	-0.48	0.72	2.22E-08	4.32E-07
**ZCCHC3**	0.58	1.50	2.23E-08	4.34E-07
**GINS1**	0.59	1.50	2.30E-08	4.45E-07
**ATG2A**	-0.50	0.71	2.30E-08	4.46E-07
**MAMDC4**	-0.85	0.56	2.32E-08	4.49E-07
**SUZ12**	0.47	1.38	2.43E-08	4.69E-07
**PRPF19**	0.39	1.31	2.45E-08	4.72E-07
**NCAPD2**	0.49	1.40	2.50E-08	4.81E-07
**CENPB**	0.43	1.34	2.50E-08	4.81E-07
**CAPRIN1**	0.37	1.29	2.53E-08	4.86E-07
**STC2**	0.92	1.89	2.57E-08	4.91E-07
**TTC28**	-0.98	0.51	2.57E-08	4.92E-07
**FAM214B**	-0.50	0.70	2.67E-08	5.11E-07
**NBEAL2**	-0.43	0.74	2.70E-08	5.15E-07
**C22orf23**	-0.74	0.60	2.71E-08	5.16E-07
**SLC22A15**	-0.54	0.69	3.02E-08	5.74E-07
**EML2**	-0.40	0.76	3.05E-08	5.79E-07
**G2E3**	0.55	1.46	3.14E-08	5.95E-07
**DST**	-0.35	0.79	3.24E-08	6.14E-07
**AGFG2**	-0.63	0.65	3.54E-08	6.69E-07
**CALCOCO1**	-0.54	0.69	3.58E-08	6.76E-07
**AK4**	0.46	1.38	3.58E-08	6.76E-07
**MYD88**	-0.44	0.74	3.67E-08	6.92E-07
**CACYBP**	0.48	1.39	3.72E-08	7.00E-07
**TRAM2**	0.50	1.42	3.74E-08	7.03E-07
**USP46**	0.53	1.44	3.75E-08	7.04E-07
**DCK**	0.63	1.54	3.77E-08	7.06E-07
**PRRC2B**	-0.45	0.73	3.83E-08	7.16E-07
**DSG2**	-0.38	0.77	3.83E-08	7.16E-07
**CSTA**	-0.47	0.72	3.87E-08	7.22E-07
**LGALSL**	-0.48	0.72	3.95E-08	7.35E-07
**C14orf1**	-0.45	0.73	3.96E-08	7.36E-07
**MAD2L1**	0.54	1.45	4.01E-08	7.44E-07
**DUSP7**	-0.51	0.70	4.07E-08	7.55E-07
**TMEM79**	-0.49	0.71	4.10E-08	7.59E-07
**MORN4**	-0.74	0.60	4.14E-08	7.65E-07
**IPO5**	0.40	1.32	4.14E-08	7.65E-07
**RBM47**	0.47	1.38	4.16E-08	7.67E-07
**TMEM180**	-0.85	0.56	4.18E-08	7.70E-07
**MAP3K2**	-0.47	0.72	4.34E-08	7.99E-07
**TUBB**	0.45	1.36	4.39E-08	8.07E-07
**CKAP4**	0.42	1.34	4.49E-08	8.24E-07
**KIF3C**	-0.49	0.71	4.60E-08	8.43E-07
**CIT**	0.54	1.45	4.65E-08	8.52E-07
**CAPN1**	-0.36	0.78	4.70E-08	8.59E-07
**ANO10**	-0.48	0.72	4.70E-08	8.59E-07
**ATP13A2**	-0.43	0.74	4.74E-08	8.63E-07
**EMP1**	-0.36	0.78	4.86E-08	8.84E-07
**STK40**	-0.59	0.66	4.90E-08	8.91E-07
**PGAP2**	-0.52	0.70	5.00E-08	9.07E-07
**RGS12**	-0.42	0.75	5.20E-08	9.42E-07
**STYX**	0.49	1.41	5.27E-08	9.53E-07
**HNRNPM**	0.38	1.30	5.29E-08	9.56E-07
**SEMA7A**	-0.64	0.64	5.38E-08	9.72E-07
**IRF1**	0.78	1.72	5.45E-08	9.83E-07
**MYBL2**	0.56	1.48	5.57E-08	1.00E-06
**OSTM1**	0.46	1.38	5.60E-08	1.01E-06
**SLC39A14**	0.48	1.39	5.61E-08	1.01E-06
**TRAK1**	-0.41	0.75	5.67E-08	1.02E-06
**C3**	1.02	2.03	5.70E-08	1.02E-06
**HNRNPR**	0.42	1.34	5.75E-08	1.03E-06
**TCP1**	0.38	1.30	5.98E-08	1.07E-06
**DUSP4**	-0.53	0.69	6.20E-08	1.11E-06
**SH2D4A**	0.53	1.44	6.21E-08	1.11E-06
**SESN2**	-0.53	0.69	6.27E-08	1.12E-06
**ERAL1**	-0.49	0.71	6.28E-08	1.12E-06
**ARMCX3**	-0.53	0.69	6.33E-08	1.12E-06
**GPC1**	-0.36	0.78	6.46E-08	1.14E-06
**FBRS**	-0.45	0.73	6.64E-08	1.18E-06
**MUC16**	-0.95	0.52	6.75E-08	1.19E-06
**CENPF**	0.52	1.44	6.90E-08	1.22E-06
**SAMD5**	-0.95	0.52	6.96E-08	1.23E-06
**DBF4**	0.60	1.51	7.18E-08	1.26E-06
**ARPC4**	-0.58	0.67	7.19E-08	1.27E-06
**MCM4**	0.54	1.45	7.39E-08	1.30E-06
**ZSCAN12P1**	1.41	2.66	7.48E-08	1.31E-06
**ZNF518B**	-0.52	0.70	7.61E-08	1.33E-06
**STEAP3**	0.44	1.35	7.63E-08	1.33E-06
**PBXIP1**	-0.60	0.66	7.71E-08	1.35E-06
**S100PBP**	0.49	1.41	7.87E-08	1.37E-06
**LGR6**	2.51	5.68	8.12E-08	1.41E-06
**MASTL**	0.53	1.44	8.13E-08	1.42E-06
**MBD6**	-0.52	0.70	8.43E-08	1.46E-06
**ANKRD17**	-0.39	0.76	8.47E-08	1.47E-06
**A2ML1**	-0.49	0.71	8.60E-08	1.49E-06
**TOB2**	-0.44	0.74	8.70E-08	1.51E-06

KANSL1	-0.44	0.74	9.92E-06	1.73E-04
HAPLN3	-0.81	0.57	1.01E-05	1.76E-04
STRN3	0.42	1.34	1.02E-05	1.76E-04
ZMYND11	0.35	1.28	1.02E-05	1.76E-04
ORAI1	-0.45	0.73	1.02E-05	1.77E-04
CDC20P1	0.61	1.52	1.02E-05	1.77E-04
SLC27A3	-0.65	0.64	1.02E-05	1.77E-04
PYCR1	-0.43	0.74	1.03E-05	1.77E-04
CENPJ	0.48	1.39	1.05E-05	1.80E-04
NUAK2	-0.49	0.71	1.05E-05	1.80E-04
GRHL1	-0.38	0.77	1.05E-05	1.81E-04
EFNA3	-0.52	0.70	1.06E-05	1.81E-04
SFSWAP	0.33	1.25	1.08E-05	1.86E-04
NGFRAP1	-0.34	0.79	1.11E-05	1.90E-04
PPP1R12C	0.43	1.34	1.11E-05	1.90E-04
MAPK8IP3	0.43	1.35	1.12E-05	1.90E-04
RPS6KA5	-0.68	0.62	1.13E-05	1.93E-04
SIK3	0.39	1.31	1.13E-05	1.93E-04
GNB1	0.24	1.18	1.14E-05	1.95E-04
ACOT9	0.37	1.30	1.16E-05	1.97E-04
WAPL	0.36	1.28	1.17E-05	1.99E-04
WWC1	0.37	1.30	1.17E-05	1.99E-04
ELOVL6	-0.38	0.77	1.18E-05	1.99E-04
UBE2O	0.36	1.28	1.20E-05	2.02E-04
SLC6A6	0.39	1.31	1.22E-05	2.07E-04
SLIT2	0.40	1.32	1.23E-05	2.08E-04
CRK	0.31	1.24	1.24E-05	2.09E-04
KMT2E	-0.35	0.79	1.25E-05	2.10E-04
WEE1	-0.45	0.73	1.26E-05	2.12E-04
TMCC3	0.33	1.26	1.27E-05	2.13E-04
RABGAP1	0.32	1.25	1.28E-05	2.15E-04
PCLO	-0.54	0.69	1.29E-05	2.15E-04
SUPT5H	0.33	1.26	1.31E-05	2.19E-04
MYLK	-0.60	0.66	1.32E-05	2.20E-04
CALB1	-0.63	0.65	1.32E-05	2.20E-04
PCDH1	-0.31	0.81	1.32E-05	2.20E-04
CBL	0.36	1.28	1.33E-05	2.22E-04
MAPK13	-0.29	0.82	1.34E-05	2.23E-04
TNRC6A	0.30	1.23	1.35E-05	2.23E-04
ZDHHC7	0.32	1.25	1.35E-05	2.23E-04
RUSC1	-0.34	0.79	1.35E-05	2.24E-04
SYNJ2	0.38	1.30	1.35E-05	2.24E-04
TMEM69	0.37	1.29	1.38E-05	2.27E-04
TRAPPC10	0.36	1.28	1.41E-05	2.33E-04
CCDC84	0.67	1.59	1.42E-05	2.33E-04
SLMAP	0.33	1.26	1.43E-05	2.35E-04
MMP10	-0.70	0.62	1.47E-05	2.41E-04
PFKFB4	-0.54	0.69	1.48E-05	2.43E-04
TRIB1	-0.46	0.73	1.48E-05	2.43E-04
PTPRB	-0.81	0.57	1.49E-05	2.44E-04
NUP58	0.30	1.23	1.49E-05	2.44E-04
ZNF721	0.57	1.48	1.50E-05	2.45E-04
KIAA0556	0.40	1.32	1.50E-05	2.45E-04
HELLS	-0.50	0.71	1.50E-05	2.45E-04
IER5	-0.31	0.80	1.50E-05	2.45E-04
H1FX	-0.45	0.73	1.52E-05	2.46E-04
ROBO4	-0.79	0.58	1.52E-05	2.47E-04
KLK14	-0.74	0.60	1.53E-05	2.48E-04
PAWR	0.38	1.31	1.57E-05	2.55E-04
INPP4B	0.38	1.30	1.58E-05	2.55E-04
CEBPD	-0.50	0.71	1.58E-05	2.56E-04
TCEB3	0.29	1.22	1.58E-05	2.56E-04
DCUN1D3	-0.53	0.69	1.59E-05	2.57E-04
NDFIP2	0.30	1.23	1.61E-05	2.59E-04
CSTB	-0.30	0.81	1.62E-05	2.61E-04
ETF1	0.26	1.20	1.63E-05	2.61E-04
C4orf48	-0.77	0.59	1.64E-05	2.63E-04
FRMD5	0.72	1.65	1.65E-05	2.64E-04
UTP20	0.34	1.27	1.67E-05	2.67E-04
ERVMER34-1	-0.54	0.69	1.67E-05	2.67E-04
RB1CC1	0.44	1.35	1.69E-05	2.69E-04
FOPNL	0.40	1.32	1.71E-05	2.73E-04
NF1	0.34	1.27	1.71E-05	2.73E-04
GCSH	-0.80	0.57	1.75E-05	2.78E-04
SH3BP2	-0.44	0.73	1.75E-05	2.79E-04
MAN2A1	0.37	1.29	1.77E-05	2.81E-04
SP6	-0.44	0.74	1.79E-05	2.85E-04
FEN1	-0.34	0.79	1.80E-05	2.85E-04
USP16	0.40	1.32	1.83E-05	2.89E-04
OBSL1	-0.53	0.69	1.83E-05	2.90E-04
TNFAIP8L3	-0.78	0.58	1.85E-05	2.92E-04
EFNB2	0.28	1.21	1.85E-05	2.92E-04
TEF	-0.52	0.70	1.87E-05	2.94E-04
CAMSAP1	0.32	1.25	1.87E-05	2.94E-04
TP53I11	-0.41	0.75	1.91E-05	3.00E-04
CLIP2	0.50	1.41	1.91E-05	3.00E-04
SMG1P3	0.80	1.74	1.91E-05	3.00E-04
PTP4A1	0.35	1.27	1.94E-05	3.03E-04
HDAC7	0.35	1.28	1.94E-05	3.03E-04
ID1	-0.33	0.79	1.96E-05	3.06E-04
PDE7A	0.48	1.40	8.83E-08	1.53E-06
ACTN4	-0.35	0.78	8.96E-08	1.55E-06
SET	0.38	1.30	9.04E-08	1.56E-06
NFIA	-0.48	0.72	9.15E-08	1.58E-06
THBS2	0.49	1.41	9.22E-08	1.59E-06
DDX3Y	0.43	1.35	9.23E-08	1.59E-06
HSPA8	0.41	1.32	9.45E-08	1.62E-06
HSPH1	0.37	1.30	9.57E-08	1.64E-06
LFNG	0.56	1.47	9.60E-08	1.64E-06
SPOPL	0.54	1.45	9.86E-08	1.69E-06
SNHG8	-0.44	0.74	1.02E-07	1.73E-06
VPS37C	-0.54	0.69	1.02E-07	1.74E-06
TAF15	0.36	1.28	1.03E-07	1.75E-06
SLC9A6	0.49	1.41	1.05E-07	1.78E-06
KIAA0513	-0.63	0.65	1.07E-07	1.82E-06
GCC2	0.49	1.41	1.08E-07	1.83E-06
PIP5K1C	-0.51	0.70	1.13E-07	1.91E-06
SSX2IP	0.48	1.39	1.14E-07	1.93E-06
UBE2F	-0.93	0.53	1.16E-07	1.96E-06
KLF11	0.69	1.62	1.17E-07	1.97E-06
CA2	0.38	1.31	1.19E-07	2.00E-06
TDG	0.46	1.37	1.19E-07	2.01E-06
PPP1CC	0.38	1.30	1.24E-07	2.08E-06
GRSF1	0.39	1.31	1.27E-07	2.13E-06
TNFRSF10C	-1.60	0.33	1.30E-07	2.18E-06
THY1	1.77	3.41	1.30E-07	2.18E-06
NUF2	0.65	1.57	1.31E-07	2.19E-06
EPN1	-0.40	0.76	1.31E-07	2.19E-06
BIRC5	0.64	1.56	1.32E-07	2.21E-06
ELAVL2	0.65	1.57	1.41E-07	2.35E-06
USP11	-0.65	0.64	1.43E-07	2.37E-06
PPT2	-0.90	0.53	1.53E-07	2.54E-06
UBQLN4	-0.49	0.71	1.58E-07	2.62E-06
MTHFD2	0.52	1.43	1.58E-07	2.62E-06
ZNF777	-0.61	0.66	1.59E-07	2.64E-06
PTMA	0.43	1.35	1.60E-07	2.65E-06
ANP32A	0.37	1.29	1.62E-07	2.67E-06
XPO4	0.43	1.35	1.63E-07	2.70E-06
RP11-1002K11.1	-0.66	0.63	1.65E-07	2.72E-06
NOC2L	0.38	1.30	1.67E-07	2.76E-06
COX7A2	-0.41	0.75	1.70E-07	2.80E-06
PHTF2	0.48	1.39	1.71E-07	2.81E-06
SHISA5	-0.36	0.78	1.73E-07	2.83E-06
SRSF7	0.39	1.31	1.75E-07	2.87E-06
MPZL3	-0.60	0.66	1.77E-07	2.90E-06
CLOCK	0.38	1.30	1.77E-07	2.90E-06
SENP2	0.45	1.36	1.79E-07	2.93E-06
SSH1	-0.43	0.74	1.80E-07	2.93E-06
FAM46A	-0.79	0.58	1.82E-07	2.96E-06
FAM134C	-0.45	0.73	1.84E-07	3.00E-06
CMSS1	0.58	1.50	1.87E-07	3.05E-06
APOL3	1.38	2.60	1.89E-07	3.07E-06
BCL2L11	0.57	1.48	1.92E-07	3.11E-06
COL5A1	0.46	1.37	1.93E-07	3.12E-06
EHD1	-0.43	0.74	1.99E-07	3.23E-06
CALM2	0.36	1.29	2.06E-07	3.32E-06
CGN	0.68	1.60	2.07E-07	3.34E-06
KRT34	-1.17	0.44	2.11E-07	3.41E-06
DVL3	-0.40	0.76	2.12E-07	3.41E-06
RDH16	1.26	2.40	2.13E-07	3.43E-06
RNF44	-0.45	0.73	2.18E-07	3.50E-06
CELF2	-0.73	0.60	2.23E-07	3.57E-06
CDCA8	0.56	1.48	2.26E-07	3.62E-06
RPTOR	-0.43	0.74	2.29E-07	3.66E-06
LDLRAD3	-0.52	0.70	2.32E-07	3.71E-06
ATN1	-0.45	0.73	2.34E-07	3.73E-06
GAB1	-0.52	0.70	2.36E-07	3.76E-06
ABCB9	0.69	1.62	2.37E-07	3.77E-06
NCOA6	-0.46	0.73	2.38E-07	3.79E-06
DUOXA1	-0.47	0.72	2.49E-07	3.95E-06
KHNYN	-0.40	0.76	2.50E-07	3.96E-06
PEA15	-0.35	0.78	2.50E-07	3.96E-06
VIM	0.76	1.70	2.55E-07	4.04E-06
LRP8	0.53	1.45	2.58E-07	4.09E-06
KANSL3	-0.42	0.75	2.62E-07	4.14E-06
DNAJB1	-0.36	0.78	2.67E-07	4.22E-06
PTRF	-0.34	0.79	2.69E-07	4.24E-06
CCT5	0.39	1.31	2.71E-07	4.27E-06
E2F4	-0.49	0.71	2.88E-07	4.53E-06
SOX4	-0.43	0.74	2.91E-07	4.57E-06
SGK223	-0.44	0.74	2.92E-07	4.57E-06
HSPA13	0.54	1.45	2.94E-07	4.61E-06
SNX4	0.48	1.40	2.97E-07	4.64E-06
FZD5	0.56	1.48	3.01E-07	4.70E-06
DAPK1	-0.67	0.63	3.05E-07	4.75E-06
SCML2	1.16	2.23	3.05E-07	4.75E-06
MSMO1	-0.34	0.79	3.13E-07	4.87E-06
ATP9A	-0.48	0.72	3.15E-07	4.89E-06
FAM101B	0.65	1.56	3.15E-07	4.89E-06
NDRG1	-0.44	0.73	3.21E-07	4.98E-06

**CPE**	-0.58	0.67	1.97E-05	3.07E-04
**SOX2**	-0.79	0.58	1.97E-05	3.07E-04
**RABGGTB**	0.37	1.29	1.98E-05	3.07E-04
**DSE**	0.28	1.21	1.98E-05	3.07E-04
**KIF2C**	0.30	1.23	2.00E-05	3.11E-04
**PPP1R1B**	-0.79	0.58	2.01E-05	3.12E-04
**TRAF3**	0.36	1.28	2.06E-05	3.19E-04
**ZNF579**	-0.68	0.62	2.06E-05	3.19E-04
**ADGRG1**	-0.33	0.79	2.09E-05	3.23E-04
**PLCD3**	-0.35	0.78	2.10E-05	3.24E-04
**TFEB**	-0.57	0.67	2.11E-05	3.25E-04
**WWTR1**	0.35	1.27	2.12E-05	3.27E-04
**CDK5R1**	-0.40	0.76	2.12E-05	3.27E-04
**LEPROT**	0.36	1.28	2.18E-05	3.34E-04
**APOL1**	-0.78	0.58	2.21E-05	3.39E-04
**B3GNT2**	0.35	1.28	2.21E-05	3.40E-04
**STAT2**	-0.42	0.75	2.23E-05	3.42E-04
**VEZT**	0.31	1.24	2.27E-05	3.48E-04
**RNF225**	-0.78	0.58	2.28E-05	3.49E-04
**ARSI**	-0.67	0.63	2.29E-05	3.50E-04
**ASXL1**	0.32	1.25	2.29E-05	3.50E-04
**GPATCH8**	0.31	1.24	2.30E-05	3.51E-04
**TMEM2**	0.39	1.31	2.34E-05	3.55E-04
**PKMYT1**	-0.42	0.75	2.34E-05	3.56E-04
**KIF5B**	0.29	1.23	2.35E-05	3.57E-04
**EWSR1**	0.26	1.20	2.35E-05	3.57E-04
**MBTPS1**	0.28	1.22	2.40E-05	3.64E-04
**CMIP**	0.31	1.24	2.41E-05	3.65E-04
**TTC39A**	-0.60	0.66	2.42E-05	3.66E-04
**ODF2**	0.29	1.23	2.44E-05	3.68E-04
**CMTR2**	0.38	1.30	2.44E-05	3.68E-04
**WDR59**	0.37	1.29	2.44E-05	3.68E-04
**OTX1**	-0.67	0.63	2.46E-05	3.70E-04
**RAB3D**	-0.32	0.80	2.47E-05	3.71E-04
**IRX3**	-0.43	0.74	2.49E-05	3.73E-04
**TMEM160**	-0.71	0.61	2.49E-05	3.74E-04
**ZNF655**	0.35	1.28	2.55E-05	3.81E-04
**TMCO4**	-0.54	0.69	2.55E-05	3.81E-04
**TTC30B**	-0.69	0.62	2.59E-05	3.87E-04
**DAPK3**	0.44	1.35	2.59E-05	3.87E-04
**SLC6A11**	-0.37	0.77	2.61E-05	3.88E-04
**PI4KA**	0.35	1.28	2.61E-05	3.88E-04
**SLC35F2**	0.33	1.26	2.62E-05	3.90E-04
**DNTTIP2**	0.32	1.25	2.63E-05	3.91E-04
**NID1**	-0.56	0.68	2.67E-05	3.97E-04
**RPS6KA4**	-0.37	0.77	2.68E-05	3.97E-04
**EPB41L5**	0.38	1.30	2.72E-05	4.03E-04
**NCSTN**	-0.33	0.80	2.73E-05	4.04E-04
**PHACTR2**	0.38	1.31	2.76E-05	4.08E-04
**CLSTN1**	0.25	1.19	2.77E-05	4.08E-04
**TTC17**	0.37	1.29	2.79E-05	4.11E-04
**FAM91A1**	0.34	1.27	2.79E-05	4.11E-04
**KIAA1217**	-0.33	0.79	2.80E-05	4.13E-04
**AUTS2**	0.40	1.32	2.81E-05	4.13E-04
**USP36**	0.34	1.27	2.83E-05	4.15E-04
**ITGB6**	-0.36	0.78	2.84E-05	4.16E-04
**MLXIP**	0.35	1.28	2.84E-05	4.17E-04
**CCDC50**	0.33	1.25	2.85E-05	4.17E-04
**NEK2**	0.44	1.36	2.86E-05	4.18E-04
**TRIP13**	0.30	1.23	2.87E-05	4.20E-04
**RPARP-AS1**	-0.77	0.59	2.92E-05	4.26E-04
**SAFB2**	0.34	1.27	3.00E-05	4.37E-04
**MTRF1**	0.62	1.53	3.03E-05	4.41E-04
**PCK2**	-0.38	0.77	3.04E-05	4.43E-04
**HDAC5**	-0.46	0.73	3.07E-05	4.47E-04
**TAF3**	0.45	1.37	3.09E-05	4.48E-04
**ACAA2**	-0.60	0.66	3.10E-05	4.49E-04
**CCNF**	-0.39	0.76	3.12E-05	4.52E-04
**SLC43A2**	-0.62	0.65	3.13E-05	4.53E-04
**MTHFD1**	-0.27	0.83	3.15E-05	4.55E-04
**NF2**	0.35	1.27	3.15E-05	4.55E-04
**OAS3**	-0.30	0.81	3.17E-05	4.58E-04
**FLG**	-0.70	0.62	3.18E-05	4.58E-04
**UBA2**	0.34	1.26	3.19E-05	4.59E-04
**LRCH1**	0.39	1.31	3.21E-05	4.61E-04
**PDZD8**	0.35	1.27	3.21E-05	4.61E-04
**GALNT5**	-0.32	0.80	3.21E-05	4.61E-04
**TPD52L2**	0.26	1.19	3.25E-05	4.65E-04
**ZNF598**	0.43	1.34	3.27E-05	4.68E-04
**LTBP1**	-0.29	0.82	3.28E-05	4.69E-04
**UBC**	-0.25	0.84	3.30E-05	4.72E-04
**ATG4B**	0.35	1.28	3.31E-05	4.73E-04
**TNIP2**	0.39	1.31	3.34E-05	4.76E-04
**MALT1**	0.35	1.28	3.36E-05	4.79E-04
**PLA2G2F**	-0.77	0.59	3.38E-05	4.81E-04
**05-sep**	-0.58	0.67	3.40E-05	4.83E-04
**EPHB6**	-0.76	0.59	3.42E-05	4.86E-04
**OSBPL7**	-0.67	0.63	3.46E-05	4.90E-04
**MVK**	-0.35	0.78	3.46E-05	4.90E-04
**FNBP1**	0.36	1.29	3.47E-05	4.90E-04
**RNF138**	0.56	1.47	3.22E-07	4.99E-06
**FAM198B**	-0.96	0.51	3.23E-07	5.00E-06
**LSM11**	0.70	1.62	3.23E-07	5.00E-06
**ADAM10**	0.35	1.27	3.26E-07	5.04E-06
**ATG16L1**	0.44	1.35	3.30E-07	5.09E-06
**ARHGEF34P**	-0.63	0.65	3.32E-07	5.12E-06
**GOLGA8B**	-0.54	0.69	3.46E-07	5.33E-06
**INIP**	0.50	1.42	3.59E-07	5.52E-06
**IREB2**	0.40	1.32	3.65E-07	5.60E-06
**TRIP13**	0.55	1.47	3.65E-07	5.60E-06
**DNAJB11**	0.40	1.32	3.73E-07	5.72E-06
**DCAF5**	-0.40	0.76	3.76E-07	5.76E-06
**FAM53C**	-0.46	0.72	3.82E-07	5.84E-06
**ZMAT2**	-0.42	0.75	3.86E-07	5.90E-06
**MROH1**	-0.55	0.68	3.89E-07	5.94E-06
**MFSD3**	-0.57	0.67	3.90E-07	5.95E-06
**DTL**	0.53	1.44	3.94E-07	6.00E-06
**AMOTL2**	-0.34	0.79	4.04E-07	6.15E-06
**GGH**	0.46	1.37	4.05E-07	6.15E-06
**PRPF6**	0.38	1.30	4.08E-07	6.19E-06
**ACVR2B**	1.03	2.04	4.09E-07	6.20E-06
**FZD6**	0.36	1.29	4.14E-07	6.27E-06
**CCNC**	0.39	1.31	4.20E-07	6.35E-06
**ACVR1B**	-0.45	0.73	4.20E-07	6.35E-06
**RP11-332M2.1**	-1.20	0.44	4.22E-07	6.36E-06
**RHPN2**	0.69	1.62	4.28E-07	6.45E-06
**ZCCHC24**	1.28	2.43	4.28E-07	6.45E-06
**RUVBL2**	0.38	1.30	4.31E-07	6.49E-06
**NCAPG2**	0.51	1.42	4.39E-07	6.58E-06
**BICD2**	-0.35	0.78	4.39E-07	6.58E-06
**CKS1B**	0.47	1.38	4.45E-07	6.68E-06
**APOBEC3B**	0.90	1.86	4.52E-07	6.77E-06
**CHAF1A**	0.46	1.38	4.67E-07	6.99E-06
**LLGL2**	-0.43	0.74	4.69E-07	7.01E-06
**ZFP91**	-0.46	0.73	4.80E-07	7.17E-06
**CYR61**	-0.36	0.78	4.99E-07	7.44E-06
**KAT2A**	-0.41	0.75	5.00E-07	7.45E-06
**NUMA1**	-0.34	0.79	5.01E-07	7.45E-06
**SGOL2**	0.60	1.51	5.08E-07	7.55E-06
**TP53INP2**	-0.72	0.61	5.20E-07	7.72E-06
**DRG1**	0.40	1.32	5.23E-07	7.75E-06
**MLEC**	-0.38	0.77	5.23E-07	7.75E-06
**TYRO3**	0.52	1.43	5.31E-07	7.85E-06
**MCM10**	0.60	1.52	5.31E-07	7.85E-06
**BCL9**	-0.69	0.62	5.32E-07	7.85E-06
**FAM167A**	0.94	1.92	5.35E-07	7.88E-06
**TBL1XR1**	-0.37	0.77	5.41E-07	7.97E-06
**SECTM1**	1.10	2.14	5.50E-07	8.08E-06
**KNSTRN**	0.51	1.43	5.50E-07	8.08E-06
**FBLN2**	0.86	1.82	5.59E-07	8.20E-06
**SLC10A3**	-0.51	0.70	5.61E-07	8.22E-06
**PLEKHG5**	-0.39	0.77	5.69E-07	8.34E-06
**ALDH3B2**	-0.53	0.69	5.74E-07	8.40E-06
**PLOD2**	0.46	1.38	5.81E-07	8.50E-06
**TCP11L1**	-0.45	0.73	5.98E-07	8.73E-06
**NACC1**	-0.44	0.74	6.19E-07	9.03E-06
**VPS39**	-0.39	0.76	6.34E-07	9.23E-06
**KBTBD6**	0.59	1.50	6.34E-07	9.23E-06
**C1QTNF6**	-0.49	0.71	6.35E-07	9.23E-06
**DONSON**	0.57	1.48	6.38E-07	9.26E-06
**FAM63B**	0.42	1.33	6.49E-07	9.41E-06
**ZYX**	-0.37	0.78	6.59E-07	9.55E-06
**TRIM47**	0.76	1.69	6.62E-07	9.59E-06
**B3GNT5**	0.47	1.38	6.73E-07	9.74E-06
**UTP18**	0.45	1.36	6.90E-07	9.96E-06
**ARHGEF19**	0.48	1.39	6.93E-07	1.00E-05
**SLC25A13**	0.41	1.33	6.94E-07	1.00E-05
**B4GALT5**	0.37	1.29	6.97E-07	1.00E-05
**NPRL3**	-0.55	0.68	7.02E-07	1.01E-05
**XPO1**	0.35	1.27	7.03E-07	1.01E-05
**CA5BP1**	-0.82	0.57	7.06E-07	1.01E-05
**OSBPL1A**	-0.44	0.74	7.14E-07	1.02E-05
**ATP8B1**	0.41	1.32	7.14E-07	1.02E-05
**BLMH**	0.53	1.45	7.32E-07	1.05E-05
**TRIM23**	0.62	1.53	7.52E-07	1.07E-05
**RANBP1**	0.40	1.32	7.53E-07	1.07E-05
**FAM214A**	-0.63	0.65	7.56E-07	1.08E-05
**MTDH**	0.33	1.26	7.76E-07	1.11E-05
**DBP**	0.83	1.78	7.86E-07	1.12E-05
**RABIF**	-0.69	0.62	7.88E-07	1.12E-05
**ITM2A**	-0.48	0.72	7.91E-07	1.12E-05
**WDR37**	0.46	1.37	8.07E-07	1.14E-05
**ZWINT**	0.47	1.38	8.16E-07	1.16E-05
**CNPPD1**	-0.42	0.75	8.23E-07	1.17E-05
**SLC35B2**	-0.42	0.75	8.32E-07	1.18E-05
**GABBR1**	-0.44	0.74	8.34E-07	1.18E-05
**PSPC1**	0.47	1.39	8.44E-07	1.19E-05
**ORC1**	0.61	1.52	8.79E-07	1.24E-05
**PTGS1**	0.76	1.69	8.86E-07	1.25E-05
**PHF8**	-0.43	0.74	8.88E-07	1.25E-05

**MAP3K9**	0.31	1.24	3.48E-05	4.91E-04
**HYOU1**	0.26	1.20	3.48E-05	4.91E-04
**SH3RF2**	0.31	1.24	3.48E-05	4.91E-04
**SPRY1**	-0.74	0.60	3.55E-05	5.00E-04
**CBX2**	-0.47	0.72	3.58E-05	5.04E-04
**ORC1**	-0.39	0.76	3.61E-05	5.07E-04
**PIR**	-0.68	0.62	3.69E-05	5.19E-04
**TGOLN2**	0.26	1.20	3.82E-05	5.36E-04
**CHSY1**	0.34	1.27	3.83E-05	5.37E-04
**NFKBIZ**	0.45	1.37	3.84E-05	5.38E-04
**GABPB2**	-0.48	0.72	3.85E-05	5.39E-04
**HAGHL**	-0.71	0.61	3.86E-05	5.40E-04
**GPR68**	-0.74	0.60	3.90E-05	5.45E-04
**MAP7**	0.32	1.25	3.91E-05	5.46E-04
**ANKLE2**	0.30	1.23	3.93E-05	5.48E-04
**FBXW11**	0.34	1.27	3.97E-05	5.51E-04
**CRTC2**	0.38	1.30	3.97E-05	5.51E-04
**NFIX**	-0.32	0.80	3.98E-05	5.52E-04
**KCNG1**	-0.46	0.73	3.99E-05	5.53E-04
**PLXNA2**	0.31	1.24	3.99E-05	5.53E-04
**ENKD1**	-0.59	0.67	4.00E-05	5.54E-04
**FOXD2-AS1**	-0.76	0.59	4.13E-05	5.72E-04
**PARD3**	0.35	1.27	4.14E-05	5.72E-04
**NTSR1**	-0.65	0.64	4.14E-05	5.72E-04
**TMEM180**	-0.64	0.64	4.16E-05	5.73E-04
**PAK2**	0.30	1.23	4.20E-05	5.79E-04
**WASF3**	0.47	1.38	4.22E-05	5.81E-04
**ZNF488**	-0.64	0.64	4.23E-05	5.82E-04
**FXR2**	0.34	1.27	4.25E-05	5.84E-04
**PLK4**	0.39	1.31	4.27E-05	5.86E-04
**CREBBP**	0.38	1.30	4.28E-05	5.87E-04
**CUL1**	0.31	1.24	4.29E-05	5.88E-04
**DCP1B**	-0.54	0.69	4.32E-05	5.90E-04
**FABP5**	-0.38	0.77	4.39E-05	6.00E-04
**HIST2H2BE**	0.65	1.57	4.49E-05	6.12E-04
**ECI1**	-0.43	0.74	4.53E-05	6.18E-04
**HPS6**	-0.39	0.77	4.54E-05	6.19E-04
**RAB36**	-0.61	0.65	4.57E-05	6.22E-04
**TK1**	-0.40	0.76	4.58E-05	6.23E-04
**YRDC**	0.50	1.41	4.62E-05	6.28E-04
**ATP2A2**	0.22	1.16	4.62E-05	6.28E-04
**DDX54**	0.33	1.25	4.64E-05	6.29E-04
**ZNF609**	0.28	1.22	4.67E-05	6.32E-04
**ELK3**	0.26	1.20	4.80E-05	6.49E-04
**TUBB**	-0.26	0.83	4.80E-05	6.49E-04
**NUF2**	0.42	1.34	4.93E-05	6.65E-04
**DNAJC11**	0.29	1.23	4.94E-05	6.65E-04
**MSLN**	-0.39	0.77	4.94E-05	6.65E-04
**DHX34**	0.39	1.31	4.94E-05	6.65E-04
**PLAU**	0.31	1.24	4.95E-05	6.66E-04
**LAMC2**	0.22	1.17	5.01E-05	6.74E-04
**PKP2**	0.27	1.21	5.08E-05	6.82E-04
**RDX**	0.29	1.22	5.09E-05	6.83E-04
**BUB1B**	0.30	1.23	5.10E-05	6.83E-04
**DROSHA**	0.29	1.22	5.10E-05	6.83E-04
**KRT6B**	-0.25	0.84	5.16E-05	6.90E-04
**RAB27A**	-0.55	0.69	5.17E-05	6.90E-04
**KCNK1**	0.42	1.34	5.19E-05	6.92E-04
**IMPA1**	0.48	1.40	5.19E-05	6.92E-04
**KIAA1211L**	-0.63	0.65	5.19E-05	6.92E-04
**BCAP29**	0.39	1.31	5.23E-05	6.96E-04
**TCF25**	0.33	1.26	5.23E-05	6.96E-04
**MVD**	-0.27	0.83	5.30E-05	7.04E-04
**PRPF3**	0.33	1.26	5.39E-05	7.15E-04
**EHD3**	-0.55	0.68	5.41E-05	7.17E-04
**OSTM1**	0.47	1.38	5.42E-05	7.18E-04
**NCAPH**	0.31	1.24	5.44E-05	7.20E-04
**TCF4**	-0.51	0.70	5.46E-05	7.21E-04
**FKBP11**	-0.53	0.69	5.48E-05	7.23E-04
**PRKAG2**	0.37	1.29	5.49E-05	7.24E-04
**NXT1**	-0.47	0.72	5.50E-05	7.24E-04
**ARID5B**	-0.52	0.70	5.54E-05	7.29E-04
**PTPN14**	0.30	1.23	5.61E-05	7.38E-04
**POLR1D**	0.28	1.21	5.62E-05	7.39E-04
**CASP8AP2**	-0.42	0.75	5.63E-05	7.39E-04
**SRGAP2**	0.33	1.25	5.64E-05	7.39E-04
**NDRG2**	-0.31	0.80	5.64E-05	7.39E-04
**NFATC2**	-0.69	0.62	5.67E-05	7.41E-04
**PABPC1L**	0.56	1.48	5.76E-05	7.53E-04
**ITGB1**	0.24	1.18	5.77E-05	7.53E-04
**WDR1**	0.26	1.19	5.79E-05	7.55E-04
**PAPD7**	0.33	1.26	5.80E-05	7.55E-04
**MTA1**	0.33	1.25	5.80E-05	7.55E-04
**ZNF57**	-0.61	0.65	5.84E-05	7.59E-04
**AMIGO2**	-0.41	0.75	5.90E-05	7.66E-04
**RP11-539I5.1**	-0.65	0.64	5.95E-05	7.72E-04
**SLC6A9**	-0.39	0.76	5.98E-05	7.75E-04
**DNAJB6**	0.31	1.24	5.98E-05	7.76E-04
**CDHR1**	-0.73	0.60	6.01E-05	7.79E-04
**USP45**	0.47	1.39	6.04E-05	7.81E-04
**LSM14A**	0.35	1.27	8.90E-07	1.25E-05
**RFX5**	-0.49	0.71	8.94E-07	1.25E-05
**SH3D19**	-0.39	0.76	8.96E-07	1.26E-05
**MZT1**	0.55	1.46	9.03E-07	1.27E-05
**MIER3**	0.48	1.40	9.07E-07	1.27E-05
**SLC4A11**	-0.45	0.73	9.13E-07	1.28E-05
**RP5-1039K5.12**	-1.05	0.48	9.22E-07	1.29E-05
**SSR1**	0.35	1.28	9.24E-07	1.29E-05
**PSME3**	-0.40	0.76	9.50E-07	1.32E-05
**DDX58**	-0.45	0.73	9.52E-07	1.32E-05
**ERH**	0.43	1.35	9.53E-07	1.32E-05
**HADHA**	-0.35	0.78	9.54E-07	1.32E-05
**RASSF3**	0.54	1.46	9.73E-07	1.35E-05
**SEC23IP**	0.38	1.31	9.74E-07	1.35E-05
**SH3TC1**	0.53	1.44	9.74E-07	1.35E-05
**RBCK1**	-0.33	0.79	9.84E-07	1.36E-05
**INPP5D**	-2.14	0.23	9.87E-07	1.36E-05
**DENND1B**	0.52	1.44	9.94E-07	1.37E-05
**CLIC3**	-0.59	0.66	1.00E-06	1.39E-05
**SYVN1**	-0.40	0.76	1.02E-06	1.41E-05
**GMPS**	0.36	1.28	1.06E-06	1.46E-05
**SMARCD1**	-1.39	0.38	1.08E-06	1.48E-05
**LARP4**	-0.40	0.76	1.08E-06	1.48E-05
**MAPKBP1**	-0.38	0.77	1.10E-06	1.50E-05
**KLK8**	-0.44	0.74	1.10E-06	1.50E-05
**MFAP2**	0.49	1.40	1.11E-06	1.52E-05
**SHPRH**	-0.53	0.69	1.11E-06	1.52E-05
**MCM7**	0.48	1.40	1.12E-06	1.53E-05
**SLC39A10**	0.46	1.37	1.15E-06	1.57E-05
**CALR**	0.36	1.28	1.16E-06	1.58E-05
**ACSS2**	-0.37	0.78	1.16E-06	1.58E-05
**PLEKHM1P**	-0.49	0.71	1.19E-06	1.62E-05
**SLC4A7**	0.37	1.29	1.19E-06	1.62E-05
**THSD4**	-0.37	0.77	1.20E-06	1.63E-05
**FAM73A**	0.45	1.37	1.20E-06	1.63E-05
**FOSL2**	-0.33	0.80	1.21E-06	1.63E-05
**POM121C**	-0.55	0.68	1.22E-06	1.65E-05
**CTNNAL1**	0.53	1.44	1.22E-06	1.65E-05
**DOT1L**	-0.56	0.68	1.23E-06	1.66E-05
**LPIN3**	-0.44	0.74	1.23E-06	1.66E-05
**RAD51D**	-0.82	0.57	1.25E-06	1.68E-05
**BLM**	0.58	1.50	1.35E-06	1.81E-05
**ZCCHC9**	-0.60	0.66	1.35E-06	1.81E-05
**PLEKHM1**	-0.41	0.75	1.37E-06	1.85E-05
**ATAD2**	0.42	1.34	1.38E-06	1.85E-05
**BRWD3**	0.40	1.32	1.40E-06	1.87E-05
**CENPA**	0.65	1.57	1.40E-06	1.87E-05
**RPA1**	0.35	1.27	1.43E-06	1.91E-05
**MTURN**	-0.53	0.69	1.45E-06	1.94E-05
**LRP6**	0.43	1.35	1.46E-06	1.95E-05
**DDIT4**	0.35	1.28	1.46E-06	1.95E-05
**GPR180**	0.42	1.34	1.46E-06	1.95E-05
**PCLO**	-0.60	0.66	1.46E-06	1.95E-05
**PPP2R4**	-0.40	0.76	1.47E-06	1.95E-05
**TNFAIP1**	-0.34	0.79	1.48E-06	1.97E-05
**EFCAB14**	0.34	1.26	1.50E-06	1.99E-05
**NMD3**	0.38	1.30	1.50E-06	1.99E-05
**CDKN3**	0.62	1.54	1.50E-06	1.99E-05
**RNF26**	0.45	1.37	1.53E-06	2.03E-05
**RPS6KB1**	0.42	1.34	1.54E-06	2.03E-05
**CACUL1**	0.38	1.30	1.57E-06	2.07E-05
**TINCR**	-0.36	0.78	1.59E-06	2.09E-05
**SVIL-AS1**	-0.55	0.68	1.59E-06	2.09E-05
**RNF43**	-0.44	0.74	1.59E-06	2.09E-05
**ZNF783**	-0.55	0.68	1.60E-06	2.11E-05
**ID3**	-0.37	0.78	1.62E-06	2.13E-05
**MIR34AHG**	-0.77	0.59	1.63E-06	2.14E-05
**ATG16L2**	-0.45	0.73	1.63E-06	2.14E-05
**MPHOSPH6**	-0.50	0.71	1.63E-06	2.14E-05
**APPL1**	0.39	1.31	1.64E-06	2.14E-05
**RP11-421F16.3**	-0.62	0.65	1.68E-06	2.21E-05
**TIMELESS**	0.46	1.37	1.71E-06	2.24E-05
**CD82**	-0.38	0.77	1.72E-06	2.25E-05
**ADGRF4**	-0.42	0.75	1.72E-06	2.25E-05
**PDPR**	0.43	1.35	1.73E-06	2.26E-05
**DKK1**	-0.48	0.71	1.75E-06	2.28E-05
**DIP2A**	-0.36	0.78	1.77E-06	2.31E-05
**RP4-794I6.4**	-1.57	0.34	1.77E-06	2.31E-05
**LINC01133**	-0.45	0.73	1.79E-06	2.32E-05
**GANC**	-0.44	0.74	1.80E-06	2.33E-05
**RARS**	0.34	1.27	1.82E-06	2.37E-05
**NIPAL2**	-0.42	0.75	1.86E-06	2.40E-05
**ORC3**	0.43	1.35	1.89E-06	2.44E-05
**NINJ1**	-0.55	0.68	1.90E-06	2.45E-05
**REL**	0.48	1.39	1.90E-06	2.46E-05
**RANBP10**	-0.51	0.70	1.91E-06	2.47E-05
**PNLIPRP3**	-0.37	0.77	1.91E-06	2.47E-05
**FXR1**	0.35	1.27	1.98E-06	2.55E-05
**FAM129B**	-0.31	0.81	1.99E-06	2.56E-05
**MICALL2**	-0.37	0.77	2.00E-06	2.57E-05

SENP1	-0.42	0.75	6.06E-05	7.84E-04
SPIRE1	0.38	1.30	6.09E-05	7.87E-04
ZNF407	0.46	1.38	6.10E-05	7.87E-04
RRP1B	0.26	1.19	6.10E-05	7.87E-04
STX16	0.35	1.28	6.12E-05	7.88E-04
CCBE1	0.42	1.34	6.13E-05	7.89E-04
C18orf8	0.45	1.36	6.19E-05	7.96E-04
GMNN	-0.37	0.78	6.20E-05	7.96E-04
PCGF3	0.33	1.26	6.24E-05	8.00E-04
LRRC61	-0.67	0.63	6.28E-05	8.04E-04
ECHDC3	-0.60	0.66	6.29E-05	8.06E-04
VTA1	0.31	1.24	6.36E-05	8.14E-04
RAI14	0.35	1.27	6.40E-05	8.17E-04
AC144652.1	-0.72	0.61	6.40E-05	8.17E-04
RAD51	-0.39	0.76	6.49E-05	8.28E-04
FAM167A	-0.47	0.72	6.50E-05	8.29E-04
KLHL2	0.44	1.36	6.56E-05	8.35E-04
SLC25A44	0.35	1.27	6.58E-05	8.37E-04
LTV1	0.42	1.33	6.60E-05	8.38E-04
ZNF324	-0.59	0.66	6.62E-05	8.41E-04
ACIN1	0.24	1.18	6.75E-05	8.56E-04
MNT	-0.44	0.74	6.78E-05	8.59E-04
EPN3	-0.36	0.78	6.79E-05	8.60E-04
MKNK1	0.35	1.27	6.82E-05	8.63E-04
COMTD1	-0.55	0.68	6.84E-05	8.65E-04
EXOC3	0.34	1.26	6.85E-05	8.65E-04
MGAT5	0.30	1.23	6.86E-05	8.65E-04
MYL12A	0.27	1.21	6.89E-05	8.69E-04
PPP3CB	0.39	1.31	6.91E-05	8.70E-04
ARMC1	0.37	1.29	6.94E-05	8.73E-04
LIPH	-0.42	0.75	6.95E-05	8.74E-04
RBMXL1	-0.42	0.75	6.99E-05	8.77E-04
LATS2	0.42	1.34	7.00E-05	8.78E-04
B4GALT5	-0.28	0.82	7.01E-05	8.78E-04
CKB	-0.54	0.69	7.06E-05	8.84E-04
MANSC1	-0.39	0.76	7.09E-05	8.86E-04
ACPP	-0.70	0.61	7.20E-05	9.00E-04
SYNGAP1	-0.51	0.70	7.22E-05	9.01E-04
CUTA	-0.35	0.79	7.22E-05	9.01E-04
MMP15	-0.48	0.72	7.24E-05	9.02E-04
VPS37B	0.30	1.23	7.29E-05	9.07E-04
BLCAP	0.29	1.23	7.31E-05	9.10E-04
MT-CYB	0.62	1.54	7.35E-05	9.14E-04
TOMM34	0.32	1.25	7.38E-05	9.17E-04
ZNF629	-0.34	0.79	7.53E-05	9.34E-04
FOXQ1	-0.46	0.73	7.54E-05	9.34E-04
VPS13B	0.37	1.29	7.54E-05	9.34E-04
DHX16	0.32	1.25	7.55E-05	9.34E-04
MYC	-0.28	0.82	7.63E-05	9.43E-04
DSG3	-0.24	0.85	7.72E-05	9.53E-04
DVL3	0.29	1.22	7.73E-05	9.54E-04
DBF4B	0.42	1.34	7.76E-05	9.57E-04
ANXA9	-0.52	0.70	7.77E-05	9.57E-04
CCDC88B	-0.57	0.67	7.94E-05	9.77E-04
EIF3A	0.25	1.19	8.01E-05	9.84E-04
CDC42EP5	-0.52	0.70	8.04E-05	9.88E-04
AGMAT	-0.73	0.60	8.05E-05	9.88E-04
DMC1	-0.73	0.60	8.07E-05	9.90E-04
ARHGEF19	-0.47	0.72	8.09E-05	9.91E-04
SLC25A37	0.37	1.29	8.13E-05	9.94E-04
TMEM206	0.48	1.40	8.13E-05	9.94E-04
CCSAP	0.44	1.36	8.19E-05	1.00E-03
KIF3B	0.30	1.23	8.22E-05	1.00E-03
PSMD2	0.24	1.18	8.24E-05	1.00E-03
RDH13	-0.43	0.74	8.31E-05	1.01E-03
CCNB2	0.31	1.24	8.36E-05	1.02E-03
KIAA0907	0.38	1.30	8.48E-05	1.03E-03
UBLCP1	0.37	1.29	8.49E-05	1.03E-03
CDT1	-0.38	0.77	8.50E-05	1.03E-03
SEC23IP	0.30	1.23	8.52E-05	1.03E-03
RRAD	-0.44	0.74	8.64E-05	1.05E-03
PKP1	-0.23	0.85	8.75E-05	1.06E-03
NT5DC3	0.51	1.43	8.75E-05	1.06E-03
TMEM79	-0.30	0.81	8.75E-05	1.06E-03
XPO6	0.28	1.21	8.80E-05	1.06E-03
CCND1	-0.28	0.83	8.80E-05	1.06E-03
GPATCH2L	0.36	1.28	8.84E-05	1.07E-03
ZNF567	0.63	1.55	9.02E-05	1.09E-03
PUS7	0.35	1.28	9.04E-05	1.09E-03
ZNF180	-0.62	0.65	9.06E-05	1.09E-03
PELP1	0.34	1.27	9.13E-05	1.10E-03
PCNX	0.33	1.25	9.22E-05	1.11E-03
MOCOS	-0.45	0.73	9.28E-05	1.11E-03
DDX39A	0.28	1.22	9.28E-05	1.11E-03
NEDD4	0.31	1.24	9.31E-05	1.11E-03
MXRA5	-0.70	0.62	9.34E-05	1.12E-03
PARP10	-0.52	0.70	9.34E-05	1.12E-03
EP300	0.33	1.25	9.39E-05	1.12E-03
HERC6	-0.40	0.76	9.41E-05	1.12E-03
SLC35D2	0.44	1.36	9.49E-05	1.13E-03

CAPNS2	-0.53	0.69	2.01E-06	2.58E-05
F2RL1	-0.40	0.76	2.02E-06	2.59E-05
TUBB4B	0.37	1.30	2.04E-06	2.62E-05
SYPL1	0.32	1.25	2.06E-06	2.64E-05
BNIPL	-0.55	0.69	2.06E-06	2.64E-05
TWISTNB	0.43	1.35	2.11E-06	2.70E-05
CASP7	0.48	1.40	2.13E-06	2.73E-05
TRIM69	-0.50	0.71	2.15E-06	2.74E-05
UBAP1	-0.37	0.77	2.16E-06	2.76E-05
DGCR2	-0.33	0.79	2.17E-06	2.77E-05
CTGF	-0.44	0.73	2.20E-06	2.80E-05
CUL7	-0.47	0.72	2.24E-06	2.85E-05
ARL4C	0.34	1.27	2.24E-06	2.85E-05
KRT10	-0.44	0.74	2.26E-06	2.87E-05
TRIT1	-0.50	0.71	2.26E-06	2.87E-05
ANKRD54	-0.57	0.67	2.28E-06	2.88E-05
ST13	0.32	1.25	2.33E-06	2.95E-05
SSRP1	0.37	1.29	2.36E-06	2.98E-05
GPRC5C	0.88	1.83	2.37E-06	2.99E-05
CHMP6	-0.72	0.61	2.37E-06	2.99E-05
PRR5L	-0.57	0.68	2.42E-06	3.05E-05
KMT2D	-0.39	0.76	2.42E-06	3.05E-05
BCAT1	0.61	1.53	2.43E-06	3.06E-05
JADE2	0.46	1.37	2.47E-06	3.10E-05
GPR155	-0.55	0.68	2.48E-06	3.12E-05
STIP1	0.33	1.26	2.49E-06	3.13E-05
PTPN14	-0.33	0.80	2.50E-06	3.14E-05
SLC35D2	0.56	1.48	2.52E-06	3.16E-05
SOWAHC	0.34	1.27	2.70E-06	3.38E-05
SPRR2D	-0.58	0.67	2.73E-06	3.42E-05
ZDHHC8	-0.41	0.75	2.74E-06	3.43E-05
LGALS3BP	-0.30	0.81	2.76E-06	3.44E-05
DEK	0.40	1.32	2.77E-06	3.45E-05
SNHG14	-0.42	0.75	2.79E-06	3.47E-05
CHD4	0.31	1.24	2.81E-06	3.49E-05
SNRPE	0.43	1.35	2.85E-06	3.55E-05
SOX7	0.45	1.36	2.88E-06	3.57E-05
HOXA9	0.48	1.40	2.89E-06	3.58E-05
PHPT1	-0.40	0.76	2.91E-06	3.60E-05
SMARCA5	0.33	1.25	2.93E-06	3.62E-05
NCR3LG1	-0.67	0.63	2.99E-06	3.70E-05
ISCA2	0.57	1.49	3.03E-06	3.75E-05
HIST1H2AC	-0.62	0.65	3.06E-06	3.78E-05
JARID2	0.40	1.32	3.07E-06	3.79E-05
MOGS	-0.34	0.79	3.11E-06	3.83E-05
TRIM14	-0.37	0.77	3.14E-06	3.86E-05
PRSS23	-0.32	0.80	3.14E-06	3.86E-05
LOXL2	0.94	1.92	3.15E-06	3.88E-05
IPO7	0.31	1.24	3.19E-06	3.91E-05
NCAPH	0.54	1.46	3.20E-06	3.93E-05
PIAS3	-0.40	0.76	3.22E-06	3.95E-05
NFKBIA	0.41	1.33	3.22E-06	3.95E-05
ARRDC4	0.38	1.31	3.24E-06	3.97E-05
UBE2Q1	-0.40	0.76	3.29E-06	4.03E-05
FAM127B	-0.47	0.72	3.30E-06	4.03E-05
TMEM63A	-0.44	0.74	3.32E-06	4.05E-05
CLCN5	0.45	1.36	3.36E-06	4.09E-05
TRAF3IP3	-1.25	0.42	3.37E-06	4.10E-05
PKMYT1	0.50	1.41	3.41E-06	4.15E-05
HNRNPD	0.33	1.26	3.42E-06	4.15E-05
RAD18	0.46	1.37	3.42E-06	4.15E-05
C10orf54	-0.38	0.77	3.47E-06	4.21E-05
MYO10	0.35	1.27	3.47E-06	4.21E-05
NEK7	0.39	1.31	3.50E-06	4.24E-05
NAP1L1	0.31	1.24	3.54E-06	4.28E-05
NIN	0.36	1.28	3.55E-06	4.29E-05
ALYREF	0.41	1.33	3.55E-06	4.29E-05
CSPG4	0.63	1.55	3.56E-06	4.30E-05
CSTF2	0.42	1.34	3.56E-06	4.30E-05
SAMD10	-1.13	0.46	3.57E-06	4.30E-05
CCNJ	-0.79	0.58	3.61E-06	4.35E-05
MMP9	1.36	2.56	3.61E-06	4.35E-05
PAPOLA	0.32	1.25	3.61E-06	4.35E-05
PVRL1	-0.34	0.79	3.64E-06	4.38E-05
CSNK1E	-0.33	0.80	3.65E-06	4.38E-05
GJA5	-1.28	0.41	3.68E-06	4.41E-05
TRADD	-0.47	0.72	3.71E-06	4.45E-05
SEC23A	0.34	1.27	3.80E-06	4.55E-05
PXDN	0.38	1.31	3.90E-06	4.67E-05
ID1	0.41	1.33	3.97E-06	4.75E-05
PSKH1	-0.45	0.73	3.99E-06	4.77E-05
TRNP1	-0.42	0.75	4.01E-06	4.78E-05
ZNF721	0.50	1.41	4.02E-06	4.78E-05
RHOT2	-0.37	0.78	4.02E-06	4.78E-05
CEP128	0.56	1.47	4.02E-06	4.78E-05
CNOT11	0.35	1.28	4.06E-06	4.82E-05
DNAJA1	0.32	1.24	4.12E-06	4.89E-05
WWP2	-0.42	0.75	4.23E-06	5.02E-05
OSBPL5	0.47	1.38	4.25E-06	5.04E-05
HS6ST1	-0.37	0.78	4.25E-06	5.04E-05

CDC42EP3	0.34	1.27	9.57E-05	1.14E-03	KCTD2	-0.47	0.72	4.28E-06	5.06E-05
ATN1	0.33	1.26	9.69E-05	1.15E-03	KIAA1161	-0.41	0.75	4.35E-06	5.14E-05
KRT14	-0.28	0.82	9.71E-05	1.15E-03	TOMM70A	0.34	1.27	4.36E-06	5.15E-05
AJAP1	0.41	1.33	9.72E-05	1.15E-03	LAPTM4A	0.31	1.24	4.40E-06	5.20E-05
RAP1GAP2	0.31	1.24	9.77E-05	1.16E-03	APBB2	-0.35	0.79	4.44E-06	5.24E-05
SLC16A9	-0.50	0.71	9.79E-05	1.16E-03	SUCO	0.48	1.40	4.45E-06	5.24E-05
TSPYL4	-0.51	0.70	9.88E-05	1.17E-03	S100A10	-0.29	0.82	4.45E-06	5.24E-05
RABGAP1L	0.43	1.34	9.93E-05	1.17E-03	ADCY7	0.46	1.38	4.54E-06	5.34E-05
KNSTRN	0.33	1.25	9.94E-05	1.17E-03	ACSL3	0.31	1.24	4.58E-06	5.38E-05
TNFSF9	-0.52	0.70	9.95E-05	1.17E-03	PNRC2	0.60	1.51	4.59E-06	5.39E-05
RBM5	-0.34	0.79	9.97E-05	1.18E-03	RSRP1	-0.42	0.75	4.61E-06	5.40E-05
DBF4	0.38	1.30	9.99E-05	1.18E-03	PRDM1	-0.59	0.66	4.75E-06	5.57E-05
PRKAA1	0.33	1.25	1.01E-04	1.19E-03	SOWAHB	-0.78	0.58	4.77E-06	5.58E-05
MYO6	0.31	1.24	1.03E-04	1.21E-03	CENPQ	0.67	1.59	4.86E-06	5.68E-05
STK24	0.27	1.21	1.04E-04	1.22E-03	SRSF2	0.33	1.26	4.88E-06	5.71E-05
SLC9A3R1	-0.28	0.82	1.05E-04	1.23E-03	LGALS7	-0.38	0.77	4.90E-06	5.72E-05
C14orf1	-0.31	0.81	1.05E-04	1.23E-03	AFG3L2	0.36	1.29	5.01E-06	5.85E-05
PIK3C2A	0.32	1.25	1.05E-04	1.24E-03	GALNT7	0.42	1.33	5.07E-06	5.91E-05
PPTC7	0.28	1.22	1.06E-04	1.24E-03	PRUNE	-0.48	0.71	5.12E-06	5.97E-05
RBM17	0.29	1.22	1.06E-04	1.24E-03	KIAA1191	-0.35	0.79	5.17E-06	6.01E-05
SEC24A	0.38	1.30	1.07E-04	1.25E-03	IDH1	-0.31	0.81	5.17E-06	6.01E-05
SPTBN2	-0.28	0.82	1.09E-04	1.28E-03	KREMEN1	0.34	1.26	5.18E-06	6.01E-05
DHX37	0.33	1.25	1.10E-04	1.29E-03	FEN1	0.38	1.30	5.25E-06	6.09E-05
ARRDC4	0.33	1.26	1.10E-04	1.29E-03	SLAMF7	-0.60	0.66	5.26E-06	6.09E-05
PRKCH	0.38	1.30	1.11E-04	1.29E-03	EFS	-0.46	0.73	5.26E-06	6.09E-05
CEBPZ	0.32	1.25	1.11E-04	1.29E-03	CAMK2G	-0.42	0.75	5.26E-06	6.09E-05
PGAM5	0.28	1.22	1.11E-04	1.30E-03	ATP6V0A1	-0.38	0.77	5.31E-06	6.13E-05
SH3RF1	0.36	1.29	1.11E-04	1.30E-03	DCAKD	-0.53	0.69	5.31E-06	6.13E-05
PBRM1	0.28	1.22	1.12E-04	1.30E-03	TRAP1	0.39	1.31	5.31E-06	6.13E-05
ORMDL1	0.42	1.34	1.12E-04	1.30E-03	PKD1	0.35	1.27	5.39E-06	6.22E-05
IFI27	-0.31	0.81	1.12E-04	1.30E-03	BHLHE41	1.12	2.17	5.45E-06	6.28E-05
HMGCS1	-0.54	0.69	1.14E-04	1.32E-03	PLD2	-0.38	0.77	5.49E-06	6.32E-05
ERCC3	0.30	1.23	1.15E-04	1.33E-03	IL7R	1.18	2.26	5.50E-06	6.33E-05
WDR70	0.38	1.30	1.15E-04	1.33E-03	NRAS	0.37	1.29	5.55E-06	6.38E-05
DDX3X	0.25	1.19	1.15E-04	1.33E-03	EDA2R	-1.02	0.49	5.58E-06	6.42E-05
EGFR	0.23	1.17	1.16E-04	1.34E-03	ESRRA	-0.37	0.77	5.66E-06	6.49E-05
CDK6	0.27	1.21	1.16E-04	1.34E-03	TMX4	0.44	1.36	5.71E-06	6.55E-05
BACE1	-0.39	0.76	1.16E-04	1.34E-03	SMARCC1	0.32	1.25	5.73E-06	6.57E-05
DUOXA1	-0.31	0.81	1.17E-04	1.35E-03	KDM1A	0.33	1.26	5.79E-06	6.63E-05
IRF9	-0.72	0.61	1.17E-04	1.35E-03	LMAN1	0.31	1.24	5.86E-06	6.70E-05
SMTN	0.28	1.22	1.19E-04	1.37E-03	MOB3A	-0.46	0.73	5.87E-06	6.70E-05
PCSK9	-0.28	0.82	1.19E-04	1.37E-03	OAS1	-0.36	0.78	5.88E-06	6.72E-05
RBM23	-0.34	0.79	1.19E-04	1.37E-03	PHACTR4	-0.37	0.78	5.93E-06	6.77E-05
SDR9C7	-0.71	0.61	1.22E-04	1.39E-03	KPNA3	0.34	1.27	5.98E-06	6.82E-05
IVL	-0.21	0.86	1.23E-04	1.41E-03	BTBD2	-0.38	0.77	5.99E-06	6.82E-05
USP22	0.23	1.18	1.23E-04	1.41E-03	SMC2	0.44	1.36	6.00E-06	6.83E-05
ZNF266	0.47	1.39	1.24E-04	1.42E-03	TTLL12	0.35	1.28	6.08E-06	6.91E-05
SRRT	0.26	1.20	1.24E-04	1.42E-03	HNRNPH3	0.33	1.26	6.12E-06	6.96E-05
TET1	-0.70	0.61	1.24E-04	1.42E-03	ODF3B	-0.78	0.58	6.14E-06	6.98E-05
SLAMF7	-0.55	0.68	1.24E-04	1.42E-03	H6PD	-0.37	0.77	6.17E-06	7.00E-05
MTHFD1L	0.26	1.20	1.24E-04	1.42E-03	ARHGAP11B	0.47	1.39	6.27E-06	7.11E-05
GIT1	0.29	1.22	1.25E-04	1.42E-03	VRK1	0.41	1.33	6.28E-06	7.11E-05
CCDC34	-0.49	0.71	1.25E-04	1.42E-03	TMEM64	0.40	1.31	6.29E-06	7.11E-05
MAPKAP1	0.25	1.19	1.25E-04	1.42E-03	IL18	-0.33	0.80	6.32E-06	7.14E-05
VPS37A	0.32	1.25	1.25E-04	1.42E-03	PTER	0.51	1.43	6.32E-06	7.14E-05
BOD1L1	0.31	1.24	1.26E-04	1.43E-03	SNX12	-1.07	0.47	6.33E-06	7.14E-05
PEX11B	-0.40	0.76	1.26E-04	1.43E-03	RNASEH2A	0.47	1.39	6.36E-06	7.17E-05
ECM1	-0.27	0.83	1.26E-04	1.43E-03	CBX6	-0.48	0.72	6.38E-06	7.19E-05
POLD3	-0.34	0.79	1.26E-04	1.43E-03	DGKE	0.56	1.48	6.40E-06	7.21E-05
AKAP8L	0.36	1.28	1.26E-04	1.43E-03	CDS1	0.36	1.29	6.41E-06	7.21E-05
GRIP1	0.53	1.45	1.27E-04	1.44E-03	RP5-1039K5.19	-0.72	0.61	6.47E-06	7.28E-05
TWISTNB	0.36	1.28	1.28E-04	1.45E-03	FAM96A	0.44	1.36	6.50E-06	7.30E-05
SRM	-0.36	0.78	1.29E-04	1.46E-03	USP12	0.41	1.33	6.59E-06	7.39E-05
CGGBP1	0.35	1.28	1.30E-04	1.46E-03	PUDP	0.46	1.37	6.70E-06	7.51E-05
BAZ1A	0.30	1.23	1.30E-04	1.46E-03	ARHGAP11A	0.48	1.40	6.74E-06	7.55E-05
SGSM2	0.37	1.29	1.30E-04	1.47E-03	TMPRSS4	0.50	1.41	6.78E-06	7.58E-05
ZCCHC11	0.34	1.27	1.31E-04	1.47E-03	USP13	0.57	1.49	6.78E-06	7.58E-05
PRR12	-0.37	0.77	1.33E-04	1.49E-03	FAM217B	0.48	1.40	6.82E-06	7.63E-05
CLK3	0.32	1.25	1.35E-04	1.51E-03	PRSS8	-0.37	0.77	6.86E-06	7.66E-05
RAB18	0.31	1.24	1.35E-04	1.52E-03	RP5-884C9.2	-1.80	0.29	6.86E-06	7.66E-05
STAT1	-0.26	0.84	1.38E-04	1.54E-03	GNPDA1	0.40	1.32	6.89E-06	7.68E-05
FZD6	0.30	1.23	1.39E-04	1.55E-03	RCHY1	0.52	1.43	6.91E-06	7.70E-05
MAP3K4	0.31	1.24	1.39E-04	1.55E-03	SPRYD3	-0.47	0.72	6.93E-06	7.71E-05
SUPT20H	0.30	1.24	1.39E-04	1.56E-03	SEC14L1	-0.39	0.76	6.93E-06	7.71E-05
TP53BP2	0.32	1.25	1.40E-04	1.56E-03	BBC3	-1.02	0.49	7.01E-06	7.79E-05
SLC12A4	0.34	1.27	1.40E-04	1.56E-03	JOSD2	-0.53	0.69	7.10E-06	7.88E-05
CDK8	0.35	1.28	1.40E-04	1.56E-03	GATA3	-0.44	0.74	7.11E-06	7.89E-05
PAPD5	0.39	1.31	1.41E-04	1.57E-03	KIF14	0.50	1.42	7.30E-06	8.09E-05
VPRBP	0.30	1.23	1.41E-04	1.58E-03	ZHX3	-0.38	0.77	7.37E-06	8.16E-05
HIST1H2BC	0.71	1.64	1.44E-04	1.60E-03	REXO1	-0.47	0.72	7.38E-06	8.16E-05
AARS	-0.23	0.85	1.44E-04	1.60E-03	TM7SF2	-0.43	0.74	7.38E-06	8.16E-05
SS18	0.27	1.21	1.44E-04	1.60E-03	LDLRAP1	-0.42	0.75	7.43E-06	8.20E-05
REV1	0.37	1.29	1.46E-04	1.62E-03	SLIT2	-0.62	0.65	7.44E-06	8.22E-05
MATN2	0.64	1.56	1.46E-04	1.62E-03	FAM160B1	0.45	1.37	7.49E-06	8.26E-05
ATP8B2	-0.37	0.77	1.48E-04	1.64E-03	PSMC6	0.35	1.27	7.68E-06	8.46E-05
PGLYRP3	-0.47	0.72	1.48E-04	1.64E-03	GOLGB1	-0.32	0.80	7.70E-06	8.48E-05
ILF3-AS1	-0.63	0.64	1.49E-04	1.64E-03	SLC25A15	0.64	1.56	7.71E-06	8.49E-05
MAEA	0.29	1.22	1.49E-04	1.65E-03	ANO9	-0.35	0.78	7.75E-06	8.52E-05
PFDN1	0.30	1.23	1.51E-04	1.67E-03	TMEM63B	-0.33	0.80	7.77E-06	8.53E-05
TEX2	0.25	1.19	1.51E-04	1.67E-03	CDK2AP2	-0.44	0.73	7.85E-06	8.62E-05

RYBP	0.34	1.27	1.51E-04	1.67E-03
DOCK7	0.30	1.23	1.52E-04	1.68E-03
ERBB3	-0.28	0.82	1.52E-04	1.68E-03
CHAF1B	-0.36	0.78	1.53E-04	1.69E-03
HIF1A	0.26	1.20	1.55E-04	1.71E-03
DDX3Y	0.31	1.24	1.55E-04	1.71E-03
PUM2	0.26	1.20	1.55E-04	1.71E-03
GM2A	-0.22	0.86	1.56E-04	1.71E-03
CMSS1	0.33	1.25	1.58E-04	1.73E-03
PODXL2	-0.55	0.68	1.58E-04	1.74E-03
SERPINH1	-0.30	0.81	1.59E-04	1.74E-03
YDJC	-0.39	0.76	1.60E-04	1.75E-03
CEP164	0.32	1.25	1.60E-04	1.75E-03
IFIT3	-0.61	0.66	1.60E-04	1.75E-03
KIF3A	0.38	1.30	1.61E-04	1.75E-03
CTSL	-0.30	0.81	1.61E-04	1.76E-03
PRMT2	0.29	1.22	1.61E-04	1.76E-03
OGT	0.32	1.24	1.61E-04	1.76E-03
PITRM1	0.24	1.18	1.62E-04	1.76E-03
SH2B3	0.38	1.30	1.63E-04	1.77E-03
IL33	-0.63	0.64	1.63E-04	1.77E-03
DNAJB12	0.33	1.26	1.63E-04	1.77E-03
RPL29	-0.23	0.85	1.65E-04	1.79E-03
LTBR	0.28	1.22	1.68E-04	1.82E-03
TSPO	-0.34	0.79	1.68E-04	1.82E-03
TAF2	0.31	1.24	1.68E-04	1.82E-03
KIAA1161	-0.39	0.76	1.69E-04	1.82E-03
UGGT2	0.46	1.38	1.72E-04	1.86E-03
CDC42BPB	0.26	1.20	1.72E-04	1.86E-03
ACTN4	0.25	1.19	1.73E-04	1.86E-03
PNO1	0.34	1.27	1.73E-04	1.87E-03
KLHL18	0.33	1.26	1.74E-04	1.87E-03
IFNGR1	0.34	1.27	1.78E-04	1.91E-03
AFF1	0.30	1.23	1.78E-04	1.91E-03
NSG1	-0.30	0.81	1.79E-04	1.92E-03
UBAP1	0.27	1.21	1.79E-04	1.92E-03
PACS1	0.31	1.24	1.80E-04	1.93E-03
ELAVL2	0.41	1.33	1.80E-04	1.93E-03
NOP14	0.27	1.21	1.81E-04	1.94E-03
PPP3CA	0.29	1.22	1.82E-04	1.94E-03
C1orf174	0.36	1.28	1.83E-04	1.95E-03
XRN2	0.26	1.20	1.83E-04	1.96E-03
FAM195B	0.41	1.33	1.84E-04	1.96E-03
GTF3C6	-0.37	0.77	1.84E-04	1.96E-03
LGALS3BP	-0.25	0.84	1.84E-04	1.96E-03
ALMS1	-0.40	0.76	1.85E-04	1.96E-03
TTC22	-0.43	0.74	1.85E-04	1.97E-03
PIEZO1	0.30	1.23	1.86E-04	1.98E-03
NRARP	-0.45	0.73	1.87E-04	1.98E-03
WRN	0.40	1.32	1.87E-04	1.98E-03
TWSG1	0.34	1.26	1.93E-04	2.04E-03
RAB23	-0.48	0.72	1.93E-04	2.04E-03
SLC6A8	-0.26	0.84	1.93E-04	2.04E-03
CXCL2	0.69	1.61	1.93E-04	2.04E-03
SLC45A3	0.54	1.46	1.94E-04	2.05E-03
MTERF3	0.39	1.31	1.99E-04	2.10E-03
C22orf29	-0.33	0.80	2.01E-04	2.12E-03
CNOT7	0.31	1.24	2.02E-04	2.14E-03
ANO8	-0.54	0.69	2.03E-04	2.14E-03
PRDM15	0.44	1.36	2.04E-04	2.15E-03
FBXO18	0.28	1.22	2.05E-04	2.16E-03
WBP11	0.23	1.18	2.06E-04	2.17E-03
RPF1	0.29	1.23	2.07E-04	2.18E-03
FOSL2	-0.24	0.85	2.08E-04	2.18E-03
ARID1A	-0.28	0.82	2.11E-04	2.21E-03
TXLNGY	0.42	1.34	2.12E-04	2.22E-03
UBE2SP1	0.60	1.52	2.13E-04	2.23E-03
POU2F3	-0.69	0.62	2.14E-04	2.24E-03
SLC25A30	0.38	1.30	2.15E-04	2.25E-03
HECTD1	0.29	1.22	2.16E-04	2.26E-03
PES1	0.25	1.19	2.17E-04	2.26E-03
ALG13	0.42	1.34	2.18E-04	2.27E-03
C20orf24	-0.61	0.66	2.18E-04	2.27E-03
SLF2	-0.35	0.78	2.18E-04	2.27E-03
TTC9	-0.34	0.79	2.19E-04	2.28E-03
BARD1	-0.43	0.74	2.23E-04	2.32E-03
SDR42E1	-0.41	0.75	2.23E-04	2.32E-03
ZNF524	-0.51	0.70	2.25E-04	2.33E-03
KIAA0141	0.35	1.27	2.25E-04	2.34E-03
PPM1D	-0.51	0.70	2.26E-04	2.34E-03
KLC2	0.29	1.23	2.28E-04	2.36E-03
MID1IP1	-0.30	0.81	2.29E-04	2.37E-03
ANTXR1	0.30	1.23	2.30E-04	2.38E-03
NCOA7	0.37	1.29	2.31E-04	2.39E-03
DNM2	0.26	1.19	2.32E-04	2.39E-03
TRAPPC9	0.38	1.30	2.33E-04	2.41E-03
TSPAN5	0.42	1.34	2.35E-04	2.43E-03
NGRN	-0.40	0.76	2.35E-04	2.43E-03
MTUS1	-0.35	0.79	2.36E-04	2.43E-03
04-mar	0.43	1.35	2.36E-04	2.43E-03

EXO1	0.59	1.50	7.86E-06	8.62E-05
CKAP2L	0.50	1.41	7.89E-06	8.65E-05
PLK4	0.53	1.45	8.06E-06	8.83E-05
KARS	0.32	1.25	8.07E-06	8.83E-05
HNRNPC	0.31	1.24	8.10E-06	8.85E-05
TMEM164	-0.36	0.78	8.12E-06	8.87E-05
RAB21	-0.41	0.75	8.13E-06	8.87E-05
IP6K2	-0.35	0.78	8.13E-06	8.87E-05
IBTK	0.34	1.27	8.15E-06	8.88E-05
KRT16P5	-0.59	0.67	8.16E-06	8.88E-05
KBTBD8	0.67	1.60	8.20E-06	8.92E-05
ESPL1	0.46	1.38	8.24E-06	8.96E-05
ILF2	0.32	1.25	8.36E-06	9.08E-05
CLCN3	0.36	1.28	8.43E-06	9.15E-05
CNOT7	0.33	1.26	8.45E-06	9.16E-05
PRKY	-0.48	0.71	8.52E-06	9.23E-05
RFC4	0.46	1.37	8.61E-06	9.32E-05
FAM83D	0.40	1.32	8.75E-06	9.46E-05
FUBP3	0.34	1.26	8.86E-06	9.58E-05
ZMYND11	0.35	1.27	8.95E-06	9.66E-05
KDELR1	-0.31	0.81	8.99E-06	9.70E-05
PTPN3	0.34	1.27	9.02E-06	9.72E-05
HEG1	0.37	1.29	9.07E-06	9.78E-05
APH1B	0.62	1.54	9.10E-06	9.80E-05
C10orf99	-0.79	0.58	9.14E-06	9.83E-05
RAET1G	-0.43	0.74	9.24E-06	9.94E-05
UBE2T	0.49	1.40	9.26E-06	9.95E-05
ARPC1A	0.31	1.24	9.35E-06	1.00E-04
FBXW11	-0.36	0.78	9.37E-06	1.01E-04
NOLC1	0.32	1.25	9.47E-06	1.01E-04
BAG6	-0.32	0.80	9.77E-06	1.05E-04
PML	-0.39	0.76	9.86E-06	1.06E-04
USP21	-0.47	0.72	9.90E-06	1.06E-04
CAPZB	-0.33	0.80	9.92E-06	1.06E-04
PI4K2A	-0.38	0.77	9.97E-06	1.06E-04
DHX58	-0.51	0.70	1.00E-05	1.07E-04
RCBTB1	-0.39	0.76	1.02E-05	1.08E-04
RFC2	0.42	1.34	1.03E-05	1.10E-04
NECAP1	0.40	1.32	1.03E-05	1.10E-04
CHEK1	0.50	1.42	1.03E-05	1.10E-04
ARL8A	-0.43	0.74	1.03E-05	1.10E-04
RHCG	-0.41	0.75	1.04E-05	1.10E-04
BAX	-0.36	0.78	1.05E-05	1.11E-04
TK1	0.41	1.33	1.06E-05	1.12E-04
TP53INP1	-0.47	0.72	1.06E-05	1.12E-04
BIRC3	0.74	1.67	1.06E-05	1.12E-04
TMCO3	0.35	1.27	1.07E-05	1.13E-04
ANPEP	0.73	1.66	1.07E-05	1.13E-04
LPAR5	-0.40	0.76	1.08E-05	1.14E-04
IL1A	-0.35	0.78	1.09E-05	1.15E-04
ZDHHC5	-0.30	0.81	1.10E-05	1.16E-04
ATP6V0E2	-0.75	0.59	1.11E-05	1.17E-04
VGLL1	-0.71	0.61	1.11E-05	1.17E-04
SLC2A4RG	0.36	1.28	1.11E-05	1.17E-04
DNA2	0.52	1.44	1.14E-05	1.20E-04
KCNN4	0.74	1.67	1.14E-05	1.20E-04
SASS6	0.58	1.50	1.16E-05	1.22E-04
DUSP6	-0.47	0.72	1.16E-05	1.22E-04
SLC16A9	0.74	1.67	1.17E-05	1.23E-04
DKC1	0.32	1.25	1.18E-05	1.23E-04
SSBP3	0.38	1.30	1.19E-05	1.24E-04
GALNT5	-0.31	0.81	1.19E-05	1.24E-04
FBXW8	-0.52	0.70	1.20E-05	1.26E-04
MAPK8IP3	-0.39	0.76	1.21E-05	1.26E-04
PDIA6	0.33	1.26	1.21E-05	1.26E-04
ZNF681	0.74	1.66	1.21E-05	1.26E-04
TMEM87B	0.35	1.28	1.23E-05	1.28E-04
TOR2A	-0.67	0.63	1.23E-05	1.28E-04
VPS54	0.37	1.29	1.23E-05	1.28E-04
RCN1	0.38	1.30	1.25E-05	1.30E-04
ETS1	0.37	1.29	1.27E-05	1.32E-04
CDC25C	0.77	1.71	1.27E-05	1.32E-04
FAM83A	-0.35	0.78	1.27E-05	1.32E-04
ITGB4	-0.34	0.79	1.28E-05	1.32E-04
MAP3K10	-0.50	0.71	1.29E-05	1.34E-04
GPRIN1	-0.47	0.72	1.30E-05	1.34E-04
SLC9A3R2	0.56	1.47	1.30E-05	1.34E-04
THRA	0.54	1.46	1.30E-05	1.34E-04
SMARCAD1	0.35	1.28	1.31E-05	1.35E-04
HS6ST2	0.77	1.71	1.32E-05	1.36E-04
SRRM1	-0.42	0.75	1.34E-05	1.38E-04
COL27A1	-0.34	0.79	1.34E-05	1.38E-04
CCT2	0.31	1.24	1.34E-05	1.38E-04
ST6GAL1	-0.48	0.72	1.35E-05	1.39E-04
ISG20L2	-0.39	0.76	1.38E-05	1.41E-04
DEPDC7	-0.40	0.76	1.40E-05	1.44E-04
NHSL1	-0.40	0.76	1.42E-05	1.45E-04
SSNA1	-0.41	0.75	1.42E-05	1.45E-04
HNRNPA1	0.28	1.21	1.43E-05	1.46E-04
HSPA9	0.29	1.22	1.43E-05	1.47E-04

NETO2	0.26	1.20	2.38E-04	2.45E-03
SENP6	0.34	1.26	2.40E-04	2.47E-03
BCOR	-0.38	0.77	2.41E-04	2.47E-03
PPP1R3D	-0.50	0.71	2.45E-04	2.51E-03
EML3	-0.40	0.76	2.46E-04	2.52E-03
SPRED2	0.29	1.23	2.48E-04	2.54E-03
DHX15	0.23	1.17	2.49E-04	2.55E-03
NUDT15	0.30	1.23	2.49E-04	2.55E-03
NAT14	-0.59	0.66	2.50E-04	2.55E-03
CKMT1A	-0.40	0.76	2.52E-04	2.57E-03
TRNP1	0.31	1.24	2.52E-04	2.57E-03
EBP	-0.30	0.81	2.53E-04	2.58E-03
ARHGEF7	0.27	1.21	2.53E-04	2.58E-03
RIC1	0.34	1.27	2.55E-04	2.60E-03
MRPS16	-0.29	0.82	2.56E-04	2.60E-03
CDCA4	-0.33	0.79	2.56E-04	2.60E-03
TES	0.26	1.20	2.59E-04	2.63E-03
PLEKHH3	-0.38	0.77	2.60E-04	2.64E-03
AATF	0.26	1.19	2.60E-04	2.64E-03
ARNTL	0.37	1.30	2.61E-04	2.65E-03
KRI1	0.33	1.26	2.62E-04	2.65E-03
TAF5L	0.30	1.23	2.62E-04	2.66E-03
JUND	-0.41	0.75	2.63E-04	2.66E-03
DCP1A	0.34	1.27	2.64E-04	2.67E-03
GPCPD1	-0.47	0.72	2.66E-04	2.68E-03
AMPD3	-0.35	0.79	2.67E-04	2.69E-03
SUOX	-0.49	0.71	2.68E-04	2.70E-03
JPH2	-0.55	0.68	2.69E-04	2.71E-03
NAV3	0.47	1.38	2.70E-04	2.72E-03
YES1	0.34	1.26	2.71E-04	2.73E-03
MLLT1	0.29	1.22	2.72E-04	2.73E-03
PACSIN2	0.26	1.20	2.73E-04	2.75E-03
CSRP2	-0.48	0.72	2.75E-04	2.76E-03
SEMA5A	-0.67	0.63	2.75E-04	2.76E-03
IDH3A	0.32	1.25	2.76E-04	2.77E-03
SLC36A1	0.41	1.33	2.76E-04	2.77E-03
FKBP5	-0.26	0.84	2.76E-04	2.77E-03
FYN	0.30	1.23	2.77E-04	2.77E-03
LAD1	-0.25	0.84	2.77E-04	2.77E-03
ST6GAL1	-0.44	0.74	2.78E-04	2.78E-03
VEGFA	-0.26	0.83	2.80E-04	2.80E-03
PKD2	0.43	1.35	2.81E-04	2.80E-03
SERPINB7	-0.30	0.81	2.81E-04	2.80E-03
CCNE1	-0.39	0.76	2.82E-04	2.80E-03
ADNP2	0.30	1.23	2.84E-04	2.82E-03
SLC2A5	-0.63	0.65	2.84E-04	2.83E-03
OSGEPL1	0.52	1.43	2.87E-04	2.85E-03
TRUB1	0.38	1.30	2.90E-04	2.88E-03
USP8	0.31	1.24	2.91E-04	2.88E-03
PHF20L1	0.33	1.26	2.91E-04	2.88E-03
RAB11FIP3	0.34	1.26	2.91E-04	2.88E-03
CRIP2	-0.55	0.68	2.93E-04	2.90E-03
USP34	0.29	1.23	2.95E-04	2.92E-03
PLEKHG4	-0.47	0.72	2.97E-04	2.94E-03
ARHGEF12	0.25	1.19	2.98E-04	2.95E-03
EPS8L2	-0.26	0.84	2.99E-04	2.95E-03
CYP4F22	-0.67	0.63	2.99E-04	2.95E-03
ZNF76	0.43	1.35	2.99E-04	2.95E-03
ANKMY1	-0.51	0.70	3.00E-04	2.95E-03
MAN2C1	0.33	1.26	3.01E-04	2.96E-03
RPS6KA1	0.28	1.21	3.02E-04	2.97E-03
TRIP6	-0.30	0.81	3.02E-04	2.97E-03
EHBP1	0.30	1.23	3.04E-04	2.98E-03
DOPEY2	0.36	1.29	3.04E-04	2.98E-03
NNT	-0.35	0.79	3.05E-04	2.99E-03
CTSV	-0.24	0.84	3.07E-04	3.01E-03
MDK	-0.50	0.71	3.07E-04	3.01E-03
RNF4	0.26	1.20	3.10E-04	3.03E-03
TIFA	-0.55	0.68	3.10E-04	3.04E-03
SLC37A3	0.33	1.26	3.11E-04	3.04E-03
CLIP4	0.32	1.25	3.13E-04	3.06E-03
SNAPC4	0.38	1.30	3.14E-04	3.06E-03
ACKR3	-0.64	0.64	3.14E-04	3.06E-03
CTDNEP1	-0.32	0.80	3.15E-04	3.07E-03
ZNF367	-0.47	0.72	3.16E-04	3.08E-03
PWP1	0.26	1.20	3.16E-04	3.08E-03
CXXC5	-0.61	0.65	3.19E-04	3.10E-03
NCOR2	0.31	1.24	3.24E-04	3.15E-03
WDR33	0.26	1.20	3.24E-04	3.15E-03
DYNC1LI2	0.25	1.19	3.26E-04	3.16E-03
UBE2V2	0.33	1.26	3.27E-04	3.17E-03
VMP1	0.29	1.22	3.28E-04	3.18E-03
GATAD2A	0.29	1.22	3.28E-04	3.18E-03
HIPK3	0.28	1.21	3.30E-04	3.19E-03
BNC1	0.21	1.16	3.31E-04	3.20E-03
DHX32	0.30	1.23	3.32E-04	3.21E-03
UBAP2	0.26	1.20	3.34E-04	3.22E-03
ARHGEF28	0.33	1.26	3.37E-04	3.25E-03
CEBPB	-0.38	0.77	3.38E-04	3.26E-03
GNA13	0.32	1.25	3.41E-04	3.29E-03
C21orf91	-0.40	0.76	1.45E-05	1.48E-04
TMPO	0.36	1.28	1.46E-05	1.49E-04
IPO11	0.40	1.32	1.50E-05	1.53E-04
CTBP2	0.35	1.28	1.50E-05	1.53E-04
PDS5A	0.30	1.23	1.51E-05	1.54E-04
TGOLN2	0.29	1.23	1.51E-05	1.54E-04
PPP2R2B	-0.75	0.60	1.51E-05	1.54E-04
TNS3	-0.49	0.71	1.52E-05	1.55E-04
WDR4	0.40	1.32	1.54E-05	1.56E-04
THSD1	-0.36	0.78	1.56E-05	1.58E-04
LRP4	0.43	1.35	1.58E-05	1.60E-04
CHPT1	0.62	1.53	1.58E-05	1.60E-04
C1QTNF1	-0.97	0.51	1.58E-05	1.60E-04
IFNLR1	-0.44	0.73	1.59E-05	1.61E-04
BNIP2	-0.35	0.79	1.60E-05	1.62E-04
RHBDD1	-0.40	0.76	1.60E-05	1.62E-04
DPP4	0.89	1.86	1.61E-05	1.63E-04
PEX19	-0.40	0.76	1.61E-05	1.63E-04
ALDH4A1	-0.35	0.79	1.62E-05	1.63E-04
RPL29	-0.28	0.82	1.65E-05	1.66E-04
CYFIP2	-1.26	0.42	1.66E-05	1.68E-04
AKT1S1	-0.37	0.77	1.67E-05	1.68E-04
NEK11	-0.87	0.55	1.70E-05	1.70E-04
ETV3	-0.38	0.77	1.72E-05	1.73E-04
GCLC	-0.35	0.78	1.74E-05	1.75E-04
C11orf49	-0.44	0.74	1.75E-05	1.75E-04
PLXNA2	-0.33	0.79	1.78E-05	1.78E-04
XRCC2	0.60	1.51	1.78E-05	1.78E-04
TRIAP1	-0.41	0.75	1.79E-05	1.79E-04
ERGIC2	0.35	1.28	1.79E-05	1.79E-04
NUP35	0.55	1.47	1.80E-05	1.80E-04
PRKRIR	0.36	1.29	1.81E-05	1.81E-04
PTPRF	-0.28	0.82	1.83E-05	1.83E-04
CTDSP1	-0.46	0.73	1.84E-05	1.84E-04
BIN1	0.54	1.46	1.88E-05	1.88E-04
GLCE	0.45	1.37	1.89E-05	1.88E-04
STAG2	0.30	1.23	1.90E-05	1.89E-04
BSDC1	-0.33	0.80	1.91E-05	1.90E-04
NCBP1	0.33	1.26	1.91E-05	1.90E-04
CCSAP	0.47	1.39	1.92E-05	1.90E-04
RBL1	0.44	1.35	1.92E-05	1.90E-04
TP53I3	-0.43	0.74	1.93E-05	1.91E-04
DNHD1	-0.46	0.73	1.94E-05	1.92E-04
FAM195B	-0.47	0.72	1.94E-05	1.92E-04
PIGR	-4.43	0.05	1.95E-05	NA
PDLIM7	0.33	1.26	1.96E-05	1.94E-04
CCT6A	0.29	1.23	1.98E-05	1.96E-04
SIN3B	-0.31	0.80	2.01E-05	1.98E-04
FAM83H-AS1	-0.46	0.73	2.01E-05	1.99E-04
KIAA1524	0.46	1.38	2.02E-05	1.99E-04
ELAVL1	0.34	1.26	2.02E-05	2.00E-04
ARSA	-0.36	0.78	2.03E-05	2.00E-04
RIF1	0.38	1.30	2.03E-05	2.00E-04
PLRG1	0.32	1.25	2.06E-05	2.03E-04
FGFRL1	0.50	1.42	2.07E-05	2.03E-04
DNAJC13	0.32	1.25	2.08E-05	2.04E-04
TLN2	0.44	1.35	2.08E-05	2.04E-04
HMOX2	-0.33	0.80	2.08E-05	2.04E-04
KIAA0895L	-0.49	0.71	2.08E-05	2.04E-04
FBLN1	0.30	1.23	2.09E-05	2.04E-04
FKBP8	-0.30	0.81	2.11E-05	2.06E-04
METTL16	-0.38	0.77	2.11E-05	2.06E-04
PRIM1	0.57	1.49	2.15E-05	2.10E-04
GDF11	0.51	1.43	2.16E-05	2.10E-04
TSPAN1	-0.59	0.66	2.16E-05	2.10E-04
TMEM14A	0.47	1.39	2.18E-05	2.13E-04
LAMP3	-0.45	0.73	2.19E-05	2.13E-04
RNF145	0.30	1.23	2.20E-05	2.14E-04
KIAA1549L	-0.67	0.63	2.21E-05	2.15E-04
SLC13A5	0.61	1.53	2.23E-05	2.17E-04
FUS	0.28	1.21	2.23E-05	2.17E-04
TCF7	1.29	2.45	2.24E-05	2.17E-04
SEC31B	-0.69	0.62	2.25E-05	2.18E-04
IL33	-3.11	0.12	2.26E-05	NA
PSMC3	0.32	1.25	2.26E-05	2.19E-04
AASDHPPT	0.37	1.29	2.27E-05	2.20E-04
RAB3GAP1	-0.30	0.81	2.27E-05	2.20E-04
C6orf48	-0.35	0.79	2.28E-05	2.21E-04
ZNF513	-0.51	0.70	2.28E-05	2.21E-04
IGFBP4	-0.47	0.72	2.29E-05	2.21E-04
P3H2	-0.30	0.81	2.29E-05	2.21E-04
MX1	-0.35	0.78	2.30E-05	2.22E-04
RP11-54H7.4	-0.33	0.79	2.31E-05	2.22E-04
ARMT1	0.42	1.33	2.31E-05	2.22E-04
UBE2Q2	0.37	1.29	2.32E-05	2.23E-04
FAM63A	-0.43	0.74	2.35E-05	2.26E-04
FAM129A	0.31	1.24	2.35E-05	2.26E-04
TCEB3	0.33	1.26	2.36E-05	2.26E-04
TNFRSF14	-0.54	0.69	2.36E-05	2.27E-04
DHTKD1	0.35	1.27	2.38E-05	2.28E-04

**RP11-84G21.1**	-0.62	0.65	3.42E-04	3.29E-03	**GPX8**	0.38	1.30	2.39E-05	2.29E-04
**SLC7A4**	-0.67	0.63	3.43E-04	3.29E-03	**SCD5**	0.31	1.24	2.44E-05	2.34E-04
**BCL2L13**	0.26	1.20	3.43E-04	3.29E-03	**KCNC4**	0.58	1.49	2.46E-05	2.35E-04
**RANGAP1**	0.27	1.21	3.44E-04	3.31E-03	**USP37**	0.50	1.41	2.46E-05	2.36E-04
**MFSD3**	-0.44	0.74	3.44E-04	3.31E-03	**TEAD4**	0.56	1.48	2.47E-05	2.36E-04
**PTPN12**	0.29	1.22	3.52E-04	3.38E-03	**CREG2**	0.39	1.31	2.50E-05	2.39E-04
**LOXL2**	-0.54	0.69	3.53E-04	3.38E-03	**ABCA5**	-0.48	0.72	2.50E-05	2.39E-04
**LINC00641**	0.51	1.42	3.56E-04	3.41E-03	**NADK**	-0.35	0.79	2.54E-05	2.42E-04
**KRCC1**	0.38	1.30	3.59E-04	3.44E-03	**DSP**	-0.29	0.82	2.57E-05	2.44E-04
**SIRT1**	0.44	1.36	3.63E-04	3.47E-03	**ADIPOR1**	-0.28	0.82	2.58E-05	2.45E-04
**ASB7**	0.46	1.38	3.65E-04	3.49E-03	**TEP1**	-0.41	0.75	2.58E-05	2.46E-04
**KDM4B**	0.32	1.25	3.65E-04	3.49E-03	**CPOX**	0.36	1.28	2.58E-05	2.46E-04
**SLC41A1**	0.30	1.23	3.66E-04	3.49E-03	**DDX51**	0.38	1.31	2.60E-05	2.47E-04
**JMJD6**	0.38	1.30	3.66E-04	3.49E-03	**USP31**	-0.35	0.78	2.62E-05	2.49E-04
**TGFBR1**	0.33	1.25	3.72E-04	3.54E-03	**TMEM184A**	-0.44	0.74	2.63E-05	2.49E-04
**HELZ2**	-0.32	0.80	3.73E-04	3.55E-03	**AIMP2**	0.48	1.40	2.65E-05	2.51E-04
**NOL10**	0.31	1.24	3.74E-04	3.56E-03	**STIL**	0.45	1.36	2.70E-05	2.56E-04
**PEX19**	0.29	1.22	3.74E-04	3.56E-03	**FASN**	-0.33	0.79	2.71E-05	2.56E-04
**RNASEH2A**	-0.30	0.81	3.75E-04	3.56E-03	**NFKBIZ**	-0.44	0.74	2.71E-05	2.56E-04
**NIPBL**	0.28	1.21	3.77E-04	3.58E-03	**METTL8**	-0.40	0.76	2.71E-05	2.56E-04
**LUZP1**	0.24	1.18	3.80E-04	3.60E-03	**ZMIZ2**	-0.39	0.76	2.73E-05	2.58E-04
**AVL9**	0.30	1.23	3.80E-04	3.60E-03	**MEF2D**	-0.37	0.78	2.77E-05	2.61E-04
**KIF14**	0.35	1.27	3.80E-04	3.60E-03	**PIDD1**	-0.42	0.75	2.80E-05	2.64E-04
**SNHG10**	-0.64	0.64	3.82E-04	3.61E-03	**CNTNAP1**	0.69	1.61	2.81E-05	2.65E-04
**CCAR1**	0.25	1.19	3.82E-04	3.61E-03	**DNAJC7**	0.33	1.26	2.84E-05	2.67E-04
**THOC2**	0.31	1.24	3.84E-04	3.63E-03	**ASPH**	0.28	1.22	2.86E-05	2.69E-04
**NFKBIA**	0.29	1.23	3.85E-04	3.64E-03	**YPEL5**	-0.39	0.76	2.90E-05	2.73E-04
**TFG**	0.22	1.16	3.85E-04	3.64E-03	**VCL**	-0.31	0.80	2.91E-05	2.74E-04
**HELZ**	0.30	1.23	3.88E-04	3.67E-03	**LPGAT1**	0.34	1.26	2.92E-05	2.74E-04
**TMEM98**	-0.45	0.73	3.91E-04	3.68E-03	**MRPL3**	0.32	1.25	2.93E-05	2.75E-04
**BRD1**	0.31	1.24	3.91E-04	3.68E-03	**CENPN**	0.53	1.44	2.93E-05	2.75E-04
**ABHD14A**	-0.62	0.65	3.92E-04	3.69E-03	**GTPBP4**	0.31	1.24	2.94E-05	2.76E-04
**TGM2**	-0.66	0.63	3.94E-04	3.70E-03	**HDDC2**	0.37	1.29	2.95E-05	2.77E-04
**GOLGA5**	0.29	1.22	3.95E-04	3.71E-03	**FUBP1**	0.29	1.23	2.97E-05	2.78E-04
**DUSP1**	0.34	1.27	3.95E-04	3.71E-03	**RHOBTB1**	-0.89	0.54	2.98E-05	2.78E-04
**ADAM9**	0.24	1.18	3.97E-04	3.73E-03	**KLF6**	-0.36	0.78	2.99E-05	2.80E-04
**RTTN**	0.30	1.23	3.98E-04	3.73E-03	**TRIM35**	-0.36	0.78	3.01E-05	2.81E-04
**REXO1**	0.30	1.23	3.99E-04	3.74E-03	**DGKD**	0.50	1.42	3.03E-05	2.83E-04
**TMEM131**	0.25	1.19	3.99E-04	3.74E-03	**NUP188**	0.30	1.23	3.03E-05	2.83E-04
**FAF2**	0.25	1.19	3.99E-04	3.74E-03	**RBM15B**	-0.35	0.78	3.05E-05	2.84E-04
**GSPT1**	0.23	1.17	4.01E-04	3.75E-03	**NUMBL**	0.46	1.38	3.07E-05	2.86E-04
**KLHDC4**	0.35	1.27	4.02E-04	3.76E-03	**PYCRL**	-0.58	0.67	3.07E-05	2.86E-04
**C4orf46**	-0.43	0.74	4.02E-04	3.76E-03	**PSD4**	-0.39	0.77	3.08E-05	2.87E-04
**TM7SF2**	-0.40	0.76	4.06E-04	3.79E-03	**TM2D2**	-0.42	0.75	3.11E-05	2.89E-04
**FBXL16**	-0.65	0.64	4.07E-04	3.80E-03	**C15orf59**	-0.83	0.56	3.12E-05	2.90E-04
**CREB5**	0.54	1.45	4.09E-04	3.82E-03	**FOXRED2**	0.51	1.42	3.12E-05	2.90E-04
**SLFN13**	-0.60	0.66	4.10E-04	3.83E-03	**ELOVL5**	0.30	1.23	3.14E-05	2.92E-04
**DENND5B**	0.46	1.38	4.11E-04	3.83E-03	**PDGFA**	0.46	1.38	3.15E-05	2.92E-04
**PPIF**	0.26	1.19	4.11E-04	3.83E-03	**RFC1**	0.32	1.25	3.17E-05	2.94E-04
**CDK12**	0.24	1.18	4.12E-04	3.83E-03	**RECQL4**	0.43	1.34	3.19E-05	2.95E-04
**HSP90AB1**	0.19	1.14	4.12E-04	3.83E-03	**FSTL1**	-0.37	0.78	3.21E-05	2.97E-04
**HTT**	0.28	1.22	4.14E-04	3.85E-03	**LIN9**	0.59	1.50	3.23E-05	2.99E-04
**CENPM**	-0.52	0.70	4.17E-04	3.87E-03	**POFUT1**	0.31	1.24	3.28E-05	3.03E-04
**USP9X**	0.23	1.17	4.17E-04	3.87E-03	**CCT8**	0.28	1.21	3.28E-05	3.03E-04
**FIZ1**	-0.48	0.72	4.18E-04	3.87E-03	**NUP43**	0.37	1.30	3.29E-05	3.03E-04
**DBNL**	-0.26	0.84	4.19E-04	3.88E-03	**MDGA1**	0.94	1.92	3.30E-05	3.04E-04
**FAM208A**	0.31	1.24	4.21E-04	3.89E-03	**TMEM45A**	-0.38	0.77	3.31E-05	3.05E-04
**STARD10**	-0.32	0.80	4.23E-04	3.91E-03	**CREB3L2**	-0.33	0.80	3.32E-05	3.05E-04
**EXOC6B**	0.27	1.20	4.24E-04	3.92E-03	**MT-RNR2**	-0.27	0.83	3.36E-05	3.09E-04
**GCSAM**	-0.64	0.64	4.25E-04	3.93E-03	**URGCP**	-0.35	0.78	3.36E-05	3.09E-04
**COQ10B**	0.43	1.35	4.27E-04	3.94E-03	**MAP2K1**	0.34	1.26	3.37E-05	3.10E-04
**RASGEF1A**	-0.56	0.68	4.28E-04	3.95E-03	**RECK**	0.63	1.55	3.40E-05	3.12E-04
**SKI**	-0.30	0.81	4.29E-04	3.95E-03	**TRAF5**	0.76	1.69	3.43E-05	3.15E-04
**SUPT6H**	0.24	1.18	4.29E-04	3.95E-03	**MAP3K4**	0.34	1.27	3.44E-05	3.15E-04
**SLC27A4**	-0.31	0.81	4.30E-04	3.96E-03	**RP1-272L16.1**	-0.73	0.60	3.44E-05	3.15E-04
**CDC25B**	-0.26	0.83	4.31E-04	3.96E-03	**MAPK11**	-0.74	0.60	3.44E-05	3.15E-04
**FGFR1OP2**	0.39	1.31	4.33E-04	3.98E-03	**PCK2**	-0.42	0.75	3.47E-05	3.18E-04
**DDI2**	0.30	1.23	4.35E-04	3.99E-03	**CXXC5**	0.58	1.50	3.48E-05	3.18E-04
**RAP1B**	0.31	1.24	4.35E-04	4.00E-03	**CALU**	0.30	1.23	3.48E-05	3.18E-04
**RAF1**	0.25	1.19	4.36E-04	4.00E-03	**IGFBP2**	-0.28	0.82	3.50E-05	3.19E-04
**PDZK1IP1**	-0.60	0.66	4.39E-04	4.03E-03	**ARHGAP18**	0.45	1.37	3.52E-05	3.21E-04
**ZMIZ1**	-0.28	0.83	4.41E-04	4.04E-03	**ZHX1**	-0.33	0.80	3.55E-05	3.24E-04
**WARS**	-0.22	0.86	4.42E-04	4.05E-03	**SQLE**	-0.30	0.81	3.55E-05	3.24E-04
**ZFP36L1**	-0.23	0.86	4.44E-04	4.06E-03	**TBC1D5**	-0.32	0.80	3.58E-05	3.26E-04
**SLFN5**	0.26	1.20	4.44E-04	4.06E-03	**RPA2**	0.37	1.29	3.59E-05	3.27E-04
**WWP1**	0.29	1.22	4.44E-04	4.06E-03	**RAD54L**	0.50	1.41	3.60E-05	3.27E-04
**TPRXL**	-0.53	0.69	4.45E-04	4.06E-03	**RAB11FIP2**	0.51	1.43	3.60E-05	3.27E-04
**SERPINB1**	-0.28	0.82	4.46E-04	4.07E-03	**MB21D1**	0.55	1.47	3.67E-05	3.33E-04
**CAV1**	0.21	1.16	4.46E-04	4.07E-03	**FDXR**	-0.34	0.79	3.68E-05	3.34E-04
**LINC00900**	-0.66	0.63	4.48E-04	4.08E-03	**DDX39A**	0.35	1.27	3.70E-05	3.35E-04
**VKORC1L1**	0.29	1.22	4.50E-04	4.10E-03	**SP2**	-0.52	0.70	3.76E-05	3.41E-04
**ZDHHC17**	0.50	1.41	4.50E-04	4.10E-03	**USP14**	0.29	1.22	3.76E-05	3.41E-04
**CYB561**	-0.29	0.82	4.52E-04	4.11E-03	**DDAH1**	0.89	1.86	3.78E-05	3.42E-04
**AXL**	0.32	1.25	4.52E-04	4.11E-03	**GFPT1**	0.33	1.26	3.79E-05	3.43E-04
**USP47**	0.27	1.21	4.56E-04	4.14E-03	**ADM**	-0.35	0.78	3.88E-05	3.51E-04
**MKL1**	0.34	1.27	4.57E-04	4.14E-03	**RPAP3**	0.41	1.33	3.89E-05	3.52E-04
**CA9**	-0.59	0.66	4.58E-04	4.16E-03	**SREBF1**	0.27	1.21	3.93E-05	3.55E-04
**SASH1**	0.32	1.25	4.59E-04	4.16E-03	**KLC4**	-0.56	0.68	3.94E-05	3.55E-04
**MCFD2**	0.25	1.19	4.68E-04	4.24E-03	**KCNK5**	1.10	2.14	3.96E-05	3.57E-04
**RGS4**	-0.65	0.64	4.71E-04	4.26E-03	**SMC3**	0.31	1.24	3.98E-05	3.59E-04

CIART	-0.59	0.67	4.71E-04	4.26E-03
RBMX	0.21	1.16	4.76E-04	4.30E-03
SPRED1	0.38	1.30	4.77E-04	4.31E-03
SGPP1	0.40	1.32	4.80E-04	4.33E-03
CBR1	-0.28	0.83	4.80E-04	4.33E-03
SNX21	-0.51	0.70	4.81E-04	4.33E-03
BCL7B	0.31	1.24	4.81E-04	4.33E-03
CACUL1	0.28	1.21	4.83E-04	4.35E-03
CDK17	0.36	1.28	4.85E-04	4.37E-03
ANKRD40	0.30	1.23	4.88E-04	4.39E-03
ZSWIM6	0.39	1.31	4.89E-04	4.40E-03
BRD8	0.29	1.22	4.91E-04	4.41E-03
ATP5G1	-0.27	0.83	4.95E-04	4.45E-03
TXN2	-0.26	0.83	4.98E-04	4.46E-03
LMO4	-0.34	0.79	5.00E-04	4.48E-03
COMT	-0.29	0.82	5.01E-04	4.48E-03
DNAJA2	0.25	1.19	5.01E-04	4.48E-03
PYGO2	-0.34	0.79	5.02E-04	4.49E-03
AC093838.4	0.46	1.38	5.05E-04	4.52E-03
SEMA4A	-0.34	0.79	5.09E-04	4.55E-03
LRP5	0.27	1.21	5.11E-04	4.56E-03
GAK	0.27	1.20	5.11E-04	4.56E-03
INCENP	-0.26	0.84	5.16E-04	4.60E-03
DOCK9	0.24	1.18	5.17E-04	4.61E-03
SDHA	0.24	1.18	5.18E-04	4.61E-03
DPYSL3	0.63	1.55	5.18E-04	4.61E-03
TYRO3	-0.35	0.79	5.21E-04	4.63E-03
GPR1	-0.61	0.66	5.22E-04	4.63E-03
TATDN2P2	0.53	1.45	5.22E-04	4.63E-03
RP11-336A10.4	-0.61	0.66	5.22E-04	4.63E-03
AMFR	0.28	1.22	5.22E-04	4.63E-03
PAQR8	-0.62	0.65	5.26E-04	4.67E-03
KIAA0040	0.24	1.18	5.29E-04	4.69E-03
MPV17L	-0.65	0.64	5.30E-04	4.69E-03
LDLR	0.23	1.17	5.31E-04	4.70E-03
CHST15	0.29	1.22	5.32E-04	4.70E-03
ITSN1	0.27	1.20	5.32E-04	4.70E-03
RNF43	0.34	1.26	5.33E-04	4.71E-03
NAA15	0.27	1.20	5.34E-04	4.71E-03
WDR82	-0.22	0.86	5.35E-04	4.72E-03
NUP35	0.37	1.29	5.37E-04	4.73E-03
GCN1	0.22	1.17	5.41E-04	4.76E-03
OSBP2	0.25	1.19	5.42E-04	4.77E-03
BLNK	-0.62	0.65	5.43E-04	4.77E-03
CENPC	-0.44	0.74	5.45E-04	4.79E-03
SSH1	0.24	1.18	5.46E-04	4.79E-03
SPIN1	0.27	1.21	5.47E-04	4.80E-03
ZNF480	0.39	1.31	5.50E-04	4.83E-03
PPP1R18	0.25	1.19	5.51E-04	4.83E-03
WDR45B	0.23	1.17	5.53E-04	4.84E-03
CCDC64B	-0.35	0.78	5.54E-04	4.85E-03
TRAF3IP2	0.26	1.19	5.55E-04	4.86E-03
GLTSCR1L	-0.43	0.74	5.56E-04	4.87E-03
NABP1	0.41	1.32	5.59E-04	4.88E-03
DCAF6	0.27	1.21	5.59E-04	4.88E-03
CWC22	0.32	1.25	5.60E-04	4.89E-03
ZC3H3	0.33	1.26	5.63E-04	4.91E-03
CDH4	0.32	1.25	5.64E-04	4.92E-03
SAC3D1	-0.50	0.71	5.66E-04	4.93E-03
SREK1IP1	0.35	1.28	5.66E-04	4.93E-03
URGCP	0.33	1.26	5.67E-04	4.93E-03
SEMA3F	-0.24	0.84	5.69E-04	4.95E-03
INSR	-0.55	0.68	5.73E-04	4.97E-03
AKR1C2	-0.56	0.68	5.73E-04	4.98E-03
PUM3	0.25	1.19	5.74E-04	4.98E-03
IL4R	0.26	1.20	5.76E-04	4.99E-03
DLG5	0.25	1.19	5.77E-04	5.00E-03
TSKU	-0.30	0.81	5.77E-04	5.00E-03
MMP1	-0.57	0.67	5.78E-04	5.00E-03
SLC2A6	-0.53	0.69	5.81E-04	5.03E-03
BCAM	0.30	1.23	5.83E-04	5.04E-03
KRT16P2	-0.64	0.64	5.84E-04	5.04E-03
KLHDC8B	-0.38	0.77	5.84E-04	5.04E-03
ARF6	-0.22	0.86	5.86E-04	5.05E-03
A4GALT	-0.41	0.75	5.88E-04	5.07E-03
RBBP9	-0.35	0.78	5.91E-04	5.09E-03
RP5-1187M17.10	0.53	1.44	5.94E-04	5.11E-03
ZNF148	0.33	1.26	5.94E-04	5.11E-03
CEP152	0.45	1.37	5.99E-04	5.15E-03
ASPH	0.22	1.17	5.99E-04	5.15E-03
PFDN4	0.47	1.38	6.01E-04	5.17E-03
ARHGAP12	0.35	1.27	6.06E-04	5.20E-03
USP12	0.36	1.28	6.12E-04	5.25E-03
MAPKAPK5	0.31	1.24	6.13E-04	5.25E-03
THYN1	-0.34	0.79	6.17E-04	5.29E-03
BASP1	0.27	1.21	6.17E-04	5.29E-03
FAM118A	0.36	1.28	6.20E-04	5.31E-03
FAM86B1	-0.63	0.65	6.21E-04	5.31E-03
NUP93	0.23	1.18	6.23E-04	5.33E-03
SOCS1	-0.64	0.64	6.24E-04	5.33E-03

ZNF812	-1.17	0.44	4.04E-05	3.64E-04
CTDSPL2	0.39	1.31	4.06E-05	3.66E-04
IDUA	-0.71	0.61	4.10E-05	3.68E-04
CENPM	0.56	1.48	4.14E-05	3.72E-04
MVK	-0.34	0.79	4.15E-05	3.73E-04
SLC48A1	-0.42	0.75	4.17E-05	3.75E-04
NOP58	0.33	1.26	4.18E-05	3.75E-04
AEN	-0.42	0.75	4.18E-05	3.75E-04
MEF2A	0.37	1.29	4.20E-05	3.76E-04
MBD3	-0.41	0.75	4.24E-05	3.80E-04
MBTPS1	-0.30	0.81	4.27E-05	3.82E-04
NF1	0.30	1.23	4.28E-05	3.82E-04
STAMBP	-0.33	0.80	4.28E-05	3.82E-04
COQ4	-0.38	0.77	4.28E-05	3.82E-04
CNNM4	0.36	1.28	4.29E-05	3.83E-04
RFTN1P1	-1.40	0.38	4.34E-05	3.87E-04
ERG	1.62	3.08	4.36E-05	3.89E-04
EPS8L1	-0.39	0.76	4.38E-05	3.90E-04
AAK1	-0.33	0.80	4.39E-05	3.90E-04
CCDC18	0.50	1.41	4.46E-05	3.96E-04
SEL1L	0.30	1.23	4.47E-05	3.97E-04
SSB	0.31	1.24	4.50E-05	3.99E-04
TIMM17A	0.32	1.25	4.50E-05	3.99E-04
LDHA	0.27	1.21	4.52E-05	4.01E-04
ADRB2	-0.38	0.77	4.57E-05	4.05E-04
NEURL1B	0.36	1.28	4.59E-05	4.07E-04
RP11-291L22.9	-0.91	0.53	4.63E-05	4.10E-04
TAF5	0.55	1.47	4.64E-05	4.10E-04
RFX7	-0.32	0.80	4.65E-05	4.11E-04
PTPN11	0.29	1.23	4.68E-05	4.13E-04
IL6ST	0.32	1.25	4.68E-05	4.13E-04
SYT12	0.72	1.65	4.73E-05	4.17E-04
COPS8	0.32	1.25	4.74E-05	4.18E-04
TWF1	0.28	1.21	4.74E-05	4.18E-04
CLU	-0.50	0.71	4.91E-05	4.33E-04
TEX261	-0.37	0.78	4.96E-05	4.37E-04
PSMC2	0.31	1.24	4.98E-05	4.37E-04
SLC30A9	0.33	1.26	4.98E-05	4.37E-04
CLDN12	-0.37	0.77	4.98E-05	4.37E-04
FOXO4	-0.57	0.67	5.00E-05	4.39E-04
ADCY6	-0.32	0.80	5.01E-05	4.39E-04
CYP2J2	-0.77	0.58	5.13E-05	4.49E-04
MIRLET7BHG	-0.58	0.67	5.18E-05	4.54E-04
CAMKK1	0.55	1.46	5.18E-05	4.54E-04
ARHGAP17	-0.42	0.75	5.24E-05	4.58E-04
FAM127A	-0.33	0.80	5.25E-05	4.59E-04
TMEM189	0.35	1.27	5.29E-05	4.62E-04
TOR1A	-0.39	0.76	5.31E-05	4.63E-04
GPX3	-0.32	0.80	5.38E-05	4.69E-04
BRI3BP	0.35	1.27	5.38E-05	4.69E-04
NUDT21	0.32	1.25	5.40E-05	4.70E-04
KLHL28	0.45	1.37	5.40E-05	4.70E-04
LCAT	-0.61	0.65	5.43E-05	4.72E-04
DPY19L1	0.43	1.35	5.44E-05	4.73E-04
COL18A1	0.38	1.30	5.44E-05	4.73E-04
TRPM4	-0.35	0.78	5.45E-05	4.73E-04
ADSS	0.35	1.27	5.45E-05	4.73E-04
CITED2	-0.46	0.73	5.50E-05	4.77E-04
MMRN2	0.60	1.52	5.53E-05	4.79E-04
SLC16A2	-0.29	0.82	5.60E-05	4.85E-04
ZNF678	0.68	1.61	5.62E-05	4.86E-04
NCAPD3	0.33	1.26	5.65E-05	4.89E-04
BUB3	0.30	1.23	5.66E-05	4.90E-04
WIPF2	-0.36	0.78	5.67E-05	4.90E-04
CD164	0.27	1.21	5.71E-05	4.93E-04
HTATSF1	0.32	1.25	5.71E-05	4.93E-04
TBC1D16	-0.39	0.77	5.74E-05	4.95E-04
DDIT3	0.74	1.67	5.79E-05	4.99E-04
DDX18	0.30	1.23	5.85E-05	5.04E-04
NEIL3	0.57	1.49	5.93E-05	5.11E-04
GTSE1	0.44	1.35	5.95E-05	5.12E-04
GORASP1	-0.34	0.79	5.97E-05	5.13E-04
CWH43	-0.35	0.79	6.05E-05	5.19E-04
ORC6	0.43	1.35	6.05E-05	5.19E-04
TSC22D3	0.39	1.31	6.07E-05	5.21E-04
M6PR	0.31	1.24	6.09E-05	5.22E-04
ARHGAP1	-0.28	0.82	6.18E-05	5.29E-04
TUBD1	0.59	1.50	6.25E-05	5.36E-04
FRS2	0.36	1.28	6.26E-05	5.36E-04
GBP6	-0.33	0.79	6.28E-05	5.37E-04
YARS	0.28	1.22	6.30E-05	5.39E-04
CPSF3	0.32	1.25	6.37E-05	5.44E-04
AGFG1	0.29	1.23	6.44E-05	5.50E-04
SYK	-0.46	0.73	6.44E-05	5.50E-04
ABLIM3	-0.56	0.68	6.49E-05	5.53E-04
LMNA	0.28	1.21	6.49E-05	5.53E-04
MCL1	-0.26	0.84	6.51E-05	5.55E-04
HSPA12A	0.61	1.52	6.54E-05	5.56E-04
IRAK3	0.99	1.98	6.54E-05	5.57E-04
ABHD17C	0.39	1.31	6.56E-05	5.58E-04

HCFC1	0.26	1.20	6.27E-04	5.35E-03	COL9A2	-0.49	0.71	6.63E-05	5.63E-04
FZD8	-0.56	0.68	6.27E-04	5.35E-03	DDX60L	-0.32	0.80	6.64E-05	5.63E-04
SELT	0.25	1.19	6.30E-04	5.37E-03	WDHD1	0.38	1.30	6.66E-05	5.65E-04
POF1B	-0.30	0.81	6.30E-04	5.37E-03	CCNI	-0.30	0.81	6.76E-05	5.73E-04
ATXN2L	0.29	1.22	6.38E-04	5.43E-03	HAT1	0.34	1.26	6.81E-05	5.77E-04
ZNF468	0.43	1.35	6.38E-04	5.43E-03	LDHB	0.29	1.22	6.86E-05	5.81E-04
MACC1	0.28	1.22	6.49E-04	5.52E-03	FAT4	-1.20	0.44	6.90E-05	5.84E-04
SSFA2	0.24	1.18	6.50E-04	5.52E-03	NET1	0.31	1.24	6.93E-05	5.86E-04
ANKRD13D	0.32	1.25	6.54E-04	5.55E-03	GABRP	-0.86	0.55	7.00E-05	5.92E-04
RNASEH1	0.33	1.26	6.59E-04	5.59E-03	USP39	-0.35	0.78	7.03E-05	5.93E-04
BCL7C	-0.34	0.79	6.60E-04	5.60E-03	COLGALT1	0.29	1.23	7.06E-05	5.96E-04
VWA1	-0.36	0.78	6.61E-04	5.61E-03	MELK	0.37	1.29	7.07E-05	5.96E-04
PEG10	-0.63	0.64	6.62E-04	5.61E-03	EGR2	-1.06	0.48	7.07E-05	5.96E-04
BAHCC1	-0.42	0.75	6.66E-04	5.64E-03	PPP2R5B	-0.41	0.75	7.08E-05	5.96E-04
CDKN1A	-0.22	0.86	6.67E-04	5.64E-03	C11orf95	0.61	1.52	7.19E-05	6.06E-04
ZFY	0.46	1.38	6.67E-04	5.64E-03	THNSL2	-0.46	0.73	7.22E-05	6.07E-04
KIAA1549	0.41	1.33	6.68E-04	5.64E-03	KIF18A	0.52	1.44	7.28E-05	6.12E-04
TGFB3	-0.63	0.65	6.70E-04	5.66E-03	SECISBP2L	0.39	1.31	7.32E-05	6.15E-04
CELSR3	-0.63	0.65	6.72E-04	5.67E-03	GPNMB	0.31	1.24	7.35E-05	6.17E-04
CERS4	-0.43	0.74	6.75E-04	5.69E-03	TRIM26	-0.31	0.81	7.42E-05	6.23E-04
ZNF830	-0.46	0.73	6.76E-04	5.69E-03	CTSC	-0.27	0.83	7.47E-05	6.26E-04
GRB7	0.34	1.26	6.82E-04	5.74E-03	DAZAP1	0.29	1.22	7.48E-05	6.27E-04
TRPV3	0.54	1.45	6.83E-04	5.75E-03	KRT9	0.56	1.47	7.50E-05	6.29E-04
ANP32A	-0.24	0.85	6.87E-04	5.78E-03	HIF1AN	0.30	1.23	7.53E-05	6.31E-04
NEK7	0.32	1.25	6.87E-04	5.78E-03	RGMA	-0.64	0.64	7.56E-05	6.32E-04
NCKAP5	0.40	1.32	6.88E-04	5.78E-03	ERLIN2	0.30	1.23	7.66E-05	6.41E-04
RFWD3	0.26	1.20	6.99E-04	5.87E-03	CMTM7	-0.79	0.58	7.74E-05	6.47E-04
SSU72	0.24	1.18	7.00E-04	5.87E-03	GSG2	0.58	1.49	7.79E-05	6.51E-04
ITGB4	0.24	1.18	7.00E-04	5.87E-03	TMC4	-0.52	0.70	7.80E-05	6.51E-04
TNFRSF14	-0.57	0.67	7.00E-04	5.87E-03	MRTO4	0.32	1.25	7.81E-05	6.52E-04
LAMC1	0.20	1.15	7.04E-04	5.89E-03	SCARA3	0.29	1.22	7.90E-05	6.58E-04
RICTOR	0.35	1.27	7.08E-04	5.92E-03	PSG5	-0.80	0.57	7.93E-05	6.61E-04
MFSD6	0.24	1.18	7.11E-04	5.95E-03	CCNY	0.31	1.24	7.99E-05	6.66E-04
TM2D2	0.34	1.27	7.14E-04	5.97E-03	ARL15	-0.45	0.73	8.04E-05	6.69E-04
BAIAP2L2	-0.48	0.71	7.15E-04	5.97E-03	ACSS1	0.56	1.47	8.07E-05	6.71E-04
SOCS4	0.35	1.27	7.15E-04	5.97E-03	ARHGEF39	0.67	1.59	8.11E-05	6.74E-04
RNF217	0.33	1.26	7.19E-04	6.00E-03	STARD3NL	0.43	1.35	8.11E-05	6.74E-04
LIG1	-0.27	0.83	7.21E-04	6.01E-03	PCMT1	0.34	1.26	8.19E-05	6.80E-04
FGD3	-0.63	0.65	7.22E-04	6.02E-03	SUGT1	0.36	1.29	8.33E-05	6.91E-04
PRRC2A	0.24	1.18	7.25E-04	6.04E-03	MRPL17	-0.38	0.77	8.49E-05	7.04E-04
C10orf99	-0.63	0.65	7.27E-04	6.05E-03	NRBP1	0.28	1.21	8.52E-05	7.06E-04
PCBP4	-0.31	0.81	7.29E-04	6.07E-03	NEMP2	0.51	1.42	8.56E-05	7.09E-04
LINC01311	-0.52	0.70	7.32E-04	NA	SCRN1	0.27	1.21	8.62E-05	7.13E-04
MAP7D1	0.24	1.18	7.35E-04	6.11E-03	HOXA7	-1.03	0.49	8.69E-05	7.19E-04
TM7SF3	-0.22	0.86	7.36E-04	6.12E-03	SYNPO	0.30	1.23	8.73E-05	7.22E-04
POLR2G	-0.30	0.81	7.43E-04	6.17E-03	C12orf75	0.33	1.26	8.75E-05	7.22E-04
CFD	-0.63	0.65	7.52E-04	6.24E-03	MAML2	-0.38	0.77	8.75E-05	7.22E-04
PTP4A2	0.25	1.19	7.55E-04	6.26E-03	TEX264	-0.36	0.78	8.75E-05	7.22E-04
SLC25A32	0.32	1.25	7.55E-04	6.26E-03	AMOT	-0.38	0.77	8.88E-05	7.32E-04
FRYL	0.26	1.20	7.56E-04	6.26E-03	KANK2	0.42	1.34	8.94E-05	7.36E-04
CHD4	0.20	1.15	7.62E-04	6.31E-03	PIK3R1	0.32	1.25	8.94E-05	7.36E-04
WFDC21P	-0.59	0.66	7.63E-04	6.32E-03	MT-RNR1	-0.25	0.84	8.97E-05	7.38E-04
CAND2	-0.56	0.68	7.65E-04	6.32E-03	TMCC3	-0.34	0.79	8.99E-05	7.40E-04
CYB5R4	0.37	1.29	7.66E-04	6.33E-03	LDAH	-0.39	0.77	9.00E-05	7.40E-04
MAFB	-0.51	0.70	7.67E-04	6.34E-03	U47924.27	-1.37	0.39	9.01E-05	7.40E-04
IFIT5	-0.45	0.73	7.68E-04	6.34E-03	DHRS11	-0.47	0.72	9.02E-05	7.40E-04
ZNF451	0.34	1.26	7.71E-04	6.35E-03	PPP2CA	0.28	1.21	9.05E-05	7.42E-04
DSC1	-0.60	0.66	7.71E-04	6.35E-03	HDHD2	0.50	1.42	9.21E-05	7.55E-04
CASP2	-0.29	0.82	7.72E-04	6.35E-03	DLX1	0.56	1.47	9.25E-05	7.58E-04
EHMT1	0.26	1.20	7.72E-04	6.35E-03	LINC00265	-1.05	0.48	9.26E-05	7.58E-04
BIRC2	0.31	1.24	7.72E-04	6.35E-03	DMKN	-0.35	0.78	9.29E-05	7.60E-04
G2E3	0.38	1.30	7.74E-04	6.37E-03	IFI44	-0.38	0.77	9.48E-05	7.75E-04
PDS5A	0.22	1.17	7.85E-04	6.46E-03	TPR	0.28	1.21	9.55E-05	7.81E-04
KDM7A	0.43	1.35	7.89E-04	6.48E-03	NELFB	-0.33	0.80	9.58E-05	7.83E-04
NUDT16	-0.33	0.80	7.93E-04	6.51E-03	RSL1D1	0.28	1.22	9.64E-05	7.87E-04
PTPN11	0.23	1.18	7.95E-04	6.52E-03	STK35	-0.33	0.80	9.68E-05	7.90E-04
FSTL1	0.23	1.17	7.99E-04	6.55E-03	USP1	0.36	1.28	9.74E-05	7.95E-04
RP5-965G21.4	0.56	1.48	8.02E-04	6.58E-03	SFRP1	0.30	1.23	9.75E-05	7.95E-04
SUDS3	0.27	1.21	8.10E-04	6.63E-03	DGKQ	-0.36	0.78	9.80E-05	7.98E-04
CCDC86	0.33	1.26	8.16E-04	6.68E-03	CDC27	0.31	1.24	9.89E-05	8.05E-04
STOML1	-0.48	0.72	8.20E-04	6.71E-03	CD248	1.44	2.72	9.89E-05	8.05E-04
ABCC1	0.25	1.19	8.22E-04	6.72E-03	PMAIP1	0.38	1.30	9.91E-05	8.06E-04
RAB35	0.25	1.19	8.25E-04	6.74E-03	UHRF1	0.42	1.34	9.95E-05	8.08E-04
COL9A3	-0.56	0.68	8.27E-04	6.75E-03	ACP5	0.45	1.37	9.97E-05	8.10E-04
CTD-3014M21.1	0.50	1.41	8.27E-04	NA	PSMD14	0.29	1.23	9.99E-05	8.11E-04
S1PR2	-0.60	0.66	8.30E-04	6.77E-03	TCTA	-0.46	0.73	1.01E-04	8.22E-04
INTS5	-0.35	0.79	8.33E-04	6.80E-03	SERBP1	0.26	1.20	1.01E-04	8.23E-04
CNTNAP2	-0.50	0.71	8.40E-04	6.85E-03	TUBA4A	0.27	1.20	1.02E-04	8.24E-04
NFKBIE	0.45	1.37	8.42E-04	6.86E-03	LIN52	-0.59	0.66	1.02E-04	8.26E-04
NDUFC1	-0.33	0.80	8.42E-04	6.86E-03	PSMB2	0.27	1.21	1.02E-04	8.29E-04
ZNF318	0.25	1.19	8.43E-04	6.86E-03	ZFAS1	-0.30	0.81	1.04E-04	8.37E-04
NFE2L1	-0.20	0.87	8.47E-04	6.89E-03	SDF2L1	0.43	1.35	1.04E-04	8.37E-04
PDCD1LG2	0.57	1.48	8.50E-04	6.91E-03	INF2	0.32	1.24	1.04E-04	8.44E-04
KIF26B	-0.56	0.68	8.52E-04	6.92E-03	SKA1	0.47	1.38	1.05E-04	8.46E-04
SNX14	0.32	1.25	8.54E-04	6.93E-03	PDIK1L	0.53	1.44	1.06E-04	8.52E-04
FUT3	-0.28	0.82	8.58E-04	6.96E-03	FMNL3	0.61	1.53	1.06E-04	8.53E-04
ADK	0.26	1.20	8.58E-04	6.96E-03	CDH16	-0.85	0.55	1.06E-04	8.57E-04
C1D	0.48	1.39	8.59E-04	6.96E-03	PNN	0.31	1.24	1.07E-04	8.64E-04
CCDC88A	0.31	1.24	8.65E-04	7.00E-03	DEGS1	-0.30	0.81	1.08E-04	8.67E-04
PAX9	-0.61	0.65	8.65E-04	7.00E-03	POLR2D	0.37	1.30	1.08E-04	8.67E-04

RBPJ	0.30	1.23	8.68E-04	7.02E-03
SDCBP2	-0.30	0.81	8.70E-04	7.03E-03
PRDX5	-0.22	0.86	8.74E-04	7.06E-03
TCF7L1	0.33	1.26	8.74E-04	7.06E-03
NXN	0.23	1.18	8.87E-04	7.16E-03
CTU1	-0.56	0.68	8.88E-04	7.16E-03
GPAT4	0.29	1.23	8.90E-04	7.17E-03
STAT5B	0.31	1.24	8.93E-04	7.19E-03
PLLP	-0.47	0.72	8.93E-04	7.19E-03
NACC1	0.27	1.20	8.94E-04	7.19E-03
ACSL4	0.26	1.20	8.98E-04	7.22E-03
COX17	0.37	1.29	9.01E-04	7.24E-03
POLR1B	-0.27	0.83	9.06E-04	7.28E-03
APIP	0.50	1.41	9.08E-04	7.29E-03
PIKFYVE	0.30	1.23	9.12E-04	7.32E-03
PSIP1	-0.35	0.78	9.14E-04	7.33E-03
ABHD5	-0.36	0.78	9.17E-04	7.34E-03
TNNT1	-0.32	0.80	9.21E-04	7.37E-03
ZNF574	-0.37	0.77	9.29E-04	7.43E-03
CHST11	0.31	1.24	9.33E-04	7.46E-03
LZTS1	-0.56	0.68	9.34E-04	7.46E-03
PUDP	0.32	1.25	9.34E-04	7.46E-03
ANKRD28	0.29	1.22	9.34E-04	7.46E-03
NAGS	-0.53	0.69	9.37E-04	7.48E-03
AIMP1	0.33	1.26	9.42E-04	7.51E-03
FRMD4B	0.25	1.19	9.43E-04	7.51E-03
SWAP70	0.31	1.24	9.43E-04	7.51E-03
SLC38A5	-0.28	0.82	9.43E-04	7.51E-03
SHMT2	-0.23	0.85	9.48E-04	7.54E-03
HAUS6	0.33	1.26	9.48E-04	7.54E-03
UBE2S	0.37	1.29	9.49E-04	7.54E-03
SUN2	0.26	1.20	9.51E-04	7.56E-03
TRAK1	0.24	1.18	9.53E-04	7.56E-03
PDCD10	0.30	1.23	9.54E-04	7.57E-03
SCARB1	-0.27	0.83	9.57E-04	7.58E-03
CDC27	0.25	1.19	9.58E-04	7.58E-03
FUCA2	-0.33	0.79	9.58E-04	7.58E-03
PISD	0.30	1.23	9.58E-04	7.58E-03
SNHG1	-0.30	0.81	9.58E-04	7.58E-03
IFNGR2	0.24	1.18	9.59E-04	7.58E-03
NUDT8	-0.59	0.66	9.61E-04	7.59E-03
BTG2	-0.28	0.82	9.63E-04	7.60E-03
PPP2R2D	0.33	1.26	9.64E-04	7.60E-03
TAOK3	0.26	1.20	9.65E-04	7.61E-03
TCEA3	-0.62	0.65	9.65E-04	7.61E-03
AP1G1	0.22	1.16	9.67E-04	7.61E-03
PNPLA1	-0.50	0.71	9.69E-04	NA
GPNMB	-0.41	0.75	9.70E-04	7.63E-03
ASCC2	0.24	1.18	9.71E-04	7.64E-03
CRCT1	-0.30	0.81	9.79E-04	7.70E-03
TPPP	-0.38	0.77	9.80E-04	7.70E-03
NMT2	0.34	1.26	9.85E-04	7.74E-03
CENPH	-0.33	0.80	9.89E-04	7.77E-03
AFF4	0.25	1.19	9.90E-04	7.77E-03
EPRS	-0.22	0.86	9.94E-04	7.79E-03
FRS2	0.31	1.24	9.96E-04	7.80E-03
CST3	-0.25	0.84	9.99E-04	7.83E-03
SH2D2A	-0.49	0.71	1.00E-03	7.84E-03
DUSP7	-0.25	0.84	1.01E-03	7.90E-03
SNHG19	-0.61	0.65	1.01E-03	7.90E-03
KLK12	-0.58	0.67	1.02E-03	7.97E-03
LRRC59	0.19	1.14	1.02E-03	7.97E-03
EIF2AK3	-0.40	0.76	1.02E-03	7.98E-03
EPHA4	-0.57	0.67	1.02E-03	7.98E-03
IREB2	0.28	1.21	1.03E-03	8.01E-03
AMOTL1	0.21	1.15	1.03E-03	8.02E-03
PTCH1	-0.49	0.71	1.03E-03	8.02E-03
DAB2IP	0.25	1.19	1.03E-03	8.03E-03
TIMM9	0.36	1.29	1.04E-03	8.12E-03
MED27	0.31	1.24	1.05E-03	8.15E-03
MOB1A	0.24	1.18	1.05E-03	8.15E-03
IGFL2	-0.32	0.80	1.05E-03	8.16E-03
DDN	-0.59	0.66	1.06E-03	8.19E-03
AES	-0.27	0.83	1.06E-03	8.22E-03
FBXO41	-0.42	0.75	1.06E-03	8.22E-03
MZT1	0.42	1.33	1.06E-03	8.24E-03
PCED1B	0.43	1.35	1.07E-03	8.25E-03
DERL1	0.28	1.22	1.07E-03	8.25E-03
LIPE	-0.42	0.75	1.07E-03	8.25E-03
OGG1	-0.37	0.77	1.07E-03	8.29E-03
GAS5	-0.29	0.82	1.07E-03	8.30E-03
IPO7	0.25	1.19	1.08E-03	8.31E-03
DNHD1	0.50	1.42	1.08E-03	8.34E-03
CACNB3	-0.35	0.79	1.08E-03	8.36E-03
TAB2	0.26	1.20	1.09E-03	8.37E-03
CHUK	0.33	1.25	1.10E-03	8.47E-03
DKK1	0.26	1.19	1.10E-03	8.49E-03
GALC	-0.47	0.72	1.11E-03	8.54E-03
PLEKHG1	-0.45	0.73	1.11E-03	8.54E-03
IL20RA	-0.37	0.77	1.11E-03	8.54E-03
C4orf3	-0.31	0.81	1.08E-04	8.68E-04
ANKRD35	-0.61	0.66	1.08E-04	8.68E-04
SAMD9L	-0.44	0.74	1.08E-04	8.69E-04
RRN3	0.33	1.26	1.08E-04	8.69E-04
PLEKHG2	-0.53	0.69	1.09E-04	8.73E-04
AUNIP	0.53	1.44	1.09E-04	8.76E-04
RCC1	0.36	1.28	1.09E-04	8.76E-04
TM9SF4	-0.28	0.82	1.10E-04	8.77E-04
SECISBP2	0.36	1.29	1.10E-04	8.77E-04
CLDN4	-0.40	0.76	1.10E-04	8.77E-04
MET	0.30	1.23	1.11E-04	8.84E-04
EIF4E	0.34	1.27	1.11E-04	8.85E-04
ERCC6L	0.50	1.41	1.11E-04	8.85E-04
ARMC1	0.34	1.26	1.11E-04	8.85E-04
TMEM229B	-0.58	0.67	1.11E-04	8.86E-04
OVCH2	-1.12	0.46	1.11E-04	8.88E-04
SPC25	0.61	1.53	1.12E-04	8.90E-04
RP4-773N10.4	-0.58	0.67	1.12E-04	8.92E-04
HMCES	0.32	1.24	1.13E-04	9.00E-04
SPICE1	-0.51	0.70	1.13E-04	9.00E-04
RIN3	-0.44	0.74	1.13E-04	9.00E-04
DCAF15	0.35	1.27	1.13E-04	9.01E-04
KIAA0895	0.47	1.39	1.14E-04	9.07E-04
RAC2	0.41	1.33	1.14E-04	9.08E-04
NCAPH2	0.35	1.27	1.15E-04	9.09E-04
RAB3D	-0.33	0.80	1.15E-04	9.13E-04
FMR1	0.36	1.28	1.16E-04	9.17E-04
SCN4B	-0.40	0.76	1.16E-04	9.19E-04
PLEKHN1	-0.40	0.76	1.17E-04	9.22E-04
PTPMT1	-1.15	0.45	1.17E-04	9.27E-04
ATAD3A	0.31	1.24	1.19E-04	9.38E-04
SNRPA1	0.34	1.26	1.19E-04	9.41E-04
ZMYM2	0.28	1.22	1.19E-04	9.41E-04
HCAR2	-0.40	0.76	1.20E-04	9.47E-04
CSE1L	0.32	1.25	1.20E-04	9.47E-04
ARFGEF1	0.28	1.22	1.21E-04	9.51E-04
DBN1	0.28	1.21	1.21E-04	9.51E-04
GSDMC	-0.35	0.78	1.21E-04	9.52E-04
ECI2	0.47	1.38	1.21E-04	9.55E-04
ADGRE5	0.44	1.36	1.23E-04	9.63E-04
POLE3	0.34	1.26	1.23E-04	9.67E-04
SLC23A2	-0.35	0.79	1.24E-04	9.71E-04
MBD5	-0.49	0.71	1.24E-04	9.71E-04
SFXN1	0.31	1.24	1.25E-04	9.77E-04
CCNG2	-0.32	0.80	1.25E-04	9.77E-04
SPRR1B	-0.86	0.55	1.25E-04	9.78E-04
TMEM198B	-0.60	0.66	1.25E-04	9.80E-04
AP1S3	-0.35	0.78	1.26E-04	9.83E-04
ASPM	0.34	1.27	1.26E-04	9.88E-04
FGF11	-0.66	0.63	1.27E-04	9.89E-04
ABCC4	0.45	1.36	1.28E-04	9.97E-04
NABP1	-0.39	0.76	1.29E-04	1.01E-03
RFNG	-0.37	0.77	1.30E-04	1.01E-03
CABLES2	0.46	1.38	1.30E-04	1.01E-03
C5orf15	0.32	1.25	1.30E-04	1.02E-03
CLMN	0.45	1.37	1.31E-04	1.02E-03
COL6A2	1.86	3.63	1.31E-04	1.02E-03
TSN	0.29	1.22	1.31E-04	1.02E-03
CENPU	0.37	1.29	1.32E-04	1.03E-03
PIF1	0.49	1.40	1.33E-04	1.03E-03
PHC2	-0.34	0.79	1.33E-04	1.03E-03
H3F3B	0.26	1.20	1.33E-04	1.03E-03
PCDHGB7	-1.28	0.41	1.33E-04	1.03E-03
KCTD10	-0.29	0.82	1.34E-04	1.04E-03
HDGF	0.24	1.18	1.34E-04	1.04E-03
ALOX12B	-0.96	0.51	1.34E-04	1.04E-03
TMPRSS13	-0.47	0.72	1.35E-04	1.05E-03
TNFRSF25	-0.35	0.79	1.36E-04	1.05E-03
MAPK1	0.26	1.20	1.38E-04	1.06E-03
PLIN3	0.30	1.23	1.38E-04	1.06E-03
RPS6KA1	0.28	1.22	1.38E-04	1.06E-03
MATR3	0.26	1.20	1.38E-04	1.07E-03
LAMTOR4	-0.34	0.79	1.39E-04	1.07E-03
NCOA3	0.28	1.21	1.39E-04	1.07E-03
MAP7D3	0.41	1.33	1.40E-04	1.08E-03
ARID3A	0.43	1.34	1.40E-04	1.08E-03
RB1CC1	0.32	1.25	1.41E-04	1.08E-03
ABCE1	0.29	1.22	1.41E-04	1.08E-03
GPAM	0.40	1.32	1.41E-04	1.08E-03
YES1	0.29	1.23	1.41E-04	1.09E-03
BAG1	-0.32	0.80	1.42E-04	1.09E-03
RP11-597D13.9	-1.15	0.45	1.42E-04	1.09E-03
NUS1	-0.32	0.80	1.43E-04	1.09E-03
KLHL15	0.65	1.56	1.43E-04	1.10E-03
ZNF655	-0.32	0.80	1.43E-04	1.10E-03
MAL2	0.29	1.22	1.45E-04	1.11E-03
SBNO1	0.30	1.23	1.45E-04	1.11E-03
PRKCE	0.65	1.56	1.45E-04	1.11E-03
DUT	0.39	1.31	1.45E-04	1.11E-03
BAG2	0.80	1.74	1.47E-04	1.12E-03

DHX36	0.28	1.22	1.12E-03	8.58E-03
PHC2	0.26	1.20	1.12E-03	8.58E-03
MT-ND1	0.50	1.41	1.12E-03	8.60E-03
STARD13	0.46	1.37	1.13E-03	8.63E-03
SON	0.19	1.14	1.13E-03	8.66E-03
SMARCA2	-0.26	0.83	1.13E-03	8.67E-03
SDAD1	0.28	1.21	1.13E-03	8.67E-03
GDF11	0.44	1.35	1.14E-03	8.70E-03
RP11-488P3.1	0.35	1.27	1.14E-03	8.70E-03
CDC42SE2	0.32	1.24	1.14E-03	8.70E-03
RARRES1	-0.57	0.67	1.14E-03	8.70E-03
SEH1L	0.26	1.19	1.14E-03	8.71E-03
C9orf40	-0.46	0.73	1.15E-03	8.75E-03
TBC1D10B	-0.29	0.82	1.15E-03	8.75E-03
R3HDM1	0.24	1.18	1.15E-03	8.78E-03
PDCD5	-0.24	0.85	1.16E-03	8.80E-03
ATRX	0.30	1.23	1.16E-03	8.81E-03
NT5DC2	-0.24	0.85	1.16E-03	8.81E-03
U2SURP	0.26	1.20	1.16E-03	8.81E-03
UBE2D3	0.21	1.16	1.16E-03	8.83E-03
ING3	0.46	1.37	1.16E-03	8.83E-03
WFDC5	-0.57	0.67	1.16E-03	8.83E-03
ZNF646	-0.35	0.79	1.17E-03	8.89E-03
FAM120C	-0.52	0.70	1.17E-03	8.89E-03
RP11-160E2.6	0.44	1.36	1.19E-03	NA
SS18L1	0.38	1.30	1.19E-03	9.05E-03
XBP1	-0.24	0.85	1.20E-03	9.10E-03
CERS2	0.25	1.19	1.20E-03	9.10E-03
QRSL1	0.31	1.24	1.21E-03	9.14E-03
TMEM30B	-0.30	0.81	1.21E-03	9.16E-03
ACTR1A	0.22	1.16	1.21E-03	9.16E-03
DYRK1A	-0.31	0.81	1.22E-03	9.19E-03
RBP1	-0.55	0.68	1.22E-03	9.23E-03
FLII	0.21	1.16	1.23E-03	9.30E-03
ZDHHC18	0.27	1.20	1.23E-03	9.31E-03
KRT13	-0.17	0.89	1.24E-03	9.31E-03
DNER	-0.40	0.76	1.24E-03	9.35E-03
GPBP1L1	0.22	1.17	1.24E-03	9.37E-03
CCDC8	-0.35	0.78	1.25E-03	9.42E-03
ULK1	0.38	1.30	1.25E-03	9.42E-03
KPRP	-0.57	0.67	1.25E-03	9.42E-03
UNC119	0.33	1.25	1.25E-03	9.42E-03
PERM1	-0.47	0.72	1.26E-03	9.42E-03
EXO1	-0.36	0.78	1.27E-03	9.56E-03
RPRD1A	0.29	1.22	1.28E-03	9.56E-03
CDC123	0.23	1.18	1.28E-03	9.60E-03
BAIAP2L1	0.24	1.18	1.28E-03	9.61E-03
PRSS3	-0.27	0.83	1.28E-03	9.61E-03
MRPS34	-0.26	0.83	1.29E-03	9.64E-03
ABLIM3	-0.37	0.78	1.29E-03	9.64E-03
DQX1	-0.60	0.66	1.29E-03	9.64E-03
ENDOU	-0.58	0.67	1.29E-03	9.64E-03
ING2	-0.41	0.75	1.29E-03	9.64E-03
CRY2	-0.42	0.75	1.29E-03	9.64E-03
UBR7	-0.31	0.81	1.29E-03	9.65E-03
CLEC16A	0.27	1.21	1.30E-03	9.68E-03
LY6E	-0.28	0.82	1.30E-03	9.68E-03
PYCARD	-0.38	0.77	1.31E-03	9.74E-03
ILF3	0.18	1.13	1.32E-03	9.78E-03
TBC1D31	0.43	1.35	1.32E-03	9.78E-03
CDC37	0.21	1.16	1.32E-03	9.78E-03
FAIM	-0.44	0.74	1.32E-03	9.79E-03
RCN1	-0.26	0.84	1.32E-03	9.79E-03
FLVCR1	-0.45	0.73	1.32E-03	9.79E-03
ZNF865	-0.42	0.75	1.32E-03	9.79E-03
ARHGAP27	-0.29	0.82	1.33E-03	9.82E-03
EIF4G3	0.20	1.15	1.33E-03	9.82E-03
DIP2B	0.22	1.17	1.33E-03	9.82E-03
RABEP1	0.26	1.20	1.33E-03	9.84E-03
MAP2K4	0.27	1.20	1.33E-03	9.84E-03
ACER3	0.28	1.21	1.33E-03	9.85E-03
E2F4	0.27	1.20	1.34E-03	9.86E-03
LRRC8C	0.30	1.23	1.34E-03	9.86E-03
JMJD1C	0.29	1.23	1.34E-03	9.86E-03
B4GALT3	0.29	1.22	1.34E-03	9.86E-03
GMFB	0.34	1.27	1.34E-03	9.90E-03
CYC1	-0.24	0.85	1.34E-03	9.90E-03
USP1	-0.32	0.80	1.35E-03	9.91E-03
CMTM7	0.29	1.23	1.35E-03	9.95E-03
TNS1	-0.60	0.66	1.35E-03	9.95E-03
INPP4A	0.33	1.26	1.36E-03	9.96E-03
CSRNP2	0.32	1.25	1.36E-03	9.98E-03
RAD21	0.24	1.18	1.36E-03	9.98E-03
BLOC1S3	-0.45	0.73	1.36E-03	9.99E-03
CHORDC1	0.35	1.27	1.36E-03	9.99E-03
MAP4K5	0.27	1.20	1.36E-03	9.99E-03
XYLT1	0.28	1.21	1.37E-03	1.00E-02
ARNTL2	0.27	1.21	1.37E-03	1.00E-02
PHTF2	0.34	1.27	1.37E-03	1.00E-02
GRPEL1	0.24	1.18	1.37E-03	1.00E-02
GSR	0.31	1.24	1.47E-04	1.12E-03
FAM20C	0.49	1.40	1.48E-04	1.13E-03
SERTAD1	-0.41	0.75	1.48E-04	1.13E-03
SLC25A28	-0.35	0.79	1.49E-04	1.13E-03
RAI14	0.32	1.25	1.49E-04	1.14E-03
FADS2	0.25	1.19	1.50E-04	1.14E-03
SPINK5	-0.27	0.83	1.50E-04	1.14E-03
SP110	-0.36	0.78	1.50E-04	1.14E-03
FASTKD2	0.30	1.23	1.51E-04	1.15E-03
POLL	-0.38	0.77	1.52E-04	1.15E-03
SNRPD3	0.28	1.21	1.52E-04	1.15E-03
GNL3	0.27	1.21	1.52E-04	1.16E-03
ATP5G1P6	-1.07	0.48	1.53E-04	1.16E-03
BAHCC1	0.41	1.33	1.53E-04	1.16E-03
DHRS1	-0.33	0.80	1.54E-04	1.16E-03
CCDC92	-0.42	0.75	1.54E-04	1.17E-03
RP11-356I2.4	-0.89	0.54	1.55E-04	1.17E-03
XRCC6	0.26	1.20	1.56E-04	1.18E-03
CDC23	0.32	1.25	1.57E-04	1.19E-03
TCF25	-0.35	0.79	1.59E-04	1.20E-03
PSMD2	0.27	1.21	1.59E-04	1.20E-03
FIGNL1	0.41	1.33	1.61E-04	1.21E-03
MRPS35	0.31	1.24	1.62E-04	1.22E-03
CALML3-AS1	-0.59	0.67	1.62E-04	1.22E-03
TMPRSS11D	-0.37	0.77	1.62E-04	1.22E-03
BAZ1B	0.27	1.21	1.62E-04	1.22E-03
PABPN1	-0.44	0.74	1.63E-04	1.22E-03
CD274	-0.48	0.72	1.64E-04	1.23E-03
ZNF254	0.49	1.40	1.65E-04	1.24E-03
CDV3	0.26	1.20	1.66E-04	1.25E-03
ATL3	0.28	1.22	1.67E-04	1.25E-03
MAK16	0.40	1.32	1.67E-04	1.25E-03
ABHD14B	-0.37	0.77	1.67E-04	1.25E-03
BGN	1.38	2.60	1.69E-04	1.26E-03
STK36	-0.39	0.77	1.70E-04	1.27E-03
DOPEY1	-0.33	0.80	1.70E-04	1.27E-03
VAMP2	-0.44	0.74	1.70E-04	1.27E-03
PSMA3	0.28	1.22	1.71E-04	1.28E-03
YAP1	0.26	1.20	1.71E-04	1.28E-03
PACSIN2	0.29	1.22	1.71E-04	1.28E-03
CTD-2267D19.3	-0.88	0.54	1.72E-04	1.28E-03
AHNAK	-0.24	0.84	1.72E-04	1.28E-03
CRTAP	0.26	1.20	1.73E-04	1.29E-03
DOCK10	0.81	1.75	1.73E-04	1.29E-03
BTBD1	0.30	1.23	1.74E-04	1.29E-03
ARHGAP35	-0.26	0.84	1.74E-04	1.30E-03
VEGFB	0.36	1.28	1.75E-04	1.30E-03
CNOT3	-0.35	0.79	1.76E-04	1.31E-03
CYBA	0.45	1.37	1.76E-04	1.31E-03
GAB2	-0.59	0.66	1.78E-04	1.32E-03
TRIM66	-0.47	0.72	1.78E-04	1.32E-03
AKR7A2	-0.34	0.79	1.79E-04	1.32E-03
GNG12	-0.28	0.82	1.79E-04	1.33E-03
ROCK2	0.29	1.22	1.80E-04	1.33E-03
MED19	-0.60	0.66	1.80E-04	1.33E-03
PCYT1A	-0.27	0.83	1.81E-04	1.34E-03
ZNF714	0.49	1.40	1.81E-04	1.34E-03
NSUN5P1	-0.46	0.73	1.82E-04	1.34E-03
ANKRD29	-0.49	0.71	1.82E-04	1.35E-03
C1orf112	0.48	1.39	1.83E-04	1.35E-03
RFK	0.41	1.33	1.84E-04	1.36E-03
FPGS	-0.31	0.81	1.84E-04	1.36E-03
POLE	0.35	1.28	1.84E-04	1.36E-03
TICRR	0.39	1.31	1.85E-04	1.36E-03
C19orf66	-0.41	0.75	1.85E-04	1.37E-03
OCRL	-0.29	0.82	1.86E-04	1.37E-03
PBX3	0.40	1.32	1.88E-04	1.38E-03
NCL	0.28	1.21	1.88E-04	1.38E-03
PLCB3	-0.29	0.82	1.88E-04	1.38E-03
MIS18A	0.47	1.39	1.89E-04	1.39E-03
TNFRSF18	1.18	2.27	1.89E-04	1.39E-03
PPTC7	0.28	1.21	1.90E-04	1.40E-03
RP4-717I23.3	-0.57	0.67	1.91E-04	1.40E-03
LYN	0.39	1.31	1.91E-04	1.40E-03
TAF6	-0.42	0.75	1.93E-04	1.42E-03
NUPR1	-0.32	0.80	1.93E-04	1.42E-03
UBAP2L	0.26	1.19	1.95E-04	1.42E-03
OSBP2	-0.38	0.77	1.96E-04	1.43E-03
RNF217	-0.28	0.82	1.96E-04	1.43E-03
HMGA1	-0.32	0.80	1.97E-04	1.44E-03
FAM98C	-0.55	0.68	1.98E-04	1.45E-03
RP11-654A16.3	-1.00	0.50	1.99E-04	1.45E-03
AP1G2	-0.32	0.80	2.00E-04	1.46E-03
CENPI	0.48	1.40	2.00E-04	1.46E-03
DCBLD1	-0.29	0.82	2.00E-04	1.46E-03
OAS3	-0.25	0.84	2.01E-04	1.46E-03
SYT7	0.50	1.41	2.01E-04	1.46E-03
SLC25A1	-0.29	0.82	2.02E-04	1.47E-03
DSE	-0.27	0.83	2.02E-04	1.47E-03
TOPBP1	0.31	1.24	2.02E-04	1.47E-03

**MYBL2**	-0.25	0.84	1.37E-03	1.00E-02
**CHERP**	0.31	1.24	1.38E-03	1.01E-02
**ADD1**	0.20	1.15	1.38E-03	1.01E-02
**CD27-AS1**	-0.59	0.66	1.38E-03	1.01E-02
**AGPAT2**	-0.30	0.81	1.38E-03	1.01E-02
**AK7**	-0.56	0.68	1.39E-03	1.01E-02
**TBC1D9B**	0.24	1.18	1.39E-03	1.01E-02
**HECTD4**	0.30	1.23	1.39E-03	1.01E-02
**SGMS2**	0.34	1.27	1.39E-03	1.01E-02
**ARGLU1**	0.28	1.22	1.39E-03	1.01E-02
**BLVRB**	-0.28	0.83	1.40E-03	1.01E-02
**MZT2A**	-0.33	0.80	1.41E-03	1.03E-02
**LZTS2**	-0.26	0.83	1.42E-03	1.03E-02
**IFITM2**	-0.46	0.73	1.42E-03	1.03E-02
**C16orf52**	0.43	1.35	1.42E-03	1.03E-02
**STX2**	0.40	1.32	1.42E-03	1.03E-02
**CDR2**	0.32	1.25	1.42E-03	1.03E-02
**NGDN**	0.30	1.24	1.42E-03	1.03E-02
**EPB41L1**	-0.43	0.74	1.42E-03	1.03E-02
**DDX23**	0.22	1.16	1.43E-03	1.03E-02
**HERC5**	-0.59	0.67	1.43E-03	1.03E-02
**RNF213**	-0.27	0.83	1.43E-03	1.03E-02
**TRMT10C**	0.33	1.25	1.43E-03	1.03E-02
**RPL17**	-0.44	0.74	1.43E-03	1.03E-02
**GNG12**	0.23	1.18	1.43E-03	1.03E-02
**TRIM66**	0.48	1.39	1.44E-03	1.04E-02
**LINC00630**	0.59	1.50	1.45E-03	1.05E-02
**CBWD1**	0.47	1.39	1.46E-03	1.05E-02
**C1orf52**	0.39	1.31	1.47E-03	1.06E-02
**DANCR**	-0.33	0.79	1.47E-03	1.06E-02
**FUT2**	-0.42	0.75	1.48E-03	1.06E-02
**ZFR**	0.21	1.16	1.48E-03	1.06E-02
**STK39**	0.28	1.22	1.48E-03	1.06E-02
**KAZALD1**	-0.45	0.73	1.48E-03	NA
**C7orf31**	-0.58	0.67	1.49E-03	1.07E-02
**YTHDF1**	0.28	1.21	1.49E-03	1.07E-02
**KLF6**	-0.21	0.87	1.49E-03	1.07E-02
**CFAP97**	0.29	1.22	1.49E-03	1.07E-02
**ALOX12**	-0.59	0.66	1.50E-03	1.07E-02
**HSP90AA1**	0.21	1.16	1.51E-03	1.08E-02
**UBR1**	0.30	1.23	1.51E-03	1.08E-02
**ZNF638**	0.28	1.22	1.51E-03	1.08E-02
**LEPR**	0.46	1.38	1.52E-03	1.09E-02
**BAG1**	-0.27	0.83	1.53E-03	1.09E-02
**GDE1**	0.24	1.18	1.53E-03	1.09E-02
**ACACB**	-0.58	0.67	1.53E-03	1.09E-02
**C6orf106**	0.20	1.15	1.54E-03	1.10E-02
**FAM135A**	0.25	1.19	1.55E-03	1.10E-02
**RHOU**	-0.58	0.67	1.55E-03	1.11E-02
**SERPINB4**	-0.59	0.67	1.56E-03	1.11E-02
**ZUFSP**	0.40	1.32	1.56E-03	1.11E-02
**KPNA3**	0.25	1.19	1.57E-03	1.12E-02
**EDNRA**	-0.52	0.70	1.58E-03	1.12E-02
**RASAL2**	0.26	1.20	1.58E-03	1.12E-02
**TMEM259**	0.27	1.21	1.58E-03	1.12E-02
**SRSF6**	-0.23	0.85	1.59E-03	1.13E-02
**EHBP1L1**	-0.23	0.85	1.62E-03	1.15E-02
**GNA11**	0.24	1.18	1.63E-03	1.15E-02
**XDH**	-0.24	0.85	1.63E-03	1.15E-02
**SSR4**	-0.23	0.85	1.63E-03	1.16E-02
**AP3M1**	0.23	1.18	1.63E-03	1.16E-02
**CTNNAL1**	0.29	1.22	1.64E-03	1.16E-02
**PAK1IP1**	0.32	1.25	1.64E-03	1.16E-02
**ANO1**	-0.36	0.78	1.64E-03	1.16E-02
**ENTPD6**	0.26	1.20	1.68E-03	1.19E-02
**MARK3**	0.21	1.15	1.69E-03	1.19E-02
**KLHL3**	-0.58	0.67	1.69E-03	1.19E-02
**AKAP12**	0.57	1.48	1.71E-03	1.21E-02
**CEP55**	0.25	1.19	1.71E-03	1.21E-02
**URB1**	0.24	1.18	1.72E-03	1.21E-02
**NRAS**	0.24	1.18	1.72E-03	1.21E-02
**SIAH2**	0.25	1.19	1.72E-03	1.21E-02
**SYK**	-0.26	0.84	1.72E-03	1.21E-02
**USP7**	0.21	1.16	1.73E-03	1.22E-02
**UTP14A**	0.27	1.20	1.73E-03	1.22E-02
**SAMD12**	-0.53	0.69	1.74E-03	1.22E-02
**SLC3A2**	-0.23	0.85	1.74E-03	1.22E-02
**GGA3**	0.27	1.20	1.74E-03	1.22E-02
**CEMIP**	-0.49	0.71	1.75E-03	1.23E-02
**FAM49B**	0.24	1.18	1.76E-03	1.24E-02
**SQLE**	-0.23	0.85	1.76E-03	1.24E-02
**NECAP2**	0.26	1.19	1.77E-03	1.24E-02
**TMEM200B**	-0.45	0.73	1.78E-03	NA
**FMN1**	-0.47	0.72	1.78E-03	1.24E-02
**PSMB8**	-0.39	0.76	1.78E-03	1.24E-02
**BNIP3L**	0.33	1.26	1.78E-03	1.24E-02
**LUC7L**	0.33	1.26	1.78E-03	1.24E-02
**HAUS2**	-0.32	0.80	1.78E-03	1.25E-02
**PLCH2**	0.24	1.18	1.79E-03	1.25E-02
**COX5A**	-0.25	0.84	1.80E-03	1.25E-02
**COIL**	0.37	1.29	2.02E-04	1.47E-03
**RUSC1-AS1**	-0.44	0.74	2.03E-04	1.47E-03
**VAPA**	0.27	1.20	2.04E-04	1.48E-03
**MTIF2**	0.30	1.23	2.04E-04	1.48E-03
**ACTR1B**	-0.31	0.80	2.05E-04	1.48E-03
**NLN**	0.32	1.25	2.05E-04	1.48E-03
**ZNF141**	0.72	1.65	2.06E-04	1.49E-03
**NGFR**	0.95	1.94	2.07E-04	1.50E-03
**GAL3ST4**	-0.57	0.68	2.08E-04	1.50E-03
**IFITM1**	-0.34	0.79	2.08E-04	1.51E-03
**LEO1**	0.32	1.25	2.11E-04	1.52E-03
**NUP93**	0.29	1.22	2.12E-04	1.53E-03
**COQ5**	0.35	1.27	2.12E-04	1.53E-03
**SF3B2**	0.25	1.19	2.13E-04	1.54E-03
**DNM1**	0.86	1.82	2.14E-04	1.54E-03
**SYNCRIP**	0.26	1.20	2.14E-04	1.54E-03
**UBE2V2**	0.32	1.25	2.15E-04	1.55E-03
**HPRT1**	0.37	1.29	2.16E-04	1.55E-03
**MARK4**	0.29	1.23	2.16E-04	1.55E-03
**WNT5A**	-0.37	0.78	2.16E-04	1.55E-03
**CDK17**	0.36	1.28	2.17E-04	1.56E-03
**DIAPH3**	0.39	1.31	2.18E-04	1.57E-03
**CDC20**	0.73	1.66	2.18E-04	1.57E-03
**CPD**	0.27	1.20	2.19E-04	1.57E-03
**MACF1**	-0.24	0.85	2.20E-04	1.58E-03
**CES3**	-0.62	0.65	2.21E-04	1.58E-03
**CPSF6**	0.28	1.22	2.21E-04	1.58E-03
**SKIV2L2**	0.27	1.21	2.21E-04	1.58E-03
**PPIL2**	-0.32	0.80	2.21E-04	1.58E-03
**ZNF107**	0.40	1.32	2.22E-04	1.59E-03
**NBL1**	-0.45	0.73	2.23E-04	1.59E-03
**RGS14**	0.39	1.31	2.24E-04	1.60E-03
**PLEKHG1**	-0.62	0.65	2.25E-04	1.61E-03
**TUBGCP3**	0.35	1.27	2.26E-04	1.61E-03
**NAA15**	0.28	1.21	2.26E-04	1.61E-03
**SFN**	-0.23	0.85	2.27E-04	1.62E-03
**NSMF**	-0.37	0.77	2.27E-04	1.62E-03
**EPHA2**	-0.25	0.84	2.29E-04	1.63E-03
**NAPRT**	0.36	1.29	2.29E-04	1.63E-03
**CDC20P1**	0.78	1.71	2.30E-04	1.64E-03
**EME1**	0.57	1.48	2.31E-04	1.64E-03
**TMEM143**	-0.70	0.62	2.32E-04	1.65E-03
**PCSK7**	-0.30	0.81	2.34E-04	1.66E-03
**TRIM5**	-0.30	0.81	2.35E-04	1.66E-03
**RP11-673C5.1**	0.64	1.56	2.36E-04	1.68E-03
**RAB31**	0.33	1.26	2.38E-04	1.68E-03
**CASC5**	0.40	1.32	2.38E-04	1.68E-03
**LRR1**	0.47	1.38	2.38E-04	1.68E-03
**HSD11B1L**	-0.85	0.55	2.39E-04	1.69E-03
**KIF1BP**	0.31	1.24	2.39E-04	1.69E-03
**KLHL29**	-0.41	0.75	2.42E-04	1.71E-03
**CFDP1**	0.40	1.32	2.44E-04	1.72E-03
**FRMD8**	-0.29	0.82	2.44E-04	1.73E-03
**MALT1**	0.29	1.22	2.46E-04	1.73E-03
**GSTO1**	0.28	1.22	2.47E-04	1.74E-03
**CERS3**	-0.28	0.82	2.48E-04	1.75E-03
**CKAP5**	0.28	1.21	2.49E-04	1.75E-03
**RBMX**	0.26	1.20	2.49E-04	1.75E-03
**PLBD1**	-0.47	0.72	2.50E-04	1.76E-03
**RP11-66B24.4**	-0.70	0.62	2.50E-04	1.76E-03
**FNBP1**	-0.38	0.77	2.51E-04	1.77E-03
**MTAP**	0.30	1.23	2.51E-04	1.77E-03
**PYCR1**	-0.52	0.70	2.52E-04	1.77E-03
**PCDH7**	-0.35	0.78	2.53E-04	1.78E-03
**TMEM147-AS1**	-0.57	0.67	2.53E-04	1.78E-03
**KLHL13**	0.46	1.38	2.55E-04	1.79E-03
**LTBP4**	-0.30	0.81	2.55E-04	1.79E-03
**YWHAE**	0.24	1.18	2.55E-04	1.79E-03
**RAPGEF2**	-0.36	0.78	2.56E-04	1.79E-03
**ATP5L**	-0.32	0.80	2.58E-04	1.80E-03
**CD2AP**	0.27	1.21	2.59E-04	1.81E-03
**TPRG1**	0.91	1.89	2.59E-04	1.81E-03
**UBTF**	-0.32	0.80	2.59E-04	1.81E-03
**NOP14**	-0.36	0.78	2.60E-04	1.82E-03
**TMUB2**	-0.33	0.79	2.60E-04	1.82E-03
**ZNF79**	-0.52	0.70	2.61E-04	1.82E-03
**RAN**	0.25	1.19	2.62E-04	1.82E-03
**ADAMTS7**	-0.88	0.55	2.63E-04	1.83E-03
**SNRPD1**	0.33	1.26	2.63E-04	1.83E-03
**RUSC2**	-0.36	0.78	2.63E-04	1.83E-03
**CCK**	1.57	2.98	2.64E-04	1.84E-03
**GMNN**	0.38	1.30	2.66E-04	1.85E-03
**CSRP1**	-0.27	0.83	2.67E-04	1.85E-03
**RASA3**	1.23	2.35	2.67E-04	1.86E-03
**CEP78**	0.39	1.31	2.68E-04	1.86E-03
**FURIN**	0.24	1.18	2.68E-04	1.86E-03
**ZNF692**	-0.36	0.78	2.69E-04	1.86E-03
**SNHG12**	-0.39	0.76	2.69E-04	1.87E-03
**LIMS1**	0.28	1.22	2.71E-04	1.88E-03
**SLF2**	0.32	1.25	2.71E-04	1.88E-03

**RAP1GAP**	-0.38	0.77	1.80E-03	1.25E-02
**SMIM5**	-0.57	0.68	1.80E-03	1.25E-02
**LPAR6**	-0.46	0.73	1.80E-03	1.26E-02
**ANKFY1**	0.23	1.17	1.80E-03	1.26E-02
**B3GNT5**	0.27	1.20	1.80E-03	1.26E-02
**TP63**	-0.19	0.88	1.80E-03	1.26E-02
**ASAP3**	-0.30	0.81	1.81E-03	1.26E-02
**SLITRK6**	-0.58	0.67	1.81E-03	1.26E-02
**SYTL1**	-0.28	0.82	1.82E-03	1.27E-02
**ZZZ3**	0.30	1.23	1.83E-03	1.27E-02
**ATP2B1**	0.26	1.20	1.84E-03	1.28E-02
**PPP2R2A**	0.22	1.16	1.84E-03	1.28E-02
**ABLIM1**	-0.21	0.86	1.84E-03	1.28E-02
**SMARCD1**	0.21	1.15	1.85E-03	1.28E-02
**C15orf52**	0.27	1.20	1.85E-03	1.28E-02
**AP001046.5**	-0.58	0.67	1.85E-03	1.28E-02
**NINJ1**	-0.45	0.73	1.85E-03	1.28E-02
**SAP30BP**	0.23	1.18	1.86E-03	1.28E-02
**SOX4**	-0.30	0.81	1.86E-03	1.29E-02
**FAM73A**	0.32	1.25	1.87E-03	1.29E-02
**PERP**	-0.18	0.88	1.87E-03	1.29E-02
**CEACAM6**	-0.28	0.83	1.88E-03	1.30E-02
**IFT46**	-0.32	0.80	1.88E-03	1.30E-02
**ATP11A**	0.28	1.22	1.89E-03	1.30E-02
**ADGRF4**	-0.27	0.83	1.90E-03	1.31E-02
**SETD2**	0.22	1.16	1.90E-03	1.31E-02
**ARIH1**	0.25	1.19	1.91E-03	1.31E-02
**MPZL2**	0.21	1.16	1.92E-03	1.32E-02
**PDK2**	-0.55	0.68	1.92E-03	1.32E-02
**KIFC3**	0.26	1.20	1.92E-03	1.32E-02
**EEA1**	0.27	1.21	1.92E-03	1.32E-02
**LINC01547**	-0.58	0.67	1.92E-03	1.32E-02
**CCNI**	0.19	1.14	1.93E-03	1.32E-02
**EXOSC10**	0.21	1.15	1.96E-03	1.34E-02
**RTF1**	0.23	1.17	1.96E-03	1.35E-02
**SDSL**	-0.56	0.68	1.96E-03	1.35E-02
**BDP1**	0.25	1.19	1.98E-03	1.36E-02
**EXTL3**	0.22	1.17	1.98E-03	1.36E-02
**SHTN1**	0.26	1.20	1.99E-03	1.36E-02
**PCYOX1L**	-0.43	0.74	1.99E-03	1.36E-02
**RNF11**	0.23	1.18	1.99E-03	1.36E-02
**HPGD**	-0.49	0.71	2.00E-03	1.36E-02
**TRAIP**	0.38	1.30	2.00E-03	1.37E-02
**ANKRD9**	-0.40	0.76	2.00E-03	1.37E-02
**PLP2**	-0.21	0.86	2.01E-03	1.37E-02
**IFITM1**	-0.40	0.76	2.01E-03	1.37E-02
**ZNF662**	-0.57	0.67	2.01E-03	1.37E-02
**VGLL3**	0.27	1.21	2.02E-03	1.38E-02
**POP1**	0.24	1.18	2.04E-03	1.39E-02
**YTHDF3**	0.24	1.18	2.05E-03	1.40E-02
**RRM2B**	0.35	1.28	2.05E-03	1.40E-02
**DMPK**	0.45	1.37	2.05E-03	1.40E-02
**ICMT**	-0.20	0.87	2.05E-03	1.40E-02
**RBL2**	0.27	1.21	2.06E-03	1.40E-02
**GCFC2**	0.38	1.30	2.07E-03	1.40E-02
**ARMC5**	-0.45	0.73	2.07E-03	1.40E-02
**DFNA5**	0.23	1.17	2.07E-03	1.40E-02
**ANAPC5**	0.20	1.15	2.08E-03	1.41E-02
**ORAOV1**	0.35	1.28	2.08E-03	1.41E-02
**YEATS2**	0.23	1.17	2.08E-03	1.41E-02
**RIBC2**	-0.52	0.70	2.09E-03	1.41E-02
**FRMD6**	0.23	1.17	2.10E-03	1.42E-02
**MTMR11**	-0.44	0.74	2.11E-03	1.43E-02
**PHLPP2**	0.39	1.31	2.11E-03	1.43E-02
**SLC26A2**	-0.37	0.77	2.12E-03	1.43E-02
**KIF21B**	-0.56	0.68	2.12E-03	1.43E-02
**FUT1**	-0.46	0.73	2.12E-03	1.43E-02
**TRPC4AP**	0.24	1.18	2.13E-03	1.43E-02
**S1PR3**	0.53	1.45	2.14E-03	1.44E-02
**ADA**	-0.35	0.78	2.15E-03	1.45E-02
**MPP1**	-0.55	0.68	2.15E-03	1.45E-02
**SEL1L**	0.25	1.19	2.15E-03	1.45E-02
**CCSER2**	0.26	1.19	2.15E-03	1.45E-02
**FRS3**	-0.57	0.67	2.15E-03	1.45E-02
**GBA**	-0.32	0.80	2.16E-03	1.45E-02
**MKL2**	0.27	1.20	2.16E-03	1.45E-02
**FGFR4**	-0.53	0.69	2.16E-03	1.45E-02
**LINC01588**	-0.57	0.67	2.17E-03	1.46E-02
**MON2**	0.28	1.22	2.17E-03	1.46E-02
**TSG101**	0.22	1.17	2.17E-03	1.46E-02
**LOXL1-AS1**	-0.56	0.68	2.18E-03	1.46E-02
**YTHDF2**	0.23	1.17	2.18E-03	1.46E-02
**RNPS1**	0.22	1.17	2.18E-03	1.46E-02
**CEP135**	0.41	1.33	2.20E-03	1.47E-02
**MARS**	-0.20	0.87	2.20E-03	1.47E-02
**ELMO3**	-0.29	0.82	2.20E-03	1.47E-02
**RHBDF2**	0.27	1.20	2.21E-03	1.48E-02
**MEIS1**	-0.41	0.75	2.22E-03	1.48E-02
**ABHD17C**	0.25	1.19	2.22E-03	1.48E-02
**BARX2**	-0.26	0.84	2.22E-03	1.48E-02
**AMPD3**	0.44	1.36	2.72E-04	1.88E-03
**RSF1**	0.30	1.23	2.72E-04	1.88E-03
**MYBL1**	0.50	1.42	2.72E-04	1.88E-03
**MLLT11**	-0.46	0.72	2.72E-04	1.88E-03
**MDK**	0.42	1.33	2.74E-04	1.89E-03
**BDKRB2**	0.30	1.23	2.74E-04	1.89E-03
**HELZ2**	-0.26	0.83	2.75E-04	1.90E-03
**MCMBP**	0.29	1.23	2.79E-04	1.92E-03
**PCED1A**	-0.43	0.74	2.79E-04	1.92E-03
**SLBP**	0.31	1.24	2.80E-04	1.93E-03
**ATP13A3**	0.26	1.20	2.81E-04	1.93E-03
**ST3GAL4**	0.35	1.27	2.81E-04	1.94E-03
**UROS**	0.32	1.25	2.82E-04	1.94E-03
**QPCTL**	-0.47	0.72	2.82E-04	1.94E-03
**SH3BGRL**	-0.30	0.81	2.82E-04	1.94E-03
**SLC35B4**	0.35	1.28	2.83E-04	1.94E-03
**CHORDC1**	0.33	1.25	2.83E-04	1.94E-03
**VASP**	0.26	1.20	2.83E-04	1.94E-03
**AMMECR1**	0.29	1.22	2.86E-04	1.96E-03
**ARFGAP1**	-0.27	0.83	2.87E-04	1.97E-03
**SFXN4**	0.42	1.34	2.90E-04	1.99E-03
**AGPAT5**	0.33	1.25	2.90E-04	1.99E-03
**ENPP5**	1.16	2.24	2.93E-04	2.00E-03
**TSR1**	0.31	1.24	2.93E-04	2.00E-03
**S100A8**	-0.31	0.81	2.93E-04	2.01E-03
**PGRMC1**	0.29	1.22	2.97E-04	2.03E-03
**MRS2**	0.32	1.25	2.97E-04	2.03E-03
**CAPN15**	-0.37	0.77	2.99E-04	2.04E-03
**RFC5**	0.38	1.30	2.99E-04	2.04E-03
**SEC24A**	0.30	1.24	2.99E-04	2.04E-03
**SEMA3G**	-1.19	0.44	3.00E-04	2.05E-03
**BBOX1**	-0.52	0.70	3.01E-04	2.05E-03
**DSG3**	-0.25	0.84	3.02E-04	2.06E-03
**LRPPRC**	0.26	1.20	3.02E-04	2.06E-03
**BLOC1S2**	-0.31	0.81	3.02E-04	2.06E-03
**NUCKS1**	0.28	1.22	3.02E-04	2.06E-03
**RP11-342K6.1**	0.51	1.42	3.03E-04	2.06E-03
**SP6**	-0.42	0.75	3.03E-04	2.06E-03
**IMPDH2**	0.26	1.20	3.03E-04	2.06E-03
**MVP**	-0.24	0.84	3.04E-04	2.06E-03
**SLC12A7**	0.52	1.44	3.06E-04	2.08E-03
**HCG18**	-0.46	0.73	3.07E-04	2.08E-03
**MTUS1**	0.28	1.22	3.07E-04	2.08E-03
**BCL2L2**	-0.30	0.81	3.07E-04	2.08E-03
**BRWD1**	0.28	1.21	3.09E-04	2.09E-03
**CSNK2A1**	0.26	1.20	3.09E-04	2.09E-03
**ZSWIM8**	-0.29	0.82	3.10E-04	2.10E-03
**EFR3A**	0.27	1.20	3.11E-04	2.10E-03
**CORO2A**	-0.32	0.80	3.11E-04	2.10E-03
**PRKAR2A**	0.27	1.21	3.11E-04	2.10E-03
**SGMS2**	0.31	1.24	3.12E-04	2.11E-03
**SRPRB**	0.29	1.22	3.13E-04	2.11E-03
**ADO**	-0.38	0.77	3.14E-04	2.11E-03
**RNU6-1**	-1.95	0.26	3.15E-04	NA
**POLA2**	0.36	1.29	3.17E-04	2.14E-03
**KPNB1**	0.25	1.19	3.17E-04	2.14E-03
**ABLIM1**	-0.27	0.83	3.18E-04	2.14E-03
**BBS2**	-0.40	0.76	3.19E-04	2.15E-03
**AES**	-0.27	0.83	3.20E-04	2.15E-03
**FBXW7**	-0.32	0.80	3.20E-04	2.16E-03
**NUP107**	0.37	1.29	3.21E-04	2.16E-03
**DNAJC2**	0.37	1.29	3.23E-04	2.17E-03
**EIF4A3**	0.27	1.21	3.24E-04	2.17E-03
**SYTL1**	-0.27	0.83	3.24E-04	2.18E-03
**KHDRBS1**	0.26	1.20	3.27E-04	2.19E-03
**ENC1**	-0.36	0.78	3.31E-04	2.22E-03
**RBBP7**	0.26	1.19	3.31E-04	2.22E-03
**FKBP4**	0.26	1.20	3.33E-04	2.23E-03
**LRP5**	0.27	1.21	3.33E-04	2.23E-03
**HRH1**	0.51	1.42	3.35E-04	2.24E-03
**TPP1**	-0.27	0.83	3.35E-04	2.24E-03
**HAUS6**	0.33	1.25	3.35E-04	2.24E-03
**TPM3**	0.25	1.19	3.40E-04	2.27E-03
**HSF4**	-0.82	0.56	3.41E-04	2.27E-03
**COL17A1**	-0.23	0.86	3.42E-04	2.28E-03
**OTUD4**	0.27	1.20	3.43E-04	2.29E-03
**MRPL57**	-0.36	0.78	3.44E-04	2.29E-03
**TLL1**	0.44	1.35	3.44E-04	2.29E-03
**MCM5**	0.35	1.28	3.44E-04	2.29E-03
**XPOT**	0.27	1.21	3.45E-04	2.30E-03
**SDCBP2**	-0.48	0.72	3.45E-04	2.30E-03
**GOLGA8A**	-0.34	0.79	3.51E-04	2.33E-03
**PLEKHM2**	-0.27	0.83	3.51E-04	2.33E-03
**SUMO2**	0.27	1.20	3.52E-04	2.34E-03
**USP9Y**	-0.27	0.83	3.52E-04	2.34E-03
**D2HGDH**	-0.37	0.77	3.52E-04	2.34E-03
**LTV1**	0.41	1.33	3.52E-04	2.34E-03
**GRINA**	-0.27	0.83	3.55E-04	2.36E-03
**SLC35F5**	0.30	1.23	3.56E-04	2.36E-03
**ARPC1B**	0.28	1.22	3.56E-04	2.36E-03

RPS23	-0.26	0.84	2.22E-03	1.48E-02
ODC1	0.19	1.14	2.22E-03	1.48E-02
SAMD4B	0.26	1.19	2.23E-03	1.48E-02
MED10	0.31	1.24	2.23E-03	1.48E-02
HS6ST2	-0.49	0.71	2.23E-03	1.49E-02
ABHD4	-0.34	0.79	2.23E-03	1.49E-02
TRA2A	0.27	1.21	2.24E-03	1.49E-02
MORN3	-0.54	0.69	2.24E-03	1.49E-02
LGALS7B	-0.55	0.68	2.24E-03	1.49E-02
PSMC3IP	-0.47	0.72	2.24E-03	1.49E-02
MYO1D	0.20	1.15	2.25E-03	1.49E-02
ZNF160	0.39	1.31	2.25E-03	1.49E-02
ELF3	-0.41	0.75	2.25E-03	1.49E-02
FADD	-0.52	0.70	2.25E-03	1.49E-02
MAFK	0.47	1.38	2.26E-03	1.50E-02
CCNY	0.23	1.17	2.26E-03	1.50E-02
ZNF703	-0.35	0.78	2.27E-03	1.50E-02
VRK3	0.33	1.26	2.28E-03	1.51E-02
RMND5A	0.28	1.21	2.29E-03	1.51E-02
CSNK1G1	0.29	1.22	2.29E-03	1.51E-02
METTL3	0.26	1.20	2.29E-03	1.52E-02
PGRMC1	-0.25	0.84	2.30E-03	1.52E-02
ZBTB14	-0.45	0.73	2.30E-03	1.52E-02
GNL2	0.22	1.17	2.31E-03	1.53E-02
RUNX1	0.27	1.21	2.33E-03	1.53E-02
PKN2	0.27	1.21	2.35E-03	1.55E-02
USP14	0.25	1.19	2.35E-03	1.55E-02
PADI1	-0.56	0.68	2.36E-03	1.55E-02
UBE2A	-0.23	0.85	2.36E-03	1.55E-02
SNRNP27	0.33	1.26	2.37E-03	1.56E-02
TPM4	0.17	1.13	2.38E-03	1.56E-02
DAZAP2	0.20	1.15	2.38E-03	1.56E-02
EXOC4	0.22	1.17	2.38E-03	1.56E-02
EFCAB14	0.19	1.14	2.38E-03	1.56E-02
SNX19	0.22	1.16	2.40E-03	1.57E-02
TTC28	-0.51	0.70	2.40E-03	1.57E-02
RNF114	0.26	1.19	2.40E-03	1.57E-02
SCYL2	0.25	1.19	2.40E-03	1.57E-02
RNF222	-0.54	0.69	2.41E-03	1.58E-02
MOGS	0.24	1.18	2.41E-03	1.58E-02
ATE1	0.26	1.20	2.42E-03	1.58E-02
EIF2A	0.24	1.18	2.42E-03	1.59E-02
CAMKK1	-0.44	0.74	2.43E-03	1.59E-02
CLPTM1L	0.21	1.16	2.44E-03	1.59E-02
SLC30A1	-0.33	0.80	2.44E-03	1.59E-02
TUB	-0.55	0.68	2.44E-03	1.59E-02
PPP1R12A	0.25	1.19	2.45E-03	1.60E-02
RYK	0.23	1.18	2.45E-03	1.60E-02
ACSS1	-0.57	0.68	2.46E-03	1.60E-02
PFDN2	0.25	1.19	2.46E-03	1.60E-02
IQGAP3	0.24	1.18	2.47E-03	1.61E-02
CLDND1	0.27	1.21	2.47E-03	1.61E-02
NIN	0.23	1.18	2.48E-03	1.61E-02
ADPRH	-0.57	0.68	2.48E-03	1.61E-02
RLF	0.32	1.25	2.48E-03	1.61E-02
ZNF425	-0.55	0.68	2.50E-03	1.62E-02
LMF2	-0.28	0.82	2.50E-03	1.62E-02
PRMT1	0.22	1.17	2.53E-03	1.64E-02
MED13	0.24	1.18	2.54E-03	1.64E-02
WNT7B	0.34	1.26	2.54E-03	1.65E-02
COTL1	0.19	1.14	2.54E-03	1.65E-02
NOL8	0.28	1.21	2.54E-03	1.65E-02
PRRT4	-0.56	0.68	2.55E-03	1.65E-02
SLC27A1	-0.48	0.72	2.55E-03	1.65E-02
PRR7	-0.54	0.69	2.56E-03	1.65E-02
SGPL1	-0.23	0.85	2.56E-03	1.66E-02
PLEKHH1	0.38	1.30	2.56E-03	1.66E-02
FBXO38	0.27	1.21	2.57E-03	1.66E-02
C11orf84	0.34	1.27	2.58E-03	1.66E-02
DHRS4-AS1	-0.38	0.77	2.58E-03	1.66E-02
PDIA5	0.29	1.23	2.58E-03	1.66E-02
CRELD2	-0.32	0.80	2.58E-03	1.66E-02
COPS2	0.26	1.20	2.60E-03	1.67E-02
ZNF335	0.28	1.21	2.60E-03	1.67E-02
ACAD10	0.35	1.28	2.60E-03	1.68E-02
KIF1C	0.18	1.14	2.61E-03	1.68E-02
AIM1L	-0.23	0.86	2.61E-03	1.68E-02
ZBTB39	-0.39	0.76	2.62E-03	1.68E-02
TNS3	-0.54	0.69	2.63E-03	1.69E-02
TMUB1	-0.32	0.80	2.63E-03	1.69E-02
NAAA	-0.37	0.77	2.65E-03	1.70E-02
RGS2	-0.24	0.85	2.67E-03	1.71E-02
DGAT2	-0.41	0.75	2.67E-03	1.71E-02
NCEH1	0.30	1.23	2.67E-03	1.71E-02
AP1B1	0.22	1.17	2.68E-03	1.72E-02
KRAS	0.31	1.24	2.69E-03	1.72E-02
NKPD1	-0.55	0.68	2.69E-03	1.72E-02
HACD4	-0.45	0.73	2.69E-03	1.72E-02
TAGAP	-0.44	0.74	2.70E-03	NA
CYP4B1	-0.56	0.68	2.71E-03	1.73E-02
SH2B1	-0.32	0.80	3.60E-04	2.38E-03
HSD17B7P2	-0.71	0.61	3.60E-04	2.39E-03
ZNF652	0.44	1.36	3.61E-04	2.39E-03
ARAP1	-0.27	0.83	3.62E-04	2.39E-03
IFI6	-0.25	0.84	3.62E-04	2.39E-03
HOXC12	0.63	1.55	3.63E-04	2.40E-03
SUDS3	0.27	1.21	3.63E-04	2.40E-03
RBMS2	-0.26	0.83	3.66E-04	2.42E-03
SCG5	1.01	2.01	3.67E-04	2.42E-03
CDC42EP4	0.28	1.21	3.69E-04	2.43E-03
CTIF	-0.31	0.81	3.69E-04	2.43E-03
ATP6V1C1	0.31	1.24	3.73E-04	2.46E-03
GALNT12	0.71	1.63	3.74E-04	2.46E-03
KMT2B	-0.31	0.81	3.74E-04	2.46E-03
TACSTD2	-0.24	0.85	3.75E-04	2.47E-03
APEX2	-0.32	0.80	3.76E-04	2.47E-03
ADD1	0.24	1.18	3.77E-04	2.47E-03
UBR7	-0.35	0.78	3.77E-04	2.48E-03
NUP153	0.28	1.22	3.79E-04	2.49E-03
METAP2	0.27	1.20	3.80E-04	2.49E-03
SPATA5	0.50	1.42	3.80E-04	2.49E-03
MYPN	-1.09	0.47	3.83E-04	2.51E-03
DSG1	-0.34	0.79	3.84E-04	2.51E-03
CBLB	-0.37	0.78	3.84E-04	2.51E-03
FAM234B	0.44	1.36	3.85E-04	2.52E-03
ANTXR1	0.29	1.22	3.85E-04	2.52E-03
SESTD1	0.33	1.26	3.86E-04	2.52E-03
HSPE1	0.43	1.35	3.86E-04	2.52E-03
CTD-2033D15.2	-0.83	0.56	3.88E-04	2.54E-03
PHF19	0.36	1.28	3.90E-04	2.54E-03
CRELD1	-0.38	0.77	3.91E-04	2.55E-03
ERC1	-0.25	0.84	3.93E-04	2.57E-03
PRICKLE4	-0.44	0.74	3.94E-04	2.57E-03
RBM3	0.26	1.19	3.95E-04	2.58E-03
SRSF3	0.26	1.20	3.97E-04	2.59E-03
HNRNPL	0.25	1.19	3.97E-04	2.59E-03
UTP6	0.30	1.23	3.98E-04	2.59E-03
SUSD1	0.79	1.73	4.02E-04	2.61E-03
PLEK2	0.32	1.25	4.02E-04	2.61E-03
MAPRE1	-0.25	0.84	4.02E-04	2.61E-03
BPGM	-0.37	0.77	4.03E-04	2.62E-03
MDH1	0.24	1.18	4.05E-04	2.63E-03
KRT24	-0.89	0.54	4.05E-04	2.63E-03
GABARAPL1	-0.42	0.75	4.08E-04	2.64E-03
MCM8	0.43	1.34	4.09E-04	2.65E-03
CTSZ	0.28	1.21	4.12E-04	2.67E-03
E2F8	0.43	1.34	4.15E-04	2.69E-03
DDX1	0.25	1.19	4.18E-04	2.71E-03
WASF1	0.33	1.26	4.19E-04	2.71E-03
BTF3L4	0.32	1.24	4.20E-04	2.72E-03
KREMEN2	0.52	1.44	4.21E-04	2.72E-03
IGF2R	0.23	1.17	4.22E-04	2.72E-03
FAM64A	0.51	1.43	4.23E-04	2.73E-03
FAM72A	0.76	1.70	4.24E-04	2.74E-03
SRP72	0.25	1.19	4.24E-04	2.74E-03
GALNT1	0.24	1.18	4.27E-04	2.75E-03
SLC36A1	-0.38	0.77	4.30E-04	2.77E-03
DDHD2	0.28	1.21	4.30E-04	2.77E-03
CCDC71	-0.36	0.78	4.31E-04	2.78E-03
SNRNP200	0.25	1.19	4.35E-04	2.80E-03
CACHD1	0.51	1.42	4.37E-04	2.81E-03
RPF2	0.37	1.29	4.38E-04	2.82E-03
SKI	0.28	1.22	4.39E-04	2.82E-03
DDIAS	0.41	1.33	4.40E-04	2.83E-03
NCOA5	-0.37	0.77	4.41E-04	2.83E-03
KIAA0556	-0.33	0.79	4.44E-04	2.85E-03
SFXN3	-0.31	0.80	4.45E-04	2.86E-03
NDFIP2	-0.24	0.85	4.46E-04	2.86E-03
BRCA2	0.43	1.35	4.46E-04	2.86E-03
TRAFD1	-0.29	0.82	4.48E-04	2.87E-03
GSTK1	-0.25	0.84	4.49E-04	2.87E-03
NSUN2	0.25	1.19	4.51E-04	2.88E-03
LCE1C	-1.12	0.46	4.52E-04	2.89E-03
RP11-235E17.6	-0.79	0.58	4.52E-04	2.89E-03
DDHD1	0.37	1.29	4.54E-04	2.90E-03
SYNPO2	-1.00	0.50	4.58E-04	2.92E-03
RDX	0.25	1.19	4.58E-04	2.92E-03
PSMD11	0.25	1.19	4.58E-04	2.92E-03
TIAM1	0.34	1.27	4.60E-04	2.93E-03
PITPNM2	-0.33	0.79	4.62E-04	2.94E-03
PRKAB1	-0.39	0.76	4.62E-04	2.94E-03
ZDHHC18	-0.30	0.81	4.62E-04	2.94E-03
CD24P4	-0.26	0.84	4.62E-04	2.94E-03
PDE12	0.29	1.22	4.63E-04	2.94E-03
DSN1	0.41	1.33	4.63E-04	2.94E-03
RNF4	0.27	1.21	4.64E-04	2.95E-03
H2AFX	0.34	1.27	4.68E-04	2.97E-03
NUP160	0.28	1.22	4.68E-04	2.97E-03
ACTL6A	0.31	1.24	4.69E-04	2.98E-03
DPF1	0.85	1.80	4.70E-04	2.98E-03

**BID**	0.27	1.20	2.71E-03	1.73E-02	**PGLS**	-0.30	0.81	4.76E-04	3.02E-03
**SLC25A42**	-0.50	0.71	2.71E-03	1.73E-02	**CLCN7**	-0.30	0.81	4.77E-04	3.02E-03
**CNP**	-0.22	0.86	2.71E-03	1.73E-02	**PACS2**	-0.31	0.81	4.80E-04	3.04E-03
**ZBTB47**	-0.43	0.74	2.74E-03	1.75E-02	**ZNF100**	0.53	1.45	4.82E-04	3.05E-03
**ZDHHC4**	-0.37	0.77	2.74E-03	1.75E-02	**PI3**	-0.34	0.79	4.82E-04	3.05E-03
**NIPAL1**	0.26	1.20	2.74E-03	1.75E-02	**WFDC5**	-0.56	0.68	4.84E-04	3.06E-03
**RNF44**	0.31	1.24	2.75E-03	1.75E-02	**VASN**	0.76	1.69	4.85E-04	3.07E-03
**ZNF107**	0.47	1.38	2.76E-03	1.76E-02	**WNK1**	-0.25	0.84	4.86E-04	3.07E-03
**COL17A1**	-0.17	0.89	2.77E-03	1.76E-02	**FANCI**	0.35	1.27	4.87E-04	3.08E-03
**NOP2**	0.23	1.17	2.77E-03	1.76E-02	**SRRM2**	-0.23	0.85	4.88E-04	3.08E-03
**ANAPC1**	0.25	1.19	2.77E-03	1.76E-02	**VAPB**	0.28	1.21	4.90E-04	3.09E-03
**PPP1CC**	0.23	1.17	2.77E-03	1.76E-02	**TBP**	0.39	1.31	4.90E-04	3.09E-03
**GGA2**	0.23	1.17	2.77E-03	1.76E-02	**RAET1E**	-0.41	0.75	4.90E-04	3.09E-03
**RHOB**	-0.33	0.80	2.78E-03	1.76E-02	**NFE2L3**	0.45	1.37	4.92E-04	3.10E-03
**CST6**	-0.56	0.68	2.79E-03	1.77E-02	**SMCR8**	-0.37	0.78	4.93E-04	3.11E-03
**SOS1**	0.26	1.19	2.79E-03	1.77E-02	**GEMIN2**	0.43	1.34	4.93E-04	3.11E-03
**RNF20**	-0.25	0.84	2.79E-03	1.77E-02	**CTPS2**	0.39	1.31	4.98E-04	3.13E-03
**NEMP2**	-0.39	0.76	2.79E-03	1.77E-02	**KLK6**	0.52	1.44	5.00E-04	3.14E-03
**EIF1**	0.18	1.13	2.79E-03	1.77E-02	**GMIP**	-0.40	0.76	5.00E-04	3.15E-03
**SYS1**	0.34	1.27	2.79E-03	1.77E-02	**ZBTB7B**	-0.26	0.84	5.02E-04	3.15E-03
**AKAP8**	0.28	1.21	2.80E-03	1.77E-02	**ETNK1**	0.30	1.23	5.02E-04	3.15E-03
**PPFIBP1**	0.22	1.16	2.80E-03	1.77E-02	**ABCF1**	0.26	1.20	5.02E-04	3.15E-03
**TMEM161B**	0.37	1.30	2.80E-03	1.77E-02	**ATXN1L**	-0.30	0.81	5.03E-04	3.15E-03
**CD274**	0.46	1.38	2.81E-03	1.77E-02	**STRBP**	0.34	1.26	5.05E-04	3.17E-03
**SESN1**	-0.46	0.73	2.81E-03	1.78E-02	**FYTTD1**	0.25	1.19	5.05E-04	3.17E-03
**KLF9**	-0.40	0.76	2.81E-03	1.78E-02	**FBXO2**	-0.31	0.81	5.07E-04	3.18E-03
**TRAK2**	0.30	1.23	2.82E-03	1.78E-02	**DLG3**	0.35	1.28	5.10E-04	3.19E-03
**PCYOX1**	-0.25	0.84	2.83E-03	1.78E-02	**LPAR3**	0.32	1.25	5.10E-04	3.19E-03
**SMAP2**	-0.27	0.83	2.83E-03	1.79E-02	**RBM12**	0.29	1.22	5.11E-04	3.20E-03
**TRAF4**	0.29	1.22	2.84E-03	1.79E-02	**SYTL2**	-0.51	0.70	5.15E-04	3.22E-03
**GNL3**	0.21	1.16	2.85E-03	1.80E-02	**FAM133B**	0.47	1.39	5.16E-04	3.23E-03
**PROB1**	-0.55	0.68	2.85E-03	1.80E-02	**CENPJ**	0.39	1.31	5.22E-04	3.26E-03
**STAU1**	0.19	1.14	2.85E-03	1.80E-02	**FTH1**	0.33	1.26	5.23E-04	3.27E-03
**NSRP1**	0.31	1.24	2.86E-03	1.80E-02	**PRKD3**	0.30	1.23	5.30E-04	3.31E-03
**F12**	-0.48	0.72	2.87E-03	1.81E-02	**SETBP1**	-0.46	0.73	5.33E-04	3.33E-03
**WNT7A**	0.29	1.22	2.87E-03	1.81E-02	**ATP7B**	-0.43	0.74	5.37E-04	3.35E-03
**FAM3C**	0.37	1.29	2.88E-03	1.81E-02	**PNPO**	-0.37	0.77	5.37E-04	3.35E-03
**IL17RA**	-0.29	0.82	2.88E-03	1.81E-02	**RTCA**	0.32	1.25	5.38E-04	3.35E-03
**EIF4G1**	0.18	1.13	2.89E-03	1.81E-02	**PDIA3**	0.25	1.19	5.39E-04	3.36E-03
**BRCA1**	-0.24	0.85	2.89E-03	1.81E-02	**CAMSAP3**	0.29	1.22	5.40E-04	3.36E-03
**CEP85**	0.27	1.21	2.90E-03	1.82E-02	**UBE2J1**	0.29	1.22	5.43E-04	3.38E-03
**TAX1BP1**	0.24	1.18	2.90E-03	1.82E-02	**RASL11B**	0.63	1.55	5.45E-04	3.39E-03
**MT-ATP6**	0.47	1.38	2.91E-03	1.82E-02	**TLR3**	-0.49	0.71	5.45E-04	3.39E-03
**PFKFB3**	0.23	1.17	2.91E-03	1.82E-02	**GEMIN5**	0.32	1.25	5.46E-04	3.39E-03
**REPS1**	0.21	1.16	2.92E-03	1.83E-02	**ESYT1**	0.28	1.21	5.47E-04	3.40E-03
**OTOP3**	-0.35	0.79	2.92E-03	NA	**MIS18BP1**	0.33	1.26	5.47E-04	3.40E-03
**COG5**	0.24	1.18	2.93E-03	1.84E-02	**PVRL4**	-0.32	0.80	5.50E-04	3.41E-03
**FAT2**	-0.24	0.85	2.94E-03	1.84E-02	**PPP6R1**	-0.26	0.83	5.51E-04	3.42E-03
**ACSL1**	-0.24	0.85	2.97E-03	1.86E-02	**DNAJC3**	0.28	1.21	5.54E-04	3.44E-03
**ERCC2**	0.31	1.24	2.97E-03	1.86E-02	**EPRS**	0.24	1.18	5.57E-04	3.45E-03
**PABPN1**	0.25	1.19	2.97E-03	1.86E-02	**ZNF266**	-0.34	0.79	5.59E-04	3.46E-03
**SPTBN1**	0.18	1.13	2.98E-03	1.86E-02	**CUL2**	0.28	1.21	5.61E-04	3.47E-03
**ABCF1**	0.21	1.15	2.98E-03	1.86E-02	**PPP2R5D**	-0.28	0.82	5.63E-04	3.49E-03
**PADI3**	-0.55	0.68	3.00E-03	1.87E-02	**L1TD1**	0.46	1.38	5.65E-04	3.50E-03
**ACER1**	-0.55	0.68	3.00E-03	1.87E-02	**BTG2**	-0.23	0.85	5.67E-04	3.51E-03
**TMEM87A**	0.26	1.20	3.01E-03	1.88E-02	**PAQR3**	0.36	1.28	5.67E-04	3.51E-03
**CERCAM**	-0.28	0.83	3.02E-03	1.88E-02	**PEX6**	-0.28	0.82	5.73E-04	3.54E-03
**ECT2**	0.26	1.20	3.03E-03	1.89E-02	**FAM72D**	0.74	1.67	5.78E-04	3.57E-03
**SLC25A36**	0.31	1.24	3.03E-03	1.89E-02	**KRT17**	-0.25	0.84	5.78E-04	3.57E-03
**CCDC51**	-0.29	0.82	3.05E-03	1.90E-02	**DGKA**	-0.24	0.85	5.78E-04	3.57E-03
**PRKCI**	0.25	1.19	3.07E-03	1.91E-02	**ERMP1**	0.24	1.18	5.80E-04	3.57E-03
**ATP2C2**	-0.39	0.76	3.08E-03	1.91E-02	**CKB**	0.58	1.50	5.80E-04	3.58E-03
**TMEM45B**	-0.27	0.83	3.08E-03	1.91E-02	**TAF9B**	0.35	1.27	5.80E-04	3.58E-03
**EMP3**	-0.25	0.84	3.08E-03	1.91E-02	**ZNF257**	0.75	1.68	5.83E-04	3.59E-03
**CLK2**	-0.31	0.81	3.09E-03	1.92E-02	**YIPF3**	-0.28	0.83	5.87E-04	3.61E-03
**CLEC2B**	-0.55	0.68	3.10E-03	1.92E-02	**C9orf142**	-0.37	0.78	5.88E-04	3.62E-03
**AGAP6**	0.53	1.44	3.10E-03	1.92E-02	**OBSCN**	0.33	1.26	5.88E-04	3.62E-03
**OVOL1**	-0.33	0.80	3.11E-03	1.93E-02	**EIF2AK3**	-0.38	0.77	5.89E-04	3.62E-03
**NCBP2**	0.23	1.17	3.11E-03	1.93E-02	**BTBD3**	0.34	1.26	5.90E-04	3.62E-03
**NAPSA**	-0.39	0.76	3.13E-03	NA	**DDX52**	0.32	1.25	5.91E-04	3.63E-03
**CLTA**	-0.20	0.87	3.14E-03	1.95E-02	**GDF15**	-1.12	0.46	5.91E-04	3.63E-03
**TM9SF3**	0.23	1.17	3.14E-03	1.95E-02	**IER3**	0.26	1.20	5.95E-04	3.65E-03
**GPR87**	0.28	1.21	3.16E-03	1.95E-02	**SLC12A4**	-0.31	0.81	5.97E-04	3.66E-03
**PAM**	0.21	1.16	3.16E-03	1.95E-02	**TRA2A**	0.27	1.21	5.98E-04	3.67E-03
**SNN**	-0.26	0.84	3.17E-03	1.95E-02	**RBBP5**	0.33	1.26	6.01E-04	3.68E-03
**CLIP1**	0.20	1.14	3.17E-03	1.95E-02	**CUL4A**	-0.27	0.83	6.03E-04	3.69E-03
**FNBP4**	0.24	1.18	3.17E-03	1.95E-02	**KLHL21**	-0.26	0.83	6.05E-04	3.71E-03
**RASL11B**	-0.55	0.68	3.17E-03	1.95E-02	**PSMB8**	0.30	1.23	6.06E-04	3.71E-03
**LIMK1**	0.24	1.18	3.17E-03	1.95E-02	**ZFYVE16**	0.28	1.21	6.06E-04	3.71E-03
**EFS**	-0.30	0.81	3.17E-03	1.95E-02	**PARPBP**	0.48	1.40	6.08E-04	3.72E-03
**CDKN1C**	-0.46	0.73	3.17E-03	1.95E-02	**ZBTB22**	-0.40	0.76	6.08E-04	3.72E-03
**SRFBP1**	0.35	1.28	3.17E-03	1.95E-02	**PSMA4**	0.24	1.18	6.09E-04	3.72E-03
**FAM180A**	-0.53	0.69	3.17E-03	1.95E-02	**LY75**	0.87	1.83	6.09E-04	3.72E-03
**PODNL1**	-0.55	0.68	3.18E-03	1.96E-02	**HIGD1A**	-0.28	0.83	6.09E-04	3.72E-03
**ZNF106**	0.23	1.17	3.18E-03	1.96E-02	**ZCCHC6**	-0.29	0.82	6.17E-04	3.76E-03
**POLQ**	0.29	1.22	3.20E-03	1.97E-02	**CLTB**	-0.22	0.86	6.17E-04	3.76E-03
**MAD2L1**	0.26	1.20	3.22E-03	1.98E-02	**SESN3**	0.27	1.21	6.20E-04	3.78E-03
**MARS2**	-0.36	0.78	3.22E-03	1.98E-02	**ARHGEF9**	-0.39	0.76	6.21E-04	3.78E-03
**SACM1L**	0.30	1.23	3.23E-03	1.98E-02	**ADIRF-AS1**	0.51	1.42	6.21E-04	3.78E-03
**CLCN4**	-0.46	0.73	3.23E-03	NA	**OBSL1**	-0.31	0.81	6.22E-04	3.78E-03

**ZC3HAV1**	-0.23	0.85	3.24E-03	1.99E-02
**TLCD2**	-0.51	0.70	3.24E-03	1.99E-02
**CCNJ**	0.39	1.31	3.24E-03	1.99E-02
**IQCB1**	0.40	1.32	3.25E-03	1.99E-02
**LRP3**	-0.44	0.74	3.25E-03	1.99E-02
**RP11-418J17.1**	0.45	1.37	3.26E-03	2.00E-02
**FARP2**	0.22	1.16	3.27E-03	2.00E-02
**DYRK2**	0.29	1.22	3.27E-03	2.00E-02
**MAPKAPK2**	0.22	1.16	3.27E-03	2.00E-02
**CDK5RAP1**	0.27	1.21	3.27E-03	2.00E-02
**NBR1**	0.21	1.16	3.27E-03	2.00E-02
**PPP4R3B**	0.22	1.16	3.28E-03	2.00E-02
**MVB12A**	-0.31	0.81	3.28E-03	2.00E-02
**AADAT**	-0.51	0.70	3.28E-03	2.00E-02
**LYPLA1**	0.27	1.21	3.28E-03	2.00E-02
**N4BP2L2**	0.30	1.23	3.28E-03	2.00E-02
**RNF19A**	0.30	1.23	3.29E-03	2.00E-02
**C1orf43**	0.19	1.14	3.29E-03	2.00E-02
**GLI1**	-0.44	0.74	3.31E-03	NA
**FAM212B**	-0.31	0.80	3.31E-03	2.01E-02
**LTN1**	0.27	1.21	3.32E-03	2.02E-02
**GOLGA4**	0.26	1.20	3.32E-03	2.02E-02
**TMEM245**	0.22	1.16	3.33E-03	2.03E-02
**PLEKHN1**	-0.29	0.82	3.36E-03	2.04E-02
**FLNA**	0.20	1.15	3.37E-03	2.05E-02
**GBP1**	-0.44	0.74	3.37E-03	2.05E-02
**FTSJ3**	0.20	1.15	3.39E-03	2.06E-02
**SMC4**	0.26	1.20	3.40E-03	2.06E-02
**RP11-832N8.1**	-0.47	0.72	3.40E-03	2.06E-02
**ZXDC**	0.31	1.24	3.44E-03	2.09E-02
**RPS13P2**	-0.45	0.73	3.44E-03	2.09E-02
**MRPL10**	0.27	1.21	3.45E-03	2.09E-02
**BSDC1**	0.24	1.18	3.46E-03	2.09E-02
**MAFG-AS1**	-0.54	0.69	3.47E-03	2.10E-02
**AGO2**	0.24	1.18	3.48E-03	2.10E-02
**TARDBP**	0.23	1.17	3.48E-03	2.11E-02
**ZNF311**	-0.53	0.69	3.49E-03	2.11E-02
**CLDN12**	0.30	1.23	3.49E-03	2.11E-02
**FAM120B**	0.25	1.19	3.49E-03	2.11E-02
**EIF2AK4**	0.21	1.15	3.49E-03	2.11E-02
**DIS3L2**	0.27	1.21	3.50E-03	2.11E-02
**CDCA8**	0.23	1.17	3.50E-03	2.11E-02
**HSPA2**	-0.33	0.80	3.53E-03	2.13E-02
**MAPKBP1**	0.23	1.17	3.53E-03	2.13E-02
**DAPP1**	0.25	1.19	3.54E-03	2.13E-02
**SGK223**	-0.31	0.80	3.55E-03	2.13E-02
**MICB**	-0.50	0.71	3.55E-03	2.14E-02
**WSB1**	0.26	1.20	3.56E-03	2.14E-02
**DHX33**	0.23	1.17	3.57E-03	2.14E-02
**KRT77**	-0.46	0.73	3.57E-03	2.14E-02
**RP11-832A4.7**	0.53	1.45	3.57E-03	2.14E-02
**IL36G**	-0.52	0.69	3.57E-03	2.14E-02
**NUP50-AS1**	0.40	1.32	3.57E-03	2.14E-02
**GAPVD1**	0.22	1.17	3.57E-03	2.14E-02
**DNAJC5**	0.20	1.15	3.58E-03	2.15E-02
**ZNF441**	-0.54	0.69	3.59E-03	2.15E-02
**MEPCE**	-0.26	0.83	3.59E-03	2.15E-02
**VTI1A**	0.26	1.20	3.60E-03	2.15E-02
**CSPP1**	0.36	1.28	3.62E-03	2.17E-02
**DNPH1**	-0.32	0.80	3.62E-03	2.17E-02
**KLK11**	-0.21	0.87	3.63E-03	2.17E-02
**SPTAN1**	0.17	1.12	3.63E-03	2.17E-02
**CLINT1**	0.19	1.14	3.63E-03	2.17E-02
**IRX2**	0.26	1.20	3.66E-03	2.19E-02
**LRRC14**	-0.37	0.77	3.66E-03	2.19E-02
**TPGS1**	-0.54	0.69	3.69E-03	2.20E-02
**SLC35C1**	-0.26	0.84	3.70E-03	2.21E-02
**HOXA1**	0.34	1.26	3.70E-03	2.21E-02
**RASSF9**	-0.47	0.72	3.70E-03	2.21E-02
**POFUT2**	0.34	1.27	3.71E-03	2.21E-02
**ARFGEF1**	0.24	1.18	3.71E-03	2.21E-02
**WLS**	-0.22	0.86	3.73E-03	2.22E-02
**CHD3**	-0.19	0.87	3.74E-03	2.23E-02
**PRKDC**	-0.21	0.86	3.75E-03	2.23E-02
**UBAC2**	0.22	1.17	3.76E-03	2.23E-02
**MINA**	0.24	1.18	3.77E-03	2.24E-02
**BTBD1**	0.24	1.18	3.77E-03	2.24E-02
**BTAF1**	0.27	1.20	3.78E-03	2.24E-02
**CAMK2G**	0.26	1.20	3.79E-03	2.25E-02
**SCAF1**	0.24	1.18	3.79E-03	2.25E-02
**SCLT1**	0.32	1.25	3.80E-03	2.25E-02
**EML4**	0.25	1.19	3.81E-03	2.26E-02
**DNASE2**	-0.30	0.81	3.81E-03	2.26E-02
**CYP26B1**	-0.52	0.70	3.82E-03	2.26E-02
**KDM5C**	0.23	1.17	3.82E-03	2.26E-02
**GPT2**	-0.26	0.84	3.84E-03	2.27E-02
**SLC38A2**	-0.21	0.86	3.84E-03	2.27E-02
**CNOT4**	0.28	1.22	3.85E-03	2.27E-02
**PIDD1**	-0.38	0.77	3.86E-03	2.28E-02
**SIN3B**	0.21	1.15	3.86E-03	2.28E-02
**DPM3**	-0.61	0.65	6.22E-04	3.79E-03
**TMEM159**	-0.31	0.81	6.24E-04	3.79E-03
**JDP2**	0.37	1.29	6.24E-04	3.79E-03
**XPR1**	0.29	1.22	6.27E-04	3.81E-03
**PLD1**	-0.33	0.79	6.27E-04	3.81E-03
**CENPL**	0.46	1.38	6.30E-04	3.83E-03
**VPS37B**	0.30	1.24	6.35E-04	3.85E-03
**APAF1**	0.36	1.28	6.37E-04	3.86E-03
**NPAS2**	-0.37	0.77	6.41E-04	3.88E-03
**NUP88**	0.28	1.21	6.42E-04	3.89E-03
**ARAP3**	0.43	1.34	6.42E-04	3.89E-03
**KLHL8**	0.36	1.28	6.42E-04	3.89E-03
**TUBGCP2**	0.26	1.20	6.43E-04	3.89E-03
**PPP2R2D**	-0.33	0.80	6.43E-04	3.89E-03
**AFAP1L1**	0.33	1.25	6.47E-04	3.91E-03
**AHSA1**	0.27	1.20	6.48E-04	3.92E-03
**IL22RA1**	0.36	1.29	6.50E-04	3.93E-03
**USP7**	0.24	1.18	6.60E-04	3.99E-03
**RAD54L2**	-0.34	0.79	6.66E-04	4.02E-03
**SLC38A9**	0.37	1.29	6.66E-04	4.02E-03
**CEP131**	0.37	1.30	6.66E-04	4.02E-03
**PPP4R2**	0.28	1.21	6.68E-04	4.03E-03
**LDHD**	-1.07	0.48	6.75E-04	4.07E-03
**SMCHD1**	0.28	1.22	6.76E-04	4.07E-03
**FAM72B**	0.67	1.59	6.78E-04	4.08E-03
**RRP7A**	0.26	1.20	6.80E-04	4.09E-03
**CFAP97**	0.34	1.26	6.91E-04	4.16E-03
**PRKCDBP**	0.35	1.27	6.92E-04	4.16E-03
**NRADDP**	-1.25	0.42	6.93E-04	4.17E-03
**ARL4D**	0.29	1.23	6.94E-04	4.17E-03
**PLAU**	0.33	1.25	6.98E-04	4.19E-03
**TRIM37**	0.30	1.23	6.98E-04	4.19E-03
**DDX21**	0.26	1.20	7.04E-04	4.22E-03
**METTL21A**	0.47	1.39	7.05E-04	4.23E-03
**TCF7L1**	-0.39	0.76	7.06E-04	4.23E-03
**UBC**	-0.22	0.86	7.10E-04	4.26E-03
**SH3BP2**	0.37	1.30	7.11E-04	4.26E-03
**FOXC1**	-0.33	0.80	7.18E-04	4.30E-03
**USP22**	-0.24	0.85	7.20E-04	4.31E-03
**FBXO18**	-0.27	0.83	7.23E-04	4.33E-03
**PPARD**	-0.26	0.83	7.27E-04	4.34E-03
**FYCO1**	0.25	1.19	7.28E-04	4.35E-03
**AMZ2**	-0.29	0.82	7.30E-04	4.35E-03
**EVC**	-0.28	0.82	7.30E-04	4.35E-03
**PRAF2**	-0.87	0.55	7.30E-04	4.35E-03
**PIP4K2A**	0.37	1.29	7.32E-04	4.36E-03
**LY6G6C**	-0.77	0.59	7.35E-04	4.38E-03
**NKIRAS1**	0.52	1.44	7.43E-04	4.42E-03
**LZTS2**	0.27	1.20	7.45E-04	4.44E-03
**POLD4**	-0.61	0.66	7.47E-04	4.44E-03
**MPRIP**	-0.22	0.86	7.48E-04	4.45E-03
**MPHOSPH9**	0.39	1.31	7.51E-04	4.47E-03
**EPN3**	0.28	1.22	7.54E-04	4.48E-03
**ARHGEF37**	-0.28	0.83	7.54E-04	4.48E-03
**TBL1X**	0.40	1.32	7.56E-04	4.49E-03
**PCDHGA12**	-0.99	0.50	7.59E-04	4.51E-03
**MITF**	-0.50	0.71	7.64E-04	4.53E-03
**HRAS**	-0.29	0.82	7.64E-04	4.53E-03
**PLEKHF1**	-0.65	0.64	7.66E-04	4.54E-03
**EIF2S2**	0.24	1.18	7.68E-04	4.55E-03
**CNN3**	0.27	1.21	7.79E-04	4.61E-03
**TSPAN13**	0.32	1.25	7.80E-04	4.62E-03
**FBXW5**	-0.26	0.84	7.80E-04	4.62E-03
**ZDHHC20**	0.25	1.19	7.84E-04	4.64E-03
**APOL1**	0.45	1.37	7.92E-04	4.68E-03
**RBMS1**	-0.25	0.84	7.93E-04	4.68E-03
**RANBP6**	0.29	1.23	7.97E-04	4.71E-03
**C2orf69**	0.36	1.28	8.00E-04	4.73E-03
**C5orf42**	-0.31	0.81	8.02E-04	4.73E-03
**RAB15**	-0.47	0.72	8.05E-04	4.75E-03
**CH17-360D5.3**	-0.27	0.83	8.15E-04	4.80E-03
**S100A16**	-0.21	0.86	8.15E-04	4.81E-03
**HOTAIRM1**	-0.52	0.70	8.16E-04	4.81E-03
**CNOT6**	0.26	1.20	8.16E-04	4.81E-03
**ZNF862**	-0.37	0.77	8.16E-04	4.81E-03
**ITGB6**	0.26	1.20	8.24E-04	4.85E-03
**RAP2C**	0.32	1.25	8.26E-04	4.86E-03
**CRIP2**	-0.67	0.63	8.29E-04	4.88E-03
**DNAJB12**	0.30	1.23	8.35E-04	4.91E-03
**IMMT**	0.24	1.18	8.38E-04	4.92E-03
**HNRNPH1**	0.21	1.16	8.40E-04	4.93E-03
**GLI1**	1.27	2.40	8.41E-04	4.94E-03
**RABEP1**	0.27	1.20	8.42E-04	4.94E-03
**MTFR2**	0.55	1.47	8.46E-04	4.96E-03
**NRD1**	0.22	1.17	8.46E-04	4.96E-03
**IRS2**	-0.59	0.66	8.48E-04	4.97E-03
**HNRNPA1P33**	-0.33	0.79	8.61E-04	5.05E-03
**MRPL19**	0.27	1.20	8.64E-04	5.06E-03
**FMNL2**	0.26	1.19	8.68E-04	5.08E-03
**REEP6**	-0.53	0.69	8.69E-04	5.08E-03

PPDPF	-0.28	0.83	3.86E-03	2.28E-02	TMED7	0.28	1.21	8.70E-04	5.09E-03
UBR5	0.20	1.15	3.87E-03	2.28E-02	DHRS3	0.30	1.23	8.71E-04	5.09E-03
UFM1	0.26	1.20	3.87E-03	2.28E-02	YY1	0.23	1.18	8.73E-04	5.10E-03
SLC1A6	-0.54	0.69	3.88E-03	2.29E-02	PIK3CB	0.27	1.21	8.74E-04	5.11E-03
PTPRG-AS1	-0.54	0.69	3.90E-03	2.30E-02	NQO1	0.26	1.20	8.76E-04	5.11E-03
AAR2	0.26	1.20	3.90E-03	2.30E-02	SIRT3	-0.43	0.74	8.77E-04	5.12E-03
FBXO28	0.28	1.22	3.91E-03	2.30E-02	STRADA	-0.89	0.54	8.86E-04	5.17E-03
SCAPER	0.35	1.27	3.91E-03	2.30E-02	RP11-541N10.3	-1.06	0.48	8.86E-04	5.17E-03
GABARAPL2	0.29	1.23	3.92E-03	2.30E-02	HNRNPK	0.21	1.16	8.87E-04	5.17E-03
MID1	0.29	1.22	3.92E-03	2.30E-02	ZBED6	-0.43	0.74	8.90E-04	5.18E-03
CYP27C1	-0.40	0.76	3.92E-03	2.31E-02	ZNF750	-0.38	0.77	8.91E-04	5.19E-03
CAPRIN2	0.35	1.28	3.93E-03	2.31E-02	CEBPZ	0.28	1.21	8.93E-04	5.20E-03
VPS25	-0.25	0.84	3.93E-03	2.31E-02	STRAP	0.23	1.17	9.00E-04	5.23E-03
ZNF619	-0.47	0.72	3.93E-03	2.31E-02	KIF5B	0.22	1.16	9.00E-04	5.23E-03
MT-ND5	0.45	1.37	3.94E-03	2.31E-02	TMEM50A	-0.26	0.84	9.03E-04	5.25E-03
MTDH	0.19	1.14	3.94E-03	2.31E-02	PARP2	0.34	1.27	9.08E-04	5.27E-03
PSD3	0.26	1.20	3.95E-03	2.31E-02	KLF8	-0.35	0.79	9.09E-04	5.28E-03
TUBGCP6	0.28	1.21	3.96E-03	2.32E-02	ANKRD33B	-0.46	0.72	9.10E-04	5.28E-03
NPIPB15	0.52	1.43	3.97E-03	2.32E-02	H2AFJ	-0.33	0.79	9.13E-04	5.30E-03
ZNF319	-0.43	0.74	3.97E-03	2.33E-02	PHIP	0.26	1.20	9.19E-04	5.33E-03
ZNF792	-0.52	0.70	3.97E-03	2.33E-02	RTN4IP1	0.41	1.33	9.20E-04	5.33E-03
GARS	-0.17	0.89	3.98E-03	2.33E-02	POM121	-0.40	0.76	9.21E-04	5.34E-03
DENND1A	0.28	1.22	3.98E-03	2.33E-02	GID4	-0.34	0.79	9.22E-04	5.34E-03
DDX31	0.28	1.21	3.98E-03	2.33E-02	VPS9D1	-0.50	0.71	9.23E-04	5.34E-03
CCNL2	0.25	1.19	3.99E-03	2.33E-02	HMGB1P5	0.50	1.41	9.23E-04	5.34E-03
DSN1	-0.32	0.80	4.00E-03	2.33E-02	AIMP1	0.28	1.21	9.23E-04	5.34E-03
CYP4F12	-0.46	0.73	4.00E-03	2.33E-02	GPSM1	-0.40	0.76	9.25E-04	5.35E-03
ELOF1	0.26	1.20	4.00E-03	2.33E-02	CCDC64	-0.37	0.78	9.26E-04	5.35E-03
PRSS12	-0.27	0.83	4.01E-03	2.34E-02	MLH1	0.29	1.22	9.30E-04	5.37E-03
ZNF668	-0.49	0.71	4.01E-03	2.34E-02	XBP1	0.27	1.20	9.30E-04	5.37E-03
TMEM223	-0.43	0.74	4.01E-03	2.34E-02	FAM83C	-0.33	0.80	9.31E-04	5.37E-03
ZMIZ2	0.26	1.19	4.02E-03	2.34E-02	LDOC1L	-0.36	0.78	9.38E-04	5.41E-03
VPS37C	0.28	1.21	4.03E-03	2.34E-02	TINF2	-0.28	0.82	9.40E-04	5.42E-03
CHDH	-0.33	0.80	4.03E-03	NA	AKAP12	0.70	1.63	9.40E-04	5.42E-03
DPP8	0.26	1.20	4.04E-03	2.35E-02	NLGN4Y	-0.37	0.78	9.41E-04	5.42E-03
PNKP	0.29	1.22	4.04E-03	2.35E-02	GAR1	0.35	1.28	9.44E-04	5.44E-03
PTEN	0.25	1.19	4.04E-03	2.35E-02	XPC	-0.31	0.81	9.46E-04	5.45E-03
SDHC	-0.23	0.85	4.07E-03	2.37E-02	ZNF680	0.45	1.37	9.53E-04	5.48E-03
WDR75	0.24	1.18	4.07E-03	2.37E-02	XKR8	0.47	1.38	9.56E-04	5.50E-03
ASTE1	-0.46	0.73	4.08E-03	2.37E-02	MYH14	-0.33	0.80	9.58E-04	5.51E-03
OAZ1	0.18	1.14	4.10E-03	2.38E-02	DIS3L	0.36	1.28	9.60E-04	5.52E-03
CD46	0.25	1.19	4.11E-03	2.39E-02	TENM3	0.32	1.25	9.62E-04	5.52E-03
GNA12	0.25	1.19	4.11E-03	2.39E-02	RYR1	0.47	1.39	9.64E-04	5.54E-03
DLC1	0.51	1.43	4.12E-03	2.39E-02	LTB4R	-0.33	0.79	9.69E-04	5.56E-03
RPL27A	-0.16	0.90	4.12E-03	2.39E-02	MXD1	-0.33	0.79	9.72E-04	5.58E-03
PI3	-0.26	0.83	4.13E-03	2.39E-02	MORF4L1P1	0.53	1.44	9.74E-04	5.59E-03
MED1	0.20	1.15	4.13E-03	2.39E-02	RAPGEF5	0.36	1.29	9.76E-04	5.59E-03
PSMA3-AS1	0.42	1.34	4.16E-03	2.41E-02	BLNK	-0.52	0.70	9.77E-04	5.60E-03
SEC14L1	0.22	1.16	4.17E-03	2.41E-02	GAPVD1	0.24	1.18	9.77E-04	5.60E-03
NAPRT	-0.27	0.83	4.18E-03	2.42E-02	VPS8	-0.30	0.81	9.79E-04	5.60E-03
TIGD2	0.36	1.28	4.18E-03	2.42E-02	POLD3	0.33	1.26	9.83E-04	5.63E-03
CEP162	-0.46	0.73	4.18E-03	2.42E-02	PARP9	-0.26	0.83	9.84E-04	5.63E-03
CD2AP	0.23	1.17	4.20E-03	2.42E-02	TMEM135	0.37	1.29	9.88E-04	5.64E-03
RCOR2	-0.53	0.69	4.23E-03	2.44E-02	NPM1	0.24	1.18	9.88E-04	5.64E-03
EIF4EBP1	-0.26	0.84	4.23E-03	2.44E-02	RAB12	0.33	1.26	9.90E-04	5.66E-03
CASP1	-0.32	0.80	4.23E-03	2.44E-02	MGAM	-1.40	0.38	9.94E-04	5.68E-03
RPL22	-0.24	0.84	4.24E-03	2.45E-02	TULP4	-0.35	0.79	9.95E-04	5.68E-03
STAT3	0.20	1.15	4.24E-03	2.45E-02	KRT80	-0.25	0.84	9.96E-04	5.68E-03
SOX15	-0.25	0.84	4.25E-03	2.45E-02	TUBGCP6	-0.26	0.83	9.97E-04	5.68E-03
AGAP1	0.26	1.20	4.27E-03	2.46E-02	NKPD1	0.49	1.40	1.00E-03	5.71E-03
CREB1	-0.26	0.84	4.28E-03	2.46E-02	LMLN	0.40	1.32	1.00E-03	5.72E-03
TFAP4	-0.33	0.79	4.28E-03	2.46E-02	CCNF	0.38	1.30	1.01E-03	5.73E-03
LIMK2	0.18	1.13	4.36E-03	2.51E-02	SUN1	0.23	1.17	1.01E-03	5.74E-03
ATP10B	-0.51	0.70	4.37E-03	2.51E-02	G0S2	0.64	1.55	1.01E-03	5.76E-03
TRAFD1	0.24	1.18	4.37E-03	2.51E-02	PHYKPL	-0.36	0.78	1.01E-03	5.77E-03
TLN2	-0.33	0.80	4.38E-03	2.52E-02	PLIN4	-0.67	0.63	1.02E-03	5.78E-03
DHX29	0.22	1.16	4.38E-03	2.52E-02	STK38	0.25	1.19	1.02E-03	5.78E-03
VRK1	0.30	1.23	4.41E-03	2.53E-02	POC1A	0.39	1.31	1.02E-03	5.80E-03
TUBB2A	0.28	1.22	4.41E-03	2.53E-02	SLC25A27	-0.56	0.68	1.02E-03	5.81E-03
TPR	0.19	1.14	4.41E-03	2.53E-02	ADAMTS4	1.62	3.08	1.02E-03	5.81E-03
TGIF1	0.25	1.19	4.44E-03	2.54E-02	NOTCH3	-0.24	0.85	1.02E-03	5.81E-03
VAMP2	-0.34	0.79	4.44E-03	2.55E-02	CORO1B	-0.24	0.85	1.02E-03	5.81E-03
SCAND1	-0.37	0.77	4.46E-03	2.55E-02	NDFIP1	0.24	1.18	1.03E-03	5.82E-03
NOTCH3	-0.20	0.87	4.46E-03	2.55E-02	FAM219B	-0.39	0.76	1.03E-03	5.82E-03
TNFAIP8L1	-0.32	0.80	4.47E-03	2.56E-02	PLCD1	-0.26	0.83	1.03E-03	5.83E-03
EREG	-0.41	0.75	4.47E-03	2.56E-02	PYGL	-0.22	0.86	1.03E-03	5.83E-03
PXDC1	0.29	1.23	4.47E-03	2.56E-02	TBC1D8B	-0.39	0.76	1.03E-03	5.83E-03
ING5	0.33	1.26	4.48E-03	2.56E-02	FXYD5	0.28	1.21	1.03E-03	5.83E-03
SPARC	-0.22	0.86	4.50E-03	2.57E-02	SNX5	0.25	1.19	1.03E-03	5.83E-03
TRAPPC3	0.20	1.15	4.51E-03	2.57E-02	LINC00094	-0.40	0.76	1.04E-03	5.85E-03
AC016735.2	-0.50	0.71	4.51E-03	2.58E-02	KIAA1958	-0.51	0.70	1.04E-03	5.85E-03
ACP1	0.21	1.16	4.53E-03	2.58E-02	FDPS	-0.23	0.86	1.04E-03	5.85E-03
EZR	0.15	1.11	4.55E-03	2.59E-02	PCGF3	-0.26	0.84	1.04E-03	5.86E-03
ALCAM	0.21	1.16	4.56E-03	2.60E-02	GTF3C4	0.26	1.20	1.04E-03	5.86E-03
SYT7	-0.53	0.69	4.58E-03	2.61E-02	FTSJ2	-0.38	0.77	1.04E-03	5.86E-03
ARHGAP26	0.36	1.28	4.58E-03	2.61E-02	MTMR2	0.27	1.20	1.04E-03	5.86E-03
MAPK8	0.31	1.24	4.58E-03	2.61E-02	EWSR1	0.21	1.16	1.04E-03	5.88E-03
GGA1	0.25	1.19	4.60E-03	2.62E-02	PSTPIP1	0.74	1.67	1.04E-03	5.88E-03
VPS16	0.31	1.24	4.60E-03	2.62E-02	NOP10	-0.27	0.83	1.05E-03	5.89E-03
SYNE1	-0.53	0.69	4.60E-03	2.62E-02	ZNF24	0.25	1.19	1.05E-03	5.89E-03

**AP1S1**	-0.20	0.87	4.61E-03	2.62E-02
**NBAS**	0.23	1.17	4.61E-03	2.62E-02
**CSGALNACT2**	0.33	1.26	4.61E-03	2.62E-02
**TMEM9**	-0.27	0.83	4.62E-03	2.62E-02
**FAM13B**	0.36	1.29	4.62E-03	2.62E-02
**LAMB2**	-0.25	0.84	4.62E-03	2.62E-02
**LRRFIP1**	0.19	1.14	4.64E-03	2.63E-02
**DNAJC6**	0.51	1.43	4.64E-03	2.63E-02
**SPPL2B**	0.33	1.26	4.64E-03	2.63E-02
**ZC3H11A**	0.22	1.17	4.64E-03	2.63E-02
**APLN**	-0.33	0.79	4.65E-03	2.63E-02
**RILPL1**	0.35	1.27	4.65E-03	2.63E-02
**TMF1**	0.25	1.19	4.66E-03	2.63E-02
**SEC24B**	0.22	1.16	4.66E-03	2.64E-02
**IGF1R**	0.22	1.17	4.68E-03	2.64E-02
**LTB4R2**	-0.48	0.72	4.70E-03	2.66E-02
**PSMD3**	0.20	1.15	4.72E-03	2.67E-02
**NOC3L**	0.26	1.20	4.74E-03	2.68E-02
**TNPO1**	0.23	1.17	4.75E-03	2.68E-02
**KHSRP**	0.17	1.13	4.77E-03	2.69E-02
**SIX4**	0.42	1.33	4.78E-03	2.69E-02
**WDR37**	0.31	1.24	4.78E-03	2.69E-02
**NLRC5**	-0.46	0.73	4.79E-03	2.70E-02
**ZNF512B**	0.24	1.18	4.80E-03	2.70E-02
**SPATA5**	0.38	1.30	4.81E-03	2.70E-02
**TPD52L1**	-0.21	0.86	4.82E-03	2.71E-02
**LCOR**	0.32	1.24	4.83E-03	2.72E-02
**B3GNT4**	-0.53	0.69	4.84E-03	2.72E-02
**CDK7**	0.27	1.21	4.86E-03	2.73E-02
**RND3**	0.23	1.17	4.86E-03	2.73E-02
**SLC16A14**	-0.51	0.70	4.87E-03	2.73E-02
**VIPR1**	-0.52	0.70	4.88E-03	2.74E-02
**GSR**	0.21	1.15	4.88E-03	2.74E-02
**KCTD15**	-0.22	0.86	4.89E-03	2.74E-02
**COX10**	0.28	1.22	4.89E-03	2.74E-02
**NRAV**	-0.34	0.79	4.89E-03	2.74E-02
**UPK3B**	-0.47	0.72	4.92E-03	2.75E-02
**PAPOLA**	0.20	1.15	4.92E-03	2.75E-02
**ITSN2**	0.24	1.18	4.93E-03	2.76E-02
**P2RX7**	-0.48	0.72	4.93E-03	2.76E-02
**ZCCHC3**	-0.31	0.81	4.93E-03	2.76E-02
**CLPTM1**	0.23	1.17	4.95E-03	2.76E-02
**PITX2**	-0.29	0.82	4.95E-03	2.77E-02
**CTBP1**	0.23	1.17	4.96E-03	2.77E-02
**PRDM2**	0.23	1.17	4.96E-03	2.77E-02
**NAPG**	0.27	1.20	4.99E-03	2.78E-02
**TRIM32**	-0.30	0.81	5.00E-03	2.79E-02
**TRIM33**	0.23	1.17	5.02E-03	2.79E-02
**TSC2**	0.23	1.18	5.02E-03	2.79E-02
**CD1D**	-0.52	0.70	5.02E-03	2.79E-02
**SENP5**	0.30	1.23	5.02E-03	2.79E-02
**DBI**	-0.22	0.86	5.02E-03	2.79E-02
**ARHGAP31**	-0.36	0.78	5.04E-03	2.80E-02
**C19orf48**	-0.27	0.83	5.05E-03	2.81E-02
**TFAP2E**	-0.50	0.71	5.07E-03	2.82E-02
**TPPP3**	-0.40	0.76	5.07E-03	NA
**ZNF845**	0.41	1.33	5.08E-03	2.82E-02
**ACY1**	-0.52	0.70	5.11E-03	2.84E-02
**FDFT1**	-0.18	0.88	5.11E-03	2.84E-02
**TLR3**	-0.47	0.72	5.12E-03	2.84E-02
**LMAN1**	0.21	1.15	5.12E-03	2.84E-02
**MIER1**	0.32	1.25	5.12E-03	2.84E-02
**C11orf80**	0.33	1.25	5.12E-03	2.84E-02
**ENY2**	0.27	1.20	5.12E-03	2.84E-02
**MICALCL**	0.29	1.22	5.13E-03	2.84E-02
**TNFSF10**	-0.44	0.73	5.14E-03	2.84E-02
**AASS**	0.40	1.32	5.17E-03	2.86E-02
**TBC1D22A**	0.26	1.20	5.17E-03	2.86E-02
**SHANK3**	-0.35	0.79	5.18E-03	2.86E-02
**HUNK**	-0.48	0.72	5.20E-03	2.87E-02
**MDGA1**	-0.52	0.70	5.20E-03	2.87E-02
**NUFIP2**	0.22	1.17	5.20E-03	2.87E-02
**MFN2**	0.16	1.12	5.20E-03	2.87E-02
**MAN1A2**	0.24	1.18	5.21E-03	2.87E-02
**PTCD2**	-0.34	0.79	5.21E-03	2.87E-02
**CAMSAP3**	0.24	1.18	5.21E-03	2.87E-02
**PDLIM4**	-0.28	0.82	5.21E-03	2.87E-02
**CARD6**	-0.38	0.77	5.21E-03	2.87E-02
**MRPL42**	0.28	1.22	5.22E-03	2.87E-02
**THOC3**	-0.35	0.79	5.24E-03	2.88E-02
**TMEM123**	-0.24	0.85	5.24E-03	2.88E-02
**RNF123**	0.28	1.22	5.25E-03	2.89E-02
**SLC16A6**	-0.51	0.70	5.25E-03	2.89E-02
**SPCS3**	0.24	1.18	5.26E-03	2.89E-02
**SENP2**	0.24	1.18	5.26E-03	2.89E-02
**UBASH3B**	0.23	1.17	5.27E-03	2.89E-02
**RP11-54H7.4**	0.38	1.30	5.29E-03	2.90E-02
**XRN1**	0.28	1.21	5.30E-03	2.91E-02
**C16orf13**	-0.30	0.81	5.31E-03	2.91E-02
**DLL1**	0.38	1.30	5.31E-03	2.91E-02
**ATP6V0B**	0.24	1.18	1.05E-03	5.90E-03
**SATB1**	-0.50	0.71	1.05E-03	5.92E-03
**CLDN7**	0.24	1.18	1.05E-03	5.93E-03
**FANCD2**	0.37	1.29	1.06E-03	5.94E-03
**NOC3L**	0.29	1.22	1.06E-03	5.96E-03
**LL22NC03-86G7.1**	-0.74	0.60	1.06E-03	5.96E-03
**ZNF767P**	-0.41	0.75	1.06E-03	5.97E-03
**CEP85**	0.32	1.25	1.07E-03	5.99E-03
**ATPIF1**	-0.25	0.84	1.07E-03	6.01E-03
**REEP5**	0.23	1.18	1.08E-03	6.06E-03
**GOLGA7B**	-0.72	0.61	1.09E-03	6.08E-03
**MFN2**	0.22	1.16	1.09E-03	6.08E-03
**ATAD2B**	0.34	1.26	1.09E-03	6.10E-03
**NAT6**	-0.48	0.72	1.09E-03	6.10E-03
**ATG5**	0.29	1.22	1.09E-03	6.10E-03
**NALT1**	-0.90	0.54	1.10E-03	6.15E-03
**IL11**	0.67	1.59	1.11E-03	6.18E-03
**PPP6C**	0.26	1.19	1.11E-03	6.18E-03
**CHMP1B**	-0.26	0.84	1.11E-03	6.18E-03
**ZFYVE1**	-0.34	0.79	1.11E-03	6.19E-03
**BARD1**	0.39	1.31	1.11E-03	6.20E-03
**NDRG3**	-0.28	0.82	1.11E-03	6.20E-03
**NDEL1**	-0.27	0.83	1.11E-03	6.20E-03
**RP11-458F8.4**	-0.60	0.66	1.11E-03	6.21E-03
**VHL**	0.28	1.21	1.12E-03	6.23E-03
**SLC11A2**	0.27	1.20	1.12E-03	6.23E-03
**SPCS3**	0.24	1.18	1.12E-03	6.24E-03
**STK4**	-0.28	0.82	1.13E-03	6.28E-03
**CHD8**	-0.23	0.85	1.14E-03	6.33E-03
**DUSP9**	1.45	2.72	1.14E-03	NA
**GCH1**	0.41	1.33	1.14E-03	6.35E-03
**C2orf81**	-0.69	0.62	1.14E-03	6.35E-03
**RHBDL2**	-0.42	0.75	1.15E-03	6.39E-03
**MON2**	-0.25	0.84	1.15E-03	6.39E-03
**MRPS7**	0.25	1.19	1.15E-03	6.40E-03
**COPZ2**	-0.45	0.73	1.15E-03	6.40E-03
**NOC4L**	0.33	1.25	1.16E-03	6.43E-03
**PDLIM1**	-0.22	0.86	1.16E-03	6.44E-03
**MAP3K1**	0.31	1.24	1.16E-03	6.44E-03
**TCAF2**	-1.22	0.43	1.16E-03	6.44E-03
**MROH6**	0.37	1.29	1.16E-03	6.44E-03
**ERI1**	0.37	1.29	1.17E-03	6.45E-03
**LRIG1**	0.49	1.41	1.17E-03	6.46E-03
**ZNF740**	-0.32	0.80	1.17E-03	6.46E-03
**CDK2**	0.28	1.22	1.18E-03	6.50E-03
**NUP54**	0.29	1.22	1.18E-03	6.50E-03
**EIF3D**	0.22	1.16	1.18E-03	6.50E-03
**EIF2D**	-0.27	0.83	1.18E-03	6.50E-03
**ACE2**	-0.98	0.51	1.18E-03	6.50E-03
**PSMD12**	0.25	1.19	1.18E-03	6.50E-03
**MICB**	-0.50	0.71	1.18E-03	6.51E-03
**BIK**	0.65	1.57	1.18E-03	6.51E-03
**CCDC138**	0.49	1.40	1.18E-03	6.51E-03
**PLA2G4A**	-0.32	0.80	1.18E-03	6.51E-03
**KCNJ15**	0.34	1.27	1.18E-03	6.51E-03
**TSPAN3**	0.23	1.18	1.18E-03	6.52E-03
**TKFC**	-0.32	0.80	1.19E-03	6.54E-03
**PMF1**	0.47	1.39	1.20E-03	6.62E-03
**FIS1**	-0.27	0.83	1.21E-03	6.65E-03
**GSE1**	0.25	1.19	1.21E-03	6.66E-03
**SEPHS1**	0.28	1.21	1.21E-03	6.66E-03
**NGEF**	1.17	2.25	1.21E-03	6.67E-03
**ANXA5**	0.25	1.19	1.22E-03	6.69E-03
**SMAD5**	-0.25	0.84	1.23E-03	6.72E-03
**MT-TP**	-0.29	0.82	1.23E-03	6.72E-03
**CARD11**	0.67	1.59	1.23E-03	6.72E-03
**UBP1**	0.24	1.18	1.23E-03	6.74E-03
**ATP1B1**	0.28	1.21	1.23E-03	6.74E-03
**PERP**	-0.23	0.86	1.23E-03	6.74E-03
**CRCT1**	-0.45	0.73	1.24E-03	6.80E-03
**TAF1C**	-0.28	0.82	1.24E-03	6.80E-03
**MIB1**	0.25	1.19	1.25E-03	6.84E-03
**MAPK8**	0.33	1.26	1.25E-03	6.85E-03
**ALDH1A3**	-0.25	0.84	1.25E-03	6.86E-03
**PUF60**	0.22	1.17	1.26E-03	6.86E-03
**SPRED1**	0.34	1.27	1.26E-03	6.87E-03
**MOXD1**	0.51	1.43	1.26E-03	6.87E-03
**FAM126B**	0.30	1.24	1.26E-03	6.87E-03
**FADS1**	0.30	1.23	1.26E-03	6.88E-03
**STX17**	0.34	1.26	1.27E-03	6.90E-03
**COPRS**	0.35	1.27	1.27E-03	6.92E-03
**CORO1C**	0.21	1.16	1.27E-03	6.93E-03
**AP3D1**	0.22	1.17	1.27E-03	6.93E-03
**CAB39**	0.22	1.17	1.27E-03	6.93E-03
**GRIP1**	0.40	1.32	1.28E-03	6.94E-03
**CUL3**	0.25	1.19	1.28E-03	6.95E-03
**LIN54**	0.39	1.31	1.28E-03	6.95E-03
**SEC14L2**	-0.24	0.85	1.28E-03	6.96E-03
**ELF3**	0.39	1.31	1.29E-03	7.00E-03
**CDC42EP1**	-0.25	0.84	1.29E-03	7.00E-03

CD109	-0.21	0.87	5.32E-03	2.91E-02
CHTF8	0.20	1.15	5.33E-03	2.92E-02
GSTO2	-0.26	0.84	5.35E-03	2.93E-02
MT-CO1	0.40	1.32	5.37E-03	2.94E-02
FDXR	-0.27	0.83	5.37E-03	2.94E-02
CUEDC1	0.36	1.28	5.37E-03	2.94E-02
DEGS2	-0.50	0.71	5.38E-03	2.94E-02
GALNT2	0.19	1.14	5.39E-03	2.94E-02
TMEM231	-0.40	0.76	5.39E-03	2.95E-02
FDPS	-0.21	0.86	5.40E-03	2.95E-02
FAM136A	-0.23	0.85	5.41E-03	2.95E-02
EP400	0.21	1.16	5.41E-03	2.95E-02
DAP	0.21	1.15	5.42E-03	2.95E-02
ZFP91	0.20	1.15	5.43E-03	2.96E-02
ARHGEF40	-0.36	0.78	5.44E-03	2.96E-02
TAPBP	-0.20	0.87	5.44E-03	2.96E-02
AKT1	0.21	1.16	5.45E-03	2.97E-02
CHKA	0.46	1.37	5.45E-03	2.97E-02
BRD9	0.25	1.19	5.45E-03	2.97E-02
PRDX2	-0.23	0.85	5.46E-03	2.97E-02
NAV1	-0.18	0.88	5.47E-03	2.97E-02
UBE2L6	-0.32	0.80	5.48E-03	2.98E-02
SULT1E1	-0.45	0.73	5.50E-03	2.99E-02
FAM78A	-0.40	0.76	5.50E-03	NA
PROM2	-0.20	0.87	5.52E-03	3.00E-02
BTN3A2	-0.43	0.74	5.52E-03	3.00E-02
PAQR7	-0.28	0.83	5.53E-03	3.00E-02
FAM118B	0.34	1.26	5.56E-03	3.01E-02
AKR1C1	-0.47	0.72	5.56E-03	3.01E-02
NPAT	-0.31	0.81	5.57E-03	3.02E-02
KCMF1	0.19	1.14	5.58E-03	3.02E-02
ZNHIT2	-0.43	0.74	5.59E-03	3.03E-02
PHC3	0.28	1.21	5.61E-03	3.03E-02
ATXN2	0.24	1.18	5.61E-03	3.03E-02
SCN4B	-0.52	0.70	5.62E-03	3.04E-02
TAF13	0.34	1.26	5.62E-03	3.04E-02
CTSH	-0.29	0.82	5.64E-03	3.04E-02
DPH6	0.42	1.34	5.64E-03	3.04E-02
ARHGEF37	-0.22	0.86	5.64E-03	3.04E-02
COA7	-0.23	0.85	5.64E-03	3.04E-02
RALGDS	-0.23	0.85	5.65E-03	3.04E-02
ALKBH1	0.34	1.26	5.65E-03	3.05E-02
HIPK2	0.22	1.17	5.68E-03	3.06E-02
PLCB2	-0.51	0.70	5.68E-03	3.06E-02
SCNN1B	-0.39	0.76	5.69E-03	3.07E-02
PTGFRN	0.17	1.13	5.71E-03	3.07E-02
PNKD	-0.32	0.80	5.71E-03	3.07E-02
CERS3	-0.23	0.86	5.72E-03	3.08E-02
KCTD13	0.36	1.28	5.73E-03	3.08E-02
ANKRD36BP2	-0.51	0.70	5.76E-03	3.09E-02
BRI3	-0.31	0.81	5.78E-03	3.10E-02
CD14	-0.49	0.71	5.78E-03	3.10E-02
TRIM69	-0.35	0.78	5.79E-03	3.11E-02
ZNF358	-0.43	0.74	5.79E-03	3.11E-02
ARIH2	0.20	1.15	5.80E-03	3.11E-02
PMPCB	0.22	1.17	5.80E-03	3.11E-02
RP11-543P15.1	-0.49	0.71	5.80E-03	3.11E-02
HGS	0.25	1.19	5.80E-03	3.11E-02
CCDC15	-0.41	0.75	5.81E-03	3.11E-02
QTRTD1	0.25	1.19	5.82E-03	3.12E-02
ALKBH4	-0.41	0.75	5.83E-03	3.12E-02
FAM76A	-0.40	0.76	5.83E-03	3.12E-02
XRCC4	0.36	1.28	5.83E-03	3.12E-02
DNAJC2	0.29	1.22	5.83E-03	3.12E-02
TYW3	0.29	1.22	5.84E-03	3.12E-02
ACD	-0.29	0.82	5.84E-03	3.12E-02
AMDHD2	-0.41	0.75	5.85E-03	3.12E-02
TPX2	0.18	1.13	5.85E-03	3.12E-02
IMP4	0.23	1.17	5.86E-03	3.12E-02
NCAPD2	0.16	1.12	5.88E-03	3.14E-02
FAM126A	0.25	1.19	5.88E-03	3.14E-02
TSSC1	0.26	1.20	5.90E-03	3.14E-02
CAPN10-AS1	-0.50	0.71	5.90E-03	3.14E-02
SYNPO	-0.32	0.80	5.91E-03	3.14E-02
PVT1	0.44	1.36	5.91E-03	3.15E-02
DTX4	-0.34	0.79	5.93E-03	3.15E-02
C8orf58	-0.37	0.77	5.94E-03	3.16E-02
SH3D21	-0.32	0.80	5.97E-03	3.17E-02
MXI1	-0.35	0.78	5.98E-03	3.18E-02
BRWD1	0.29	1.22	5.98E-03	3.18E-02
AC004951.5	-0.46	0.73	5.98E-03	3.18E-02
ERP29	-0.19	0.87	5.99E-03	3.18E-02
IFI44	-0.40	0.76	6.00E-03	3.18E-02
HN1L	0.17	1.13	6.01E-03	3.19E-02
RRP15	0.25	1.19	6.02E-03	3.19E-02
GS1-393G12.12	-0.49	0.71	6.04E-03	3.20E-02
IDH1	-0.23	0.85	6.05E-03	3.20E-02
DHRS9	-0.49	0.71	6.05E-03	3.20E-02
SUMO3	-0.18	0.88	6.06E-03	3.21E-02
DPM1	0.25	1.19	6.07E-03	3.21E-02
RABGGTA	-0.27	0.83	1.29E-03	7.00E-03
TROAP	0.38	1.30	1.29E-03	7.00E-03
SLC19A2	0.36	1.28	1.29E-03	7.01E-03
OAS2	-0.22	0.86	1.30E-03	7.04E-03
PHF20	-0.27	0.83	1.31E-03	7.08E-03
UGCG	0.33	1.26	1.31E-03	7.11E-03
SAE1	0.24	1.18	1.32E-03	7.13E-03
DCP1B	-0.46	0.72	1.32E-03	7.15E-03
SUN2	0.24	1.18	1.32E-03	7.15E-03
NDUFAB1	0.27	1.20	1.33E-03	7.20E-03
HAGH	-0.31	0.81	1.33E-03	7.21E-03
PODXL	0.82	1.77	1.34E-03	7.22E-03
POLR3D	-0.31	0.80	1.34E-03	7.23E-03
STAT1	-0.21	0.86	1.34E-03	7.24E-03
PTMS	0.28	1.21	1.35E-03	7.29E-03
UBASH3B	0.41	1.33	1.35E-03	7.30E-03
ARPP19	0.23	1.17	1.36E-03	7.31E-03
CERCAM	-0.28	0.82	1.36E-03	7.32E-03
ANGPTL4	-0.61	0.66	1.36E-03	7.32E-03
EZH1	-0.35	0.78	1.36E-03	7.32E-03
WDR82	0.23	1.17	1.37E-03	7.36E-03
TRIM32	-0.31	0.81	1.38E-03	7.42E-03
KIF1C	0.21	1.16	1.38E-03	7.44E-03
NAV2	-0.23	0.85	1.38E-03	7.44E-03
SLC30A1	0.25	1.19	1.39E-03	7.45E-03
TFAP2C	0.30	1.23	1.39E-03	7.48E-03
KCNIP3	0.83	1.77	1.40E-03	7.49E-03
MNT	-0.33	0.79	1.40E-03	7.50E-03
C22orf29	-0.26	0.83	1.40E-03	7.50E-03
ABL2	0.26	1.20	1.40E-03	7.51E-03
NONO	0.22	1.16	1.40E-03	7.51E-03
RAD21	0.25	1.19	1.40E-03	7.52E-03
UBE3C	-0.24	0.84	1.41E-03	7.54E-03
AMIGO2	-0.36	0.78	1.42E-03	7.61E-03
SUB1	0.24	1.18	1.43E-03	7.63E-03
CYB5RL	0.73	1.66	1.43E-03	7.67E-03
GJB6	0.24	1.18	1.43E-03	7.67E-03
ATXN10	0.23	1.17	1.44E-03	7.68E-03
ENAH	0.22	1.17	1.44E-03	7.71E-03
C5orf22	0.29	1.22	1.44E-03	7.71E-03
RNF215	-0.47	0.72	1.45E-03	7.72E-03
HSPA4L	-0.26	0.83	1.45E-03	7.73E-03
TAGLN	-0.35	0.78	1.45E-03	7.73E-03
CAPN14	-0.64	0.64	1.45E-03	7.73E-03
WNT10B	1.07	2.10	1.45E-03	7.74E-03
GALR2	2.75	6.71	1.45E-03	NA
ZNF488	-0.44	0.74	1.47E-03	7.82E-03
MRPS21	-0.29	0.82	1.47E-03	7.82E-03
LGR4	0.23	1.17	1.47E-03	7.85E-03
HNRNPLL	0.28	1.22	1.48E-03	7.85E-03
H2AFV	0.27	1.21	1.48E-03	7.85E-03
RPL41	-0.21	0.86	1.48E-03	7.88E-03
SARAF	0.25	1.19	1.48E-03	7.88E-03
PART1	-0.85	0.56	1.48E-03	7.88E-03
CDK16	-0.26	0.83	1.48E-03	7.88E-03
PSAT1	0.24	1.18	1.49E-03	7.90E-03
UTP20	0.32	1.25	1.50E-03	7.94E-03
IPPK	0.35	1.27	1.50E-03	7.94E-03
SLC4A2	0.24	1.18	1.50E-03	7.95E-03
STT3B	0.21	1.16	1.50E-03	7.97E-03
ADCY3	0.27	1.21	1.51E-03	7.98E-03
PIEZO1	0.21	1.15	1.51E-03	7.99E-03
JRK	0.32	1.25	1.51E-03	8.01E-03
RMI2	0.41	1.32	1.52E-03	8.03E-03
EFTUD2	0.22	1.16	1.52E-03	8.07E-03
TMX1	0.26	1.20	1.53E-03	8.07E-03
LRSAM1	-0.29	0.82	1.53E-03	8.08E-03
EIF1AX	0.24	1.18	1.53E-03	8.09E-03
STRIP1	-0.28	0.82	1.53E-03	8.10E-03
TMEM203	-0.33	0.80	1.53E-03	8.10E-03
SNX13	0.26	1.20	1.54E-03	8.12E-03
IL1R1	0.45	1.37	1.54E-03	8.13E-03
HACD2	-0.22	0.86	1.55E-03	8.15E-03
HERC4	-0.23	0.85	1.55E-03	8.16E-03
GS1-124K5.11	-0.59	0.66	1.56E-03	8.22E-03
PKD1P6	0.30	1.23	1.56E-03	8.22E-03
GFM1	0.25	1.19	1.58E-03	8.30E-03
EPB41	-0.28	0.83	1.58E-03	8.30E-03
EIF4EBP1	-0.33	0.79	1.59E-03	8.35E-03
SOGA1	-0.23	0.85	1.59E-03	8.36E-03
CHTF8	-0.30	0.81	1.60E-03	8.39E-03
VIPAS39	0.31	1.24	1.60E-03	8.43E-03
CCDC77	0.41	1.33	1.61E-03	8.45E-03
NRIP3	1.15	2.22	1.61E-03	8.47E-03
NSG1	0.28	1.21	1.61E-03	8.47E-03
CA12	0.25	1.19	1.62E-03	8.48E-03
CSNK1G1	-0.26	0.83	1.62E-03	8.48E-03
SEMA4B	0.26	1.20	1.62E-03	8.48E-03
RBPJ	0.27	1.20	1.62E-03	8.49E-03
ANKRD28	0.27	1.20	1.62E-03	8.49E-03

MED29	-0.24	0.85	6.07E-03	3.21E-02
FAT1	0.23	1.18	6.08E-03	3.21E-02
NUTM2B-AS1	0.37	1.30	6.08E-03	3.21E-02
DNAJA1	0.18	1.14	6.08E-03	3.21E-02
NME4	-0.26	0.83	6.09E-03	3.22E-02
SAR1A	0.20	1.15	6.09E-03	3.22E-02
KDM4D	-0.50	0.71	6.11E-03	3.22E-02
CCT3	0.15	1.11	6.11E-03	3.22E-02
SRPK1	0.20	1.14	6.15E-03	3.24E-02
NSDHL	-0.22	0.86	6.15E-03	3.24E-02
DIAPH2	0.30	1.23	6.17E-03	3.25E-02
MTHFD2	-0.21	0.86	6.18E-03	3.25E-02
GOLGA7	0.24	1.18	6.19E-03	3.26E-02
RAD54B	0.48	1.39	6.22E-03	3.27E-02
LRRC37A4P	-0.51	0.70	6.22E-03	3.27E-02
TBRG1	0.26	1.19	6.22E-03	3.27E-02
TJP1	0.18	1.13	6.24E-03	3.28E-02
ZNF146	0.22	1.16	6.26E-03	3.29E-02
DLGAP5	0.23	1.17	6.26E-03	3.29E-02
FAM58A	0.27	1.20	6.27E-03	3.29E-02
RP11-600F24.7	-0.38	0.77	6.27E-03	NA
SMPD1	-0.34	0.79	6.27E-03	3.29E-02
N4BP3	-0.39	0.76	6.28E-03	3.29E-02
DNAH14	0.49	1.40	6.29E-03	3.30E-02
NUDT22	-0.32	0.80	6.31E-03	3.31E-02
CRB3	-0.30	0.81	6.31E-03	3.31E-02
PPRC1	0.23	1.18	6.31E-03	3.31E-02
ADAMTSL5	-0.41	0.75	6.31E-03	3.31E-02
PTPN23	0.23	1.17	6.33E-03	3.32E-02
CTD-2619J13.13	-0.51	0.70	6.35E-03	3.32E-02
WDR46	0.22	1.16	6.36E-03	3.33E-02
DTX3	-0.48	0.72	6.37E-03	3.33E-02
MNAT1	0.28	1.22	6.37E-03	3.33E-02
RPL38	-0.16	0.89	6.37E-03	3.33E-02
01-mar	-0.50	0.71	6.38E-03	3.33E-02
NEURL1B	-0.30	0.81	6.38E-03	3.33E-02
POLE2	-0.34	0.79	6.39E-03	3.33E-02
EHD2	-0.21	0.87	6.43E-03	3.35E-02
STXBP5	0.26	1.20	6.43E-03	3.35E-02
DNAJC3	0.24	1.18	6.44E-03	3.35E-02
CWC25	0.27	1.21	6.44E-03	3.35E-02
PIK3CB	0.23	1.17	6.44E-03	3.35E-02
ITPR2	0.26	1.20	6.44E-03	3.35E-02
PRICKLE3	-0.26	0.83	6.45E-03	3.35E-02
LINC01003	-0.41	0.75	6.46E-03	NA
SHROOM3	0.25	1.19	6.46E-03	3.36E-02
CAPN7	0.25	1.19	6.50E-03	3.38E-02
ACBD5	-0.27	0.83	6.51E-03	3.38E-02
LENG9	-0.47	0.72	6.51E-03	3.38E-02
NMI	-0.44	0.74	6.52E-03	3.39E-02
MMAA	-0.41	0.75	6.57E-03	3.41E-02
HEXDC	0.44	1.35	6.58E-03	3.41E-02
PPP6R2	0.24	1.18	6.63E-03	3.44E-02
INTS12	0.31	1.24	6.65E-03	3.45E-02
STAT6	0.17	1.13	6.66E-03	3.45E-02
MTHFR	-0.32	0.80	6.69E-03	3.47E-02
ELOVL4	-0.33	0.80	6.71E-03	3.48E-02
ADCY7	-0.30	0.81	6.72E-03	3.48E-02
PKIB	0.33	1.26	6.72E-03	3.48E-02
RNASEH2B	-0.32	0.80	6.74E-03	3.49E-02
ECH1	-0.26	0.84	6.75E-03	3.49E-02
IGFBP4	-0.34	0.79	6.76E-03	3.49E-02
ZNF790	0.47	1.38	6.77E-03	3.50E-02
BAP1	0.19	1.14	6.77E-03	3.50E-02
VPS72	-0.22	0.86	6.78E-03	3.50E-02
ENAH	0.20	1.15	6.78E-03	3.50E-02
GSS	0.20	1.15	6.80E-03	3.51E-02
SPAG9	0.20	1.15	6.81E-03	3.51E-02
BLOC1S5	-0.37	0.77	6.82E-03	3.52E-02
CD24P4	0.17	1.13	6.83E-03	3.52E-02
SMPD4	0.20	1.15	6.85E-03	3.53E-02
RP11-1002K11.1	-0.39	0.76	6.88E-03	3.54E-02
COPS7B	0.23	1.18	6.88E-03	3.54E-02
ARHGAP17	0.24	1.18	6.89E-03	3.55E-02
NBPF1	0.29	1.23	6.91E-03	3.56E-02
SAPCD2	-0.25	0.84	6.92E-03	3.56E-02
ZNF774	-0.50	0.71	6.93E-03	3.56E-02
CLUH	0.22	1.16	6.93E-03	3.56E-02
ATG5	0.26	1.19	6.93E-03	3.56E-02
MAOA	-0.24	0.84	6.94E-03	3.57E-02
SLC43A1	-0.50	0.71	6.97E-03	3.58E-02
DNAJB2	-0.25	0.84	6.97E-03	3.58E-02
ROM1	-0.45	0.73	6.97E-03	3.58E-02
CCNG2	-0.30	0.81	6.98E-03	3.58E-02
SPC24	-0.31	0.80	6.98E-03	3.58E-02
KIAA1033	0.25	1.19	7.01E-03	3.59E-02
PLA2G4A	-0.30	0.81	7.03E-03	3.60E-02
CCDC150	0.46	1.38	7.04E-03	3.61E-02
TVP23B	0.35	1.28	7.05E-03	3.61E-02
GLYR1	0.21	1.16	7.06E-03	3.61E-02
CASP8	0.35	1.27	1.62E-03	8.49E-03
FNBP1L	0.29	1.22	1.62E-03	8.50E-03
RAB3B	0.71	1.63	1.63E-03	8.51E-03
MISP	0.30	1.23	1.63E-03	8.52E-03
SNX19	-0.24	0.85	1.64E-03	8.55E-03
SKIDA1	0.92	1.89	1.64E-03	8.56E-03
TTF1	0.35	1.28	1.64E-03	8.58E-03
PFKP	0.22	1.16	1.64E-03	8.58E-03
NUDT15	0.30	1.23	1.65E-03	8.59E-03
LZTR1	-0.25	0.84	1.65E-03	8.60E-03
PTAFR	-0.35	0.78	1.65E-03	8.62E-03
ABCA7	-0.25	0.84	1.66E-03	8.64E-03
BST2	-0.45	0.73	1.66E-03	8.67E-03
CCNL2	-0.26	0.84	1.66E-03	8.67E-03
ZNF736	0.38	1.30	1.66E-03	8.67E-03
CDC42SE2	0.27	1.21	1.67E-03	8.69E-03
EIF5B	0.22	1.17	1.67E-03	8.70E-03
ZFP36L2	-0.25	0.84	1.67E-03	8.71E-03
TNFSF12	-1.30	0.41	1.68E-03	8.72E-03
SPIN1	-0.25	0.84	1.68E-03	8.72E-03
KDM2A	-0.21	0.86	1.68E-03	8.72E-03
VAC14	-0.26	0.83	1.68E-03	8.73E-03
MND1	0.55	1.47	1.68E-03	8.74E-03
ZNF503	-0.31	0.81	1.68E-03	8.74E-03
CLCF1	-0.66	0.63	1.68E-03	8.74E-03
SH3BP5L	-0.26	0.83	1.69E-03	8.75E-03
TARDBP	0.23	1.17	1.70E-03	8.79E-03
ZNF217	0.25	1.19	1.70E-03	8.79E-03
CHP1	-0.22	0.86	1.71E-03	8.85E-03
RASL10B	1.30	2.47	1.71E-03	8.86E-03
DTYMK	0.30	1.23	1.72E-03	8.90E-03
C9orf16	-0.32	0.80	1.72E-03	8.91E-03
SLC25A36	0.24	1.18	1.72E-03	8.91E-03
NMU	0.37	1.29	1.72E-03	8.91E-03
GPT2	0.28	1.21	1.72E-03	8.91E-03
PABPC4	0.22	1.17	1.73E-03	8.92E-03
SUV39H2	0.40	1.32	1.73E-03	8.92E-03
DOCK3	-0.76	0.59	1.74E-03	8.96E-03
ANKRD36C	-0.65	0.64	1.74E-03	8.97E-03
DFNA5	-0.22	0.86	1.74E-03	9.00E-03
C1D	0.34	1.27	1.75E-03	9.02E-03
NPTN	0.22	1.17	1.75E-03	9.03E-03
C14orf80	0.41	1.33	1.76E-03	9.05E-03
CSRNP3	-0.85	0.56	1.76E-03	9.08E-03
AMMECR1L	-0.26	0.84	1.77E-03	9.09E-03
MIS12	0.30	1.23	1.78E-03	9.16E-03
NOL3	-0.38	0.77	1.78E-03	9.16E-03
ENOX2	0.30	1.23	1.79E-03	9.21E-03
DLG5	0.28	1.21	1.79E-03	9.22E-03
PTPRJ	0.55	1.46	1.81E-03	9.29E-03
WWC1	0.27	1.20	1.81E-03	9.30E-03
CTPS1	0.24	1.18	1.82E-03	9.35E-03
CYP4B1	-0.81	0.57	1.83E-03	9.40E-03
MSH2	0.28	1.22	1.85E-03	9.51E-03
PFKM	0.23	1.17	1.86E-03	9.52E-03
ZNF292	0.31	1.24	1.86E-03	9.52E-03
CEL	-0.69	0.62	1.86E-03	9.54E-03
TARS	0.21	1.16	1.86E-03	9.55E-03
LACC1	-0.40	0.76	1.88E-03	9.65E-03
CDC123	0.26	1.20	1.88E-03	9.65E-03
ADNP	0.22	1.17	1.89E-03	9.69E-03
TOB1	-0.25	0.84	1.89E-03	9.70E-03
CTD-2228K2.7	-0.36	0.78	1.90E-03	9.70E-03
NES	0.98	1.98	1.90E-03	9.71E-03
BZW1	0.22	1.16	1.91E-03	9.74E-03
SGOL1	0.54	1.46	1.92E-03	9.83E-03
FCHO1	0.60	1.52	1.92E-03	9.83E-03
ADSL	0.27	1.21	1.92E-03	9.83E-03
ESCO2	0.40	1.32	1.94E-03	9.88E-03
ERO1B	0.67	1.59	1.95E-03	9.93E-03
LCORL	0.50	1.41	1.95E-03	9.93E-03
ANKRD18A	-0.57	0.67	1.96E-03	9.98E-03
USF3	-0.36	0.78	1.97E-03	1.00E-02
NUP205	0.26	1.20	1.97E-03	1.00E-02
ZC3H15	0.24	1.18	1.97E-03	1.00E-02
ZNF91	0.35	1.28	1.98E-03	1.01E-02
RAB17	1.23	2.35	1.98E-03	1.01E-02
IPO8	0.24	1.18	1.98E-03	1.01E-02
RAB4B	-0.72	0.61	1.99E-03	1.01E-02
CKAP2	0.22	1.17	1.99E-03	1.01E-02
TESK2	-0.47	0.72	1.99E-03	1.01E-02
ZNF219	-0.25	0.84	1.99E-03	1.01E-02
FBXO42	-0.28	0.82	2.00E-03	1.01E-02
LPCAT3	-0.40	0.76	2.00E-03	1.01E-02
HDAC6	-0.27	0.83	2.00E-03	1.01E-02
CCDC58	0.37	1.29	2.01E-03	1.02E-02
ATL2	0.25	1.19	2.01E-03	1.02E-02
ETF1	0.20	1.15	2.02E-03	1.02E-02
NAP1L4	0.21	1.16	2.02E-03	1.02E-02
REPS1	0.25	1.19	2.02E-03	1.03E-02

HOXC11	-0.42	0.75	7.08E-03	3.62E-02
RMND5B	-0.25	0.84	7.09E-03	3.62E-02
ASF1B	-0.27	0.83	7.09E-03	3.62E-02
OPLAH	-0.32	0.80	7.09E-03	3.62E-02
FBXL15	-0.46	0.73	7.10E-03	3.62E-02
XRCC2	0.34	1.26	7.10E-03	3.63E-02
BORCS5	-0.39	0.76	7.15E-03	3.65E-02
ATP5D	-0.26	0.84	7.15E-03	3.65E-02
CCNK	0.31	1.24	7.17E-03	3.65E-02
FAM83F	-0.25	0.84	7.18E-03	3.66E-02
ORAI2	-0.47	0.72	7.20E-03	3.67E-02
PRRT1	-0.47	0.72	7.22E-03	3.68E-02
ZNF500	-0.42	0.75	7.23E-03	3.68E-02
NPIPB4	0.50	1.42	7.23E-03	3.68E-02
CYB5A	-0.27	0.83	7.25E-03	3.69E-02
CBLL1	0.26	1.20	7.25E-03	3.69E-02
TTC27	0.24	1.18	7.27E-03	3.70E-02
PDXK	-0.19	0.88	7.28E-03	3.70E-02
FBL	-0.16	0.89	7.28E-03	3.70E-02
SNX1	0.18	1.14	7.30E-03	3.71E-02
FAM84A	-0.23	0.85	7.31E-03	3.71E-02
TP53I13	-0.32	0.80	7.33E-03	3.72E-02
FAXC	-0.48	0.72	7.33E-03	3.72E-02
TGIF2	-0.32	0.80	7.35E-03	3.73E-02
C19orf57	-0.47	0.72	7.35E-03	3.73E-02
B3GNT8	-0.43	0.74	7.36E-03	3.73E-02
ANKRD27	0.22	1.16	7.36E-03	3.73E-02
DNAJB1	-0.15	0.90	7.36E-03	3.73E-02
GNA15	0.22	1.16	7.37E-03	3.73E-02
MAPRE2	-0.26	0.84	7.37E-03	3.73E-02
SMIM20	0.36	1.28	7.38E-03	3.73E-02
DIP2A	0.21	1.16	7.38E-03	3.73E-02
ILVBL	-0.22	0.86	7.41E-03	3.74E-02
ELL	0.30	1.23	7.44E-03	3.76E-02
HIPK1	0.20	1.15	7.44E-03	3.76E-02
ZNF518A	-0.29	0.82	7.45E-03	3.76E-02
TMEM39A	0.27	1.20	7.46E-03	3.76E-02
RGS14	-0.31	0.81	7.46E-03	3.76E-02
PDE4A	-0.39	0.76	7.46E-03	NA
NUS1	0.29	1.22	7.48E-03	3.77E-02
PDE5A	-0.50	0.71	7.49E-03	3.77E-02
WTAP	0.23	1.17	7.50E-03	3.78E-02
ZNF16	-0.46	0.73	7.50E-03	3.78E-02
USP20	0.31	1.24	7.51E-03	3.78E-02
OPA1	0.21	1.15	7.54E-03	3.79E-02
IQGAP1	0.17	1.12	7.55E-03	3.80E-02
NCLN	0.24	1.18	7.56E-03	3.80E-02
BCLAF1	-0.18	0.88	7.58E-03	3.81E-02
ABI1	0.19	1.14	7.58E-03	3.81E-02
SNHG14	-0.29	0.82	7.61E-03	3.82E-02
STEAP1B	-0.48	0.72	7.61E-03	3.82E-02
CPT1A	-0.20	0.87	7.61E-03	3.82E-02
ZWILCH	0.23	1.17	7.62E-03	3.83E-02
AQP9	-0.33	0.80	7.63E-03	3.83E-02
MINK1	0.17	1.13	7.63E-03	3.83E-02
SAMHD1	-0.33	0.80	7.64E-03	3.83E-02
STARD7	0.17	1.13	7.64E-03	3.83E-02
CTD-2510F5.4	0.46	1.38	7.64E-03	3.83E-02
USH1G	-0.41	0.75	7.67E-03	NA
MVB12B	-0.41	0.75	7.74E-03	3.87E-02
UGCG	0.22	1.16	7.74E-03	3.87E-02
APCDD1	-0.49	0.71	7.74E-03	3.87E-02
DHCR24	-0.15	0.90	7.74E-03	3.87E-02
IMMP2L	0.41	1.33	7.80E-03	3.90E-02
WASH1	0.45	1.37	7.82E-03	3.90E-02
GNAQ	0.22	1.16	7.82E-03	3.90E-02
PID1	-0.46	0.73	7.83E-03	3.91E-02
FAM212A	-0.37	0.78	7.84E-03	3.91E-02
HEBP2	-0.19	0.87	7.85E-03	3.92E-02
DDX18	0.20	1.15	7.87E-03	3.92E-02
RP11-115H13.1	-0.48	0.72	7.87E-03	3.92E-02
CDH1	0.15	1.11	7.87E-03	3.92E-02
GNAL	-0.26	0.84	7.87E-03	3.92E-02
SPR	-0.33	0.79	7.87E-03	3.92E-02
FLRT3	-0.21	0.86	7.88E-03	3.92E-02
ZNF548	-0.47	0.72	7.90E-03	3.93E-02
ARHGAP11B	0.33	1.26	7.91E-03	3.94E-02
ALKBH2	-0.36	0.78	7.91E-03	3.94E-02
TCOF1	0.19	1.14	7.92E-03	3.94E-02
RTCA	0.24	1.18	7.93E-03	3.94E-02
SLC25A23	-0.20	0.87	7.93E-03	3.94E-02
CEP170	0.30	1.23	7.94E-03	3.94E-02
MRPL44	0.24	1.18	7.94E-03	3.94E-02
ANO7P1	-0.49	0.71	7.95E-03	3.94E-02
NTM	0.31	1.24	7.95E-03	3.94E-02
RNF165	-0.47	0.72	7.96E-03	3.95E-02
ADAMTS14	-0.46	0.73	7.98E-03	3.95E-02
ZNF70	-0.46	0.73	7.99E-03	3.96E-02
BCAR1	0.25	1.19	8.01E-03	3.96E-02
KLHL22	-0.36	0.78	8.02E-03	3.97E-02
KDELR2	0.22	1.16	2.03E-03	1.03E-02
RAB28	-0.45	0.73	2.05E-03	1.04E-02
ADRBK2	-0.34	0.79	2.06E-03	1.04E-02
TTLL3	-0.44	0.73	2.07E-03	1.04E-02
FLG	-0.40	0.76	2.07E-03	1.04E-02
KIAA0196	0.25	1.19	2.07E-03	1.05E-02
NPIPB15	-1.09	0.47	2.07E-03	1.05E-02
DCTD	-0.25	0.84	2.07E-03	1.05E-02
CCNB1	0.64	1.55	2.08E-03	1.05E-02
HMG20A	0.29	1.22	2.08E-03	1.05E-02
HCP5	0.38	1.30	2.08E-03	1.05E-02
TMEM9	0.26	1.20	2.08E-03	1.05E-02
RP11-367G18.1	-0.82	0.57	2.09E-03	1.05E-02
SF3A1	0.22	1.16	2.10E-03	1.06E-02
PIGCP1	-0.61	0.65	2.11E-03	1.06E-02
DRAM1	-0.38	0.77	2.11E-03	1.06E-02
TMED2	0.21	1.15	2.12E-03	1.07E-02
PEF1	-0.25	0.84	2.12E-03	1.07E-02
TRAF3	0.27	1.21	2.12E-03	1.07E-02
IL4R	-0.23	0.85	2.13E-03	1.07E-02
DNAJC9	0.34	1.27	2.13E-03	1.07E-02
CDK5RAP2	0.23	1.17	2.13E-03	1.07E-02
CTNS	-0.37	0.78	2.13E-03	1.07E-02
DAPK2	0.53	1.44	2.14E-03	1.07E-02
CHAF1B	0.39	1.31	2.14E-03	1.07E-02
RASSF8	0.55	1.46	2.14E-03	1.07E-02
DAPP1	-0.34	0.79	2.15E-03	1.08E-02
ATIC	0.24	1.18	2.15E-03	1.08E-02
RP11-277P12.20	-0.70	0.62	2.17E-03	1.09E-02
SCML1	0.32	1.25	2.17E-03	1.09E-02
MCM6	0.33	1.26	2.17E-03	1.09E-02
LGALS9C	-0.40	0.76	2.18E-03	1.09E-02
POLD2	0.23	1.17	2.18E-03	1.09E-02
PLOD1	0.25	1.19	2.18E-03	1.09E-02
KRT6A	-0.20	0.87	2.19E-03	1.09E-02
P2RY2	-0.27	0.83	2.19E-03	1.09E-02
CDK19	-0.46	0.73	2.19E-03	1.09E-02
ZNF384	-0.30	0.81	2.20E-03	1.10E-02
CDCA3	0.38	1.30	2.21E-03	1.10E-02
NCS1	-0.28	0.82	2.22E-03	1.11E-02
OBFC1	-0.34	0.79	2.23E-03	1.11E-02
TTC27	0.28	1.21	2.23E-03	1.11E-02
C12orf65	-0.37	0.77	2.23E-03	1.11E-02
MBNL1	0.22	1.17	2.23E-03	1.11E-02
FNIP1	0.32	1.25	2.24E-03	1.11E-02
SIX4	0.41	1.33	2.24E-03	1.12E-02
SNRPA	0.26	1.20	2.25E-03	1.12E-02
MGAT1	-0.22	0.86	2.25E-03	1.12E-02
DARS	0.25	1.19	2.26E-03	1.12E-02
AC006116.20	-0.93	0.53	2.26E-03	1.13E-02
MCHR1	1.24	2.36	2.27E-03	1.13E-02
ANXA1	-0.25	0.84	2.27E-03	1.13E-02
EMC8	0.30	1.23	2.27E-03	1.13E-02
FN3KRP	0.27	1.21	2.27E-03	1.13E-02
CTC-425O23.5	-0.88	0.54	2.27E-03	1.13E-02
WRNIP1	0.24	1.18	2.28E-03	1.13E-02
MAN2B1	-0.26	0.83	2.28E-03	1.13E-02
RPIA	0.35	1.27	2.29E-03	1.14E-02
UBR1	0.25	1.19	2.29E-03	1.14E-02
MFI2	0.37	1.29	2.30E-03	1.14E-02
PDXDC1	0.22	1.16	2.31E-03	1.14E-02
SETX	0.22	1.16	2.31E-03	1.14E-02
KLHDC7B	-1.64	0.32	2.31E-03	NA
PDDC1	-0.26	0.84	2.32E-03	1.15E-02
KIAA1033	0.23	1.17	2.32E-03	1.15E-02
RSU1	-0.24	0.85	2.32E-03	1.15E-02
COL16A1	0.20	1.15	2.32E-03	1.15E-02
SON	-0.28	0.82	2.33E-03	1.15E-02
RHBDF1	-0.26	0.83	2.34E-03	1.15E-02
EHBP1L1	0.23	1.17	2.34E-03	1.16E-02
NLRP10	-0.70	0.61	2.36E-03	1.16E-02
ZNF600	-0.36	0.78	2.36E-03	1.16E-02
TIMM44	0.26	1.20	2.38E-03	1.17E-02
PER3	0.52	1.44	2.39E-03	1.18E-02
GPR157	0.30	1.23	2.40E-03	1.18E-02
EDEM1	-0.25	0.84	2.40E-03	1.18E-02
PICALM	0.20	1.15	2.41E-03	1.18E-02
OIP5	0.53	1.45	2.41E-03	1.18E-02
ULK3	-0.27	0.83	2.41E-03	1.19E-02
KLC2	-0.27	0.83	2.42E-03	1.19E-02
SLC39A6	-0.22	0.86	2.42E-03	1.19E-02
HECTD2	0.52	1.44	2.42E-03	1.19E-02
IMPAD1	0.22	1.17	2.42E-03	1.19E-02
NFYB	0.34	1.26	2.42E-03	1.19E-02
TRAIP	0.39	1.31	2.43E-03	1.19E-02
TMEM140	-0.50	0.71	2.43E-03	1.19E-02
MYDGF	-0.26	0.84	2.45E-03	1.20E-02
LAMB4	-1.20	0.43	2.45E-03	1.20E-02
SLC35D1	0.23	1.17	2.45E-03	1.20E-02
HEATR1	0.24	1.18	2.46E-03	1.20E-02

PTPN21	0.28	1.21	8.02E-03	3.97E-02	ZWILCH	0.35	1.28	2.46E-03	1.20E-02
FBXW2	0.18	1.13	8.03E-03	3.97E-02	PSMA5	0.22	1.17	2.46E-03	1.20E-02
ARMC8	0.24	1.18	8.05E-03	3.98E-02	CLTA	-0.22	0.86	2.46E-03	1.20E-02
ZNF296	-0.46	0.73	8.06E-03	3.99E-02	GJA1	-0.23	0.85	2.46E-03	1.20E-02
RP5-1052M9.1	-0.41	0.75	8.07E-03	3.99E-02	NACA	0.19	1.14	2.46E-03	1.20E-02
RBM43	-0.47	0.72	8.08E-03	3.99E-02	MGLL	-0.29	0.82	2.46E-03	1.20E-02
B4GAT1	-0.35	0.78	8.08E-03	3.99E-02	C20orf194	-0.29	0.82	2.47E-03	1.21E-02
BORA	0.34	1.26	8.09E-03	3.99E-02	PGBD5	-0.52	0.70	2.48E-03	1.21E-02
LYNX1	-0.48	0.72	8.10E-03	4.00E-02	CHUK	0.28	1.21	2.48E-03	1.21E-02
FUT11	-0.33	0.80	8.10E-03	4.00E-02	INCENP	0.25	1.19	2.49E-03	1.21E-02
SPATS2L	0.19	1.14	8.11E-03	4.00E-02	PPIG	0.24	1.18	2.49E-03	1.21E-02
ANKS1A	0.20	1.14	8.12E-03	4.00E-02	GINS2	-0.43	0.74	2.49E-03	1.21E-02
ZBED5	0.29	1.22	8.12E-03	4.00E-02	KIF26A	1.21	2.31	2.50E-03	1.22E-02
BNIP2	0.24	1.18	8.12E-03	4.00E-02	MAF	-0.32	0.80	2.50E-03	1.22E-02
SYDE1	-0.34	0.79	8.13E-03	4.00E-02	DOCK8	-0.37	0.77	2.50E-03	1.22E-02
FAM107B	0.30	1.24	8.15E-03	4.01E-02	XAF1	-0.45	0.73	2.50E-03	1.22E-02
CARD14	-0.43	0.74	8.18E-03	4.03E-02	POLE2	0.45	1.37	2.50E-03	1.22E-02
TJAP1	0.26	1.20	8.20E-03	4.03E-02	NEFH	0.45	1.36	2.50E-03	1.22E-02
EPS15	0.20	1.14	8.24E-03	4.05E-02	MAD2L2	0.32	1.25	2.52E-03	1.23E-02
C16orf59	-0.36	0.78	8.27E-03	4.07E-02	RP11-164J13.1	-0.97	0.51	2.52E-03	1.23E-02
UBIAD1	-0.31	0.81	8.28E-03	4.07E-02	PDHX	0.28	1.22	2.53E-03	1.23E-02
NOP58	0.19	1.14	8.30E-03	4.08E-02	MLANA	-1.40	0.38	2.53E-03	NA
PEBP1	-0.16	0.90	8.30E-03	4.08E-02	C12orf4	0.30	1.23	2.54E-03	1.23E-02
MT1X	-0.26	0.84	8.30E-03	4.08E-02	NUP155	0.25	1.19	2.54E-03	1.24E-02
ATAD1	0.23	1.17	8.32E-03	4.08E-02	ANKRD65	-0.36	0.78	2.55E-03	1.24E-02
PCM1	0.21	1.15	8.33E-03	4.08E-02	LMAN2L	0.29	1.23	2.56E-03	1.24E-02
TSR3	-0.26	0.84	8.33E-03	4.09E-02	APPBP2	-0.28	0.82	2.56E-03	1.24E-02
SDPR	-0.33	0.80	8.34E-03	4.09E-02	CDK5RAP1	0.27	1.21	2.57E-03	1.25E-02
DHRS11	-0.29	0.82	8.35E-03	4.09E-02	MMD	0.55	1.46	2.57E-03	1.25E-02
RNF149	0.24	1.18	8.36E-03	4.09E-02	RPS19	-0.19	0.88	2.57E-03	1.25E-02
DDX42	0.17	1.13	8.38E-03	4.10E-02	ZMYM4	0.24	1.18	2.61E-03	1.26E-02
POLR3A	0.22	1.16	8.38E-03	4.10E-02	ENTPD3	-0.34	0.79	2.61E-03	1.26E-02
ROCK1	0.20	1.15	8.39E-03	4.10E-02	KIFC3	-0.34	0.79	2.62E-03	1.27E-02
RSBN1	0.30	1.23	8.41E-03	4.11E-02	FAM136A	0.28	1.21	2.62E-03	1.27E-02
RPL14P1	-0.48	0.72	8.43E-03	4.12E-02	OPA1	0.22	1.16	2.62E-03	1.27E-02
TARS2	-0.21	0.87	8.45E-03	4.13E-02	TNIP2	0.29	1.22	2.63E-03	1.27E-02
METTL2A	0.29	1.22	8.49E-03	4.14E-02	NABP2	-0.29	0.82	2.64E-03	1.27E-02
TMEM87B	0.26	1.20	8.49E-03	4.14E-02	CLCA4	-0.36	0.78	2.64E-03	1.28E-02
MBP	0.20	1.15	8.51E-03	4.15E-02	THOP1	0.23	1.17	2.65E-03	1.28E-02
PCID2	0.25	1.19	8.51E-03	4.15E-02	PLD3	-0.22	0.86	2.65E-03	1.28E-02
PITPNC1	0.22	1.16	8.51E-03	4.15E-02	MLKL	0.35	1.27	2.65E-03	1.28E-02
SNHG3	-0.30	0.81	8.54E-03	4.16E-02	SULF2	0.22	1.16	2.66E-03	1.28E-02
PLEKHA2	-0.22	0.86	8.55E-03	4.17E-02	SLC6A6	0.32	1.25	2.66E-03	1.28E-02
GRK5	0.35	1.27	8.55E-03	4.17E-02	ACAT2	-0.22	0.86	2.66E-03	1.29E-02
VPS13A	0.25	1.19	8.56E-03	4.17E-02	HOOK1	0.25	1.19	2.67E-03	1.29E-02
RAB22A	0.22	1.16	8.61E-03	4.19E-02	NAV1	-0.28	0.82	2.67E-03	1.29E-02
BTN3A1	-0.44	0.74	8.64E-03	4.20E-02	ZNF383	-0.48	0.72	2.68E-03	1.29E-02
SMARCAD1	-0.25	0.84	8.64E-03	4.20E-02	UVSSA	-0.35	0.79	2.68E-03	1.29E-02
DEF6	0.23	1.17	8.64E-03	4.20E-02	NIPA2	0.26	1.20	2.69E-03	1.29E-02
ITPKC	0.20	1.15	8.65E-03	4.20E-02	KIAA0101	0.52	1.44	2.69E-03	1.30E-02
TMEM164	-0.21	0.86	8.66E-03	4.21E-02	ZFP92	-0.68	0.62	2.70E-03	1.30E-02
PLEKHG6	-0.30	0.81	8.66E-03	4.21E-02	PPP3CB	0.25	1.19	2.70E-03	1.30E-02
COL9A2	-0.46	0.73	8.69E-03	4.22E-02	EXD2	-0.27	0.83	2.70E-03	1.30E-02
PAXIP1	0.25	1.19	8.69E-03	4.22E-02	FAM89A	-0.36	0.78	2.70E-03	1.30E-02
TSEN34	-0.26	0.84	8.71E-03	4.23E-02	ZBTB26	0.48	1.40	2.70E-03	1.30E-02
RALGPS1	0.45	1.37	8.71E-03	4.23E-02	TXLNG	0.31	1.24	2.70E-03	1.30E-02
PRPF4B	0.21	1.16	8.71E-03	4.23E-02	EIF5	0.22	1.16	2.71E-03	1.30E-02
ADCK4	-0.30	0.81	8.73E-03	4.23E-02	NFIB	0.33	1.26	2.71E-03	1.30E-02
PLXNB2	-0.19	0.87	8.73E-03	4.23E-02	MT1X	0.48	1.39	2.71E-03	1.30E-02
KTN1	0.21	1.16	8.74E-03	4.23E-02	PSMC5	0.22	1.17	2.72E-03	1.30E-02
KLHL29	0.27	1.20	8.75E-03	4.24E-02	POLDIP3	0.23	1.18	2.72E-03	1.30E-02
FAM53B	-0.26	0.83	8.76E-03	4.24E-02	PDPN	-0.32	0.80	2.72E-03	1.30E-02
BIN3	0.29	1.22	8.78E-03	4.25E-02	PLEKHA3	0.40	1.32	2.72E-03	1.31E-02
MT-ND6	0.40	1.32	8.79E-03	4.25E-02	SIPA1	0.30	1.23	2.73E-03	1.31E-02
ARHGAP40	-0.33	0.80	8.80E-03	4.26E-02	POMK	-0.54	0.69	2.73E-03	1.31E-02
SLC37A4	-0.31	0.80	8.82E-03	4.27E-02	FEZ2	0.29	1.22	2.74E-03	1.31E-02
ZNF827	-0.34	0.79	8.83E-03	4.27E-02	PRDX5	-0.22	0.86	2.74E-03	1.31E-02
GHDC	-0.37	0.77	8.85E-03	4.27E-02	IFIT1	-0.62	0.65	2.74E-03	1.31E-02
BCL2L11	-0.39	0.76	8.85E-03	4.28E-02	MPHOSPH10	0.28	1.21	2.75E-03	1.31E-02
MOB3B	0.21	1.16	8.87E-03	4.28E-02	SNIP1	-0.37	0.77	2.76E-03	1.32E-02
UTP18	0.25	1.19	8.87E-03	4.28E-02	GRPEL1	0.25	1.19	2.78E-03	1.33E-02
UPK1B	-0.18	0.88	8.91E-03	4.30E-02	ATP2A2	0.19	1.14	2.78E-03	1.33E-02
RIPK2	0.28	1.22	8.92E-03	4.30E-02	LSM4	0.22	1.16	2.78E-03	1.33E-02
STK26	0.24	1.18	8.92E-03	4.30E-02	TGFBRAP1	-0.26	0.84	2.78E-03	1.33E-02
MICALL2	-0.32	0.80	8.92E-03	4.30E-02	PCGF6	0.38	1.30	2.78E-03	1.33E-02
C19orf60	-0.38	0.77	8.97E-03	4.32E-02	CASP10	-0.34	0.79	2.79E-03	1.33E-02
HIF1AN	-0.20	0.87	8.98E-03	4.33E-02	RSAD2	-0.54	0.69	2.79E-03	1.33E-02
RP11-705C15.2	-0.48	0.72	8.99E-03	4.33E-02	PITPNM1	-0.24	0.85	2.80E-03	1.33E-02
HMGN2	-0.21	0.86	9.00E-03	4.33E-02	PCNX	0.23	1.17	2.80E-03	1.33E-02
BTN3A3	-0.49	0.71	9.01E-03	4.33E-02	ABR	-0.24	0.84	2.80E-03	1.33E-02
FBLN1	-0.22	0.86	9.02E-03	4.34E-02	WAPL	0.23	1.17	2.81E-03	1.34E-02
CHFR	0.23	1.17	9.06E-03	4.35E-02	ENOPH1	0.26	1.20	2.81E-03	1.34E-02
MORC3	0.27	1.20	9.07E-03	4.36E-02	PSMD1	0.21	1.15	2.81E-03	1.34E-02
RTCB	0.17	1.13	9.07E-03	4.36E-02	CLSTN1	-0.21	0.86	2.83E-03	1.35E-02
PEX5	0.23	1.17	9.07E-03	4.36E-02	NUP210	0.59	1.51	2.84E-03	1.35E-02
TVP23C	0.44	1.36	9.07E-03	4.36E-02	C16orf58	-0.31	0.81	2.84E-03	1.35E-02
KRT16P6	0.37	1.29	9.08E-03	4.36E-02	SCAF4	-0.27	0.83	2.84E-03	1.35E-02
PNPT1	0.20	1.15	9.09E-03	4.36E-02	FRAS1	0.28	1.21	2.84E-03	1.35E-02
THRA	-0.33	0.80	9.11E-03	4.37E-02	07-sep	0.21	1.16	2.84E-03	1.35E-02
C9orf142	-0.34	0.79	9.17E-03	4.40E-02	TMEM94	-0.27	0.83	2.84E-03	1.35E-02

SLC37A2	-0.23	0.85	9.17E-03	4.40E-02
PEG13	-0.37	0.77	9.18E-03	NA
SCYL1	0.20	1.15	9.18E-03	4.40E-02
SPINK7	-0.48	0.72	9.19E-03	4.40E-02
DCAF13	0.23	1.18	9.22E-03	4.42E-02
RSF1	0.25	1.19	9.25E-03	4.43E-02
TMCC1	0.24	1.18	9.25E-03	4.43E-02
HDDC3	-0.44	0.74	9.26E-03	4.43E-02
BRIX1	0.25	1.19	9.26E-03	4.43E-02
FBXO46	-0.35	0.78	9.27E-03	4.43E-02
INTS9	0.27	1.20	9.29E-03	4.44E-02
MRE11A	0.29	1.22	9.32E-03	4.45E-02
DYNC1LI1	0.21	1.16	9.35E-03	4.46E-02
RAD9A	-0.34	0.79	9.36E-03	4.47E-02
BCORL1	-0.34	0.79	9.36E-03	4.47E-02
CNTLN	-0.38	0.77	9.37E-03	4.47E-02
PER3	-0.36	0.78	9.37E-03	4.47E-02
MAB21L3	-0.47	0.72	9.37E-03	4.47E-02
SMS	0.19	1.14	9.41E-03	4.48E-02
TGFA	0.17	1.12	9.41E-03	4.48E-02
SMG5	0.20	1.15	9.42E-03	4.48E-02
TRIM41	0.26	1.19	9.42E-03	4.48E-02
FBXO4	-0.41	0.75	9.43E-03	4.48E-02
KIAA1191	0.19	1.14	9.45E-03	4.49E-02
HIBCH	0.33	1.25	9.46E-03	4.49E-02
ELP3	0.22	1.17	9.48E-03	4.50E-02
DIMT1	0.23	1.18	9.51E-03	4.52E-02
IQCE	0.28	1.21	9.51E-03	4.52E-02
TNF	0.42	1.34	9.53E-03	4.52E-02
RNF26	0.25	1.19	9.53E-03	4.52E-02
RP5-862P8.2	0.28	1.21	9.53E-03	4.52E-02
TXNDC12	0.20	1.15	9.55E-03	4.53E-02
ANP32B	-0.16	0.89	9.56E-03	4.53E-02
PTTG1IP	0.17	1.13	9.57E-03	4.53E-02
SLC39A4	-0.35	0.79	9.58E-03	4.54E-02
SRP72	0.17	1.12	9.63E-03	4.56E-02
ADRM1	0.25	1.19	9.64E-03	4.56E-02
CLHC1	0.48	1.40	9.65E-03	4.57E-02
DOCK10	-0.38	0.77	9.65E-03	NA
SNRNP200	0.15	1.11	9.66E-03	4.57E-02
NMT1	0.18	1.13	9.71E-03	4.59E-02
TMEM45A	-0.26	0.84	9.71E-03	4.59E-02
SNX2	0.22	1.16	9.72E-03	4.59E-02
MPHOSPH8	0.24	1.18	9.74E-03	4.60E-02
CMTM4	0.21	1.16	9.75E-03	4.61E-02
NOL11	0.19	1.14	9.76E-03	4.61E-02
GTF2F2	0.23	1.17	9.79E-03	4.62E-02
VWF	-0.44	0.74	9.80E-03	4.62E-02
RNF17	0.48	1.40	9.81E-03	4.62E-02
MAF	-0.46	0.73	9.81E-03	4.62E-02
RGS16	-0.45	0.73	9.81E-03	4.62E-02
SHF	-0.31	0.81	9.82E-03	4.62E-02
UBE3A	0.22	1.17	9.82E-03	4.62E-02
WIZ	-0.24	0.85	9.82E-03	4.62E-02
ABHD11	-0.32	0.80	9.83E-03	4.62E-02
PRRC2B	0.20	1.15	9.85E-03	4.63E-02
MT-ND2	0.40	1.32	9.86E-03	4.64E-02
SF3A1	0.17	1.13	9.95E-03	4.68E-02
MT-ND4	0.40	1.32	9.96E-03	4.68E-02
MRPS6	0.27	1.21	9.97E-03	4.68E-02
DOPEY1	-0.27	0.83	9.98E-03	4.69E-02
SRGAP3	-0.27	0.83	9.99E-03	4.69E-02
ULK4	0.45	1.37	1.00E-02	4.70E-02
POLA2	-0.23	0.85	1.00E-02	4.70E-02
NIPAL4	0.21	1.16	1.00E-02	4.71E-02
REL	0.31	1.24	1.00E-02	4.71E-02
UBE2C	-0.20	0.87	1.01E-02	4.73E-02
RNF167	-0.20	0.87	1.01E-02	4.73E-02
ARHGAP25	-0.35	0.78	1.01E-02	4.74E-02
WDR3	0.20	1.15	1.01E-02	4.74E-02
CCDC18	0.36	1.28	1.01E-02	4.74E-02
PPP2R5B	0.28	1.21	1.01E-02	4.74E-02
STARD3	0.24	1.18	1.01E-02	4.74E-02
CORO1C	0.15	1.11	1.02E-02	4.75E-02
MYB	-0.43	0.74	1.02E-02	4.75E-02
KRT18	0.18	1.14	1.02E-02	4.77E-02
MRPL11	-0.21	0.87	1.02E-02	4.77E-02
MXRA8	-0.46	0.73	1.02E-02	4.77E-02
ELFN2	-0.48	0.72	1.02E-02	4.77E-02
HMOX1	-0.48	0.72	1.02E-02	4.77E-02
B3GALNT1	-0.42	0.75	1.03E-02	4.81E-02
SZRD1	0.19	1.14	1.03E-02	4.82E-02
PPP6R1	0.21	1.15	1.04E-02	4.83E-02
LINC00511	-0.37	0.77	1.04E-02	4.83E-02
RHBDF1	-0.27	0.83	1.04E-02	4.84E-02
KDM1A	0.17	1.12	1.04E-02	4.85E-02
LRRFIP2	0.18	1.14	1.04E-02	4.85E-02
KIAA0513	-0.32	0.80	1.04E-02	4.85E-02
KANK2	-0.29	0.82	1.04E-02	4.85E-02
LTBP3	-0.33	0.80	1.04E-02	4.85E-02
INPP4A	-0.27	0.83	2.85E-03	1.35E-02
CADM1	0.46	1.38	2.85E-03	1.35E-02
FOXN1	0.34	1.26	2.85E-03	1.35E-02
ARSB	0.41	1.33	2.87E-03	1.36E-02
S100P	-0.29	0.82	2.87E-03	1.36E-02
TLE1	0.26	1.20	2.88E-03	1.37E-02
ATAD5	0.38	1.30	2.89E-03	1.37E-02
FICD	0.64	1.56	2.89E-03	1.37E-02
STK38L	0.25	1.19	2.90E-03	1.37E-02
HELLS	0.33	1.26	2.91E-03	1.38E-02
CCDC64B	-0.35	0.79	2.91E-03	1.38E-02
SKAP2	0.27	1.21	2.91E-03	1.38E-02
RMND5B	-0.29	0.82	2.93E-03	1.38E-02
ARL5A	0.25	1.19	2.93E-03	1.39E-02
TEAD3	-0.25	0.84	2.95E-03	1.39E-02
UBB	0.24	1.18	2.96E-03	1.40E-02
C20orf27	0.27	1.20	2.96E-03	1.40E-02
INO80E	-0.28	0.82	2.96E-03	1.40E-02
HBS1L	0.23	1.17	2.96E-03	1.40E-02
SLC44A5	-0.43	0.74	2.98E-03	1.40E-02
ANXA4	-0.21	0.86	2.99E-03	1.41E-02
EPS15	0.24	1.18	3.00E-03	1.41E-02
PTGER4	-0.59	0.66	3.00E-03	1.41E-02
ATP6V1B2	0.23	1.17	3.01E-03	1.42E-02
CLCN2	0.41	1.33	3.02E-03	1.42E-02
RTN3	0.21	1.16	3.02E-03	1.42E-02
NLK	0.36	1.28	3.04E-03	1.43E-02
PSMC4	0.22	1.17	3.04E-03	1.43E-02
CNGA1	-0.64	0.64	3.05E-03	1.43E-02
GALNT2	0.19	1.14	3.05E-03	1.43E-02
WDR76	0.37	1.30	3.05E-03	1.43E-02
SRSF5	-0.21	0.86	3.07E-03	1.44E-02
ADIPOR2	0.21	1.15	3.07E-03	1.44E-02
CHD1	0.25	1.19	3.07E-03	1.44E-02
COPG1	-0.21	0.87	3.07E-03	1.44E-02
CDCA7L	0.40	1.32	3.08E-03	1.44E-02
STRN	0.23	1.17	3.09E-03	1.45E-02
CTBP1-AS2	-0.56	0.68	3.10E-03	1.45E-02
ZNF732	0.70	1.62	3.11E-03	1.46E-02
PPIL1	0.29	1.22	3.11E-03	1.46E-02
TPPP	-0.47	0.72	3.12E-03	1.46E-02
KRT75	0.41	1.33	3.13E-03	1.46E-02
MBD2	0.22	1.16	3.13E-03	1.47E-02
ME2	0.26	1.20	3.14E-03	1.47E-02
C5orf34	0.51	1.43	3.14E-03	1.47E-02
PSMB1	0.21	1.16	3.14E-03	1.47E-02
NAE1	0.23	1.17	3.16E-03	1.47E-02
PSMB9	0.42	1.34	3.19E-03	1.49E-02
CLPX	0.25	1.19	3.19E-03	1.49E-02
DEDD2	0.32	1.25	3.19E-03	1.49E-02
PIK3C2A	0.22	1.16	3.19E-03	1.49E-02
SLC43A1	1.00	2.00	3.19E-03	1.49E-02
GTF2I	-0.25	0.84	3.20E-03	1.49E-02
FLJ26850	-2.32	0.20	3.20E-03	NA
SRSF1	0.24	1.18	3.20E-03	1.49E-02
UACA	0.27	1.21	3.21E-03	1.49E-02
CNIH1	0.21	1.16	3.21E-03	1.50E-02
RBM14	0.24	1.18	3.22E-03	1.50E-02
FAM98A	0.22	1.17	3.22E-03	1.50E-02
FECH	0.28	1.21	3.22E-03	1.50E-02
PRRT4	0.96	1.95	3.23E-03	1.50E-02
CNEP1R1	0.37	1.30	3.24E-03	1.51E-02
UIMC1	-0.30	0.81	3.24E-03	1.51E-02
PDCD10	0.25	1.19	3.24E-03	1.51E-02
AGO4	0.32	1.25	3.25E-03	1.51E-02
KCMF1	0.24	1.18	3.25E-03	1.51E-02
VPS26A	0.22	1.17	3.26E-03	1.51E-02
NIPSNAP1	0.23	1.17	3.26E-03	1.51E-02
CDKN2AIPNL	0.36	1.28	3.27E-03	1.52E-02
SLC22A17	0.51	1.43	3.27E-03	1.52E-02
KLHL42	0.26	1.20	3.27E-03	1.52E-02
HN1L	0.20	1.15	3.30E-03	1.53E-02
MARVELD2	-0.27	0.83	3.30E-03	1.53E-02
AKAP1	0.26	1.20	3.30E-03	1.53E-02
TMEM219	-0.25	0.84	3.31E-03	1.53E-02
PKN3	0.37	1.29	3.34E-03	1.54E-02
SSBP2	-0.38	0.77	3.34E-03	1.55E-02
GATAD1	-0.31	0.81	3.35E-03	1.55E-02
PTPN4	0.28	1.22	3.37E-03	1.56E-02
C18orf54	0.35	1.28	3.39E-03	1.56E-02
DDX24	0.21	1.15	3.39E-03	1.56E-02
STK10	0.28	1.22	3.39E-03	1.56E-02
DDX60	-0.22	0.86	3.39E-03	1.56E-02
CNNM1	1.18	2.26	3.39E-03	1.56E-02
NADK2	0.31	1.24	3.40E-03	1.57E-02
SLC45A3	-0.53	0.69	3.40E-03	1.57E-02
RP11-297A16.4	-0.71	0.61	3.43E-03	1.58E-02
ADRA1B	1.09	2.13	3.44E-03	1.59E-02
KRTDAP	-0.25	0.84	3.44E-03	1.59E-02
LINC00886	-0.64	0.64	3.45E-03	1.59E-02

INPPL1	0.23	1.17	1.05E-02	4.86E-02
DNM1L	0.18	1.13	1.05E-02	4.88E-02
TLCD1	-0.39	0.76	1.05E-02	4.88E-02
HPCAL1	0.27	1.21	1.05E-02	4.88E-02
CTD-2267D19.3	-0.47	0.72	1.05E-02	4.89E-02
CDC16	0.21	1.15	1.05E-02	4.89E-02
ESYT2	0.17	1.13	1.05E-02	4.89E-02
SLC30A4	0.40	1.32	1.05E-02	4.89E-02
QSOX2	0.24	1.18	1.06E-02	4.90E-02
PAN3-AS1	-0.29	0.82	1.06E-02	NA
SHANK2	0.36	1.28	1.06E-02	4.91E-02
MAD1L1	0.25	1.19	1.06E-02	4.92E-02
RNASE7	0.44	1.36	1.06E-02	4.92E-02
XRCC1	-0.24	0.85	1.07E-02	4.95E-02
ZNF398	-0.29	0.82	1.07E-02	4.95E-02
COMMD4	-0.26	0.84	1.07E-02	4.95E-02
PLAGL2	0.21	1.15	1.07E-02	4.95E-02
CORO2A	0.19	1.14	1.07E-02	4.96E-02
FAR2	-0.35	0.78	1.07E-02	4.96E-02
ITPRIPL2	-0.29	0.82	1.07E-02	4.97E-02
GTF3C3	0.22	1.17	1.08E-02	4.97E-02
ZNF747	-0.44	0.74	1.08E-02	4.98E-02
SLC24A1	-0.29	0.82	1.08E-02	4.99E-02
NSD1	-0.18	0.88	1.08E-02	4.99E-02
TMEM184C	0.21	1.16	1.08E-02	5.00E-02
DENND1B	0.30	1.23	1.09E-02	5.00E-02
NXPH4	-0.44	0.74	1.09E-02	5.01E-02
NME3	-0.42	0.75	1.09E-02	5.02E-02
UEVLD	0.24	1.18	1.09E-02	5.04E-02
AKAP10	0.26	1.20	1.09E-02	5.04E-02
POLR3H	0.26	1.20	1.10E-02	5.04E-02
ROBO1	0.19	1.14	1.10E-02	5.05E-02
CWC27	0.25	1.19	1.10E-02	5.05E-02
NISCH	-0.20	0.87	1.10E-02	5.07E-02
IER3	0.22	1.16	1.10E-02	5.07E-02
MTERF2	0.45	1.37	1.10E-02	5.07E-02
LMTK3	-0.34	0.79	1.10E-02	5.07E-02
PSMB9	-0.47	0.72	1.10E-02	5.07E-02
NUP205	0.16	1.12	1.11E-02	5.07E-02
SYNRG	0.24	1.18	1.11E-02	5.08E-02
AC141586.5	-0.46	0.72	1.11E-02	5.08E-02
ZBTB7C	-0.46	0.73	1.11E-02	5.08E-02
RAB20	-0.45	0.73	1.11E-02	5.09E-02
CDC5L	0.20	1.15	1.11E-02	5.09E-02
SLC35E4	-0.34	0.79	1.11E-02	5.10E-02
AOX1	-0.34	0.79	1.11E-02	5.10E-02
CCDC59	0.28	1.21	1.12E-02	5.11E-02
PLEKHA6	-0.37	0.77	1.12E-02	5.11E-02
C16orf72	0.20	1.15	1.12E-02	5.12E-02
TRPM7	0.24	1.18	1.12E-02	5.12E-02
HERC2P2	0.41	1.33	1.12E-02	5.12E-02
TNRC6C	0.40	1.32	1.12E-02	5.12E-02
C9orf64	-0.30	0.81	1.12E-02	5.12E-02
XPO4	0.21	1.16	1.13E-02	5.14E-02
PIP4K2A	0.27	1.20	1.13E-02	5.14E-02
RHPN2	0.35	1.28	1.13E-02	5.15E-02
MORC2	0.21	1.16	1.13E-02	5.16E-02
AREL1	-0.19	0.87	1.13E-02	5.16E-02
USB1	0.21	1.16	1.13E-02	5.16E-02
WNT4	-0.37	0.78	1.13E-02	NA
TMEM30A	0.22	1.16	1.13E-02	5.17E-02
SAP30	-0.35	0.78	1.14E-02	5.18E-02
HIST1H2AC	0.36	1.28	1.14E-02	5.18E-02
ZSCAN29	0.26	1.20	1.14E-02	5.18E-02
KMT2E-AS1	-0.38	0.77	1.14E-02	NA
MSH2	-0.24	0.85	1.14E-02	5.20E-02
ITGA3	0.18	1.13	1.14E-02	5.20E-02
NR2F6	-0.25	0.84	1.14E-02	5.20E-02
CLPP	-0.21	0.86	1.14E-02	5.20E-02
MRFAP1	0.15	1.11	1.15E-02	5.22E-02
ITPRIPL1	-0.45	0.73	1.15E-02	5.22E-02
NECAP1	0.24	1.18	1.15E-02	5.22E-02
S100A11	-0.14	0.91	1.15E-02	5.23E-02
AKR1C3	-0.46	0.73	1.15E-02	5.23E-02
C3orf58	-0.33	0.80	1.15E-02	5.23E-02
SAMD8	0.25	1.19	1.16E-02	5.25E-02
C12orf10	-0.26	0.84	1.16E-02	5.26E-02
MPHOSPH6	0.25	1.19	1.16E-02	5.26E-02
GPC1	-0.20	0.87	1.16E-02	5.28E-02
GPX7	-0.46	0.73	1.17E-02	5.28E-02
SIK2	0.23	1.17	1.17E-02	5.28E-02
XRCC6BP1	-0.40	0.76	1.17E-02	5.29E-02
SLAIN2	0.19	1.14	1.17E-02	5.29E-02
ALDOC	-0.26	0.83	1.17E-02	5.31E-02
MAP2K7	0.24	1.18	1.17E-02	5.31E-02
NACC2	0.25	1.19	1.18E-02	5.31E-02
FAM195A	-0.36	0.78	1.18E-02	5.32E-02
LONRF1	-0.34	0.79	1.18E-02	5.33E-02
ARMCX1	-0.35	0.79	1.18E-02	5.34E-02
AFG3L2	0.20	1.15	1.18E-02	5.34E-02
CLUAP1	0.43	1.35	3.45E-03	1.59E-02
KIF15	0.41	1.32	3.46E-03	1.59E-02
PAPD7	0.25	1.19	3.46E-03	1.59E-02
PFAS	0.31	1.24	3.47E-03	1.60E-02
ERCC2	0.26	1.20	3.51E-03	1.61E-02
CEP70	0.34	1.27	3.51E-03	1.61E-02
DCLRE1A	0.33	1.26	3.52E-03	1.61E-02
FBXL18	-0.32	0.80	3.52E-03	1.61E-02
RP11-800A3.4	-0.27	0.83	3.52E-03	1.61E-02
HECTD1	0.20	1.15	3.52E-03	1.61E-02
ZNF512B	-0.24	0.85	3.53E-03	1.62E-02
THBS3	-0.36	0.78	3.56E-03	1.63E-02
MLLT6	-0.27	0.83	3.57E-03	1.63E-02
CCDC34	0.40	1.32	3.58E-03	1.64E-02
FANCE	0.29	1.22	3.58E-03	1.64E-02
PLEKHB2	-0.23	0.86	3.59E-03	1.64E-02
CD27-AS1	-0.55	0.68	3.60E-03	1.65E-02
LAS1L	0.24	1.18	3.60E-03	1.65E-02
DSCC1	0.45	1.36	3.60E-03	1.65E-02
NFKB2	0.31	1.24	3.60E-03	1.65E-02
PCBP2	0.23	1.17	3.61E-03	1.65E-02
TSC1	0.24	1.18	3.61E-03	1.65E-02
EXT2	-0.21	0.87	3.61E-03	1.65E-02
ANXA6	0.61	1.52	3.62E-03	1.65E-02
NMT2	0.30	1.23	3.63E-03	1.66E-02
STARD13	-0.32	0.80	3.64E-03	1.66E-02
BRIX1	0.29	1.22	3.64E-03	1.66E-02
RFX2	-0.34	0.79	3.64E-03	1.66E-02
ACBD5	0.26	1.20	3.67E-03	1.67E-02
RRP9	0.26	1.20	3.68E-03	1.67E-02
SRRT	0.23	1.17	3.68E-03	1.68E-02
DHX9	0.24	1.18	3.68E-03	1.68E-02
TUB	0.52	1.43	3.69E-03	1.68E-02
ATP10B	-0.45	0.73	3.70E-03	1.68E-02
LMNB2	0.24	1.18	3.70E-03	1.68E-02
ZFYVE9	0.25	1.19	3.71E-03	1.68E-02
JUND	-0.27	0.83	3.72E-03	1.69E-02
TRIM33	0.23	1.17	3.72E-03	1.69E-02
BCCIP	0.24	1.18	3.73E-03	1.69E-02
ARHGAP22	-0.44	0.74	3.73E-03	1.69E-02
SPC24	0.38	1.30	3.73E-03	1.69E-02
AC013461.1	0.21	1.16	3.73E-03	1.69E-02
LZIC	0.28	1.22	3.74E-03	1.70E-02
SH3TC2	-0.44	0.74	3.74E-03	1.70E-02
LAMB1	0.21	1.16	3.76E-03	1.70E-02
RNF19B	-0.28	0.82	3.76E-03	1.70E-02
EXOSC8	0.29	1.23	3.77E-03	1.71E-02
ELOVL6	0.21	1.16	3.77E-03	1.71E-02
CLSPN	0.36	1.28	3.79E-03	1.71E-02
ISCA1	0.28	1.22	3.79E-03	1.72E-02
HACD3	0.21	1.16	3.80E-03	1.72E-02
RSPRY1	0.24	1.18	3.81E-03	1.72E-02
ATP10D	-0.22	0.86	3.82E-03	1.73E-02
CASC4	0.29	1.22	3.82E-03	1.73E-02
ZBTB39	0.34	1.26	3.83E-03	1.73E-02
GLUD1	0.21	1.15	3.83E-03	1.73E-02
CDK10	-0.24	0.85	3.84E-03	1.73E-02
ETV4	-0.39	0.76	3.84E-03	1.73E-02
GALNT14	0.30	1.23	3.85E-03	1.74E-02
DBNL	-0.21	0.86	3.86E-03	1.74E-02
CEP97	0.28	1.22	3.87E-03	1.74E-02
MAP3K9	-0.28	0.82	3.87E-03	1.75E-02
PSMD4	0.22	1.16	3.89E-03	1.75E-02
ALG13	0.31	1.24	3.90E-03	1.76E-02
MATN3	0.78	1.71	3.91E-03	1.76E-02
IRF5	-0.39	0.76	3.93E-03	1.77E-02
TMEM175	-0.33	0.79	3.94E-03	1.77E-02
TAF11	0.31	1.24	3.95E-03	1.77E-02
API5	0.22	1.16	3.95E-03	1.77E-02
AGTPBP1	0.42	1.33	3.95E-03	1.77E-02
CDC42	0.19	1.14	3.95E-03	1.78E-02
PTCHD4	-1.42	0.37	3.96E-03	NA
THEM6	-0.36	0.78	3.96E-03	1.78E-02
HNRNPAB	0.20	1.15	3.99E-03	1.79E-02
SYT2	-1.74	0.30	4.00E-03	NA
CIART	0.87	1.83	4.01E-03	1.80E-02
IRAK4	-0.31	0.81	4.01E-03	1.80E-02
TAOK1	0.20	1.15	4.01E-03	1.80E-02
KIAA2026	-0.27	0.83	4.02E-03	1.80E-02
HEATR3	0.27	1.21	4.05E-03	1.81E-02
FNIP2	-0.31	0.81	4.05E-03	1.81E-02
RPS6KA6	-0.51	0.70	4.06E-03	1.82E-02
TCERG1	0.22	1.16	4.08E-03	1.83E-02
PKN2	0.21	1.16	4.08E-03	1.83E-02
CHST2	-0.98	0.51	4.10E-03	1.83E-02
SEMA4D	0.38	1.30	4.12E-03	1.84E-02
NBR2	-0.59	0.66	4.12E-03	1.84E-02
TBC1D4	0.25	1.19	4.12E-03	1.84E-02
UAP1L1	0.33	1.26	4.14E-03	1.85E-02
DGKH	0.23	1.17	4.15E-03	1.85E-02

DGKH	0.27	1.20	1.19E-02	5.35E-02	ABI2	0.27	1.21	4.15E-03	1.85E-02
ADPGK	0.22	1.17	1.19E-02	5.35E-02	MAST3	-0.38	0.77	4.15E-03	1.85E-02
RITA1	-0.28	0.83	1.19E-02	5.36E-02	LAMA3	-0.22	0.86	4.18E-03	1.87E-02
ZBTB45	-0.30	0.81	1.19E-02	5.36E-02	C7orf43	-0.34	0.79	4.20E-03	1.87E-02
PARP9	-0.26	0.83	1.19E-02	5.36E-02	CHST11	0.29	1.22	4.20E-03	1.87E-02
PRPF31	0.23	1.17	1.19E-02	5.36E-02	BTBD9	-0.35	0.79	4.21E-03	1.88E-02
HIST1H1C	0.43	1.34	1.19E-02	5.36E-02	MYLIP	0.49	1.40	4.22E-03	1.88E-02
TAF1A	0.37	1.29	1.19E-02	5.36E-02	MICAL1	-0.25	0.84	4.24E-03	1.89E-02
TRIP10	0.16	1.12	1.19E-02	5.36E-02	CYB561D1	-0.31	0.81	4.24E-03	1.89E-02
RERE	0.21	1.16	1.19E-02	5.36E-02	PGLYRP4	-0.52	0.70	4.25E-03	1.89E-02
NAT10	0.17	1.12	1.19E-02	5.36E-02	NFIX	0.23	1.17	4.25E-03	1.89E-02
WDR27	0.33	1.26	1.19E-02	5.36E-02	EIF2AK4	0.20	1.15	4.25E-03	1.89E-02
CTD-2555C10.3	-0.47	0.72	1.19E-02	5.36E-02	HNRNPUL1	0.20	1.15	4.25E-03	1.89E-02
APOBEC3C	-0.19	0.88	1.19E-02	5.37E-02	LARS2	0.23	1.18	4.26E-03	1.89E-02
IFI44L	-0.43	0.74	1.20E-02	5.38E-02	DUSP5	-0.29	0.82	4.26E-03	1.90E-02
ZNF844	-0.46	0.73	1.20E-02	5.38E-02	WRAP53	0.31	1.24	4.29E-03	1.91E-02
ZNF219	-0.26	0.83	1.20E-02	5.40E-02	TXNDC12	0.24	1.18	4.29E-03	1.91E-02
TENM3	0.25	1.19	1.20E-02	5.40E-02	DSC1	-0.38	0.77	4.32E-03	1.92E-02
KPNA5	0.44	1.35	1.20E-02	5.41E-02	SERINC2	-0.20	0.87	4.32E-03	1.92E-02
FERMT2	0.32	1.25	1.21E-02	5.41E-02	FAM83G	-0.20	0.87	4.33E-03	1.92E-02
ZNF302	0.39	1.31	1.21E-02	5.41E-02	PRODH	-0.33	0.79	4.33E-03	1.92E-02
MARCO	-0.41	0.75	1.21E-02	5.42E-02	CROCC	0.27	1.21	4.34E-03	1.92E-02
KLHL9	-0.26	0.83	1.21E-02	5.43E-02	PAK6	0.26	1.20	4.35E-03	1.93E-02
HCCS	0.23	1.17	1.21E-02	5.43E-02	CUL5	0.24	1.18	4.35E-03	1.93E-02
AHR	0.26	1.19	1.22E-02	5.44E-02	SLC50A1	-0.24	0.85	4.36E-03	1.93E-02
TRAF3IP3	0.43	1.35	1.22E-02	5.45E-02	HSDL1	0.30	1.23	4.36E-03	1.93E-02
SERTAD3	-0.33	0.80	1.22E-02	5.46E-02	MBD4	0.27	1.21	4.36E-03	1.93E-02
MLH1	-0.22	0.86	1.22E-02	5.46E-02	CENPK	0.35	1.28	4.38E-03	1.94E-02
ZNF213-AS1	-0.45	0.73	1.22E-02	5.46E-02	ZNF83	-0.28	0.83	4.39E-03	1.94E-02
CKMT1B	-0.28	0.83	1.22E-02	5.47E-02	PPP1R15B	0.21	1.16	4.40E-03	1.95E-02
GPX8	-0.29	0.82	1.23E-02	5.48E-02	RPAP1	-0.25	0.84	4.40E-03	1.95E-02
ARHGAP11A	0.21	1.15	1.23E-02	5.48E-02	DOCK4	0.35	1.28	4.40E-03	1.95E-02
SOCS7	0.24	1.18	1.23E-02	5.48E-02	SART3	0.24	1.18	4.40E-03	1.95E-02
RAB11B	-0.23	0.85	1.23E-02	5.49E-02	SFR1	0.50	1.42	4.42E-03	1.95E-02
GNG4	-0.47	0.72	1.23E-02	5.49E-02	CYSRT1	-0.49	0.71	4.43E-03	1.96E-02
USP51	-0.39	0.76	1.23E-02	NA	TRANK1	-0.33	0.80	4.43E-03	1.96E-02
C9orf91	-0.31	0.80	1.23E-02	5.50E-02	RASAL2	-0.22	0.86	4.44E-03	1.96E-02
UBQLN2	-0.21	0.87	1.24E-02	5.52E-02	ADAM9	0.19	1.14	4.45E-03	1.96E-02
CUL2	0.22	1.17	1.24E-02	5.52E-02	CCNE1	-0.45	0.73	4.45E-03	1.96E-02
RPL13	-0.19	0.88	1.24E-02	5.53E-02	SH3BP4	-0.27	0.83	4.46E-03	1.97E-02
IRF6	0.14	1.10	1.24E-02	5.53E-02	XRCC3	0.31	1.24	4.48E-03	1.97E-02
MAPK8IP2	-0.46	0.73	1.25E-02	5.55E-02	CRABP2	-0.27	0.83	4.48E-03	1.97E-02
JADE1	-0.33	0.79	1.25E-02	5.56E-02	VTA1	0.23	1.17	4.48E-03	1.97E-02
SCARA3	-0.19	0.87	1.25E-02	5.56E-02	TMEM138	-0.28	0.83	4.49E-03	1.98E-02
IL17C	0.27	1.20	1.25E-02	NA	BMS1	0.21	1.16	4.50E-03	1.98E-02
NPM1P43	-0.47	0.72	1.25E-02	5.58E-02	VPS36	-0.23	0.85	4.50E-03	1.98E-02
LBR	0.20	1.15	1.25E-02	5.58E-02	PARK7	0.21	1.15	4.51E-03	1.99E-02
SLC52A3	-0.40	0.76	1.26E-02	5.58E-02	CMTM3	-0.31	0.81	4.52E-03	1.99E-02
MANEAL	-0.46	0.73	1.26E-02	5.61E-02	HOXA10	0.62	1.53	4.53E-03	1.99E-02
AOC2	0.46	1.38	1.26E-02	5.61E-02	KNTC1	-0.29	0.82	4.55E-03	2.00E-02
PTGER4	0.45	1.37	1.26E-02	5.61E-02	SRP9	0.21	1.16	4.55E-03	2.00E-02
NGEF	-0.46	0.73	1.26E-02	5.61E-02	ZSWIM4	0.44	1.35	4.57E-03	2.01E-02
COPB1	0.18	1.13	1.26E-02	5.61E-02	IL27RA	0.42	1.34	4.58E-03	2.01E-02
ESPNL	-0.46	0.73	1.27E-02	5.63E-02	MYO6	-0.21	0.87	4.58E-03	2.01E-02
WDR20	0.27	1.20	1.27E-02	5.63E-02	02-mar	-0.33	0.80	4.63E-03	2.03E-02
PRELID3B	0.20	1.15	1.27E-02	5.63E-02	ARTN	-0.23	0.85	4.64E-03	2.04E-02
HLTF	0.27	1.21	1.27E-02	5.63E-02	CSDE1	0.18	1.14	4.67E-03	2.05E-02
RFC1	0.21	1.16	1.27E-02	5.64E-02	UBAP2	-0.23	0.85	4.68E-03	2.05E-02
IRF2BP2	-0.18	0.88	1.27E-02	5.64E-02	HSD11B2	0.51	1.42	4.68E-03	2.05E-02
DPH2	0.26	1.20	1.28E-02	5.65E-02	ARL6IP5	0.21	1.16	4.69E-03	2.05E-02
KIAA1109	0.24	1.18	1.28E-02	5.66E-02	GPR132	-0.87	0.55	4.69E-03	2.05E-02
CHAF1A	-0.21	0.86	1.28E-02	5.66E-02	CUL4B	0.23	1.17	4.69E-03	2.06E-02
ZNF143	0.28	1.21	1.28E-02	5.67E-02	HOPX	-0.31	0.81	4.70E-03	2.06E-02
ADCY3	0.20	1.15	1.28E-02	5.67E-02	SNTB2	0.26	1.19	4.70E-03	2.06E-02
RRN3	0.23	1.17	1.28E-02	5.68E-02	USP30	-0.39	0.76	4.72E-03	2.06E-02
IGFBP3	-0.46	0.73	1.28E-02	5.68E-02	SCYL1	0.23	1.17	4.73E-03	2.07E-02
CNNM1	-0.46	0.73	1.29E-02	5.68E-02	SERPINB1	0.27	1.20	4.73E-03	2.07E-02
KIF18A	0.30	1.23	1.29E-02	5.69E-02	PHLDB2	-0.22	0.86	4.73E-03	2.07E-02
RNF207	0.45	1.36	1.29E-02	5.69E-02	TRAM1	-0.20	0.87	4.74E-03	2.07E-02
LSM2	-0.22	0.86	1.29E-02	5.70E-02	ALG1	0.31	1.24	4.74E-03	2.07E-02
SNHG12	-0.33	0.80	1.29E-02	5.71E-02	IRF2BP2	-0.21	0.86	4.74E-03	2.07E-02
WTIP	0.36	1.28	1.29E-02	5.71E-02	APBB1	0.64	1.56	4.75E-03	2.08E-02
KBTBD2	0.24	1.18	1.29E-02	5.71E-02	MAMDC2	0.69	1.62	4.76E-03	2.08E-02
GTF2E1	0.30	1.23	1.30E-02	5.72E-02	IER2	0.24	1.18	4.78E-03	2.08E-02
NFATC1	-0.43	0.74	1.30E-02	5.72E-02	PCOLCE2	1.15	2.22	4.78E-03	2.08E-02
CYP2D7	-0.43	0.74	1.30E-02	5.74E-02	MINPP1	0.30	1.23	4.79E-03	2.09E-02
LMNTD2	-0.39	0.76	1.30E-02	NA	REEP2	-0.72	0.61	4.80E-03	2.09E-02
SERINC3	0.17	1.12	1.30E-02	5.75E-02	COL4A2	0.20	1.15	4.80E-03	2.09E-02
MBD6	0.24	1.18	1.31E-02	5.75E-02	C12orf49	0.24	1.18	4.85E-03	2.11E-02
C5orf24	0.27	1.21	1.31E-02	5.75E-02	POLR1E	0.25	1.19	4.85E-03	2.11E-02
ZFYVE21	-0.19	0.88	1.31E-02	5.75E-02	FAM72C	0.91	1.88	4.86E-03	2.11E-02
EARS2	0.21	1.16	1.31E-02	5.75E-02	SDAD1	0.25	1.19	4.86E-03	2.12E-02
ZNF213	-0.29	0.82	1.31E-02	5.76E-02	SBNO2	0.22	1.16	4.89E-03	2.13E-02
PP7080	-0.41	0.75	1.31E-02	5.76E-02	01-mar	0.62	1.53	4.89E-03	2.13E-02
TMOD3	0.16	1.12	1.31E-02	5.76E-02	PIGK	0.26	1.20	4.89E-03	2.13E-02
LSM14A	0.16	1.12	1.31E-02	5.76E-02	LEMD3	0.24	1.18	4.90E-03	2.13E-02
SLC16A7	-0.46	0.73	1.31E-02	5.76E-02	DCTN4	0.22	1.17	4.90E-03	2.13E-02
LHFP	0.31	1.24	1.31E-02	5.76E-02	TMEM30B	0.25	1.19	4.90E-03	2.13E-02
MTOR	0.17	1.12	1.31E-02	5.77E-02	SWAP70	0.25	1.19	4.91E-03	2.13E-02
RBBP5	-0.27	0.83	1.32E-02	5.77E-02	TRMT1L	0.26	1.19	4.92E-03	2.13E-02

**CHD1L**	0.20	1.15	1.32E-02	5.79E-02
**SLC22A3**	-0.39	0.76	1.32E-02	5.80E-02
**FAM43A**	-0.45	0.73	1.33E-02	5.81E-02
**KDM5B**	-0.17	0.89	1.33E-02	5.82E-02
**SERTAD4**	0.32	1.25	1.33E-02	5.83E-02
**RP11-864N7.2**	-0.35	0.79	1.33E-02	5.83E-02
**RP5-1056L3.3**	-0.39	0.76	1.34E-02	5.85E-02
**ENTPD2**	-0.38	0.77	1.34E-02	5.86E-02
**RIN2**	-0.22	0.86	1.34E-02	5.86E-02
**LIMD2**	-0.45	0.73	1.34E-02	5.86E-02
**MIIP**	0.28	1.21	1.34E-02	5.87E-02
**GOLPH3**	0.19	1.14	1.34E-02	5.87E-02
**KLK7**	-0.14	0.90	1.34E-02	5.87E-02
**RP3-395M20.3**	-0.31	0.80	1.35E-02	NA
**IL6ST**	0.23	1.17	1.35E-02	5.90E-02
**USP31**	0.23	1.17	1.35E-02	5.90E-02
**DTX2**	0.25	1.19	1.36E-02	5.93E-02
**ZNF75A**	0.34	1.26	1.36E-02	5.93E-02
**MIR9-3HG**	-0.46	0.73	1.36E-02	5.93E-02
**ASPG**	-0.44	0.74	1.36E-02	5.94E-02
**GPALPP1**	0.28	1.22	1.36E-02	5.95E-02
**CLASP1**	0.17	1.13	1.37E-02	5.95E-02
**DUSP6**	0.18	1.14	1.37E-02	5.97E-02
**ARFGAP1**	0.23	1.17	1.38E-02	5.99E-02
**SCCPDH**	-0.34	0.79	1.38E-02	6.00E-02
**GALNT18**	0.23	1.17	1.38E-02	6.01E-02
**SEC31A**	0.15	1.11	1.39E-02	6.03E-02
**PLAGL1**	0.25	1.19	1.39E-02	6.04E-02
**TUBAL3**	-0.43	0.74	1.39E-02	6.04E-02
**NR2F2**	-0.21	0.87	1.39E-02	6.05E-02
**CD3EAP**	-0.36	0.78	1.39E-02	6.05E-02
**KIFAP3**	-0.25	0.84	1.39E-02	6.05E-02
**ZNF711**	-0.46	0.73	1.39E-02	6.05E-02
**SEC16A**	0.16	1.12	1.39E-02	6.05E-02
**COBLL1**	-0.19	0.87	1.40E-02	6.06E-02
**TNS4**	0.18	1.13	1.40E-02	6.06E-02
**FAM109B**	-0.32	0.80	1.40E-02	6.06E-02
**CTPS1**	0.16	1.12	1.40E-02	6.06E-02
**SNHG25**	-0.46	0.73	1.40E-02	6.06E-02
**KCTD5**	0.22	1.16	1.40E-02	6.08E-02
**MEGF9**	-0.25	0.84	1.41E-02	6.09E-02
**IFI16**	-0.19	0.88	1.41E-02	6.10E-02
**RCOR3**	-0.28	0.83	1.41E-02	6.11E-02
**RPS3AP47**	-0.45	0.73	1.41E-02	6.11E-02
**CCP110**	-0.26	0.84	1.41E-02	6.12E-02
**CCDC82**	0.33	1.26	1.41E-02	6.12E-02
**FOXC1**	-0.22	0.86	1.42E-02	6.12E-02
**WDR44**	0.24	1.18	1.42E-02	6.12E-02
**RANBP10**	0.23	1.17	1.42E-02	6.12E-02
**EME1**	0.35	1.28	1.42E-02	6.12E-02
**LINC01089**	-0.45	0.73	1.42E-02	6.14E-02
**NABP2**	-0.22	0.86	1.42E-02	6.14E-02
**TIAL1**	0.17	1.13	1.43E-02	6.15E-02
**XPOT**	-0.17	0.89	1.43E-02	6.16E-02
**DDX24**	0.15	1.11	1.43E-02	6.17E-02
**ATXN7L3B**	-0.20	0.87	1.44E-02	6.19E-02
**GGPS1**	0.24	1.18	1.44E-02	6.20E-02
**CNTD2**	-0.34	0.79	1.44E-02	NA
**FAM60A**	0.20	1.15	1.44E-02	6.20E-02
**USMG5P1**	-0.45	0.73	1.44E-02	6.20E-02
**GADD45B**	-0.30	0.81	1.44E-02	6.20E-02
**SPAG7**	0.21	1.16	1.44E-02	6.20E-02
**USP40**	0.20	1.15	1.44E-02	6.20E-02
**FZD1**	-0.32	0.80	1.44E-02	6.21E-02
**EXOC2**	0.21	1.16	1.45E-02	6.22E-02
**CASP10**	-0.32	0.80	1.45E-02	6.22E-02
**RASIP1**	-0.25	0.84	1.45E-02	6.22E-02
**SNX25**	0.29	1.22	1.45E-02	6.23E-02
**JMJD8**	-0.29	0.82	1.46E-02	6.25E-02
**PDE4B**	-0.44	0.74	1.46E-02	6.25E-02
**RBM18**	0.26	1.20	1.46E-02	6.25E-02
**TMA16**	0.24	1.18	1.46E-02	6.26E-02
**RP13-942N8.1**	-0.34	0.79	1.46E-02	NA
**MAP2K6**	-0.43	0.74	1.46E-02	6.26E-02
**DSCAM**	0.30	1.23	1.46E-02	6.26E-02
**SLC7A11**	-0.25	0.84	1.46E-02	6.27E-02
**CDCA7**	0.19	1.14	1.47E-02	6.28E-02
**DIS3L**	-0.29	0.82	1.47E-02	6.29E-02
**SLC16A3**	-0.24	0.85	1.47E-02	6.30E-02
**PLB1**	-0.33	0.79	1.47E-02	6.31E-02
**C16orf74**	0.23	1.17	1.48E-02	6.34E-02
**GAS6**	0.19	1.14	1.48E-02	6.34E-02
**RALY**	0.17	1.12	1.48E-02	6.35E-02
**ARAP2**	0.25	1.19	1.49E-02	6.35E-02
**TGFBRAP1**	0.20	1.15	1.49E-02	6.35E-02
**ZNFX1**	0.20	1.15	1.49E-02	6.36E-02
**TMEM64**	0.25	1.19	1.49E-02	6.36E-02
**PRSS22**	0.31	1.24	1.49E-02	6.37E-02
**ASCL2**	-0.40	0.76	1.49E-02	6.37E-02
**AAGAB**	0.19	1.14	1.50E-02	6.40E-02
**IFI27**	-0.24	0.84	4.92E-03	2.14E-02
**ESRP1**	0.19	1.14	4.94E-03	2.14E-02
**ARMC8**	0.26	1.19	4.95E-03	2.14E-02
**TIAF1**	-0.65	0.64	4.95E-03	2.14E-02
**PNPLA8**	-0.28	0.82	4.95E-03	2.14E-02
**ZBED2**	-0.25	0.84	4.96E-03	2.15E-02
**CPNE2**	0.37	1.29	4.96E-03	2.15E-02
**USP16**	0.25	1.19	4.97E-03	2.15E-02
**UBLCP1**	0.25	1.19	4.99E-03	2.16E-02
**SLC30A4**	-0.41	0.75	4.99E-03	2.16E-02
**SMG5**	-0.20	0.87	4.99E-03	2.16E-02
**MMS22L**	0.30	1.23	4.99E-03	2.16E-02
**EIF3B**	0.18	1.14	4.99E-03	2.16E-02
**TCEAL4**	-0.23	0.86	4.99E-03	2.16E-02
**PHLDB1**	0.26	1.20	5.00E-03	2.16E-02
**RAB11FIP1**	0.24	1.18	5.01E-03	2.16E-02
**KCNK7**	0.66	1.58	5.01E-03	2.16E-02
**RBM26**	0.22	1.17	5.01E-03	2.16E-02
**AAAS**	0.23	1.17	5.02E-03	2.17E-02
**DOCK6**	-0.27	0.83	5.03E-03	2.17E-02
**TRIQK**	-0.47	0.72	5.04E-03	2.17E-02
**PRPF31**	0.24	1.18	5.05E-03	2.17E-02
**WASF3**	0.33	1.26	5.07E-03	2.18E-02
**PPP1R26**	0.26	1.19	5.09E-03	2.19E-02
**NUDT5**	0.22	1.17	5.10E-03	2.19E-02
**FAM46C**	1.83	3.55	5.10E-03	NA
**GTF2A1**	0.25	1.19	5.10E-03	2.20E-02
**RP3-510D11.2**	-0.79	0.58	5.10E-03	2.20E-02
**EDRF1**	0.30	1.23	5.11E-03	2.20E-02
**KCTD3**	0.21	1.16	5.11E-03	2.20E-02
**TNPO2**	-0.21	0.87	5.12E-03	2.20E-02
**HLTF**	0.27	1.20	5.12E-03	2.20E-02
**LAMB3**	-0.49	0.71	5.14E-03	2.21E-02
**CDC37L1**	0.29	1.22	5.15E-03	2.21E-02
**TMEM150A**	-0.46	0.73	5.16E-03	2.22E-02
**DBNDD1**	-0.38	0.77	5.18E-03	2.22E-02
**CEP120**	0.31	1.24	5.20E-03	2.23E-02
**IRAK2**	0.82	1.77	5.20E-03	2.23E-02
**SMARCC2**	-0.23	0.85	5.21E-03	2.23E-02
**LINC00342**	-0.53	0.69	5.21E-03	2.23E-02
**RASD2**	-1.15	0.45	5.21E-03	2.23E-02
**KIF7**	0.41	1.33	5.22E-03	2.23E-02
**CCNH**	0.28	1.21	5.23E-03	2.24E-02
**NEK6**	0.26	1.20	5.24E-03	2.24E-02
**SLC20A2**	-0.20	0.87	5.24E-03	2.24E-02
**ASIC1**	-0.37	0.77	5.25E-03	2.25E-02
**YIPF2**	-0.27	0.83	5.27E-03	2.25E-02
**SNHG18**	-0.43	0.74	5.28E-03	2.25E-02
**CBLL1**	0.26	1.20	5.30E-03	2.26E-02
**THOC5**	0.25	1.19	5.30E-03	2.26E-02
**RRNAD1**	-0.33	0.79	5.30E-03	2.26E-02
**HPSE**	0.37	1.29	5.31E-03	2.27E-02
**RAP1A**	0.23	1.17	5.31E-03	2.27E-02
**LMO1**	1.74	3.34	5.33E-03	NA
**PRDM2**	0.22	1.17	5.36E-03	2.29E-02
**UST**	0.36	1.29	5.38E-03	2.30E-02
**PDS5B**	0.27	1.21	5.39E-03	2.30E-02
**MRPL28**	0.22	1.17	5.41E-03	2.30E-02
**DGCR6**	-0.48	0.71	5.41E-03	2.31E-02
**GAPDH**	0.19	1.14	5.42E-03	2.31E-02
**TMEM129**	-0.26	0.83	5.43E-03	2.31E-02
**MIR210HG**	-0.74	0.60	5.44E-03	2.32E-02
**METTL7A**	-0.25	0.84	5.45E-03	2.32E-02
**TMEM181**	0.22	1.17	5.47E-03	2.32E-02
**CTD-3247F14.2**	-0.97	0.51	5.47E-03	2.33E-02
**CARD6**	-0.38	0.77	5.48E-03	2.33E-02
**DNAJB14**	0.23	1.17	5.48E-03	2.33E-02
**GNB4**	0.24	1.18	5.48E-03	2.33E-02
**CLCN6**	-0.30	0.81	5.48E-03	2.33E-02
**GPR39**	0.60	1.52	5.53E-03	2.35E-02
**SNX17**	0.21	1.16	5.55E-03	2.36E-02
**RNF123**	-0.25	0.84	5.56E-03	2.36E-02
**SLC39A9**	0.21	1.15	5.56E-03	2.36E-02
**AHNAK2**	-0.18	0.88	5.58E-03	2.36E-02
**ZNF92**	0.42	1.34	5.58E-03	2.36E-02
**FOXJ3**	0.22	1.17	5.59E-03	2.37E-02
**RBBP4**	0.20	1.15	5.60E-03	2.37E-02
**ADTRP**	0.36	1.28	5.61E-03	2.38E-02
**CYB561A3**	-0.30	0.81	5.62E-03	2.38E-02
**MRPL15**	0.24	1.18	5.65E-03	2.39E-02
**RRP1**	0.24	1.18	5.68E-03	2.40E-02
**HIP1**	0.31	1.24	5.70E-03	2.41E-02
**VEZT**	-0.21	0.86	5.72E-03	2.42E-02
**RTN4R**	0.35	1.28	5.72E-03	2.42E-02
**KIF20A**	0.56	1.48	5.75E-03	2.43E-02
**PSMG1**	0.26	1.20	5.78E-03	2.44E-02
**TLE3**	0.26	1.20	5.79E-03	2.44E-02
**HYLS1**	0.41	1.33	5.81E-03	2.45E-02
**DFNB31**	-0.41	0.75	5.81E-03	2.45E-02
**SPRY4**	-0.40	0.76	5.81E-03	2.45E-02

ABCC4	0.29	1.22	1.50E-02	6.40E-02
ATP5SL	0.22	1.17	1.51E-02	6.44E-02
PIM1	-0.20	0.87	1.51E-02	6.44E-02
PNPLA3	-0.45	0.73	1.51E-02	6.46E-02
AKAP9	0.25	1.19	1.52E-02	6.46E-02
PGS1	0.33	1.26	1.52E-02	6.46E-02
TMX4	-0.28	0.83	1.52E-02	6.47E-02
HOMER1	0.26	1.20	1.52E-02	6.47E-02
MACF1	0.22	1.16	1.52E-02	6.47E-02
ZNF740	0.24	1.18	1.52E-02	6.47E-02
ITM2A	-0.32	0.80	1.52E-02	6.47E-02
LRRC42	0.22	1.16	1.52E-02	6.47E-02
SRSF1	0.15	1.11	1.52E-02	6.47E-02
CREBZF	-0.25	0.84	1.52E-02	6.47E-02
PDLIM5	0.18	1.13	1.52E-02	6.48E-02
BAZ2A	0.15	1.11	1.53E-02	6.48E-02
TTC9C	-0.32	0.80	1.53E-02	6.48E-02
ZNF544	0.26	1.20	1.53E-02	6.48E-02
NOP14-AS1	0.28	1.21	1.53E-02	6.49E-02
BCL11A	-0.41	0.75	1.53E-02	6.50E-02
RAMP1	-0.44	0.73	1.53E-02	6.50E-02
SLC10A6	-0.36	0.78	1.54E-02	6.51E-02
NKD1	-0.43	0.74	1.54E-02	6.51E-02
COG3	0.21	1.16	1.54E-02	6.51E-02
RAD51D	-0.35	0.79	1.54E-02	6.53E-02
RAN	0.14	1.10	1.54E-02	6.53E-02
NFKB1	0.18	1.13	1.54E-02	6.53E-02
ECD	0.21	1.15	1.54E-02	6.53E-02
ARHGAP21	0.19	1.14	1.54E-02	6.53E-02
SPTSSA	0.22	1.16	1.55E-02	6.54E-02
UQCRC1	-0.17	0.89	1.55E-02	6.54E-02
NMD3	0.20	1.15	1.55E-02	6.54E-02
SLC30A9	0.23	1.17	1.55E-02	6.54E-02
C17orf96	-0.31	0.81	1.55E-02	6.54E-02
PRKD3	0.24	1.18	1.55E-02	6.55E-02
TIAM1	0.20	1.15	1.56E-02	6.58E-02
CDKAL1	0.30	1.23	1.56E-02	6.59E-02
DSCC1	-0.34	0.79	1.56E-02	6.59E-02
MED26	0.36	1.28	1.56E-02	6.60E-02
KCTD10	0.17	1.13	1.57E-02	6.62E-02
KIAA0232	-0.22	0.86	1.57E-02	6.63E-02
NUDT4	0.26	1.20	1.58E-02	6.64E-02
LRRC58	0.23	1.17	1.58E-02	6.66E-02
SV2A	-0.37	0.77	1.58E-02	NA
GIGYF2	0.19	1.14	1.58E-02	6.67E-02
LTBP4	-0.24	0.85	1.59E-02	6.67E-02
PDS5B	0.25	1.19	1.59E-02	6.68E-02
FOLR3	-0.33	0.80	1.59E-02	NA
RASL10B	-0.34	0.79	1.59E-02	NA
PEX12	-0.39	0.76	1.60E-02	6.72E-02
TRMT10A	0.35	1.27	1.60E-02	6.72E-02
UTP15	0.23	1.17	1.60E-02	6.73E-02
FIP1L1	0.22	1.17	1.60E-02	6.74E-02
PWWP2A	-0.28	0.82	1.60E-02	6.74E-02
TMEM216	-0.37	0.77	1.61E-02	6.74E-02
EDN1	-0.22	0.86	1.61E-02	6.74E-02
BFAR	0.18	1.13	1.61E-02	6.75E-02
HLA-G	-0.38	0.77	1.61E-02	6.75E-02
RBBP8	-0.20	0.87	1.61E-02	6.77E-02
RP11-115C21.2	-0.43	0.74	1.62E-02	6.77E-02
UCKL1	0.25	1.19	1.62E-02	6.77E-02
ZCCHC4	0.32	1.24	1.62E-02	6.77E-02
TPGS2	-0.17	0.89	1.62E-02	6.79E-02
CXCR1	-0.38	0.77	1.62E-02	NA
GSDMB	0.45	1.36	1.62E-02	6.79E-02
ORAI3	-0.45	0.73	1.62E-02	6.80E-02
POMGNT1	0.20	1.14	1.63E-02	6.81E-02
EXOG	-0.39	0.76	1.63E-02	6.82E-02
SHQ1	0.26	1.20	1.63E-02	6.82E-02
ZC3H4	-0.23	0.85	1.63E-02	6.82E-02
SSBP4	-0.25	0.84	1.64E-02	6.84E-02
SF3B3	-0.14	0.91	1.64E-02	6.86E-02
BCL2L2	0.22	1.16	1.64E-02	6.86E-02
C12orf66	-0.32	0.80	1.65E-02	6.88E-02
ZBTB8A	-0.44	0.74	1.65E-02	6.88E-02
BRMS1	0.20	1.15	1.65E-02	6.89E-02
ARID1B	0.20	1.15	1.65E-02	6.89E-02
SP100	0.21	1.16	1.65E-02	6.89E-02
UTP3	0.22	1.17	1.65E-02	6.89E-02
NAT6	-0.32	0.80	1.66E-02	6.91E-02
TRAF7	0.18	1.13	1.66E-02	6.93E-02
AHCYL2	0.20	1.15	1.67E-02	6.94E-02
TMEM147-AS1	-0.36	0.78	1.67E-02	6.95E-02
TEAD2	-0.34	0.79	1.67E-02	6.95E-02
SYT12	-0.41	0.75	1.67E-02	6.96E-02
NCF1	-0.38	0.77	1.67E-02	NA
TRAPPC8	0.22	1.16	1.67E-02	6.97E-02
CD55	0.25	1.19	1.68E-02	6.97E-02
SEMA4C	-0.33	0.80	1.68E-02	6.98E-02
NUDCD1	0.22	1.16	1.68E-02	6.99E-02
MIA3	0.22	1.17	5.82E-03	2.45E-02
SMAD1	0.34	1.26	5.82E-03	2.45E-02
RP11-303E16.2	0.54	1.46	5.82E-03	2.45E-02
SNRPC	0.22	1.16	5.85E-03	2.46E-02
RPS6KB2	-0.23	0.85	5.85E-03	2.47E-02
SLC7A2	0.64	1.56	5.87E-03	2.47E-02
BAP1	0.20	1.15	5.87E-03	2.47E-02
COA7	0.24	1.18	5.88E-03	2.47E-02
DDX6	0.19	1.14	5.89E-03	2.48E-02
SH3BGRL2	-0.39	0.76	5.90E-03	2.48E-02
CCM2	0.21	1.16	5.90E-03	2.48E-02
DUBR	-0.42	0.75	5.91E-03	2.48E-02
PKIA	0.43	1.35	5.91E-03	2.48E-02
ADAP2	-0.74	0.60	5.91E-03	2.48E-02
COX5A	0.21	1.15	5.92E-03	2.49E-02
SAP130	-0.25	0.84	5.93E-03	2.49E-02
CHCHD3	0.24	1.18	5.94E-03	2.49E-02
SUMF2	-0.20	0.87	5.95E-03	2.50E-02
EXOC2	0.22	1.17	5.95E-03	2.50E-02
CEP63	0.29	1.23	5.95E-03	2.50E-02
MAGED2	-0.22	0.86	5.96E-03	2.50E-02
GUCD1	0.21	1.16	5.96E-03	2.50E-02
FAAH	0.31	1.24	5.99E-03	2.51E-02
PKN1	0.41	1.33	6.01E-03	2.52E-02
TIGD1	0.90	1.87	6.01E-03	2.52E-02
PCDHGB5	-0.87	0.55	6.02E-03	2.52E-02
LGALS9B	-0.54	0.69	6.02E-03	2.52E-02
COL9A3	0.62	1.54	6.05E-03	2.53E-02
CXCL14	-0.68	0.62	6.07E-03	2.54E-02
MOB4	0.29	1.22	6.07E-03	2.54E-02
CNKSR1	-0.26	0.84	6.08E-03	2.54E-02
COQ2	0.30	1.23	6.08E-03	2.54E-02
LRRC75A-AS1	-0.18	0.88	6.09E-03	2.54E-02
TNS1	0.57	1.48	6.09E-03	2.54E-02
TNK2	-0.20	0.87	6.11E-03	2.55E-02
SKP2	-0.21	0.86	6.14E-03	2.56E-02
PTPDC1	0.33	1.26	6.15E-03	2.56E-02
MBNL3	-0.32	0.80	6.15E-03	2.57E-02
ZNF491	-1.07	0.48	6.17E-03	2.57E-02
SKA2	0.21	1.15	6.18E-03	2.58E-02
C1orf122	-0.27	0.83	6.19E-03	2.58E-02
ELOVL7	-0.27	0.83	6.20E-03	2.58E-02
UTP14A	0.24	1.18	6.21E-03	2.58E-02
PRKAR1B	0.29	1.22	6.21E-03	2.58E-02
NOP14-AS1	-0.34	0.79	6.22E-03	2.59E-02
TPRG1L	0.23	1.17	6.22E-03	2.59E-02
FAM208B	0.21	1.16	6.23E-03	2.59E-02
SCAMP4	-0.26	0.84	6.23E-03	2.59E-02
CCDC88A	0.30	1.23	6.26E-03	2.60E-02
PLD5	-0.35	0.78	6.26E-03	2.60E-02
DHX33	0.22	1.17	6.27E-03	2.60E-02
RP11-20I20.4	1.27	2.42	6.27E-03	2.60E-02
KLHL18	-0.24	0.85	6.29E-03	2.61E-02
LYRM5	-0.42	0.75	6.31E-03	2.62E-02
SYDE1	0.44	1.35	6.32E-03	2.62E-02
HCCS	0.24	1.18	6.34E-03	2.63E-02
OTUD6B	0.33	1.26	6.34E-03	2.63E-02
MLF2	-0.19	0.88	6.35E-03	2.63E-02
CDK6	-0.20	0.87	6.39E-03	2.65E-02
ZNF687	-0.25	0.84	6.43E-03	2.66E-02
RASIP1	0.31	1.24	6.44E-03	2.67E-02
RRM2	0.58	1.49	6.45E-03	2.67E-02
L3MBTL1	-0.44	0.74	6.45E-03	2.67E-02
RP11-58O9.2	-0.26	0.84	6.46E-03	2.67E-02
OXLD1	-0.33	0.79	6.47E-03	2.68E-02
MED20	-0.25	0.84	6.52E-03	2.69E-02
PAK1IP1	0.28	1.21	6.53E-03	2.70E-02
METAP1D	0.33	1.26	6.54E-03	2.70E-02
TCOF1	0.26	1.20	6.56E-03	2.71E-02
PHAX	0.23	1.18	6.60E-03	2.72E-02
SCO1	0.23	1.17	6.61E-03	2.73E-02
ZFR	0.20	1.15	6.61E-03	2.73E-02
KHK	0.63	1.55	6.62E-03	2.73E-02
PRELID2	0.68	1.60	6.65E-03	2.74E-02
PRDM10	-0.32	0.80	6.66E-03	2.75E-02
MAP3K13	-0.30	0.81	6.68E-03	2.75E-02
RPS4Y1	0.18	1.13	6.70E-03	2.76E-02
POLR2M	0.34	1.27	6.74E-03	2.78E-02
ZNF746	-0.28	0.83	6.76E-03	2.78E-02
TCEA1	-0.23	0.85	6.76E-03	2.78E-02
SORL1	0.22	1.16	6.78E-03	2.79E-02
PAF1	0.23	1.17	6.80E-03	2.80E-02
PARVA	-0.23	0.85	6.82E-03	2.81E-02
AL627309.1	-0.42	0.75	6.83E-03	2.81E-02
PSMD13	0.20	1.15	6.84E-03	2.81E-02
SELT	0.20	1.15	6.85E-03	2.81E-02
NSMCE1	0.25	1.19	6.89E-03	2.83E-02
SAT2	-0.27	0.83	6.89E-03	2.83E-02
BORCS8	-0.46	0.73	6.90E-03	2.83E-02
TMEM209	0.29	1.22	6.92E-03	2.84E-02

UPK1A	-0.32	0.80	1.68E-02	NA	PARP1	0.20	1.15	6.92E-03	2.84E-02
JKAMP	0.24	1.18	1.68E-02	6.99E-02	ZDHHC9	-0.22	0.86	6.97E-03	2.86E-02
CASP4	0.20	1.15	1.68E-02	6.99E-02	RAD51	0.31	1.24	6.99E-03	2.87E-02
SPAG1	0.30	1.23	1.69E-02	7.02E-02	C2orf68	-0.32	0.80	7.09E-03	2.90E-02
COL8A1	-0.42	0.75	1.69E-02	7.03E-02	SOCS3	0.80	1.74	7.09E-03	2.91E-02
ARAF	0.20	1.15	1.70E-02	7.06E-02	S100A9	-0.22	0.86	7.10E-03	2.91E-02
ATG12	0.25	1.19	1.70E-02	7.06E-02	BHLHE40	-0.19	0.87	7.10E-03	2.91E-02
SPOPL	0.29	1.22	1.70E-02	7.06E-02	MN1	0.23	1.17	7.12E-03	2.91E-02
VIMP	0.22	1.16	1.70E-02	7.06E-02	ING4	-0.34	0.79	7.12E-03	2.92E-02
BORCS6	-0.34	0.79	1.70E-02	7.06E-02	FRAT2	0.36	1.28	7.14E-03	2.92E-02
DEPDC5	-0.29	0.82	1.71E-02	7.07E-02	KCTD11	-0.28	0.83	7.16E-03	2.93E-02
PDXDC2P	0.41	1.33	1.71E-02	7.08E-02	FBXO5	0.36	1.28	7.17E-03	2.93E-02
METAP1	0.17	1.13	1.71E-02	7.10E-02	TVP23B	0.26	1.20	7.18E-03	2.94E-02
TYW1	0.28	1.21	1.71E-02	7.10E-02	PIAS2	0.31	1.24	7.24E-03	2.96E-02
PCGF2	-0.28	0.82	1.72E-02	7.11E-02	VWA5A	-0.33	0.80	7.24E-03	2.96E-02
TAP2	0.19	1.14	1.72E-02	7.11E-02	FERMT1	-0.20	0.87	7.29E-03	2.98E-02
SYNCRIP	0.18	1.13	1.72E-02	7.12E-02	SLC30A5	0.24	1.18	7.30E-03	2.98E-02
MRPL50	0.26	1.20	1.72E-02	7.12E-02	ANXA2	-0.17	0.89	7.31E-03	2.98E-02
GPAT3	-0.34	0.79	1.72E-02	7.13E-02	OSGEPL1	0.37	1.29	7.31E-03	2.98E-02
DNAJC13	0.18	1.14	1.73E-02	7.13E-02	C18orf25	-0.24	0.85	7.31E-03	2.98E-02
GLMN	0.34	1.26	1.73E-02	7.13E-02	KIAA0232	0.23	1.17	7.32E-03	2.98E-02
MARVELD2	-0.23	0.85	1.73E-02	7.13E-02	ITM2C	0.26	1.20	7.33E-03	2.99E-02
SOX12	-0.26	0.83	1.73E-02	7.14E-02	FAM98B	0.23	1.18	7.35E-03	2.99E-02
LINC01094	-0.37	0.78	1.73E-02	NA	GOT1	0.21	1.16	7.35E-03	3.00E-02
TIGD5	-0.36	0.78	1.73E-02	7.15E-02	UCHL1	0.90	1.86	7.37E-03	3.00E-02
GSDMA	0.30	1.23	1.73E-02	7.15E-02	IER3IP1	0.30	1.23	7.38E-03	3.00E-02
ADAM10	0.19	1.14	1.73E-02	7.15E-02	GDAP1	-0.33	0.80	7.38E-03	3.00E-02
MOK	0.32	1.25	1.73E-02	7.15E-02	SLFN13	0.40	1.32	7.39E-03	3.01E-02
SRD5A1	-0.24	0.85	1.75E-02	7.20E-02	RAB18	0.20	1.15	7.41E-03	3.01E-02
SLC22A20	0.43	1.35	1.75E-02	7.20E-02	VAMP3	-0.19	0.88	7.41E-03	3.01E-02
PELI3	-0.36	0.78	1.75E-02	7.21E-02	PRPF40A	0.18	1.14	7.41E-03	3.01E-02
G6PC3	-0.21	0.86	1.75E-02	7.21E-02	WAC-AS1	-0.33	0.80	7.41E-03	3.01E-02
IGF2BP2	0.14	1.10	1.75E-02	7.22E-02	ELMOD3	-0.27	0.83	7.44E-03	3.02E-02
CYB5R1	0.16	1.12	1.75E-02	7.22E-02	GNB1L	0.42	1.33	7.48E-03	3.04E-02
SLC25A25-AS1	0.44	1.35	1.76E-02	7.22E-02	MIDN	-0.22	0.86	7.48E-03	3.04E-02
PPM1F	0.19	1.14	1.76E-02	7.22E-02	IFT88	0.39	1.31	7.49E-03	3.04E-02
ZNF324B	-0.37	0.77	1.76E-02	7.23E-02	TOM1L1	0.23	1.18	7.53E-03	3.05E-02
RPL7L1	0.15	1.11	1.76E-02	7.23E-02	TSPAN2	1.25	2.37	7.55E-03	3.06E-02
RAPGEF5	0.30	1.23	1.76E-02	7.25E-02	RP11-890B15.3	-0.44	0.74	7.56E-03	3.07E-02
RPL32	-0.13	0.91	1.76E-02	7.25E-02	CEP164	-0.24	0.85	7.60E-03	3.08E-02
RP11-157J24.2	-0.23	0.85	1.77E-02	NA	SNX25	0.37	1.29	7.63E-03	3.09E-02
C20orf194	0.26	1.20	1.77E-02	7.28E-02	CORO1A	0.47	1.38	7.65E-03	3.10E-02
RP11-640M9.2	0.33	1.26	1.77E-02	7.28E-02	MORC2	-0.25	0.84	7.70E-03	3.12E-02
TMEM147	-0.19	0.88	1.77E-02	7.28E-02	HAUS5	0.26	1.20	7.71E-03	3.12E-02
ULBP2	-0.25	0.84	1.78E-02	7.32E-02	CUTC	0.32	1.25	7.71E-03	3.12E-02
EXT2	0.16	1.12	1.78E-02	7.32E-02	PLAGL1	-0.27	0.83	7.71E-03	3.12E-02
SDCBP	0.21	1.15	1.79E-02	7.32E-02	PIK3IP1	-0.32	0.80	7.75E-03	3.14E-02
KCNJ15	-0.25	0.84	1.79E-02	7.33E-02	AFAP1	0.23	1.17	7.76E-03	3.14E-02
DLGAP4	0.19	1.14	1.79E-02	7.35E-02	ZNF579	-0.45	0.73	7.77E-03	3.14E-02
KXD1	0.21	1.16	1.80E-02	7.36E-02	PAIP2	0.30	1.23	7.79E-03	3.15E-02
PSPH	-0.24	0.85	1.80E-02	7.36E-02	NIPAL1	-0.24	0.85	7.80E-03	3.15E-02
TMEM109	-0.19	0.88	1.80E-02	7.36E-02	UBE2D2	0.20	1.15	7.81E-03	3.15E-02
JUN	-0.23	0.85	1.80E-02	7.37E-02	PRRG2	-0.38	0.77	7.82E-03	3.16E-02
MCAT	-0.30	0.81	1.80E-02	7.37E-02	BAIAP2L2	0.56	1.48	7.82E-03	3.16E-02
KDELR1	-0.16	0.90	1.80E-02	7.37E-02	C11orf58	0.20	1.15	7.85E-03	3.17E-02
FBXL19-AS1	0.41	1.33	1.80E-02	7.37E-02	OR2A7	-1.12	0.46	7.87E-03	3.18E-02
PPP1R16A	-0.31	0.80	1.81E-02	7.39E-02	GPATCH8	-0.21	0.86	7.87E-03	3.18E-02
SH3GLB2	0.20	1.15	1.81E-02	7.39E-02	TP53TG1	-0.33	0.80	7.88E-03	3.18E-02
TRNT1	0.31	1.24	1.81E-02	7.39E-02	ULBP3	0.59	1.50	7.89E-03	3.18E-02
PTS	0.26	1.20	1.81E-02	7.41E-02	PHF11	-0.28	0.83	7.90E-03	3.18E-02
IL1B	-0.19	0.88	1.82E-02	7.42E-02	MAN2A1	0.21	1.16	7.91E-03	3.19E-02
POLG2	0.38	1.30	1.82E-02	7.42E-02	TWSG1	-0.22	0.86	7.93E-03	3.20E-02
CKAP2L	0.20	1.14	1.82E-02	7.42E-02	BMF	1.01	2.02	7.95E-03	3.20E-02
PPP2R5E	0.20	1.15	1.82E-02	7.43E-02	HEBP2	-0.20	0.87	8.00E-03	3.22E-02
PGD	-0.14	0.91	1.82E-02	7.43E-02	SACS	0.24	1.18	8.01E-03	3.22E-02
TBL2	0.21	1.16	1.82E-02	7.43E-02	LTBR	0.19	1.14	8.01E-03	3.22E-02
MRI1	-0.26	0.83	1.82E-02	7.44E-02	MRPL20	0.23	1.17	8.02E-03	3.22E-02
DMTN	-0.35	0.78	1.82E-02	7.44E-02	CNTNAP2	0.32	1.25	8.04E-03	3.23E-02
C6orf132	-0.16	0.90	1.83E-02	7.44E-02	MBTD1	0.34	1.27	8.06E-03	3.24E-02
TK2	-0.34	0.79	1.83E-02	7.45E-02	PPP1R14C	0.19	1.14	8.06E-03	3.24E-02
FAR1	0.20	1.15	1.83E-02	7.45E-02	CEP55	0.57	1.49	8.09E-03	3.25E-02
SNW1	0.18	1.13	1.83E-02	7.46E-02	NAT9	-0.26	0.84	8.10E-03	3.25E-02
RPL39	-0.29	0.82	1.83E-02	7.46E-02	ADH7	-1.72	0.30	8.11E-03	NA
ANKRD13B	-0.33	0.80	1.84E-02	7.48E-02	P4HA2	-0.21	0.87	8.12E-03	3.26E-02
PITPNA	0.16	1.11	1.84E-02	7.50E-02	DTNB	-0.29	0.82	8.16E-03	3.27E-02
CPNE3	0.17	1.12	1.85E-02	7.51E-02	ENTPD4	0.20	1.15	8.16E-03	3.27E-02
STK38	0.19	1.14	1.85E-02	7.52E-02	PCAT6	-0.64	0.64	8.17E-03	3.28E-02
OAT	0.17	1.12	1.86E-02	7.54E-02	ADGRF1	0.38	1.30	8.17E-03	3.28E-02
ACAP2	0.19	1.14	1.86E-02	7.57E-02	ZNF326	0.24	1.18	8.17E-03	3.28E-02
PVRL1	-0.15	0.90	1.87E-02	7.58E-02	C2CD2L	-0.35	0.78	8.19E-03	3.28E-02
NMB	-0.39	0.76	1.87E-02	7.59E-02	ABTB1	-0.37	0.77	8.19E-03	3.28E-02
KIAA2013	0.20	1.15	1.87E-02	7.59E-02	ATP5C1	0.18	1.14	8.20E-03	3.28E-02
GTF2A2	0.22	1.17	1.87E-02	7.60E-02	DEPDC1B	0.29	1.23	8.20E-03	3.28E-02
IMPDH2	-0.13	0.91	1.87E-02	7.60E-02	RAP2B	-0.26	0.84	8.23E-03	3.29E-02
FKBP9	-0.16	0.90	1.88E-02	7.62E-02	ZNF528	-0.37	0.78	8.24E-03	3.30E-02
SRF	0.20	1.15	1.88E-02	7.62E-02	STEAP4	-0.30	0.81	8.24E-03	3.30E-02
TNFRSF1A	-0.17	0.89	1.88E-02	7.62E-02	TIMM13	0.21	1.15	8.26E-03	3.30E-02
COL5A2	-0.32	0.80	1.88E-02	7.62E-02	SCRN2	-0.25	0.84	8.27E-03	3.30E-02
STK25	0.16	1.12	1.88E-02	7.63E-02	RB1	0.21	1.15	8.27E-03	3.30E-02
NELFA	0.24	1.18	1.89E-02	7.65E-02	CRNKL1	0.25	1.19	8.28E-03	3.31E-02

TCEAL8	-0.25	0.84	1.89E-02	7.66E-02
CSNK1G3	0.23	1.17	1.89E-02	7.66E-02
C1orf109	0.25	1.19	1.89E-02	7.66E-02
UNC13B	0.19	1.14	1.90E-02	7.67E-02
MBNL1	0.18	1.13	1.90E-02	7.67E-02
PNN	0.18	1.13	1.90E-02	7.67E-02
SLC29A2	-0.36	0.78	1.90E-02	7.67E-02
TMPRSS11D	-0.27	0.83	1.90E-02	7.67E-02
VPS53	0.18	1.14	1.90E-02	7.68E-02
CCT4	0.15	1.11	1.91E-02	7.70E-02
USP11	-0.24	0.84	1.91E-02	7.70E-02
UBE2K	0.19	1.14	1.91E-02	7.70E-02
SIRT2	-0.21	0.87	1.91E-02	7.71E-02
RTN4RL1	-0.25	0.84	1.91E-02	7.71E-02
FTH1	-0.37	0.77	1.91E-02	7.71E-02
TMEM50A	0.18	1.13	1.91E-02	7.71E-02
SEC61G	0.28	1.21	1.92E-02	7.73E-02
SART1	0.18	1.13	1.92E-02	7.73E-02
UBE2E2	0.22	1.16	1.92E-02	7.73E-02
AEN	0.21	1.15	1.92E-02	7.74E-02
CLPB	0.24	1.18	1.93E-02	7.75E-02
TMEM154	-0.19	0.88	1.93E-02	7.77E-02
TCEA2	-0.35	0.79	1.93E-02	7.78E-02
LASP1	0.15	1.11	1.94E-02	7.81E-02
FNDC3A	0.19	1.14	1.94E-02	7.81E-02
C2orf69	0.29	1.22	1.94E-02	7.81E-02
CALCRL	-0.42	0.75	1.95E-02	7.81E-02
HMG20B	0.18	1.14	1.95E-02	7.83E-02
MTF2	0.30	1.23	1.95E-02	7.83E-02
SRPRB	-0.20	0.87	1.95E-02	7.83E-02
FOXO3	-0.24	0.85	1.96E-02	7.84E-02
RAD23B	0.13	1.10	1.96E-02	7.84E-02
SREK1	0.20	1.15	1.96E-02	7.86E-02
RPL28	0.17	1.12	1.97E-02	7.88E-02
C9orf172	-0.44	0.74	1.97E-02	7.88E-02
LSG1	0.19	1.14	1.97E-02	7.89E-02
TOM1L1	0.21	1.16	1.97E-02	7.89E-02
MANBAL	0.19	1.14	1.97E-02	7.89E-02
CHP2	-0.44	0.74	1.97E-02	7.90E-02
LRP8	0.21	1.15	1.97E-02	7.90E-02
PRKCQ-AS1	-0.39	0.76	1.98E-02	7.90E-02
TNFAIP2	0.32	1.25	1.98E-02	7.93E-02
BTD	0.38	1.30	1.98E-02	7.93E-02
RAB8A	0.19	1.14	1.99E-02	7.95E-02
GMEB1	-0.27	0.83	1.99E-02	7.95E-02
AMBRA1	0.23	1.17	1.99E-02	7.95E-02
LIAS	0.29	1.22	1.99E-02	7.95E-02
BOLA1	-0.35	0.78	2.00E-02	7.97E-02
TMCO3	0.19	1.14	2.00E-02	7.97E-02
MT-ND4L	0.36	1.28	2.00E-02	7.97E-02
DENND6A	0.23	1.18	2.00E-02	7.97E-02
KIAA1524	0.22	1.17	2.00E-02	7.97E-02
CHCHD7	0.26	1.19	2.00E-02	7.97E-02
PCYT2	-0.21	0.87	2.00E-02	7.97E-02
JMJD7-PLA2G4B	-0.42	0.75	2.00E-02	7.98E-02
CDCA7L	0.21	1.16	2.01E-02	7.98E-02
CCDC106	-0.39	0.76	2.01E-02	7.99E-02
F8	-0.42	0.74	2.01E-02	8.00E-02
MTFMT	0.27	1.21	2.01E-02	8.01E-02
PUSL1	-0.28	0.82	2.01E-02	8.01E-02
TBPL1	0.29	1.22	2.02E-02	8.02E-02
ARL5A	0.21	1.16	2.02E-02	8.02E-02
CROCCP2	0.34	1.27	2.02E-02	8.03E-02
PPT2	-0.33	0.79	2.03E-02	8.05E-02
ICE1	0.19	1.14	2.03E-02	8.06E-02
CEP44	0.33	1.26	2.03E-02	8.07E-02
RP11-6N17.4	-0.43	0.74	2.03E-02	8.07E-02
THUMPD1	0.21	1.16	2.04E-02	8.09E-02
MLLT10	0.20	1.15	2.04E-02	8.10E-02
TMPO-AS1	-0.43	0.74	2.04E-02	8.10E-02
ORMDL2	0.23	1.17	2.04E-02	8.10E-02
GATSL3	-0.35	0.79	2.05E-02	NA
RPL35A	-0.13	0.91	2.05E-02	8.11E-02
ZBED4	0.19	1.14	2.05E-02	8.12E-02
PPP3CB-AS1	0.42	1.34	2.05E-02	8.14E-02
CCT2	0.15	1.11	2.06E-02	8.17E-02
RP1L1	-0.36	0.78	2.06E-02	NA
OTUD6B	0.29	1.22	2.06E-02	8.17E-02
ZNF467	-0.43	0.74	2.07E-02	8.18E-02
HDAC3	0.19	1.14	2.07E-02	8.18E-02
RFC5	-0.22	0.86	2.07E-02	8.18E-02
PPP6C	0.18	1.13	2.07E-02	8.18E-02
DHRS2	0.43	1.35	2.07E-02	8.19E-02
SOCS3	0.41	1.33	2.07E-02	8.19E-02
VWA5A	-0.22	0.86	2.07E-02	8.19E-02
OTUD1	-0.36	0.78	2.07E-02	8.19E-02
CSRP1	0.16	1.12	2.07E-02	8.19E-02
FRMD8	-0.22	0.86	2.07E-02	8.19E-02
UBXN2B	0.25	1.19	2.08E-02	8.19E-02
SEC14L2	0.18	1.14	2.08E-02	8.20E-02
ALG10B	0.34	1.27	8.30E-03	3.32E-02
CLPTM1L	0.19	1.14	8.31E-03	3.32E-02
BAZ1A	0.19	1.14	8.35E-03	3.33E-02
ICK	0.29	1.22	8.36E-03	3.33E-02
FAM135A	0.21	1.16	8.37E-03	3.34E-02
ATP5G3	0.18	1.14	8.40E-03	3.35E-02
ST5	-0.27	0.83	8.41E-03	3.35E-02
FBN2	-0.22	0.86	8.42E-03	3.36E-02
ZNF106	0.19	1.14	8.43E-03	3.36E-02
VEGFA	0.22	1.16	8.45E-03	3.37E-02
MAP4K4	0.20	1.15	8.46E-03	3.37E-02
ATF6B	-0.18	0.88	8.46E-03	3.37E-02
PPP1R11	-0.22	0.86	8.48E-03	3.37E-02
AEBP1	0.99	1.99	8.49E-03	3.38E-02
AKT1	0.19	1.14	8.52E-03	3.39E-02
KTN1	0.17	1.13	8.53E-03	3.39E-02
RPGRIP1L	0.31	1.24	8.53E-03	3.39E-02
SRF	-0.20	0.87	8.56E-03	3.40E-02
RBM25	0.19	1.14	8.56E-03	3.40E-02
KLHDC9	-0.89	0.54	8.57E-03	3.40E-02
SLPI	-0.53	0.69	8.59E-03	3.41E-02
SLC28A3	-0.85	0.56	8.63E-03	3.42E-02
PAPD5	0.24	1.18	8.65E-03	3.43E-02
HLA-DQB1	-0.47	0.72	8.66E-03	3.43E-02
CDC5L	0.21	1.16	8.67E-03	3.44E-02
ARVCF	0.46	1.37	8.67E-03	3.44E-02
DCPS	0.28	1.22	8.68E-03	3.44E-02
MLF1	0.42	1.34	8.70E-03	3.45E-02
POMP	0.21	1.16	8.70E-03	3.45E-02
MAFB	0.28	1.22	8.71E-03	3.45E-02
ZBTB2	0.32	1.25	8.71E-03	3.45E-02
ANOS1	0.52	1.44	8.73E-03	3.45E-02
HGH1	-0.92	0.53	8.75E-03	3.46E-02
PTPN9	-0.24	0.85	8.75E-03	3.46E-02
LEPROTL1	0.23	1.17	8.77E-03	3.47E-02
MAP2K5	-0.32	0.80	8.84E-03	3.49E-02
BRI3	0.24	1.18	8.85E-03	3.50E-02
TRMT1	-0.22	0.86	8.85E-03	3.50E-02
C12orf10	0.29	1.22	8.85E-03	3.50E-02
LARP4B	0.19	1.14	8.88E-03	3.51E-02
TTC3	0.18	1.14	8.91E-03	3.52E-02
CLASRP	-0.27	0.83	8.92E-03	3.52E-02
SYNJ2	0.27	1.20	8.92E-03	3.52E-02
NID2	-1.23	0.43	8.92E-03	NA
ID4	-1.33	0.40	8.93E-03	NA
ZNF138	0.40	1.32	8.93E-03	3.52E-02
DMBT1	-2.40	0.19	8.95E-03	NA
MT1E	0.33	1.26	8.97E-03	3.54E-02
RP11-115D19.3	-2.12	0.23	8.98E-03	NA
MAPK4	-1.08	0.47	9.00E-03	3.55E-02
PLPP5	-0.32	0.80	9.00E-03	3.55E-02
SGK494	-0.54	0.69	9.04E-03	3.56E-02
ATP1A1	0.19	1.14	9.05E-03	3.56E-02
TMBIM6	0.17	1.12	9.05E-03	3.56E-02
DDX55	0.26	1.19	9.06E-03	3.56E-02
PRICKLE2	-0.32	0.80	9.06E-03	3.57E-02
GNB5	0.27	1.20	9.06E-03	3.57E-02
INPP5A	0.32	1.25	9.08E-03	3.57E-02
SERPINB6	0.21	1.16	9.10E-03	3.58E-02
PLAC4	-1.98	0.25	9.10E-03	NA
TMEM254	-0.28	0.82	9.11E-03	3.58E-02
ZNF85	0.39	1.31	9.14E-03	3.59E-02
IFIT3	-0.31	0.81	9.17E-03	3.60E-02
GINS4	0.37	1.29	9.18E-03	3.60E-02
TRIM68	-0.33	0.80	9.18E-03	3.60E-02
PABPC1L	-0.31	0.81	9.22E-03	3.62E-02
PTGR1	0.21	1.16	9.25E-03	3.63E-02
C1orf226	0.43	1.35	9.28E-03	3.64E-02
NASP	0.22	1.16	9.28E-03	3.64E-02
FBXO45	0.23	1.17	9.34E-03	3.66E-02
ZFP36L1	-0.18	0.88	9.35E-03	3.66E-02
SOD1	0.19	1.14	9.35E-03	3.66E-02
PDSS1	0.35	1.27	9.35E-03	3.66E-02
ZNF552	-0.33	0.79	9.37E-03	3.67E-02
PSMD5	0.20	1.15	9.41E-03	3.68E-02
GALE	0.23	1.17	9.42E-03	3.68E-02
TSTD1	-0.27	0.83	9.45E-03	3.70E-02
NTN4	0.25	1.19	9.46E-03	3.70E-02
SERPING1	-0.30	0.81	9.49E-03	3.71E-02
TRIM27	-0.23	0.85	9.53E-03	3.72E-02
DDX27	0.21	1.16	9.54E-03	3.73E-02
PAQR8	0.70	1.62	9.54E-03	3.73E-02
GSTT1	-0.32	0.80	9.55E-03	3.73E-02
MTMR4	0.23	1.17	9.55E-03	3.73E-02
PPP6R3	0.19	1.14	9.56E-03	3.73E-02
S100A11	-0.18	0.89	9.59E-03	3.74E-02
YIPF1	-0.26	0.84	9.60E-03	3.74E-02
ITGB3BP	0.29	1.22	9.61E-03	3.75E-02
COTL1	0.22	1.17	9.62E-03	3.75E-02
PVRL2	0.23	1.18	9.64E-03	3.76E-02

ORC6	0.23	1.17	2.08E-02	8.21E-02
GHR	-0.36	0.78	2.08E-02	NA
CXXC1	0.21	1.16	2.09E-02	8.23E-02
ANO4	0.27	1.21	2.09E-02	8.23E-02
SLC27A5	-0.38	0.77	2.09E-02	8.23E-02
NUP88	0.17	1.12	2.10E-02	8.26E-02
PTRH2	0.23	1.17	2.10E-02	8.26E-02
ATPIF1	-0.16	0.89	2.10E-02	8.26E-02
TANGO6	0.24	1.18	2.10E-02	8.26E-02
MMP17	-0.41	0.75	2.11E-02	8.28E-02
CEP192	0.19	1.14	2.11E-02	8.28E-02
FSTL4	-0.36	0.78	2.11E-02	NA
TLR6	-0.40	0.76	2.11E-02	8.29E-02
RANBP3	0.19	1.14	2.11E-02	8.29E-02
ARL5B	0.28	1.21	2.11E-02	8.29E-02
CCDC115	-0.25	0.84	2.11E-02	8.29E-02
WDR5B	-0.40	0.76	2.11E-02	8.30E-02
ARHGEF10L	-0.22	0.86	2.12E-02	8.31E-02
MPP3	0.34	1.27	2.12E-02	8.32E-02
PTPN3	0.18	1.13	2.12E-02	8.32E-02
INTS10	0.18	1.13	2.12E-02	8.33E-02
TBP	0.24	1.18	2.12E-02	8.33E-02
ATXN1L	0.19	1.14	2.12E-02	8.33E-02
NR2C2AP	-0.29	0.82	2.13E-02	8.34E-02
FAM83B	0.19	1.14	2.13E-02	8.34E-02
NADK	0.21	1.15	2.13E-02	8.36E-02
NLK	0.27	1.20	2.14E-02	8.37E-02
BAG3	0.15	1.11	2.14E-02	8.37E-02
NARFL	-0.26	0.83	2.14E-02	8.38E-02
TFB2M	0.28	1.21	2.16E-02	8.44E-02
ITM2C	-0.27	0.83	2.16E-02	8.44E-02
PARP6	0.26	1.20	2.16E-02	8.44E-02
POLR2J4	0.40	1.32	2.16E-02	8.45E-02
ZC3H7A	0.21	1.16	2.16E-02	8.45E-02
WWC3	0.19	1.14	2.16E-02	8.46E-02
ADGRF2	-0.43	0.74	2.17E-02	8.46E-02
NOMO1	0.19	1.14	2.17E-02	8.48E-02
PUS1	0.23	1.17	2.18E-02	8.49E-02
AKT1S1	-0.19	0.88	2.19E-02	8.54E-02
HS3ST6	-0.41	0.75	2.19E-02	8.55E-02
ADAMTSL4	-0.20	0.87	2.19E-02	8.55E-02
ABCE1	0.18	1.13	2.19E-02	8.55E-02
ADIRF-AS1	-0.38	0.77	2.19E-02	8.56E-02
GON4L	0.20	1.15	2.21E-02	8.60E-02
NKIRAS1	0.35	1.27	2.21E-02	8.61E-02
TEK	-0.28	0.82	2.21E-02	NA
KLHL34	-0.27	0.83	2.21E-02	NA
ATP6V1G1	0.16	1.12	2.21E-02	8.62E-02
TP53I3	-0.21	0.87	2.23E-02	8.67E-02
STEAP4	0.25	1.19	2.23E-02	8.68E-02
LRRK1	0.21	1.15	2.23E-02	8.68E-02
NQO1	-0.21	0.87	2.23E-02	8.68E-02
SEC23A	0.18	1.13	2.23E-02	8.68E-02
ANKRD10	0.31	1.24	2.24E-02	8.70E-02
RP11-58O9.2	0.26	1.19	2.24E-02	8.71E-02
SLC35G1	-0.32	0.80	2.24E-02	8.71E-02
IL6R	-0.22	0.86	2.24E-02	8.71E-02
CIAPIN1	0.18	1.13	2.24E-02	8.71E-02
ANK1	-0.42	0.75	2.24E-02	8.71E-02
RPL23A	-0.19	0.88	2.24E-02	8.71E-02
DGKE	0.35	1.27	2.25E-02	8.73E-02
TMEM177	-0.27	0.83	2.25E-02	8.74E-02
QRICH1	0.16	1.12	2.25E-02	8.74E-02
HDGF	-0.12	0.92	2.26E-02	8.75E-02
METTL4	0.30	1.23	2.26E-02	8.76E-02
RNF19B	0.20	1.15	2.26E-02	8.76E-02
MIEN1	0.23	1.18	2.26E-02	8.77E-02
PANX1	0.19	1.14	2.26E-02	8.77E-02
RELA	0.17	1.12	2.27E-02	8.78E-02
RHBDL1	-0.35	0.79	2.27E-02	NA
VARS	-0.17	0.89	2.27E-02	8.80E-02
KRT42P	-0.43	0.74	2.28E-02	8.83E-02
CDC42BPA	0.19	1.14	2.28E-02	8.83E-02
CAMKK2	0.19	1.14	2.28E-02	8.83E-02
NRTN	-0.38	0.77	2.29E-02	8.84E-02
LAYN	0.35	1.27	2.30E-02	8.87E-02
TLDC1	0.15	1.11	2.30E-02	8.87E-02
TMEM70	0.26	1.20	2.30E-02	8.90E-02
RFX7	0.19	1.14	2.30E-02	8.90E-02
OXSR1	0.17	1.12	2.31E-02	8.92E-02
SLC22A18AS	-0.36	0.78	2.31E-02	NA
TMEM254	-0.25	0.84	2.32E-02	8.95E-02
FBXL18	-0.26	0.84	2.32E-02	8.95E-02
SPIN4	-0.30	0.81	2.32E-02	8.95E-02
FBXO6	-0.30	0.81	2.32E-02	8.96E-02
MRPL40	-0.22	0.86	2.32E-02	8.96E-02
ITM2B	-0.16	0.89	2.33E-02	8.97E-02
CWH43	-0.19	0.88	2.33E-02	8.97E-02
TIMM23	0.22	1.16	2.33E-02	8.98E-02
KLC1	0.23	1.18	2.33E-02	8.99E-02
OLFML3	0.96	1.95	9.65E-03	3.76E-02
E2F6	-0.37	0.77	9.67E-03	3.77E-02
TNKS2	0.20	1.15	9.68E-03	3.77E-02
CTSB	0.17	1.12	9.72E-03	3.78E-02
ESPN	0.31	1.24	9.72E-03	3.78E-02
MTCH2	0.19	1.14	9.75E-03	3.79E-02
TONSL	0.25	1.19	9.78E-03	3.80E-02
FTLP14	-0.99	0.50	9.78E-03	3.80E-02
ZNF724P	0.63	1.55	9.78E-03	3.80E-02
KCND1	0.64	1.56	9.80E-03	3.81E-02
C9orf3	0.24	1.18	9.81E-03	3.81E-02
CEP170	-0.28	0.82	9.82E-03	3.81E-02
TMEM256-PLSCR3	-0.19	0.87	9.82E-03	3.81E-02
AC092171.4	-0.27	0.83	9.85E-03	3.82E-02
PKD2	0.26	1.20	9.86E-03	3.82E-02
DNPEP	0.22	1.16	9.87E-03	3.83E-02
SLC31A2	-0.80	0.58	9.88E-03	3.83E-02
GNPNAT1	0.25	1.19	9.90E-03	3.84E-02
ALDH9A1	0.23	1.17	9.92E-03	3.84E-02
DNMT3A	-0.29	0.82	9.94E-03	3.85E-02
SNW1	0.21	1.16	9.94E-03	3.85E-02
RP11-382A20.3	0.24	1.18	9.97E-03	3.86E-02
OGDHL	0.87	1.82	9.99E-03	3.87E-02
CNOT1	-0.17	0.89	1.00E-02	3.87E-02
FABP5	-0.25	0.84	1.00E-02	3.87E-02
EPB41L4A	-0.31	0.81	1.00E-02	3.87E-02
TEAD1	0.19	1.14	1.00E-02	3.87E-02
SEMA3D	-0.44	0.74	1.00E-02	3.87E-02
ARID4B	0.23	1.18	1.00E-02	3.87E-02
DUSP8	-0.44	0.74	1.00E-02	3.87E-02
EMC9	0.33	1.26	1.00E-02	3.87E-02
APCDD1	0.23	1.17	1.00E-02	3.87E-02
CHURC1	-0.28	0.82	1.00E-02	3.87E-02
MGEA5	0.20	1.15	1.00E-02	3.87E-02
KLF10	0.19	1.14	1.01E-02	3.88E-02
LARS	0.20	1.15	1.01E-02	3.88E-02
IFIH1	-0.23	0.85	1.01E-02	3.88E-02
NQO2	0.31	1.24	1.01E-02	3.88E-02
FOXJ1	3.24	9.43	1.01E-02	NA
DIEXF	-0.27	0.83	1.01E-02	3.88E-02
PDP2	0.22	1.17	1.01E-02	3.88E-02
ZNF276	-0.32	0.80	1.01E-02	3.88E-02
CPT1C	0.69	1.61	1.01E-02	3.89E-02
KIF18B	0.31	1.24	1.01E-02	3.89E-02
BAG5	0.21	1.15	1.01E-02	3.89E-02
TP73-AS1	0.26	1.20	1.02E-02	3.91E-02
ZSCAN31	-0.34	0.79	1.02E-02	3.92E-02
TLK1	0.24	1.18	1.02E-02	3.92E-02
AC006262.5	-0.43	0.74	1.02E-02	3.93E-02
PPP1R10	0.22	1.17	1.02E-02	3.93E-02
PRKDC	0.19	1.14	1.02E-02	3.93E-02
FLJ20021	-0.67	0.63	1.02E-02	3.93E-02
RHBDF2	-0.25	0.84	1.03E-02	3.94E-02
SBDSP1	-0.29	0.82	1.03E-02	3.94E-02
PHF23	0.22	1.17	1.03E-02	3.94E-02
UBE4A	0.21	1.16	1.03E-02	3.94E-02
PAPSS2	0.72	1.64	1.03E-02	3.95E-02
NCKAP1	0.17	1.12	1.03E-02	3.95E-02
NPAT	0.27	1.21	1.03E-02	3.96E-02
EGFL7	-0.48	0.72	1.03E-02	3.96E-02
TNFAIP2	-0.33	0.79	1.03E-02	3.96E-02
STK17A	-0.20	0.87	1.04E-02	3.97E-02
RAB29	0.24	1.18	1.04E-02	3.97E-02
CARKD	-0.26	0.84	1.04E-02	3.98E-02
RP11-517C16.2	-0.91	0.53	1.04E-02	3.98E-02
PDCD11	0.19	1.14	1.04E-02	3.98E-02
CDC42EP3	-0.24	0.84	1.04E-02	3.98E-02
AK2	0.18	1.13	1.04E-02	3.99E-02
DNAJC6	0.55	1.46	1.04E-02	3.99E-02
BTG1	-0.27	0.83	1.04E-02	3.99E-02
PAX8-AS1	-0.31	0.81	1.04E-02	3.99E-02
TMEM170A	0.29	1.23	1.05E-02	3.99E-02
WIPI1	-0.32	0.80	1.05E-02	4.00E-02
MRPL14	-0.22	0.86	1.05E-02	4.00E-02
PIN1	-0.22	0.86	1.05E-02	4.00E-02
SLC9A9	-0.47	0.72	1.05E-02	4.01E-02
ZNF701	0.43	1.35	1.05E-02	4.01E-02
GOPC	0.19	1.14	1.05E-02	4.01E-02
LRCH1	0.26	1.20	1.05E-02	4.01E-02
RP11-73M18.8	0.38	1.30	1.05E-02	4.02E-02
SLC6A2	-0.81	0.57	1.05E-02	4.02E-02
NSRP1	0.25	1.19	1.06E-02	4.02E-02
TLE4	-0.27	0.83	1.06E-02	4.04E-02
SOD2	0.21	1.16	1.06E-02	4.04E-02
RPL39L	0.41	1.33	1.06E-02	4.04E-02
SLC5A3	0.23	1.17	1.06E-02	4.04E-02
EPS15L1	0.22	1.16	1.07E-02	4.05E-02
CAPN2	-0.18	0.89	1.07E-02	4.06E-02
FAM102B	0.44	1.36	1.07E-02	4.06E-02
BTBD10	-0.21	0.87	1.07E-02	4.06E-02

FAM3D	-0.42	0.75	2.34E-02	9.00E-02
UBE2Z	0.14	1.10	2.34E-02	9.00E-02
BET1L	-0.22	0.86	2.34E-02	9.00E-02
POC1A	0.22	1.16	2.34E-02	9.02E-02
NOD2	-0.30	0.81	2.35E-02	9.03E-02
NHLH2	-0.40	0.76	2.35E-02	9.03E-02
SOX6	-0.40	0.76	2.35E-02	9.03E-02
MPRIP	0.14	1.10	2.35E-02	9.04E-02
MICAL3	0.20	1.15	2.35E-02	9.04E-02
C6orf48	-0.19	0.88	2.36E-02	9.04E-02
MUC16	-0.42	0.75	2.36E-02	9.04E-02
RAPGEF6	0.38	1.30	2.36E-02	9.05E-02
ARF1	0.15	1.11	2.36E-02	9.06E-02
AKR1B15	-0.33	0.80	2.36E-02	NA
ZYX	0.18	1.13	2.37E-02	9.08E-02
MCL1	0.14	1.10	2.37E-02	9.08E-02
BCL2A1	0.32	1.25	2.37E-02	NA
MYO9B	0.17	1.13	2.37E-02	9.10E-02
INPP5J	-0.42	0.75	2.38E-02	9.12E-02
MYOM3	-0.37	0.77	2.38E-02	9.13E-02
SMAD3	0.16	1.11	2.38E-02	9.13E-02
SNUPN	-0.31	0.81	2.38E-02	9.13E-02
GBP4	-0.30	0.81	2.39E-02	NA
ADD3	-0.21	0.86	2.39E-02	9.16E-02
MAP1A	-0.42	0.75	2.40E-02	9.17E-02
GTF3C1	0.16	1.11	2.40E-02	9.17E-02
NT5C2	0.21	1.16	2.40E-02	9.17E-02
KCNS1	-0.42	0.75	2.40E-02	9.17E-02
STRN	0.20	1.15	2.40E-02	9.17E-02
IGSF3	-0.15	0.90	2.40E-02	9.17E-02
CBX7	-0.42	0.75	2.40E-02	9.18E-02
YKT6	0.15	1.11	2.41E-02	9.19E-02
SBNO1	0.18	1.13	2.41E-02	9.19E-02
HOMER3	-0.19	0.88	2.41E-02	9.21E-02
WRNIP1	0.17	1.13	2.41E-02	9.21E-02
IL12RB2	-0.41	0.75	2.42E-02	9.23E-02
GDAP1	-0.29	0.82	2.42E-02	9.24E-02
RP11-660L16.2	-0.41	0.75	2.42E-02	9.24E-02
D2HGDH	-0.30	0.81	2.43E-02	9.25E-02
RILP	-0.42	0.75	2.43E-02	9.25E-02
BPTF	0.17	1.12	2.43E-02	9.26E-02
AL627309.1	0.42	1.34	2.43E-02	9.27E-02
ADAP1	-0.35	0.78	2.43E-02	9.27E-02
ATP6V1E1	0.16	1.12	2.43E-02	9.27E-02
LIG4	0.25	1.19	2.44E-02	9.27E-02
ARV1	0.25	1.19	2.44E-02	9.28E-02
LINC00657	0.15	1.11	2.44E-02	9.28E-02
SNRNP40	-0.19	0.87	2.44E-02	9.28E-02
CD320	-0.27	0.83	2.44E-02	9.29E-02
DYRK4	-0.27	0.83	2.45E-02	9.29E-02
CLDN4	-0.19	0.88	2.45E-02	9.30E-02
B4GALT1	-0.15	0.90	2.45E-02	9.30E-02
COX6B1	-0.15	0.90	2.45E-02	9.30E-02
ZNF787	-0.24	0.85	2.45E-02	9.31E-02
TCP1	0.14	1.10	2.45E-02	9.32E-02
GNB5	0.24	1.18	2.46E-02	9.32E-02
RBM22	-0.19	0.88	2.46E-02	9.34E-02
ASXL2	0.16	1.12	2.47E-02	9.35E-02
JAK1	0.16	1.12	2.47E-02	9.38E-02
DUOXA2	0.37	1.29	2.48E-02	9.39E-02
PP14571	-0.26	0.84	2.48E-02	NA
ZNF263	0.25	1.19	2.48E-02	9.39E-02
ZC3H7B	0.16	1.12	2.49E-02	9.42E-02
SURF6	0.19	1.14	2.49E-02	9.42E-02
NT5C3A	0.27	1.21	2.49E-02	9.43E-02
KIF7	0.33	1.26	2.50E-02	9.47E-02
ATAT1	0.36	1.29	2.50E-02	9.47E-02
SSR3	0.16	1.12	2.51E-02	9.48E-02
CTDSP2	0.17	1.12	2.51E-02	9.49E-02
IFI35	-0.34	0.79	2.51E-02	9.49E-02
MAPK12	-0.33	0.80	2.52E-02	9.51E-02
LRFN4	-0.30	0.81	2.52E-02	9.52E-02
UBE2B	0.22	1.16	2.52E-02	9.53E-02
ATP6V0A2	0.24	1.18	2.52E-02	9.53E-02
C11orf45	-0.38	0.77	2.53E-02	9.53E-02
CASC5	0.21	1.16	2.53E-02	9.53E-02
C10orf2	0.22	1.16	2.53E-02	9.53E-02
RPL30	-0.15	0.90	2.53E-02	9.54E-02
HEATR1	0.16	1.12	2.53E-02	9.54E-02
FHL1	0.16	1.11	2.53E-02	9.54E-02
TMEM91	-0.42	0.75	2.53E-02	9.54E-02
ACADVL	0.15	1.11	2.53E-02	9.54E-02
FAM200B	0.31	1.24	2.53E-02	9.55E-02
PTCD3	0.18	1.14	2.54E-02	9.55E-02
FNIP1	-0.29	0.82	2.54E-02	9.56E-02
TRIM56	-0.19	0.88	2.54E-02	9.56E-02
PITPNM1	0.20	1.15	2.55E-02	9.58E-02
SIAE	-0.24	0.85	2.55E-02	9.58E-02
UROD	-0.18	0.88	2.55E-02	9.58E-02
LINC01232	-0.35	0.78	2.55E-02	9.58E-02

SNRNP48	0.27	1.21	1.07E-02	4.06E-02
AIFM1	0.21	1.16	1.07E-02	4.07E-02
SMG6	-0.23	0.85	1.07E-02	4.08E-02
DSTYK	0.21	1.16	1.07E-02	4.08E-02
MCRS1	0.20	1.15	1.07E-02	4.08E-02
NUDCD3	-0.20	0.87	1.08E-02	4.08E-02
TMEM222	-0.27	0.83	1.08E-02	4.08E-02
SUPT16H	-0.21	0.86	1.08E-02	4.08E-02
ADNP2	-0.23	0.85	1.08E-02	4.08E-02
CCDC167	-0.36	0.78	1.08E-02	4.10E-02
TREM2	-2.09	0.23	1.08E-02	NA
GEN1	0.27	1.21	1.09E-02	4.11E-02
KLK1	0.64	1.55	1.09E-02	4.12E-02
UTRN	-0.20	0.87	1.09E-02	4.12E-02
CPSF2	0.20	1.15	1.09E-02	4.12E-02
PKP3	0.18	1.13	1.09E-02	4.12E-02
DVL2	0.21	1.15	1.09E-02	4.12E-02
GHR	-0.90	0.54	1.09E-02	4.12E-02
STC1	3.14	8.84	1.09E-02	NA
CICP14	-0.36	0.78	1.09E-02	4.13E-02
SLC38A5	-0.21	0.87	1.09E-02	4.13E-02
RRM1	0.19	1.14	1.10E-02	4.14E-02
YIPF5	-0.21	0.86	1.10E-02	4.15E-02
KIAA1715	-0.21	0.86	1.10E-02	4.15E-02
ENO1	0.17	1.13	1.10E-02	4.15E-02
PDCD1LG2	-0.60	0.66	1.10E-02	4.15E-02
BRCC3	0.22	1.17	1.10E-02	4.15E-02
ECM1	-0.23	0.85	1.11E-02	4.18E-02
USP53	-0.21	0.86	1.11E-02	4.18E-02
HACL1	0.30	1.23	1.11E-02	4.19E-02
LMBRD2	-0.26	0.84	1.12E-02	4.20E-02
DLX4	-1.16	0.45	1.12E-02	NA
CTR9	0.19	1.14	1.12E-02	4.22E-02
PRKCH	0.29	1.23	1.12E-02	4.22E-02
GALNT6	-0.18	0.88	1.12E-02	4.22E-02
RALGAPA1P	-1.14	0.45	1.12E-02	NA
HAUS1	0.28	1.22	1.12E-02	4.23E-02
ZPLD1	-1.15	0.45	1.12E-02	NA
TPI1	0.17	1.12	1.12E-02	4.23E-02
TRIP11	0.21	1.15	1.13E-02	4.24E-02
TEKT4P2	-0.55	0.68	1.13E-02	4.25E-02
EIF1AD	-0.27	0.83	1.13E-02	4.26E-02
LRRFIP1	0.18	1.13	1.14E-02	4.29E-02
TET2	-0.21	0.87	1.14E-02	4.29E-02
SPRR1A	-0.56	0.68	1.15E-02	4.31E-02
SPA17	0.48	1.40	1.15E-02	4.31E-02
ANO8	-0.35	0.78	1.15E-02	4.31E-02
DDX41	0.20	1.15	1.15E-02	4.32E-02
ZNF81	-0.32	0.80	1.15E-02	4.32E-02
ZNF846	-0.46	0.73	1.15E-02	4.32E-02
THOC3	0.24	1.18	1.15E-02	4.33E-02
MGA	-0.22	0.86	1.16E-02	4.33E-02
LINC01420	-0.25	0.84	1.16E-02	4.34E-02
LPCAT2	0.21	1.16	1.16E-02	4.34E-02
SMARCA4	0.17	1.13	1.16E-02	4.34E-02
RSBN1L	0.26	1.19	1.16E-02	4.34E-02
RP11-274B21.4	-0.40	0.76	1.16E-02	4.35E-02
CEP57L1	0.32	1.25	1.16E-02	4.35E-02
FANCB	0.49	1.40	1.17E-02	4.36E-02
EARS2	0.25	1.19	1.17E-02	4.36E-02
RHNO1	0.25	1.19	1.17E-02	4.37E-02
PLA2G4E	-0.49	0.71	1.17E-02	4.37E-02
LINC01503	-0.28	0.82	1.17E-02	4.38E-02
NUDCD1	0.24	1.19	1.17E-02	4.38E-02
GYLTL1B	0.22	1.16	1.17E-02	4.38E-02
CSTF2T	0.22	1.17	1.17E-02	4.38E-02
DECR1	-0.21	0.87	1.17E-02	4.38E-02
ECHDC2	-0.23	0.85	1.18E-02	4.39E-02
RET	0.82	1.77	1.18E-02	4.41E-02
MANF	0.21	1.16	1.18E-02	4.41E-02
VOPP1	0.25	1.19	1.18E-02	4.41E-02
ZNF302	0.30	1.23	1.19E-02	4.42E-02
ZDHHC24	-0.28	0.83	1.19E-02	4.43E-02
TMEM102	-0.33	0.80	1.19E-02	4.44E-02
DPH6	0.41	1.33	1.19E-02	4.45E-02
GNL3L	0.30	1.23	1.19E-02	4.45E-02
ZNF207	0.18	1.13	1.20E-02	4.45E-02
TP53BP2	0.22	1.16	1.20E-02	4.45E-02
RQCD1	0.20	1.14	1.20E-02	4.46E-02
PTDSS2	-0.23	0.86	1.20E-02	4.47E-02
TRIM25	0.18	1.14	1.20E-02	4.47E-02
SDR9C7	-0.44	0.74	1.20E-02	4.47E-02
CTF1	-0.67	0.63	1.20E-02	4.48E-02
ZFAND6	0.22	1.16	1.21E-02	4.48E-02
RGMB	-0.25	0.84	1.22E-02	4.52E-02
COPE	-0.19	0.87	1.22E-02	4.52E-02
SRGAP2C	-0.50	0.70	1.22E-02	4.52E-02
PHLDB3	-0.26	0.83	1.22E-02	4.53E-02
C2CD4C	0.69	1.62	1.22E-02	4.53E-02
VDAC3	0.18	1.14	1.22E-02	4.53E-02

HOXC12	-0.42	0.75	2.55E-02	9.58E-02
NIP7	0.17	1.12	2.55E-02	9.59E-02
NFE2L2	0.17	1.13	2.55E-02	9.60E-02
CPSF2	0.18	1.13	2.56E-02	9.60E-02
RAD23A	0.17	1.13	2.56E-02	9.61E-02
TMEM181	0.19	1.14	2.56E-02	9.61E-02
MPP5	0.20	1.15	2.57E-02	9.63E-02
HSPD1	0.16	1.11	2.57E-02	9.63E-02
NIF3L1	0.21	1.16	2.57E-02	9.63E-02
NUDT18	-0.38	0.77	2.57E-02	9.64E-02
RP11-385F5.5	0.25	1.19	2.57E-02	NA
CEACAM5	-0.42	0.75	2.58E-02	9.66E-02
ZFYVE27	0.26	1.19	2.58E-02	9.66E-02
PCMT1	0.18	1.13	2.58E-02	9.66E-02
E4F1	0.23	1.17	2.58E-02	9.66E-02
PDCD6IP	0.14	1.10	2.58E-02	9.66E-02
SHC3	-0.38	0.77	2.59E-02	9.68E-02
ANKRD13A	0.17	1.12	2.59E-02	9.68E-02
ETV3	0.17	1.13	2.60E-02	9.71E-02
FZD5	-0.29	0.82	2.60E-02	9.71E-02
UBL4A	-0.19	0.88	2.60E-02	9.72E-02
POLG	0.18	1.13	2.60E-02	9.72E-02
MVP	0.16	1.12	2.60E-02	9.72E-02
CADM4	-0.23	0.85	2.60E-02	9.72E-02
RP11-430B1.2	-0.35	0.79	2.61E-02	NA
FOXM1	0.16	1.12	2.61E-02	9.73E-02
BAIAP2	-0.16	0.90	2.61E-02	9.74E-02
ARCN1	0.14	1.10	2.61E-02	9.74E-02
MICA	0.22	1.16	2.61E-02	9.74E-02
42803	-0.30	0.81	2.61E-02	9.74E-02
TCEAL4	-0.17	0.89	2.61E-02	9.74E-02
DRP2	0.40	1.32	2.61E-02	9.74E-02
TEX10	0.19	1.14	2.62E-02	9.76E-02
TGFB1I1	-0.32	0.80	2.62E-02	9.76E-02
ZNF761	0.30	1.23	2.62E-02	9.76E-02
THAP7	-0.28	0.82	2.62E-02	9.77E-02
HS3ST1	-0.24	0.85	2.62E-02	9.77E-02
BRCC3	-0.22	0.86	2.63E-02	9.77E-02
ZNF34	-0.42	0.75	2.63E-02	9.77E-02
WDR18	-0.22	0.86	2.63E-02	9.77E-02
PPP4R3A	0.18	1.14	2.63E-02	9.77E-02
GOLIM4	0.18	1.13	2.63E-02	9.77E-02
APOL2	-0.29	0.82	2.63E-02	9.77E-02
ST6GALNAC4	-0.28	0.82	2.63E-02	9.77E-02
TMEM33	0.16	1.12	2.63E-02	9.77E-02
ALG3	-0.21	0.86	2.63E-02	9.78E-02
CYTH2	0.20	1.15	2.64E-02	9.79E-02
RP11-541N10.3	-0.41	0.75	2.65E-02	9.82E-02
CCDC12	-0.23	0.85	2.65E-02	9.82E-02
IRAK1BP1	-0.33	0.80	2.65E-02	9.82E-02
THUMPD3	0.20	1.15	2.65E-02	9.83E-02
ALDH1B1	-0.22	0.86	2.65E-02	9.84E-02
SNHG18	-0.36	0.78	2.66E-02	9.85E-02
RPS29	-0.20	0.87	2.66E-02	9.85E-02
PPP1R14B	-0.17	0.89	2.66E-02	9.85E-02
RDH12	-0.41	0.75	2.67E-02	9.87E-02
CTA-29F11.1	-0.41	0.75	2.67E-02	9.90E-02
NFAT5	0.22	1.16	2.68E-02	9.91E-02
ZNF442	-0.36	0.78	2.69E-02	9.94E-02
SLC4A2	0.20	1.15	2.69E-02	9.94E-02
PPP2CB	0.17	1.13	2.69E-02	9.94E-02
OGDH	0.16	1.11	2.69E-02	9.94E-02
TRIM11	0.22	1.17	2.69E-02	9.96E-02
VCL	0.13	1.09	2.70E-02	9.97E-02
UVRAG	0.24	1.18	2.70E-02	9.97E-02
EDC3	0.20	1.15	2.70E-02	9.97E-02
RBFA	0.27	1.20	2.70E-02	9.97E-02
MFN1	0.19	1.14	2.70E-02	9.98E-02
ZFAND5	0.17	1.13	2.70E-02	9.98E-02
RP3-428L16.2	-0.36	0.78	2.71E-02	NA
TBX19	0.40	1.32	2.71E-02	1.00E-01
SIVA1	-0.23	0.86	2.71E-02	1.00E-01
PGM3	0.24	1.18	2.72E-02	1.00E-01
ASB8	-0.25	0.84	2.72E-02	1.00E-01
FKBPL	-0.34	0.79	2.72E-02	1.00E-01
IRAK2	0.38	1.30	2.72E-02	1.00E-01
HKDC1	-0.29	0.82	2.72E-02	NA
SBF1	0.19	1.14	2.73E-02	1.00E-01
CLOCK	0.17	1.13	2.73E-02	1.01E-01
CYP1A1	-0.35	0.79	2.73E-02	NA
ANKLE1	-0.34	0.79	2.73E-02	1.01E-01
MYO5B	0.15	1.11	2.73E-02	1.01E-01
MSL1	-0.17	0.89	2.73E-02	1.01E-01
CCDC146	0.41	1.33	2.74E-02	1.01E-01
ATP7B	-0.32	0.80	2.74E-02	1.01E-01
TINCR	-0.19	0.88	2.74E-02	1.01E-01
RP11-1275H24.1	-0.37	0.77	2.74E-02	1.01E-01
MMADHC	0.18	1.13	2.74E-02	1.01E-01
TRIM27	-0.20	0.87	2.74E-02	1.01E-01
NPIPB11	0.35	1.27	2.75E-02	NA
NOTCH2	0.16	1.12	1.22E-02	4.53E-02
CHID1	0.20	1.15	1.22E-02	4.53E-02
PWWP2A	0.29	1.23	1.22E-02	4.53E-02
CDC42SE1	-0.18	0.88	1.22E-02	4.53E-02
DPP9	0.19	1.14	1.22E-02	4.53E-02
GLRX3	0.18	1.14	1.22E-02	4.53E-02
POF1B	-0.22	0.86	1.23E-02	4.54E-02
F2R	0.30	1.23	1.23E-02	4.54E-02
GPR137B	0.32	1.25	1.23E-02	4.54E-02
RP11-34P13.13	-1.16	0.45	1.23E-02	NA
PPFIA3	-0.32	0.80	1.23E-02	4.55E-02
CDC34	0.21	1.16	1.23E-02	4.55E-02
LIMD1	0.21	1.16	1.23E-02	4.55E-02
APBB3	-0.44	0.74	1.23E-02	4.56E-02
TTLL5	-0.24	0.85	1.24E-02	4.57E-02
DKFZp434J0226	-0.49	0.71	1.24E-02	4.57E-02
ZNF558	-0.30	0.82	1.24E-02	4.57E-02
ADGRG6	0.22	1.17	1.24E-02	4.57E-02
FANCG	0.24	1.18	1.24E-02	4.58E-02
PHLDA2	0.24	1.18	1.24E-02	4.58E-02
GOLT1B	0.22	1.17	1.24E-02	4.58E-02
TP53AIP1	-0.27	0.83	1.24E-02	4.58E-02
MTCO1P12	-0.23	0.85	1.24E-02	4.58E-02
NARS	0.17	1.13	1.24E-02	4.58E-02
LAMA5	0.16	1.12	1.24E-02	4.58E-02
INO80C	-0.27	0.83	1.25E-02	4.60E-02
AIF1L	0.37	1.29	1.25E-02	4.60E-02
CDC42BPB	-0.20	0.87	1.25E-02	4.60E-02
RNF11	0.19	1.14	1.25E-02	4.61E-02
HOOK2	0.22	1.17	1.25E-02	4.62E-02
09-sep	0.17	1.13	1.26E-02	4.64E-02
SERTAD4	0.46	1.38	1.26E-02	4.65E-02
SPAG9	0.18	1.13	1.27E-02	4.66E-02
GBP1	-0.25	0.84	1.27E-02	4.67E-02
HACD4	0.27	1.21	1.27E-02	4.68E-02
JPH1	-0.33	0.80	1.27E-02	4.68E-02
PRDX2	0.18	1.13	1.27E-02	4.68E-02
POFUT2	0.26	1.20	1.28E-02	4.68E-02
UBE2L6	-0.20	0.87	1.28E-02	4.68E-02
WSB2	-0.18	0.88	1.29E-02	4.72E-02
KCTD15	-0.25	0.84	1.29E-02	4.74E-02
AL591893.1	-1.04	0.49	1.29E-02	4.74E-02
PTHLH	-0.60	0.66	1.29E-02	4.74E-02
PRPF3	0.21	1.16	1.30E-02	4.76E-02
RP1-253P7.4	-0.68	0.63	1.30E-02	4.76E-02
MUT	-0.22	0.86	1.30E-02	4.76E-02
RMI1	0.31	1.24	1.30E-02	4.77E-02
FXR2	0.20	1.15	1.30E-02	4.78E-02
LINC00662	-0.43	0.74	1.30E-02	4.78E-02
TGFBR2	-0.20	0.87	1.31E-02	4.78E-02
NEK4	0.25	1.19	1.31E-02	4.79E-02
GTF2H5	-0.25	0.84	1.31E-02	4.80E-02
CLASP1	-0.18	0.88	1.31E-02	4.81E-02
ARHGAP32	-0.19	0.88	1.32E-02	4.81E-02
ANO1	0.68	1.60	1.32E-02	4.81E-02
ARRDC1-AS1	-0.41	0.75	1.32E-02	4.82E-02
TTC23	-0.27	0.83	1.32E-02	4.82E-02
MBNL2	0.22	1.17	1.32E-02	4.84E-02
TESK1	-0.29	0.82	1.32E-02	4.84E-02
MXI1	-0.31	0.81	1.33E-02	4.84E-02
PPHLN1	0.19	1.14	1.33E-02	4.84E-02
CHMP2B	0.21	1.15	1.33E-02	4.85E-02
MRPL2	0.24	1.18	1.33E-02	4.85E-02
POLR3B	0.24	1.18	1.33E-02	4.86E-02
EXOSC7	0.24	1.18	1.33E-02	4.86E-02
VPS13C	-0.20	0.87	1.33E-02	4.86E-02
C7orf50	0.19	1.14	1.33E-02	4.86E-02
RP11-755F10.1	-0.82	0.57	1.33E-02	4.86E-02
MAF1	-0.20	0.87	1.34E-02	4.88E-02
DNAJA2	0.19	1.14	1.35E-02	4.90E-02
SRD5A1	0.21	1.15	1.35E-02	4.90E-02
PLXNB1	-0.17	0.89	1.35E-02	4.90E-02
SC5D	-0.22	0.86	1.35E-02	4.90E-02
RAC3	0.34	1.27	1.35E-02	4.91E-02
RAPGEF3	-0.31	0.80	1.35E-02	4.91E-02
ZNF195	-0.26	0.84	1.35E-02	4.91E-02
SULT1E1	-0.42	0.75	1.35E-02	4.92E-02
FCF1	0.21	1.16	1.36E-02	4.93E-02
DDI2	0.19	1.14	1.36E-02	4.93E-02
CCDC69	0.32	1.25	1.36E-02	4.93E-02
FAM102A	-0.25	0.84	1.36E-02	4.93E-02
PSMC3IP	0.46	1.38	1.36E-02	4.94E-02
C17orf62	-0.24	0.85	1.36E-02	4.94E-02
MAGOHB	0.34	1.27	1.37E-02	4.96E-02
JAGN1	-0.23	0.85	1.37E-02	4.96E-02
FAAP20	-0.28	0.82	1.37E-02	4.96E-02
MFSD6	0.19	1.14	1.37E-02	4.96E-02
FAM162A	-0.22	0.86	1.37E-02	4.96E-02
KCTD21	-0.35	0.79	1.37E-02	4.97E-02
PAFAH1B1	0.17	1.12	1.37E-02	4.98E-02

**RP11-146F11.1**	-0.30	0.81	2.75E-02	NA
**EIF6**	-0.17	0.89	2.75E-02	1.01E-01
**ITGA6**	0.15	1.11	2.75E-02	1.01E-01
**KHDRBS3**	-0.40	0.76	2.75E-02	1.01E-01
**CSNK1G2**	0.17	1.13	2.76E-02	1.01E-01
**TCF7**	-0.40	0.76	2.77E-02	1.02E-01
**TTYH2**	-0.41	0.75	2.77E-02	1.02E-01
**COL27A1**	0.21	1.16	2.77E-02	1.02E-01
**ALS2**	0.19	1.14	2.77E-02	1.02E-01
**PTBP3**	0.15	1.11	2.78E-02	1.02E-01
**SLC28A3**	-0.41	0.75	2.78E-02	1.02E-01
**GOPC**	0.22	1.16	2.78E-02	1.02E-01
**RHOD**	-0.20	0.87	2.78E-02	1.02E-01
**RNF216**	0.19	1.14	2.78E-02	1.02E-01
**AZIN1**	0.15	1.11	2.79E-02	1.02E-01
**TSPAN4**	-0.21	0.86	2.79E-02	1.02E-01
**NUP85**	0.17	1.12	2.79E-02	1.02E-01
**ZNF496**	0.20	1.15	2.79E-02	1.02E-01
**SGSM3**	0.19	1.14	2.79E-02	1.02E-01
**ZNF83**	0.25	1.19	2.80E-02	1.02E-01
**PART1**	-0.40	0.76	2.80E-02	1.02E-01
**RP11-336A10.5**	-0.31	0.81	2.80E-02	NA
**TTC7A**	-0.19	0.88	2.80E-02	1.02E-01
**GOLGB1**	0.18	1.13	2.81E-02	1.02E-01
**GRB2**	0.16	1.12	2.81E-02	1.02E-01
**PPP2CA**	0.16	1.12	2.81E-02	1.03E-01
**MAML3**	-0.33	0.80	2.82E-02	1.03E-01
**CHPF2**	0.23	1.17	2.83E-02	1.03E-01
**SUMF2**	-0.16	0.90	2.83E-02	1.03E-01
**BDH2**	-0.37	0.78	2.83E-02	1.03E-01
**TUBG2**	0.24	1.18	2.83E-02	1.03E-01
**ATPAF1**	-0.20	0.87	2.83E-02	1.03E-01
**RP11-425L10.1**	-0.24	0.84	2.83E-02	1.03E-01
**THOC1**	0.25	1.19	2.84E-02	1.03E-01
**EHD1**	0.19	1.14	2.84E-02	1.03E-01
**SMIM10L2B**	-0.39	0.76	2.84E-02	1.03E-01
**PLIN3**	0.16	1.12	2.84E-02	1.04E-01
**ABCC5**	0.19	1.14	2.85E-02	1.04E-01
**KPNA2**	-0.13	0.91	2.85E-02	1.04E-01
**RP11-404P21.9**	0.26	1.20	2.85E-02	NA
**OASL**	-0.37	0.77	2.86E-02	1.04E-01
**C1QL4**	-0.20	0.87	2.86E-02	NA
**NDUFB10**	-0.17	0.89	2.86E-02	1.04E-01
**NUDT16L1**	-0.29	0.82	2.86E-02	1.04E-01
**LRRC20**	-0.30	0.81	2.86E-02	1.04E-01
**FADS6**	-0.27	0.83	2.86E-02	NA
**TCTN1**	-0.28	0.82	2.86E-02	1.04E-01
**TRPC6**	-0.41	0.75	2.86E-02	1.04E-01
**PLSCR1**	0.17	1.13	2.86E-02	1.04E-01
**AC092066.1**	0.33	1.26	2.87E-02	1.04E-01
**SLC9A7**	0.21	1.16	2.87E-02	1.04E-01
**GJA1**	0.15	1.11	2.87E-02	1.04E-01
**CAPZA2**	0.19	1.14	2.88E-02	1.04E-01
**PRPS2**	-0.19	0.88	2.88E-02	1.04E-01
**CNTN1**	-0.17	0.89	2.89E-02	1.05E-01
**ATF3**	0.41	1.33	2.89E-02	1.05E-01
**CMBL**	-0.37	0.77	2.89E-02	1.05E-01
**STS**	-0.33	0.79	2.90E-02	1.05E-01
**LSS**	-0.15	0.90	2.90E-02	1.05E-01
**LYRM4**	0.21	1.16	2.90E-02	1.05E-01
**ZNF28**	0.31	1.24	2.91E-02	1.05E-01
**PPWD1**	0.22	1.17	2.91E-02	1.05E-01
**FAM81A**	-0.36	0.78	2.91E-02	1.05E-01
**NADSYN1**	0.19	1.14	2.91E-02	1.05E-01
**FAM69A**	0.26	1.20	2.92E-02	1.05E-01
**GPI**	-0.14	0.91	2.92E-02	1.05E-01
**LRFN1**	-0.31	0.81	2.92E-02	NA
**PPP2R1B**	0.16	1.12	2.92E-02	1.06E-01
**CLCA4**	-0.37	0.77	2.93E-02	1.06E-01
**CHCHD2**	-0.15	0.90	2.93E-02	1.06E-01
**COX15**	-0.21	0.87	2.93E-02	1.06E-01
**CNOT3**	0.18	1.14	2.93E-02	1.06E-01
**CPSF7**	0.15	1.11	2.93E-02	1.06E-01
**RNF10**	0.15	1.11	2.93E-02	1.06E-01
**ZNF417**	-0.41	0.75	2.94E-02	1.06E-01
**MINCR**	-0.38	0.77	2.95E-02	1.06E-01
**PARG**	0.26	1.20	2.95E-02	1.06E-01
**AHI1**	0.30	1.23	2.96E-02	1.06E-01
**NRIP1**	0.20	1.15	2.96E-02	1.06E-01
**PPARG**	-0.40	0.76	2.96E-02	1.06E-01
**ATP6V1A**	-0.18	0.88	2.96E-02	1.06E-01
**SLC39A8**	0.19	1.14	2.96E-02	1.06E-01
**NLRP3P**	0.40	1.32	2.96E-02	1.07E-01
**ASPRV1**	-0.34	0.79	2.96E-02	1.07E-01
**RANBP2**	0.18	1.13	2.97E-02	1.07E-01
**TRMT10B**	0.32	1.25	2.97E-02	1.07E-01
**PTP4A3**	-0.38	0.77	2.98E-02	1.07E-01
**CDKN2B**	-0.28	0.82	2.98E-02	1.07E-01
**GCNT2**	-0.33	0.79	2.98E-02	1.07E-01
**RAB8B**	0.28	1.22	2.98E-02	1.07E-01
**MRPS30**	0.22	1.17	1.38E-02	4.98E-02
**AC016747.3**	-0.43	0.74	1.38E-02	4.98E-02
**POU2F2**	-0.81	0.57	1.38E-02	4.98E-02
**TARSL2**	0.33	1.26	1.38E-02	4.98E-02
**SBF1**	-0.17	0.89	1.38E-02	4.99E-02
**CHMP4B**	0.18	1.14	1.39E-02	5.01E-02
**GPX4**	0.18	1.13	1.39E-02	5.01E-02
**IRS1**	-0.20	0.87	1.39E-02	5.02E-02
**CHST4**	2.36	5.13	1.39E-02	NA
**NOCT**	-0.31	0.81	1.39E-02	5.03E-02
**ALDH1A1**	-0.43	0.74	1.40E-02	5.04E-02
**ZNF286A**	0.39	1.31	1.40E-02	5.04E-02
**LLGL1**	0.20	1.15	1.40E-02	5.04E-02
**RRP36**	0.20	1.15	1.40E-02	5.04E-02
**MPP5**	0.19	1.14	1.40E-02	5.05E-02
**CRACR2B**	0.93	1.91	1.40E-02	5.05E-02
**ACOT7**	0.19	1.14	1.40E-02	5.05E-02
**CHAC1**	0.92	1.89	1.40E-02	5.05E-02
**NAP1L5**	0.61	1.53	1.40E-02	5.06E-02
**SLC31A1**	0.19	1.14	1.40E-02	5.06E-02
**RCOR2**	0.81	1.76	1.41E-02	5.06E-02
**TMED10**	0.17	1.12	1.41E-02	5.07E-02
**CABLES1**	0.89	1.86	1.41E-02	5.09E-02
**GDI1**	0.18	1.14	1.41E-02	5.09E-02
**SLC25A37**	0.23	1.17	1.41E-02	5.09E-02
**DHFR**	0.31	1.24	1.41E-02	5.09E-02
**SCAP**	-0.18	0.88	1.41E-02	5.09E-02
**LGALS1**	-0.18	0.88	1.42E-02	5.09E-02
**LINC00571**	-2.52	0.17	1.42E-02	NA
**SLC2A11**	-0.47	0.72	1.42E-02	5.10E-02
**AATF**	0.19	1.14	1.42E-02	5.10E-02
**PPP5C**	0.20	1.15	1.42E-02	5.12E-02
**SUCLG2**	0.20	1.15	1.42E-02	5.12E-02
**PPL**	-0.20	0.87	1.43E-02	5.12E-02
**TMEM41B**	0.20	1.15	1.43E-02	5.13E-02
**ZNF644**	0.22	1.16	1.43E-02	5.13E-02
**PDAP1**	0.17	1.13	1.43E-02	5.13E-02
**FLNC**	0.58	1.50	1.43E-02	5.13E-02
**DZIP1**	0.45	1.37	1.43E-02	5.13E-02
**DUSP13**	-0.83	0.56	1.43E-02	5.13E-02
**TTC26**	0.33	1.26	1.44E-02	5.14E-02
**ZNF699**	-0.53	0.69	1.44E-02	5.14E-02
**DOK4**	0.23	1.17	1.44E-02	5.15E-02
**KLF4**	-0.21	0.87	1.44E-02	5.16E-02
**ATAD3B**	0.20	1.15	1.44E-02	5.16E-02
**PLXNB3**	-0.28	0.83	1.44E-02	5.16E-02
**MYL6**	-0.16	0.90	1.44E-02	5.16E-02
**CAPZA1**	0.16	1.12	1.44E-02	5.16E-02
**GAS5**	-0.19	0.88	1.44E-02	5.16E-02
**ZGRF1**	0.29	1.22	1.44E-02	5.16E-02
**RP11-574K11.24**	-0.57	0.67	1.45E-02	5.17E-02
**MFN1**	-0.20	0.87	1.45E-02	5.17E-02
**NREP**	-0.67	0.63	1.45E-02	5.17E-02
**UBA5**	0.22	1.16	1.45E-02	5.18E-02
**SHKBP1**	-0.20	0.87	1.45E-02	5.18E-02
**FASTKD3**	0.37	1.29	1.46E-02	5.20E-02
**C1RL**	-0.31	0.80	1.46E-02	5.20E-02
**TMEM167A**	0.19	1.14	1.46E-02	5.20E-02
**EMP2**	0.19	1.14	1.46E-02	5.21E-02
**FUK**	-0.25	0.84	1.46E-02	5.21E-02
**CALML3**	-0.76	0.59	1.46E-02	5.21E-02
**KPNA5**	0.44	1.35	1.46E-02	5.21E-02
**BCORL1**	-0.28	0.82	1.47E-02	5.22E-02
**FABP5P7**	-0.42	0.75	1.47E-02	5.22E-02
**BID**	0.23	1.17	1.47E-02	5.23E-02
**IQGAP3**	0.27	1.20	1.47E-02	5.24E-02
**CACNG4**	0.76	1.69	1.47E-02	5.24E-02
**YWHAG**	0.16	1.12	1.48E-02	5.25E-02
**EMP3**	0.38	1.30	1.48E-02	5.25E-02
**MYCBP**	0.27	1.20	1.48E-02	5.25E-02
**GPATCH3**	0.34	1.26	1.48E-02	5.25E-02
**C5orf24**	0.23	1.17	1.48E-02	5.25E-02
**HIC1**	-1.01	0.50	1.48E-02	5.25E-02
**PLEKHM3**	-0.50	0.71	1.48E-02	5.26E-02
**WARS**	0.19	1.14	1.48E-02	5.27E-02
**PCNP**	-0.20	0.87	1.49E-02	5.28E-02
**DHRS4-AS1**	-0.30	0.81	1.49E-02	5.29E-02
**KDELC1**	0.38	1.30	1.49E-02	5.29E-02
**MYO18A**	-0.17	0.89	1.49E-02	5.29E-02
**PRSS27**	-0.31	0.80	1.49E-02	5.29E-02
**GLIPR1**	-0.69	0.62	1.49E-02	5.29E-02
**XRCC5**	0.17	1.12	1.49E-02	5.29E-02
**FGFR4**	0.47	1.39	1.50E-02	5.31E-02
**PRSS16**	-0.40	0.76	1.50E-02	5.31E-02
**SLC6A14**	-0.24	0.84	1.50E-02	5.32E-02
**MAGT1**	0.18	1.14	1.50E-02	5.33E-02
**GLYCTK**	-0.55	0.68	1.51E-02	5.35E-02
**LRCH4**	-0.34	0.79	1.51E-02	5.35E-02
**AP2A1**	-0.17	0.89	1.51E-02	5.35E-02
**FNBP4**	0.18	1.14	1.52E-02	5.36E-02

CHD1	-0.20	0.87	2.99E-02	1.07E-01
LCMT1	0.21	1.16	2.99E-02	1.07E-01
YBX1	-0.12	0.92	2.99E-02	1.07E-01
FAM50B	-0.34	0.79	3.00E-02	1.08E-01
FLOT2	-0.17	0.89	3.00E-02	1.08E-01
USP54	-0.22	0.86	3.00E-02	1.08E-01
ZFX	0.27	1.20	3.00E-02	1.08E-01
CALU	0.14	1.10	3.00E-02	1.08E-01
NHLRC3	-0.32	0.80	3.01E-02	1.08E-01
SMC5	0.20	1.15	3.01E-02	1.08E-01
ENC1	0.19	1.14	3.01E-02	1.08E-01
PLA2G4E-AS1	-0.35	0.78	3.02E-02	1.08E-01
RAC1P2	-0.39	0.76	3.02E-02	1.08E-01
RFTN1	0.19	1.14	3.02E-02	1.08E-01
NOP16	0.17	1.13	3.03E-02	1.08E-01
TMEM134	-0.23	0.85	3.03E-02	1.08E-01
KCNK15	-0.40	0.76	3.03E-02	1.08E-01
SHROOM1	-0.40	0.76	3.03E-02	1.09E-01
ZMYM3	-0.20	0.87	3.04E-02	1.09E-01
LAT2	-0.40	0.76	3.04E-02	1.09E-01
RP5-1014D13.2	0.35	1.28	3.05E-02	1.09E-01
CDK5	-0.29	0.82	3.05E-02	1.09E-01
SULT1B1	-0.31	0.81	3.05E-02	NA
PGP	-0.23	0.85	3.05E-02	1.09E-01
ANK2	-0.40	0.76	3.05E-02	1.09E-01
ZNF720	0.29	1.22	3.06E-02	1.09E-01
MTMR6	0.20	1.15	3.07E-02	1.09E-01
BOLA3	-0.23	0.85	3.07E-02	1.09E-01
LRP4	-0.28	0.82	3.08E-02	1.10E-01
HIST1H4I	0.39	1.31	3.08E-02	1.10E-01
GNG2	-0.40	0.76	3.08E-02	1.10E-01
CLIC1	-0.13	0.91	3.09E-02	1.10E-01
CRTC3	0.22	1.16	3.09E-02	1.10E-01
TIPARP	0.17	1.12	3.09E-02	1.10E-01
CASC4	0.22	1.17	3.10E-02	1.10E-01
ARMCX3	0.25	1.19	3.10E-02	1.10E-01
FAM162A	-0.18	0.88	3.11E-02	1.11E-01
RASL11A	-0.40	0.76	3.11E-02	1.11E-01
ATP2A1-AS1	-0.32	0.80	3.11E-02	NA
CEBPA	-0.31	0.81	3.11E-02	NA
ZNF627	-0.32	0.80	3.11E-02	1.11E-01
SPTLC1	0.17	1.12	3.12E-02	1.11E-01
S100A14	-0.12	0.92	3.12E-02	1.11E-01
FAM63A	-0.20	0.87	3.12E-02	1.11E-01
WDHD1	-0.19	0.88	3.13E-02	1.11E-01
CTD-2619J13.14	-0.35	0.79	3.13E-02	1.11E-01
CYP27B1	0.19	1.14	3.14E-02	1.11E-01
GLI2	-0.27	0.83	3.14E-02	NA
CACTIN	0.22	1.16	3.14E-02	1.12E-01
TMX1	0.20	1.15	3.15E-02	1.12E-01
BNIPL	-0.23	0.85	3.15E-02	1.12E-01
IRX5	-0.29	0.82	3.15E-02	1.12E-01
ATRN	0.14	1.10	3.15E-02	1.12E-01
FAM83A	0.13	1.09	3.15E-02	1.12E-01
CNTRL	0.21	1.15	3.15E-02	1.12E-01
TOR1AIP1	0.18	1.13	3.16E-02	1.12E-01
CMTM6	0.19	1.14	3.16E-02	1.12E-01
RPL41	-0.33	0.79	3.16E-02	1.12E-01
BTBD9	0.24	1.18	3.17E-02	1.12E-01
ERCC6L2	0.27	1.21	3.17E-02	1.12E-01
CEP70	0.32	1.25	3.17E-02	1.12E-01
LRRC4	-0.36	0.78	3.17E-02	1.12E-01
ARID4B	0.23	1.17	3.19E-02	1.13E-01
BRD4	0.18	1.13	3.19E-02	1.13E-01
HLF	-0.27	0.83	3.20E-02	NA
FAM214B	0.18	1.13	3.20E-02	1.13E-01
RAB4A	0.23	1.17	3.21E-02	1.13E-01
SMIM22	-0.40	0.76	3.21E-02	1.14E-01
PKNOX1	-0.24	0.85	3.22E-02	1.14E-01
DLX6	-0.30	0.81	3.22E-02	NA
PRSS16	-0.32	0.80	3.22E-02	1.14E-01
RPUSD2	-0.27	0.83	3.22E-02	1.14E-01
TMPRSS13	-0.26	0.84	3.22E-02	1.14E-01
TRIM4	0.20	1.15	3.22E-02	1.14E-01
DHFRL1	-0.32	0.80	3.23E-02	1.14E-01
ZFP36	-0.18	0.88	3.23E-02	1.14E-01
SKA3	0.20	1.15	3.24E-02	1.14E-01
ZBTB24	-0.28	0.83	3.24E-02	1.14E-01
GID8	-0.16	0.90	3.24E-02	1.14E-01
SGSM1	-0.37	0.77	3.24E-02	1.14E-01
LINC00672	0.30	1.23	3.25E-02	NA
PRR14	-0.20	0.87	3.25E-02	1.14E-01
RP11-30P6.6	0.40	1.32	3.25E-02	1.14E-01
GTDC1	0.27	1.20	3.25E-02	1.15E-01
CUL5	0.23	1.17	3.25E-02	1.15E-01
MTSS1L	-0.20	0.87	3.26E-02	1.15E-01
C8orf82	-0.34	0.79	3.26E-02	1.15E-01
TICAM1	0.20	1.15	3.26E-02	1.15E-01
GAS1	-0.40	0.76	3.26E-02	1.15E-01
ANKIB1	0.19	1.14	3.27E-02	1.15E-01
AFF1	-0.21	0.86	1.52E-02	5.37E-02
GNG5	0.20	1.15	1.52E-02	5.37E-02
RWDD4	0.26	1.20	1.52E-02	5.37E-02
PYROXD1	0.28	1.22	1.53E-02	5.39E-02
PIM2	0.31	1.24	1.53E-02	5.40E-02
IFI27L2	-0.21	0.86	1.53E-02	5.40E-02
HLA-B	0.19	1.14	1.53E-02	5.41E-02
COBLL1	-0.23	0.85	1.54E-02	5.42E-02
C7orf49	-0.29	0.82	1.54E-02	5.42E-02
ADM2	0.50	1.41	1.54E-02	5.42E-02
BRD3	0.23	1.17	1.54E-02	5.42E-02
ZNF507	0.24	1.18	1.54E-02	5.43E-02
NUDT1	0.28	1.22	1.54E-02	5.43E-02
ARFIP1	0.22	1.17	1.54E-02	5.43E-02
PTDSS1	0.17	1.12	1.54E-02	5.43E-02
RP13-554M15.7	0.76	1.69	1.54E-02	5.43E-02
THUMPD2	-0.33	0.79	1.54E-02	5.43E-02
TMEM65	0.26	1.19	1.55E-02	5.45E-02
MTHFD1L	0.24	1.18	1.55E-02	5.45E-02
ARMCX6	-0.38	0.77	1.55E-02	5.45E-02
WTAP	0.21	1.15	1.55E-02	5.45E-02
WNT3	-0.42	0.75	1.55E-02	5.45E-02
C9orf69	0.21	1.16	1.55E-02	5.46E-02
NUP37	0.25	1.19	1.56E-02	5.48E-02
ARHGAP33	0.41	1.32	1.56E-02	5.49E-02
ADAMTS13	-0.52	0.70	1.56E-02	5.49E-02
TIPARP	-0.20	0.87	1.56E-02	5.49E-02
NDOR1	-0.23	0.85	1.57E-02	5.51E-02
KIAA0922	0.25	1.19	1.57E-02	5.51E-02
ATP11B	-0.19	0.88	1.57E-02	5.51E-02
HDAC2	0.17	1.13	1.57E-02	5.51E-02
CCDC85C	0.19	1.14	1.57E-02	5.51E-02
POLR3A	-0.21	0.86	1.57E-02	5.51E-02
GINM1	0.26	1.20	1.57E-02	5.51E-02
RP11-379B18.5	-0.63	0.65	1.57E-02	5.51E-02
MRPS23	0.22	1.16	1.57E-02	5.51E-02
SPRED2	-0.23	0.85	1.57E-02	5.51E-02
HOXA1	-0.33	0.79	1.57E-02	5.52E-02
SGSH	-0.27	0.83	1.58E-02	5.54E-02
HDAC9	0.55	1.47	1.58E-02	5.54E-02
TPMT	-0.21	0.86	1.58E-02	5.54E-02
VSTM5	2.13	4.38	1.59E-02	NA
MRFAP1	-0.16	0.89	1.59E-02	5.57E-02
IL36G	-0.65	0.64	1.60E-02	5.60E-02
CD6	1.38	2.61	1.60E-02	NA
TBC1D25	-0.26	0.83	1.60E-02	5.61E-02
RP11-539I5.1	-0.46	0.72	1.61E-02	5.62E-02
RBM42	0.21	1.15	1.61E-02	5.62E-02
TRIM59	0.29	1.22	1.61E-02	5.63E-02
APBA1	-0.69	0.62	1.61E-02	5.63E-02
INAFM2	-0.44	0.74	1.61E-02	5.63E-02
LMF1	-0.50	0.71	1.61E-02	5.64E-02
MAMLD1	-0.53	0.69	1.62E-02	5.64E-02
DPYSL3	0.68	1.60	1.62E-02	5.65E-02
FAM157A	-0.59	0.66	1.62E-02	5.65E-02
NKTR	-0.18	0.88	1.62E-02	5.67E-02
COPS3	0.19	1.14	1.63E-02	5.69E-02
CCDC51	0.22	1.17	1.63E-02	5.69E-02
ECM2	0.83	1.77	1.63E-02	5.70E-02
MUC15	-0.31	0.81	1.64E-02	5.71E-02
SLC27A1	-0.33	0.79	1.64E-02	5.72E-02
ZNF880	-0.54	0.69	1.64E-02	5.72E-02
KCTD17	0.28	1.22	1.64E-02	5.73E-02
MBOAT7	-0.18	0.88	1.65E-02	5.73E-02
FAM207A	0.26	1.20	1.65E-02	5.74E-02
RNF7	-0.20	0.87	1.65E-02	5.75E-02
SCAI	0.36	1.28	1.65E-02	5.75E-02
NKX1-2	-0.62	0.65	1.65E-02	5.75E-02
STK24	0.18	1.13	1.65E-02	5.75E-02
ENTHD2	-0.36	0.78	1.65E-02	5.75E-02
PANK3	0.18	1.13	1.65E-02	5.75E-02
AVEN	0.27	1.20	1.65E-02	5.75E-02
RPN2	0.17	1.12	1.66E-02	5.76E-02
TSPAN31	-0.26	0.84	1.66E-02	5.77E-02
PLEKHA4	0.28	1.22	1.66E-02	5.78E-02
ZNF76	0.24	1.18	1.67E-02	5.78E-02
RP11-420A23.1	-0.63	0.65	1.67E-02	5.80E-02
ITGB1	0.16	1.12	1.67E-02	5.80E-02
GPR161	0.23	1.17	1.67E-02	5.80E-02
RUVBL1	0.18	1.13	1.68E-02	5.81E-02
DNER	0.72	1.64	1.68E-02	5.82E-02
MYO15B	-0.85	0.56	1.68E-02	5.82E-02
NEK1	0.26	1.20	1.68E-02	5.82E-02
MYH10	-0.18	0.88	1.68E-02	5.82E-02
TOLLIP	-0.20	0.87	1.68E-02	5.83E-02
HOXC8	0.55	1.46	1.69E-02	5.84E-02
MTERF2	-0.38	0.77	1.69E-02	5.85E-02
ZNF876P	0.78	1.72	1.69E-02	5.85E-02
PPP1R15A	-0.26	0.84	1.69E-02	5.86E-02
TCF4	-0.24	0.84	1.69E-02	5.86E-02

EPN2	0.19	1.14	3.27E-02	1.15E-01
ABCA3	0.40	1.32	3.27E-02	1.15E-01
SMIM15	0.21	1.16	3.28E-02	1.15E-01
CDC23	0.19	1.14	3.29E-02	1.15E-01
WDR11	0.16	1.12	3.29E-02	1.15E-01
SLC38A10	0.19	1.14	3.31E-02	1.16E-01
EHHADH	-0.23	0.85	3.31E-02	1.16E-01
BCL2L12	0.24	1.18	3.31E-02	1.16E-01
TM9SF4	0.17	1.12	3.31E-02	1.16E-01
TMEM52	-0.35	0.78	3.34E-02	1.17E-01
ABHD17A	-0.20	0.87	3.34E-02	1.17E-01
ENDOG	-0.39	0.76	3.34E-02	1.17E-01
GOSR1	0.17	1.12	3.35E-02	1.17E-01
F11R	0.17	1.13	3.35E-02	1.17E-01
RP11-696N14.1	0.40	1.32	3.36E-02	1.18E-01
POMP	0.18	1.13	3.36E-02	1.18E-01
ZNF337-AS1	-0.32	0.80	3.36E-02	NA
STXBP3	0.25	1.19	3.37E-02	1.18E-01
NDUFB4	-0.17	0.89	3.37E-02	1.18E-01
STK40	0.20	1.15	3.37E-02	1.18E-01
AP1S3	0.19	1.14	3.38E-02	1.18E-01
ZC3HC1	0.24	1.18	3.38E-02	1.18E-01
CREM	-0.31	0.81	3.38E-02	1.18E-01
KLK1	-0.38	0.77	3.40E-02	1.19E-01
TMEM238	-0.40	0.76	3.40E-02	1.19E-01
SIPA1L2	-0.23	0.85	3.40E-02	1.19E-01
ICAM1	0.39	1.31	3.41E-02	1.19E-01
FAM193B	0.22	1.16	3.41E-02	1.19E-01
CCDC71	-0.22	0.86	3.41E-02	1.19E-01
APPL1	0.17	1.13	3.42E-02	1.19E-01
VEGFB	-0.25	0.84	3.42E-02	1.19E-01
DOCK11	-0.24	0.85	3.42E-02	1.19E-01
C2orf44	0.26	1.20	3.44E-02	1.20E-01
SETD1A	0.19	1.14	3.44E-02	1.20E-01
CIRH1A	0.14	1.10	3.44E-02	1.20E-01
PRPF40A	0.16	1.12	3.45E-02	1.20E-01
CEACAM19	-0.29	0.82	3.45E-02	1.20E-01
GLUD1	-0.15	0.90	3.46E-02	1.20E-01
PIK3IP1	-0.37	0.77	3.46E-02	1.20E-01
ATL1	-0.39	0.76	3.46E-02	1.20E-01
PDCD6	0.16	1.12	3.46E-02	1.20E-01
FNTA	0.22	1.17	3.46E-02	1.20E-01
KIDINS220	0.16	1.12	3.47E-02	1.21E-01
FAM120A	0.12	1.09	3.47E-02	1.21E-01
SLC25A3	-0.12	0.92	3.47E-02	1.21E-01
EAF1	0.22	1.16	3.48E-02	1.21E-01
INAFM2	0.37	1.29	3.48E-02	1.21E-01
TSPAN10	-0.32	0.80	3.48E-02	NA
ZNF839	0.31	1.24	3.48E-02	1.21E-01
ZNF197	0.23	1.17	3.48E-02	1.21E-01
XXbac-B135H6.15	-0.23	0.85	3.49E-02	NA
SCNN1G	-0.37	0.77	3.49E-02	1.21E-01
42799	0.20	1.15	3.49E-02	1.21E-01
HMOX2	0.14	1.10	3.49E-02	1.21E-01
MAP3K11	0.20	1.15	3.49E-02	1.21E-01
RAB40B	-0.32	0.80	3.50E-02	1.21E-01
ZNF317	0.19	1.14	3.50E-02	1.21E-01
LINC01405	-0.39	0.76	3.51E-02	1.22E-01
SLC15A1	-0.32	0.80	3.51E-02	1.22E-01
ADAMTS1	-0.20	0.87	3.52E-02	1.22E-01
ZDHHC24	-0.26	0.83	3.52E-02	1.22E-01
RP11-471B22.2	-0.19	0.88	3.52E-02	NA
TRIM2	-0.21	0.86	3.53E-02	1.22E-01
MCPH1	0.24	1.18	3.53E-02	1.22E-01
ODF3B	-0.39	0.76	3.53E-02	1.22E-01
COL13A1	-0.39	0.76	3.53E-02	1.22E-01
METAP2	0.17	1.12	3.53E-02	1.22E-01
H2AFV	0.17	1.12	3.54E-02	1.22E-01
PHF8	-0.19	0.88	3.54E-02	1.22E-01
MLKL	-0.24	0.84	3.54E-02	1.22E-01
HIRA	0.26	1.20	3.54E-02	1.22E-01
MFNG	0.38	1.31	3.54E-02	1.22E-01
HTRA2	-0.27	0.83	3.55E-02	1.22E-01
FAM122B	0.20	1.15	3.55E-02	1.22E-01
ZNF440	-0.31	0.81	3.55E-02	1.23E-01
FBXL20	-0.32	0.80	3.55E-02	1.23E-01
RNF138	0.25	1.19	3.55E-02	1.23E-01
MAMSTR	-0.39	0.76	3.56E-02	1.23E-01
KLHL8	0.24	1.18	3.56E-02	1.23E-01
RP11-499P20.2	0.32	1.25	3.57E-02	NA
MGAT4A	0.37	1.30	3.57E-02	1.23E-01
URI1	0.16	1.12	3.58E-02	1.23E-01
SLC5A6	-0.18	0.88	3.58E-02	1.23E-01
SH3YL1	0.18	1.14	3.58E-02	1.23E-01
GDPD3	-0.33	0.79	3.58E-02	1.23E-01
MTFP1	-0.39	0.76	3.58E-02	1.23E-01
SPRYD7	0.21	1.16	3.58E-02	1.23E-01
WBP4	0.22	1.16	3.59E-02	1.23E-01
STYXL1	-0.18	0.88	3.59E-02	1.24E-01
SMARCA5	0.17	1.12	3.59E-02	1.24E-01

FHL1	0.23	1.17	1.69E-02	5.86E-02
XYLT2	0.19	1.14	1.70E-02	5.86E-02
TRIM29	-0.18	0.88	1.70E-02	5.86E-02
KDM3B	0.17	1.13	1.70E-02	5.87E-02
UBL4A	0.21	1.16	1.70E-02	5.88E-02
SDF4	0.18	1.13	1.70E-02	5.89E-02
PERM1	-0.52	0.70	1.71E-02	5.89E-02
RIC8A	-0.17	0.89	1.71E-02	5.91E-02
CRELD2	0.23	1.18	1.71E-02	5.91E-02
ABCB7	0.24	1.18	1.71E-02	5.91E-02
PAGR1	-0.69	0.62	1.72E-02	5.92E-02
TYSND1	-0.20	0.87	1.72E-02	5.92E-02
AGGF1	0.21	1.16	1.72E-02	5.93E-02
LRP3	-0.30	0.81	1.72E-02	5.94E-02
MRPL23	0.21	1.16	1.73E-02	5.95E-02
PNPLA6	0.18	1.13	1.73E-02	5.95E-02
SAAL1	0.27	1.21	1.73E-02	5.96E-02
COL4A5	-0.17	0.89	1.73E-02	5.96E-02
BET1L	-0.25	0.84	1.73E-02	5.96E-02
LSM3	0.22	1.16	1.73E-02	5.97E-02
KIAA0319L	-0.19	0.88	1.74E-02	5.98E-02
C12orf43	0.26	1.20	1.74E-02	5.98E-02
WSB1	-0.18	0.88	1.74E-02	5.99E-02
MAFF	-0.34	0.79	1.74E-02	5.99E-02
ZNF90	0.51	1.43	1.74E-02	5.99E-02
ZNF577	-0.40	0.76	1.74E-02	5.99E-02
PEX5	0.23	1.17	1.75E-02	6.00E-02
FAM127C	-0.32	0.80	1.75E-02	6.00E-02
LRRC59	0.18	1.13	1.75E-02	6.00E-02
ACCS	-0.39	0.77	1.75E-02	6.00E-02
ERGIC1	0.16	1.12	1.75E-02	6.01E-02
GALNT10	0.20	1.15	1.75E-02	6.01E-02
NTN1	-1.10	0.47	1.75E-02	NA
JMY	0.26	1.20	1.76E-02	6.02E-02
GMPR	0.99	1.98	1.76E-02	6.03E-02
ANXA9	-0.35	0.78	1.76E-02	6.04E-02
CDH24	-0.28	0.82	1.76E-02	6.04E-02
RFWD2	0.21	1.16	1.77E-02	6.05E-02
RASA2	0.28	1.22	1.77E-02	6.06E-02
NOL11	0.19	1.14	1.77E-02	6.06E-02
SHMT1	0.20	1.14	1.78E-02	6.10E-02
GNPTG	-0.24	0.85	1.78E-02	6.10E-02
RP11-887P2.3	-1.29	0.41	1.79E-02	NA
MON1B	-0.20	0.87	1.79E-02	6.11E-02
ZFAND5	0.19	1.14	1.79E-02	6.11E-02
ZNF449	-0.39	0.76	1.79E-02	6.11E-02
FBXW12	-0.83	0.56	1.79E-02	6.12E-02
ZNF793	-0.34	0.79	1.79E-02	6.12E-02
ZNF629	-0.20	0.87	1.79E-02	6.13E-02
ST3GAL5	0.35	1.27	1.79E-02	6.13E-02
RGPD8	0.65	1.57	1.79E-02	6.13E-02
LRRC8A	-0.16	0.89	1.80E-02	6.15E-02
LINC01451	-0.60	0.66	1.80E-02	6.15E-02
SORCS2	-0.48	0.72	1.80E-02	6.15E-02
ASCL2	1.11	2.16	1.80E-02	NA
GEM	1.26	2.40	1.81E-02	NA
DNAJB9	0.33	1.26	1.81E-02	6.16E-02
DKK3	-0.16	0.89	1.81E-02	6.18E-02
TSPAN18	2.08	4.23	1.81E-02	NA
ZNF580	-0.33	0.80	1.81E-02	6.18E-02
AAGAB	0.18	1.13	1.82E-02	6.19E-02
PROSER1	0.19	1.14	1.82E-02	6.20E-02
VAMP8	-0.17	0.89	1.82E-02	6.20E-02
SHC1	-0.16	0.90	1.82E-02	6.20E-02
SKA3	0.28	1.22	1.82E-02	6.22E-02
CPTP	-0.23	0.85	1.83E-02	6.22E-02
NXPH4	0.50	1.41	1.83E-02	6.24E-02
CD164L2	-0.59	0.66	1.84E-02	6.25E-02
FKBP14	-0.22	0.86	1.84E-02	6.26E-02
ZNF416	-0.41	0.75	1.84E-02	6.27E-02
ERMAP	-0.43	0.74	1.84E-02	6.27E-02
FNDC3B	0.18	1.13	1.85E-02	6.28E-02
PLCD3	-0.23	0.85	1.85E-02	6.29E-02
IL20RB	-0.16	0.89	1.85E-02	6.29E-02
RP13-104F24.3	-0.59	0.66	1.85E-02	6.29E-02
ZFP36	0.19	1.14	1.86E-02	6.31E-02
QTRTD1	0.20	1.15	1.86E-02	6.31E-02
MTMR10	-0.23	0.85	1.86E-02	6.33E-02
BAG3	0.17	1.12	1.87E-02	6.33E-02
DYRK2	0.20	1.15	1.87E-02	6.33E-02
GLB1L2	0.35	1.27	1.87E-02	6.34E-02
MAP1B	0.61	1.53	1.87E-02	6.34E-02
SHOC2	0.19	1.14	1.87E-02	6.34E-02
SH3D21	-0.23	0.85	1.87E-02	6.34E-02
HDAC1	0.16	1.12	1.87E-02	6.34E-02
GTF2H1	0.19	1.14	1.87E-02	6.34E-02
TMEM248	0.18	1.13	1.87E-02	6.34E-02
INTS3	-0.18	0.89	1.87E-02	6.34E-02
ASF1B	0.27	1.20	1.87E-02	6.34E-02
AFAP1L2	0.20	1.15	1.88E-02	6.35E-02

SAV1	0.24	1.18	3.59E-02	1.24E-01
CDK16	0.15	1.11	3.60E-02	1.24E-01
TCN2	-0.39	0.76	3.61E-02	1.24E-01
PNMAL1	-0.25	0.84	3.61E-02	1.24E-01
MICU2	0.21	1.15	3.61E-02	1.24E-01
PABPC4L	-0.39	0.76	3.62E-02	1.24E-01
LRRC41	-0.16	0.90	3.62E-02	1.24E-01
ZSCAN5A	0.35	1.28	3.63E-02	1.25E-01
PSMD1	0.14	1.10	3.63E-02	1.25E-01
ERMAP	0.30	1.23	3.63E-02	1.25E-01
PRPF6	0.14	1.10	3.63E-02	1.25E-01
FAU	-0.15	0.90	3.63E-02	1.25E-01
MOCS1	-0.30	0.81	3.64E-02	1.25E-01
FAM161B	-0.38	0.77	3.64E-02	1.25E-01
PIP5KL1	-0.38	0.77	3.65E-02	1.25E-01
MAP2K1P1	-0.21	0.86	3.65E-02	NA
CD164L2	-0.39	0.76	3.65E-02	1.25E-01
NFKBID	-0.36	0.78	3.65E-02	1.25E-01
HN1	-0.14	0.91	3.66E-02	1.25E-01
CNOT2	0.17	1.12	3.66E-02	1.25E-01
CABLES2	-0.24	0.84	3.66E-02	1.25E-01
GJB3	0.15	1.11	3.66E-02	1.25E-01
CHRAC1	-0.20	0.87	3.67E-02	1.25E-01
ACVR1	0.24	1.18	3.67E-02	1.25E-01
SPATA6	-0.38	0.77	3.67E-02	1.25E-01
FUZ	-0.37	0.77	3.67E-02	1.25E-01
ARF4	0.13	1.10	3.67E-02	1.25E-01
FBXO10	0.39	1.31	3.67E-02	1.25E-01
HSPA4L	0.17	1.13	3.68E-02	1.26E-01
RP11-475C16.1	-0.28	0.83	3.68E-02	1.26E-01
FAM46B	-0.18	0.88	3.69E-02	1.26E-01
ZNF876P	0.39	1.31	3.69E-02	1.26E-01
PFKM	0.15	1.11	3.69E-02	1.26E-01
ACSL3	0.16	1.12	3.69E-02	1.26E-01
AC007038.7	0.19	1.14	3.70E-02	NA
S1PR1	0.25	1.19	3.71E-02	1.26E-01
RNF13	0.21	1.16	3.71E-02	1.26E-01
ATP8B1	0.18	1.13	3.73E-02	1.27E-01
FCHO2	0.21	1.16	3.73E-02	1.27E-01
KLF2	-0.24	0.84	3.74E-02	NA
FBXL6	-0.26	0.83	3.74E-02	1.27E-01
DPP7	-0.21	0.87	3.74E-02	1.27E-01
FAM126B	0.24	1.18	3.74E-02	1.27E-01
FES	-0.37	0.78	3.75E-02	1.28E-01
CRYBG3	0.21	1.16	3.76E-02	1.28E-01
MOCS3	-0.26	0.84	3.76E-02	1.28E-01
ZNF264	-0.25	0.84	3.77E-02	1.28E-01
WARS2	0.26	1.20	3.77E-02	1.28E-01
PSMA6	-0.32	0.80	3.77E-02	1.28E-01
GLTSCR1	-0.28	0.82	3.77E-02	1.28E-01
CEACAM7	-0.25	0.84	3.77E-02	NA
GFOD2	0.19	1.14	3.77E-02	1.28E-01
EDEM3	-0.19	0.87	3.77E-02	1.28E-01
RNF125	-0.37	0.77	3.79E-02	1.29E-01
TBC1D22B	0.22	1.17	3.79E-02	1.29E-01
ABCC10	0.22	1.16	3.79E-02	1.29E-01
LSM11	-0.32	0.80	3.80E-02	1.29E-01
SFXN5	-0.32	0.80	3.80E-02	1.29E-01
LINC00865	-0.26	0.83	3.81E-02	NA
WDR81	-0.22	0.86	3.82E-02	1.29E-01
FAM83H	-0.16	0.89	3.83E-02	1.30E-01
MAPK3	-0.17	0.89	3.83E-02	1.30E-01
LIX1L	-0.25	0.84	3.84E-02	1.30E-01
LRP12	0.24	1.18	3.84E-02	1.30E-01
THAP4	0.17	1.13	3.84E-02	1.30E-01
ZNF217	-0.18	0.88	3.84E-02	1.30E-01
BTBD7	0.18	1.13	3.84E-02	1.30E-01
RIOK1	0.19	1.14	3.85E-02	1.30E-01
NUP54	0.19	1.14	3.85E-02	1.30E-01
ZNF267	0.32	1.25	3.85E-02	1.30E-01
AC004381.6	-0.32	0.80	3.85E-02	1.30E-01
FAM46A	-0.35	0.78	3.85E-02	1.30E-01
ZSCAN9	-0.36	0.78	3.85E-02	1.30E-01
KIAA0753	0.21	1.15	3.86E-02	1.30E-01
DDA1	0.19	1.14	3.86E-02	1.30E-01
TTPAL	-0.15	0.90	3.86E-02	1.30E-01
STEAP3	-0.18	0.88	3.86E-02	1.31E-01
S100P	-0.15	0.90	3.87E-02	1.31E-01
CCDC80	-0.20	0.87	3.87E-02	1.31E-01
PSME3	0.12	1.09	3.87E-02	1.31E-01
COX5BP1	0.38	1.30	3.88E-02	1.31E-01
SRSF7	0.14	1.10	3.88E-02	1.31E-01
WFS1	-0.23	0.85	3.89E-02	1.31E-01
C9orf114	0.22	1.16	3.89E-02	1.31E-01
MAST3	-0.33	0.79	3.89E-02	1.31E-01
DGCR2	0.16	1.12	3.89E-02	1.31E-01
IKZF5	0.28	1.22	3.90E-02	1.31E-01
SREBF2	-0.12	0.92	3.90E-02	1.31E-01
ITPK1	0.15	1.11	3.90E-02	1.31E-01
RPS18	-0.13	0.92	3.91E-02	1.31E-01
NGDN	0.24	1.18	1.88E-02	6.35E-02
ARHGEF25	-1.55	0.34	1.88E-02	NA
AASS	-0.28	0.82	1.88E-02	6.35E-02
XPO7	0.17	1.12	1.88E-02	6.35E-02
CHI3L2	-1.08	0.47	1.88E-02	NA
PKNOX1	0.26	1.20	1.88E-02	6.37E-02
PROSER3	-0.31	0.81	1.89E-02	6.39E-02
UGGT1	0.18	1.13	1.89E-02	6.40E-02
SRP68	0.17	1.12	1.89E-02	6.40E-02
ZBTB43	-0.22	0.86	1.90E-02	6.41E-02
FAM110C	-0.22	0.86	1.91E-02	6.44E-02
RTCB	0.17	1.13	1.91E-02	6.45E-02
UBE2W	0.25	1.19	1.91E-02	6.45E-02
NPLOC4	-0.16	0.89	1.92E-02	6.47E-02
BOK	-0.18	0.88	1.92E-02	6.47E-02
G3BP1	0.16	1.11	1.92E-02	6.49E-02
DUX4L50	-0.56	0.68	1.93E-02	6.50E-02
OLA1	0.18	1.13	1.93E-02	6.50E-02
TRAPPC9	-0.24	0.85	1.93E-02	6.50E-02
FAM3A	-0.24	0.85	1.93E-02	6.50E-02
MID1IP1	0.19	1.14	1.93E-02	6.50E-02
CTC-518B2.8	0.38	1.30	1.93E-02	6.51E-02
ERN1	-0.27	0.83	1.94E-02	6.53E-02
AC093495.4	-0.72	0.61	1.94E-02	6.53E-02
UPP1	-0.19	0.88	1.94E-02	6.53E-02
C11orf57	0.22	1.17	1.94E-02	6.54E-02
FAM157C	-0.77	0.59	1.95E-02	6.54E-02
DDX39B	-0.19	0.88	1.95E-02	6.55E-02
JPX	-0.33	0.80	1.95E-02	6.55E-02
CRYAB	-0.24	0.85	1.95E-02	6.55E-02
QDPR	0.21	1.16	1.95E-02	6.57E-02
JMJD8	-0.31	0.81	1.96E-02	6.57E-02
KRAS	0.19	1.14	1.96E-02	6.57E-02
UBE2H	-0.16	0.90	1.96E-02	6.57E-02
KCNE4	-3.03	0.12	1.96E-02	NA
MVD	-0.16	0.90	1.96E-02	6.58E-02
ALDOB	-2.39	0.19	1.96E-02	NA
CCNT1	0.19	1.14	1.97E-02	6.60E-02
NAT10	0.18	1.14	1.97E-02	6.60E-02
SREK1IP1	0.21	1.16	1.97E-02	6.60E-02
SF3B4	-0.20	0.87	1.97E-02	6.60E-02
INTS7	0.20	1.15	1.97E-02	6.60E-02
AHCTF1	0.18	1.14	1.97E-02	6.61E-02
SIRT1	0.28	1.21	1.97E-02	6.61E-02
SLC38A1	0.15	1.11	1.98E-02	6.62E-02
WRB	0.25	1.19	1.98E-02	6.63E-02
SLC25A35	-0.40	0.76	1.98E-02	6.63E-02
RP11-554I8.2	0.36	1.29	1.99E-02	6.66E-02
ABCC1	-0.16	0.89	1.99E-02	6.66E-02
KDELC2	0.27	1.20	1.99E-02	6.67E-02
ACHE	-2.32	0.20	2.00E-02	NA
CYB561	-0.19	0.87	2.00E-02	6.70E-02
NOS1	-0.31	0.81	2.01E-02	6.72E-02
CLDN11	1.13	2.19	2.01E-02	NA
LSM5	0.23	1.17	2.01E-02	6.72E-02
CTNNA1	0.15	1.11	2.01E-02	6.73E-02
CD59	-0.18	0.88	2.01E-02	6.73E-02
MSX2	-0.49	0.71	2.01E-02	6.73E-02
KIAA0368	0.16	1.12	2.01E-02	6.73E-02
ANAPC1	0.20	1.15	2.02E-02	6.73E-02
RPUSD2	0.27	1.20	2.02E-02	6.75E-02
RP11-345P4.9	-0.87	0.55	2.02E-02	6.75E-02
CNOT10	0.22	1.16	2.04E-02	6.80E-02
NSF	0.20	1.15	2.04E-02	6.81E-02
SMAP2	-0.22	0.86	2.04E-02	6.82E-02
PKP1	-0.17	0.89	2.05E-02	6.82E-02
RPA3	0.27	1.20	2.05E-02	6.83E-02
CBARP	0.42	1.34	2.05E-02	6.83E-02
SPTLC3	-0.25	0.84	2.06E-02	6.85E-02
VEGFC	-0.34	0.79	2.06E-02	6.85E-02
VPS13B	-0.20	0.87	2.06E-02	6.86E-02
KPNA7	-0.99	0.50	2.06E-02	6.86E-02
KIAA0586	0.24	1.18	2.06E-02	6.86E-02
TAP1	-0.18	0.88	2.06E-02	6.87E-02
MPP6	0.26	1.20	2.07E-02	6.88E-02
ATE1	0.21	1.16	2.07E-02	6.88E-02
STPG1	-0.25	0.84	2.07E-02	6.88E-02
CD83	0.63	1.55	2.07E-02	6.88E-02
SDK2	-0.20	0.87	2.07E-02	6.88E-02
RP1-239B22.5	-0.68	0.63	2.07E-02	6.89E-02
PSMB3	0.17	1.13	2.09E-02	6.93E-02
PPP1R14A	0.59	1.50	2.09E-02	6.94E-02
LIPT2	1.03	2.04	2.09E-02	NA
AARS	0.17	1.13	2.10E-02	6.96E-02
SLC27A5	-0.47	0.72	2.10E-02	6.96E-02
MRPL37	0.18	1.13	2.10E-02	6.98E-02
PPP4R3B	0.17	1.12	2.10E-02	6.98E-02
KIAA0907	0.20	1.15	2.11E-02	6.98E-02
FAM49A	-0.39	0.76	2.11E-02	6.99E-02
MAST1	1.26	2.40	2.12E-02	NA

KIAA1958	0.31	1.24	3.91E-02	1.32E-01
PROCR	0.20	1.15	3.91E-02	1.32E-01
SNX13	0.20	1.15	3.92E-02	1.32E-01
LA16c-358B7.3	-0.36	0.78	3.92E-02	1.32E-01
GDF15	-0.37	0.77	3.92E-02	1.32E-01
MYCL	-0.26	0.83	3.93E-02	1.32E-01
TBCA	0.18	1.14	3.94E-02	1.32E-01
CPT2	-0.25	0.84	3.94E-02	1.32E-01
AJUBA	-0.14	0.91	3.94E-02	1.32E-01
MYRFL	-0.37	0.77	3.94E-02	1.32E-01
ZDHHC16	0.18	1.13	3.95E-02	1.33E-01
GCH1	-0.27	0.83	3.95E-02	1.33E-01
PTPN18	-0.20	0.87	3.95E-02	1.33E-01
BBS7	0.21	1.15	3.95E-02	1.33E-01
AMN	-0.38	0.77	3.95E-02	1.33E-01
LANCL2	0.23	1.17	3.95E-02	1.33E-01
WRB	-0.23	0.85	3.95E-02	1.33E-01
RSL24D1	0.20	1.15	3.96E-02	1.33E-01
NIPA1	0.22	1.16	3.96E-02	1.33E-01
HMGXB3	0.17	1.12	3.96E-02	1.33E-01
APRT	-0.19	0.88	3.98E-02	1.33E-01
MIR210HG	-0.32	0.80	3.98E-02	1.33E-01
RASA2	0.27	1.20	3.98E-02	1.33E-01
WDR5	0.15	1.11	3.99E-02	1.34E-01
ELOVL5	0.16	1.11	3.99E-02	1.34E-01
FKBP9P1	-0.36	0.78	4.00E-02	1.34E-01
RCC2	-0.12	0.92	4.01E-02	1.34E-01
LEF1	-0.37	0.77	4.01E-02	1.34E-01
HOXB7	-0.29	0.82	4.01E-02	NA
DLX2	-0.37	0.77	4.01E-02	1.34E-01
MNS1	-0.38	0.77	4.01E-02	1.34E-01
PDCD11	0.13	1.10	4.02E-02	1.34E-01
TTC5	-0.25	0.84	4.02E-02	1.34E-01
DGCR5	-0.38	0.77	4.02E-02	1.34E-01
JUP	-0.15	0.90	4.02E-02	1.34E-01
VPS13D	0.17	1.13	4.03E-02	1.34E-01
PRSS8	-0.16	0.89	4.04E-02	1.35E-01
ADORA2B	0.20	1.15	4.04E-02	1.35E-01
RNASEL	-0.28	0.82	4.04E-02	1.35E-01
MED6	0.24	1.18	4.05E-02	1.35E-01
CHMP6	-0.25	0.84	4.05E-02	1.35E-01
PSMD12	0.19	1.14	4.05E-02	1.35E-01
ZNF616	-0.30	0.81	4.06E-02	1.35E-01
KRT34	-0.38	0.77	4.06E-02	1.35E-01
MRPL45	0.18	1.13	4.06E-02	1.35E-01
DZIP1L	-0.34	0.79	4.06E-02	1.35E-01
STK36	-0.24	0.84	4.07E-02	1.35E-01
PHF13	-0.22	0.86	4.08E-02	1.36E-01
TMX2	0.16	1.12	4.08E-02	1.36E-01
GPR3	-0.38	0.77	4.09E-02	1.36E-01
USO1	0.16	1.12	4.09E-02	1.36E-01
PTGES	0.24	1.18	4.10E-02	1.36E-01
MAT2B	0.19	1.14	4.10E-02	1.36E-01
IGF2R	0.13	1.09	4.10E-02	1.36E-01
SLC10A7	-0.29	0.82	4.10E-02	1.36E-01
TANC1	-0.14	0.91	4.11E-02	1.36E-01
EMC2	0.21	1.16	4.11E-02	1.36E-01
RP11-500G22.5	-0.29	0.82	4.11E-02	NA
MCCC2	0.14	1.11	4.11E-02	1.36E-01
ZNF48	-0.27	0.83	4.11E-02	1.36E-01
SPCS1	-0.16	0.90	4.11E-02	1.36E-01
NINJ2	-0.32	0.80	4.11E-02	NA
TUBGCP2	0.16	1.11	4.12E-02	1.37E-01
DUSP14	0.17	1.12	4.12E-02	1.37E-01
AGPAT4	-0.31	0.81	4.12E-02	1.37E-01
ZNF18	-0.31	0.81	4.12E-02	1.37E-01
PDE7A	0.23	1.17	4.12E-02	1.37E-01
ZNF528	0.33	1.26	4.12E-02	1.37E-01
DUS1L	0.17	1.13	4.12E-02	1.37E-01
NAPEPLD	-0.32	0.80	4.13E-02	1.37E-01
BCAT1	-0.19	0.88	4.13E-02	1.37E-01
DMXL1	-0.21	0.86	4.13E-02	1.37E-01
CHMP5	0.18	1.13	4.13E-02	1.37E-01
CAMK2N1	-0.16	0.90	4.14E-02	1.37E-01
CDK5RAP2	0.14	1.11	4.15E-02	1.37E-01
KLHL28	0.30	1.23	4.15E-02	1.37E-01
42802	-0.23	0.85	4.15E-02	1.37E-01
FAM20C	-0.34	0.79	4.16E-02	1.37E-01
AL603965.1	0.37	1.30	4.17E-02	1.38E-01
JMJD4	-0.32	0.80	4.17E-02	1.38E-01
TXNL1	0.17	1.13	4.18E-02	1.38E-01
FBXW4P1	0.32	1.25	4.18E-02	NA
RBCK1	-0.15	0.90	4.19E-02	1.38E-01
BAD	-0.22	0.86	4.19E-02	1.38E-01
HAUS3	0.24	1.18	4.20E-02	1.39E-01
WBP5	-0.22	0.86	4.20E-02	1.39E-01
PLEKHB2	0.13	1.09	4.20E-02	1.39E-01
SNRPB2	0.19	1.14	4.21E-02	1.39E-01
SLC22A18	-0.35	0.78	4.21E-02	1.39E-01
ARL8A	-0.19	0.88	4.21E-02	1.39E-01
GPN3	0.27	1.20	2.12E-02	7.02E-02
RP3-395M20.3	-0.81	0.57	2.12E-02	7.04E-02
RPP25	0.49	1.40	2.13E-02	7.05E-02
POLB	0.27	1.20	2.13E-02	7.05E-02
ANKEF1	0.23	1.17	2.13E-02	7.05E-02
FSCN1	0.18	1.13	2.13E-02	7.05E-02
RANBP3	0.20	1.15	2.13E-02	7.06E-02
DHX30	0.17	1.12	2.13E-02	7.06E-02
COL5A2	-0.19	0.88	2.14E-02	7.09E-02
RAB25	-0.18	0.88	2.14E-02	7.10E-02
LRRC27	-0.43	0.74	2.15E-02	7.10E-02
PPP1R8	0.20	1.15	2.15E-02	7.10E-02
CLCN4	0.76	1.69	2.15E-02	7.10E-02
EFNB2	-0.16	0.89	2.15E-02	7.10E-02
AADACL2	-1.77	0.29	2.15E-02	NA
GPSM2	0.18	1.13	2.15E-02	7.12E-02
PPP1R13L	-0.16	0.89	2.15E-02	7.12E-02
GLRX2	-0.39	0.76	2.16E-02	7.12E-02
TMEM30A	0.16	1.12	2.17E-02	7.15E-02
TTC38	0.23	1.17	2.17E-02	7.16E-02
YPEL3	-0.29	0.82	2.17E-02	7.16E-02
ATP2C2	0.50	1.41	2.18E-02	7.18E-02
VBP1	0.21	1.15	2.18E-02	7.18E-02
POLI	-0.31	0.81	2.18E-02	7.19E-02
NUBPL	0.24	1.18	2.18E-02	7.19E-02
NATD1	-0.35	0.78	2.18E-02	7.19E-02
ARMC5	-0.31	0.81	2.19E-02	7.21E-02
RP11-274B21.3	-0.37	0.78	2.19E-02	7.21E-02
TDP1	0.23	1.17	2.19E-02	7.21E-02
TRMT6	0.22	1.17	2.19E-02	7.22E-02
NEDD1	0.20	1.15	2.19E-02	7.22E-02
ZYG11A	0.43	1.34	2.20E-02	7.23E-02
PCNXL4	-0.21	0.86	2.20E-02	7.23E-02
LYST	-0.18	0.88	2.20E-02	7.23E-02
RP5-882C2.2	-0.92	0.53	2.20E-02	7.24E-02
GRAMD2	-0.30	0.81	2.20E-02	7.25E-02
PM20D2	0.26	1.20	2.21E-02	7.25E-02
C14orf132	0.90	1.87	2.21E-02	7.26E-02
HEMK1	-0.21	0.86	2.21E-02	7.27E-02
TNFSF9	-0.28	0.82	2.22E-02	7.28E-02
CWC15	0.22	1.16	2.22E-02	7.30E-02
RGS16	-1.21	0.43	2.22E-02	7.30E-02
IKBKE	-0.32	0.80	2.22E-02	7.30E-02
MSH6	0.19	1.14	2.23E-02	7.31E-02
WDR13	-0.21	0.87	2.23E-02	7.31E-02
ZNF827	-0.27	0.83	2.23E-02	7.31E-02
LYSMD2	0.40	1.32	2.23E-02	7.31E-02
RSPH3	-0.28	0.83	2.23E-02	7.32E-02
TRIOBP	0.18	1.13	2.24E-02	7.34E-02
PAPD4	0.20	1.15	2.24E-02	7.34E-02
CIB2	-0.61	0.66	2.24E-02	7.34E-02
SBSN	-0.19	0.88	2.24E-02	7.34E-02
ZNF618	0.24	1.18	2.24E-02	7.34E-02
GABPB1-AS1	-0.22	0.86	2.24E-02	7.35E-02
RCAN1	-0.21	0.86	2.24E-02	7.35E-02
KRTAP2-3	-1.44	0.37	2.25E-02	NA
FST	-0.43	0.74	2.25E-02	7.36E-02
MOB3C	-0.24	0.85	2.25E-02	7.37E-02
H3F3A	0.21	1.16	2.26E-02	7.38E-02
SURF6	0.20	1.15	2.26E-02	7.39E-02
TNF	0.84	1.80	2.26E-02	7.40E-02
GREM1	1.60	3.02	2.27E-02	NA
ADAMTS6	-0.44	0.74	2.27E-02	7.44E-02
CCBE1	0.28	1.21	2.28E-02	7.44E-02
TBC1D22A	-0.21	0.87	2.28E-02	7.45E-02
EYA3	-0.22	0.86	2.28E-02	7.46E-02
MTMR11	-0.30	0.81	2.29E-02	7.46E-02
GABPA	0.22	1.16	2.29E-02	7.47E-02
GNB1	0.15	1.11	2.29E-02	7.47E-02
PDIA5	0.29	1.22	2.29E-02	7.47E-02
ABCC3	0.26	1.20	2.29E-02	7.48E-02
CSPG4P11	-0.65	0.64	2.31E-02	7.53E-02
GGT1	-0.29	0.82	2.32E-02	7.56E-02
CEP72	0.33	1.25	2.32E-02	7.56E-02
MAP1LC3A	-0.49	0.71	2.32E-02	7.57E-02
LMX1B	1.10	2.14	2.33E-02	NA
OTUD3	0.25	1.19	2.33E-02	7.59E-02
TTBK2	-0.24	0.85	2.34E-02	7.61E-02
TPRXL	-0.32	0.80	2.34E-02	7.61E-02
CTA-384D8.36	-0.54	0.69	2.34E-02	7.62E-02
P3H4	0.26	1.20	2.34E-02	7.62E-02
THAP11	0.26	1.20	2.34E-02	7.62E-02
GAL	0.48	1.39	2.34E-02	7.62E-02
GRK6	-0.21	0.87	2.34E-02	7.62E-02
ATG4C	0.27	1.21	2.35E-02	7.63E-02
SNX8	0.27	1.21	2.35E-02	7.64E-02
PDE6D	-0.29	0.82	2.36E-02	7.67E-02
OXTR	0.62	1.54	2.37E-02	7.69E-02
BRD8	0.19	1.14	2.37E-02	7.69E-02
ATP6V1A	-0.18	0.88	2.37E-02	7.69E-02

CENPQ	0.30	1.23	4.21E-02	1.39E-01
CPEB2	-0.23	0.85	4.21E-02	1.39E-01
LOX	-0.36	0.78	4.21E-02	1.39E-01
HMGN2P5	-0.36	0.78	4.21E-02	1.39E-01
SEMA4B	-0.14	0.91	4.22E-02	1.39E-01
PSMD7	0.16	1.12	4.23E-02	1.39E-01
DPY19L3	-0.29	0.82	4.24E-02	1.39E-01
CTNNA1	0.12	1.08	4.24E-02	1.40E-01
TSHZ1	-0.22	0.86	4.25E-02	1.40E-01
GALT	-0.29	0.82	4.25E-02	1.40E-01
ZNF772	-0.32	0.80	4.25E-02	1.40E-01
STRAP	0.12	1.09	4.25E-02	1.40E-01
EVPL	-0.13	0.91	4.26E-02	1.40E-01
FGD4	-0.21	0.87	4.26E-02	1.40E-01
FICD	-0.37	0.77	4.27E-02	1.40E-01
L1CAM	0.36	1.28	4.27E-02	1.40E-01
MRPL34	-0.22	0.86	4.29E-02	1.41E-01
VARS2	-0.22	0.86	4.29E-02	1.41E-01
TRA2B	0.12	1.09	4.29E-02	1.41E-01
CTB-193M12.5	0.27	1.21	4.29E-02	1.41E-01
INPP5A	0.23	1.17	4.29E-02	1.41E-01
C2CD2	0.22	1.16	4.30E-02	1.41E-01
PIK3R3	-0.32	0.80	4.31E-02	1.41E-01
GALNT10	0.19	1.14	4.31E-02	1.41E-01
C14orf80	-0.26	0.83	4.31E-02	1.41E-01
CYYR1-AS1	-0.25	0.84	4.31E-02	NA
NDUFA6	-0.17	0.89	4.31E-02	1.41E-01
PSMD9	0.25	1.19	4.31E-02	1.41E-01
SMG7-AS1	-0.35	0.78	4.32E-02	1.41E-01
NPIPB5	0.37	1.29	4.32E-02	1.41E-01
ZNF891	-0.37	0.77	4.32E-02	1.41E-01
PWWP2B	-0.23	0.85	4.32E-02	1.41E-01
ARPP19	0.17	1.12	4.33E-02	1.41E-01
FAM155B	-0.37	0.77	4.33E-02	1.42E-01
ADRB2	-0.21	0.87	4.33E-02	1.42E-01
CFL2	0.23	1.17	4.34E-02	1.42E-01
BECN1	0.16	1.12	4.34E-02	1.42E-01
TTC7B	0.23	1.17	4.34E-02	1.42E-01
RSPH3	0.25	1.19	4.34E-02	1.42E-01
SRSF8	-0.19	0.88	4.35E-02	1.42E-01
CPNE7	0.30	1.23	4.36E-02	1.42E-01
MIA3	0.17	1.13	4.36E-02	1.42E-01
CUL4A	0.15	1.11	4.36E-02	1.42E-01
INTS8	0.19	1.14	4.36E-02	1.42E-01
C6orf141	-0.31	0.81	4.37E-02	1.42E-01
RAB38	-0.14	0.91	4.37E-02	1.43E-01
HOXC10	-0.21	0.87	4.38E-02	1.43E-01
MMAB	-0.22	0.86	4.39E-02	1.43E-01
FERMT3	0.31	1.24	4.40E-02	NA
LNP1	-0.36	0.78	4.41E-02	1.44E-01
IHH	-0.24	0.85	4.41E-02	NA
LETMD1	0.22	1.17	4.42E-02	1.44E-01
FAM65A	0.21	1.16	4.43E-02	1.44E-01
ALOX12-AS1	-0.33	0.80	4.43E-02	1.44E-01
ST7	0.24	1.18	4.43E-02	1.44E-01
ARFIP1	0.19	1.14	4.44E-02	1.44E-01
CDK1	-0.18	0.88	4.44E-02	1.44E-01
ZBTB11	0.21	1.16	4.44E-02	1.44E-01
HNRNPR	0.14	1.10	4.44E-02	1.44E-01
TMEM165	0.18	1.13	4.44E-02	1.44E-01
PSENEN	-0.25	0.84	4.44E-02	1.44E-01
PCBD1	-0.19	0.88	4.44E-02	1.44E-01
FAM3C2	0.37	1.30	4.45E-02	1.44E-01
PPIP5K1	0.20	1.15	4.45E-02	1.45E-01
ZDHHC13	0.22	1.16	4.45E-02	1.45E-01
AFAP1L2	0.16	1.12	4.46E-02	1.45E-01
C3orf52	0.32	1.25	4.46E-02	1.45E-01
FANCC	-0.23	0.85	4.46E-02	1.45E-01
XPC	0.20	1.15	4.46E-02	1.45E-01
P2RX5	-0.22	0.86	4.46E-02	NA
RPL30P4	0.34	1.26	4.46E-02	1.45E-01
SMOC1	0.18	1.13	4.46E-02	1.45E-01
SCRN1	0.12	1.09	4.46E-02	1.45E-01
RQCD1	0.14	1.10	4.47E-02	1.45E-01
C11orf31	-0.21	0.86	4.49E-02	1.45E-01
FOXL1	-0.37	0.77	4.50E-02	1.46E-01
FGFR2	0.18	1.14	4.51E-02	1.46E-01
PSMG3	-0.21	0.87	4.51E-02	1.46E-01
MIPOL1	0.28	1.21	4.51E-02	1.46E-01
WDR77	0.18	1.13	4.52E-02	1.46E-01
CES3	-0.36	0.78	4.52E-02	1.46E-01
TMEM106C	-0.16	0.90	4.52E-02	1.46E-01
RP11-147L13.15	-0.31	0.81	4.52E-02	NA
COL7A1	-0.15	0.90	4.52E-02	1.46E-01
ATHL1	-0.37	0.77	4.53E-02	1.46E-01
TAF1	-0.18	0.88	4.53E-02	1.46E-01
NR3C1	0.16	1.12	4.54E-02	1.47E-01
EIF2B5	0.16	1.12	4.54E-02	1.47E-01
ZNF689	-0.31	0.81	4.56E-02	1.47E-01
TMEM14C	-0.16	0.89	4.56E-02	1.47E-01
LRRCC1	0.31	1.24	2.37E-02	7.69E-02
SLC25A4	-0.24	0.84	2.38E-02	7.71E-02
ZNF675	0.28	1.21	2.38E-02	7.72E-02
MYPOP	-0.36	0.78	2.38E-02	7.72E-02
RP11-1094H24.4	-0.82	0.56	2.38E-02	7.73E-02
FBXL16	0.69	1.61	2.38E-02	7.73E-02
CERS5	0.22	1.16	2.39E-02	7.73E-02
YIF1B	0.21	1.15	2.39E-02	7.73E-02
CAND1	0.16	1.12	2.39E-02	7.73E-02
LCE1E	-1.73	0.30	2.39E-02	NA
HECTD4	-0.16	0.89	2.39E-02	7.75E-02
E2F7	0.24	1.18	2.39E-02	7.75E-02
ENO2	0.65	1.56	2.40E-02	7.76E-02
ZNF43	0.33	1.25	2.40E-02	7.76E-02
ESPNL	0.82	1.77	2.40E-02	7.77E-02
CNGB1	1.18	2.26	2.40E-02	NA
ANKS6	0.21	1.16	2.40E-02	7.78E-02
UBTD2	-0.23	0.85	2.41E-02	7.80E-02
KCNS3	0.44	1.36	2.41E-02	7.80E-02
GPR108	-0.18	0.88	2.41E-02	7.80E-02
IL1RAP	0.22	1.16	2.42E-02	7.82E-02
GRB7	0.26	1.20	2.42E-02	7.83E-02
PIK3C2G	-0.40	0.76	2.43E-02	7.84E-02
MTA2	0.15	1.11	2.43E-02	7.84E-02
TNPO3	0.17	1.12	2.43E-02	7.84E-02
UQCRFS1	0.17	1.13	2.43E-02	7.84E-02
CASP14	-0.63	0.65	2.43E-02	7.85E-02
FUT10	0.28	1.22	2.44E-02	7.87E-02
SEPSECS	0.25	1.19	2.44E-02	7.89E-02
MKI67	0.41	1.33	2.45E-02	7.90E-02
EHD2	-0.15	0.90	2.45E-02	7.90E-02
FZD10-AS1	0.68	1.60	2.45E-02	7.90E-02
PFKFB3	0.25	1.19	2.45E-02	7.90E-02
CSRNP2	-0.22	0.86	2.45E-02	7.91E-02
PPOX	-0.33	0.80	2.46E-02	7.92E-02
PDZD8	0.18	1.14	2.46E-02	7.92E-02
HES2	-0.52	0.70	2.46E-02	7.92E-02
DLX3	0.27	1.21	2.46E-02	7.92E-02
HIST2H2BE	-0.36	0.78	2.47E-02	7.95E-02
CES4A	-0.41	0.75	2.48E-02	7.97E-02
RP11-160O5.1	0.87	1.83	2.48E-02	7.97E-02
RTN4RL2	2.96	7.76	2.48E-02	NA
TRIP6	-0.16	0.90	2.49E-02	8.00E-02
WDYHV1	0.33	1.26	2.49E-02	8.01E-02
FAM13A-AS1	-0.59	0.66	2.49E-02	8.01E-02
AGK	-0.23	0.86	2.49E-02	8.01E-02
RP11-632K20.7	-0.71	0.61	2.49E-02	8.01E-02
ZCCHC2	0.20	1.15	2.50E-02	8.02E-02
NFRKB	0.18	1.14	2.50E-02	8.04E-02
MIR3936	-1.22	0.43	2.51E-02	NA
SDHD	0.19	1.14	2.52E-02	8.08E-02
LRRC16B	-0.53	0.69	2.52E-02	8.09E-02
MYADM	-0.24	0.85	2.52E-02	8.09E-02
TADA2A	0.26	1.19	2.52E-02	8.10E-02
TCEB1	0.19	1.14	2.53E-02	8.10E-02
GLIPR2	-0.48	0.72	2.53E-02	8.10E-02
CCT7	0.15	1.11	2.53E-02	8.10E-02
02-sep	0.15	1.11	2.53E-02	8.11E-02
MDFI	0.19	1.14	2.53E-02	8.11E-02
BARX2	0.25	1.19	2.53E-02	8.11E-02
PINLYP	-0.36	0.78	2.53E-02	8.11E-02
CNPY4	-0.40	0.76	2.53E-02	8.11E-02
TMEM63C	0.22	1.17	2.53E-02	8.11E-02
FMO4	-0.50	0.71	2.54E-02	8.12E-02
C14orf159	0.22	1.16	2.54E-02	8.12E-02
CCNK	-0.29	0.82	2.54E-02	8.12E-02
ZC3H11A	-0.15	0.90	2.54E-02	8.13E-02
RNF219	0.29	1.23	2.55E-02	8.15E-02
IDH3G	0.20	1.15	2.56E-02	8.17E-02
LONP1	0.17	1.12	2.56E-02	8.17E-02
NDUFA11	-0.33	0.79	2.56E-02	8.17E-02
TMBIM1	-0.14	0.90	2.56E-02	8.17E-02
ARL9	1.33	2.52	2.56E-02	NA
KIF24	0.27	1.21	2.56E-02	8.19E-02
MPC1	-0.27	0.83	2.57E-02	8.20E-02
TRDMT1	0.44	1.35	2.57E-02	8.20E-02
EIF4E3	0.37	1.29	2.57E-02	8.20E-02
CMPK2	-0.40	0.76	2.57E-02	8.20E-02
TRNT1	0.25	1.19	2.57E-02	8.21E-02
AC005863.1	-0.80	0.57	2.58E-02	8.22E-02
CC2D2A	0.27	1.20	2.58E-02	8.22E-02
TRAPPC13	0.23	1.17	2.58E-02	8.22E-02
REEP4	0.18	1.13	2.58E-02	8.22E-02
YME1L1	0.15	1.11	2.58E-02	8.22E-02
CISD1	0.24	1.18	2.59E-02	8.24E-02
WDR36	0.18	1.13	2.59E-02	8.25E-02
CYP2D7	-0.75	0.59	2.60E-02	8.27E-02
TNFAIP8L1	0.28	1.22	2.60E-02	8.28E-02
HILPDA	-0.28	0.82	2.60E-02	8.28E-02
HMGN1	0.15	1.11	2.60E-02	8.28E-02

DALRD3	-0.24	0.84	4.57E-02	1.47E-01
PURA	0.20	1.15	4.57E-02	1.48E-01
TOMM5	-0.35	0.79	4.57E-02	1.48E-01
FUS	0.12	1.09	4.58E-02	1.48E-01
MXD3	-0.35	0.78	4.58E-02	1.48E-01
CCNG1	0.19	1.14	4.58E-02	1.48E-01
CUL3	0.17	1.13	4.58E-02	1.48E-01
RP11-204K16.1	0.24	1.18	4.58E-02	NA
TXNDC15	-0.23	0.85	4.58E-02	1.48E-01
HSPA1B	0.20	1.15	4.59E-02	1.48E-01
ABHD15	-0.30	0.81	4.59E-02	1.48E-01
TMEM170A	0.24	1.18	4.59E-02	1.48E-01
CEP350	-0.18	0.89	4.59E-02	1.48E-01
C16orf62	-0.19	0.88	4.60E-02	1.48E-01
RP11-756P10.3	0.35	1.27	4.60E-02	1.48E-01
C1orf21	-0.18	0.88	4.60E-02	1.48E-01
RBMS1P1	-0.22	0.86	4.61E-02	NA
CCDC117	0.22	1.16	4.61E-02	1.48E-01
INO80D	-0.24	0.84	4.62E-02	1.48E-01
ARL4A	-0.30	0.81	4.62E-02	1.49E-01
TRAPPC2L	-0.20	0.87	4.63E-02	1.49E-01
PER1	-0.18	0.88	4.63E-02	1.49E-01
EIF4A3	-0.14	0.91	4.63E-02	1.49E-01
XPOTP1	-0.23	0.85	4.64E-02	NA
SCNN1A	-0.19	0.88	4.64E-02	1.49E-01
UBA3	0.18	1.13	4.65E-02	1.49E-01
ZNF114	-0.34	0.79	4.66E-02	1.50E-01
GTF2H1	0.17	1.13	4.66E-02	1.50E-01
LRP11	0.15	1.11	4.67E-02	1.50E-01
CDK2	-0.17	0.89	4.67E-02	1.50E-01
SENP8	-0.36	0.78	4.67E-02	1.50E-01
RP11-275I14.4	-0.17	0.89	4.68E-02	NA
NIPA2	0.17	1.13	4.68E-02	1.50E-01
KANK3	0.29	1.22	4.68E-02	NA
RPL27	-0.11	0.92	4.69E-02	1.50E-01
MTMR14	0.18	1.13	4.69E-02	1.50E-01
CSTA	-0.16	0.90	4.69E-02	1.50E-01
AC005083.1	0.37	1.29	4.70E-02	1.50E-01
UTP6	0.16	1.12	4.70E-02	1.51E-01
OSBPL5	-0.24	0.85	4.70E-02	1.51E-01
FASTKD1	0.20	1.15	4.71E-02	1.51E-01
ZNF812	-0.35	0.78	4.71E-02	1.51E-01
ZC3H13	0.14	1.10	4.71E-02	1.51E-01
KIF23	0.15	1.11	4.71E-02	1.51E-01
HDAC11	-0.22	0.86	4.71E-02	1.51E-01
EPHA1	-0.16	0.90	4.72E-02	1.51E-01
BSCL2	-0.35	0.78	4.72E-02	1.51E-01
GATC	0.22	1.17	4.72E-02	1.51E-01
COQ3	-0.26	0.84	4.72E-02	1.51E-01
PCOLCE2	-0.28	0.82	4.72E-02	NA
DBP	-0.37	0.78	4.72E-02	1.51E-01
WASH7P	0.30	1.23	4.73E-02	1.51E-01
ZMYND8	-0.15	0.90	4.73E-02	1.51E-01
RP11-585P4.5	-0.36	0.78	4.73E-02	1.51E-01
DHRS13	-0.37	0.77	4.73E-02	1.51E-01
PHKA2	0.20	1.15	4.73E-02	1.51E-01
YLPM1	0.14	1.10	4.74E-02	1.51E-01
EOMES	-0.33	0.80	4.76E-02	1.52E-01
ARHGEF17	-0.22	0.86	4.77E-02	1.52E-01
PLPP6	-0.31	0.81	4.78E-02	1.52E-01
CRISPLD2	-0.24	0.84	4.78E-02	1.52E-01
BAZ1B	0.14	1.10	4.79E-02	1.53E-01
TET2	0.18	1.14	4.80E-02	1.53E-01
PPP1R3E	-0.37	0.77	4.81E-02	1.53E-01
ALDH6A1	-0.29	0.82	4.81E-02	1.53E-01
ZDHHC3	0.13	1.10	4.81E-02	1.53E-01
SORBS3	-0.19	0.88	4.81E-02	1.53E-01
POLR3G	0.22	1.17	4.81E-02	1.53E-01
HOXB4	-0.22	0.86	4.81E-02	NA
C14orf159	-0.22	0.86	4.81E-02	1.53E-01
SOWAHD	-0.20	0.87	4.81E-02	NA
SLC19A2	0.23	1.18	4.81E-02	1.53E-01
TAF4B	0.26	1.20	4.82E-02	1.53E-01
EIF5A2	0.29	1.23	4.82E-02	1.53E-01
CTA-384D8.35	-0.17	0.89	4.82E-02	NA
FOXN3	-0.24	0.85	4.83E-02	1.53E-01
MRVI1	-0.36	0.78	4.83E-02	1.53E-01
TNPO3	0.13	1.10	4.83E-02	1.53E-01
GUSBP1	0.36	1.29	4.83E-02	1.53E-01
NDE1	0.15	1.11	4.83E-02	1.54E-01
GAREM	0.28	1.21	4.84E-02	1.54E-01
ANKRD37	-0.36	0.78	4.84E-02	1.54E-01
KRT16P3	-0.26	0.84	4.85E-02	NA
TSEN15	-0.19	0.88	4.85E-02	1.54E-01
NCOA2	-0.19	0.87	4.85E-02	1.54E-01
TNNI3	-0.34	0.79	4.85E-02	1.54E-01
TLDC2	-0.19	0.87	4.86E-02	NA
CASP9	0.30	1.23	4.86E-02	1.54E-01
TUBE1	0.26	1.20	4.86E-02	1.54E-01
ZNF585B	-0.29	0.82	4.87E-02	1.54E-01
MYO1C	-0.15	0.90	2.61E-02	8.29E-02
ADI1	0.17	1.13	2.61E-02	8.30E-02
TAZ	-0.23	0.86	2.61E-02	8.30E-02
EIF1	0.15	1.11	2.61E-02	8.30E-02
GALNT3	0.17	1.13	2.61E-02	8.31E-02
MRPL35	0.19	1.14	2.61E-02	8.31E-02
ZFP57	-0.37	0.78	2.62E-02	8.31E-02
ANKH	-0.21	0.86	2.62E-02	8.31E-02
ABHD10	0.23	1.17	2.62E-02	8.31E-02
RPTN	-0.48	0.72	2.62E-02	8.32E-02
GCFC2	0.29	1.22	2.63E-02	8.36E-02
ZNF18	-0.29	0.82	2.63E-02	8.36E-02
IER5L	0.37	1.29	2.63E-02	8.36E-02
ADGRA3	0.18	1.13	2.64E-02	8.37E-02
UTP11L	0.20	1.15	2.64E-02	8.38E-02
E2F2	0.30	1.23	2.64E-02	8.39E-02
ZMYM1	0.21	1.15	2.65E-02	8.39E-02
MTRF1	-0.30	0.81	2.65E-02	8.39E-02
GTPBP10	0.25	1.19	2.65E-02	8.39E-02
SLC25A40	0.25	1.19	2.65E-02	8.39E-02
EPB41L4B	0.23	1.17	2.65E-02	8.39E-02
RPRD1A	0.17	1.12	2.65E-02	8.40E-02
TMEM33	0.17	1.13	2.66E-02	8.42E-02
MBD1	-0.16	0.89	2.66E-02	8.42E-02
TMEM51-AS1	-0.43	0.74	2.67E-02	8.43E-02
MAPK7	-0.23	0.85	2.67E-02	8.45E-02
ACBD4	-0.32	0.80	2.67E-02	8.45E-02
ATXN2L	-0.16	0.89	2.68E-02	8.46E-02
UBE2Q2P1	-0.62	0.65	2.68E-02	8.46E-02
PRMT6	0.22	1.16	2.68E-02	8.46E-02
AIFM2	0.38	1.30	2.68E-02	8.47E-02
CACFD1	-0.35	0.78	2.68E-02	8.47E-02
HNRNPDL	0.16	1.12	2.68E-02	8.47E-02
TECPR2	0.20	1.15	2.68E-02	8.47E-02
VPS33B	0.22	1.16	2.69E-02	8.47E-02
HNRNPF	0.18	1.14	2.69E-02	8.47E-02
SEMA5A	-0.36	0.78	2.69E-02	8.47E-02
CYC1	0.16	1.12	2.69E-02	8.47E-02
ABTB2	0.27	1.21	2.69E-02	8.47E-02
FH	-0.16	0.89	2.69E-02	8.47E-02
TMEM101	-0.23	0.85	2.69E-02	8.48E-02
TRIB1	-0.23	0.85	2.69E-02	8.48E-02
ZNF654	-0.26	0.84	2.69E-02	8.48E-02
FGF2	0.91	1.88	2.69E-02	8.48E-02
SRP54	0.19	1.14	2.69E-02	8.49E-02
RP11-418J17.1	-0.38	0.77	2.70E-02	8.49E-02
ACOT2	-0.58	0.67	2.70E-02	8.49E-02
DYRK4	-0.29	0.82	2.70E-02	8.49E-02
INPP5K	-0.23	0.85	2.70E-02	8.49E-02
FGD6	0.19	1.14	2.70E-02	8.51E-02
ADCK3	-0.35	0.79	2.71E-02	8.52E-02
ASCC2	-0.16	0.89	2.71E-02	8.52E-02
CAPS	-0.52	0.70	2.71E-02	8.52E-02
CCDC47	0.17	1.12	2.71E-02	8.52E-02
TBC1D1	0.23	1.17	2.72E-02	8.55E-02
LAMP2	0.15	1.11	2.72E-02	8.55E-02
PIGP	-0.32	0.80	2.72E-02	8.56E-02
MAPRE3	-0.22	0.86	2.73E-02	8.56E-02
RP11-706O15.1	-0.31	0.81	2.73E-02	8.56E-02
CHEK2	0.26	1.20	2.73E-02	8.56E-02
TPPP3	1.14	2.20	2.73E-02	NA
ANG	-1.50	0.35	2.73E-02	NA
RP3-368A4.5	-0.45	0.73	2.73E-02	8.58E-02
CCDC159	-0.43	0.74	2.74E-02	8.59E-02
PCDHGA9	-1.34	0.40	2.74E-02	NA
PDK3	0.45	1.36	2.74E-02	8.60E-02
EBAG9	0.26	1.20	2.74E-02	8.61E-02
HPS1	-0.18	0.88	2.75E-02	8.63E-02
AGMAT	0.62	1.53	2.76E-02	8.65E-02
ZNF530	0.41	1.33	2.76E-02	8.65E-02
CLDND1	-0.18	0.88	2.76E-02	8.65E-02
AURKB	0.54	1.46	2.76E-02	8.66E-02
GALNT11	-0.20	0.87	2.77E-02	8.66E-02
SMU1	0.17	1.13	2.77E-02	8.66E-02
GABRQ	0.66	1.58	2.77E-02	8.67E-02
SV2A	0.59	1.50	2.78E-02	8.69E-02
POLR3F	0.26	1.20	2.78E-02	8.69E-02
TMEM139	-0.54	0.69	2.78E-02	8.71E-02
MKRN1	0.17	1.13	2.79E-02	8.71E-02
PHF13	0.21	1.16	2.79E-02	8.72E-02
NDUFB10	0.17	1.13	2.80E-02	8.74E-02
TBC1D10B	-0.17	0.89	2.80E-02	8.74E-02
BAIAP2-AS1	-0.33	0.79	2.80E-02	8.75E-02
SNHG9	-0.62	0.65	2.80E-02	8.75E-02
TNNT1	0.21	1.15	2.81E-02	8.77E-02
ORC5	0.22	1.16	2.81E-02	8.77E-02
MIR647	-1.20	0.44	2.81E-02	NA
DOPEY2	-0.21	0.86	2.82E-02	8.80E-02
COMMD10	0.26	1.20	2.82E-02	8.81E-02
SNX16	0.31	1.24	2.82E-02	8.81E-02

**NAV2**	-0.15	0.90	4.87E-02	1.54E-01
**MED16**	-0.19	0.88	4.88E-02	1.55E-01
**RORA**	-0.33	0.79	4.88E-02	1.55E-01
**TKT**	-0.11	0.93	4.90E-02	1.55E-01
**VPS54**	0.20	1.15	4.90E-02	1.55E-01
**AGER**	0.34	1.26	4.90E-02	1.55E-01
**DAAM1**	0.15	1.11	4.90E-02	1.55E-01
**CCNH**	0.20	1.15	4.90E-02	1.55E-01
**SLC25A38**	0.21	1.15	4.90E-02	1.55E-01
**42993**	0.16	1.12	4.90E-02	1.55E-01
**OTUB1**	0.15	1.11	4.91E-02	1.55E-01
**BST2**	-0.35	0.78	4.91E-02	1.55E-01
**MFSD9**	0.24	1.18	4.91E-02	1.55E-01
**USP9Y**	0.19	1.14	4.91E-02	1.55E-01
**USP25**	0.18	1.13	4.92E-02	1.55E-01
**RP11-767C1.2**	-0.23	0.85	4.92E-02	NA
**TUBA1A**	0.25	1.19	4.92E-02	1.55E-01
**GATAD2B**	-0.20	0.87	4.92E-02	1.55E-01
**NRBP1**	0.13	1.09	4.92E-02	1.55E-01
**DHODH**	-0.27	0.83	4.93E-02	1.56E-01
**PLXNA1**	0.15	1.11	4.94E-02	1.56E-01
**RAB30**	-0.33	0.80	4.94E-02	1.56E-01
**RAB10**	-0.12	0.92	4.94E-02	1.56E-01
**GPR153**	-0.19	0.88	4.94E-02	1.56E-01
**PITPNM2**	0.19	1.14	4.95E-02	1.56E-01
**CIC**	-0.16	0.89	4.95E-02	1.56E-01
**DEPDC1B**	0.18	1.13	4.95E-02	1.56E-01
**RP11-566E18.1**	-0.37	0.78	4.95E-02	1.56E-01
**TEX261**	0.16	1.12	4.96E-02	1.56E-01
**RP11-798M19.6**	-0.31	0.81	4.97E-02	NA
**PHKG2**	0.25	1.19	4.97E-02	1.56E-01
**RP11-834C11.4**	-0.32	0.80	4.97E-02	1.56E-01
**ANKRD22**	-0.23	0.85	4.97E-02	1.56E-01
**PRKAR2A**	0.13	1.09	4.97E-02	1.56E-01
**POSTN**	-0.22	0.86	4.97E-02	NA
**SNTA1**	-0.32	0.80	4.98E-02	1.57E-01
**CYCS**	0.16	1.11	4.99E-02	1.57E-01
**ATP6V0E2-AS1**	-0.27	0.83	4.99E-02	NA
**BCHE**	-0.35	0.78	4.99E-02	1.57E-01
**SERINC2**	0.15	1.11	5.00E-02	1.57E-01
**ALS2CL**	-0.16	0.90	5.00E-02	1.57E-01

**ORAI2**	0.37	1.29	2.84E-02	8.85E-02
**ACSF2**	-0.17	0.89	2.84E-02	8.85E-02
**SCAMP2**	-0.16	0.90	2.84E-02	8.87E-02
**ZNF471**	-0.39	0.77	2.85E-02	8.87E-02
**AL357673.1**	0.55	1.47	2.85E-02	8.88E-02
**MUC13**	-2.79	0.14	2.85E-02	NA
**ADCK2**	-0.26	0.84	2.85E-02	8.89E-02
**SLC39A8**	-0.27	0.83	2.85E-02	8.89E-02
**ABCB10**	0.21	1.16	2.86E-02	8.90E-02
**PIK3CD**	-0.23	0.86	2.86E-02	8.91E-02
**THOC1**	0.23	1.17	2.87E-02	8.92E-02
**WDR81**	-0.20	0.87	2.87E-02	8.94E-02
**STON1**	0.48	1.40	2.87E-02	8.94E-02
**GARS**	0.16	1.11	2.88E-02	8.95E-02
**PROM2**	-0.16	0.89	2.88E-02	8.95E-02
**NEDD4L**	0.20	1.15	2.88E-02	8.95E-02
**PARP6**	-0.21	0.86	2.88E-02	8.95E-02
**EXD3**	-0.34	0.79	2.88E-02	8.95E-02
**CALM1**	0.14	1.10	2.89E-02	8.97E-02
**KLHDC8B**	0.21	1.16	2.89E-02	8.99E-02
**TMEM37**	0.87	1.83	2.90E-02	8.99E-02
**ICE1**	0.17	1.13	2.90E-02	9.00E-02
**FBXL20**	-0.28	0.83	2.90E-02	9.00E-02
**KCNS2**	-1.39	0.38	2.90E-02	NA
**BDP1**	0.17	1.12	2.91E-02	9.04E-02
**RCL1**	-0.23	0.85	2.91E-02	9.04E-02
**ZNHIT6**	0.21	1.16	2.91E-02	9.04E-02
**DHX15**	0.15	1.11	2.91E-02	9.04E-02
**PCM1**	0.16	1.12	2.92E-02	9.04E-02
**GLO1**	0.15	1.11	2.92E-02	9.05E-02
**GTF2IP12**	-0.48	0.72	2.92E-02	9.05E-02
**WDR45**	-0.22	0.86	2.92E-02	9.05E-02
**EIF4B**	0.15	1.11	2.93E-02	9.06E-02
**TCEAL1**	-0.36	0.78	2.93E-02	9.06E-02
**PSMD6**	0.17	1.12	2.93E-02	9.06E-02
**DCAF13**	0.19	1.14	2.93E-02	9.06E-02
**BMPR1A**	0.21	1.15	2.93E-02	9.07E-02
**DLK2**	-0.25	0.84	2.93E-02	9.08E-02
**TAF1B**	0.26	1.20	2.94E-02	9.08E-02
**U2AF2**	0.15	1.11	2.94E-02	9.09E-02
**R3HDM1**	0.18	1.13	2.94E-02	9.09E-02
**PADI1**	0.42	1.34	2.95E-02	9.11E-02
**SLCO4A1**	0.44	1.36	2.95E-02	9.11E-02
**MAN2A2**	-0.21	0.87	2.95E-02	9.11E-02
**PPFIA1**	0.18	1.13	2.95E-02	9.13E-02
**IL21R**	3.15	8.87	2.95E-02	NA
**PANX1**	0.21	1.16	2.96E-02	9.13E-02
**RBPMS**	-0.23	0.85	2.96E-02	9.13E-02
**DSG4**	-1.17	0.45	2.97E-02	NA
**OASL**	-0.40	0.76	2.98E-02	9.20E-02
**GJB5**	-0.17	0.89	2.98E-02	9.21E-02
**MRPL42**	0.19	1.14	2.99E-02	9.22E-02
**SDHA**	0.16	1.11	2.99E-02	9.23E-02
**PNO1**	0.22	1.16	3.00E-02	9.24E-02
**SSBP1**	0.17	1.12	3.00E-02	9.25E-02
**TXLNA**	-0.17	0.89	3.01E-02	9.27E-02
**SNRPF**	0.19	1.14	3.01E-02	9.27E-02
**RAD1**	0.22	1.17	3.01E-02	9.27E-02
**S100A14**	-0.14	0.91	3.01E-02	9.27E-02
**ZNF532**	-0.18	0.88	3.01E-02	9.28E-02
**FOXM1**	-0.62	0.65	3.02E-02	9.29E-02
**ATF5**	0.27	1.20	3.02E-02	9.29E-02
**GABPB1**	0.23	1.18	3.02E-02	9.29E-02
**ERV3-1**	-0.31	0.80	3.02E-02	9.29E-02
**C19orf24**	-0.24	0.84	3.02E-02	9.30E-02
**URB2**	0.21	1.16	3.02E-02	9.30E-02
**AKAP10**	0.21	1.16	3.03E-02	9.32E-02
**CEBPA-AS1**	0.61	1.52	3.03E-02	9.33E-02
**ABCA10**	-0.93	0.52	3.05E-02	9.36E-02
**INTS4P2**	-3.13	0.11	3.05E-02	NA
**TOM1**	0.22	1.16	3.05E-02	9.36E-02
**RP11-347C18.3**	-0.98	0.51	3.05E-02	NA
**N4BP2L2**	-0.18	0.88	3.06E-02	9.39E-02
**SRSF10**	0.21	1.16	3.06E-02	9.40E-02
**TARBP2**	-0.23	0.85	3.06E-02	9.40E-02
**PUS10**	0.36	1.28	3.07E-02	9.41E-02
**DDAH2**	-0.30	0.81	3.07E-02	9.42E-02
**ARFRP1**	-0.21	0.87	3.07E-02	9.43E-02
**RIOK2**	0.23	1.17	3.07E-02	9.43E-02
**FKTN**	0.18	1.14	3.08E-02	9.43E-02
**NDUFB9**	0.19	1.14	3.09E-02	9.47E-02
**RASSF6**	-0.26	0.84	3.09E-02	9.47E-02
**NUP50**	-0.16	0.90	3.09E-02	9.48E-02
**CCND3**	0.18	1.13	3.10E-02	9.49E-02
**KRT19**	0.16	1.12	3.10E-02	9.50E-02
**SYNRG**	0.20	1.15	3.10E-02	9.50E-02
**INTS6**	0.17	1.13	3.10E-02	9.50E-02
**ATF3**	-0.60	0.66	3.10E-02	9.50E-02
**CYP2E1**	-0.71	0.61	3.11E-02	9.51E-02
**PRMT2**	-0.18	0.88	3.11E-02	9.52E-02

**CBLC**	-0.18	0.88	3.11E-02	9.52E-02
**AMT**	-0.38	0.77	3.12E-02	9.54E-02
**WNT9A**	0.28	1.22	3.12E-02	9.54E-02
**NKAPL**	-0.72	0.61	3.12E-02	9.54E-02
**ARHGAP26**	-0.30	0.81	3.12E-02	9.54E-02
**XDH**	-0.27	0.83	3.13E-02	9.56E-02
**FGFR2**	0.16	1.12	3.13E-02	9.57E-02
**CST3**	-0.17	0.89	3.13E-02	9.57E-02
**PTAR1**	-0.18	0.88	3.13E-02	9.57E-02
**PVR**	-0.16	0.89	3.14E-02	9.57E-02
**ACTR1A**	-0.15	0.90	3.14E-02	9.58E-02
**GRAMD4**	0.18	1.14	3.14E-02	9.58E-02
**EIF3A**	0.14	1.10	3.14E-02	9.59E-02
**RAB11FIP5**	-0.17	0.89	3.15E-02	9.59E-02
**EIF4H**	0.14	1.10	3.15E-02	9.59E-02
**XPNPEP1**	0.18	1.13	3.15E-02	9.60E-02
**ERP29**	0.16	1.12	3.15E-02	9.61E-02
**HNRNPUL2**	0.18	1.13	3.15E-02	9.61E-02
**SSFA2**	0.15	1.11	3.15E-02	9.61E-02
**IFNGR1**	-0.19	0.88	3.16E-02	9.61E-02
**MTMR14**	-0.22	0.86	3.16E-02	9.62E-02
**RP5-890E16.5**	1.23	2.34	3.16E-02	NA
**PSORS1C1**	-0.44	0.74	3.17E-02	9.64E-02
**NHS**	0.22	1.16	3.17E-02	9.65E-02
**CECR1**	-0.44	0.74	3.18E-02	9.67E-02
**SNX27**	0.20	1.15	3.18E-02	9.68E-02
**GSPT2**	0.39	1.31	3.19E-02	9.69E-02
**NFATC2IP**	0.16	1.12	3.19E-02	9.69E-02
**LYPLA1**	0.16	1.12	3.19E-02	9.69E-02
**KIAA1211L**	0.33	1.26	3.19E-02	9.70E-02
**SOX2**	-0.51	0.70	3.19E-02	9.71E-02
**ABHD14A**	-0.61	0.65	3.20E-02	9.71E-02
**UGDH**	-0.23	0.85	3.20E-02	9.71E-02
**GALM**	0.49	1.40	3.20E-02	9.71E-02
**SNCA**	-0.32	0.80	3.20E-02	9.71E-02
**RP11-443P15.2**	-0.40	0.76	3.20E-02	9.72E-02
**CTD-2366F13.1**	-0.50	0.71	3.21E-02	9.73E-02
**FANCM**	0.24	1.18	3.21E-02	9.75E-02
**PICK1**	-0.20	0.87	3.21E-02	9.75E-02
**PKM**	0.14	1.10	3.22E-02	9.75E-02
**FAM110A**	0.22	1.17	3.22E-02	9.75E-02
**TRPM6**	-1.14	0.45	3.22E-02	NA
**RNASEH2C**	0.25	1.19	3.22E-02	9.76E-02
**GTF2F2**	0.17	1.13	3.22E-02	9.76E-02
**LSM6**	0.30	1.23	3.22E-02	9.76E-02
**PRELID3B**	0.17	1.13	3.22E-02	9.77E-02
**H2AFY2**	0.19	1.14	3.22E-02	9.77E-02
**YTHDF2**	0.16	1.12	3.23E-02	9.77E-02
**PPIH**	0.23	1.17	3.23E-02	9.78E-02
**SAFB**	0.16	1.12	3.23E-02	9.79E-02
**LRRC29**	-0.79	0.58	3.23E-02	9.79E-02
**CEACAM6**	-0.22	0.86	3.24E-02	9.79E-02
**RP11-283G6.3**	1.21	2.31	3.24E-02	NA
**ARID5A**	0.43	1.34	3.24E-02	9.81E-02
**UBAC1**	0.17	1.13	3.24E-02	9.81E-02
**B3GALT6**	-0.30	0.81	3.24E-02	9.81E-02
**WBSCR16**	0.27	1.20	3.25E-02	9.81E-02
**ST6GALNAC4**	0.22	1.17	3.25E-02	9.81E-02
**MEST**	0.31	1.24	3.25E-02	9.81E-02
**RNF149**	0.19	1.14	3.25E-02	9.81E-02
**EAF1**	0.21	1.16	3.25E-02	9.81E-02
**FARSB**	0.16	1.12	3.25E-02	9.81E-02
**TENM2**	-0.14	0.90	3.25E-02	9.81E-02
**CRY2**	0.28	1.21	3.25E-02	9.81E-02
**USP10**	0.16	1.11	3.25E-02	9.81E-02
**SPG11**	-0.16	0.90	3.25E-02	9.81E-02
**HCN3**	-0.32	0.80	3.25E-02	9.81E-02
**TMEM80**	-0.27	0.83	3.25E-02	9.81E-02
**SLC10A6**	-0.38	0.77	3.26E-02	9.82E-02
**PIGL**	-0.29	0.82	3.26E-02	9.82E-02
**TLR1**	-0.79	0.58	3.27E-02	9.84E-02
**TMCO6**	-0.32	0.80	3.27E-02	9.87E-02
**ATP5F1**	0.15	1.11	3.28E-02	9.87E-02
**FIP1L1**	0.26	1.20	3.28E-02	9.87E-02
**MAP4K3**	0.20	1.15	3.28E-02	9.87E-02
**SAMD12**	-0.28	0.82	3.28E-02	9.87E-02
**CLCA2**	-0.15	0.90	3.28E-02	9.87E-02
**PRMT1**	0.17	1.12	3.28E-02	9.88E-02
**NUP85**	0.18	1.13	3.29E-02	9.89E-02
**R3HDM4**	-0.18	0.88	3.29E-02	9.91E-02
**ATP5G2**	0.15	1.11	3.29E-02	9.91E-02
**UHRF2**	0.19	1.14	3.30E-02	9.91E-02
**WDR44**	-0.20	0.87	3.30E-02	9.93E-02
**TCF12**	0.16	1.12	3.31E-02	9.95E-02
**CPSF7**	0.16	1.12	3.31E-02	9.95E-02
**RAB10**	0.15	1.11	3.31E-02	9.95E-02
**TMEM200B**	0.50	1.41	3.31E-02	9.95E-02
**DHPS**	-0.22	0.86	3.32E-02	9.95E-02
**ZNF711**	0.37	1.29	3.32E-02	9.95E-02
**EIF4G1**	0.13	1.10	3.32E-02	9.95E-02

**PEX10**	0.24	1.18	3.32E-02	9.97E-02
**PTPN23**	-0.17	0.89	3.33E-02	1.00E-01
**GJC1**	0.22	1.16	3.33E-02	1.00E-01
**RBM12B**	0.23	1.17	3.34E-02	1.00E-01
**METTL2B**	0.21	1.16	3.34E-02	1.00E-01
**FAM111B**	0.24	1.18	3.35E-02	1.00E-01
**SERPINB13**	-0.46	0.73	3.35E-02	1.00E-01
**POU2F1**	0.25	1.19	3.36E-02	1.01E-01
**CHMP7**	0.19	1.14	3.36E-02	1.01E-01
**CRIPAK**	-0.29	0.82	3.36E-02	1.01E-01
**VARS2**	-0.18	0.88	3.36E-02	1.01E-01
**WASH1**	-0.29	0.82	3.36E-02	1.01E-01
**FHL2**	-0.18	0.88	3.36E-02	1.01E-01
**ZBTB47**	-0.25	0.84	3.37E-02	1.01E-01
**ALDH1B1**	0.25	1.19	3.37E-02	1.01E-01
**MCOLN1**	-0.27	0.83	3.37E-02	1.01E-01
**RGS3**	-0.20	0.87	3.37E-02	1.01E-01
**ITGA4**	0.44	1.36	3.37E-02	1.01E-01
**CISD3**	0.23	1.17	3.38E-02	1.01E-01
**COPS2**	0.16	1.12	3.38E-02	1.01E-01
**CDK18**	-0.23	0.85	3.38E-02	1.01E-01
**SEC24D**	-0.19	0.88	3.39E-02	1.01E-01
**ANKRD36**	-0.36	0.78	3.39E-02	1.01E-01
**FAM171A1**	0.25	1.19	3.39E-02	1.01E-01
**FN1**	-0.46	0.73	3.40E-02	1.01E-01
**LAPTM4B**	0.14	1.10	3.40E-02	1.02E-01
**B3GNT2**	-0.19	0.88	3.41E-02	1.02E-01
**ARF3**	0.14	1.10	3.41E-02	1.02E-01
**THRAP3**	0.15	1.11	3.41E-02	1.02E-01
**TIFA**	0.34	1.26	3.42E-02	1.02E-01
**TTTY16**	-1.29	0.41	3.42E-02	NA
**RALBP1**	0.15	1.11	3.43E-02	1.02E-01
**RNF208**	-0.58	0.67	3.44E-02	1.02E-01
**GYG2P1**	-0.48	0.72	3.44E-02	1.02E-01
**CDKN1C**	0.44	1.36	3.44E-02	1.02E-01
**RASSF9**	-0.25	0.84	3.44E-02	1.02E-01
**SLK**	0.14	1.10	3.44E-02	1.02E-01
**DLEU2**	0.48	1.40	3.44E-02	1.02E-01
**GLI2**	1.60	3.03	3.45E-02	NA
**SS18**	0.16	1.11	3.45E-02	1.03E-01
**DAXX**	-0.17	0.89	3.45E-02	1.03E-01
**CASZ1**	-0.18	0.88	3.46E-02	1.03E-01
**C11orf45**	-0.32	0.80	3.46E-02	1.03E-01
**SAMD9**	-0.16	0.89	3.46E-02	1.03E-01
**PODXL2**	-0.40	0.76	3.47E-02	1.03E-01
**RNASEH2B**	0.25	1.19	3.47E-02	1.03E-01
**SNX33**	-0.17	0.89	3.47E-02	1.03E-01
**RP1-228H13.5**	-0.71	0.61	3.47E-02	1.03E-01
**MTPAP**	0.21	1.16	3.47E-02	1.03E-01
**TKT**	-0.14	0.91	3.48E-02	1.03E-01
**HMGN2P5**	0.45	1.37	3.48E-02	1.03E-01
**FAM81A**	0.95	1.93	3.48E-02	1.03E-01
**GADD45B**	-0.18	0.88	3.48E-02	1.03E-01
**RP11-206L10.2**	-0.39	0.76	3.49E-02	1.04E-01
**PVT1**	-0.33	0.80	3.49E-02	1.04E-01
**NBPF10**	-0.21	0.87	3.49E-02	1.04E-01
**SGK3**	0.46	1.38	3.49E-02	1.04E-01
**AGPAT3**	-0.15	0.90	3.50E-02	1.04E-01
**FAM84B**	-0.17	0.89	3.50E-02	1.04E-01
**C6orf141**	-0.21	0.86	3.50E-02	1.04E-01
**RP11-334C17.5**	-0.64	0.64	3.51E-02	1.04E-01
**MAGI2-AS3**	0.33	1.26	3.51E-02	1.04E-01
**GPAA1**	-0.15	0.90	3.51E-02	1.04E-01
**AP003068.23**	-0.56	0.68	3.52E-02	1.04E-01
**RP11-21B23.2**	-0.73	0.60	3.52E-02	1.04E-01
**USP32**	0.17	1.13	3.52E-02	1.04E-01
**SPATS2**	-0.18	0.88	3.52E-02	1.04E-01
**MAP7**	0.16	1.12	3.52E-02	1.04E-01
**HNRNPH2**	0.16	1.12	3.52E-02	1.04E-01
**GLOD4**	0.17	1.13	3.53E-02	1.04E-01
**PARD6B**	0.46	1.38	3.53E-02	1.04E-01
**ZNF200**	0.30	1.23	3.53E-02	1.04E-01
**CCDC28B**	0.38	1.30	3.53E-02	1.04E-01
**FAM53B**	-0.18	0.88	3.54E-02	1.04E-01
**PKP4**	0.17	1.13	3.54E-02	1.04E-01
**TNK2-AS1**	-0.97	0.51	3.54E-02	1.04E-01
**ASXL2**	-0.16	0.89	3.54E-02	1.05E-01
**MRPL44**	0.20	1.15	3.54E-02	1.05E-01
**CAMK4**	0.78	1.72	3.55E-02	1.05E-01
**NRARP**	0.47	1.38	3.55E-02	1.05E-01
**UHRF1BP1**	0.18	1.13	3.56E-02	1.05E-01
**FRMD4A**	-0.24	0.85	3.56E-02	1.05E-01
**CD14**	0.47	1.39	3.56E-02	1.05E-01
**YWHAZ**	0.14	1.10	3.56E-02	1.05E-01
**TBC1D10A**	-0.25	0.84	3.57E-02	1.05E-01
**SEH1L**	0.19	1.14	3.57E-02	1.05E-01
**KAT7**	0.19	1.14	3.58E-02	1.06E-01
**NT5DC3**	-0.27	0.83	3.58E-02	1.06E-01
**UBE2E1**	0.16	1.12	3.58E-02	1.06E-01
**MYEF2**	1.20	2.29	3.58E-02	NA

**AJUBA**	-0.15	0.90	3.59E-02	1.06E-01
**STRIP2**	0.22	1.16	3.59E-02	1.06E-01
**GMPPB**	0.21	1.15	3.60E-02	1.06E-01
**BMP7**	0.70	1.62	3.60E-02	1.06E-01
**MAT2A**	0.15	1.11	3.60E-02	1.06E-01
**C2orf44**	-0.27	0.83	3.61E-02	1.06E-01
**PPP2R5C**	0.15	1.11	3.61E-02	1.06E-01
**PPP3R1**	0.20	1.15	3.61E-02	1.06E-01
**PSMA3-AS1**	-0.26	0.84	3.62E-02	1.06E-01
**YRDC**	0.23	1.17	3.62E-02	1.06E-01
**KXD1**	-0.21	0.86	3.62E-02	1.06E-01
**FAM83F**	0.16	1.11	3.62E-02	1.06E-01
**UCHL5**	0.17	1.13	3.64E-02	1.07E-01
**LIG4**	-0.20	0.87	3.64E-02	1.07E-01
**PUS1**	0.19	1.14	3.64E-02	1.07E-01
**GLRB**	-0.70	0.62	3.65E-02	1.07E-01
**SDHB**	0.16	1.12	3.65E-02	1.07E-01
**KLHL36**	-0.16	0.89	3.65E-02	1.07E-01
**ECSIT**	0.20	1.15	3.66E-02	1.07E-01
**BCAT2**	-0.20	0.87	3.66E-02	1.07E-01
**FGD3**	-0.56	0.68	3.66E-02	1.07E-01
**PLAC1**	-0.51	0.70	3.67E-02	1.07E-01
**ZNF841**	-0.29	0.82	3.67E-02	1.07E-01
**CARS2**	0.16	1.12	3.67E-02	1.08E-01
**PDE5A**	-0.34	0.79	3.67E-02	1.08E-01
**ITPRIP**	-0.16	0.90	3.68E-02	1.08E-01
**DEF8**	-0.16	0.89	3.68E-02	1.08E-01
**UQCRC1**	0.14	1.10	3.69E-02	1.08E-01
**DLAT**	0.17	1.12	3.69E-02	1.08E-01
**UQCRC2**	0.14	1.10	3.69E-02	1.08E-01
**EPS8**	0.45	1.36	3.70E-02	1.08E-01
**FAM122A**	-0.25	0.84	3.70E-02	1.08E-01
**HSPA14**	0.19	1.14	3.70E-02	1.08E-01
**CTD-3014M21.1**	1.27	2.41	3.70E-02	NA
**RAET1E-AS1**	-0.75	0.59	3.71E-02	1.08E-01
**YWHAB**	0.14	1.10	3.71E-02	1.08E-01
**SLTM**	0.16	1.12	3.72E-02	1.09E-01
**UMAD1**	0.30	1.23	3.73E-02	1.09E-01
**RPL23AP82**	-0.33	0.80	3.73E-02	1.09E-01
**IER5**	-0.15	0.90	3.74E-02	1.09E-01
**WDR7**	-0.20	0.87	3.74E-02	1.09E-01
**MPP2**	0.58	1.50	3.74E-02	1.09E-01
**RNF8**	0.18	1.14	3.75E-02	1.09E-01
**AKAP6**	-0.37	0.77	3.75E-02	1.09E-01
**TMEM120B**	-0.21	0.86	3.75E-02	1.09E-01
**CLIP2**	0.29	1.22	3.75E-02	1.09E-01
**HDDC3**	-0.45	0.73	3.75E-02	1.09E-01
**LINC00998**	-0.20	0.87	3.76E-02	1.09E-01
**FXYD3**	-0.16	0.90	3.76E-02	1.09E-01
**OTUB2**	0.33	1.26	3.76E-02	1.09E-01
**TM4SF1**	-0.19	0.88	3.77E-02	1.10E-01
**CARD10**	0.16	1.12	3.77E-02	1.10E-01
**GPAT3**	0.73	1.65	3.77E-02	1.10E-01
**TIPIN**	0.28	1.21	3.77E-02	1.10E-01
**TNK1**	-0.22	0.86	3.77E-02	1.10E-01
**PRELID1**	0.20	1.15	3.77E-02	1.10E-01
**RP11-582E3.6**	0.33	1.26	3.77E-02	1.10E-01
**GAN**	0.21	1.16	3.77E-02	1.10E-01
**DAP3**	-0.16	0.90	3.78E-02	1.10E-01
**WDR24**	-0.32	0.80	3.79E-02	1.10E-01
**KIF1B**	0.15	1.11	3.80E-02	1.10E-01
**IGF2BP2**	0.16	1.11	3.80E-02	1.11E-01
**SMIM20**	0.27	1.21	3.80E-02	1.11E-01
**ADAM17**	0.17	1.13	3.81E-02	1.11E-01
**TUFM**	0.14	1.10	3.81E-02	1.11E-01
**RP11-196G11.5**	-0.45	0.73	3.81E-02	1.11E-01
**SAMD8**	0.17	1.13	3.82E-02	1.11E-01
**NFS1**	-0.21	0.86	3.83E-02	1.11E-01
**AIDA**	0.19	1.14	3.84E-02	1.11E-01
**HS2ST1**	0.20	1.15	3.84E-02	1.11E-01
**RNF223**	0.51	1.43	3.84E-02	1.11E-01
**SLC45A1**	1.79	3.46	3.85E-02	NA
**ADAD2**	0.76	1.69	3.85E-02	1.12E-01
**HIATL1**	-0.15	0.90	3.85E-02	1.12E-01
**DCUN1D5**	0.18	1.13	3.85E-02	1.12E-01
**TMEM216**	-0.47	0.72	3.86E-02	1.12E-01
**FIBP**	-0.18	0.88	3.87E-02	1.12E-01
**LYPD6**	-0.24	0.85	3.88E-02	1.12E-01
**TRAF4**	0.16	1.11	3.88E-02	1.12E-01
**PPM1J**	-0.45	0.73	3.88E-02	1.12E-01
**ZNF385B**	-1.33	0.40	3.88E-02	NA
**FAM95C**	-0.64	0.64	3.89E-02	1.12E-01
**RP11-598P20.3**	-0.52	0.70	3.89E-02	1.12E-01
**MED25**	-0.18	0.88	3.89E-02	1.12E-01
**RFTN1**	0.20	1.15	3.89E-02	1.13E-01
**CSNK2A2**	-0.17	0.89	3.90E-02	1.13E-01
**FAM69A**	0.19	1.14	3.90E-02	1.13E-01
**RP11-511P7.5**	-0.65	0.64	3.91E-02	1.13E-01
**HES7**	0.60	1.51	3.92E-02	1.13E-01
**DHX57**	0.18	1.14	3.92E-02	1.13E-01

**KRT16P4**	-0.46	0.72	3.92E-02	1.13E-01
**TBRG4**	0.15	1.11	3.93E-02	1.14E-01
**RPS13**	-0.13	0.91	3.93E-02	1.14E-01
**NUAK2**	-0.19	0.88	3.94E-02	1.14E-01
**FAM107B**	0.36	1.28	3.94E-02	1.14E-01
**EP400**	-0.16	0.89	3.94E-02	1.14E-01
**SLMAP**	0.15	1.11	3.94E-02	1.14E-01
**TMTC4**	0.24	1.18	3.94E-02	1.14E-01
**ERCC3**	0.16	1.12	3.95E-02	1.14E-01
**USP9X**	0.14	1.10	3.96E-02	1.14E-01
**RIN1**	0.16	1.12	3.96E-02	1.14E-01
**SHF**	-0.48	0.71	3.96E-02	1.14E-01
**SPG20**	0.18	1.14	3.97E-02	1.14E-01
**SH3PXD2A-AS1**	-0.44	0.74	3.97E-02	1.14E-01
**CRK**	0.15	1.11	3.97E-02	1.14E-01
**PRSS12**	-0.17	0.89	3.97E-02	1.14E-01
**COA4**	-0.20	0.87	3.97E-02	1.14E-01
**NAGLU**	-0.22	0.86	3.97E-02	1.14E-01
**ILF3-AS1**	0.41	1.33	3.97E-02	1.14E-01
**DPY19L4**	0.20	1.15	3.98E-02	1.14E-01
**LINC00893**	-0.86	0.55	3.98E-02	1.14E-01
**ZNF697**	0.37	1.29	3.98E-02	1.14E-01
**ZNF726**	0.72	1.65	3.98E-02	1.14E-01
**TSPAN15**	0.42	1.34	3.98E-02	1.14E-01
**BORA**	0.31	1.24	3.98E-02	1.14E-01
**TLK2**	0.18	1.14	3.99E-02	1.15E-01
**SNAPC1**	0.31	1.24	4.00E-02	1.15E-01
**SAPCD2**	0.25	1.19	4.00E-02	1.15E-01
**LY6E**	0.14	1.10	4.00E-02	1.15E-01
**ATXN1**	0.18	1.14	4.00E-02	1.15E-01
**AP001468.1**	-0.56	0.68	4.00E-02	1.15E-01
**FBXO28**	0.17	1.13	4.00E-02	1.15E-01
**QRICH1**	0.15	1.11	4.00E-02	1.15E-01
**MT1F**	0.64	1.56	4.00E-02	1.15E-01
**PPFIBP2**	0.24	1.18	4.01E-02	1.15E-01
**HERC1**	-0.15	0.90	4.01E-02	1.15E-01
**LINC00680**	-0.54	0.69	4.01E-02	1.15E-01
**TRAPPC4**	0.20	1.15	4.02E-02	1.15E-01
**FHDC1**	-0.20	0.87	4.02E-02	1.15E-01
**TRIP12**	0.14	1.10	4.02E-02	1.15E-01
**PHACTR2**	0.20	1.15	4.02E-02	1.15E-01
**LRFN1**	0.88	1.84	4.02E-02	1.15E-01
**RNASEH2CP1**	-0.81	0.57	4.02E-02	1.15E-01
**DMC1**	0.84	1.79	4.03E-02	1.15E-01
**NKX3-1**	0.68	1.60	4.03E-02	1.15E-01
**ZNF492**	0.61	1.52	4.04E-02	1.16E-01
**ATP2B1**	0.17	1.12	4.04E-02	1.16E-01
**G3BP2**	0.15	1.11	4.04E-02	1.16E-01
**FAR2**	0.39	1.31	4.05E-02	1.16E-01
**EXOSC9**	0.22	1.17	4.05E-02	1.16E-01
**FTX**	-0.63	0.65	4.05E-02	1.16E-01
**BRAT1**	0.16	1.12	4.05E-02	1.16E-01
**ZDHHC3**	0.15	1.11	4.06E-02	1.16E-01
**MIR621**	0.51	1.43	4.06E-02	1.16E-01
**H1FX**	0.20	1.15	4.06E-02	1.16E-01
**RIT1**	0.20	1.15	4.07E-02	1.16E-01
**LINC00174**	-0.44	0.74	4.07E-02	1.16E-01
**RP11-429J17.8**	-1.76	0.30	4.08E-02	NA
**ARHGAP30**	-0.47	0.72	4.09E-02	1.17E-01
**MID2**	-0.30	0.81	4.09E-02	1.17E-01
**HIST1H3G**	1.81	3.50	4.09E-02	NA
**RP11-150O12.6**	1.99	3.97	4.09E-02	NA
**RNGTT**	0.20	1.14	4.10E-02	1.17E-01
**C19orf12**	-0.25	0.84	4.10E-02	1.17E-01
**JADE3**	0.24	1.18	4.10E-02	1.17E-01
**IRX4**	0.20	1.14	4.10E-02	1.17E-01
**RPP30**	0.22	1.17	4.10E-02	1.17E-01
**TAF10**	-0.19	0.88	4.10E-02	1.17E-01
**RUSC1**	-0.17	0.89	4.11E-02	1.17E-01
**LATS2**	0.21	1.16	4.11E-02	1.17E-01
**PDHA1**	0.15	1.11	4.11E-02	1.17E-01
**LYSMD3**	0.20	1.15	4.11E-02	1.17E-01
**HOMER2**	0.19	1.14	4.11E-02	1.17E-01
**AP006621.5**	-0.56	0.68	4.11E-02	1.17E-01
**PAIP1**	0.17	1.13	4.12E-02	1.17E-01
**MKL1**	-0.16	0.90	4.12E-02	1.17E-01
**MEIS1**	-0.24	0.85	4.12E-02	1.17E-01
**ACTG1**	-0.16	0.90	4.13E-02	1.17E-01
**ATP5D**	0.16	1.12	4.14E-02	1.18E-01
**SMYD5**	-0.23	0.85	4.14E-02	1.18E-01
**RAVER2**	0.20	1.15	4.14E-02	1.18E-01
**ARHGAP23**	0.14	1.10	4.15E-02	1.18E-01
**DBF4B**	-0.25	0.84	4.15E-02	1.18E-01
**FOXK2**	-0.15	0.90	4.16E-02	1.18E-01
**RP11-156E6.1**	0.33	1.26	4.16E-02	1.18E-01
**RAMP1**	0.45	1.36	4.16E-02	1.18E-01
**RTF1**	0.15	1.11	4.16E-02	1.18E-01
**NOL12**	-0.46	0.73	4.16E-02	1.18E-01
**CCDC113**	0.25	1.19	4.17E-02	1.18E-01
**USP45**	0.22	1.16	4.18E-02	1.18E-01

TULP3	-0.21	0.86	4.19E-02	1.19E-01
CCAR1	0.15	1.11	4.19E-02	1.19E-01
VPRBP	0.18	1.13	4.19E-02	1.19E-01
RAD23B	0.13	1.10	4.19E-02	1.19E-01
CORO6	0.23	1.17	4.21E-02	1.19E-01
OARD1	-0.24	0.85	4.21E-02	1.19E-01
FAM155B	-0.83	0.56	4.21E-02	1.19E-01
ERMARD	0.21	1.16	4.22E-02	1.19E-01
POLRMT	0.18	1.13	4.22E-02	1.20E-01
SLC7A11	0.21	1.15	4.23E-02	1.20E-01
YTHDC2	0.16	1.11	4.23E-02	1.20E-01
UTP15	0.23	1.17	4.23E-02	1.20E-01
GNG2	0.74	1.66	4.23E-02	1.20E-01
PRKAR2A-AS1	-1.01	0.50	4.23E-02	NA
05-sep	-0.31	0.81	4.24E-02	1.20E-01
HIC2	0.26	1.20	4.25E-02	1.20E-01
RP11-274B21.2	-0.30	0.81	4.25E-02	1.20E-01
SEC61B	-0.16	0.89	4.26E-02	1.20E-01
ST6GALNAC2	-0.16	0.90	4.26E-02	1.20E-01
MAZ	-0.28	0.82	4.26E-02	1.20E-01
MLLT3	-0.29	0.82	4.26E-02	1.20E-01
L3MBTL2	0.20	1.15	4.27E-02	1.20E-01
ZNF133	-0.21	0.87	4.27E-02	1.20E-01
PPP1R3E	-0.43	0.74	4.27E-02	1.21E-01
ISYNA1	0.27	1.20	4.27E-02	1.21E-01
ITGB8	0.15	1.11	4.28E-02	1.21E-01
DDX56	0.15	1.11	4.28E-02	1.21E-01
TENM4	0.18	1.13	4.28E-02	1.21E-01
CCT6P1	-0.53	0.69	4.28E-02	1.21E-01
FOSL1	-0.22	0.86	4.29E-02	1.21E-01
NAPEPLD	0.24	1.18	4.30E-02	1.21E-01
DNAJC4	-0.29	0.82	4.30E-02	1.21E-01
DDX42	0.14	1.10	4.30E-02	1.21E-01
SRPR	0.14	1.10	4.30E-02	1.21E-01
KIFAP3	0.20	1.15	4.30E-02	1.21E-01
ZNF281	0.19	1.14	4.30E-02	1.21E-01
RPS27	-0.13	0.91	4.31E-02	1.21E-01
RP11-196G18.22	0.48	1.40	4.31E-02	1.21E-01
SCOC	0.15	1.11	4.32E-02	1.22E-01
RGS10	0.23	1.17	4.33E-02	1.22E-01
TMEM38B	0.26	1.20	4.33E-02	1.22E-01
MED13	0.15	1.11	4.33E-02	1.22E-01
GHITM	0.14	1.10	4.34E-02	1.22E-01
FAM96B	-0.17	0.89	4.34E-02	1.22E-01
YPEL2	-0.34	0.79	4.34E-02	1.22E-01
FLAD1	-0.17	0.89	4.34E-02	1.22E-01
MFF	0.16	1.12	4.34E-02	1.22E-01
MT2A	0.50	1.41	4.34E-02	1.22E-01
YIPF4	0.18	1.13	4.35E-02	1.22E-01
GOLGA2	-0.15	0.90	4.35E-02	1.22E-01
TNPO1P3	-0.90	0.54	4.37E-02	1.23E-01
UBE2SP1	0.64	1.56	4.37E-02	1.23E-01
GLTP	-0.18	0.88	4.38E-02	1.23E-01
SDC3	0.17	1.13	4.38E-02	1.23E-01
TYW3	0.19	1.14	4.38E-02	1.23E-01
PDLIM2	-0.18	0.89	4.38E-02	1.23E-01
RP11-221N13.3	-0.86	0.55	4.39E-02	1.23E-01
GPRASP2	0.33	1.26	4.40E-02	1.23E-01
C1RL-AS1	-0.29	0.82	4.41E-02	1.23E-01
NR2F6	0.18	1.13	4.41E-02	1.23E-01
10-sep	-0.15	0.90	4.43E-02	1.24E-01
ALDH7A1	-0.14	0.91	4.43E-02	1.24E-01
CCDC85B	0.26	1.20	4.44E-02	1.24E-01
ERVMER34-1	0.28	1.21	4.44E-02	1.24E-01
NSA2	0.15	1.11	4.45E-02	1.25E-01
TSKU	0.16	1.12	4.45E-02	1.25E-01
KCNG1	-0.22	0.86	4.46E-02	1.25E-01
SNAI2	-0.15	0.90	4.46E-02	1.25E-01
STAR	0.94	1.92	4.46E-02	NA
LYNX1	-0.28	0.83	4.47E-02	1.25E-01
KPRP	-0.55	0.68	4.47E-02	1.25E-01
LIF	-0.74	0.60	4.48E-02	1.25E-01
GPR107	0.14	1.10	4.49E-02	1.25E-01
TRIM56	0.14	1.11	4.49E-02	1.25E-01
EVI5	-0.22	0.86	4.49E-02	1.25E-01
SLC29A3	0.66	1.58	4.49E-02	1.25E-01
PFDN4	0.27	1.21	4.49E-02	1.25E-01
PLPP3	0.42	1.34	4.51E-02	1.26E-01
MPP7	-0.19	0.88	4.52E-02	1.26E-01
JAK2	-0.26	0.83	4.52E-02	1.26E-01
AOX1	-0.33	0.80	4.52E-02	1.26E-01
MEX3C	0.17	1.13	4.52E-02	1.26E-01
RP11-33B1.1	-0.37	0.77	4.53E-02	1.26E-01
C11orf68	-0.22	0.86	4.53E-02	1.26E-01
IGSF8	0.16	1.11	4.54E-02	1.27E-01
DANCR	-0.21	0.86	4.54E-02	1.27E-01
NOSIP	0.20	1.15	4.55E-02	1.27E-01
TMEM184C	-0.18	0.88	4.55E-02	1.27E-01
RAE1	0.18	1.13	4.55E-02	1.27E-01
CDYL	0.19	1.14	4.56E-02	1.27E-01

**GRTP1**	-0.25	0.84	4.56E-02	1.27E-01
**VSIG10**	0.17	1.12	4.57E-02	1.27E-01
**IFT81**	0.29	1.22	4.57E-02	1.27E-01
**MTFR1**	0.18	1.14	4.57E-02	1.27E-01
**KLK11**	-0.16	0.89	4.58E-02	1.28E-01
**CSNK1G3**	0.18	1.13	4.59E-02	1.28E-01
**LYAR**	0.24	1.18	4.59E-02	1.28E-01
**MMADHC**	0.15	1.11	4.60E-02	1.28E-01
**EREG**	-0.38	0.77	4.60E-02	1.28E-01
**ZDHHC2**	0.24	1.18	4.61E-02	1.28E-01
**ELOVL1**	-0.15	0.90	4.61E-02	1.28E-01
**CCDC109B**	0.24	1.18	4.61E-02	1.28E-01
**ANGEL2**	0.19	1.14	4.63E-02	1.28E-01
**REC8**	-0.30	0.81	4.63E-02	1.28E-01
**SPHK1**	-0.23	0.85	4.63E-02	1.29E-01
**TP73**	0.48	1.39	4.63E-02	1.29E-01
**GATB**	0.18	1.13	4.64E-02	1.29E-01
**DFFA**	0.15	1.11	4.64E-02	1.29E-01
**PSMA7**	0.14	1.10	4.64E-02	1.29E-01
**AK6**	0.28	1.22	4.64E-02	1.29E-01
**DYNC2LI1**	0.24	1.18	4.65E-02	1.29E-01
**WNT7B**	0.64	1.55	4.65E-02	1.29E-01
**FAM114A1**	0.17	1.13	4.65E-02	1.29E-01
**DYNLT3**	-0.21	0.87	4.65E-02	1.29E-01
**MRPL45**	0.18	1.13	4.66E-02	1.29E-01
**SORD**	0.17	1.13	4.66E-02	1.29E-01
**IMPDH1**	-0.43	0.74	4.67E-02	1.29E-01
**TMED8**	-0.20	0.87	4.68E-02	1.29E-01
**USH1G**	-0.35	0.78	4.68E-02	1.30E-01
**RP11-140K17.3**	-1.01	0.50	4.69E-02	NA
**SLC16A14**	0.32	1.25	4.70E-02	1.30E-01
**TTL**	0.16	1.12	4.70E-02	1.30E-01
**IDS**	-0.17	0.89	4.70E-02	1.30E-01
**WNT7A**	-0.19	0.87	4.71E-02	1.30E-01
**SERINC3**	0.14	1.10	4.71E-02	1.30E-01
**MGAT4B**	0.14	1.10	4.71E-02	1.30E-01
**TAGAP**	-1.02	0.49	4.71E-02	NA
**STX1A**	-0.48	0.72	4.71E-02	1.30E-01
**NUP133**	0.16	1.12	4.72E-02	1.30E-01
**ALDOA**	0.12	1.09	4.72E-02	1.30E-01
**C1orf210**	-0.31	0.81	4.72E-02	1.30E-01
**PUM3**	0.16	1.12	4.73E-02	1.31E-01
**SPAST**	0.22	1.16	4.74E-02	1.31E-01
**DENR**	0.15	1.11	4.75E-02	1.31E-01
**BCAS2**	0.19	1.14	4.77E-02	1.32E-01
**MRPL48**	0.23	1.17	4.77E-02	1.32E-01
**PSMC1**	0.18	1.14	4.77E-02	1.32E-01
**AGO1**	0.17	1.12	4.78E-02	1.32E-01
**RP4-756H11.5**	-0.49	0.71	4.78E-02	1.32E-01
**PRTFDC1**	0.35	1.28	4.79E-02	1.32E-01
**ZNF318**	0.16	1.12	4.79E-02	1.32E-01
**MCAT**	0.23	1.17	4.79E-02	1.32E-01
**DDX23**	0.14	1.10	4.79E-02	1.32E-01
**PPP2R2C**	0.31	1.24	4.80E-02	1.32E-01
**DISP2**	-0.94	0.52	4.80E-02	NA
**MIR503**	-1.43	0.37	4.81E-02	NA
**PCSK1**	1.01	2.01	4.81E-02	NA
**CEACAM1**	-0.32	0.80	4.81E-02	1.33E-01
**GOLGA4**	0.14	1.10	4.82E-02	1.33E-01
**CHD9**	0.14	1.11	4.82E-02	1.33E-01
**RNF135**	-0.22	0.86	4.83E-02	1.33E-01
**CHTF18**	0.22	1.17	4.83E-02	1.33E-01
**DCAF17**	-0.20	0.87	4.83E-02	1.33E-01
**RP3-475N16.1**	-1.46	0.36	4.83E-02	NA
**CAPRIN2**	0.24	1.18	4.84E-02	1.33E-01
**HOXB3**	4.23	18.79	4.84E-02	NA
**SYNJ1**	0.21	1.16	4.85E-02	1.33E-01
**RNF220**	0.18	1.13	4.85E-02	1.33E-01
**ZZZ3**	0.17	1.13	4.85E-02	1.33E-01
**PCBP1-AS1**	-0.45	0.73	4.85E-02	1.33E-01
**MUC2**	-1.37	0.39	4.86E-02	NA
**RP1-152L7.5**	0.89	1.86	4.86E-02	NA
**VEZF1**	0.21	1.16	4.86E-02	1.34E-01
**KLK5**	-0.14	0.91	4.86E-02	1.34E-01
**SGSM3**	-0.15	0.90	4.87E-02	1.34E-01
**TMTC2**	0.23	1.17	4.88E-02	1.34E-01
**EXOC6**	0.42	1.34	4.88E-02	1.34E-01
**MAP1A**	-0.58	0.67	4.88E-02	1.34E-01
**NPHP4**	0.21	1.15	4.88E-02	1.34E-01
**PPP1R2**	0.19	1.14	4.88E-02	1.34E-01
**KLF9**	0.24	1.18	4.89E-02	1.34E-01
**CD55**	0.29	1.22	4.89E-02	1.34E-01
**GADD45GIP1**	0.18	1.13	4.89E-02	1.34E-01
**UVRAG**	0.22	1.16	4.89E-02	1.34E-01
**NBR1**	-0.15	0.90	4.89E-02	1.34E-01
**STX19**	-0.67	0.63	4.89E-02	1.34E-01
**BMPR2**	-0.14	0.91	4.90E-02	1.34E-01
**ZZEF1**	-0.15	0.90	4.90E-02	1.34E-01
**ABI3BP**	-0.28	0.82	4.90E-02	1.34E-01
**KLHL11**	0.39	1.31	4.91E-02	1.34E-01

**SF1**	0.13	1.09	4.91E-02	1.34E-01
**CWF19L2**	0.24	1.18	4.91E-02	1.34E-01
**SLC4A1AP**	0.18	1.13	4.91E-02	1.34E-01
**RBM22**	-0.18	0.88	4.91E-02	1.34E-01
**RP11-400F19.18**	-1.20	0.44	4.92E-02	NA
**SLC4A3**	-0.19	0.88	4.92E-02	1.35E-01
**SCRIB**	-0.15	0.90	4.92E-02	1.35E-01
**NDUFB4**	0.16	1.12	4.93E-02	1.35E-01
**SPCS2**	0.18	1.13	4.93E-02	1.35E-01
**NOD1**	0.27	1.20	4.93E-02	1.35E-01
**NFE2L2**	-0.14	0.91	4.93E-02	1.35E-01
**LRRFIP2**	-0.14	0.91	4.93E-02	1.35E-01
**MMP25-AS1**	-0.53	0.69	4.94E-02	1.35E-01
**MPDZ**	0.26	1.20	4.94E-02	1.35E-01
**VANGL2**	0.15	1.11	4.95E-02	1.35E-01
**TNFRSF10A**	-0.21	0.86	4.95E-02	1.35E-01
**POMGNT2**	0.22	1.16	4.95E-02	1.35E-01
**RP13-516M14.1**	0.79	1.72	4.96E-02	1.35E-01
**CNOT6L**	0.17	1.12	4.96E-02	1.35E-01
**SMC1A**	0.16	1.11	4.96E-02	1.35E-01
**RC3H2**	0.14	1.11	4.96E-02	1.35E-01
**SAMHD1**	0.17	1.12	4.97E-02	1.35E-01
**RALGAPB**	-0.15	0.90	4.97E-02	1.36E-01
**ABI1**	0.15	1.11	4.97E-02	1.36E-01
**OCIAD2**	0.16	1.12	4.97E-02	1.36E-01
**LRFN4**	0.30	1.23	4.99E-02	1.36E-01
**TTC31**	-0.19	0.88	4.99E-02	1.36E-01
**LONRF1**	0.28	1.21	4.99E-02	1.36E-01
**DNAJC16**	0.18	1.13	4.99E-02	1.36E-01
