# Supplementary material for: Sumo-regulatory SENP2 controls the homeostatic squamous mitosis-differentiation checkpoint
Source: Cell Death Dis. 2024 Aug 16;15(8):596. doi: 10.1038/s41419-024-06969-z (PMC11329632; doi:10.1038/s41419-024-06969-z)
Supplement: Supplementary file 4 — Original western blots [file 41419_2024_6969_MOESM4_ESM.pdf]

Sumo-regulatory SENP2 controls the homeostatic squamous mitosis-differentiation checkpoint.

Galán et al

ORIGINAL WESTERN BLOTS

Figure 2J

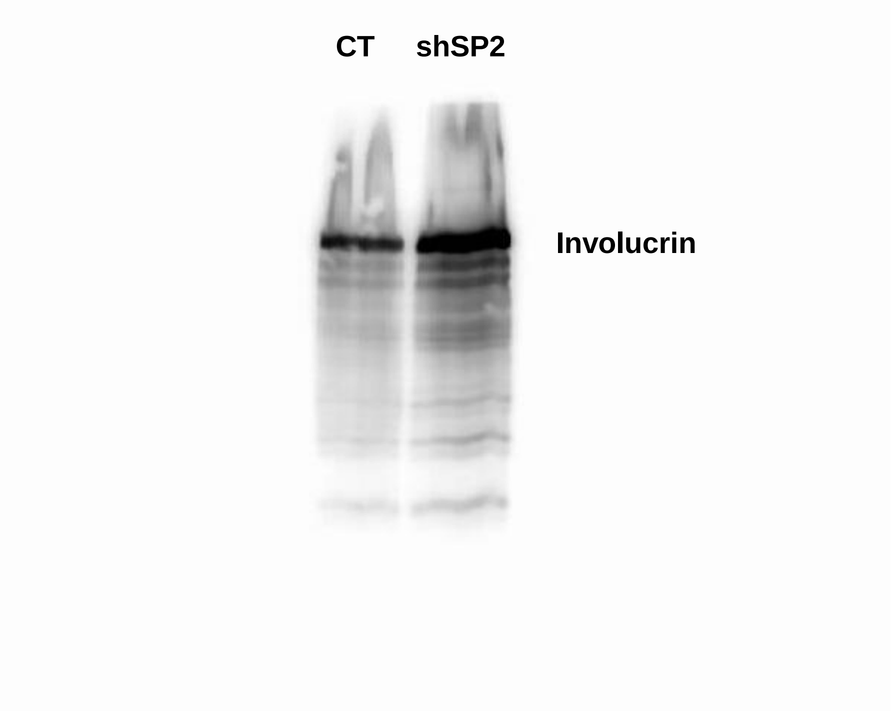

Figure 3G

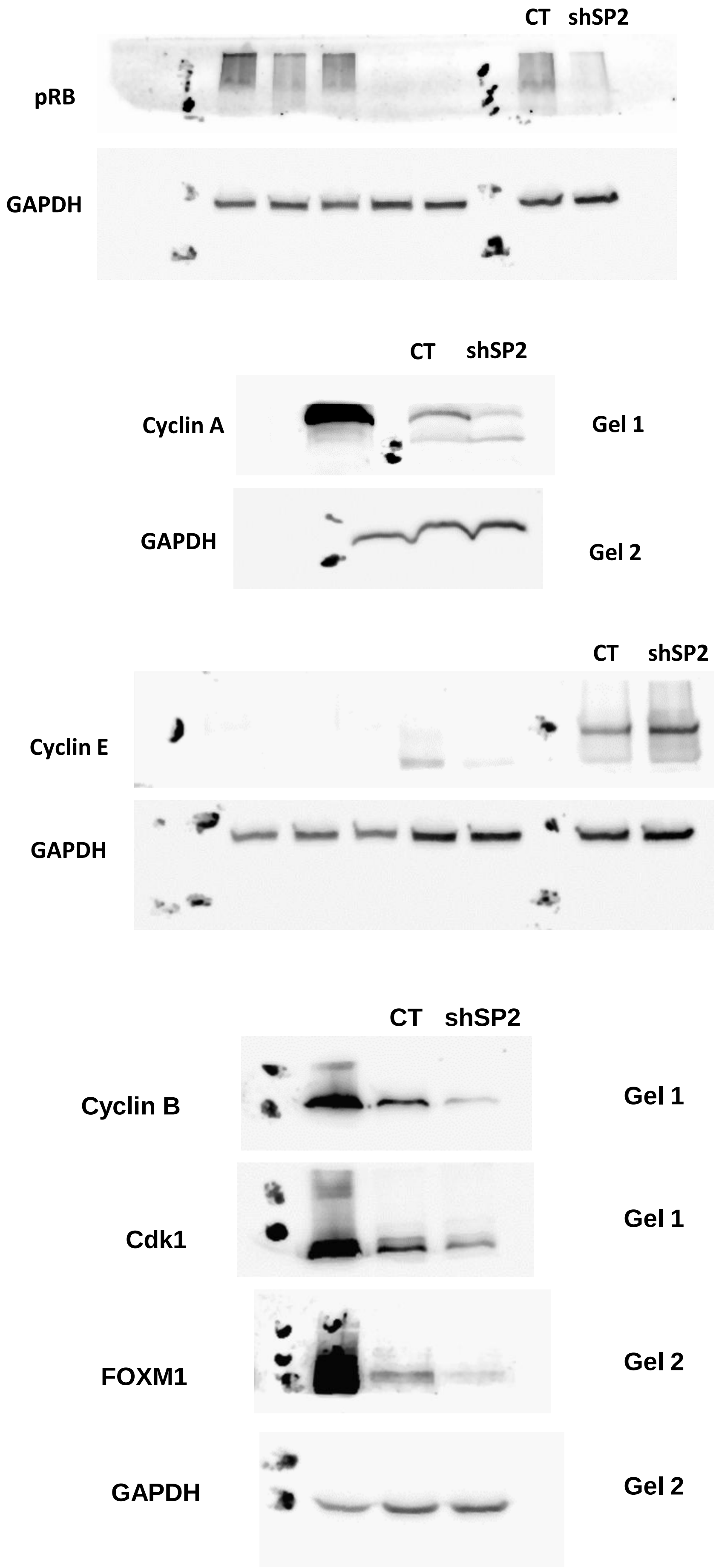
CT
shSP2
pRB
GAPDH
CT
shSP2
Cyclin A
Gel 1
GAPDH
Gel 2
CT
shSP2
Cyclin E
GAPDH
CT
shSP2
Cyclin B
Gel 1
Cdk1
Gel 1
FOXM1
Gel 2
GAPDH
Gel 2

Figure 3G

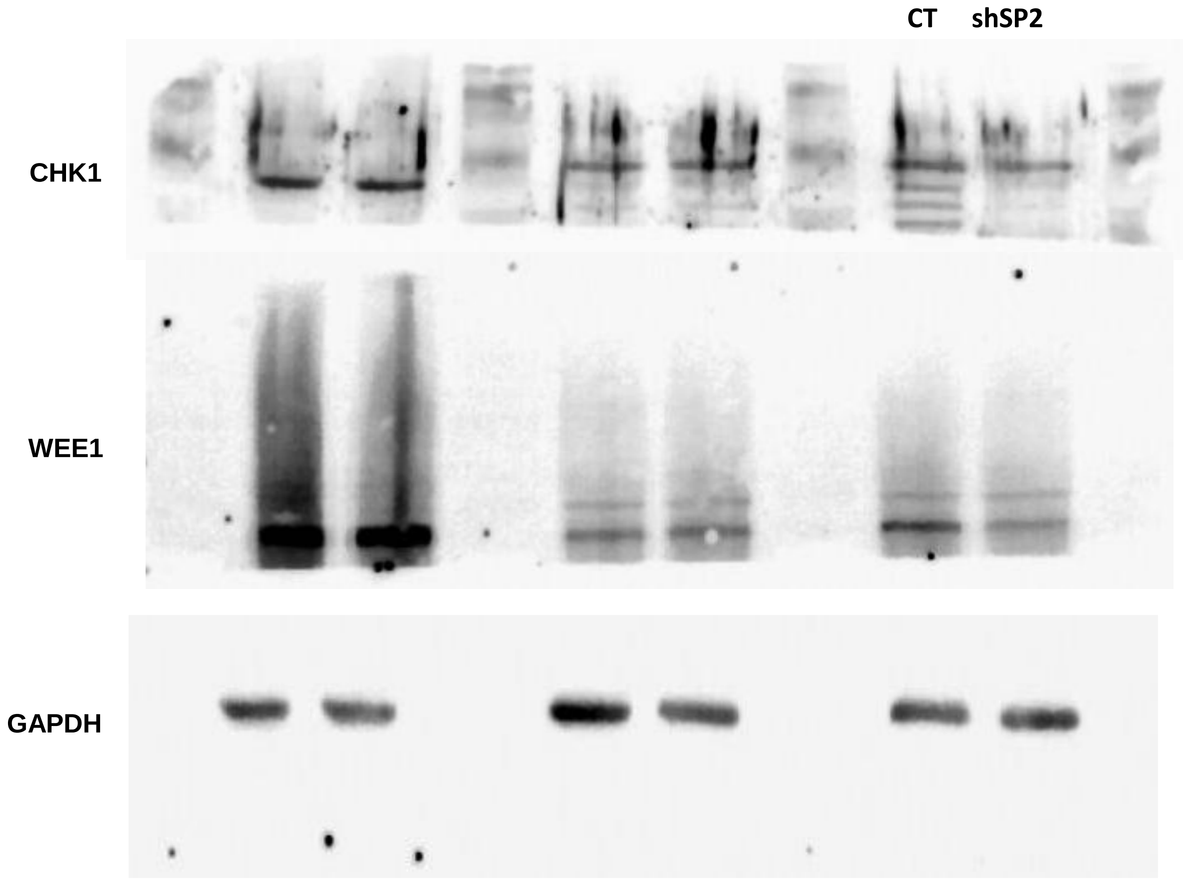

Figure 4B

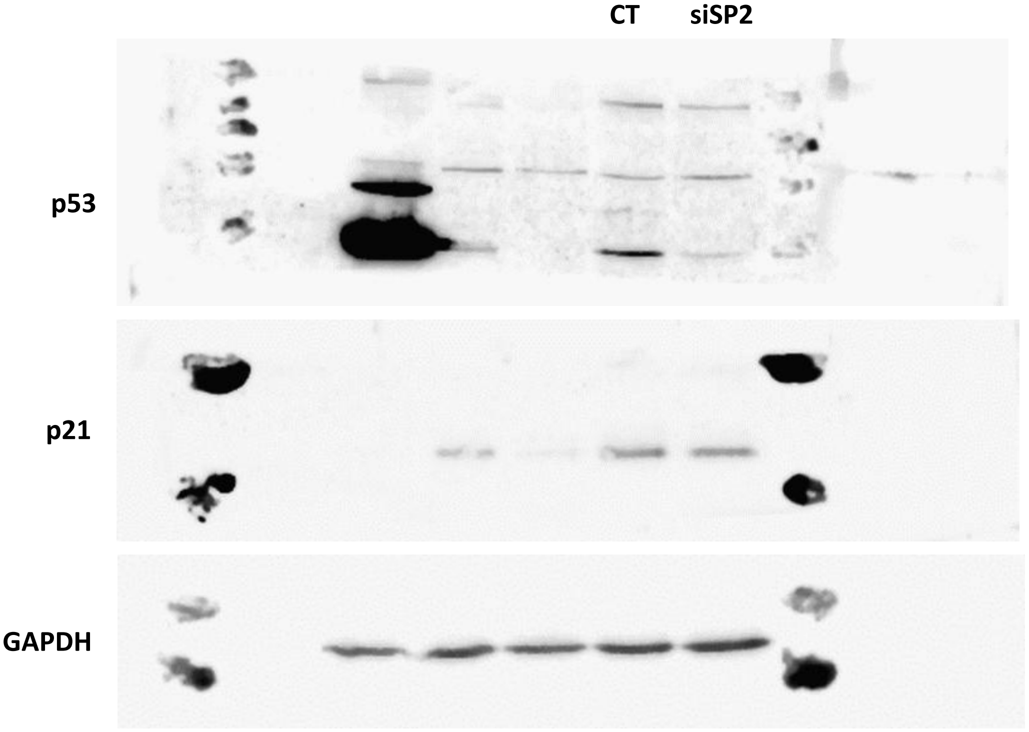

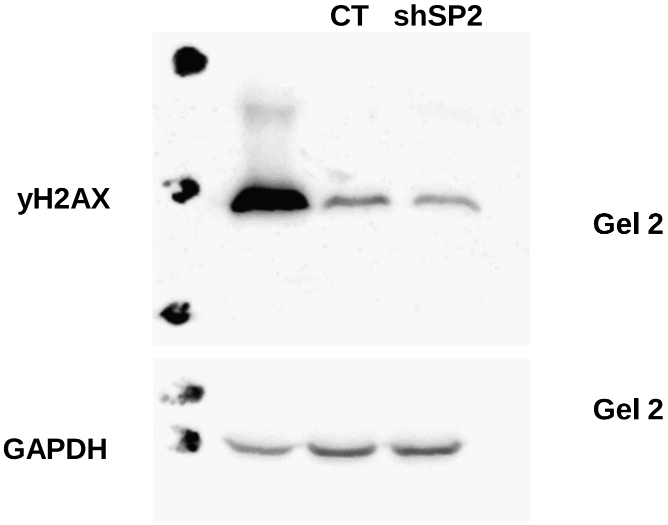

Figure 4D

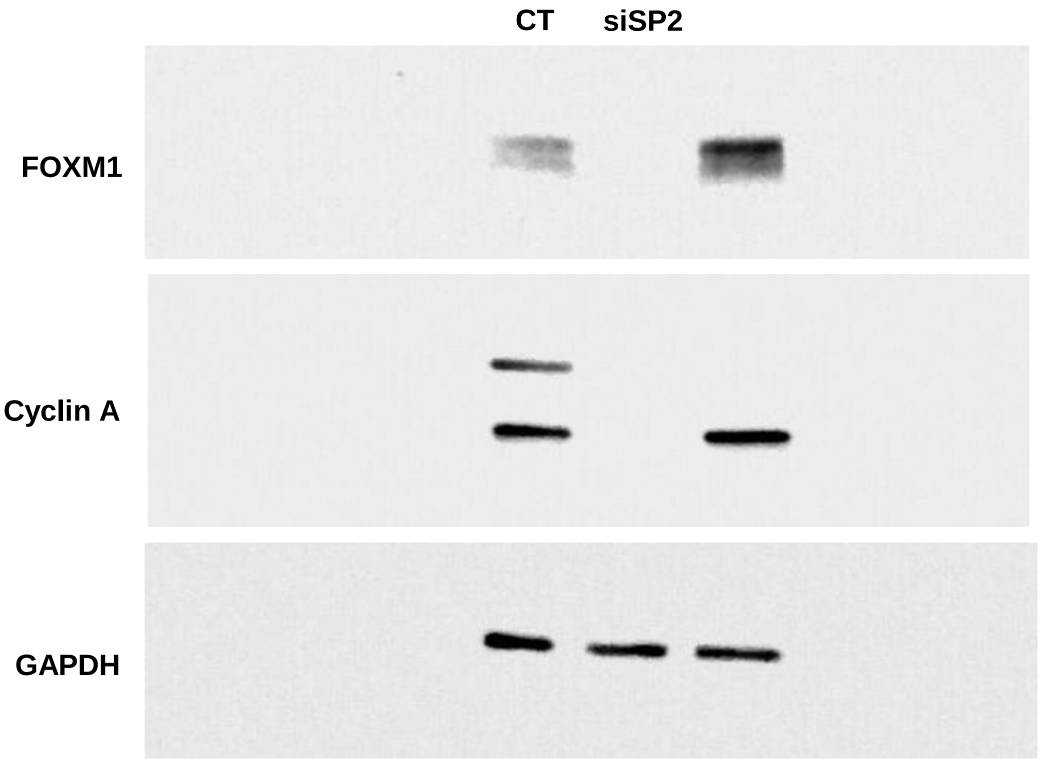

Figure 6E

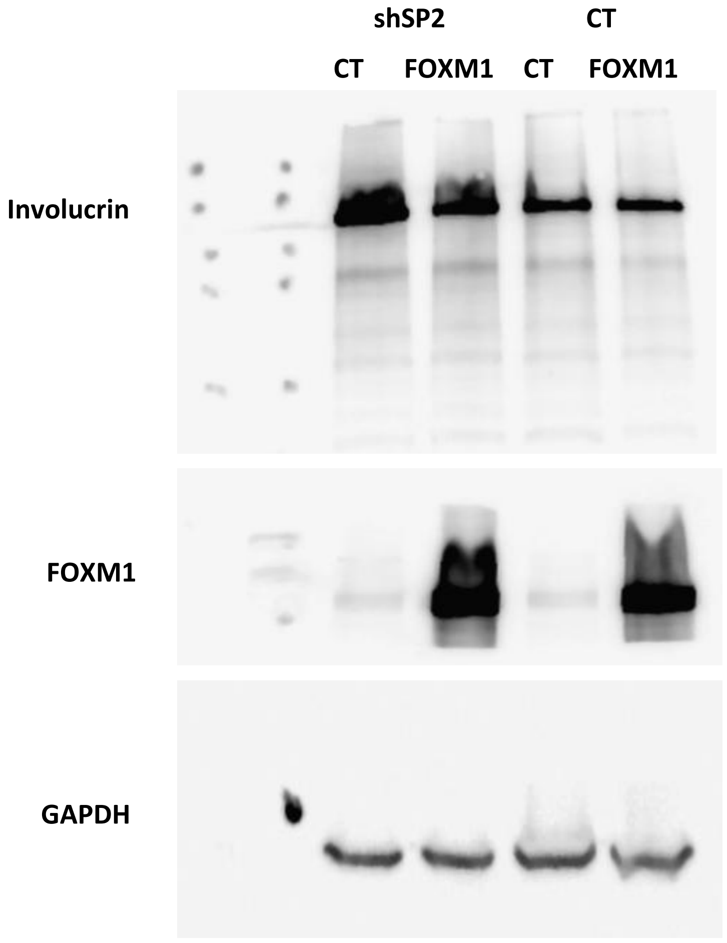

Figure 6F

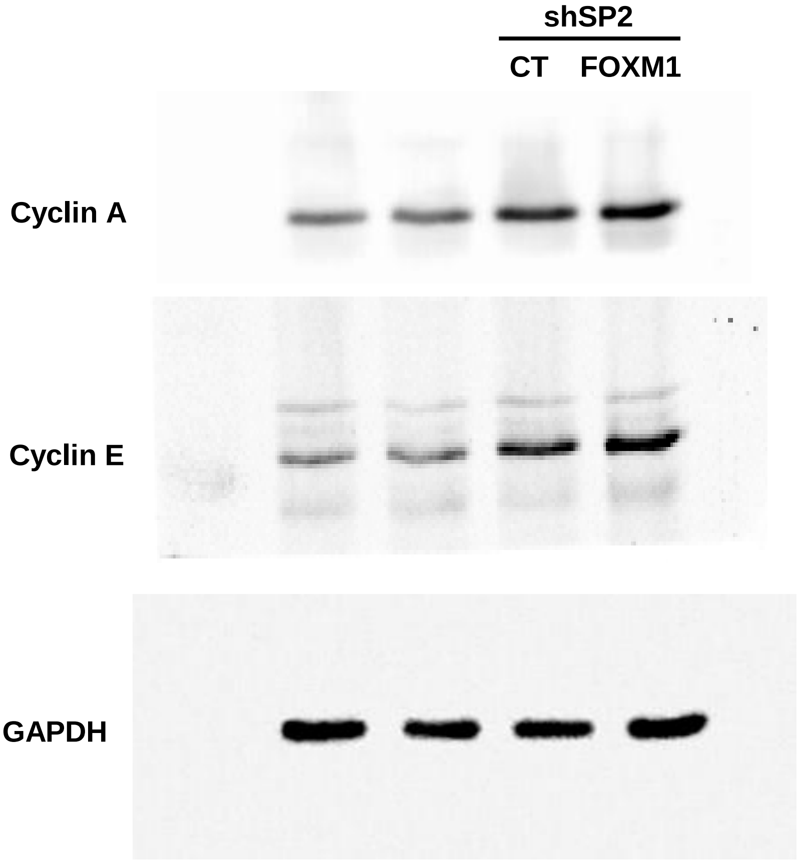
shSP2
CT
FOXM1
Cyclin A
Cyclin E
GAPDH

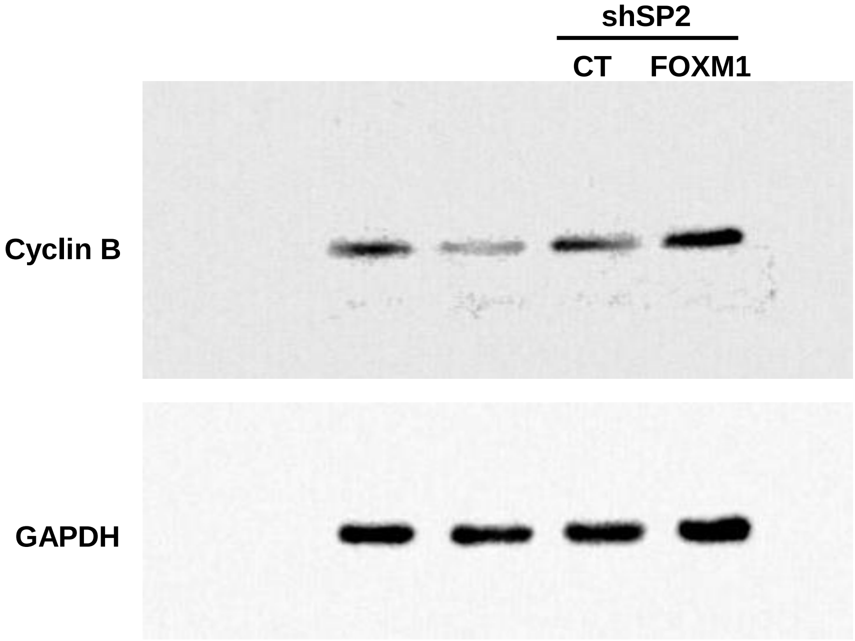
shSP2
CT
FOXM1
Cyclin B
GAPDH
